# Ancient Eukaryotic Origin and Evolutionary Plasticity of Nuclear Lamina

**DOI:** 10.1093/gbe/evw087

**Published:** 2016-04-28

**Authors:** Ludek Koreny, Mark C. Field

**Affiliations:** School of Life Sciences, University of Dundee, Dundee, UK

**Keywords:** lamina, lamins, evolution, origin of the nucleus, eukaryogenesis, nuclear structure, nuclear organization, heterochromatin

## Abstract

The emergence of the nucleus was a major event of eukaryogenesis. How the nuclear envelope (NE) arose and acquired functions governing chromatin organization and epigenetic control has direct bearing on origins of developmental/stage-specific expression programs. The configuration of the NE and the associated lamina in the last eukaryotic common ancestor (LECA) is of major significance and can provide insight into activities within the LECA nucleus. Subsequent lamina evolution, alterations, and adaptations inform on the variation and selection of distinct mechanisms that subtend gene expression in distinct taxa. Understanding lamina evolution has been difficult due to the diversity and limited taxonomic distributions of the three currently known highly distinct nuclear lamina. We rigorously searched available sequence data for an expanded view of the distribution of known lamina and lamina-associated proteins. While the lamina proteins of plants and trypanosomes are indeed taxonomically restricted, homologs of metazoan lamins and key lamin-binding proteins have significantly broader distributions, and a lamin gene tree supports vertical evolution from the LECA. Two protist lamins from highly divergent taxa target the nucleus in mammalian cells and polymerize into filamentous structures, suggesting functional conservation of distant lamin homologs. Significantly, a high level of divergence of lamin homologs within certain eukaryotic groups and the apparent absence of lamins and/or the presence of seemingly different lamina proteins in many eukaryotes suggests great evolutionary plasticity in structures at the NE, and hence mechanisms of chromatin tethering and epigenetic gene control.

The nuclear lamina is a filamentous structure consisting of coiled-coil proteins associated with the inner nuclear membrane (INM) of the nuclear envelope (NE). This structure influences nuclear morphology and acts as a platform organizing chromatin and hence regulating gene expression (Dechat et al. 2008). The best characterized constituent proteins of the nuclear lamina are the animal lamins, type V intermediate filaments (IF) (Dechat et al. 2008). However, distinct coiled-coil proteins of substantially greater size have been described as major lamina constituents in distantly related eukaryotes: NUP-1 in trypanosomes and NMCP proteins in plants (DuBois et al. 2012; Ciska et al. 2013). The previously known phylogenetic distribution of lamins and other IF proteins is very limited, originally believed restricted to animals, but more recently expanded to a few protists related to metazoa and only two more distant lineages: several species of Oomycetes (belong to Stramenopiles) and one partial mRNA sequence corresponding to an IF domain in a Rhizarian (Krüger et al. 2012; Kollmar 2015). This taxonomically limited distribution does not discriminate between vertical evolution and possible horizontal gene transfer (HGT) events and together with the discoveries of the seemingly distinct lamina proteins in plants and trypanosomes has obscured defining an ancestral state for the eukaryotic lamina. To understand the lamina origin and evolution, we investigated the phylogenetic distribution of the known lamina proteins.

## Identification of Lamina Proteins

**Fig. 1.—:**
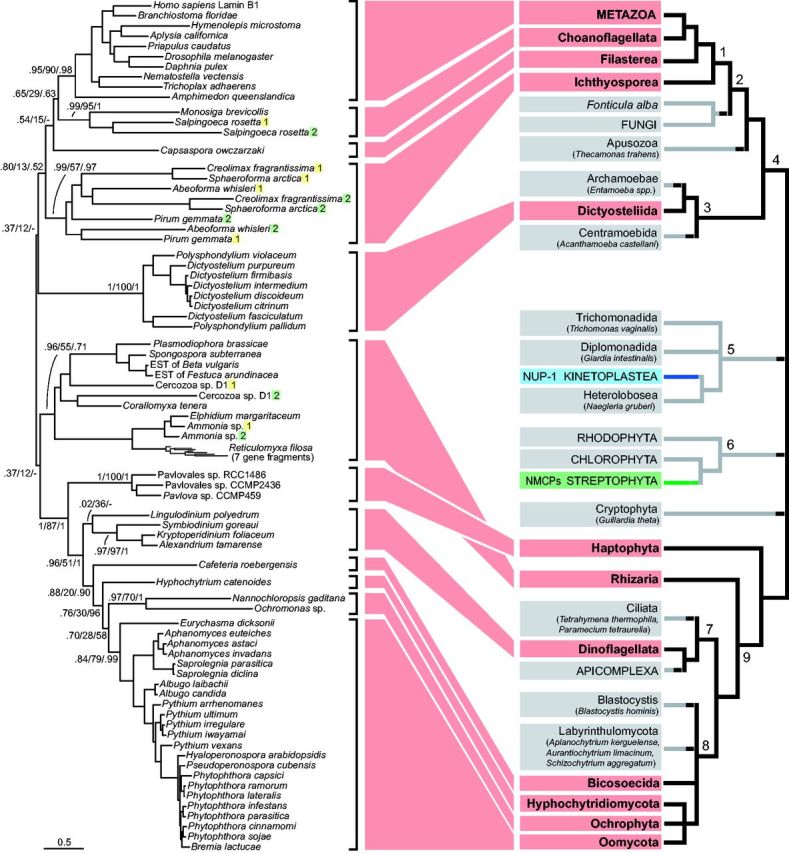
The phylogeny of lamins reflects the eukaryote species topology. A maximum likelihood tree of the lamin homologs (left) is compared with a schematic tree of eukaryotic taxa with available genome sequences, and represents the current view of eukaryotic phylogeny (right). Numbers above branches are PhyML SH-like approximate likelihood-ratio test/bootstrap/Bayesian posterior probability values. The duplicated lamin genes are distinguished by number and each copy is highlighted with yellow or green. In case of metazoa, only the B-type lamin homologs were used for phylogenetic inference. For details on the gene duplications that led to expansion of IF proteins in metazoa, see the recent phylogenetic analysis in Kollmar (2015). Species names are listed in brackets for taxa with a limited number of sequenced genomes (≤3). Lineages with identifiable lamin homologs are highlighted in red. The distribution of distinct lamina systems, that is NUP-1 in trypanosomatids and NMCPs in plants, is highlighted in blue and green, respectively. The numbers of branches on the tree of eukaryotes stand for higher order taxa: 1—Holozoa, 2—Opisthokonta, 3—Amoebozoa, 4—Amorphea (or Opimoda, former Unikonts), 5—Excavata, 6—Archaeplastida (or Plantae), 7—Alveolata, 8—Stramenopiles, 9—SAR clade.

According to our BLAST and HMMER iterative homology searches, the phylogenetic distributions of NUP-1 and NMCPs are very restricted. NUP-1 is apparently limited to Trypanosomatida (fig. 1 and supplementary fig. S1A, Supplementary Material online). However, the inability to detect NUP-1 elsewhere is unsurprising due to the extremely high sequence divergence even among trypanosomatids and leaves open the possibility that NUP-1 homologs are more broadly distributed. The NMCPs were previously reported as land plant (Embryophyta) restricted (Ciska et al. 2013), but here we identified homologs in charophyte algae (supplementary fig. S1A, Supplementary Material online). Significantly, low sequence conservation between NMCPs from even closely related taxa suggests that, similar to NUP-1, NMCP distribution may be broader, but current tools are unable to detect any such putative divergent homologs. The major issue with identifying distant homologs of coiled-coil proteins in general is that certain amino acids are favored depending on their position within the heptad repeats of the α-helix to enable the coiled-coil interaction with the other chain(s). Consequently, there is a certain level of sequence similarity even between coiled-coil proteins with presumed independent origins (*e*-values typically between 1E−04 and 1E−07). Thus, proteins that diverged to this level of similarity cannot be unambiguously determined as homologs, even if they were in fact closely related. Furthermore, coiled-coil filaments are generally prone to rapid evolution (Fleury-Aubusson 2003; Gould et al. 2011; Holden et al. 2014), likely due to few interactions with other proteins within a cell (Fleury-Aubusson 2003) as well as the low-sequence complexity within coiled-coil regions. As a result, coiled-coil proteins belonging to the same family often display similar levels of divergence as that between proteins from unrelated families and this makes identification of distant homologs of any coiled-coil protein a difficult task. This issue concerns both NUP-1 and NMCPs as they lack other distinctive or discriminatory conserved domains (supplementary fig. S1B, Supplementary Material online).

In contrast, metazoan lamins possess a highly distinctive additional lamin tail domain (LTD) that bears an immunoglobulin-like fold (Dechat et al. 2008). Our expanded iterative homology searches for lamins identified significant hits in a much broader array of protists than previously. Altogether, we found lamin homologs in 12 distinct eukaryotic lineages: Metazoa, Choanoflagellates, Filasterea, Ichthyosporea, Dictyostelids, Rhizaria, Haptophytes, Dinoflagellates, Bicosoecida, Hyphochytridiomycetes, Oomycetes, and Ochrophytes (fig. 1 and supplementary table S1, Supplementary Material online). All candidates were verified by reverse BLAST against metazoa and returned lamins as top hits. By using either the whole lamin protein sequences or specific domains as queries, we found that all homologs could be identified using the LTD domain alone, while some homologs failed to be found when queried with just the rod domain and other coiled-coil proteins were frequently identified as unspecific hits. This suggests that putative lamin homologs with divergent or no LTD may be challenging to identify.

A domain recognized as LTD by NCBI CD-search also occurs in a variety of bacterial proteins, including enzymes that are clearly not lamins, but were retrieved by searches using the LTD as query. Proteins with such an LTD-like domain are present also in several eukaryotes (supplementary table S1, Supplementary Material online) but none contain coiled-coil regions or other lamin-like features and hence can be excluded as direct lamin relatives. Nevertheless, this raises the possibility that the LTD domain descended from prokaryotes and fused with an ancestral coiled-coil protein in the course of early eukaryotic evolution to produce the eukaryotic lamin.

## Evolution of Lamins

A phylogenetic tree of identified lamin homologs mirrors the current view of eukaryotic evolution, itself derived from phylogenetic reconstructions using concatenated data from hundreds of proteins (Burki 2014) (fig. 1). Such a coincident topology between species and gene trees is strong evidence for vertical evolution, suggesting that HGT events are unlikely. The most parsimonious interpretation is direct descent from an ancient eukaryotic lamin. Although the root position within the eukaryotic phylogeny is uncertain (Burki 2014) the most favored ‘unikont–bikont’ rooting, supported by a recent study (Derelle et al. 2015) suggests a pre-last eukaryotic common ancestor (LECA) lamin origin as homologs are present in both subgroups.

**Fig. 2.—:**
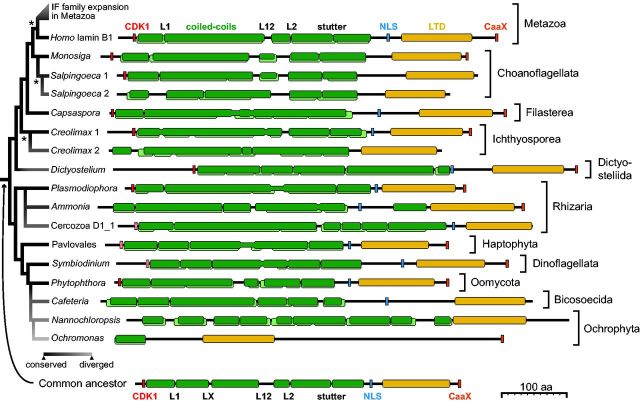
Comparison of lamin architecture across eukaryotes. Sequence motifs and structural elements are highlighted: CDK1 phosphorylation consensus sequence in red, classical NLS in blue, CaaX motif in orange, and Ig-like LTD domain highlighted in yellow. Coiled-coil regions and the interruptions in the heptad repeats were predicted in Marcoil (dark green) and Pcoils (light green). The heptad-repeat interruptions are named according to animal IF proteins. An additional interruption between L1 and L12 that was predicted in many of the lamins and was presumably present in LECA is marked as LX. See supplementary figure S3, Supplementary Material online, for sequence alignment and predicted pattern of heptad repeats in the filament domain and secondary structure elements in the LTD domain. The phylogenetic relationships between lamins are displayed on left, together with the level of divergence in respect to sequence, domain architecture and a presence of sequence motifs (grey scale). The asterisks point to the lineage-specific duplications of lamin gene.

By comparing homologous lamin sequences, we attempted to reconstruct the ancestral lamin (fig. 2 and supplementary fig. S2, Supplementary Material online). All major eukaryotic lineages possessing identifiable lamin homologs also possess representatives with well-conserved domain architecture, retaining all sequence motifs typical for metazoan lamins: a CDK1 phosphorylation site, monopartite nuclear localization signal (NLS), and a CaaX prenylation motif (Dechat et al. 2008). The level of conservation between these well-conserved lamins is further underlined by conserved interruptions in the predicted coiled-coil regions (supplementary fig. S2, Supplementary Material online). The ancestral eukaryotic lamin is therefore predicted as very similar to the B-type lamins of metazoa but likely possessed an additional heptad-repeat interruption (LX) (fig. 2 and supplementary fig. S2, Supplementary Material online). The lamins of Oomycetes, Dinoflagellates, and Haptophytes are most similar to this ancestral architecture.

**Fig. 3.—:**
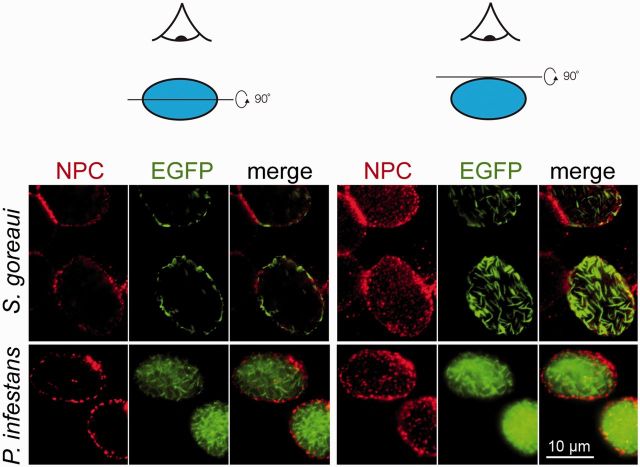
Localization of eGFP-Lamin fusion proteins expressed in HEK293T mammalian cells. The green signal is the fluorescence of the eGFP fused to the lamin of *S. goreaui* or *P. infestans.* Two sets of images of two different optical sections were taken to better evaluate the intra-nuclear localization. The instrument was focused on the midsection of the nucleus for the images in the left column and on the top of the NE for the images in the right column. The mAb414 antibody that stains the nuclear pore complex (in red), visible as a ring on a midsection or as dots at the top of the NE, was used as a reference. The scale bar on the right bottom of the figure is common to all images.

The conservation of predicted secondary structure, even in distant lamin homologs, implies conserved function. Genetic tools are limited or absent from the protists where novel lamins were identified, so we turned to a mammalian heterologous system. We selected two candidate lamins from the taxa least related to animals for which an entire mRNA coding sequence was available and expressed them as N-terminal fusions with eGFP in HEK293T cells. One homolog selected was from *Symbiodinium goreaui*, a Dinoflagellate, and the second from *Phytophthora infestans*, an Oomycete. Both proteins were detected as filamentous structures within the nucleus (fig. 3). The lamin of *S. goreaui* was present as long filaments (≤5 μm) concentrating at the nuclear periphery. The filaments of the *P. infestans* lamin were shorter and spread throughout the nucleus with no clear perinuclear enrichment, suggesting little or no anchoring to the NE (fig. 3), despite the presence of a likely functional prenylation signal (see supplementary text and supplementary fig. S3, Supplementary Material online, for details). Therefore, additional sequence features are likely required for NE association and the *P. infestans* lamin is not fully compatible with the mammalian system. Nonetheless, heterologous expression demonstrated that *S. goreaui* and *P. infestans* lamins are nuclear targeted and assemble into filaments. Taken together with conservation of predicted secondary structure, these data are strong evidence for conservation of function and assignment as *bona fide* lamins.

Although broad, the phylogenetic distribution of lamin homologs in eukaryotes is patchy, with multiple examples of clear homologs identified in one taxon but not in a sister lineage (fig. 1). Most homologs are relatively well conserved and easily identified in searches, even between distant eukaryotic lineages and with a dramatic *e*-value discrimination by significance between lamin homologs and the next hit. In taxa where lamin homologs were not identified, top hits were clearly nonspecific, making it unlikely that canonical lamins were overlooked (supplementary fig. S4, Supplementary Material online).

The lamin distribution suggests a large number of independent losses and is difficult to explain given the biological importance of the nuclear lamina. A possibility is that lamin sequences diverged dramatically in those eukaryotes that appear to be lamin-free. Despite the presence of well-conserved lamins across eukaryotic lineages, we also identified several poorly conserved homologs that lack one or more of the canonical lamin features (fig. 2). This is well documented in animals, where lamins expanded into a variety of IF proteins that acquired novel functions outside nucleus (Weber et al. 1989; Dodemont et al. 1990; Döring and Stick 1990; Kollmar 2015). However, we found that duplications of lamin genes had occurred also in ichthyosporeans, three rhizarian species, and a choanoflagellate *Salpingoeca* (fig. 1). Each of the ichthyosporean species sampled has two lamin homologs. One is a well-conserved lamin, while the second lacks the CDK1 site, NLS, and CaaX (fig. 2, represented by *Creolimax*). This suggests a functional divergence, with one of the duplicates potentially acquiring novel roles outside the nucleus, echoing the IF family expansion of Metazoa. In contrast, the multiple copies of lamin genes in *Salpingoeca* and the three rhizarian species have remained fairly similar.

All remaining lamin-containing taxa possess a single identifiable lamin gene per genome, usually highly conserved, but in several species even this single copy gene is diverged significantly. The most divergent and atypical lamin examples are the two homologs from Ochrophytes (*Nannochloropsis* and *Ochromonas*), in contrast to the canonical and well-conserved lamins of the closely related Oomycetes (fig. 2, represented by *Phytophthora*). This demonstrates great evolvability of lamin homologs in a narrow taxonomic context and also suggests that the inability to identify lamins in some lineages may reflect high divergence rather than complete loss. This also raises the possibility that the NMCP proteins of plants and NUP-1 of trypanosomes represent extremely diverged homologs of lamins. There is a high variability in NMCP and NUP-1 sequences even among closely related taxa, which is also apparent from comparisons of domain architecture and heptad-repeat patterns in the coiled-coil regions (supplementary fig. S1B, Supplementary Material online), and suggests that NUP-1 and NMCPs have undergone fast evolution. However, it is not currently possible to discriminate between the possibilities of NMCPs and NUP-1 being diverged lamins or products of convergence.

It is likely that novel lamina configurations are present in taxa lacking homologs of lamins, NUP-1, or NMCPs (fig. 1). Indeed, lamina-like structures were revealed by ultrastructural analyses in some of these lineages (Pappas 1956; Beams et al. 1957; Raikov 1982; Wen 2000) but biochemical fractionation and proteomics studies are needed to identify the molecular identity of the proteins involved. However, the conclusion that some eukaryotes lack the lamina-like meshwork of filamentous proteins is inescapable. Fungi provide a robust example of a lineage where such a structure is apparently missing (Cardenas et al. 1990).

## Lamina-Associated Proteins and Functional Redundancy at the NE

Several integral membrane proteins of the INM, that usually bind to the nuclear lamina, possess functions ascribed to lamins in lamin-deficient organisms and may potentially compensate for their absence (Taddei et al. 2004; Bupp et al. 2007; Hattier et al. 2007; Gonzalez et al. 2012). Previous reports have suggested a constrained distribution of these INM proteins, but were based on limited sampling (Brachner and Foisner 2011; Wilson and Dawson 2011). We re-examined the evolutionary representation of these lamin-associated INM proteins to evaluate possible co-evolution within the lamin system, with improved taxon sampling and more sensitive search algorithms. We were unable to conduct a similar investigation for NUP-1 or the NMCPs as characterization of these systems in terms of interacting proteins is rudimentary.

**Fig. 4.—:**
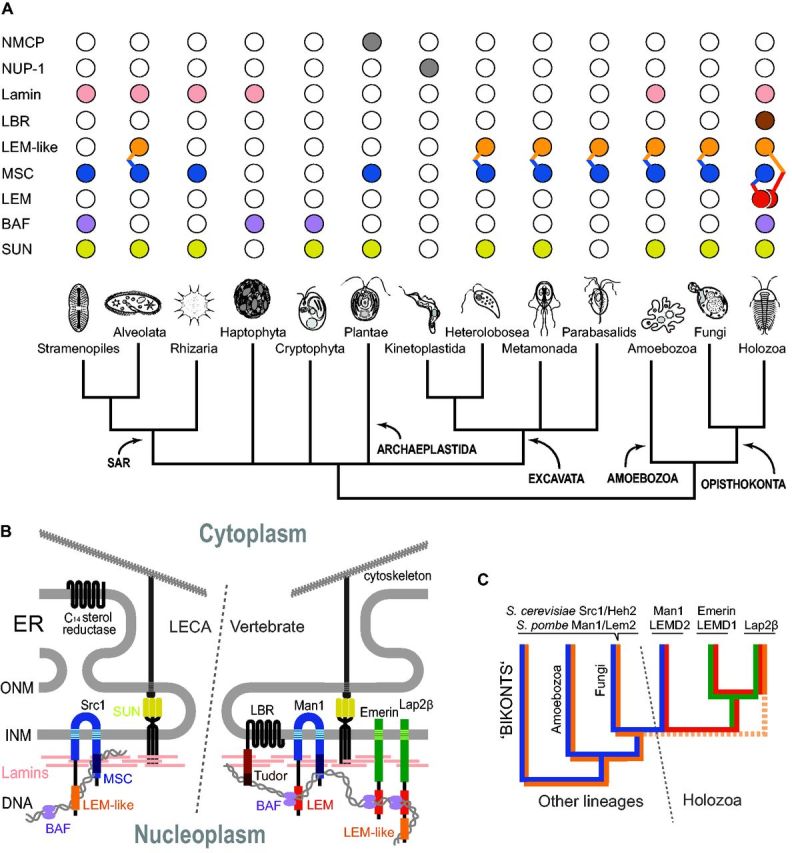
Phylogenetic distribution and evolution of NE proteins in eukaryotes. (*A*) Distribution of proteins with lamin-like functions and lamina-associated proteins among major eukaryotic lineages. Colored circles correspond to colors of proteins and protein domains in (*B*, *C*), while proteins represented by grey circles are omitted in (*B*). For the LEM-domain proteins, the distribution is shown separately for each conserved domain (LEM, LEM-like, and MSC domain). The circles are connected in taxa where two different domains exist as parts of the same polypeptide. Names of eukaryotic supergroups are capitalized. Haptophytes and Cryptophytes currently cannot be assigned to any recognizable supergroup. (*B*) Chromatin-binding NE proteins likely present in LECA compared with NE of vertebrates. Although the C-terminal part of LBR, which has C_14_ sterol reductase activity, is broadly distributed among eukaryotes and presumably present in the ER of LECA, the N-terminal fusion with the lamin- and chromatin-binding tudor domain is restricted to Metazoa. The role of the chromatin-binding protein BAF in tethering of chromatin to the NE *via* interactions with the LEM domain of various proteins is also likely Metazoa restricted. (*C*) Co-evolution and origin of individual domains of known vertebrate LEM domain-containing proteins. While the origin of MSC (blue) and LEM-like (orange) domains can be tracked back to LECA, the LEM-domain (red), as well as the C-terminal region of emerin and Lap2β (green), is restricted to Holozoa.

The LINC complex bridges both nuclear membranes and connects the lamina with the cytoskeleton and contains SUN and KASH domain proteins (Tzur et al. 2006). SUN domain proteins are widely distributed across eukaryotes (fig. 4A) and have conserved secondary structures, despite great size variation (Field et al. 2012). Their binding partners, the KASH domain proteins, in contrast display high-sequence variability, precluding confident identification outside metazoa. However, functionally analogous proteins with similar properties and domain architecture have been described in fungi and plants (Rothballer and Kutay 2013; Graumann et al. 2014), suggesting that both protein components of the LINC complex were present in LECA (fig. 4B).

LEM domain proteins connect chromatin to the NE (Wilson and Foisner 2010); their phylogenetic distribution is complex, as individual domains present in these proteins appear to have been shuffled and have distinct phylogenetic patterns (fig. 4). Using the emerin N-terminal LEM domain as query for PSI-BLAST and iterative HMMER searches, we identified all known mammalian LEM domain proteins (Man1, Lap2, LEMD1 plus Ankle1, and 2) but only a single nonanimal homolog in *Capsaspora*, a protist closely related to animals. This suggests that current animal LEM domain protein diversity was created by metazoan gene duplications and that the LEM domain is indeed Holozoa-specific. However, the MSC (Man1-Src1p C-terminal) domain of Man1 and LEMD2, shared also by the Scr1/Heh2/Man1/Lem2 proteins of yeasts, is broadly distributed (fig. 4A). Significantly, the MSC proteins of fungi and many protists possess a distinct N-terminal LEM-like domain, also present in the vertebrate Lap2 proteins where it is positioned at the very N-terminus and upstream to the canonical LEM domain (fig. 4B). In summary, among the known vertebrate LEM-domain proteins, only those containing the C-terminal MSC domain are widely distributed, but possess distinct N-terminal domains or none. The MSC and LEM-like domains bind directly to DNA and are evolutionarily older, while the LEM domain binds to chromatin *via* BAF and evolved within the animal lineage (Brachner and Foisner 2011) (fig. 4B and *C*).

The lamin B receptor (LBR), which also facilitates connections between NE and chromatin in mammalian cells, is similar to certain LEM domain proteins restricted to animals (deuterostomes in case of LBR). It is possible that some animal-specific chromatin-binding NE proteins such as emerin, Lap2, and LBR evolved as an adaptation to open mitosis, since they reconstitute around chromosomes early in telophase and play a pivotal role in the NE reformation process (Foisner 2003).

Overall, the phylogenetic distribution of lamin-binding proteins suggests that NE components facilitating connections between the lamina, the cytoskeleton, and the chromatin were likely also included in the LECA NE (fig. 4). Significantly, none of this cohort of lamina-associated proteins is detected in trypanosomatids.

Growing evidence suggests that lamin-binding integral NE proteins with adhesive domains are sufficient for tethering chromatin to the nuclear periphery and regulation of position-mediated gene expression (Brachner and Foisner 2011). The MSC/LEM-like domain containing proteins anchor telomeres to the NE in fission yeast (Gonzalez et al. 2012) while the SUN-domain containing protein Mps3 and perinuclear protein Esc1 facilitate the attachment to chromatin via interaction with the Sir4 pathway of chromatin tethering and silencing in the budding yeast (Taddei et al. 2004; Bupp et al. 2007). Such interactions between chromatin and INM proteins likely also impart stiffness to yeast nuclei (Schreiner et al. 2015), demonstrating that, as for chromatin tethering, the role of lamins as scaffolds supporting nuclear shape may be dispensable and supported by other proteins. In mammals, lamins are required for correct organ development but, surprisingly, are nonessential for NE structure and proliferation in embryonic stem cells (Fong et al. 2006; Coffinier et al. 2011). The yeast nuclear basket coiled-coil protein Mlp2 forms filaments and a network interconnecting individual NPCs potentially providing further structural support (Kosova et al. 2000) and the human Mlp homolog Tpr also can polymerize into long filamentous structures. Mlp/Tpr homologs are widely distributed among eukaryotes and likely present in LECA (Field et al. 2014). It is possible that lamins emerged as the dominant scaffold proteins supporting the NE only in some lineages, whereas in others this function was assumed by phylogenetically unrelated proteins.

## Conclusion

Lamins are broadly distributed across eukaryotes, but with examples of within-lineage divergence and many losses. Such distribution supports the view of lamins as an ancient nuclear feature and suggests, in line with previous studies, that cytoplasmic IFs (e.g., keratin, vimentin, or desmin) arose much later in the evolution by duplications of the ancestral lamin gene in the metazoan lineage. However, the analyses presented here also suggest that a similar functional expansion may have occurred independently in other eukaryotic group(s). While several alternative solutions to building a lamina are now known, a coiled-coil architecture is common. As LECA possessed lamins this suggests the ability to regulate genes by inactivating chromosomal regions *via* heterochromatization, which itself implies a complex life cycle with developmentally regulated gene expression, and which is likely consistent with the great complexity of the LECA as revealed by many reconstructions. Determining those proteins comprising the nuclear lamina in other eukaryotic lineages, resolving how lamins diverged or came to be replaced and what effect this has on gene expression and other functions is of significant importance to understanding eukaryotic origins and diversity.

## Materials and Methods

### Comparative Genomics

Metazoan lamin protein sequences, both full-length or the C-terminal tail domain, were used as queries for iterative BLAST (PSI-BLAST) (Altschul et al. 1997) and HMMER (jackhmmer) (Finn et al. 2011) searches against the NCBI-predicted protein database. Both BLAST and HMMER identified lamin-like proteins in Oomycetes, Choanoflagellates, and *Capsaspora* and the NE81 proteins of Dictyostelids as significant hits. This enlarged lamin dataset was subsequently used to screen additional genomic resources by BLAST, including NCBI’s ESTs and whole genome shotgun contigs, Joint Genome Institute’s genomes, MMETSP, and the Origins of Multicellularity Database at Broad Institute. The same strategy was applied to screen for homologs of lamin-binding proteins. All proteins used in this study are listed in supplementary table S1, Supplementary Material online.

### Phylogenetic Analyses

Lamin sequences of the representative metazoan taxa and all the available lamin-like proteins of protists were aligned in Mafft (Katoh and Standley 2013) and the alignment was edited in BioEdit (Hall 1999). Edited alignments are provided below as supplementary information online. Maximum likelihood and Bayesian phylogenetic trees were constructed in PhyML 3.1 (Guindon et al. 2010) and Mr. Bayes (Ronquist and Huelsenbeck 2003), respectively. The branch supports were evaluated by bootstrap (1,000 iterations) and Bayesian posterior probabilities (10,000,000 generations).

### Identification of Conserved Domains and Secondary Structure Predictions

The NCBI CD-search was used to identify conserved domains. Secondary structure elements and folds were predicted in Phyre2 (Kelley and Sternberg 2009). PCOILS and MARCOIL (http://toolkit.tuebingen.mpg.de) were used for prediction of coiled-coil regions and the presence of heptad repeats.

### Heterologous Expression and Localization

*Phytophthora P. infestans* RNA was kindly provided by Sebastian Schornack (SLCU, Cambridge, UK), reverse-transcribed to cDNA and used for PCR amplification of the lamin coding sequence. The coding sequence of *S. goreaui* lamina was optimized for mammalian expression and custom-synthesized as GeneArt Strings DNA Fragment (Life Technologies Ltd, Paisley, UK). Both lamin coding sequences were cloned into pEGFP-C3 and sequenced. pEGFP-C3::lamin constructs were transfected into HEK293T cells using FuGENE^®^6 (Promega, Southampton, UK) and the expression of the full-length constructs was confirmed by Western blot (supplementary fig. S3, Supplementary Material online). eGFP::lamin fusion proteins were visualized using a ZEISS Axiovert 200M fluorescence microscope and the images captured with a ZEISS AxioCam. Nuclear pore complexes were costained using MAb414 monoclonal antibody (BioLegend UK Ltd, London, UK, 1:3,000) and Alexa Fluor 568 goat anti-mouse secondary antibody (Life Technologies Ltd, Paisley, UK, 1:1,000).

## Supplementary Material

Supplementary Figures and Tables
