# Supplementary material for: Ancient Eukaryotic Origin and Evolutionary Plasticity of Nuclear Lamina
Source: Genome Biol Evol. 2016 Apr 28;8(9):2663–71. doi: 10.1093/gbe/evw087 (PMC5630835; doi:10.1093/gbe/evw087)
Supplement: Supplementary Figures and Tables [file Supplementary_material_evw087.pdf]

**Supplementary material for:**

# Ancient eukaryotic origin and evolutionary plasticity of nuclear lamina

Ludek Koreny and Mark C. Field

School of Life Sciences, University of Dundee, Dundee, DD1 5EH, UK.

## Supplementary text

**Mutagenesis experiments on *P. infestans* lamin:** While the lamin of *S. goreaui* localized preferentially to the nuclear periphery in the human HEK293T cells, the filaments of the *P. infestans* lamin were spread throughout the nucleus with no clear perinuclear enrichment, suggesting little or no anchoring to the NE (Fig. 3). Therefore, additional sequence features are likely required for NE association and the P. infestans lamin is not fully compatible with the mammalian system. One possible explanation is that the prenylation signal (CaaX motif) of *P. infestans* lamin is not recognized by human farnesyltransferase and the posttranslational modification for increasing hydrophobicity of the C-terminus and attachment to the INM is consequently not taking place. The CaaX of Phytophthora possesses methionine at a2, which is uncommon (Supplementary Table S1, Supplementary Material online). However, mutation of the CaaX sequence to the more conventional CAIM of human lamin B1 had no impact on the localization. We also considered the possibility that *P. infestans* lamin could be processed by Zmpste24 protease since, within its sequence, we identified a hypothetical cleavage site with properties similar to that contained in pre-laminA, a natural target of Zmpste24 (Barrowman et al. 2012. PLoS One 7:e32120) (Supplementary Fig. S3, Supplementary Material online). We expressed *P. infestans* lamin with a non-cleavable mutation at this site as well as a truncated version with a premature stop codon immediately downstream of the hypothetical Zmpste24 site. None of these mutations significantly altered the localisation of the lamin fusion proteins. SDS-PAGE and western blot of the isolated wild type fusion protein failed to reveal the predicted processed form (Supplementary Fig. S3, Supplementary Material online), suggesting that, despite the presence of a likely functional prenylation signal in the *P. infestans* lamin, additional sequence features are required for NE association in the mammalian nucleus.

## Supplementary figure legends

**Fig. S1. Distribution of NUP-1 and NMCP proteins.** (*A*) Phylogenetic trees of NUP-1 and NMCP proteins. Searches for NUP-1 and NMCP identified orthologs restricted to Trypanosomatida and Streptophyta, respectively. Phylogenetic relationships are based on analyses of protein sequences (Supplementary Table S1, Supplementary Material online). The edited alignments used for the phylogenetic analyses are below in the Supplementary

Material online. The numbers above branches are PhyML bootstrap/Bayesian posterior probability values. (*B*) Secondary structure of NUP-1 and NMCP proteins. Predicted nuclear localization signals (NLS) are marked by blue rectangles. The central portion of NUP-1 orthologs consists of repeats of variable size and number. The presence of direct repeats precludes unambiguous assembly of sequence data for many orthologs, and repeat numbers for *P. serpens* and *S. culicis* were estimated based on repeat regions assembled into several contigs, while repeat regions are absent for NUP-1 of *P. confusum* and *H. muscarum*. The four NMCP paralogs of *A. thaliana* are the result of three duplications in the plant lineage (red dots) in the ancestors of Spermatophyta (seeded plants), Eudicots and Brassicales.

**Fig. S2. Sequence alignment of lamin protein sequences from representatives of major eukaryotic groups.** Coiled-coil regions were predicted by Marcoil and Pcoils tools and are highlighted in green; the parts recognized as filament domains by NCBI CD-search are highlighted in darker shade of green and LTD domains in dark yellow. The light yellow delimits the regions that match the Ig-like LTD fold predicted by Phyre2. The red box marks the CDK1 phosphorylation site consensus sequence, the blue box marks the classical monopartite NLS, and the CaaX motif is in the orange box. The first and fourth positions of the predicted heptad pattern in the filament domain are marked by letters a and d above the sequence. Where they match hydrophobic and uncharged amino acids favourable for the coiled-coil formation, they are in a black box, while a letter in a white box marks the less favourable amino acid. The haptad-repeat interruptions and gaps within the coiled-coil region are marked by red dots and their hypothetical positions were estimated based on both Marcoil and Pcoils predictions and the level of conservation of the respective regions in the alignment. The secondary structural elements in the LTD domain were predicted by Phyre2 and are in grey. The arrows indicate beta sheets and spirals alpha helices. The known secondary structure of the human lamin B1 is shown using red symbols for comparison.

**Fig. S3. Comparison of C-terminal regions of human lamins with lamin homologs of Oomycetes.** (*A*) Amino acids with similar properties surrounding the cleavage site of human prelamin A and hypothetical cleavage site of *P. infestans* lamin (arrow heads) are highlighted in red. The cleavage of prelamin A by Zmpste24 protease results in loss of the hydrophobic C-terminus and mature lamin A therefore localizes to the nuclear interior. A-type lamins are unique to vertebrates, suggesting that there would be no selection pressure for other eukaryotes to avoid a Zmpste24 cleavage site (Barrowman et al. 2012. PLoS One 7:e32120). Additional to several amino-acid groups surrounding the cleavage site, the spacing from the farnesyl-cysteine to the cleavage site is important for optimal cleavage (Barrowman et al. 2012. PLoS One 7:e32120). We noticed that the C-terminal region of *P. infestans* lamin contains F-L (500-501) sequence 10 amino-acids upstream of the farnesyl-cysteine, similar to the Y-L (646-647) cleavage site of human prelamin A. Furthermore, arginine is present in the -4 position of the putative F-L cleavage site of *P. infestans* lamin, while the presence of arginine in the -3 position of the cleavage site in human

prelamin A is essential for cleavage. The L647R mutation in human prelamin A effectively abolishes cleavage by Zmpste24 (Barrowman et al. 2012. PLoS One 7:e32120). We therefore created similar mutations in the *P. infestans* lamin, i.e. L501R and L501M (methionine is the most common amino-acid in that position in other *Phytophthora* species), and a version containing a STOP codon after F500. The intranuclear localization of all mutated lamins was indistinguishable to the wild-type lamin. (*B*) Western blot detection of EGFP-Lamin constructs in lysates of HEK293T cells. The wild-type (WT) lamin of *Phytophthora infestans* is compared with mutagenised lamins. There is an apparent shift in size of the lamin L501Stop compared to the wild-type lamin and the L501R and L501M mutations that were all detected as proteins of the same size by a western blot, which clearly demonstrates that the lamin of *P. infestans* is not cleaved by the human Zmpste24. The constructs were detected using Roche mouse monoclonal anti-GFP antibody, version 06 (1:1000) and secondary goat anti-mouse HRP conjugated antibody (1:10000). Samples were separated alongside the Prestained Protein Marker, Broad Range (New England BioLabs; 7-175 kDa). The calculated theoretical molecular weights (kDa) of the eGFP-lamin constructs for lamins of *Symbiodinium goreaui* (S) and *P. infestans* (P) are shown next to the protein bands.

**Fig. S4. Specificity in searches for lamins.** E-value distribution of the top blastp hits in representative genomes/transcriptomes of eukaryotic taxa that possess lamin homologs identified in this study (*A*) and in representative genomes of related taxa, where no lamin homologs were found (*B*). The lamin protein sequences of metazoa, namely of *Homo sapiens*, *Branchiostoma floridae*, *Priapulus caudatus*, *Hymenolepis microstoma*, *Aplysia californica*, *Drosophila melanogaster*, *Daphnia pulex*, *Nematostella vectensis* and *Trichoplax adhaerens* were used as queries to search in protein or predicted peptide databases of selected species (supplementary table S1, Supplementary Material online). The accession numbers and annotations of the hits are listed in the tables beside the e-value distribution charts. There is a substantial difference between the e-values of the 1st hit for each taxon in A - the actual lamin orthologs (surrounded by a dashed line) and all the other hits that mostly represent various coiled-coil proteins that are clearly distinct from lamins.

Fig. S1

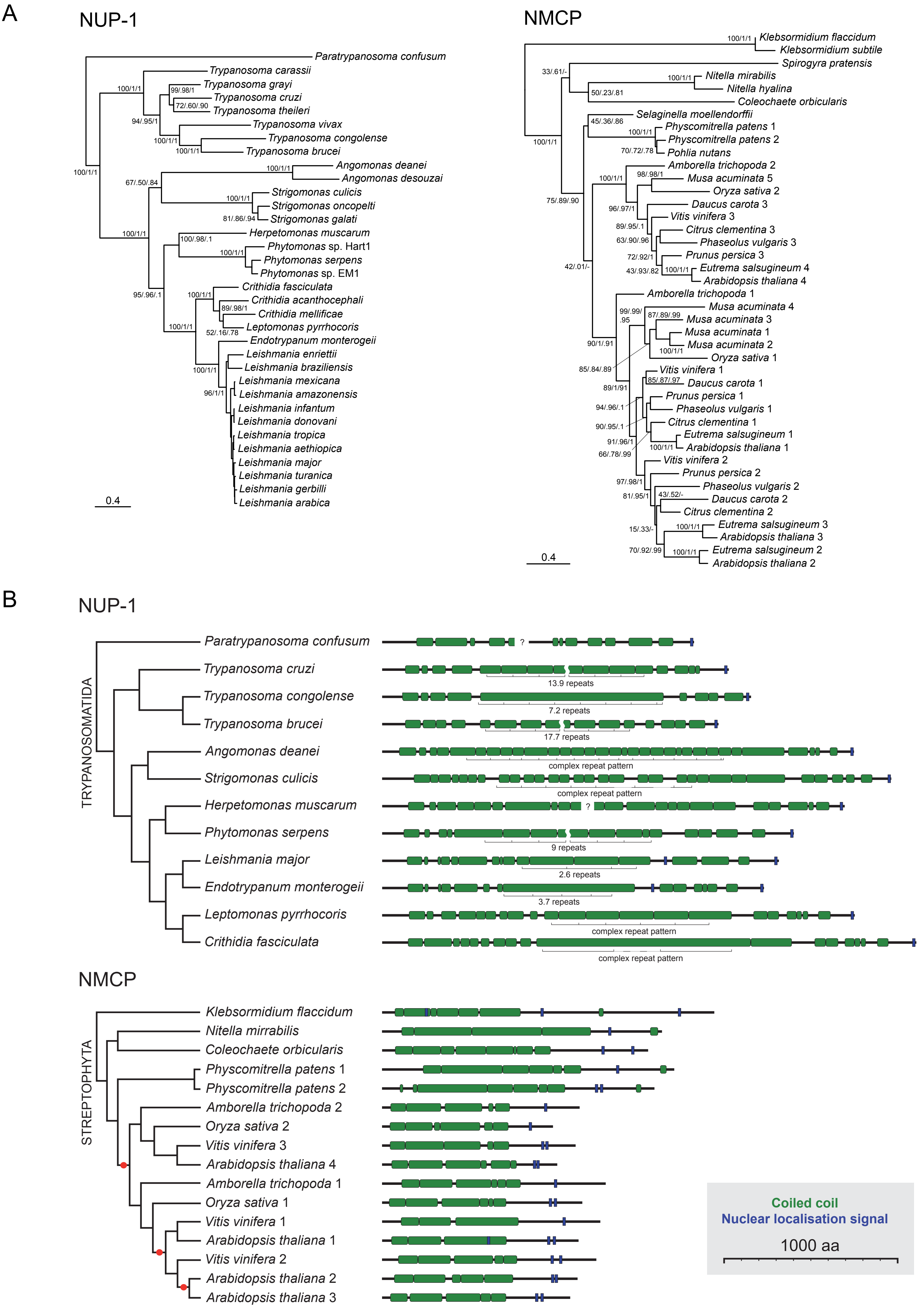
A
NUP-1
NMCP
0.4
0.4
B
NUP-1
TRYPANOSOMATIDA
Paratrypanosoma confusum
Trypanosoma cruzi
13.9 repeats
Trypanosoma congolense
7.2 repeats
Trypanosoma brucei
17.7 repeats
Angomonas deanei
complex repeat pattern
Strigomonas culicis
complex repeat pattern
Herpetomonas muscarum
Phytomonas serpens
9 repeats
Leishmania major
2.6 repeats
Endotrypanum monterogeii
3.7 repeats
Leptomonas pyrrhocoris
complex repeat pattern
Crithidia fasciculata
complex repeat pattern
NMCP
STREPTOPHYTA
Klebsormidium flaccidum
Nitella mirrabilis
Coleochaete orbicularis
Physcomitrella patens 1
Physcomitrella patens 2
Amborella trichopoda 2
Oryza sativa 2
Vitis vinifera 3
Arabidopsis thaliana 4
Amborella trichopoda 1
Oryza sativa 1
Vitis vinifera 1
Arabidopsis thaliana 1
Vitis vinifera 2
Arabidopsis thaliana 2
Arabidopsis thaliana 3
Coiled coil
Nuclear localisation signal
1000 aa

Fig. S2

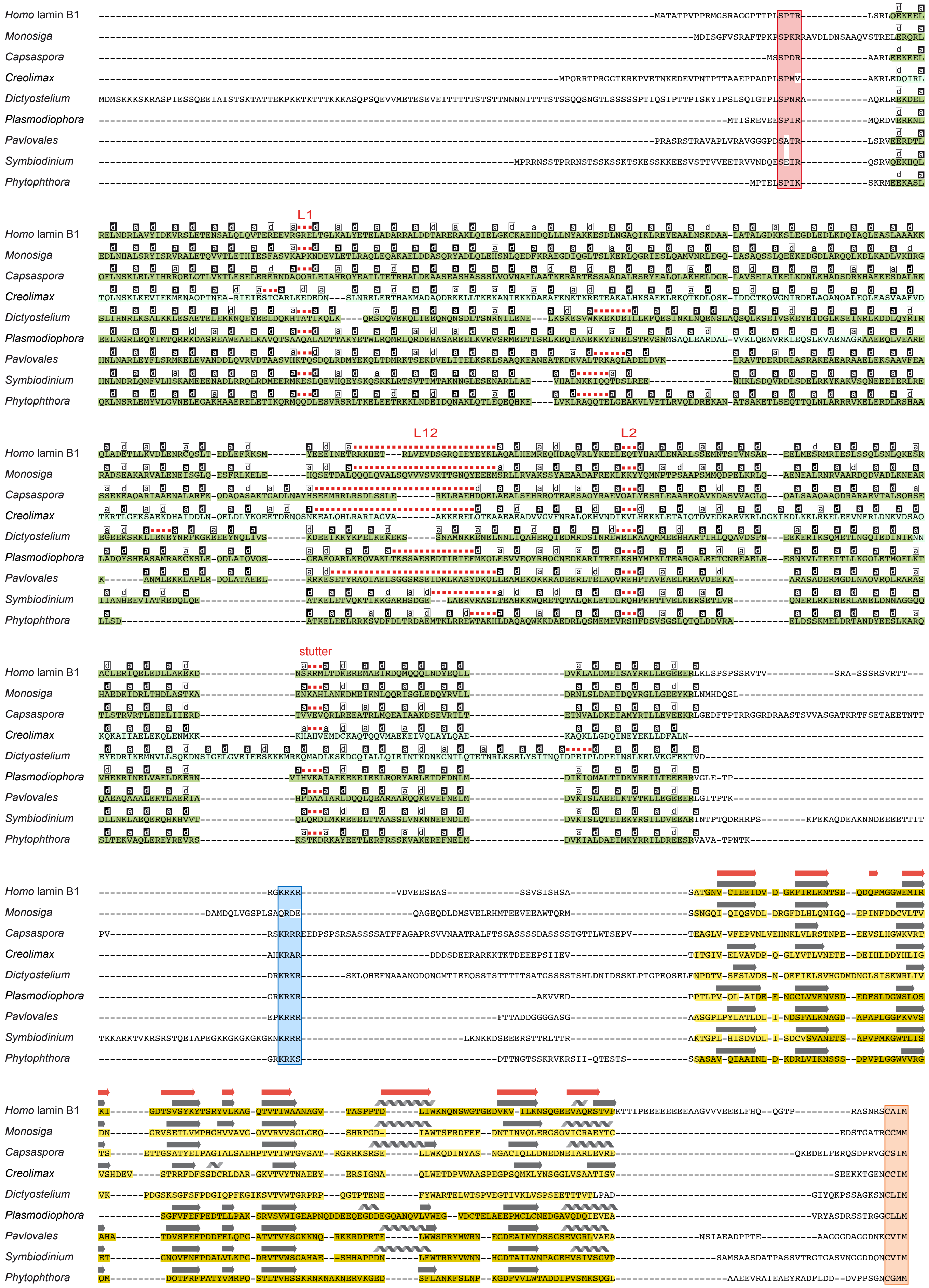
Homo lamin B1
Monosiga
Capsaspora
Creolimax
Dictyostelium
Plasmodiophora
Pavlovales
Symbiodinium
Phytophthora
L1
L12
L2
stutter

# Fig. S3

A

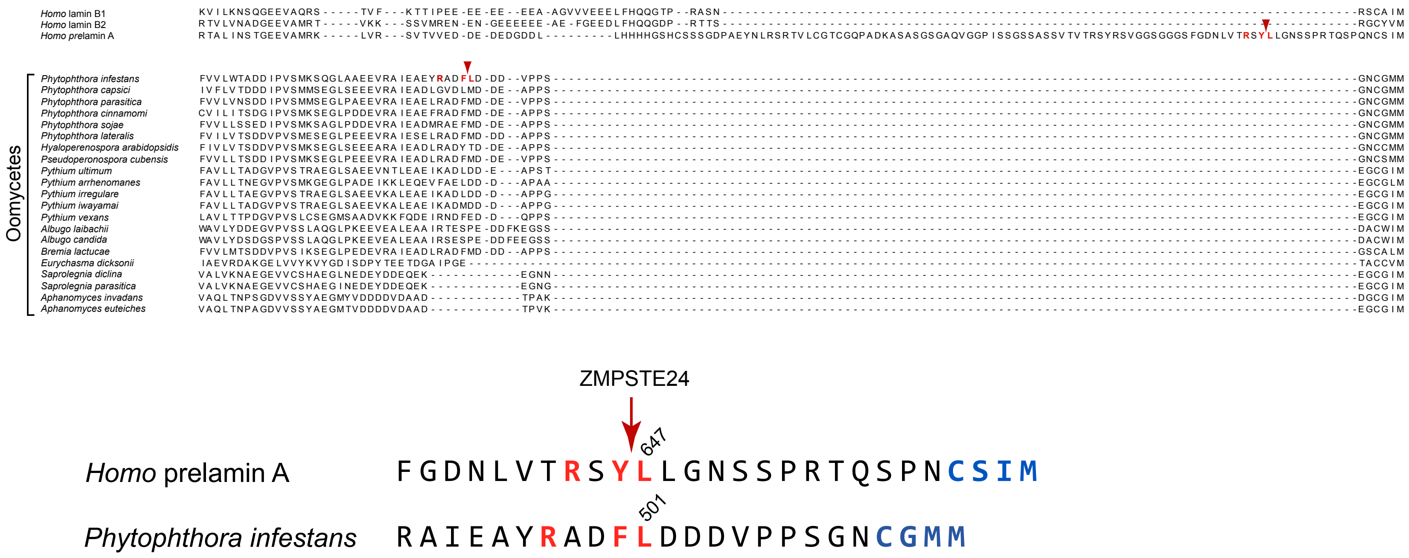
Homo lamin B1
Homo lamin B2
Homo prelamin A
Oomycetes
Phytophthora infestans
Phytophthora capsici
Phytophthora parasitica
Phytophthora cinnamomi
Phytophthora sojae
Phytophthora lateralis
Hyaloperenospora arabidopsidis
Pseudoperonospora cubensis
Pythium ultimum
Pythium arrhenomanes
Pythium irregulare
Pythium iwayamai
Pythium vexans
Albugo laibachii
Albugo candida
Bremia lactucae
Eurychasma dicksonii
Saprolegnia diclina
Saprolegnia parasitica
Aphanomyces invadans
Aphanomyces euteiches
ZMPSTE24
647
Homo prelamin A
F G D N L V T R S Y L L G N S S P R T Q S P N C S I M
501
Phytophthora infestans
R A I E A Y R A D F L D D D V P P S G N C G M M

B

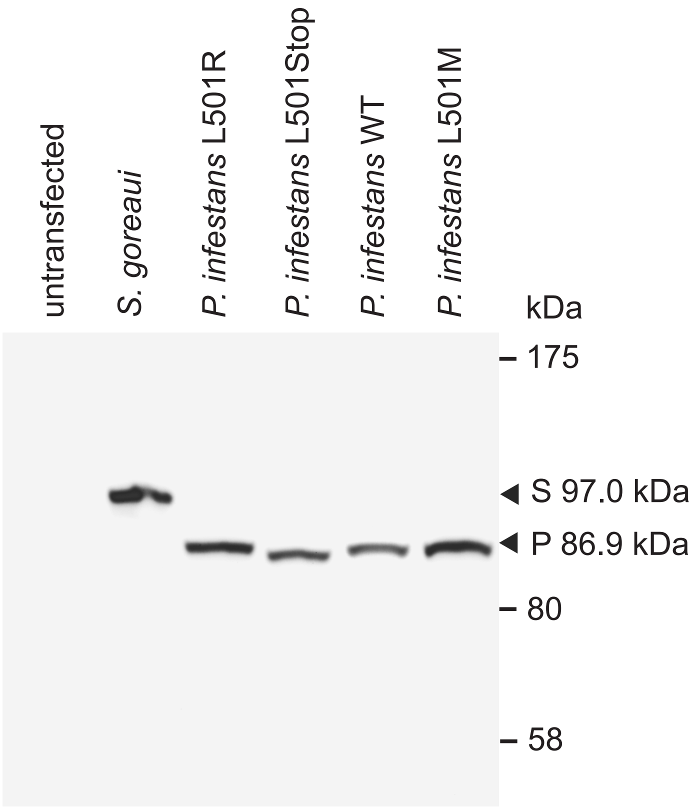
untransfected
S. goreaui
P. infestans L501R
P. infestans L501Stop
P. infestans WT
P. infestans L501M
kDa
175
S 97.0 kDa
P 86.9 kDa
80
58

# Fig. S4

A

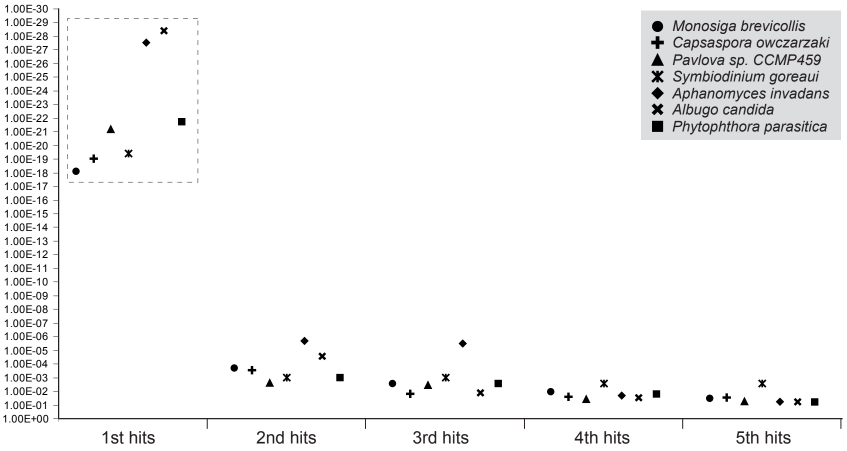


| Genome | e-value | Accession nb. | BLAST HIT Annotation |
|---|---|---|---|
| *Monosiga brevicollis* | 8E-19 | XP_001745977 | Lamin |
| | 2E-04 | XP_001750258 | SH3 domain-containing coiled-coil protein |
| | 0.003 | XP_001742372 | RNR_PFL domain-containing coiled-coil protein |
| | 0.011 | XP_001745137 | Calponin homology domain-containing coiled-coil protein |
| | 0.033 | XP_001746167 | hypothetical coiled-coil protein |
| *Capsaspora owczarzaki* | 1E-19 | XP_004365259 | Lamin |
| | 3E-04 | XP_004344350 | cenpe protein |
| | 0.017 | XP_004346859 | cut-like homeobox 1 (CASP) |
| | 0.028 | XP_004345230 | unconventional myosin-XVIIIa-like |
| | 0.031 | KJE95785 | pseudouridine synthase domain-containing coiled-coil protein |
| *Pavlova sp. CCMP459* | 8E-22 | 190508862 | Lamin |
| | 0.003 | 190510564 | Choline transporter domain-containing protein |
| | 0.004 | 190525724 | dynein heavy chain 7 |
| | 0.047 | 190503290 | outer dynein arm-docking complex protein-like |
| | 0.062 | 190525808 | LIM and Calponin homology domain-containing protein |
| *Symbiodini-um goreaui* | 4E-20 | 199596768 | Lamin |
| | 0.001 | 199636648 | DnaJ-domain-containing protein |
| | 0.001 | 199640602 | ubiquitin-like domain containing coiled-coil protein |
| | 0.003 | 199570084 | Leucine Rich Repeat family protein |
| | 0.003 | 199596170 | fas-binding factor 1-like |
| *Aphanomy-ces invadans* | 3E-28 | XP_008867452 | Lamin |
| | 2E-06 | XP_008870094 | TNFR domain-containing coiled coil protein |
| | 3E-06 | XP_008873497 | WWP domain-containing coiled-coil protein |
| | 0.031 | XP_008877304 | hypothetical coiled-coil protein |
| | 0.075 | XP_008869784 | hypothetical coiled-coil protein |
| *Albugo candida* | 4E-29 | CCI10441 | Lamin |
| | 3E-05 | CCI50727 | putative spindle assembly protein |
| | 0.013 | CCI48786 | hook protein-like domain cointaining coiled-coil protein |
| | 0.047 | CCI41228 | Leucine rich repeats-containing coiled-coil protein |
| | 0.072 | CCI41579 | TPR/Mlp nucleoporin |
| *Phytophthora parasitica* | 2E-22 | XP_008905021 | Lamin |
| | 0.001 | XP_008895485 | plectin-like protein |
| | 0.003 | XP_008902902 | hypothetical coiled-coil protein |
| | 0.019 | XP_008902191 | WD repeat-containing protein 65 |
| | 0.081 | XP_008905554 | coiled-coil domain-containing protein 57-like |

B

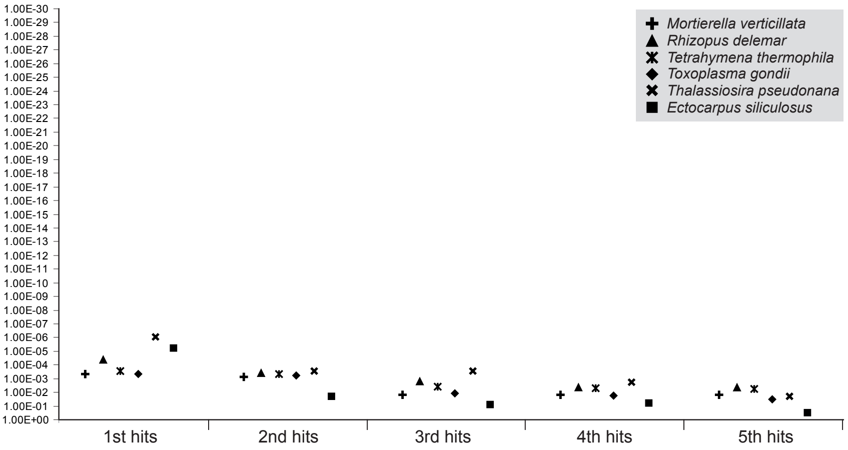


| | | | |
|---|---|---|---|
| *Mortierella verticillata* | 5E-04 | MVEG_05989 | myosin 9-like protein |
| | 8E-04 | MVEG_07393 | hypothetical coiled-coil protein |
| | 0.016 | MVEG_00433 | Zuo1/DnaJ subfamily C member 2 |
| | 0.017 | MVEG_00637 | tuberous sclerosis 1-like |
| | 0.017 | MVEG_04666 | glutaredoxin domain-containing coiled-coil protein |
| *Rhizopus delemar* | 5E-05 | EIE92276 | hypothetical coiled-coil protein |
| | 5E-04 | EIE76308 | PT repeat-containing coiled-coil protein |
| | 0.002 | EIE85364 | P-loop containing nucleoside triphosphate hydrolase protein |
| | 0.005 | EIE90623 | GRIP domain-containing coiled-coil protein |
| | 0.005 | EIE81526 | Putative Dynein heavy chain |
| *Tetrahymena thermophila* | 3E-04 | XP_001015907 | golgin-like coiled-coil protein |
| | 5E-04 | XP_001008452 | FYVE zinc finger-containing coiled-coil protein |
| | 0.004 | XP_001014796 | hypothetcal coiled-coil protein |
| | 0.005 | XP_001009123 | EEIG1/EHBP1 protein amine-terminal domain protein |
| | 0.006 | XP_001032362 | hypothetcal coiled-coil protein |
| *Toxoplasma gondii* | 5E-04 | EPT28173 | hypothetical coiled-coil protein |
| | 6E-04 | XP_002371471 | RCC1 domain-containing protein |
| | 0.013 | XP_002366795 | hypothetical coiled-coil protein |
| | 0.019 | EPT31185 | surface antigen repeat-containing protein |
| | 0.035 | EPT29827 | RCC1 domain-containing protein |
| *Thalassiosira pseudonana* | 1E-06 | XP_002294807 | TPR/Mlp nucleoporin |
| | 3E-04 | XP_002286197 | plectin-like protein |
| | 3E-04 | XP_002291803 | hypothetical coiled-coil protein |
| | 0.002 | XP_002287281 | hypothetical coiled-coil protein |
| | 0.022 | XP_002294067 | hypothetical coiled-coil protein |
| *Ectocarpus siliculosus* | 6E-06 | CBN77699 | TNFR domain-containing coiled coil protein |
| | 0.021 | CBJ48665 | hypothetical coiled-coil protein |
| | 0.081 | CBJ48925 | structural maintenance of chromosomes protein 6 |
| | 0.064 | CBJ25580 | WD repeat-containing protein 65 |
| | 0.31 | CBN74724 | kinetochor protein NUF2 |

**Table S1.** Protein sequences used in this study.

| Species | Group | Source | Sequence |
|---|---|---|---|
| LAMINS: | | | |
| Homo sapiens | Metazoa | NCBI\|<br>AAC37575 | MATATPVPPRMGSRAGGPTTPLSPTRLSRLQEKEELRELNDRLAVYID-<br>KVRSLETENSALQLQVTEREEVRGRELTGLKALYETELADARRALDDTARERAK-<br>LQIELGKCKAEHDQLLLNYAKKESDLNGAQIKLREYEAALNSKDAALATALGD-<br>KKSLEGDLEDLKDQIAQLEASLAAAKKQLADETLLKVDLENRCQSLT-<br>EDLEFRKSMYEEEINETRRKHETRLVEVDSGRQIEYEYKLAQALHE-<br>MREQHDAQVRLYKEELEQTYHAKLENARLSSEMNTSTVNSAREELM-<br>ESRMRIESLSSQLSNLQKESRACLERIQELEDLLAKEKDNSRRMLTD-<br>KEREMAEIRDQMQQQLNDYEQLLDVKLALDMEISAYRKLLEGEEERL-<br>KLSPSPSSRVTVSRASSSRSVRTTRGKRKRVDVEESEASSSVSISHS-<br>ASATGNVCIEEIDVDGKFIRLKNTSEQDQPMGGWEMIRKIGDTSVSY-<br>KYTSRYVLKAGQTVTIWAANAGVTASPPTDLIWKNQNSWGTGEDVK-<br>VILKNSQGEEVAQRSTVFKTTIPEEEEEEEEEAAGVVVEEELFHQQGTPRASNRSCA-<br>IM |
| Nematostella vectensis | Metazoa | NCBI\|<br>XP_001629288 | MATATKSPASSSSTPKTPVSSSRIMGSSPPGSAKFTRAQEKAELQHLNDR-<br>LATYIDRVKNLEQENSKLRSEVTVSRKTVEREVDSMKSLYETELADARRLLDE-<br>TAKEKAKQQIESSKNSNDAQEFKNKFDKEAAARKKAEKELNDVRKLLHDKENQL-<br>TRRNQEALNLESVLRELQGECEELKDALKAAKYALEQETLTRVDLENRCQSLQ-<br>EEQNFKKQMYDKELSDIRSQLKTVETKRVVVETDYKDKYEGLMAEKLQELRED-<br>YDSDARSFKEETELLYSSKFEELRIQRERDSEALAKLREENRNLSKSVDELSSQ-<br>VHQLEAKNNALVSRVSDLQGLRAQDKEKHDNEILLRENEIAELRTSIDDALRDY-<br>EDLMGVKVALDMEITAYRKLLESEETRLEITPPASPVLGGSSSVSQTRRGNKRA-<br>RTEETESTMTTTTTAEGAIQFTEADPDGKYIKIYNSGEKDEALGGWTIQRQVGT-<br>EDPSVYKFTPKYVLKSQSHVTVWSAQGGGTHKPPSDLVFKQLPSWGSGNEAR-<br>TALVNAGGEEMATLLEEKVFQHYSTDVIDSGRRGRGRRDGEVAKGCAVM |
| Trichoplax adhaerens | Metazoa | Krüger et al. 2012. Mol Biol Cell. 23:360-70. | MSPLKIKRVQEKEELKNLTDRLASYIEKSRFLESRNARLNEEIRSSRRSGDENIKSL-<br>KALYEQELQEARKTIDEMANEKSKITITVMKYTKQVEELTSDLEKVKASKSSLQ-<br>VALNNAEKRATELEGEVVNERKNVAELREVVIGHKEKIQRSSEELESANKLAE-<br>QQALRRVQIENEYQTLKESAEFSTEMHQQEIRSLKKNLKTVDDQR-<br>STLQDDFQSEYDTKMTEALQQLRRDNEENSKRLKQEVEELYRSQ-<br>VKELESQRERDTKIITELKNECRKFRDKSESISSEVHVLRAENSSLQT-<br>RKRELEESLIEERQKNTNKFEEYEMEMRALQLKLEEQSTEYKELLDV-<br>KIELDNEIAAFRKLLEGEETRLSSSPSSPFAQRKRRRIDEDSDYIVSS-<br>HATGDVQISDVDKHGLYVRLLNTSKTDCHIGNFVIKHQVDNKNEISY-<br>KFNTKAILKGNCVTTVMAADSGQSHKPPANYLWKQQNNFGLGEE-<br>MVTRLVNAGGEEIAIFNLTKAQEVEIKDELFQSYQVKDTGTNRCCIQ |
| Branchiostoma floridae | Metazoa | NCBI\|<br>XP_002586730 | MSRRSQKLTQKTVVTETTTSSVSTPKVTKQVQEAFVTQLSPTRLTR-<br>MQEKQELQWLNDRLAQYIDRVRYLEAENSRLMVQVTSSEEITQREVTNIKSMFE-<br>QELTDARKLLDDTAKEKARVQIEAGKYRAEADELRAKLAKSEGALATAEKKRHQAE-<br>SALNEKEGRLQNAIADKQRAEGELAALRLEFANLEKQLATARKQLEEETLLRVDLE-<br>NKVQTLREEVEFNKQVYEQELTESRTRKEVSITEVDAGEAAFESRLQEALREMRE-<br>QHELEARSMREELETMYTQKITSMRTDSERGSNALSVAREEMRESRARIDSLMSQ-<br>VSGLQGQNASLEARCKELEGMMARMSEESRSSAEQYEREIRQLREEISQMMVDY-<br>QELMDIKIALDLEISAYRTLLEGEEQRLKLTPTPSPTASNHSISHDCRGRTSKRKRV-<br>EMESEESGSSSSGAVAVGGVDLEGKFVKLQNTSAEKDMSMGGWLLKRTVGGGEE-<br>ISYKFPSRYVLKAGQSVTVWATEGGGTHSPPSDLLFRGQASWGSGDDTKTLLVND-<br>SGEEMATRSVTRVASSGFSSTSGGTFVEVGQEELFHQQGDPASPRQRCSIM |
| Priapulus caudatus | Metazoa | NCBI\|<br>NP_999665 | MASQTKSQRYEKRSTRTEFKTTSTPKASSSSQAKSLLSPAKISRHEEKEELIGLNDR-<br>LATYIDRVRHLELENGRLLVQITSFEETQTRDIEGIKVLYEKELADARKLLDE-<br>TAGEKAKLQIECGKYKTELDSLRPRVGKLEKELNAANKRVASLEAQVAEKD-<br>VRIRSLSNDKRSLEDELNELKKDLGNKEKQLKVAKKQVEEETLLRVDLENRLQSL-<br>KEELSFKEQLFKEELRETRTKRQVDMSTIEGQSIEELNNSLYESLQELREQTSEQT-<br>TLLRQETESLYFSKLADLKALAERHRNAAVNSQDEVRKLRSTVDDLTSEITTIRAQ-<br>NNALIARIKDLEKQLRQQDDHLEATTLRDKELQQLRDAVAEQLKDYDDLLNIKLSL-<br>DNEISTYRKLLEGEETRLRITTPPRKTKSTGRDPRPTKRRRVEDESSVTQRPTNN-<br>GVVAIVESDPEGNFVKLHNNSDTDQALGGWHLKRSVDGGSQQSYKFTAKYVLK-<br>AGQEVTVWASGSGKSHSPPKDLVYKNVESWGTGDNVETSLLDASGEIMATR-<br>TVIKEVTTHEYREGREDRCSLM |
| Daphnia pulex | Metazoa | NCBI\|<br>EFX71199 | MSTRSKKTPASSSQASSSASQSRSSPLSPTRLTRLQEKVELQNLNDR-<br>LANYIDRVRQLESENNRLTLQITTTQDTISREVTSVRGSYEKELSDTRQLLDE-<br>TAKEKARLQLEAGRARAELGELTPKYNKAKNDLLQAEKKVQQLETRNNELNASL-<br>HQLKSDMARAIEEKKDAVSERDKLAKQVPELQQQLETELLARTDLENRNMTLK-<br>EELSFKQQMYEREMTEVRSNKQVEISEIDGRLQEQYQARLQSTLQELREQYE-<br>SQIQQSRDEVEVLYQNKVEDLEDQVKKQRGAAGAHYEELIQARNRLKDANLK-<br>LSELELANSHMNDRLAEMERNMETERQTHATAMQDASAVYSSMCQGEITRL-<br>REQMAIQLQEYQDLMDIRTALDMEISAYRKLLEGEETRLKLTPTAGASTSQSTR-<br>SGTPSRRTPTRALKRKRTMLEESSSYSLADFITSSSAKGEVQVEEVDSEGKFIR-<br>LINKSEKEVSLSGWQLVHKAQETETIYKFHRSLKVAPGGTVSVWSAGSGTAHE-<br>PPSTLVMKEQRWFVANEMVTQLLDNSGEEMAQRESKRAQTSTSILRQREEY-<br>RGLGGPEELHHQQGDPEGKDRCVVM |
| Drosophila melanogaster | Metazoa | NCBI\|<br>P08928 | MSSKSRRAGTATPQPGNTSTPRPPSAGPQPPPPSTHSQTASSPLSPTRHSR-<br>VAEKVELQNLNDRLATYIDRVRNLETENSRLTIEVQTTRDTVTRETTNIKNIFEAEL-<br>LETRRLLDDTARDRARAEIDIKRLWEENEELKNKLDKKTKECTTAEGN-<br>VRMYESRANELNNKYNQANADRKKLNEDLNEALKELERLRKQFEETRK-<br>NLEQETLSRVDLENTIQSLREELSFKDQIHSQEINESRRIKQTEYSEIDGR-<br>LSSEYDAKLKQSLQELRAQYEEQMQINRDEIQSLYEDKIQRLQEAAARTS-<br>NSTHKSIEELRSTRVRIDALNANINELEQANADLNARIRDLERQLDNDRE-<br>RHGQEIDLLEKELIRLREEMTQQLKEYQDLMDIKVSLDLEIAAYDKLLVGE-<br>EARLNITPATNTATVQSFSQSLRNSTRATPSRRTPSAAVKRKRAVVDES-<br>EDHSVADYYVSASAKGNVEIKEIDPEGKFVRLFNKGSEEVAIGGWQLQR-<br>LINEKGPSTTYKFHRSVRIEPNGVITVWSADTKASHEPPSSLVMKSQKW-<br>VSADNTRTILLNSEGEAVANLDRIKRIVSQHTSSSRLSRRRSVTAVDGNEQLYH-<br>QQGDPQQSNEKCAIM |

| | | | |
|---|---|---|---|
| Amphime-<br>don<br>queenslan-<br>dica | Metazoa | NCBI\|<br>XP_003386<br>754 | MSQQQATSSPSLPTPERRTRMKEKEDLQLLNDRFVTYINRVRRLRDEKEKL-<br>NVTLEHMQTTTQEESGAIKRMFEKELQDARLLIDETAKEKARYQIQSSKT-<br>EDRIKDLETELKEFREDYDRMQQELAEAKKSAETFESLYKSSLVDNQSAKQK-<br>IQDLEEEIDDLRARLREVQDSLEQETLAKVDIQNQNQSLREELAFKKKVYDEELLTIK-<br>RTGLTTVHRDGVEETDDDFAERLQAAIDEAREEIEKETEQFKLDLEKSYKTRLESLE-<br>SQSQRDASARVRLDGELKNCHTTLAKYNRDIAKLQEKNEQLESSLREKSEDLARA-<br>KQLHSAELANLMDEMRALRVNYDQKMKEYEELFDLRIQLEQEIATLSALLQEEEQR-<br>LNLQTTPREKRPRVARDGEGPRSKRKKQVAATSSSAVGFIQIIDVDPDGRFVQIKN-<br>MSDKVTEIPKNKLQLVGVTAMLIASKYEEMYAPEVADFVYITDSTYSNTEIKAMERNI-<br>LKTLDYSFGNPLCLHFLRRNSRAGDATPQMHTMAKFLMELCLPDYSMLEYLPSLVA-<br>AAALYISNKLYSDGEWTPALRHYSQYTEPDVLPCVGKMASLVLSMHTAKQQAVKN-<br>KYCSSKFMRIAKEPCLQGKIMKELASVSLSQ |
| Hymenole-<br>pis micros-<br>to-ma | Metazoa | NCBI\| CD-<br>J06754 | MSARTKKSKQSQPVAEKLATPKKVERSSTTISSPVQSSDAEYRSPSPLNISRVDE-<br>KEELAHLNDRLASYIDYVRKLERDKESLTRRISTITEERLAKVDNARSTYENEIQSLR-<br>RLVDDLAKEKAARDVELKKHMDDASDAKSKLNKRDVELRNLQRKYDTLERDIG-<br>TYKSDHERYQKLLPEYEHLEKSLEAAKEGLKAETILRTDLENKVASLREELDFKD-<br>RLFEEERSKLVMRSLTVEEEVAETKAKEYESRLADELQAYRESANEELEQYRLQ-<br>MESTFQNKLEQLQKANEEANRDGGRLNEELIAMRRMNDDLSHELARKSREVEL-<br>LHSRIADLEKLLNKAHDDYQAQLAAEREEVKRLRAELEQRFAEFTDLMNTKIVLD-<br>QEILMYRKMLEGEESRLNLTSPKRHSVFSGGFMGVKRRRLDSGDGFDEESNS-<br>TGSQTAIRVSTSAVGSIEFSADQDGSGNWIKLSNVGKEDINIGNWVLKHKSDGE-<br>EVTYKFGRNVILKPGTPCTVWSVDAGATHDPPEDIVMKNQRFHAGANTTVTLT-<br>NENEEEQASCLIVRESRPKIIPRRFRAGAARGEDKCSLM |
| Aplysia cali-<br>fornica | Metazoa | NCBI\|<br>XP_005094<br>526 | MKQTRKTVTTTYSSSSGGGGGAGGEEAGTSFSSSSSQSRRSGGLGRPPSPARL-<br>SRIQEQEELQGLNDRLAAYIDKVRSLEAENSRLALQVRSTEEVVKREVTSVKTLYE-<br>SELAELRRLLDETSKEKARLQIEANKYKTDYEDLLAKYNKRDRDANALER-<br>RVQVLERQLAEFSARDFEKENQNLKNELNRLEQQLAAAKKQLEEETLLRVD-<br>LQNRVKTLKEDLHFKSQVYEQELEESRVRTTTQLEEVDGRIEQEYEARLQDA-<br>LREIRAQHDYDLQSVKVELETLYESKVEDLRNQLERNSQSSSAAWEELMLA-<br>RRSVDDLSAEIGRLKSENAGLDLRVKDIDKQLERERDEFRLRMKQRDEEISR-<br>LRAQVEELEREYAALLEIKIKLDREIEAYRKLLESEETRLNISASMTEQRSMTT-<br>ASSSSGGRKRKRMDIGQAVEEYTQRTASSGFSRSATSSCGVDISDVDTDGK-<br>FVKLTNTTDKDVHLFHWQIRHVSGDNETRHKFQKNVLKAGQTITVWSADSD-<br>QTHNPPSDLVMKGKRWFVSGDMTTTLLDQDETEVASCSMSRNIGSVASYSTRRR-<br>GPRDELDGEELQDKDKCFVM |
| Monosiga<br>brevicollis | Choano-<br>flagellata | NCBI\| com-<br>pound of<br>XP_001745<br>977 and<br>FE885114 | MDISGFVSRAFTPKPSPKRRAVDLDNSAAQVSTRELERQRLEDLNHALSRYIS-<br>RVRALETQVVTLETHIESFASVKAPKNDEVLETLRAQLEQAKAELD-<br>DASQRYADLQLEHSNLQEDFKRAEGDIQGLTSLKERLQGRIESLQAMVNR-<br>LEGQLASAQSSLQEEKEDGDLARQQLKDLKADLVKHRGRADSEAKARV-<br>ALENEISGLQESFRLKELEHQSETDALQQQLQVALSQVVVSVKTTGNQ-<br>YEEEMSRLLRVAKSSYAEAADAFREKLKKYYQMNPTPSAAPSMMQDP-<br>ELKRLQAENEALRNRVAARDQDVADLKNEARHAEDKIDRLTHDLASTKA-<br>ENKAHLANKDMEIKNLQQRISGLEDQYRVLLDRNLSLDAEIDQYRGLLG-<br>EEYKRLNMHDQSLDAMDQLVGSPLSAQRDEQAGEQDLDMSVELRHM-<br>TEEVEEAWTQRMSSNGQIQIQSVDLDRGFDLHLQNIGQEPINFDDCVL-<br>TVDNGRVSETLVMPHGHVVAVGQVVRVVSGLGEQSHRPGDIAWTSFRD-<br>FEFDNTINVQLERGSQVICRAEYTCEDSTGATRCCMM |
| Salpingo-<br>eca rosetta<br>1 | Choano-<br>flagellata | NCBI\|<br>XP_004988<br>923 | MADTASTPGSPQFRRRVKEREQLDELNNELAAYLVRVRELQKENGELTSQLEAIRF-<br>SRKRFESTATIKLEGQVNDLLEEIRRKTTEIGTLNHQVQDLRKQLNK-<br>TEGDALEATREKDDLAKQLNEALSKVQELEGQVSGLQRDVSQRDTTVTNLQR-<br>RVASLEADLGRQVDDTKRERLARLEAESRAQGLEDKLSSQDDMYQQQVTALEEQ-<br>IKIGGTSGGLSEEDLEAALTEAKEHYKQAVNNFRTQMRSYFAQQPIIEPDTRGLEE-<br>RARLQTEVIEWRSKYEDVKAQGATAQTEIEGLKSKLAGLEDVFAQIKKSHTDEIRH-<br>KEDFIFKLTKTMQDKDDLYRDLLDERIALDAEIDRYRRQLESALRMFEARSPGTG-<br>APQMVASVRKTTRELVALSGRRAVSTKRRTAAQADGGDGGADGELGDGSPKAK-<br>MSRLAEEAHSFTVETNTQGALSVEDVDYREGQYILLKNTTASPLDLADWAVTVTN-<br>RDTQLGTFEFPAVTLAPGRSTRVIRGAGDGAEKLEGDVVWTEFPDLAAQPELAVY-<br>AFDPDDNESSTWAIMRD |
| Salpingo-<br>eca rosetta<br>2 | Choano-<br>flagellata | XP_004988<br>925 | MASFDGGADAANNSSHAAIAAVRNTMKEREEMEKLNDNLEVYIRRTSALYSSH-<br>STLVSLQASRRTSVVASTPTASPSKSNEERLRKEVKRQQAEIDRLQS-<br>LASQLEKQFENAVEVKDTALNQLEECAATQQDLRTELEKQKEAYDAR-<br>LASTEATISSMTAELEESRARAHAAERRVIAAEAEVVTLKHQLNHQQSLHETTVT-<br>TLEEELQACTKAMASKQAPDTTIDAAVAEVKKEFQAAANAYRHKLRQYYATHAR-<br>GPQAMSSSWFTEERMALKQQVFTLTKEKADLSTTNASLHATVDACKQEVAAIR-<br>QSMKDAVHHHTTEIKAKEERIAALTAHIRANEKKYADLLDERLWLDAEIDEYRRLL-<br>NSIQRPPRPKKKKATPTRLSLPAGTPAPRTQATTAPVESDKSDASSGGPGRLEL-<br>RDVDTKVECLLVANPSKTSSVDIDGWALTASADNDLISTLEFSAHVLPPGGVLR-<br>VFQQRNEHFVENRDCLWDELTRPREWGDGQKGAGSDGEGL |
| Capsa-spo-<br>ra ow-<br>czarzaki | Filasterea | NCBI\|<br>XP_004365<br>259 | MSSPDRAARLEEKEELQFLNSKLELYIHRRQELQTLVKTLESELERERNDAQQR-<br>LEIAHRQYEATLTETRHALDKAASEASHASSSLVQVNAELAATKERARTES-<br>SAADALRSRYEALQLAKHELDGRLAVSEIAIKELKDNLKHADSDRKHAEKESDAL-<br>RKSSEKEAQARIAAENALARFKQDAQASAKTGADLNAYHSEEMRRLRSDLS-<br>SLERKLRAEHDQELAEALSEHRRQTEAESAQYRAEVQALYESRLEAAREQA-<br>VKDASVVAGLQQALSAAQAAQDRARAEVTALSQRSETLSTRVRTLEHELIIE-<br>RDTVVEVQRLREEATRLMQEAIAAKDSEVRTLTETNVALDKEIAMYRTLLEVE-<br>EKRLGEDFTPTRRGGRDRAASTSVVASGATKRTFSETAEETNTTPVRSKRR-<br>REEDPSPSRSASSSSATFFAGAPRSVVNAATRALFTSSASSSSDASSSSTGT-<br>TLWTSEPVTEAGLVVFEPVNLVEHNKLVLRSTNPEEEVSLHGWKVRTTSETT-<br>GSATYEIPAGIALSAEHPTVTIWTGVSATRGKRKSRSELLWKQDINYASNGAC-<br>IQLLDNEDNEIARLEVREQKEDELFERQSDPRVGCSIM |
| Creolimax<br>fragran-tis-<br>sima 1 | Ichthyo-<br>sporea | Origins of<br>Multecellu-<br>larity db\|<br>Cfra_2736T<br>1 | MPQRRTPRGGTKRKPVETNKEDEVPNTPTTAAEPPADPLSPM-<br>VAKRLEDQIRLTQLNSKLKEVIEKMENAQPTNEARIEIESTCARLKEDEDNSLNREL-<br>ERTHAKMADAQDRKKLLTKEKANIEKKDAEAFKNKTKRETEAKALHKSAEKL-<br>RKQTKDLQSKIDDCTKQVGNIRDELAQANQALEQLEASVAAFVDTKRTLGEK-<br>SAEKDHAIDDLNQELDLYKQEETDRNQSNKEALQHLRARIAGVAAKKERELQ-<br>TKAAEAEADVVGVFNRALQKHVNDIKVLHEKKLETAIQTDVEDKAEVKRLDGK-<br>IKDLKKLRKELEEVNFRLDNKVDSAQKQKAIIAELEKQLENMKKKHAHVEMD-<br>CKAQTQQVMAEKEIVQLAYLQAEKAQKLLGDQINEYEKLLDFALNAHKRAR-<br>DDDSDEERARKKTKTDEEEPSIIEVTITGIVELVAVDPQGLYVTLVNETEDEIHL-<br>DDYHLIGVSHDEVSTRRFDFSSDCRLDARGKVTVYTNAEEYERSIGNAQLW-<br>ETDPVWAASPEGPSQMKLYNSGGLVSAATISVSEEKKTGENCCIM |
| Creolimax<br>fragran-tis-<br>sima 2 | Ichthyo-<br>sporea | Origins of<br>Multecellu-<br>larity db\|<br>Cfra_6445T<br>1 | MTQQTDAYKQEQERDLRELSKIVLLNKVLGEKIKKYAAMNPDPAQKAADELKA-<br>SLMEEVEKLRTDNKVQIDVLHEKLNTIVSQCDALETENRLTTDNEAAL-<br>VAQTEAIEVEKVKLMDDIESLHEVHADLQDLLKRKRQECKELESETGKAIKKKKR-<br>LEETERKTAQQCDSLTSNNAEKDKVLADLRNEADVKNEQMKREISDHENVITNLTIE-<br>LQKTEDAAKAEYAQKFKKIASEKRDEMKALLEEKKKQRTAELEAELQSALPIDLKKSI-<br>TKKQNAVNKAQAEVDELSTIIKEKEASLPDVEHLKQAAEHIKADIKMWKERIDTLKK-<br>KTEEEIAELTNEKLGHDEQMTQLDRYGITLEEQLNNYRDLLHVDLVSAAEGDGVAD-<br>PSHIPVYSLDSINPNTSSVGVRNNSITDVDLAGWTLTIEYTDEDRSTIESAESIKYTF-<br>GDNTMIKPEAIYTVTTDPDESVTHNAVWIMEDDKVDVWGYNNVKIVLKDTSGELQ-<br>CSAVVSANDFSSQLG |

| | | | |
|---|---|---|---|
| Sphaero-forma arcti-ca 1 | Ichthyo-sporea | NCBI\|SAR-C_12785/13776/13427 | MARNKGSARGASRRKANSTSQATPTPEVENTKDTQENTVTNES-<br>LLKSPAKNGKESRPSSPLVKQRKTAKRKLEALNAQLANVIHRMEHAEPTEEDRAEV-<br>CIHVNLIESVCXXXTIEEYARLLDYASNGFKRKHDGSDAELDASFLQPTNKRPR-<br>VENEDSFHVTITGVVSLKEVDPKGTKVLLENQTEEVVDLSGWHLVGVSHDK-<br>ATARYDFDKDATLNARVCSVLVFSYGYGYDIPXXXWKTDPVWSASKEGPST-<br>MKLYHEGGLVSAATISVDESAEQSEGCNIM |
| Sphaero-forma arcti-ca 2 | Ichthyo-sporea | NCBI\|SAR-C_07948/15657/16962 | QQLEKETGKLFKKKKKFEDTERKTAQKCDDLTVEIKDLDGLLVQRQQEADQKNEN-<br>MKEAFKAHEATVNALKEELXXXVDLDEQLNDYQNLLHVKPSADGLFAPTTTP-<br>SLYIDHIRPAQSSLGVRNNTERDVDLHSWMIRVRYTDETVDASGYDLPGTQHI-<br>TYTFSGPAIVSPDQLYTLTTDEHERSTHDEVWEIQDDSLDVWAYRTVYLELLDA-<br>DKLLVSTAVV |
| Pirum gemmata 1 | Ichthyo-sporea | Origins of Multecellu-larity db\|c30326_fr4 | MLSSFSDMMRTTPDASKKSPKKSPNSTHNSAKGMLSPASQERKAEFERLCNL-<br>NGRLKYYIRMVREKYPEELERVHNQHQREMASQKDRSDNEKQEMKEHFNGKIQN-<br>LEEEVARHSASNRESLKKIDQLHREKANFEKAIENLQTDLVKKENHINQLKEEKK-<br>TSSVLVQELEEELSELKKELADAQDEKAGAIQEIEENLRS-<br>QLNSQIELVDKLTEELEETKSNLENNEQELNSAYEERLE-<br>IALAEAKKVHQQNADKMLDDVERSYEKIFAEKEADIKRV-<br>TQGLQKENDELKEQKETILQEGEEIKNENNELQQSVVV-<br>LKAKIDELEKQYNNVSKSKANLTREIDSINKLLTEEEARL-<br>ERDIAKEKTKNKESSVVVDDNTGGSRKRGRDDLEEDEE-<br>DVEPVPKRSDVDKLQVDIIDSSGDVIIKEVDAEGFFIRL-<br>QNVTSAPIQMDQWLLRSYYEDDAIDFNFSKDLVLEPEQYL-<br>TIITRLDEEEIQPDSEQELNVQWDTSEPLWNNEGGFVELFNQEGASVSAAQI-<br>ELEKRKTCIQQ |
| Pirum gemmata 2 | Ichthyo-sporea | Origins of Multecellu-larity db\|c13682_fr2 | ELQNGREVLESKIEDLKVTLSSKTQKFTEEKQNLQDSLDNAERELNEIKTIFKDQY-<br>EEKLEKTRQDLAEEYNARLEDALLNARDQHERENEEFELRLNNEHQEELSELNVK-<br>MMKNESLLKQSIAKLKQVNDTNDELRRELSNQVMKEEQKEAEKKELENQLVD-<br>VKKSFEQLQADYEEQVNENDSHRRLLDEVAEENSDLRTEIDYTFKMIEQEEARMG-<br>MKFQVDPKTVKTNKTSSRVFSDNYPQPSFGEDEDEEEQDVEDVGSKRSKNIEFQ-<br>LQSSINQHSTTFTLDEVDPEGNFFYLKNNSKKPVDLSTWVLRASSASRDYVDFHF-<br>PDNITLKGSEKVAVLTSQNTEESDMKLDRREFNYVLFWTNVTSIWANDKGSVNLFK-<br>GEKLCSAATITEEEVQESPAKRFSSYIFGL |
| Abeoforma whisleri 1 | Ichthyo-sporea | Origins of Multecellu-larity db\|c11593_fr4 | MTSAPGTPANQTIDTKNMLSPQSLAKEQKRLADLNDRLSDLFSGLRGKRDDIISGY-<br>ERKIQDLNKQLLEAGEVLHNLEADIAERDAAIQKLEIVKQN-<br>LEASREDLQKVYESKERQCEDLVEDREQKVS-<br>RFHREIQLQVEKLSEEKAKNKEMTKELKEVNKQLNSASQKLEKAIDEKSEAINK-<br>AKQLEENLDFKTKELNAQIEKLEHDLKEKDEENASRLEEAECDFDVRMQLAL-<br>QEARQLFETDLESQLEAVREELNNDLVEKESKREAEMKNIESDLENVNSLNEA-<br>LREELKTQTAKGAKAAKKADESHAKEKKKLEDQLEQLKANFENEREQNVREA-<br>EDRYSKMKQKLEDEKEQCQKKMEDQLLQMKKELEKERKQSEKNLSDWRKT-<br>KKKMDEEYQREKETQKAANDLAYDELKAEYENLVQTITDVTNLLELEEERLGIP-<br>IEQENRLSTTVTKLMKKHQTPSRTGGKRTAQQAEPANTSQFGKKMLRFDESD-<br>DESENDSVGKQVQITKYGHQINFDEIKTNGQYIKIKNESEENVSLTGWSLKCTF-<br>ESEEITFTFDDYELASEDSVAIVTSSDYMSNIKGNKILFETEPFWAVDNGVVEL-<br>YDDRGDVICSAVVEEYDNLNKSRNGPLCYQAQQ |
| Abeoforma whisleri 2 | Ichthyo-sporea | Origins of Multecellu-larity db\|c11301_fr3 | CTATISCLYVIALLSLLNMSMVKKPLQSRNLNLNSNYEATPVRKIVKDSGQ-<br>NARLQNLNGRLKYYINLVRQKHPEEMDKVKNEYEVKMKDLQGQI-<br>NALQDEISELNSLNVLQEKELKEKNASLRQKDDLMNKQEQFTEGLEQSIADLQ-<br>VANKVLTNKERDLKAKVSALKEDYDELCEANEALEKLRVKLEENMKKAADN-<br>AAEQKKLHAIEMKLSHDQFAEKERLASEKHSNEMREMSEEFFEKERNLKNE-<br>LKQAERQVEKIKIESEKATTEILGKAVSDFNGKFEQKENELKEVTHKLNKLAD-<br>SKKTLEDENAKLLQKIKDLELNSEDLYKKLTKVETKLENKTRKYHDSSQTILEQ-<br>LDIYNNLLLEEEKRIEGLPKFSEASSSRFFHESPQFSSAVAAPLEDDDKYKRP-<br>RESDDENSEETEYHEKRQNTVETQLQSEIVSSTGSIVLEEIDATGAFIRIFNN-<br>SKHPVCLSGWNILVKNPSGEIGQFCFPDDFTLAASGKVSVTSPSFNDNDDD-<br>TEVHLQWNEPSIWSSDGCTIQLFDMNGIIASTGKVEEVEVGVDLSTSIWGLFSSFLG |
| Dictyo-stelium dis-coideum | Dictyo-steli-ida | NCBI\|XP_636248 | MDMSKKKSKRASPIESSQEEIAISTSKTATTEKPKKTKTTTKKKASQPSQEVVMETE-<br>SEVEITTTTTSTSTTNNNNITTTSTSSQQSNGTLSSSSSPTIQSIPTTPISKYIP-<br>SLSQIGTPLSPNRAAQRLREKDELSLIHNRLKSALKKLESAETELEKKNQEY-<br>EELDQKHTATIKQLKQRSDQVEKQLIEEQNQNSDLTSNRNI-<br>LENELKSKESVWKKEKDEILLKFQESINKLNQENSLAQSQLKS-<br>EIVSKEYEIDGLKSEINRLKDDLQYRIREGEEKSRKLLENEYN-<br>RFKGKEEEYNQLIVSKDEEIKKYKFELKEKEKSSNAMNKKE-<br>NELNNLIQAHERQIEDMRDSINREWELKAAQMMEEHHARTI-<br>HLQQAVDSFNEEKERIKSQMETLNGQIEDINIKNNEYEDRIK-<br>EMNVLLSQKDNSIGELGVEIEESKKKMRKQMADLKSKDGQIA-<br>LLQIEINTKDNKCNTLQTETNRLKSELYSITNQIDPEIPLDPEI-<br>NSLKELVKGFEKTVDDRKRKRSKLQHEFNAAANQDQNGMT-<br>IEEQSSTSTTTTTSATGSSSSTSHLDNIDSSKLPTGPEQSEL-<br>FNPDTVSFSLVDSNQEFIKLSVHGDMDNGLSISKWRLIVVK-<br>PDGSKSGFSFPDGIQPFKGIKSVTVWTGRPRPQGTPTENE-<br>FYWARTELWTSPVEGTIVKLVSPSEETTTVTLPADGIYQKPSSAGKSNCLIM |
| Dictyo-stelium purpureum | Dictyo-steli-ida | NCBI\|XP_003294572 | MESTKKKTASKRSQSPVDVAEKEKVVEEEVQTVAPTKTKKGKATTT-<br>TKKSKSKEVVVSTPVSNTVSLSSADDMATESEIEPSQTTTTTTTTTTSNGTTTST-<br>SIPTTPISKYIPSLSNMGTPLSPNRNALRLKEKDELNNIKSKLQL-<br>CLKKLESTENELEKKNKELDDMEHEHSLAIGNLKNRNEHVEKQLIEEIKTKSELMS-<br>SRDALESELKTKEASWKKDQDEYVTKLTETINKLNQEHSTIVSQLKTDIAAREYES-<br>DTLKTEVNRLKEDLQYRMRESEDKSRKLLENEYKRMKEKEEEFNQVLLIKDEEIK-<br>KLKVEVKEKDKLHSASSKKENELKHLIEAHERQIEDIRDSINREWEVRAVQMVEE-<br>HNARTFELQQSVESFEEEKQRYKGQISLYGAQIDELNIKNNEFEDKVKELNDLIAQ-<br>KDSSIGVLNNELEESKKRYRKQAADLKNKDSQISLLQVEMNGRESKCATLQAEIS-<br>RLKSEIFSITSQTEPDIPLTREIEEIKTLVGVFEKNVNERKRKRNKVDDNNAANVNQ-<br>TTADPNISTDIPSSAAQVDPEELEKFNPNTVTFAVVDTNQEFIRLSVHGDYDNGLS-<br>LSKWRLIVAKPDGSKAGFSFPEGIQPFRGIKSVTVWTGRPRPGDSTTTENEFYW-<br>SRQGLWSQPVEGTIVKLVSPSEETTTVTIPETGIYEKVQEEKSKSCLIM |
| Dictyo-stelium fas-cicula-tum | Dictyo-steli-ida | NCBI\|XP_004349984 | MIIVQKEEEEMEPTKSKSSSASSVKKTKKTEKRDVAAEESAHEESDVQVKD-<br>KRKKKKSTAVPKDTAASSSSSSSQVDQADANSPSMAIDEPNESPRSSTTVT-<br>STSSTSSTTTTSNVTKTPQHNGSISHNISTPIQTPSFQKLLSMTTPLASPG-<br>GRSSTAARLKEREDLIQLNSRLRDLLASSEQKDNEIRRLRDEYITTSRQSTDQISN-<br>LEKKLEDIEARLGNESKLTVELSSQLQLAHQDARSKEQGHQAEIQNFQNKMDETIK-<br>KITEENSQLVVALRNDNSKKVMEIESLKSDVRQLQAELLTRSKEAEEKAHRILESE-<br>YNRTKLKEEELRNHINQKDAEIKKLKTELKEKEKLQVEYSRKESQLQQTIEGYER-<br>QAEDMRDSVNREWEIKCVQLVEEQNAKITQLEISANSFEDEKEIYKSQLQVYQGQ-<br>IDDLNIKNHEFEDRIISLQKDLDTKDDTISQLAKEIDEVKKAARKVQADSKKKDIAIA-<br>MLQDEINGKDVKCNSLQSEMGKLRKELIMFTNASPEQDIPLTREIEQLKSLVNNFE-<br>RRVKRKREEDEDEESNQDEQMNGGQQQQQNQQHQQQHDQQQKQQSEDFQV-<br>NTISLLSIDAISECIRLTVSGDFEDGVSISGYKIVVTKPDSSRIGFSFPENIVPFKGLNI-<br>INLFTGKTRPGTTPPNEFYWSRPNIWESPEGTIVKLLTPTDEVLANVTLPSNGLYHK-<br>DDKQSNCLIM |

| | | | |
|---|---|---|---|
| Dictyo-stelium cit-rinum | Dictyo-steli-ida | NCBI\| AJWG0100 4320 | MDITKKKSKRSSPIESSQEEIAIXXXKYIPSLSQMGTPLSPNRAAQRLREKDELSLIH-<br>NRLKTALKKLESAEIELEKKNQEYEELDQKHTATIKQLKQRSDQFEKQLMEE-<br>QKQNSDLASNRNILENELKSKESVWKKEKDEILLKFQESINKLNQENSLVQSQLKS-<br>DIVSKEYEIDSLKSEINRLKDDLQYRIREGEEKSRKLLENEYNRFKGKEEEYNQLILS-<br>KDxxxSSNAMNKKENELNNLIQAHERQIEDMRDSINREWELKAAQMMEEHHSRTIH-<br>LQQAVDSFNEEKERMKSQMETLNGQIEDLNIKNNEFEDKIKEMNVLLSQKDNSIGE-<br>LGVEIEELKKKMRKQLADLKSKDGQIALLQIEINTKDNKCNTLQTETNRLKSELYSIT-<br>NQIDPEIPLDPEINSLKELVKGFEKTVDDRKRKRSKLQHEFNAAANQDQNGMIIEEQ-<br>STTTTSSANSSSTSHLDNIDSSKLPTGPEQSELFNSDTVTFSLIDSNQEFIRLSVHG-<br>NWDNGLSISKWRLIVVKPDGSKAGFSFPDGIQPFKGISSVTVWTGRPRPQGTPTE-<br>NEFYWARTELWTSPVEGTIVKLVSPSEETTTVTLPADGIYQKPSATGKSNCLIM |
| Dictyo-stelium fir-mibasis | Dictyo-steli-ida | NCBI\| AJWH0100 3706 | KYIPSLSQMGTPLSPNRAAQRLREKDELSLIHQRLKSSLKKLESTEIELEKKN-<br>QELEEIDQKHTLTINKLKQRSDQFERQLLEEQNQNSDLTSNFNILDNELKTKESS-<br>WKKEKDEMLSKFQESINKLNQENSLVQSQLKADIVTKEYEIEGLKTEINRLKD-<br>DLQYRVREGEDKSRKLLENEYSRFKIKEDEYHQLISSKDxxxSSNAMNKKENELN-<br>NLIQAHERQIEDIRDSINREWELKAAQMMEEHHSRTIHLQQAVDSFNEEKERIK-<br>SQMDTLNGQIEDLNIKNNEYEDKVKEMNVLLSQKDNSIGELSVEIEELKKKLRKQ-<br>LADLKSKDGQISLLQIEINTKDNKCNTLQTETNRLKSELYSITNQIDPEIPLDPEINS-<br>LKELVKGFEKTVDDRKRKRSKLQHEFNAVANQDQNGMIVEETLQTTSTTTSSD-<br>NGSSASHLNNIDSSNLPTGPEQSELFNPGTVSFSLIDSTQEFIRLSVHGDMDEGL-<br>SISKWRLIVVKPDGSKAGFSFPDGIQPFKGIKSVTVWTGRQRPQGTPTENEFY-<br>WSRQELWTSPVEGTIVKLVSPSEETTTVTLPVDGIYQKPSASGKSNCLIM |
| Dictyo-stelium in-terme-dium | Dictyo-steli-ida | NCBI\| AJWI01001 930 | KYIPSLSQMGTPLSPNRAAQRLREKDELSLIHQRLKASIKKLESVEIELEKKTQEY-<br>EELDQKHTATISKLKQRSDQVEKQLIEEQTQNSDLSSNFNILENELKSKESS-<br>WKKEKDELLLKFQESINKLNQENSLLQSQLKADILNKEYEIESLKTEINRLKD-<br>DLQYRIREGEDKSRKLFEFEQSRFKTREDEYNQLILSKD |
| Polysphon-dylium pal-lidum | Dictyo-steli-ida | NCBI\| ADB-J01000003 | MSETTSASKKKSSSKSKRSSEKEKEKELSSSVINTSNDGDEEMSEPSTPTIEKKEK-<br>ERKKKSSSSSSSSSKTRAERGDKSGADKSTTVTSSTSSTTTSEKDTGAGHISS-<br>MQVEESTQSVTTSSNLNNNNVSKTPTHSNANGSGITASSLPMTPLTKMFGLGSIGT-<br>PLQSPSRLSINRMKEKEELTEVTKKLSKILTILEERNNEIVRLKAELNDSNKSSS-<br>VEVSALRTRLDETENRLTQEIKQRAEAASTLELTLADLKNKEDLYMHDRNTFD-<br>ARLEDTISSITKEHSAQLTVLRTDLNKRLHEIEQLKGDIKLLNADLLTKSKEQDE-<br>RTHRLLDAEYKRMKQKEDELLNLISQKDDDIKKVKLTLKEKEKIIIANQRKEAEL-<br>NQTVEAFERQVEDIRDSINREWEIKVAEAIEEQTSKYASLEMTAKSFDFERETF-<br>RNQIAVIQQQTEDLNVKNHELSDRILDLSNELRNKEQQIITLTAEQEELKKNLRR-<br>SQAELKSKDGKLSLLQDEISSKEIKFQQYQSEMHTLKKELHSANNQSMDPEIPI-<br>GDEIRRIKELVNTLTHQSRPHKRQRSGEYSGENNNNNGNELAANHEEEMSD-<br>AEQQSPSSTSNSVEPAASTLKLNTMCILSIDTTKETIRLNASGDHVNGMTITDWK-<br>IIVNKPDGFKYGFQFPANITPLKGINVITLHTGRSRALNTTAAENEFYWSRTGIW-<br>DSPEGTTIQLVSPKEVLQESVSLTNGAYVREESNPKEQGNCLIM |
| Polysphon-dylium vio-laceum | Dictyo-steli-ida | NCBI\| AJWJ01000 603 | TPLNKMRESLLGTPMSPSRSVMRLKEKDELNAIHKKLQQCIT-<br>SLKEKDEEIEKKEREIESLQKEKTKSTNAYKTKLEEAERLLSQEIKEKTEFLSRAD-<br>FLESELKTKEATWNKDKQDMISKMDEAINKLTHEHTLSLSSVKSDLVKSEYD-<br>NETKKNEIARLQGELHTRVKEYDEKSRKLLDNEYNRMRGKEDEFNSLLKQKDEDI-<br>KKLKMELKEKEKLSLVAQRKENELNQTIQSYERQLEDIRDSINREWELKTAAMVE-<br>EXXXERERFKTQITVIQEQVNELNIKNQEYQDLIEQLNNTLMNKENNLNDLSQEN-<br>EDNKKKLRKLQADLKNKDSQISLLQDEINSKETKLITYQNETTKLRTEIQYINNQND-<br>PEIPLTREIASLKELVNGFEKSVAEDSSRKRKRSKXXXQEYIKLSVHGDFKDGFCI-<br>SGWKLIVLKPGGKCGFSFPENIQPVKGIHTITVWTGRSRPTHIQTPENEFFWSRE-<br>EIWTKPYEEITLKLVAGVSNETTAKVSLPETGVYQKDPSGKSNCLIM |
| Pavlovales sp. CCM-P2436 | Haptophyta | MMET-SP0985\| CAMN-T_0021991 105 | GASRNRRPSCSPAFASMSESGRRSSIGAGPESATRLTRVEERETLQNLNSR-<br>LQYFLLRMKELEARNGELQTKVDSGSAGAKAHNDSMRAVFEKQIAELRSAQETD-<br>VELIAELKSRAAMSEAKASENGKVASAYKKKAERCDEAEVKLRAVIEERD-<br>KALVLFKNTEERAEKAEQAAATFEAKASLLEKKLAPLREQLSTAEELRRKEASTF-<br>RETIDELSGANRGELSRMQAAYDAQMQDALDVAKLRAEEGSRAELEQLAAHFT-<br>SVESELTREVAEEKAAKESALKRANDLAANKESIQARVSIAERRVTELERTLADN-<br>SRESDGKCAKLEQALEVARSERKTKEGEFNELMDVKISLDEELKTYNMLLGREE-<br>ARLGITPTQDRTKRRRITQGPMPYVSQLNLNDDCVAVKNAGDEPALLGGYTIKS-<br>VHSEDLVFVFPSEFELLPGASVTIWSGKKNQRRRRDPQTELWWSARYMWSD-<br>DGDMATLFDTTGAEVSKLEAAAGTVADAPDPTTDYEEPGEESEAAAGGERQCAIM |
| Pavlova sp. CCMP459 | Haptophyta | MMET-SP1140\| CAMN-T_0007433 825 | FSSPRNLRERRKTEHHSSRPAHAMSESRRLSTGSGSDSATRLSRVEERDTL-<br>HNLNSRLQLFLSRMKELEARNTELQSKLDMGSSASQKVTDSMHALMEKQIQDL-<br>RAASEKDTELIAELKGKLAALQASSGENIRSNQAMKAKAERAAELEGKLRVITDER-<br>DRLAAALKDAERRADVAEQAAVTSESKATVLEKKLAPLRDQLQTSEELRRKETAA-<br>YRSQISELSTANKTEVARIQADYDKQLQAALAAAKKRADEERAAEVAQLAEHFNV-<br>VEAQLTREAKDERASKEAALARAAELGKTNDQLQSRADASEKRAAAAEKNLTER-<br>ARQHEATVAELDSLLHQARAARKEKEGEFNDLMDVKISLDEELKTYRLLLEQEEQ-<br>RLGYTPEPVRAAKRRRVAKSGPVPYLAQVDLENDCFTVQNAGDEACVLGGYSV-<br>KAGVADSEGAYEFPADFELLPGSSVTVWSGKKNQRKKRDPQAELWWTARYMW-<br>NNEGDTGILLGPGGEEVNRVTAPANGVGNAPDPTVGEGGDAGGGADSGGQCAIM |
| Pavlovales sp. RC-C1486 | Haptophyta | MMET-SP1335\| CAMN-T_0007433 825 | PRASRSTRAVAPLVRAVGGGPDSATRLSRVEERDTLHNLNARLEYFLSRMKELE-<br>VANDDLQVRVDTAASVHKTQSDQLRDMYEKQLTDMRKTSEKDVELITELKSKL-<br>SAAQKEANEATKDKVALTRKAQLADELDVKLRAVTDERDRLASRAKEAEAR-<br>AAELEKSAAVFEAKANMLEKKLAPLRDQLATAEELRRKESETYRAQIAELSGG-<br>SRSEIDKLKASYDKQLLEAMEKQKKRADEERLTELAQVREHFTAVEAELMRA-<br>VDEEKAARASADERMGDLNAQVRQLRARASQAEAQAAALEKTLAERIAHFD-<br>AAIARLDQQLQEARAARQQKEVEFNELMDVKISLAEELKTYTKLLEGEEERLG-<br>ITPTKEPKRRRFTTADDGGGGASGAASGPLPYLATLDLINDSFALKNAGDAP-<br>APLGGFKVVSAHATDVSFEFPDDFELQPGATVTVYSGKKNQRKKRDPRTE-<br>LWWSPRYMWRNEGDEAIMYDSSGSEVGRLVAEANSIAEADPPTEAAGGGDAGG-<br>DNKCVIM |
| Symbiodi-nium gore-aui | Dino-flagel-lata | MMET-SP1369\| CAMN-T_0049246 593 | MPRRNSSTPRRNSTSSKSSKTSKESSKKEESVSTTVVEETRVVNDQESEIRQS-<br>RVQEKHQLHNLNDRLQNFVLHSKAMEEENADLRRQLRD-<br>MEERMKESLQEVHQEYSKQSKKLRTSVTTMTAKNNGLESENARL-<br>LAEVHALNKKIQQTDSLREENHKLSDQVRDLSDELRKYKAKVSQNEEE-<br>IERLREIIANHEEVIATREDQLQEATKELETVQKTIKKGARHSDGELAE-<br>RVRASLTEAHKKWQRETQTALQKLETDLRQHFKHTTVELNERSETL-<br>VRQNERLRKENERLANELDNNAGGQQDLLNKLAEQERQHKHVVTQ-<br>LQRDLMKREEELTTAASSLVNKNNEFNDLMDVKISLQTEIEKYRSILDV-<br>EEARINTPTQDRHRPSKFEKAQDEAKNNDEEEETTITTKKARKTVKR-<br>SRSTQEIAPEGKKGKGKGKGKNKRRRLKNKKDSEEERSTTRLTTRA-<br>KTGPLHISDVDLISDCVSVANETSAPVPMKGWTLISETGNQVFNFPD-<br>ALVLKPGDRVTVWSGAHAESHHAPPDNLFWTRRYVWNNHGDTAILV-<br>NPAGEHVSIVSGVPSAMSAASDATPASSVTRGTGASVNGGDDQNCVIM |

| | | | |
|---|---|---|---|
| Alexandri-<br>um<br>tamarense | Dino-flagel-<br>lata | MMET-<br>SP0384\|<br>CAMN-<br>T_0004031<br>397 | LTQSKKSAVSQARKAGQQKLASERQKWKEESDSRAAGIEAELREAFTSSSEQLK-<br>ERAQILNTQNVRLQEEMDSLMEELQKANEEAKERLQELQASRASH-<br>SEESQAWAMERSRLEKEVTSLHEAHAEKIDEFNDLMDIKVSLQAEIEQYRAI-<br>LEGEEARLGVDAPSSVFAGVKRDAVIADSEAEDDDGDHSAAGAGAGAGGGGSGL-<br>DEDDVADEEEETPRRSKQRGGRRARKSSRAKSSRTTRSAKRARVEVASSEVGAA-<br>AAEASATSRQLAAAASGVKLDNVDLVSDCVSIKNFTSEDQPMKGWVLKSAVGSQV-<br>FRFPDDLVLPPGKRVTVWSGKTAHRHHSPPYNLFWTRRFIWNNHGDTAVLINADN-<br>MEVSRITSAPGDDGGMRADDDASEEGEGEGEAGEG |
| Krypto-<br>peridinium<br>foliaceum | Dino-flagel-<br>lata | MMET-<br>SP0119_2\|<br>CAMN-<br>T_0007964<br>289 | GEARRSSRSRRTTRSTKKSSSSRHASGTSKRSRKDKGGAGASGTSASTH-<br>PHRHAPSGIKIDNVDLVSDCVSIVNTTGASQSLKGWHLTSEVGDQIFHF-<br>PDDLVLPPGERVTVWSGKNAQRHHNPPYHLFWTKRFIWNNNGDTAVLV-<br>DANNNTVSKITSGPGGYSRDASSDGAGEGEGEGDGSNCSIM |
| Lingulo-<br>dinium<br>polyedrum | Dino-flagel-<br>lata | NCBI\|<br>GABP01011<br>089 | LAVKPEAEPEAAEVKVVISSLDLEADSVVITNNGSEAVSLKGWKLVSKT-<br>GDQEYAFKAKDSIDAGASLTVLSGKKAKAAARKGGADHVFWTARYIWN-<br>NEGDEAGLLNADGEEVSSLAGTGT |
| Hyphochy-<br>trium<br>catenoides | Hyphochyt-<br>ridiomycota | NCBI\| com-<br>pound of<br>CAFC02154760,CA<br>FC02067206,CAFC<br>02215379,CAFC022<br>16946,CAFC020361<br>97,CAFC02052195,<br>CAFC02276476,CA<br>FC02179373 | LNNRLEAYVLRVKEMDDARSHLDHELKTISTRYQKEMDALRERLSRD-<br>VDTLRSELEYEIDQKTVHQVKAEDLTLQVEKMTAQVRITSKLNLEEEEEREREM-<br>DRDEKESCAFKREMTGEPPELXXXXXXXXXXXXTPPTKQELEFKIKQTEDV-<br>GREEAKRYKDEIRRLEKEVSALRDSQAESEKQFNDLMDVKIGLDNEIRHFKAI-<br>LDMEEDRVGVFSPERKKRKKSIKTPKSSAKKARVVAEPAVPDVVVQMIDTENE-<br>CVVIKNNSSEAKDLSHWTLSNKAGTQKFEFPEGFSIPADSSVRIWNGKKNKSK-<br>HSPPENIYWSSKFVWNADGEAALLLSPSGDLAASSAGGEVAGGAETGPSQE-<br>YEEVEEEYEEEERCPLVQGDQEQKCTIM |
| Phyto-ph-<br>thora cap-<br>sici | Oomycota | jgi\|estEx-<br>t2_fge-<br>nesh1_pg.C<br>_PHYCAs-<br>caf-<br>fold_160053 | MAKELSPIKSKRMEEKASLQKLNSRLEMYVLGVNELESAKHAAERELETIKQR-<br>MQQDLESVRIRLTKELEETRKKLDDEVDQNNRLQTLEQEQNRELVKLRAQLKE-<br>FGELKVLVETLKVERDREKTNAESAKQALSEQTTQLNSARRSVKELERDLR-<br>SHKAALTDATQELQDLRKKCANYDMAKDSELTKLRREWAAKHLEAQAIW-<br>KKDAEERLATMEMEVRSHFNSMSTSLESQLDDVRTELESTKKELDRTAN-<br>DYEDSLKARQSLTEKVAQLERDYREARSKSTKDRKAYEETLERFRSSKV-<br>AKEREFNELMDVKIALDAEIMKYRRILDREESRVAITTPNTKGRKRKSEHT-<br>NGTSSKKAKPSSAVQIASLDLEKDRIVIQNTSNEPVSLGGWAVRGQMDQT-<br>FRFPKTYVMRPRSTLTVLSSKRNKSAKHESKKGEAAFLATQFSLNPNGDI-<br>VFLVTDDDIPVSMMSEGLSEEEVRAIEADLGVDLMDDEAPPSGNCGMM |
| Phyto-ph-<br>thora para-<br>sitica | Oomycota | NCBI\|<br>ETO76261 | MPTELSPIKSKRMEEKASLQKLNSRLEMYVLGVNELEGAKHAAERELETIRQR-<br>MQLELESVRSRLTKELEETRKKLNDEIDQNAKLQTLEQEQHKELVKLRAQQTEL-<br>GEAKVLVETLRVQLDREKANAVSAKETLSEQTTQLNLARRHVKELERELRGHSAAL-<br>GDATKELEELRKKSVDFDLTRDAELTKLRREWTAKHLEAQAQWKKDAE-<br>DRLQSMEMEVRSHFESVSGSLQTQLDDVRTELESTKTELDRTANDYEES-<br>LKARQSLTEKVAQLEREYREVRSKATKDRKAYEETLERFRSSKVAKEREF-<br>NELMDVKIALDAEIMKYRRILDREESRVAVATPNTKGRKRKSESSNGTSSKR-<br>AKRAIIETETFSSSASAVQIASMNLEKDRIVIKNSSSDPVPLGGWVVRAQM-<br>DQIFRFPATYVMRPQSTLTVHSSKRNKNAKNERKKGEDSFLANKFTLNPK-<br>GDFVVLVNSDDIPVSMMSEGLPAEEVRAIEAELRADFMDDEVPPSGNCGMM |
| Phyto-ph-<br>thora cin-<br>namomi | Oomycota | jgi\|estExt_-<br>Ge-<br>newise1-<br>Plus.C_460<br>078 | MPTELSPIKSKRMEEKASLQKLNSRLEMYVLGVNELEGAKHAAERELETIKQR-<br>MQQDLDSVRARLTKELEETRKKLDDEMDQNARLQTLEQEQHKELVKLRAQQVEL-<br>GEVKVLVETLRVQLDREKANADSAKETLSEQTTQLNVARRRVKDLEREVRGH-<br>VAALGDATQELQELRKKSADFDLTRDAELSKLRREWTAKHLDAQAQWKK-<br>EAEDRLLSMEMEVRNHFETVSGSLQGQLDDVRAELDSTKKELDRTANDY-<br>EESLKARQSLTEKVAQLEREYREVRSKSTKDRKAYEETLERFRSSKVAKE-<br>REFNELMDVKIALDAEIMKYRRILDREESRVAVSTPNTKGRKRKSENTNG-<br>SSAKRPKRALAAESVVSSSSSAVQIAALDLAKDRIVLKNSSSDKVPLGG-<br>WEVRGQMDQTFRFPPTYLMRPQSTLTVHSNKRNKNAKHERKKGEDSFL-<br>ANKFSLNPKGDCVILITSDGIPVSMKSEGLPDDEVRAIEAEFRADFMDDEAPPSG-<br>NCGMM |
| Phyto-ph-<br>thora infes-<br>tans | Oomycota | NCBI\|<br>AATU01004<br>774 | MPTELSPIKSKRMEEKASLQKLNSRLEMYVLGVNELEGAKHAAERELETIKQR-<br>MQQDLESVRSRLTKELEETRKKLNDEIDQNAKLQTLEQEQHKELVKLRAQQTEL-<br>GEAKVLVETLRVQLDREKANATSAKETLSEQTTQLNLARRRVKELERDLR-<br>SHAALLSDATKELEELRRKSVDFDLTRDAEMTKLRREWTAKHLDAQAQWK-<br>KDAEDRLQSMEMEVRSHFDSVSGSLQTQLDDVRAELDSSKMELDRTAND-<br>YEESLKARQSLTEKVAQLEREYREVRSKSTKDRKAYEETLERFRSSKVAKE-<br>REFNELMDVKIALDAEIMKYRRILDREESRVAVATPNTKGRKRKSDTTNGT-<br>SSKRVKRSIIQTESTSSSASAVQIAAINLDKDRLVIKNSSSDPVPLGGWVVR-<br>GQMDQTFRFPATYVMRPQSTLTVHSSKRNKNAKNERVKGEDSFLANKFS-<br>LNPKGDFVVLWTADDIPVSMKSQGLAAEEVRAIEAEYRADFLDDDVPPSGNCGMM |
| Phyto-ph-<br>thora sojae | Oomycota | NCBI\|<br>AAQY0200<br>0086 | MPTELSPIKSKRMEEKASLQKLNSRLEMYVLGVNELEGAKHAAEKELETIKQR-<br>MQQDLDSVRTRLTKELEETRKKLDDEMDQNARLQTLEQEQHKELVKLRAQQVEL-<br>GEVKVLVETLRVQLDREKANADSAKETLSEQTTQLNSARRRVKELEREMRGH-<br>VAALSDATQELEELRKKSADFDLTRDAELSKLRREWTAKHLEAQAQWKK-<br>EAEDRQRTMEMEIRSHYDTVSGSLQSQLDDVNAELESTKKELDRTANDY-<br>EESLNARQSLTEKVAQLEREYREVRSKSTKDRKAYEETLERFRSSKVAKE-<br>REFNELMDVKIALDAEIMKYRRILDREESRVTVSTPNTKGRKRKSENTNG-<br>TSAKRTKRALVAEPAVSSSSSAVQIAALDLVKDRIVLKNSSSDAIPLGGWI-<br>VRGQMEQTFRFPPTYVMRPQSTLTVHSSKRNKNAKNERKKGEDSFLAN-<br>KFSLNPKGDFVVLLSSEDIPVSMKSAGLPDDEVRAIEADMRAEFMDDEAPPSG-<br>NCGMM |
| Phyto-ph-<br>thora ramo-<br>rum | Oomycota | NCBI\|<br>AOBL01008<br>173 | MPTELSPIKSKRMEEKTSLQKLNSRLEMYVLGVNELEGAKHAAERELETIKQR-<br>MQQDMESVRSRLTKELEETRKKLDDEMDQNARLQTLEQEQHKELVKLRAQQTEL-<br>GEAKVLVETLRIQLDREKANAESAKETLSEQTTQLNSARRRVKELERDMRGH-<br>VAALSDATQELEELRKKSADFDLTRDAEMTKTRREWATKHLEAQALWKK-<br>DAEDRLHTMEMEVRSHFDNVVTSLQNQLDDVRTELDSTKKELDRTANDY-<br>EDSLKARQSLTEKVAQLERDYRVERAKSTKDRKAYEETLERFRSSKVSK-<br>EREFNELMDVKIALDAEIMKYRRILDREESRVALATPKTKGRKRKSENTNG-<br>TSSKRTKRALAVAESVTDLSSSSVQIASLDLEKDRIVLKNSSSEPVPLGG-<br>WVVRGQMDQTFRFPATYVMRPQSTLTVHSSKRNKNAKNERKKGEDSFL-<br>ANKFSLNPDGDFVILVTSDDVPVSMESEGLPEEEVRAIE |

<table>
<tr><td>Phytophthora lateralis</td><td>Oomycota</td><td>NCBI| AOFH01002457</td><td>MPTELSPIKSKRMEEKASLQKLNSRLEMYVLGVNELEGAKHAAERELETIKQR-<br>MQQDVESVRSRLTKELEETRKKLDDEMDQNARLQTLEQEQHKELVKLRAQQTEL-<br>GEVKVLVETLRVQLDREKANADSAKETLSEQTTQLNSARRRVKELERDMRGH-<br>VAALSDATQELEELRKKSADFDLTRDAEMTKTRREWAAKHLEAQALWKK-<br>DAEDRLHTMEMEVRSHFDNVVTSLQTQLDDVRTELDSTKKELDRTANDY-<br>EDSLKARQSLTEKVAQLERDYRVERAKSTKDRKAYEETLERFRSSKVSK-<br>EREFNELMDVKIALDAEIMKYRRILDREESRVALATPKTKGRKRKSENTNG-<br>TSSKRTKRALAVEESVTDLSSSSVQIASLDLEKDRIVLKNTSSEPVPLGG-<br>WVVRGQMDQTFRFPATYVMRPQSTLTVHSSKRNKNAKNERKKGEDSFL-<br>ANKFSLNPNGDFVILVTSDDVPVSMESEGLPEEEVRAIESELRADFMDDDAPPS-<br>GNCGMM</td></tr>
<tr><td>Bremia lactucae</td><td>Oomycota</td><td>NCBI| compound of JP956599 and JP961338</td><td>MTTELSPIKSKRMEEKASLQQLNSRLEMYVLGVNELEGAKNAAERELETIRQR-<br>MQQDLDSVRTRLTKELEETRKYXARGFMLYDVIVAVTYRGLYRKLDDEVDQ-<br>NAXXXXXXXXXXXXXXXXXXDYEESLKARQALTEKVAQLEREYREIRSKATRDRKAY-<br>EETLERFRSSKVAKEHEFNELMDVKIALDAEIMKYRRILDREEHRVAVATPNT-<br>LGHKRKKEATRNVASKRTKRTSTETNVLVSPVQIAAIDLKKDRITLKNTSNDA-<br>IPLGNWVVRGQMDQTFRFPETYVMRPHSTLTVHSSKRNKNAKNERQKGED-<br>SFLANKFSLNVKGDFVVLMTSDDVPVSIKSEGLPEDEVRAIEADLRADFMD-<br>DDAPPSGSCALM</td></tr>
<tr><td>Hyaloperonospora arabidopsidis</td><td>Oomycota</td><td>NCBI| ABWE02006180</td><td>MSAELSPIKSKRMEEKASLQKLNSRLEMYVLGVNELEGAKHAAERELAIIKQR-<br>MQQDLERVRSRLTKELEDTRRKLNNEMDQNARLQTLEQEQHKELVKLRAQQTEW-<br>SEVKVMLETLKAQLIGEKANAKAAKETLSEQTTLLNAAQRRVKELERETRSQS-<br>VALSDATQELEELKKKSADFDLTRDVELTKLRREWTAKQLE-<br>SQAQMKKDAEDRLLTMETEVRSHFESVVSSLQNQLDDVKS-<br>ELESTKQELDRTANDYEESLKARQALTEKVAQLERDYREVR-<br>SKATKDRKAYEETLERFRSSKVSKEREFNELMDVKIALDAEI-<br>MKYRRILDREESRVAGATPNSKGRKRKSENTNGFQSKRAK-<br>RAVVFEREGLSSSASAVEIASLDLEKDRIVLRNTSSKPVPL-<br>GGWVVRGLMDQTFRFPATYVMRPHSTLTVHSSKRNKNAK-<br>DERKKGEDSFLANKFSLNQKGDFIVLVTSDDVPVSMKSEGLSEEEARAIEADLRAD-<br>YTDDEAPPSGNCCMM</td></tr>
<tr><td>Pseudoperonospora cubensis</td><td>Oomycota</td><td>NCBI| AHJF01000192</td><td>MPTELSPIKSKRMEEKASLQKLNSRLEMYVLGVNELEGAKHAAERELETIKQR-<br>MQQDLENMRSRLTKELEETRKKLDDEMDQNARLQTLEQEQHKELVKLRAQQVEL-<br>GEAKVLIESLRVQLDREKANAESAKETLSEQTVQLNSARRRVKVLERDM-<br>RGNAAVLNDATQELEELRKKCADFDLARDAELSKLRREWTAKHLETQA-<br>QWKKDAEDRLQTMEMEVRSHFESVSTSLQSQFDDVKAELESTKKELDR-<br>TASDYEESLKARQSLTEKVAQLERDYREVRTKATKDRKAYEETLERFRSS-<br>KVSKEREFNELMDVKIALDAEIMKYRRILDREESRVAVATPNTKSRKRKSE-<br>STNGTSSKRAKRAVFETEKFSSPVSAVRIDSLNLEKDRIVLKNTSSEPVPL-<br>GGWIVHGQMDQIFRFPATYVMRPQSTLTVHSSKRNKNAKNERKKGEDSF-<br>LANKFSLNPKGDFVVLLTSDDIPVSMKSEGLPEEEVRAIEADLRADFMDDEVPPSG-<br>NCSMM</td></tr>
<tr><td>Pythium ultimum</td><td>Oomycota</td><td>NCBI| ADOS01000666</td><td>MPTALSPIKSKRLDEKSTLQFLNGRLELYVLRVKEMEDAKNVAERELETIRQRMQA-<br>DIDALRLRMSKELDETRKKLDVELDQKARLQVLEQEQHVELVKLRAQVKEL-<br>SEIKALAESLQVELTKERSNSKASKETLSVQTTQLQSARRRIKDLERENRSYEASLS-<br>DTTKELDELRKKSSDFDLTRDLEITSIRKEMNQKHQEALAQWKKDTEERVLGVEK-<br>EVRLYFEGVVQGFKSQVEDLSVELDSTKKELDRTASDYEESLQVRQQLTEKVAHL-<br>EREYREERTKFKEDRKKYESTLEHFRSSKFAKEQEFNDLMDVKIALDAEITAYRRI-<br>LDREETRFGLPTPTNTPNAKQTARKRKSETSGASAAAAASKRIKRAPSSVQIVHL-<br>DLEKDRIVLENSSDKPVSLAGWEVRGRAENQVFRFPATYSMRPKSKMTVYSSKR-<br>NKNAKDERKPNEDSFLATKFSLNASGDFAVLLTADGVPVSTRAEGLSAEEVNTLEAE-<br>IKADLDDEAPSTEGCGIM</td></tr>
<tr><td>Pythium arrhenomanes</td><td>Oomycota</td><td>NCBI| AKXY02009383</td><td>MPTALSPIKSKRLDEKTSLQTLNSRLELYVMRVKEMEDAKNVGERELETIRERMQK-<br>DIDNVRSRLSHELEETRKKLDVELDQKARLQVLEQEQHVELVKLRAQVKEL-<br>SEIKALAESLKAELSKEKANAQLSKEELSTLTTQLQAARRRIKDLERENRAHEASLN-<br>DATQELEHLRKKSADFDLTRDAEISNIRKEMSLKHTEALAQ-<br>WKKDTEDRLKGVEKEVRNYFESVVSGLKNQVEDLSSELDS-<br>TKKELDRTANDYEESLKVRQSLTDKVAQLEREYREDRAKF-<br>KEDRKVYETNLEKLRSWKVTKEGEFNDLMDVKIALDAEIETY-<br>RRILDREETRVGLPTPHTTPNKGSRKRKSDSAASAAKRAKRT-<br>TAPTAIHIAHLDLEKDRIVVENTSDQAVSLGGWEVRGRAEN-<br>QVFRFPSTYSMRPKSKMTVYSSKRNKNAKDERKPNEDSFL-<br>ATKFSLNQSGDFAVLLTNEGVPVSMKGEGLPADEIKKLEQEVFAELDDDAPAAE-<br>GCGLM</td></tr>
<tr><td>Pythium irregulare</td><td>Oomycota</td><td>NCBI| AKXZ02005003</td><td>MPTALSPIKSKRLDEKSTLQLLNGRLELYVMRVKEMEDAKNVAERELETIRQRMQA-<br>DIDAIRLRLSRELEETRKKLDIELDQKARLQVLEQEQHVELVKLRSQVKEL-<br>SEIKALAESLRVELTKEQANSKSSKETLSVQTTQLQSARRRIKDLERDNR-<br>SHEASLNDVTKELEDLRKKSADFDLTRDSEITTIRREMNQKHQEALAQWK-<br>KDTEERLQAVEKEVRHYFEGVVQGFKNQVEDLSLELDSTKSELDRTAND-<br>YEESLKVRQSLTEKVAHLEREYREERTKFKEDRKKYESTLEHFRSSKFAK-<br>EQEFNDLMDVKIALDAEITTYRRILDREETRVGLPTPTSTPNAKQTPRKRK-<br>SESIVNGAAKRVKRAPSSVQIVHLDLEKDRIVLENTSDKAVSLAGWEVRG-<br>RSENQVFRFPSTYSMRPKSKMTVYSSKRNKNAKEERKPNEDSFLATKFS-<br>LNAGGDFAVLLTAEGVPVSTRAEGLSAEEVKALEAEIKADLDDDAPPGEGCGIM</td></tr>
<tr><td>Pythium iwayamai</td><td>Oomycota</td><td>NCBI| AKYA02003977</td><td>MPTALSPIKSKRLDEKSTLQLLNGRLELYVMRVKEMEDAKNVAERELETIRQRMQA-<br>DIDAIRLRLSKELDETRKKLDIELDQKARLQVLEQEQHVELVKLRSQVKEL-<br>SEIKALAESLQIELTKERSNSKSSKETLSVQTTQLQSARRRIKDLERDNR-<br>SHEASLNDVTKELEDLRKKSADFDLTRDSEITTIRKEMNQKHQEALAQWK-<br>KDTEERLQAVEKEVRHYFEGVVAGYKNQVEDLGQELDSTKKELDRTAND-<br>YEESLQVRQKLTEKVALLEVEYREERNKFKEDRKRYESTMESFRSSKFA-<br>KEQEFNDLMDVKIALDAEITTYRRILDREEVRVGLPTPTSTPNAKQTPRK-<br>RKGDSSAGTAAKRAKKAPNTVHIVHLDLEKDRIELENTSDKPVSLAGWE-<br>VRGRSENQVFRFPSTYSMGPKSKMTVYSSKRNKNAKEERKPNEDSFLA-<br>TKFSLNASGDFAVLLTADGVPVSTRAEGLSAEEVKALEAEIKADMDDDAPPGEG-<br>CGIM</td></tr>
<tr><td>Pythium vexans</td><td>Oomycota</td><td>NCBI| AKYC02003929</td><td>MPTALSPIKSKRMDEKTSLQTLNSRLELYVLRVKEMEDAKNVAERELETIRQRMQL-<br>DIDNIRTRLTKELEETRKNLSDELEQNARLQVLEQEQHAELVKLRAQMKEL-<br>SEVKALAETLRLELTREQSNAKASKETLSEQTTQLQLARRRLKEL-<br>ERENRSQSAHLNDTSMELEELRKKSASFDLTRDGEIANIRKEMNAKHQE-<br>ALAQWKKETEERLLGVESETRSYFDNVVTGYKSQVEDLSSELESTKAEL-<br>DRTATDYEESLKVRQQLTEKVAQLEREYREDRTKFKEDRKQFEITIERAR-<br>SSKFAKEQEFNELMDVKIALDAEITAYRRILDREESRVGLPTPNTKGRKRK-<br>NESSASSASKRVKRTPPSSSVQIVRLDLEKDSVYIENTGDKPVALGGWE-<br>VRGQLEGQLFRIPSTYTIRPKTTCVVISSMRNKNAKKERKKGEDSFLATK-<br>FSLNPKGDLAVLTTPDGVPVSLCSEGMSAADVKKFQDEIRNDFEDDQPPSEGCGIM</td></tr>
</table>

| | | | |
|---|---|---|---|
| Albugo laibachii | Oomycota | http://protists.ensembl.org\| FR824125 | MPSSQFSPIKSKRLDEKASLQSLNSRLEIYVMRVKEMEDAKTVAERELETIRDR-<br>MQQDMDVVRLRLTKELDDTRKKLDHELDQKTRLQILEQEQHVELVKLRAQVKEL-<br>GDLKTLTEQLKSELSKERSNATAAKEELSRQTTELQAVRRRIKDLERERRGM-<br>DAALSDATTELEHLRKKSATFDLVRDTELSNIRKEMNAKHQEALA-<br>NWKKDTEDRIHAVEKEVRNYFEGVVTTSKSQAEELSLELDSTKKE-<br>LDRTANDYEESLQVRQSLADKVAQLERDYREERKKFKDDRKMYE-<br>TMVEKLRYAKLSKEEEFNDLMDVKIALDAEITAYRRILDREETRVG-<br>LPTPKTTPMEKGKKRKSLAFSGNRKRVKQENDDPIRIAQLNLEK-<br>DFVVFENQGDKPVSLGNWEVRGKLETQVFRFPSNYVVKPHARV-<br>TVFSAKRNKNARADVKPDEDAFMTKKFSWNHSGDWAVLYDDEG-<br>VPVSSLAQGLPKEEVEALEAAIRTESPEDDFKEGSSDACWIM |
| Albugo candida | Oomycota | NCBI\| CAIW01001050 | MSSSQFSPIKSKRLDEKASLQSLNSRLELYVLRVKEMEDAKTVAERELETIRDR-<br>MQQDMDVVRMRLTKEVEDTRKKLDHELDQKTRLQILEQEQHVELVKLRAQVKEL-<br>GDLKTLTEQLKSELSKERSNATAAKEELSRQTTDLQAVRRRIKDLERERRGLD-<br>SALSDATTELEHLRKKSATFDLVRDTEISSIRKEMNAKHQESLANW-<br>KKDTEERIHAVEKEVRNYFEGVVQSFKSQVEELSLELDSTKKELD-<br>RTANDYEESLQVRQSLADKVAQLEREYREERKKYKDDRKVYETT-<br>VEKLRYAKQSKEEEFNDLMDVKIALDAEITAYRRILDREETRVGLP-<br>TPKTTPMEKGKKRKSAAMKGNRKRVKQENDGPIRIAQLNLERDF-<br>VVFENQSEKPVALGNWEVRGKLETQVFRFPSTYVIKPHARVTVF-<br>SAKRNKNARADVKPDEDAFMTKKFSWNHSGDWAVLYDSDGSPV-<br>SSLAQGLPKEEVEALEAAIRSESPEDDFEEGSSDACWIM |
| Saprolegnia diclina | Oomycota | NCBI\| EQC27961 | MSSSSLASKRESFSPMKSKRLDEKTTLQALNNRLEMYVLRVKEVEDSKEIAEKELE-<br>SIRDRMQLDLTMTKTRLSKELEEVRKKYEEEREAKIRVQNLEKEQYNELVQLRE-<br>QVKELNNTKTYLETVQSDLAKEKESCKAAKEALASNATALQSERRKLSDMEKEYRK-<br>TLASLHDATSELEQLKQKTSEFSMTRDSEMTALRKEMNAKHSEALAAWRRESEER-<br>QQVVENEVRSHFEGVVEGLRSQLEEATKETTALRTDYERTANDYDESLKLRQSLTE-<br>KLAVVESQYRADRKKFQEDRRVYESNIEAARKARKEKDLEFNDLMDIKIALDAEISA-<br>YRSILDREETRFGVEPKASTQKKRKSTPQSSRAQKRRKTHASGDFRIRQLNLDEGR-<br>IILENTGATPLPLNGWQITSKSSNHVFSFPDDYVIQPGGKVSVLSGKNPTVPEGEQD-<br>LMDFYAVKKAIWNPKGDVALVKNAEGEVVCSHAEGLNEDEYDDEQEKEGNNEG-<br>CGIM |
| Saprolegnia parasitica | Oomycota | NCBI\| ADCG01001748 | MSSSSLASKRESFSPMKSKRLDEKTTLQALNNRLEMYVLRVKEVEDSKEIAEKELE-<br>SIRDRMQLDLTMTKTRLSKELEEVRKKYEEEREAKIRVQNLEKEQYNELVQLRE-<br>QVKELNNTKTYLETVQNELTKEKESCKAAKEALASNATALQSERRKLNDMEKEYRK-<br>TLASLHDATSELEQLKQKTSEFSMTRDSEMTALRKEMNAKHSEALASWRRESEER-<br>QQVVENEVRSHFEGVVEGLRSQLDEATKEITDLRTDNERTANDYDESLKLRQSLTE-<br>KLAVVESQYRADRKKFQEDRRAYEANIEAARKARKEKDLEFNDLMDIKIALDAEISA-<br>YRSILDREETRFGVEPKATTQKKRKSTPHSVRAQKRRKTHASGDFRIRQLNLDEGRI-<br>ILENTGATPLPLNGWQITSKSSNHVFSFPDDYVIQPGGKVSVLSGKNPTVPEGEQD-<br>QMDFYAVKKAIWNPKGDVALVKNAEGEVVCSHAEGINEDEYDDEQEKEGNGEG-<br>CGIM |
| Eurychasma dicksonii | Oomycota | NCBI\| compound of gb\| FR844597,FR840649,FR844317,FR844598,FR840650 | IREPEDETKPTCNHRVALEKYTALEKNFADHKKRWKEEKAALEHTLDVN-<br>RNARVEKEKQYNTLLDQKLTLDAEINXXXLRLMQRSINGSILEREESRVGLPTP-<br>KANKRKVARTPMTDKRAKRSNEPVIIHDLDFEKDFIQLRNTSDSAISFS-<br>GWTVLSVEAPDGPTHKFSFPENYSLKANTVVTVLGGKKNKQKGDPDKDKFWTNK-<br>YMWKDSNIAEVRDAKGELVVYKVYGDISDPYTEETDGAIPGETACCVM |
| Aphanomyces euteiches | Oomycota | http://www.polebio.lrsv.ups-tlse.fr/aphano/\| Ae_2AL7951 | NELEQLKQKTSEFSLTRDTEITAVRKEMNAKHLEALAAWRRETEERMHNIEAEVR-<br>SHFEGQIEGLRTQVEETNAELDQLKVEYERTANDYDESLKIRQSLTDKL-<br>STLETQYRNERKKFQEDRKTYETNIDAARQARLAKENEFNDLMDIKIALDAEIAAYR-<br>SILDREESRVGLDSAKHKKRKLSTTPSATRQLKRRKSHSTGDLRITYLNLEQ-<br>GRITLENTGSTPLPLTGWKVTSKTSKHVFTFPDDYVIQPGGQVSVVSGRNA-<br>TPSEEEKEAMDFYVIKKAMWNPQADIALLTNPQDEVV |
| Aphanomyces invadans | Oomycota | NCBI\| ETW04496 | MASKRESFSPMKSKRLDEKTTLQELNNRLEMYVLRVKEVQDSRDVAEKELETIR-<br>ERMQMDLSMTKTRLSKELEDTRKLLEFEIDQKTRLQVLEQEQHTELVKLRTQVKE-<br>FGDIRVELEQVQAELAKEKESSKAAKEALALQTTSLQSARRKLKDLDKEN-<br>RKLTSSLSDTTNELDQLKQKTSEFSLTRDTEITLVRKEMNAKHLEALAAWRRESEER-<br>LHSVEAEVRAHFEGQIEQLRSQVDEVNLELDSLKVEYERTANDYDESLKIRQSLTD-<br>KLSTIETQYRNERKKFQEDRKMYEANIDNARQARLAKETEFNDLMDIKIALDAEISA-<br>YRSILDREESRVGIDQANHSKKRKASLTPVKSSTTRQHKRRKSHSTGVLRITYLNL-<br>EQGRITLENTGSDALSLSGWQVSSKATNVVFAFPEDYVIQPNGRVSVISGRNAAPT-<br>EEEKESMDFYVIKKAMWNTQADVAQLTNPSGDVVSSYAEGMYVDD-<br>DDVDAADTPAKDGCGIM |
| Aphanomyces astaci | Oomycota | NCBI\| XP_009842573 | MASKRESFSPMKSKRLDEKTTLQELNNRLEMYVLRVKEVQDSRDVAEKELDTIR-<br>DRMQMDLSMTKTRLSKELEDTRKLLEFEIDQKTRLQVLEQEQHTELVKLRTQVKE-<br>FGDIRVELDAVQGELAKEKESSKAAKEALALQTTSLQSARRKLKDLDKENRK-<br>LASSLSDTTNELEQLKQKTSEFSLTRDTEITLVRKEMNAKHLEALAAWRRESEDRL-<br>HNIEAEVRSHFEGQIEGLRSQVEEANLELDSLKIEYERTANDYDESLKIRQSLTDKLS-<br>TIETQYRNERKKFQEDRKTYEINIDNARQARLAKETEFNDLMDIKIALDAEISAYRSIL-<br>DREETRVGIDHANHSKKRKASLTPVKSTSSRQHKRRKSHSTGAVRITYLNLEQGRI-<br>TLENTGTVALPLAGWQVTSKATNVVFAFPEDYVIQPSGHVSVISGRNAAPTEEEKE-<br>SMDFYVIKKAMWNSHADVAQLTNPAGDVVSSYAEGMTVDD-<br>DDVDAADTPVKEGCGIM |
| Cafeteria roebergensis | Bicosoecida | MMETSP0942\| CAMNT_0005518387 | MASPGFAGSRRRVEEMRVLGSLNNRLEQHVRQMRTLAKERDALLERVA-<br>GLEVDHSTTMRDRAAAHQEEVSRLNRQIRSLTGKADKAETAVRRLEQVVK-<br>TLQGQVDDIDVVRQQRDSLAANLRNTRSDLANMEELLRDAEG-<br>DRDQYMEGLEAAKAGSKDLELQLAAETAAHDRTREDLDAAQTALRRLRDQADA-<br>ASEASDDEEDTIRALRDELVELRARKVELEDAEAELRAELEGLRGGSAGQIAAL-<br>KAELERVRAELAEALSQQEQLTERANDAEKAAAAAEARAAAEASERAVEAAAA-<br>REAAAAARAAAEAEVSDMRRRLSAALAQAADASAAPGSAGGSSMGERVRELE-<br>EERSVLAAELKQYERILDTDMAFLGINTPQRKGxxxGASHLQLWLDLHQEVVIVV-<br>NTGSDTVAMDGWHVLSEQGQRFDLPEGQSIKAGSCIAVWSGPDGAVNAATHKA-<br>SLLWTERPVWNNKGDVARLVDSSGDIAAERAVSDKAAPLNLASLRR |
| Ochromonas sp. Strain CCMP1899 | Ochrophyta | MMETSP1177\| CAMNT_0007030967 | MEEQNTIDDLRRDLESYVNSVAVIESENKQFKEIIAQLEKERELEAER-<br>WLSVLNKGKESWREQAIQHGRHQKKQTMIECEKVMQSFQSEL-<br>RTQYFQYQTRDSSVLLSCLLRANSSTERAEHDRGVFESMALF-<br>GAIKPTVTVQDFDIEDNHVSISNRTDMEVSLTGCSLKFKGSGAKYKFPDDLIIHPTST-<br>ISLWYGKEANSSIRQAQNRGSLYWHSNSAITTDFAESVGLVCKKGTTFCSVLANKE-<br>RQARHATGQDPTDNSVVHPSHFLKFNQFFSLKNDDEPLVGQQKDLNDDDKYPNP-<br>STESKHSGNKSSTSSNKSSSSSSARKRHYSVIASPQPQTTDAITSEDKGSIITSQSS-<br>GLSVSSALSSHFTSPSVLNYRPLHLRPYLPLRSQGCLHFNKVLRKQGEGIKGAVAV-<br>TVSNSEEGPVALEGWRLIAVGNPSHEFFLPSDAVIPGRSSITLLSSTCDDTLLWAQIK-<br>LSDINPTPDMDISNGSKDVKMPRSEKKNGKKAKISSILSIRDPEGVSDRQIAVVAGL-<br>SALSKGDILCLHLIDQLGRNICMVQGTEVDDTHTKDTTVFGDNANDNNKISYLSSL-<br>TNIAKNSKDSCSIQ |

| | | | |
|---|---|---|---|
| Nanno-chloropsis gaditana | Ochrophyta | NCBI\| EWM28057 | MVRTRKSDAAPGTPAIGKDKQNKSRGDDMYKQVEEYVMRVKDLEITNKQLREEL-<br>RRSGGDDTVGKGIRCSTRMSSPNCVKKMRDLRQHIQTLESKIKEARRENQQLRE-<br>QFNESFEDAREKFRSQGRIHGNLMAAKKADEFSGQILYLKDSVHRLEKTVKE-<br>LEAERRRLQQEVKEVDELRNDLKRAEERHAMLERNVENLQDENENLERELEHQ-<br>EGRHSLGVVVEEEKPGVPELEAEIRTLKEQVEGLTEDLRTRMSAAEETESLKDEV-<br>EELRASLKAATSADRERREARDSIVTQLEVCREQARKEKDRCQELQKRAEALVE-<br>AQKAAEEKARRLEDSPSRTIVKARATALESTVKSLEGELSLLRRERNKNLGDVDA-<br>LQEDKEALEDEVRRLALQIEQQRRQAEAVLARETESLERKIDRLQEELDTANAKS-<br>VAAVEASASAVARALQEVGPASAARRQGRGTSLGGTEEGSEQRLLVEENARLRR-<br>EVDVYRELAGSQHDQISLVYIQEVNAQADYLVVENRTNLDVSLAGWALGTSLSES-<br>EIQFEFPEELMLSSRASLKVWWGARNISYKSRPTRGNLFWEESDRIDIFQAQNDE-<br>VLLMDAEGCEMSRMRIVSDKRRARAGPFVGTPTPDSKRFRSSPWSGGGSGMR-<br>DNFGVRTSILDEDQGESDMFMMAVEGSFNEEEG |
| Corallo-myxa ten-era | Rhizaria | NCBI\| GAB-V01000374 | MDDAPKTPARVSASLFEHALASPARQTRASEKDEMQELNRRLEFYINKQRERE-<br>ARRSTEERELMTAKQVVSREIDRARAEYQSQLAEVRKQRDQHVASKTHLQTTIAR-<br>LQQNVYDLEGRLKQEQAEHKELESRRDQLSNQLKDVNKELQ-<br>NAKELLRSTEGDLEDQQKQNDALEGEIDRLRKEYEDAVKNASEAEARRISIEEDLAI-<br>ERTKAEKASQRFDEEMKQLSNIDKDVENQLRQEFTNQLNSILAERQNQYEAEKNA-<br>LANELRTLYDEKVSAYRAQVEKSSQEKAQLEAECKRFDLETQAAQAEVNKAIADRN-<br>SLIAQIQALQQELSQARDGPEREIEHKKRIIRRMKEAYQRKEAEFDALMDVKIA-<br>LSMEIKRYRQMLDEEEDRLGI |
| Reticulo-myxa filosa | Rhizaria | NCBI\| ETO28743 | MFNKTKYKKLKKQQHRLEEKRELRDLNQRLTMLVRHQREGRSEVNHLKKL-<br>LAETEVDFRQKLKDSEKHYQLLQDKFMAENEQLNLHNKTLREQLDTTEKLNTFF-<br>PQYFTFVNFVFLFNPKEPVKMLRADMTELELKDKKLQAMQNQLEGLMNEL-<br>VAVKASVKASSKNDEQLAGQLQEQCDQWKSKFDMTCEELAKIKSRHKCQVDG-<br>YQRELNGKSEENEKLKEQLREYVKKGQELQNQIRQEFDTKMTEVLNDLLLYQN-<br>NLFFFFFVEKREIQYQSEKEEWMKIFKEEYQKKMTQFKEINAQLVESNKKLESEI-<br>NDLKLRLTRVRREKAEIDAEKHHLEEELEKARNDTDELRQRKDDELREKTLLVS-<br>SLQESLKQKETAFEELSGAKIQLDNEIAVENEGWRDLLCAFVCVLFFIQCPPPSF-<br>FKKTEKEAGYVNPLESGRKKRNTDDGNNCSVSTPGLNRARQAARQELQDVGT-<br>STDKNSPNNVRDNEIMEEDEEENGSEEDESEDQENIFDTNNLPRGNITTNPLG-<br>SNNHNHKRKYPDQKDVDKTMSSNSESKHNDIDSVWQIEKR |
| Reticulo-myxa filosa | Rhizaria | NCBI\| ETO24379 | MSESQNDCDPVSSQYFQDIGDLETSDAQQHVTEKKELSNLNHRLEMFVRHQRD-<br>SRNEISRLRKALVDTEVDLRQRLKDAEKRHQIIQDKIMAENEQLSIQNKTLQDQLET-<br>TEKAYQSSESRRASLDEKVTCKMTEIELKDQQLEMLQNQLEALT-<br>NELITVKSSVKLMVKNDEHTAEHLQAQCDQWKNKFDVTCEELAKVTYAF-<br>FSFLLQTLTPNIDHCFFFRNEDFGKFSTKKKKLFLNKCLNCNIRSFSDKL-<br>VENEKLKEQLNESTTKFQDMEDQLRKEFDVKMLEVWSGFIVFLFWSHP-<br>FVHVRTYKKFTQKRESQYQNEKEEWIKMFKEEFQKKLAHFKEENAQYL-<br>ESNKKLECEIVDTKARLARVRREKLELEAEKRSLVHFLLKDVVETKGKNIY-<br>IRIFFQKKEEEVEKSHSDVNELRQKKDEEIHEKTESLRTLNDTIRQKDIQYQQLSS-<br>AKVQLDNEIA |
| Reticulo-myxa filosa | Rhizaria | NCBI\| ETO09514 | IANLQNSIKQKDCEYQKLSAAKVQLDNEIALYRNILSNAEKETDHFNPLGSALKKRK-<br>TSDGKKHIIDTPDAAYSRQTAGNNVQHIEDKKSSKTKYNKVESENINKSGNKGKD-<br>NGNKRKHLEFYEAKIGETKQRANDTNEHNETDSYVSFAYLSLALSFCSPPPP-<br>KNKLYFFVQKTPGQLCPLQFSAMDLSSSTLEVQNTSEEPLNLNGFYLTNTDTSKQF-<br>HLPSNRIAHIGFVFADERIKIIVGDTPGLKVKTGDLRWHDNVWSGLKDELVRLYNPQ-<br>HEEIARIEIAPEMLPKPERTRENCLIIKKLSNLKMFFSTIFSSFVSAHQIS-<br>HFELAFLHVFLTKLNQ |
| Reticulo-myxa filosa | Rhizaria | NCBI\| ETO02958 | EEELEKSHNDLDELRQRKDEEIREKTLLIMSLQEQIRQKDIQFDALSGAKIQLDNEIA-<br>LYRSIWSEAEKEAGYSCPLGSGSKKRKTDNGDTQSMDTPGSMRARQLARQDLE-<br>HVGTDDQNGASESEQGDGNEEEEEDKEKEKDEENDDEAKDAKDDEEND-<br>DENNKNRGN |
| Reticulo-myxa filosa | Rhizaria | NCBI\| ETO17410 | LYRDILNEAEKDAECFFQTNSGCKKRVSINDKPRTAVTTPGLTRARQAAKNDL-<br>SRVVNSNSKCATPKNTEKKNETNVICKNENVYEESSNLGENDENQNRNNFNQNS-<br>GLHKSSSNKSSSVSNSSHKRKSFEFDKTNSKKINETDYSPTRVKLSTFFQVCDPF-<br>PLQFSGMDLCSSTLELQNVSEEPVDLKGFYLTNSNKSKIFILPHHHVLM-<br>PKPKFNADPFFVTTLDKKIKIIVGNDGDLNVGKDDLWWQENVWNGLEDE-<br>IARLFNSKNEEVATVNITTEMIPEKIRARNKCLFM |
| Reticulo-myxa filosa | Rhizaria | NCBI\| ETO27405 | MKRNNVECPFNRDDEIDPSIQQQMDDKHKIRDLNTRLKMFVRHQQEGRSEVAR-<br>LKQLLEETEFNFRQRIKDSEKRFQLLQDKMQAENEQLTVQNNSLREQFENAEKYYII-<br>IFVVVPNRARQNAESRCANLDQTVARKSTELEVKDQKLQALQKHLDSLTNDLI-<br>AVKASAKASLKNDEQLTGQLQEQCDQWKAKFESNCEDLAKVLDCFIKARHKTQ-<br>VEAYQHSLSDKSEEIEKLKEKLKECWKKSQDMENQMRKEVDIKMSE |
| Reticulo-myxa filosa | Rhizaria | NCBI\| ET-N99247 | TPGEPNPLQFSTMDLCSSTLEVQNVSDHAIILKGFYLTNLDKSKKFLLPPGRVLKPS-<br>FVIFYFYFCFMFFFFFIIVSCMIMAVDVDVQLNYAGERVKIIVGNDSNLRVAEGDL-<br>WWNESVWSGTKDEIARLYDSSNQEISRVEISPEMLHEKVRSCKNCLIIMCLIFFFV |
| Ammonia sp. 2 | Rhizaria | MMETSP 1384\| CAMPEP_0 197081440 | MSDEPGSQSSLPIAKQALAQKRNVRDLNTRLEMFVRAQAEKTRAINQLKESLAR-<br>QEMDYKSKLTQQRVQFQDNVEKLRKENQNLAYENKCTQQEFTNALTSKAEFEERL-<br>LKSEEKNAALSSENSNIRTDLSRTQQEFDDMRKAFATMKYKYDSYEAERCSFESR-<br>MAKQKSKTEQLLKELTQKEATLKTEKATFERLIVEKNEEIEHFTEAVKKLELEN-<br>STTQSRLRQEFDNKLAEFVQKREEQYKQEKDEWMRIFKEEFNRKLRSFKEA-<br>NQELSHSNIKQSEEIQDLRARISKLKQQKTELEVTNRNNEEEIEKLRNDLDDL-<br>RRTKDAEIKLKNSLIIQERDRFKAKELQFDELAGMKLQLDAEIELYRNILNEAE-<br>RDCGYNSPMVMKTNTGTRNSRKRKRMNNYNMTPIFRKIITPGVSRAAKIAQ-<br>KDLKAFESDGDHEDQDMKEQESESFDTYETPSGIEGGALQFSGLDLNKGMI-<br>EIQNMGEEPVSLDGFALTNQSGSMIYDLPTDMELQAKNTLRIYVGEALFKDM-<br>CGSSESEKDQQRRSRKFIGDYDGAYVFWGNDVWTGTDTDCARLYNPSQ-<br>EEIARIEISPDMIDGVDKKNNCLMM |
| Ammonia sp. 1 | Rhizaria | MMETSP 1384\| CAMPEP_0 197029186 | MSKPEPGSVRDRDVPPAKQALTQKRTVRDLNTRLEMYVRAQAEKTRNIN-<br>QLKEALARNEMDYRNKLKQQKIHFQEKVEKQRKQIENLEYDKKCTQQELENAM-<br>SAKTEYEERLISSEGRNAALASECEQLKTDIGRLSKELEGLRK-<br>TYASLKYKYDSYEIERNSFGEKMQKLKSKNEQLLRELTANEQHLKSEKETFQRVIIE-<br>KNDELDNLKKQLKELELTHNSTQTRLRQEFDQKLAEFVTNREQQYETEKAEWMRI-<br>FKEEFNRKLRSFKEANQELSHANVKQSEEITDLRTRMSKLKQQKTELEVLNRNIEE-<br>DAEKLRTDLDNLRRQKDEEIKQKNAVILQQRDAYKAKELQFDELASIKMQLDSEIEL-<br>YRNILNDAEQECGYTSPLATHGGDKGTRNSRKRKRYNAMIHGTPMGPTPFAENSN-<br>NREDGMNTSNSSSNSNNSAALKVETPGITRAAKRAKKEVKQVLQSDGSAVQQSE-<br>DEMEEEEQVNEEEMKDKEEDDSEFDEYSTPGNAEGARLQFSGLDLLKGMIEIQN-<br>MCEQPISLRGYTLSNDTGTSQFPLPNDVVLDHNQKIRIYCGKQLYQEMCQFDDED-<br>AAPQIDKKKQEIIGDFNGIYVSWGQDVWTGQSNDCARLYNPNHHEIARIEISPDM-<br>VDMNASSKSGCFVM |

<table>
<tr><td>Elphidium margarita-ceum</td><td>Rhizaria</td><td>MMETSP<br>1385|<br>CAMPEP_0<br>202725738</td><td>VRDLNTRLEMFVRAQCEKSREVNTLKEALARSEMEYRNKMKQQKLQYQDQIEK-<br>SRKAIENLEYDNKCTQQELDNGIASKKELEDRLMSSESRNASMSSENDTLSTELQR-<br>LKTELDTVRTAYAALKYKYESYELERNSFDDKVNALKSKNDKLLRELTQTTSTHK-<br>SEKETFSRVLVEKNDEIETLRTENKQLKLANTTTQTRLRKEFDSKLAEFVQKREE-<br>QYKQEKDEWMRIFKEEFNRKLRSFKEANQELKLANCKQLEENTELRERISKLKQ-<br>LKMELEVQNRNNEEETEKLRTDLDNLRRQKDEELKQKNAIIIQQRDAYKAKELQF-<br>DELAGIKMQLDSEIELYRSILNEAEQACGYVSPFNADKHGCTSGGPSRKRKRVH-<br>MTPTGGLHSHATHMTLGGHPFGGQHHNSHSNTGNGSSSNTTTTAKQTTDGDG-<br>GKKVETPGIGRAAKQAHTDIKQNSNTGNGSSSSNTTTTAKQTTDGDGGKKVETP-<br>GIGRAAKQAHTDIKQIKQALESDTDGDANEAEMKDAEEDEDEEDCQSVSGSLGT-<br>PGDLEGSALQFSGLDLVKGMIEIQNVGEHAMPLKAHALSNKEGTQQFPLPSHITL-<br>DSHQKLRIYVGERYKAVSSAEDDAQQEEERKRIVGSFDGHYVFWTKDVWTGDS-<br>KDCARLYNAQQQQIQHIEISPDMVDVHGSKKNGCFIM</td></tr>
<tr><td>Cercozoa sp. strain D1<br>2</td><td>Rhizaria</td><td>MMETSP|<br>CAMN-<br>T_0037965<br>289</td><td>DSLNLGQEIIRARADAGSANLKLEIANKNIARLQDQLVEYQAADT-<br>DTKALLATERQKRKSLQAELNLLNARLKEQVLSPSTQKSDAAGPLMYTQQVFDKR-<br>LQSVIADQCSAAERNRQIAMTEQKLYFQEKVKGYQAQLQVAGCEIEQQKQRCRE-<br>LENRLEAAAEDYNRREAEAMAFANALEVRIFGLEEAIKKEKSKPSKIIAEKN-<br>EIIRKVQEVLKEREEEYDELMETKINLQVELQNYQSLLQQEESRCGWENA-<br>TNNEEHEEQGNELQKVVGHKRKRRGSHVSRRTKVSRGKGVQSRRCVE-<br>DTETQQLEADNKIRCVLSAGSDHRGLFLVLSSSEFLDISEWSFQVENSSG-<br>QRLVYTVPRDFSIEGAGSFRVFISSEPVSVEEKTEAWWNWPESFAVPVDTTSPDD-<br>LD</td></tr>
<tr><td>Cercozoa sp. strain D1<br>1</td><td>Rhizaria</td><td>MMETSP|<br>CAMN-<br>T_0037988<br>625</td><td>MPRTPASRRKGGLETPQPKPPNTPGSTTRKARLEEKSELQDLNKRLEFY-<br>ILKQREKDANAGSIQRTISENKEYYENEIKKLKSLHETQVNNFRKQRDELD-<br>SEIQSLTEENKRQASMLKSYTSQLSADKTRGTDLEDRVSQLTEEL-<br>LEAQAATAVAEADAKKSAHGLKKAESEIKHLQKELKDSQARDKDHAV-<br>ELAAAQSLAKNLQDDINIMSQKASESAAKVEELESALSSTRANAEDK-<br>LRALFGAQLKEILAERQRQFDEKKAEGLQELRDSYEGKLSEANEKCH-<br>TLLEKLEEAHKAAAEAAGASSVVDTDASDAVAKVWENRIATLQNQLA-<br>EQRTQADQIAQQTLREHSKEVAALKAEIEVLEEQANSASSTNSKLAT-<br>SEEENSFLRSERTKLNAKINTQKKELSKAEGQARKIAALQKQAAFLE-<br>EQCESHKQENQTLNEEYISLMDVKVGLDMEISEFRRLLSDEESRLQP-<br>QEEEPAPIEEEEDEEEEQKEVRTTNKKRGRQKASTPAPKAKRARAS-<br>TAKRGKKAAVEEEEEPAKPTLIISGIDSKKWIEITNSVKEAVCLDGWSL-<br>KVVSSKECFFFPDVAPLKPGKTLKINLGSKAKKGKNAVVWAKDDIFKEEDKV-<br>YLMSPEGVGHSSVAWNDE</td></tr>
<tr><td>Plasmo-diophora brassicae</td><td>Rhizaria</td><td>NCBI|<br>CEO95498</td><td>MTISREVEESPIRMQRDVERKNLEELNGRLEQYIMTQRRKDASREAWEAELKA-<br>VQTSAAQALADTTAKYETWLRQMRLQRDEHASAREELKVRVSRMEETISRLKE-<br>QIANEKKYENELSTRVSNMSAQLEARDALVVKLQENVRKLEQSLKVAENA-<br>GRAAEEQLVEARELADQYSHEASAMRAKCKSLEQDLAIQVQSGEA-<br>EQARLKEQVAKLTKSSAESEDTIRTEFMKQLESVVEQYRHQCNEDK-<br>ARITRELKSHYMPKLTEARQALEETCNREAELRESNKVLTEEITLLK-<br>GQLETMQELKTVHEKRINELVAELDKERNVIHVKAIAEKEKEIEKLR-<br>QRYARLETDFDNLMDIKIQMALTIDKYREILTEEERRVGLETPGRKR-<br>KRAKVVEDPPTLPVQLAIDEENGCLVVENVSDEDFSLDGWSLQSSG-<br>FVFEFPEDTLLPAKSRVSVWIGEAPNQDDEEQEGDDEGQANQVL-<br>VWEGVDCTELAEEPMCLCNEDGAVQDQIEVEAVRYASDRSSTRGGCLLM</td></tr>
<tr><td>Spongo-spora sub-terranea</td><td>Rhizaria</td><td>UniProt|<br>A0A0H5R4<br>D7</td><td>MATNVNVEESPIRIHPEVERKNLEELNGRLQQYIMKQRAKDASREAFEKDV-<br>VAIQQQARVAIHANTKKYEEQLRQMRQQRDEHASAREELQVRVARQEATVTR-<br>LKDQIAEEKKFQSDLNGRLSSLSSQLEAANAQVQRLQEQLRKTEHNFKSAE-<br>SAGRAAEEQLIEARDAADQYSHEASSLRAKLKSFEQDMTINAKASEAEISALKAK-<br>IEQLSAQVTITEDSCRQEFASQLSSIVEDYRKQCNDDKTRITRELKSHFVPKLAE-<br>LRAAYEESCQHESELREINVTVTEQVNQLKKDNQTLAEMKSLHEKRIAELSEEL-<br>DTERNVTHNDAIAAKDKEIEKLRHTCQRLETDFDDLMDVKIQLALTIEKYRELLS-<br>EEEHRLGLETPGRKRKRSKRSLPAPQTPALKLWVNDEVENCLFVKNTSDKIVN-<br>LNGMILQSGSFSFDFPDDAYVGGQCEVCIWIGETSNSLEASSEDRTNISWEGV-<br>NPEDLTAAPMQLIDQNGTVIDQFEVETISAGDDKNACLLM</td></tr>
<tr><td>Cercozoan plant para-site (EST of Beta vulgaris)</td><td>Rhizaria</td><td>NCBI| KM-<br>S94577</td><td>VRPRSLISDMTAADVVGESPVRLQRDVEQKNLEELNARLEKYILKQRAKDAS-<br>REAFEKDLAKIQQQARQAIHANTKKYEEQLRLMRQQRDEHASAREELQIRVS-<br>RQEAVITRLKDQLTDEKKYQNDLNQQVSGLKSQLEEARGQNSKLQDQLRK-<br>TVHALKVAESAGRSAEEQLAEAREGADKSSHEASALRAKCKSL</td></tr>
<tr><td>Cercozoan plant para-site (EST of Festuca arun-dinacea)</td><td>Rhizaria</td><td>NCBI|<br>GT045861</td><td>MTVADVVGESPVRLHRDVEQKNLEELNARLEKYIFKQRAKDASRESFEKD-<br>LANIQQSARLAIHSNTKKYEEQLRLMRQQRDEHASAREELQIRVSRQEAVITR-<br>LKDQLTEEKKYQNDLNQQVSSLKTKVEEAHGQNSKLQDQLRKTLHALKVAESAGR-<br>SAEEQLAEARDTADKFSHEASSLRAKCKSYEQDTSISASANENEITKLKETIDKL-<br>QAQIAITEDNCRSEFSSQLAKIVEDYRKQCNDDKTRVTRELKAHYMPKLTELRV-<br>SLDEAMRRENDLREANIALTDQVSQLKREMQSLNDIRTVQEKRIQELTEELDRE-<br>RNFTHSDVIGAKDKEIERLRHECQRLECDFDDLMDIKIQLALTIEKYRELLSEEEH-<br>RLGLETPGRKRKRSKRLPEGPSLQLGVHEQLDNCLLIKNASGETMNVNGFVLK-<br>SGSLDFHFPDNIQLPAYGEICVWIGEEVPEQMWGAS</td></tr>
<tr><td colspan="4">LTD-like domain-containing proteins:</td></tr>
<tr><td>Nanno-chloropsis gaditana</td><td>Ochrophyta</td><td>Uniprot| tr|<br>W7TNN4</td><td>MRRLIEIPWLDECDQDGTSARCQNPRRRSILYRSPPYISDVDVKREIITIS-<br>NPESVPVDLSGHSLCDKGQHHRYEFEEGYTLAPGAELHVY-<br>CAPGKYGVEEEERPGELQILLWTTREGRPRRREVLNQEGDRVTLIDQNG-<br>MEIAALEVTVNGDDEEEEGEREKRGTGKAERREEIKRLYLYRLEVLQLKYAKSVVT-<br>WLQGSRLALMVLAFIFVLLNDLHSYVLLEVGGFALDMAARYLDPVRLPRSRQRVFA-<br>LVADRLDSASLLAVLMTGLVETGGPLYGPNPVHRVRLGLGMLLIEVLAFWFEVLVE-<br>GSTRLAAADAVAPYWRQGPAPAGEEEVSVQTGPYRKWLRQHLGRCPALITFATGG-<br>NHLFLVLAVVRLAHAQAGGSEGLVVRAVSVASRIMAYLPLPRAIVSNWQFLLIAVCL-<br>SAMYRCILIWAELLDLLESKL</td></tr>
<tr><td>Ectocarpus siliculosus</td><td>Ochrophyta</td><td>Uniprot| tr|<br>D8LHR1</td><td>MATAAFHVPSVPRSLLLPGLDTHQVQEEDDVPTDPTPPGSHWLFRPPPFIA-<br>HADLEAETVTIRNPSVWRGANLAGYTLTDRLQRHTYHFPERFVLRARTSLTLYC-<br>CPGKFSEGALHNLPQPFLLWHNQDGSLRRKEVMENSGETLVLS-<br>DAKGNEVAVLEVPGTDGEETSRTLPGVKRSQVLALRTLVLSLCYL-<br>RLVCTGLAAARVTVHPDVFLLVTALAHVFDILSRWASSRPGVPMD-<br>DFGVVLATMGDRFSYLVLLGSLVLLDKDPRHSQIFGGMLALDMTA-<br>NWLQLSVASISNGSHPLAGTLVSGRGAAPDFATHIMLTHPVELTLV-<br>SLGSEAFLVWSYLVASSDLPLGLRAMVAKAGDFLSSLFTELLSSFS-<br>PSSSSSSSFLSSDIADEEVASILISGEETTTAAEDGGVFGGFEPNR-<br>IDSAALGLGEDLGVREAEDDGDGSPFSTAWKAVWMTLMVCCAAR-<br>QLLSCIQALISMSSLSSGVIADAAGKDMQRRHPRSRRSSGGGGSSRGRSRSRAR</td></tr>
</table>

| | | | |
|---|---|---|---|
| Saprolegnia parasitica | Oomycota | NCBI\| XP_012194028 | MASAKLRRVFLPTLDRRVARASMQIYSATAMTGMYISRADIEEQWIVVSNPSAY-<br>DIDLSGYTLANENGTRIFRFPRHYIIFAGEETTVWCTPGATAFNPRAL-<br>REPYLLWTQPNGSLAHTPFFNHKDAQHDVLLLDPYLSEVASLQITASGHKT-<br>FRVRQCRAPADGAYVFARYSNVASDASSLAHVVAVVITPVLEMGRLWLLMTLLL-<br>QVHWVPASTEPHLLVYAFVCDIAARVASLFLQHASLASFLAQTSMGIDQLHVVVV-<br>YLALSTAYPAISIFRTLMTAELTANFVGLLGADRVAQQSRWHPMHMMLEGAIYIHP-<br>LIFAGCYLAKESLVYMLFLKATLPSTTIAMHALIYLCVPLFAVSTAISIARGATVAL-<br>HLADLTLARLRYGPP |
| Micromo-nas sp. | Chlorophyta | Uniprot\| tr\| C1E2Y2 | MGKVSYKAVLAGALLAASGAHASVVINEVAPKGDGVKCGGNDWIELYN-<br>GAAAEVNIGNFQIHDNKGAGDADTFTFAAGTKIASKGFLLLCQKDAPTAATE-<br>FAFGIGGEDTVTLLNAAGTVVDTTGKLPKVDFEVTDPSPTVYKTYAREKDGA-<br>GAVDGTAFKVLRKPSPGASNKDMLPDKLQVYLNEIADKGTDMAKTVACDPPADYV-<br>EIYNAEPDAVNITGWRLHDDNGKDHKDAYVHSGAVIPAGGFMLLCGEYKDGTGG-<br>FHDFKFGIGSDDKITLLDAANNTMDTAGPLLKRGDTTTVYGRTEDGSGIWTYLTP-<br>PTPKKTNKGMKYKPEGDAAATAPPGSSTVVLNEIADKGSDAVCNGEDWVELYNY-<br>GKNPVDIGKWHLADEKGVLHADVHIFPDNDKHVIPGYGFLLLCGEETFKFGIGGT-<br>DTVTLLNDKKQVVDEIELTGLGSPTKTMQRKVDGTGEWAYSENPAPSPGKSNCV-<br>GGCPLPAEDDDKKVSGGVIAGAVLGTVAGATVAGVIAHRVVNNYNKEKVPKTDRG-<br>FIDVNNV |
| Bathy-coc-cus prasi-nos | Chlorophyta | Uniprot\| tr\| K8EQX8 | MNTNTLLQVLLVLLAVATFLGSTECKVIITEVGANSDINDSTGSGADFIEIHNTGSIGT-<br>DAVDLAGYKLADDKGVADADVFTFPAETPPLRPKETRVLVKGDPSSFAFGIGKS-<br>DTVTLFNAAGIEEDTTGPLDGGDISGQTWQRLTNSAHGYGTDDTDLAAFGRQ-<br>TYMTPGRLNHPVKVFISEVSSKDSAADSVCSGVDFVELYNAGTSTVDLGAGYSLS-<br>DDKWHEDEDSWDIPAGTTILPGEFKVFCTGTHFAYGIGKDDAVVLHDPSDIAVDTT-<br>GTMDQSQHVEGYSWSRQGDETNEMDFVSFATSVQQTPGASNNVGICTSLVLNEI-<br>ADKGTGTSTCNDEDWIELYNNCPTAINLHNWKLHDDKGVDHEDSYLHGNSDVIIEA-<br>GGHHILCGATDFQFGVGSDDTITLISPGGDTVDTVGPLVAGRGNSSVTYQRQADG-<br>TGEWTYRTPPTPGTTNGASTYVPANGASLYAPPGSVTVALNEISSSNTSAAHCNG-<br>EDWIELYNYGCEDVNLENFYIADEKGHLHADVYRFPSGSSIQAKSLKVLCKITDFAY-<br>GIGGSDTISLHNKEEQIIDSYALTGLQTKENLSTMQRSPDGTGDWTLLKASPNAYNS-<br>ATQGGTSCSSKSALSSEVLGGAIGGAIVGVLLIAFVLVKTCTSKKRILVQEIRKDVRA-<br>RSDDPEFGLGMRAQYEQKGKTSLMLTEY |
| Bathy-coc-cus prasi-nos | Chlorophyta | NCBI\| XP_007514062 | MMVSSSHSLRRVPSLVFLLFCFLLMVSLFDETFAAMRINEIADKGSDNTCEGED-<br>WIELFNDSTDDVDLEGYILHDDDYPKEVGTELTFGAGTSLSGGAYLVLCCND-<br>NDGINNPKFKVGKSDTVTLLDSSKNVVSTSGKLPGLGEENVTYAFSAL-<br>GEYVQTWTPTPGELNVITSKPLKDLRAQNDEGSSFFSMDEKGLPVSGFDAVVDLY-<br>AEVDAGDWAILRLNPYAETYIDVQTFKVKSSTIGEHILPSPGRMRTRGQSTLFYPV-<br>CMGSEAVPFKLDFASVNSSQTLYGVESAYLRTHLSDESKMKEWVVHRMLANFGLP-<br>HVRTRHVRFHVNDDLLGFYTFMEAVDQEYVSARSFGFNSFDQHNNALYKVKSLSL-<br>GCGNEEEYQKERVEGFPEKCTSPDGMGGFDCCASYAWGEPKTCAPGYIVKEQP-<br>SRSDWGIENCWYTCLSNSEDEVTSGGPYQFERGEHREKITPRYQEDWCWAEFF-<br>GRIGKERLSVVQAFYETGYDTADDCGKFLLSQGLVDRDLGTNEWDSAMEKFINSH-<br>LSVKGACQNKQCSNKRQISDLIDVDNWLKNFAVYATIIEQDSPMGNGNNYFLAAHA-<br>GDGGTDTPKWKMIPYDHNNDVYAASTLCDAQCGNKDLTEWSVIRPTCKHLSENPL-<br>VGPLLLDESLHERYLVFVKQFVEETFSNSTFLEHMKLHSKEIEATANESPDSVIYGNI-<br>DNSNIWDWISTRGEKVSLQLARWYEKSDNSAFPIDSSVPCVTSDEEFCSSSLCTS-<br>PNGLGGYDCWAGTFFEPCTCSAGVAKETGESTEYLGQTYYSYTCCTDGSGTGQ-<br>QCGLFSSGIKSNGLLGILFAGVVAFALV |
| Bathy-coc-cus prasi-nos | Chlorophyta | NCBI\| XP_007512269 | MRHTKRRTSRATPAHEEDEMRTRCCFRKAGLARLPFLFFFFFFFFCFLSC-<br>FAFAFDQEENERGASANAFSMTTRTPKLELSSSHSSSSSQALGERRRTFLETNES-<br>LVVRARLENGHGYERKVYLKFLLGTTSNEEEKFEKMRDDGKYPDE-<br>TANDGVYAVAVDVGKELGAKNGDVVLFRAEAWTESGDKKPLRSPKEDAKKDD-<br>REVTVVSQSQYSYYAVAIVSPSLKSDTNLPVLHVLRANEQDMTTDRGTTAKVF-<br>FDGKFYDEVKIRRRGSGRDEEKVGVELPPKDWPKHKFKFDFKGEQFVWNKG-<br>EPAVEEFNLNSQWQEPGEETYMRLNLANAIMDVAKVPAPTTFHVHVQVNGKY-<br>YGLFSFVEQIDETYLKRRKMWDEQNSLYKAVNWKYSNLRAPNASLSKCPWA-<br>TPDWPRRWSERDGYCPQIWRKSYPAKSKTVDDLWDFTTNIARAKKYALGTS-<br>QQVYAEKVLFQILEVPSVINEMAAQTILLNNDRCAKNYYVFRNAKTKRWSRLP-<br>WDLEDALPADNRYGLVLCPKRDCSPKSTRYCVLTCEKFNSPLYCDSEHPQDI-<br>FYSEGIAQEAKSTYNELVNVILKTDRTRAMYFARLRTLMNDILGTNFIERYVART-<br>LKTIRRDALRDSVKWKVGGIRSIDQGVLQLLSQSVKLRREQLFETYSDMIPKT-<br>VTEGEILESVNIKAFSDIDDTGIGLRSWIEIENNLPAAADISGFSIACGSNKMKVW-<br>TFAPGTVLDAKQSIYVVADESNFLDDITSNAKDNYYFFVQHGNDKFIERLEKCV-<br>KIEGRSDSLSEEKKVFFSIEPRTVDRALKTLSSSPLSIDSSNCTEPEAGPWEE-<br>WGECCRGNKCGVYCDEPDASRGRRRELNFDKFIDQLLREEEEEEEKLNETP-<br>KQQLCYPSLTANWQVDKKCGAKKCGERRVPYTCTTALQYAKKIASSSSSSSV-<br>LFGAGKSESGWDSSFSWGVKERNNIAIGIDGQEDGSGSTGSVKIQLYGEGAF-<br>ALGKKITDGKVFLETFSRFNRLNVFVARERYPQPLPRLTLQIEFYFWRDGGVF-<br>SNNDASVIFSSNARFETATSDDGWQSGCYEKAFASFKNEFTRGDGGESLSDF-<br>LRNKKQLFESADTVTIKVAVRNASRKSPIVFYVGSVAFVA |
| Volvox car-teri | Chlorophyta | Uniprot\| tr\| D8UC51 | MVGQTAAGVGSGNSDVRINELMAANQGAVLDERGR-<br>SPDWVELFNPGQGEVQLGGWMLRTPKENWTFPQESSIPAGGYLLVYTSGKSQSS-<br>NDAATGPAGSPPPIRAALKLSADGESVSLLRPDGSAASLVGPDMARQPANIS-<br>FGVPGTADSGDVAAASRDAAVAAPLTFLARPTPGAANSGPMAHGPFIHSVSRATQ-<br>PPRAPAGSDIPVNITVEPNLQNIDEQNVVLSYVINFGEEQDLPAVKVASGSGRNTVF-<br>TASIPSSAFKSGDMVRWRAKVTDTAGHQSIFPGPPGPSSSATTDSEGKKYPRYFG-<br>TVVAIPASDLVTDQVPVLEWFSPDPAAATTRDGAEGQVLFYNGCLYDNVFSRRRGV-<br>TALNWPKPKLKFELPQTNFEYAADMPPVGEFGLQSFWYELGERSYMKETLALQLM-<br>RDAGVLAPNSFHVHVRQNGHFYGLFAFVEIPDMDFLSRHKLPRSGPMFKSVSGEL-<br>SNLRWDLPIKEMPAYWEKENSRQRDTGDWVLLANLSKGLAGGSPVPRSKYIFDAV-<br>NLPQDLLRSTPWGQVSATLNGAYEGPAVRKSPGGQRKRRLLQQQQQQQPQQAT-<br>QQDATPPTVPRTRRRAGGASQPGGGGGGAAGMEFPYKAAGNNITTFPMPRGWD-<br>NPDRSTISQTSPNGPTGTYNHLYDAILDVASARAMYMRRLRSMADKYLAGGVLAQ-<br>FANQTYVRIKPLADLDAKTWNSGISIDRGFQQITQEFLPVRKEQLLGALYGPKGRRP-<br>LLPEAQPAQVSLRIARFVTAESGTTGGDASGFLEVANPNQFAVDVSGWRLEGPATF-<br>TFPPGTVIPSAASAFITVDPVEFRSRTTPPSGGQGLLVLSPLQLGEAAAQGGGSGG-<br>ASSAASSGPYKLIDTAGTSVHQMNTGTSRKLS |
| Auxeno-chlorella proto-the-coides | Chlorophyta | NCBI\| XP_011395789 | MRGITLPGFSAHYLPALPTWQVLINEILASNTAGLKDSSGKASDWLELRNTGSST-<br>EDLGGYVLSDGENSWTIPSGVSIPAGGYQLIFASGKGSKSGDAEVHASFKIGAQ-<br>DAALSLSDASGNVVSAVPYPTTPTDVSFGVVGSGQTVQASS-<br>GAAAAQYAILASPTPGATNSAARTGSPSAAGTTRDPSPRPDGSSAIAIQTSAFP-<br>SQSPIAGVDLVYTINYGKEVVVPMRAQSPGSSVYAGSIPASAAAAGTLVRWHVV-<br>VRDEAGNEGRDPAPPETTDPQNFGTIVADTSDSTSLPIFELFCADANAPWSTG-<br>PESGGQALTGGKGYVDGCSLWFNGTYYDNVSLRRKGSTSLAWPKPKMRVSA-<br>GNQGKVFATSAGYKVKSFSLSANWAEPGENTFTREPLVWKTFQEMGVDYLES-<br>YQSHVRFNGAYFGRFIYVEDWTPESLKRNGYDTSDIGSLFKSESGEYSNLRWD-<br>LPKDQVPFYWGQDEPKADESALLLELTRGLAGAGSKERENYLFDGLNLPKVIN-<br>YMAAQTLILNQDRCTKNYFVYRDRPSGQWSMLPWDVESGFGIDRGLGGVPAP-<br>DYCILACDQWNSPLYCDRNHVQDLTVSTPWTLILSNYSLGTSSGVSTAGRRLLR-<br>SDVVDRQLLQAAAASGGALAEIAPNGAAEVGLKLPLNSTVPANAQDDQTLLGT-<br>PPTGANGTYNYMLDAVLSFPRTRSMYMRRLRSLMDEFTNGRLEGLVTSFYEQV-<br>KEEATRDNAKWGNPGTPLRGYQSLTTEQLPIRKQQLYNTFGVDGPIPLIPDAQP-<br>SSYRLNLGSVSGDAGGYVELVNPNDFAIDLSDAKLSGAAEFTFAKGTTVAAGDS-<br>VHVAADVGAFVKAKGGAGLYVVGPFSGSIQGPAASVSVQSAGGDSLVG |

| | | | |
|---|---|---|---|
| Auxeno-chlorella proto-the-coides | Chlorophyta | NCBI\| XP_011395790 | MCRSSTSRPVPHGYQLTVSKASGKASAWTFPAGVSVPGNGYLLILADGLDTVSG-<br>SEVHAGIKLSGSSQGLSLLDASGASIGSWDLPELADDQAYGVAEGDVQALDGPS-<br>GTSRLVYLSQPTPTSANSGAADTGPVVARVTEDPAPRPNGGSAIPISAVVTPQQAT-<br>VAGATLTYVIGYGSPVSVDMAATGGTYTADIPAAEAAAGQLVRWYVTATDA-<br>GGRNTVVPAKDSNGAVQYFGTIVADPTDSASLPILELYSENDRAPFSTGPSS-<br>NAIAVPGGKGDVTGCSVWFNGTFYDNVSIRRRGATSLGWPKPKIKIKSENG-<br>KVFKLGGLDYEVSAFGLNSNWFEPGANTFTREPLVWEAFNQMGVESLLS-<br>FHVHVRFNGKYFGRFSLQEEWSKDALERNGFDTSSSGYGPFWKSNSGIG-<br>TNLRFGVTTTDLPVVYELESSKSKQDAAVQALEAFTAGLAGGGPVARSKYL-<br>FDAVNLPQVINYLAAQTLILNQDRCTKNFYVYLDASSGQWSMLPWDVESG-<br>FGIDRGLGGQPAPDYCTLACEQWNSPLYCDRNHTQDLVISTPYGRISQPVT-<br>LGATSGTPASRRRRLSSLQTALGTPRLGRKLLQSLFQDTTVDTGAGQDLD-<br>SDYDANLTSGRPASGAIGSYNYLADAVLSLPRTRAMYMRRLRTLMDEFTGG-<br>RLAGIVTTLHTQIREEAIRDNAKWGNSGNPDTGYQQLIKEQLPLRKQQLYD-<br>VYGPGGDHPFIPGAQAAGAGLTIGQAHTDAGSWLELTNPGAEAVDVSGW-<br>ALTGDVDFTFRPGTVVDAGDTIVVAADILQFKASYGGQGKYVVGPLPQPLTSS-<br>SPSVSLVKA |
| Helico-sporidium sp. | Chlorophyta | NCBI\| KD-D75179 | MVYISEILLDSSESDSSGWVELGNSGTNPVTLDTLSIGVLDDGPSAAARLPGNITIH-<br>PQSFIIVSLGRHGIAGSVPSTLRVPKARGGSIAVWDDGQLAGRLTYP-<br>PLLKDQAFGLTLPPTYNESLIQAASVSNSPGSPSLLQSPTPGAPNSRLRQGP-<br>PAIVGTTSGLAALPNSQGNATISAAVLPQGAEIQSVQLIYQQGYSEPQTLLMLPTR-<br>CGHFTATLPSHLFTPGALVRWQVAATDSQGRTALDPPDSEGARKHGIIVRDAGET-<br>TFLPLLQLSCPDSNAPWSTGPETNAAGRTGGKGGVMGCSMVFSDEFYDNVFVR-<br>RRGASSTAWPKPKIKITSNKQGKLFRYPGQTYRIKALYLNSNWMEPGENSYTRE-<br>PLMWDALGAMGVPALGAFHAVVHFNGAYFGRFSLTEEWSLDALEARGLVSRSAP-<br>KPVLWKANSGWMSNLRWDVSRRQIEQYYGLEESPSGTAGAVTELEQLMTGLAG-<br>GDSLPRSEYLFQALHLPRVINYMAAQTLVLNQDRCTKNYFMLREAATGQWSVLP-<br>WDVESGFGIDRGYGGVPAPDYCILACEQWNSPLYCDRQHVQDVPLAGTEGTWD-<br>FYGGPVPPTGGGQPFFSFLFQTPQPQPSQPFQPQSQVQPQSSPQPQSQSPTLQ-<br>PATVSTTAEAVPAQPDGARAPQPPQFDPGAIFMRWIFGRRRLSSTGRSLKQAPET-<br>VAAPEPEESSPSPQTISDAPDPFYNVDQTKNRNAMVYNYSSSFSWLADAVLGHP-<br>RTRSMYMRRLRSLMDEFTSGKLEALVHKWNNVSSMEIARDCEKWNCPRDPTLG-<br>YQQLIVEQLPIRRAQLYDTYSVGGTHPLIPGPAAAEAQQTVAVEMFMEDLSGSLV-<br>LRNPSADAVDLSAHKLVASLDYDFLPGTVIPANDTLIVTLDVVGFLRRHPGK-<br>GYFVVGPAQRGTERRAPKIVPK |
| Chlamydo-monas reinhardtii | Chlorophyta | NCBI\| XP_001699673 | RCTSGWELRLLDHDEEDGNDSSRSSNTWTFPPDTSIAPNAYLVIYASGKNVSAG-<br>NATAGSAAAPASLHASFKLPAAGGVGVQLLGPGGVVVERVQL-<br>PRLVWLQQAAPLAGPPASHNLTTSRRQYANVSFGVPAAADGVKRDAATAAPLTYL-<br>SAATPGADNAAPLAIGPIITSVSRPTGPDRPPAGADLLVNITLRPNLNPVDGAGVSLV-<br>YVVNWGQEQELAAAQEGPAGADGELLYTAAIPAAAFKAGDMVRWRARAADTAGY-<br>RSALPRPPPSAGTAAAEEWPPAMAAAALGQAQGGEGQEQEPHYYGTAIAVLEDQV-<br>ITPDVPILEWFSPNAEAATSVDGALGQVLVYGGTLHDNVAARRRGVTALSWPKPKL-<br>KFILPSRDFRYSDTAPEVSEFGLQSFWYELGERSYMKEPLALQFMREAGVIAPTSF-<br>HVHVRLNGQFYGLFAFVEVLDDTYLQRHGLPPSGPLFKSVSGELSNLRWDLPLKE-<br>YPSFWEKENRDKVAADWDALRNFSRGLAGGGPEPRSEFVVQSVNLPSMINNMAA-<br>QTLVNNMDRCTKNFFMYLHPHTREWYMLPWDMDGSFGQDNGLGGKPDLDNYCV-<br>LACEQWNSPLYCDSEHPQDLSRVTPWGTVAVKLNDNSYQGAAVRKPSNPAGRRS-<br>RALLQQEADTAANINKSPMYAAGPTITKFPPPRGWDNPDRVTLTQTSPNGPAGTY-<br>NHLYDAILDTNATRAMYLRRLRTLADTFLASGRIAQMANATYTRIKPLADLDAKKWS-<br>SGISVDRGYQQITTEFLPIRTDQLLGSLYGPTGRRPLLPTSQKPEQGTEAVDLSGW-<br>QLRPAAAAAAGANLTLPAGTVVAPSSSVVATTDLATARELQRRAGAAAPAGGAAAA-<br>GAAAEPYVELSTSAQGSGKAGGSAPGSGGGGAGKWELVDPSGAVAGAGGGV-<br>AMQTKK |
| Mono-raphidium neglectum | Chlorophyta | NCBI\| XP_013901328 | MPNYWGKANRKNNPEDWQLLANLAKGLAGGGLVPRSRFIFDALDLPSVINE-<br>MALETLLGNMDRCTKNYYLWYNPKAQTWTRIPWDLEASMGQDNGLGGAPGNLY-<br>CLLACEQWSSPLYCDSEHPQDLEVRTPWGATTVVGLNSYFGPGRGAAGGSTAQT-<br>GRRLLQGGAAGGKLLAEGNQQLSFPAPRGWDDPDRVTFGTPSPNGAPGSYN-<br>HLTDAVLDVNATRAMYLRRLRTLADKFYGPEPDKGRLRQIIDETWARIKDAAAR-<br>DNAKWARGDGTRGAKQLLQTYAPGGQRPLLPGPQDSSKAKISVTKVETSGPA-<br>GEHYIQIANGSPDEAVDASGWTLSSGDASWALPPGTILPGGASAFLTWDPPAF-<br>KARKASPKGGEGLLVVQAPAQMMALAGPGQWSVKDAAGAEVSKASL |
| Ostreo-coccus tauri | Chlorophyta | NCBI\| CE-F99931 | MDARARGARPRRGRRANEGRGLGAALVVVTCALLGTGTGAGAITVDVDVAS-<br>GAGLGSGDAVEVRAVVEGGGAATAARATCALGWGAERTTGMRYDGNDERGRM-<br>RFTGWCDTNGAPEGERVRVKVSVAAGTARATSAWRGAALERRVKEETPLPIL-<br>HVWTPDYDKITTDAGERVAVYFEGRYYDDVFMRRRGSNRNEAVIGVELSAANW-<br>PKRKFKLDFGRRRFKLNANMSRVSEINLNSHYQEPGEETYMRENLGFAILRRAG-<br>VPAPLTRHVHVRVNNEYYGLWSLIEQVDKRFLKRNNLFTGDGYSLYKAVNWKYS-<br>NLRAGDPSLPCPYATPDYRREWMNDGCPEIYRKASKPRDKWDDLWQLTNDVIE-<br>RVRRNPEGEAYLLFDHLNLPSLVNEMAAQTLMLGADRCTKNYYMHRDWTGEWT-<br>RIPWDVEDIFPSDKRYGTSLCKPSECAATANLYCILSCEKFNSPLYCDRNHPQDIF-<br>YWDGSRPEQDPRSTYNVLIDVMLSVWPVKEMYLTRLRTLMDQILATSFVDDYVR-<br>RKVNLIRKDALRDSKKWGVGAGRAIDQGVNNLLDVIVPTRRKQLFEQYSYMIPPS-<br>IPSNARVYVSHAQRSGDESYVKLSNPNGFAVDVSDWIVSTPGGWTWTLKPGSV-<br>MGPGRVLFVVKNAKEFRNRATWARREYPQGLFVQGNFYRDLDVDDPRMFTVKPA-<br>RL |
| Ostreo-coccus lu-cimarinus | Chlorophyta | NCBI\| XP_001420675 | MTTDEGERVGVYFEGRYYDDVLMRRRGSNRKEAVIGVDLSAANWPKHKFKLDFH-<br>GRVFKFDENERKVEEINLQSHYQEPGEETYLRENLGFAILRRAGV-<br>PASLTKHVQVRVNNEFYGLFSLIEQVDSTFLRRNYLDPEGALYKAVNWKYSNL-<br>RAGDPNLPCPYATPDYKREWMNDGCPEIYRKASKANRDNWDDLWELTQ-<br>VIERVRRNPGGEAYLLYDHTNLPALVNEMAAQTLMLGADRCTKNYYMH-<br>KDWTGEWSRIPWDVEDVFPGDKRYGIDLCKSSECDKKSTAYCILSCEKF-<br>NSPLYCDRNHPQDIFYAQSPEQDPRSTYNVLIDVMLAVWPVKEMYFTRLR-<br>TLMDEILATSFIDDYARRTLNIIRKDALRDSEKWGVGAVRAIDQGVLQLLSQ-<br>IVPKRRDQLFNQYSSMIPSSTPSNARVYVSHAQKNGVEAYVKISNPNGYA-<br>VDVSFWIVETRGGWKYTLKPGTVLGAGRTLFVVRDAKEFRRRATWARR-<br>EYPEGVFVQGNFQSDLDTDDVNMISVTRTSERVAALNGRGRAFRRFRP-<br>RVGFDNTSCALPDVGPWSEYGDCCAEGKCGLWCGVQNAARVRRRDVN-<br>ITLGLDPNDLPQCLVHSYEIDRRCASLECGTPRPPNSTACRIPSNESFVR-<br>LSRNDQSVIFSPIFGLNTRTWEDWSYKVEVAPHDAHGVDGASKSLKILVH-<br>SGGGLAFRATARNDTRLTSAHFWIARHAPPQPLPRLVFDLESTSDNTTTT-<br>RAAPGEFLRGIARAQSDWNAGCYVEIIADFAGFDDSPTTAAREPSSVKIV-<br>LKSAFGSPTRLWLGRAVAVFSSHENAP |

| | | | |
|---|---|---|---|
| Emiliania huxleyi | Haptophyta | NCBI\|<br>XP_005763<br>134 | MPPPPPPRGWGGGLAGSLHRVDPAARPAVLARAVRDASSGLAVHLCGVSHVD-<br>PASATAVRAYAEALAAAPGGLGAVAVEDEPRALLLTRAARAALHGLPPERI-<br>RAEGEGLVRHALFGLAEVHAWAAEAGATLDGAERAEAAEAAEELGARVACLGGT-<br>PAPPPHVPLLTRRGAPHGLTVHPRAVRRRAAVRLAYWLRVRALDPGHDERSPAEL-<br>HVVASNRALAEAFFRASPPVAPSAHEAVLLRKDAHFAAALRTVCADAARAGGEPPP-<br>AVLAVVGATHVEGIAERLQAGDGTPRNSVPLLLVCSVSANVIINEVSDKGTLGTCDG-<br>ADWVELVNVGADVVDLDGWMLCDSGGCQDNVNGTIAMAGTIAPGEYRLVCPQAQ-<br>PKWVGDSDTISLVDEHALEADITTLGGDGEFGKSWARWPDSTGPFRYTYVPTPGA-<br>PYADSVIINEVSDKGTLGACDGQDWVELVNVGAEEVDLDGWSLCDSDGCADNDTI-<br>ALSAMVVAPSGYLLVCPQARGWMHRKWISDSDTITLINAQGMQADATMLGGDGEF-<br>EKTWARWPDSTGSFRYTFHPTPGAPYAKSVIINEVSDKGTLGTCDGADWVELVNV-<br>GADVVDLDGWMLCDSGGCQDNVNGTIAMAGTIAPGEYRLVCPQAQPKWVGDSDT-<br>ISLVDEHALEADITTLGGDGEFGKSWARWPDSTGPFRYTYVPTPGAPYAEAVETLQ-<br>VNEVADKGDPTDPCDGADWVELRNPTHGALPLSGLLLSDDKGPHNEKALALGGP-<br>GCPFSLGTGELLLLCRNGNASMGGANYAGCGFAFGIGKNDVVSLHQIDAAADRVV-<br>LVDEAVGCCAGDANLSYGRPPRGGAFSILPWRTPGTANAVAARQPSPRVTPPSVL-<br>RPLSLSLSSPFPSSVVSFEEGSGAGFGKDRFPEVVLGPPQGAGNAGSLDVLSLGE-<br>AGEVVLRFDGIELVDGDGPDLLVFENPFTNWYETGVVAVSADGMTWFEWNCDPE-<br>DATSLYPGCAGVGLVWSNPDNGIDPTDPESAGGDAFDLADLDIPPAHLADLGVRG-<br>FRYVRIRDSGRNSYGGISGGFDLDAIAAVNGEPLPAVSTALEPVAEQIMRSDCDPAP-<br>DLDGDGAFRLADAVYVAEVWSGSKQWSRGAMPCGDPAGDFDGDGAFRLADAVY-<br>LAEVWAGKKVLGPTSDHRLLIAWKIPMEITEHEVTAGTTVAFEWQNYHNVMLLSTQ-<br>AEYDACSRTGEIVAGTEAPQNQKVKIVVK |
| Emiliania huxleyi | Haptophyta | NCBI\|<br>XP_005784<br>342 | MAAVTCTSTPPPAEYDDACTEPRVIISEVAASGADGEPCHDDWVELFNPHPTR-<br>SAPLLGLVLSDDHGFDRSDRLALGASGCPTSLANRSYLLLCRADSPCGFAFGI-<br>GRHDVVLLFNASGSPLDETAGCCANSAQRSFGRSPGGAWTELDVRTPGAPNTG-<br>VAHAPPPAPPTAPLAGSALGLGDLRHVATLVVPPPRPGAAAVVDLSGVAVAAWED-<br>LSSGGWRATLQTVVNSEPAVIEVEVEATSAASPPVLRRRRTFLLEGFEDTEGIALL-<br>PSGQVAIAQERRMAVSVLLLPPASSSEGDTASHLETRLGLGAANTFDTRLPAERN-<br>KGLEGVAYDPEQGLLYAEKSPIRVVALNLTTGAVADLFDAPSVLGRHVSDLAGAW-<br>FAPRQRVLLLLSQESRLLVEVRAVQRISGTQPEGVALSPDLSTLFLASEPNELLVY-<br>SAEKKGTPIFKAVGASGQTAGVEARSSYA |
| Emiliania huxleyi | Haptophyta | NCBI\|<br>XP_005771<br>646 | MAAVTCTSTPPPAEYDDACTEPRVIISEVAASGADGEPCHDDWVELFNPHPTR-<br>SAPLLGLVLSDDHGFDRSDRLALGASGCPTSLANRSYLLLCRADSPCGFAFGI-<br>GRHDVVLLFNASGSPLDETAGCCANSAQRSFGRSPGGAWTELDVRTPGAPNTG-<br>VAHAPPPAPPTAPLAGSALGLGDLRHVATLVVPPPRPGAAAVVDLSGVAVAAWED-<br>LSSGGWRATLQTVVNSEPAVIEVEVEATSAASPPVLRRRRTFLLEGFEDTEGIALL-<br>PSGQVAIAQERRMAVSVLLLPPASSSEGDTASHLETRLGLGAANTFDTRLPAERN-<br>KGLEGVAYDPEQGLLYAEKSPIRVVALNLTTGAVADLFDAPSVLGRHVSDLAGAW-<br>FAPRQRVLLLLSQESRLLVEVRAVQRISGTQPEGVALSPDLSTLFLASEPNELLVY-<br>RRPPSPLSPPPPSLIVEGGVIAAVGVVSVYDPVNLETVGTLDVGAAMALECQEVD-<br>CIYEDMDFGGGSAPCGYAPMKNIIFDKGDCSACAESGTCDASMCVYTSNPGYN-<br>AGTLSVDASVSTVEGCQAACVADNACEFFSWENEVWANGDTSFFGETYNGGPY-<br>GTASREGADISSTDCESFVHWEASHHRQHHPPAPSPPPAHKSQTHRGDLCTGIT-<br>EEQCNGIPDAQVEGGACTLGGAGRSGPNYGDDVLCTDLGGTVSSPEWVGGPR-<br>YAFGFAPGNHYTHVYDKDWIGASGPALCGNFRAESVGVTHVPNYPNAIVAAASP-<br>HHYSFADGMLAFFDAKTLDFLAQDLVHQRGVGARTLRDPARRRGLDDHDFSPPN-<br>FLPGAWKHAPEFRTDMVRLFGPNGNNVTYDMSPKAVPQREAISTKKGGKSTYDP-<br>IAVAPEREVVVEEKLSTGAIIGIAIGA |
| Emiliania huxleyi | Haptophyta | NCBI\|<br>XP_005789<br>335 | MTTTVPVSLLLMFLNAAVGMRRLSSSAVLINEVTNQGSETDLCAGED-<br>WVELVNVVGAPSSVAGMALDDSSSSPDLGIVSDLPAEVAAGKALVLGQGSC-<br>PAEIPAHGYLVVCKGEKAFTFDAAGAVVASYLEGCGFGFGNGNTDDVVLFSSAEG-<br>NVTADMIVDRTGVWNDALGNNPTAAQSLGRNGAGDSAVLINEVTNQGSE-<br>TDLCAGEDWVELVNVVGAPSSVAGMALDDSSSSPDLGIVSDLPAEVAAG-<br>KALVLGQGSCPAEIPANGYLVVCKGEKAFTFDAAGAVVASYLEGCGFGF-<br>GNGNSDDVVLFSSAEGNVTADMIVDRTGVWNDALGNNPTAAQSLGRNGA-<br>GDSAVLINEVTNQGSETDLCAGEDWVELVNVVGAPSSVAGMALDDSSSS-<br>PDLGIVSDLPAEVAAGKALVLGQGSCLAEIPAHGYLVVCKGEKAFTFDAAG-<br>AVVASYLEGCGFGFGNGNTDDVVLFSSAEGNVTADMIVDRTGVWNDAL-<br>GNNPTAAQSLGRNGAGDSAVLINEVTNQGSETDLCAGEDWVELVNVVG-<br>APSSVAGMALDDSSSSPDLGIVSDLPAEVAAGKALVLGQGSCPAEIPAHG-<br>YLVVCKGEKAFTFDAAGAVVASYLEGCGFGFGNGNTDDVVLFSSAEGNV-<br>TADMIVDRTGVWNDALGNNPTAAQSLGRINWAESAVFVVFDERTLGVAN-<br>PFFVEQWDLSESSKAEDLSGVAIIPGGAGDIDSIIFCNNNPLNSDVAKGDE-<br>KLVRLFEYTLPTPSSPPKLMRMVRMDGFDDVEGLALVEKTSTGVRLVVTE-<br>EGRLNLVLITLPLMGAADGVVVEATVDYAAVPPEDMYAVSLVDDAKSTNR-<br>GLEGVAYDPAGFFYTLTEELPMRVLRVDMRTRETVLLFSGPPRLASGELRI-<br>QSHSSSIRNDEQALLSGVATDIAGMTYDYKSRSLIFVSHEGIDDGSIGPAT-<br>VFTSDLSGKLLSGPLKLQGAQPEGIVMKDASTMYIIGEPAEVQMYVSKKA-<br>GVLASPPPPSSPLRGDCPPGLEVACRVWTVNPAFA |
| Dictyo-<br>chophycea<br>e sp. | Strameno-<br>piles | MMETSP\|<br>CAMN-<br>T_0037720<br>169 | MSSSGMHRAFQRSMIFLFPERTVVEDELPGWFLRTILAPMGYTAPDIIWVHEISL-<br>DRGFIVIMNGSSWEVDLTGFHVCNEERRQRFDFPDGFVLPANGRVKIYCGVTA-<br>PLSPSRSAAEVELANGACLAPLTLYWEHERHEPVLNRHGDAIYVWDRYARIVTA-<br>LAKCGDGASKRYTPSRLERLHNSFQNLGLLVGLLRLAFVAGGCVFGLWPLLGHL-<br>ASAQARATPFSPNTPESAVSGGFDALAFGSLNLEAVLVCWVVAGGLDLAERLCA-<br>ERCGPLCPPLAHSVALLGDRLNAFQLYGSLALAYDGNASLAFLGLGLLDFTTVWF-<br>LARPSSSVSPTSTHDESAATTTAAAATSTAVVKPPKFLLALALSAPHGLPALCLAS-<br>EGFLVLCLVSHPVLWPTLSTAPTPPLLWSGVASRLLAAFCAARHLLQVLQLQVALA-<br>TLLGGGGTAGAASYPAGGGGRGASASSYLSGSRGSSPSSPSSPSRRLGDHYSD-<br>APAPVGSEMWAIGALVKKGRKTLTSFDGRVLSATAPVKHDSSRGGRGRAPTPAA-<br>AAALPRRQGGLDRTSQSHVTHGASIASSQSPGSSTSNVESEGDESSSEGDGND-<br>DDDEGMADVQTKPAGQSSVRRRGKRPNRSSSPVRPNLKTMRATRLTSLN |
| Dictyo-<br>chophycea<br>e sp. | Strameno-<br>piles | MMETSP\|<br>CAMN-<br>T_0037655<br>291 | VCCCWQDPMMAGQTPMRRSTTDQSEGDLPASLDAFQTGRLTRTIEVPYYERQR-<br>RILPFQATWLGSLFQKKHGVRIYDIDLQEDWVLIENFSNSPVSMGAYTLCDEEREN-<br>KLEFPVDLVLQPGRYLKVICAPGRTKNLDVFEEEDGRTSLFLWKTPRGAPRSE-<br>HVLREDGDCMYLCDLSNEVVSAFSKTDDGAFKEYGALNQAFEQKLWRGLGLM-<br>FVFARIALLGTAGYCAIHTNLSSESPSAHPFKVVGCMVGAVVLDLLQREALLYAH-<br>LHDFFGEALAIFADRLHAMVIYGTLALFHGDYCALFLLFLTLDLASCWFQLFASD-<br>CGELEMKAVAGAIAQRAPSLLTLIGLASEAFLLLLYMDHGHVQFRLYQGIQPAAA-<br>AIGWSFFQSPVLTLPVIKDVLLMAFLTKQIIVLSQLWFCMGSLMNTTFPCVGDDA-<br>KTYLQHALESRQRPNLELLRNSGSLQMQPSE |
| Tricho-<br>sphaerium<br>sp. | Amoebozoa | MMETSP\|<br>CAMN-<br>T_0008558<br>515 | TKKAKIDNKTCPIALHKLDLKDDVVTLINKTTKEVSLSGWSLKSQSGGQEYSF-<br>PDDATLKPGCTLSVWSGAKNSNKAKGGKSSLFWTKSYVWKDSGDACVLY-<br>NAEGEPVDEVEVTKEREPPSIAIQKLDADSQCISIINRGGEDVDIGGYFLRTMRL-<br>HTFTFPKKTVLKAGDVTTVWSGTKHKTKNVPPSSFCWKSHDGWTDTDVAVLYD-<br>NHCVLVHKVTEMIHLGELTERNVRPKYTSEPGEYPECIIL |
| Stereomyxa ramosa | Amoebozoa | MMETSP\|<br>CAMN-<br>T_0015333<br>301 | MQAEKRIAKRKSKDNTQEGGATKKAKIDSAVSILSLDLIADSVTLLNSGLEEVDVSG-<br>WIIDSKVGDQSYEIPAGTVIGPGATLTVWSGAKNKGKDNPPNSFFWTAKYI-<br>WNNKGDSVVLKNADGDEVDDKEEKPEIEEEPAVAIHSLDLKRECAVIENQSNT-<br>DQDISGWFLNSVVGDQTFLFPENTILKAGQSIVIWSGPESDQKVGPFSFSWTRRY-<br>IWNDNGDTAALYNKAGNLVSKRVEFPNVTPSHVEPPTKTKG |

<table>
<tr><td>Tannerella sp.</td><td>Bacteria</td><td>Uniprot| tr|<br>W2CEI4</td><td>MASPKGAKGLPETDYVELYNTTDHTISLKGWSFIYDGTVIRLPDVPLAANRYAV-<br>LYRKGKSLPLNKKVLGLGFSNFPLNMSDEGKQLTLKDSSGTVIHSYTYPKAKAGR-<br>SIERGEGDKWHLSSDPRGGTPGEENSVAPSVPTPKPDDPATSPDTPSAPPSSPS-<br>DTVKSGDIVINEVMASPKGAKGLPETDYVELYNTTDHTISLKGWSFIYDGTVIR-<br>LPDVPLAANHYAVLYRKGKSLPLDKKVLGLGFSNFPLNMSDEGKRLTLKDSSG-<br>TVIHSYTYPKAKAGRSIERGEGDKWHLSTDPRGGTPGEENSEGAPDKPERE-<br>KSSPGDVLINEVMADPHGLTKLPATEYVELHNTTDHEINLEGWAFVYDKTSIPL-<br>PDAELSAGGYAVLYKAGREISVADGAAEVAVKRFPANMINAGKPLALKDPSGTV-<br>IHSYTYPKAKAGRSIERGEGDKWHLSTNPRGGTPGEENSEGAPDKPDKPEKE-<br>KSSPGDVLINEVMADSRGLTKLPATEYVELHNTTEHEINLEGWAFVYDKTSIPL-<br>PDAELPAGGYAVLYKAGREISVADGAAEVAVKRFPANMVNAGKPLTLKDPSGTV-<br>IYSYAYPKAKAGRSIERGEGDKWHLSSDPRGGTPGEENSEGAPDKPDEPEKE-<br>KSSPGDVLINEVMADPRGLTKLPATEYVELHNTTDHEINLEGWAFVYDKTSIPL-<br>PDAELPAGGYAVLYKAGREISVADGAAEVAVKRFPANMINAGKPLALKDPSGTV-<br>IHSYTYPKAKAGRSIERGEGDKWHLSSDPRGGTPGEENSESTPDEPEKPKAS-<br>PGDVLINEVMADPHGLTKLPATEYVELHNTTDHEIDLEGWAFVYDKTSIPLPDA-<br>ELPAGGYAVLYKAGREISVADGAAEVAVKRFPANMINAGKPLALKDPSGTVIHS-<br>YTYPKAKAGRSIERGEGYKWHLSSDPRGGTPGEENSEGTPDRPDESEKPKA-<br>SPGDVLINEVMADPRGLTKLPATEYVELHNTTDHEINLEGWTFVYDKTSIPLPD-<br>TELSAGGYAVLYKAGREISVADGAAEVAVKRFPANMINAGKPLALKDPSGTVIH-<br>SYTYPKAKAGRSIERGEGDKWHLSTNPRGGTPGEENSEGAPDKPDEPEKEK-<br>SSPGDVLINEVMADPRGLTKLPTTEYVELHNTTDHEIDLEGWAFVYDKTSIPLP-<br>DAELPAGGYAVLYKAGREISIADGAAEVAVKRFPVNMVNAGKPLALKDPSGTMI-<br>HSYAYPKAKAGRSIERGEGDKWHLSTDPRGGTPGEENSEGALDKPDEPKVF-<br>SPGDVLINEVMADPRGLTKLPATEYVELHNTTDHEINLEGWTFVYDKTSIPLPD-<br>AELPAGGYAVLYKAGREISVGEGAAEVVVKRFPANMVNAGKPLALKDPSGTVI-<br>HSYTYPKAKAGRSIERGEGDTWHLSSDPRGGTPGEENSEGAPDKPDEPNKP-<br>NEPNATEQVEPREIVLNEILFDPQPRGSEYIELYNRSDRTLSTHGLAIALRKSD-<br>GHLGTRHSLTSLATTLAPGDYLVLTSDPNGVTSLIRTPALDVIRRFKLPALNNQGA-<br>TIVLLRTADSTVVDEVTYSAKWHSSAVKIRRGVALERISPDGSSQEAANWTSA-<br>SSETGYGTPGYKNSQSGTSSQIEEGATISEPEYNASTRDYLIRYRMDKPDYR-<br>CQMAVYSSNGQKVAVIANNQLLTPEGEIRWDGAGLTPGVYIFYVELYHPDGSSQHI-<br>RKPLLVH</td></tr>
<tr><td>Vibrio nigri-<br>pulchritudo</td><td>Bacteria</td><td>Uniprot| tr|<br>U4KER2</td><td>MALTIQYIQEQLKTLLEIRLATALTKQQLDELWEDLNRIQETSQVDIGELDFKGQT-<br>DHLDEYIDIVNRGGLAIDLTSWKILAGSPDQEFEFPEGSVLAPYGKIRVATSGDSEFS-<br>FQSDKPIWNNHGDTATLLNPHGQSVSTLAYGGDAYPDVLIT-<br>NIYFDGEEKHTEGDEYVEISNVSDNTVDIGAWRVESVRNQTTFTFPEGIRLQAQSS-<br>VKVFTNKEHLSEGEFSFCSPRAIWNNEQGHCKLFDYLDHEVGSYQY</td></tr>
<tr><td>Microcystis<br>aeruginosa</td><td>Bacteria</td><td>Uniprot| tr|<br>I4IMT5</td><td>MTKSTITGGNELKTRGVSDSRSNFYLVDLNVPISGKGVLNSWEIWADKVT-<br>PVQLVIYRKTANAWSVVGKSDLKTPALGLNQFALTTAIAVQPNDFAGVYYPQT-<br>GSVSFNRKTEQEAWDLGNLSGSVLFTSTGAAETAFSGSSNRVYSLKVHGID-<br>DAPVFVPIEKPANGRIIVHNDEWALSDAGFQSVSDTATFALNVATYFVGTTQGK-<br>FHVLSNNFGLVGATLERTMTGAGHTWTKGMNIAINAVALAQYDAIFVGGDPVD-<br>NQVLIDYVKNGGKVYLCAGTGHGGSQAEAGQWNTFLTAFGLKFDGVYNKISG-<br>SLAVTSPSHPLFSNVKTLYQNNGNSISLLPNATANIVMTGSNQGLIATADYIKPN-<br>APARSEPTPSPATSEPPKPKTIETEEFSITAEDDVFMSNLVYKGLVKKTQSDEY-<br>VELSNRGNHPVNISGWKITSVGSARQQFTFPANTVLASGKTFRVYTNEVHSE-<br>TGGFSFASKTAIWNDAGDELNLYDAMGKKISTLAYGIARTKKS</td></tr>
<tr><td>Pseudo-<br>altero-<br>monas ma-<br>rina</td><td>Bacteria</td><td>NCBI|<br>WP_010556<br>046</td><td>MTIKPLLFYSLITSTTFLYGCGGTEQTTDSSTEQTDSSSTITSTGLVINEIVASPSDT-<br>TYDWIELYATEDIADLSVFSLVDDNADREPQALPAVALSQGEFIVIQAIDETDTP-<br>PDNGYYVTFKLGSDDAVTLYEDGTAISVLDWEEGEAAEGFSYGLYTDGTG-<br>TAQTLTPTEGAANQTANTSTLVTTLIAEDAPLRINEVVAKDSSGGEDWIELYVTSS-<br>SDVYLADYTLSDDNNEQFALPDITLSPGEFYRIYASTDDLGDLPSVAFKLGASDT-<br>VSLYSNNVIIEQLSWNKGQALSGYSYGRYPDGSDATAVLTPTELSSNSQATHGPL-<br>VINEIVASAADDGNDWFELYNNSANTINLANYQVIDESDDIDPVTLPDIDLYAGEYI-<br>TIYATDEDPGTYYVPFKLGKEDELSLILNDEVIDYIDWDESDVATGFSYGLSSSTG-<br>FTHAFLTPTPSSENTVATAFTPTAVNTLSITITDENWQNILDNPLDEEYHETAITFN-<br>GVTLDSVAIRTKGGSSLRNVANSSSDRYSFKVDINEYVSGQKFFGLKKFTLQNS-<br>FNDPSYMREVIAYELMDEMGVPTPEHAYVNFYVNGELFGLYLMVEAVDGEFVE-<br>KHFANSNGDLYKPDGTGSDLLWLGDDIQSYTDINLQTNEDTTDNGAFINFVESL-<br>DKGETSAIEVDTLLRYMSVSTSLSNLDSYHGPLAHNYYIYDDDGVFSILPWDFN-<br>ESFGTFAMDCNGVDVRELYIDEPVSGALSERPLIANVFAEQSNLDTYHSYLTQLI-<br>NGSLSSDTFNARVNEIADLIREHVQNDPTSFYGSTYFEQNLTSTTGQFYGLTSF-<br>MQYRVANMAAQLDGTLPSADDGSGFCAR</td></tr>
<tr><td>Dorea<br>formici-<br>generans</td><td>Bacteria</td><td>NCBI|<br>WP_005339<br>862</td><td>MKRAVRMLCTFLSATMLVPLQVLGADAVSKPQVEQPSIVINEVESDAPNKGND-<br>WVEITNIGTEAVDISGWYLTDDKGEERKTEGKTTPLAKGTILEPGAFLVLEETKNFD-<br>FGFGKVDTAILYDAASTEIDSYAYTSVAAGTWSRQTDGDFSDVEATPGTANAAP-<br>TAPEPSKPEQTEPSAKPSLVLNEINSSPDDWMELMNTGAETLDISGFELRDNSD-<br>DHRWRFPDGSTIDAGKLMVVDAKSNGLVYDDQTKSFADGTFEEAIGIGSGDSIR-<br>LYDKGGKLLDEYSWTEHASYDGDPAKASYGRYPDGTGEFRLTQETKGTANAYY-<br>APTVVINEVESNGDTTDWVEIMNIGTQAVDISGWYLLDNDPVGHKGDVTPVADG-<br>TTLEPGALYVFDQNKDFTFGLGKDDKAVIYDAGGSIVAEYAWEAHANGVYARIP-<br>DGTGEFQDFATATKNKKNKVMNPVVINEVQSNDPSGGPDWVELANPTADELSI-<br>SGLVLKDNKDKDPYTIPEGTTIPANGYLVIYQNDSGATGFGFGLGKGDSVRLFE-<br>DGEQIAATTWPDGSHTNPTWGLYPDVNGNSYRNTLEATPGAANKFAGIPDVIA-<br>WPGSDQVRIFDTTPTFLEDSSGLDFANGKLYAVDNGTATFWVMDVAKDGTLTFA-<br>DGFEQGKRVCFQKDSDNEKAKGPDAEGITVDGSDMVYLASERDNSSKGVNYD-<br>TILMVNPSESGTRLVARKQWDLTATLPQVSANMGVEAVEWVANSDVNGKLIDQ-<br>NTGSAFDAAKYPNAVAGGVFFVALEDNGHVYAYILNEDETVVQIADIDSKLGGA-<br>MALDYDTYEKKLWVAADDGYGNRMAQITLNGTENPEIVHVLPAAGVDTTANNEG-<br>FAIADASYTVNGQRPAYRFCDGVTSGALTIGSINCRYCGTEQPDPSEPIDPGKP-<br>DNPKPSEPTRPSTPTEKPTTPSKRPAVPSAGTSVSTEKTTNVPKTGDSSTMGVL-<br>ITLAVLSVAVCFATLLILRNQKKKH</td></tr>
<tr><td>Alistipes sp.</td><td>Bacteria</td><td>NCBI|<br>CCZ98558</td><td>MLVINEVYTYADGSEKDDLDFIELYNAGGSDIDLGGLKLWESGGSAEAWSFPEGSR-<br>VAAGGFFVVVCDKDNAWYADPVNYPGWGLSKGPDEYVTLADAEMNEIDC-<br>VACPSMKRGESYGRVTDGAPEWQIFAAFTKGTPNEGPARQPVTNT-<br>MGLWVNEVFTNNQDTAQLPWNESVDFIEFYNSSDTAIDFGGYVIKDDKGAD-<br>DESYTVPAGTVIPAGGFLTYDVCKKNAEGPSFGLGKSGDWVFVYDPAGVLVA-<br>ELEIPAFADDEVMSYGRMPDGGDVLRKMEPTKNSSNGGEVHPEVAVVINEIL-<br>TNGSQDEDWVEFYNGGSADADLEGYVLYDDGGVEKAFTFPAGTVVPAGGY-<br>LVMVAKEEGSFDFGLGKKGDALTLLDASGAVTDEVEIPALDDDETYGRRT-<br>DGAAEWTVFGTSTRGASNAGGTVRE</td></tr>
</table>

| | | | |
|---|---|---|---|
| Micro-bac-<br>terium sp. | Bacteria | NCBI\|<br>WP_029266<br>970 | MPRLHRAAAVTAVCALSAAALLATPIAAIGADPGIRPSVATSTAPSLVLNEIVYDDAAT-<br>GLADQVEIYNAGTEVVDLAGWKIADEKRDTFGAAPDGTSLAPGEFLVLVKDVD-<br>FAFGLGKGDEVVLFDPDGTEVDSYAYANTAPLSVWARCPDGTGAWAPATQVTP-<br>GAANDCTVAPVAGSIVINEVDSQPADWVEFHNPGTAALDISGYEIRDNSDDH-<br>RWQFLPGTQIGAGQFLVVEEGTVGVSGGVEAAFREPIGIGSADRIRLYDTSG-<br>AMIDDTLPWQGHAAIDGDFAAATLARCPDGVGSFVLAHPTPGATNSCVMPDV-<br>VINEIESNGDTTDWVEVVNTGSTAVDLSGWTVMDSDPVGHAGETTPLPAGT-<br>ILQPGGYFVFDQPANFVFGLGNGDTVSIRDANGNTVDEHVYAAHATGVLAR-<br>CADGTGDFVDIAVSTKGLRNACGNPVRINEVESDGGSPDDWVELVNPTSTAL-<br>DVSGIVVKDDDDTHAYAIPAGTLLGAGEYLVIERAQFGFGLGDGDAVRVFDG-<br>DLLVDETTWGAGHAATTWGRCPDTTGAFAVTAAPTKGTANVCPGEVAVSP-<br>WPGSAEVRVVDGIPTFLEDSSGLDVQETADGAFLWAVDNGEGRIWKLDAH-<br>ADGSVEKAEGWDAGKRVRFQKDAANPGAAGPDTEGITVDGDGFVYVASER-<br>DNSAKGVTQNVVLKVDPEASDGDLVAQQEWDLTALLPAVGANLGMEAVQW-<br>VPDAALAGKLFDDRTGAAYDPRDYAGHGDGLFFVAVEDNGHVYAFALGADG-<br>SATLVSEIAPGLTGVMALDYDTVRNTLWAVCDDGCQGRSAEITLNGTGQPSL-<br>VHYARPAGMPDINNEGFATAPASLSVDGQRPVWWFADGFASEALRTGTLPG-<br>VDDGTPGGETPPLPGTGLVDDNRHGLTVDPSVATRGQKVTITVGAGGAGT-<br>DVSVWMYSDPTRIASGTLTDAGTISVTVPADAPLGAHRIAVFDASGALLGWA-<br>DLRVAAGNGAGAGAGAGAGAGAGAGAGGLATTGAELPVAALALALMLLTAGA-<br>VAVRRKRTA |
| Verruco-<br>microbia<br>bacterium | Bacteria | NCBI\|<br>WP_038132<br>244 | MKLLAQTFLALLLITAFRAQAELVAHWPLDANAKDLLGNHDGAESGVIFGVEGAAN-<br>HTGTAAEFNGSSSTITVPFDSAMNPESFTLSMWVNADSTNGFASPVTSRD-<br>DTPTSVHGYLVYNDSSGNWNFWTGTGGPSGAWNQMSGGKVEIGSWTHIAVSYD-<br>DGSQTKKLYVNGSLAGSNSAPNLYDPNGPQSENLH-<br>IGSGADSGGAFFFDGLIDDVALWDHALTSVEIKDLMT-<br>NGVPGGPPSITVFEASPPFIDLGQDVTLSWEIQNATS-<br>VSISPNVGSVAADRGSIVVTPSKTTTYTITAIGESSPAAT-<br>SQITVGVDVESLDPIITEFLADNKTGLIAPDGNRSDW-<br>VELHNPNPFAIDIGGWHLSDDSTQLEKFTFPSGQIPA-<br>GDYKIFFADSSLEALNFKLAKTGDYLALSDPNGNLS-<br>SEFSPAYPAQFDDVSYGLSNSGTLTYLEPTPGVANG-<br>SSRSEIGPKVEGLTRNPLPPTPGEDLVISANITPRIGA-<br>IASSKLFYRVGFGTDRQLTMTKGADDQFTTTIPASAY-<br>EAGEMVRWYLIATTTSGESTREPPFPDLTESAEYFG-<br>TVISDPSISVDQPVMHWFVKNIGAADTRGGTRGSLY-<br>FEGQFYDNIFCRIRGQSTANWPKHKYKFDFYRGDH-<br>FRWKPEAKRVEEINVNSHYRDSYVRENTIFAFLNE-<br>AGAMAPETRYLWIKRNGSDMGLFTFVEQVDEEFLER-<br>RGINPTGSMYKAINVPATLSPTVNSSLYRKVLRKNEPY-<br>TELRELTSGINISNPNRFEFVADAVNVPNYINVMAA-<br>MCVPFNHDQLTKNYYVYHDLDRGEWFRIAWDGD-<br>QGLPTGRTNGNENWSSPLYGDALHTQELVGGNPN-<br>PIWQNHLHAAILDNPVTREMYMRRVRTLMDEYLAIPE-<br>TGPSTTILDRGARLRYLAPIDASLESSWHLPEFDDSD-<br>WLSGIAGLGYENNPGDYLGLIETRVKPSESLPNGTS-<br>VYQRFHFNVADSPSSDLVLRMRYDDGFVAYLNGKEI-<br>ARDNINGAVRYNSTASSHPDSQAINFVEFPLPGVSLIP-<br>GENVLAIQIINQSSGSSDLLCEPELVDRPGANGGYF-<br>EGLLEGFRNTIQRDVVVDQALWSGAGITNFNNGYN-<br>GVLNTSLPNRRNALFKTYGPSGSGLIPQEQSSGLVIN-<br>FGNIESNPDSGNQDEEFIELKNPNNEAVDLSGWNISG-<br>GVELSLPPGTVIPSNSSLYLSPDVHDFRLRPASPTGR-<br>EGHYVIGNYDGHLSNFTEILSLSDSDGSLISEVITEDQ-<br>PSDAQRFLVISEIMYHPGEGAGEEFIEIMNISKSVTLNL-<br>GGISFTDGINYTIPAGTLLAPGNRMVISASDFKNGTAL-<br>SNGGELLKLEDASRSTISEFRYDDQAPWPISPDGGG-<br>TSLILINPTLKPDPSLASNWRPSLTTGGNPGTTDAISF-<br>RGQANGDLNGNGISDLIDYALGNGGMTQMTGDSFTY-<br>LKRLGADDATFQIEASTNLINWTDASALLIEQSRTDQGDGTALIKSKLSPAVSGTRW-<br>FFRIRVTLNLLQN |
| Clostridium<br>stramini-<br>solvens | Bacteria | NCBI\|<br>GAE86954 | MKKVVSSTVVIAMLLAIVIPAYYFGAFAEETQTLFINEVMSSNESTIRDGDVTDP-<br>KHGSKGGAYSDWIEIYNSGSTDVDLTGYILEDSSAEWTFPQGIVPAGGYLLVWASD-<br>KNMVAQDGQLHTNFKLSASGETVTLKKPDGTVVDSVAVIGLGDDQSY-<br>GRIRDGAPEFVVFSKATPGAANVYDAPGTTTLFINEVMASNASTIRDGDVDDP-<br>KDGSKGGAYSDWIEIYNSGSVDVDLTGYILADSSAEWTFPQGVVPAGGYLLV-<br>WASDKNKVAADGQLHTNFKISSSGEALTLKDPDGNVIDMLVTINLLDDESYGR-<br>KSDGSSELALLRPTPGAANIYDPRLIPVSEPAFSHKGDFIPMRLI |
| Limno-<br>chorda pi-<br>losa | Bacteria | NCBI\|<br>BAS28019 | MLRSSALLFLAGVLALVGALNLGERQVGWDPALAAAGAPGSGNG-<br>PDDLVRAGVVEVIDGDTFVVAFAGTPSARARLVGVDAPESVHP-<br>TRGVEPYGLAAAAFLADLLRPGIELRLEFDVEVADQNGRLLVYAYLPDGRMVNEVL-<br>LEAGYAQIYTFPPNVRYVERFRAAQRAGREQGRGLWAIPAEGDGEGPGPRVV-<br>IASVDLEAEQVEIWNGTARALEMTGWRLISVQGGQEYEFPEGFVLGPGEAVV-<br>VTSGSAFQHQPPATLGWTRRSVWANRGDPAELVDPSGRVEAAFP |
| Desulfo-<br>sporosinus<br>orientis | Bacteria | NCBI\|<br>WP_014184<br>986 | MIMRKSRLFYVLVICITLLVTGCGNTSTTTATSTSSSQTSVDQGEELNNNQPTPM-<br>NAHGNLQVHFIDVGQADSILIKASDGTAVLIDGGNNPDGPNVVNYLKSQQVKE-<br>LAAVIATHPHEDHIGGLDTVINSFPVKAVYMPDASTTTKTFEDFISAVAAS-<br>GAKRIQAKVGVKLDVPGLTGQFIAPNGSGYEDLNNYSAVLRLTYGKVSFLFTGDA-<br>EEVSETEMLGSGQIVKADVLKVGHHGSSSSTSSAFLKAVSPKYAVISVGADNDYG-<br>HPTASTLSKLSGAGVKVFRTDLDGTIVATCDGESVTFNKKGSAPASVSITSIDLAGE-<br>IVTLVNDGSSAVDLTGWKLVSEKGNQTYSFPSGITIPAGGTLKVLSGSKAESSTGA-<br>LLWTKSDVWNNDGDPGSLYNAQGQVVSVK |
| Petrotoga<br>mobilis | Bacteria | NCBI\|<br>KUK80215 | MKKVLLIFCILLLPFSLSLSFNFPSFIWNYVSSYQTDEQIYWKVNYTQKLIK-<br>LIEDYLPFLEKSGISLPITSNVEVHFIDVGQGDSVYIKLPNNVDILIDGGN-<br>NWYGDDVVTYLKSQDVDDIEYLIATHPDADHIGGLDDVLKAFEVENVYAPDVSHT-<br>TKTYNDFVLEVRNEGTYIKTTKAGMTLINSALAAVIGCPSFSIVYFLGPVKNYGTDL-<br>NSWSAVMKLDFGNSSYLFTGDADRISEKDMIDNGMFLKADVLKVGHHGSYSSTS-<br>KEFLKEVSPKYAVISVGKNNNYGHPAQEILEGLEMYKVDLFRTDLQGHIIAISDGNK-<br>INFNVEPINTVNLMEDAQESAPQKTSILITNLDVSDEKVTICNNTDEDVDLTGWVLV-<br>SEVGNQKFNFPDGYVLRVGECVNILSGRNAIDEPPVNLKWTGVYIWNNDSDIAVL-<br>YDAEGTLISRLEF |
| Methano-<br>culleus<br>marisnigri | Archaea | NCBI\|<br>KUK63359 | MSSIPGYLITIALCTCIIACFITAGCSAPIAQPEIASGLSGDLVVHFID-<br>VGQGDSILIEFRDKTMLIDAGERGMGERVIAYLDERNVEELDVVVATHAHSDHIG-<br>GLGSVVSAYPVGRFVDAAQPHSTATYEDLLVLVEEQGIPYTAAERGQTIALDPDLEIL-<br>VLNPTPQPLGDINEDSVVLMVTYGEISYLFMGDAGNAAEESMMEA-<br>GLDLDADVLKVGHHASRYASSAEFLAAVSPAISVIPVGEGNDYGHPH-<br>EEAVERIEATGSRIYRTDLDGTVIVATDGRALTVAAGGAPSATVTARA-<br>TTSTPAATSSGVYITDLDIQDEWITVTNAETAAVNLTGWTITDEGTRN-<br>TYTFPLFTLSPGADVTVHSGPGNDTVSDLYWGRETPVWNNDGDLATLA-<br>DANGTVVSTLER |

| | | | |
|---|---|---|---|
| Methano-<br>culleus<br>marisnigri | Archaea | Uniprot\| tr\|<br>A3CW90 | MSSIPARYLICIALCACTVACLVTAGCSNPSGQQPEIGSDLSGDLVVHFID-<br>VGQGDSILIEFRDKTMLIDAGERGMGERVIAYLDERNVETLDVVVATHAHSDHIG-<br>GLSDVISAYPVGRFVDAAQPHPTATYENLLVLVEDLGIPYTAAERGQSVALD-<br>PDLEILILNPGAEPLGDINEDSVVLMVTYGEISYLFTGDAGTPAEESMA-<br>EAGLDLDADVLKVGHHASRYASSAEFLSSVSPAISVIEVGEGNDYGHP-<br>HEEAVERLEATGSRIYRTDLDGTVIVATDGTALTVAAGGAPAATVTAGAT-<br>TAAATATPTATATPAPSSGVYISDLGLQEEWIAVANADETAVNLTGWTIT-<br>DEGTRNTYTFPVYTLDPGADVTVHSGAGNDTATDLYWGRGSAVWNNDGDLA-<br>TLADANGTVVSTMER |
| Haloferax<br>volcanii | Archaea | NCBI\|<br>WP_004043<br>458 | MVRRATATLVVVVLAVLAGCLGGGGAGAATTVESTATETASPTAQSGSPT-<br>TAAAAGDVTVEVVEVVDGDTIKVVMPDGARETVRLLGVDTPEVHAENTPDE-<br>FEGVPETEAGRTCLRAAGEDASAYAKSRLADRTVELRYDEKAGERGYYGRL-<br>LAYVVVDGAEFNYDLITEGHARLYESSFEERERYERAERDARERGVGLWSCATEG-<br>AAAGGADATTDDGLSLSIVADAPGNDNDNLNEEYVTLRNDGDAAIDLSGWTVSDA-<br>AGATYTFAGGTELDPGASLTLHTGSGTDTDEDVYWGRGGAVWNNGGDTVTVRD-<br>ATGDEVLAYTYE |
| Methano-<br>methylo-<br>vorans hol-<br>landica | Archaea | NCBI\|<br>WP_015324<br>112 | MQIKLNRLICLSILLLFIVMTSGIGSADEVGVFDNGQWAVGNDPATATIFGFGWIN-<br>TKPMVGDWNGDGKEEVGVYNPSGKNFVIQDANGYKVIGLGWTGVIPVVGNWNG-<br>DRADEVGVYNTEGTWALWNTDTNSADIVGFG-<br>WVGTEPVVGDWDGDDVTEVGIYNRRGNNFLLQKGSGFDIIGLGWTGVTPVVGD-<br>WNGDGADEVGVYNNAGTWALWNTDTNSAEIVGFGWAGTEPIVGDWNGDGIFEVG-<br>IYNTAGNNFLIQTENGFDVIGLGWSRVTPVIGSWIDASTIYKPQDKDSPEQPPTQQT-<br>PTEQIPSPVQDSNLTVHFIDVGQGDSILLEYGGKTMLIDAGENNMGYTVSAFLRERK-<br>ISSLDYVVATHPHADHIGGMLTTLNNYPVGQFIDSGYPHTSKTYENMLMAIDAQNIL-<br>FHVAERGETINLAPGTEIRVLNPKKEQSEELNENSVVLKITHGDVSFLLMGDAGLEA-<br>EENIMAAGYDVDVDILKVGHHGSTSASGYEFISAVSPDISVIEVGAGNDYGHPHANI-<br>LQRLQGASTVYRTDYDGTITITTDGSRYTVTTEKTGSSGTSTTSTSEDAVYISDLDL-<br>RNEFVIITNPGSSTVDLTGWKIKDEGNKHTYTFPYFQLESGATVTIYTSKGLDSKTKL-<br>YWGSGNPFWNNDGDTASLYDSNGNLVDSLVG |
| NMCPs: | | | |
| Klebsormi-<br>dium flac-<br>cidum | Klebsormi-<br>diophyceae | http://<br>genome.mi-<br>crobedb.jp/<br>klebsormid-<br>ium \|<br>kfl00193_00<br>80 | MFDSPGTAILAEELHAREAARSIRVWTAVNESVGVSVDRREENTERALTAVNPS-<br>GEEEVWRRFRGAGALDEEVLVRREKEELQRQLDDVERELADYRYNL-<br>GVLILEHKKCKPQIDELEKALQRTREELQKEGRSLQLALDDVTRREDGLRASLKA-<br>ERTVIADLKQSLEEMHAKLQAEEKSSRERLARAQELEAGAVKDRERSEE-<br>LFEKAARDLKGAQQLEEGLKVRESELDGKLRGLAEREDDAASREKALNE-<br>GEAALNAGKRELTEKGIKLAERKRKVEGELEEERKKLGREREEEELRVEA-<br>LRRSVEGEREQMQKEMQAEKEAAEHDMSTRKAEISVKEDSLAAQEKAL-<br>QMSQDELERKQEDANKRDQDLDQREKLLGDEKREMLRTEADLREQQK-<br>SMNEERARLAEQESEWQRIEAKIDARVKEIDAREAAVQAFESTAESSAKE-<br>LEEGAKALRERQAGLEEKEEAVQKKEEDLGKLEKQLKGRAQMLESERA-<br>ALERRKGEDVLEREQAEKKAAQLEMEAAALLDKEAQLEEQTRQLASKDK-<br>DLAAREHELLLRESGTDENLRQQKEELEAQRRQAEADVAKQLQQLQGD-<br>RAKGEALLQASRAQLDKERAAMQLELQETRASLEIEFQTKMLTADAATQR-<br>AAKSEVEKRVKDLEREAEIRAEERATVRLQERLAGVDLEVERRVSAQLKE-<br>RVSELEVRNREEVERRVSEGSKKGVEREVKAQVKDAVKKAKEEAKAKYD-<br>QERGAAIQKAVRDAERKAEGEKRAAIDKAVKEERSRTAGQARAVSEAEG-<br>EKSSLQNGEGLEGVSQLTEGEQAGGPKEGVPRRSWFGLGSPRRGAQP-<br>SEARENEEGERQILVDGVPPLEQIDGLLSQVGMETPPVAVNPVGSSEAA-<br>GVEGASADEVAVHAEPTPVVSAGVNGAPSPKRPKLQSKRKQPETPPEEA-<br>PEERPLKQRRIIADAGPDKPVLEEKHGGPVEALGAAVTAVGRAADTITGP-<br>FGFVLDDTDLGFVPGHTDRKAPREVSVKKPRWSLWPGFLSPRKAKAEE-<br>GGQDGAGTSPSGEEGAEKNLPGGQVEGQSSPLLENARNDGGEVGPV-<br>LMDVQQGPLPTENETPPEMDLRGDETRAAEERATRVAKTPNTRVGRAVL-<br>PTPKPARTPKPPLSAVRTSSRLRERTLSSDLKGTLEPTSATQLAGSSGKR-<br>GKSSPPGADVGPDAQSQEPPKQTERVSPLTDGTRNQAPGKSAEIAGRE-<br>QRDPSPAKTPGTGPARRLQRALTAEEQAKIERIMARLEAEERAIAADEAAV-<br>GPLNARTSPDLAPEREFLNAGSADGAKTSVPEREAANADIVDGATVKGL-<br>EKVRGAPETGNSGLKKAKVAVKRRSGERAGASGKTDETLLIEAAAAKNQ-<br>ELPNDDPQEAPAPNPMAPGGASAAVDAAELPTVSPSPPGPTSTLPPKTP-<br>KSAFGRAFDGFRAAWGFGAGSSRGPASEGDEEGSLEGSGGVYRSQVG-<br>ELRSIVGDLVEAADRDEDMEMGGPERLEFGTPEGVPFDGPEGEERGR-<br>EAAQGGEGLLNTFTETVVEKSEAALREVVEKGQKAGSVIVKKAKTAAFDL-<br>NETAMELADAAVGVSASDVADDVTRSAADLADDVTALGGAVEATVGGVAD-<br>ELTGNIGEALADELGAVHTRDAQGKSLVETGSDLVTGALGSLQGAGAAG-<br>SSALAADPSRGAEGGARSTRGGAKVQSKKEGGNGEERRGSKRKAPES-<br>GKGKGKVTDSENEDDVGPKRGKVKRRVKQADSEDEAGLVDPRVVEFAT-<br>KFRGEVEGEDTPSEGGGKRHLRAVVDPRDGEDMSAGLRRYNLRKSTLV-<br>KMHIWEPRGADFGTVSTGSEGIHTRGGASSTPRNLQRSVFGGDNPFLED-<br>IAPGNPHSLEEIRTPRSGRHGARDDEKSGGAAGGTTADDEEADQANAGWRWLGF-<br>LGW |
| Klebsormi-<br>dium subtile | Klebsormi-<br>diophyceae | NCBI\|<br>JG442173 | GEAALKAGKREVIENGIELAGRKRKVEGELEEERKKLEWKRQEEELRIEAL-<br>RRSVEGEREQMRKEMQEEREAAERDLSARKADISTKEESLAAQEKALQASQEEL-<br>ERKHKNAIKRGQDLDQREKLLGDEKREMLRKDADLREQEKSMEEKRARLTEQE-<br>SEWRRIEAKIDARVKELDGREAAVQAKESAAESSTKELEEGGAALRERQAVL |
| Spirogyra<br>pratensis | Zygnemo-<br>phyceae | NCBI\| GB-<br>SM0102128<br>9 | MRNAFSPLRLRTQRDADENMSNYNRIGSESIPSSSIMPRHRAQEVIWTDAQG-<br>PVIDEVSVIRSERDALSIRLQELELEYGDYKLQTEAAFDERDRCLSQFS-<br>TIQSQLKEMVEKFKLEKNAHQSTIASNTEKERKLRDALTHTQRTADELELALTEKN-<br>LEFDNFRQSLTSLENETIRKKCIAEEKILECEISLKESFSLKGEYEKKKVEY-<br>ESKLQETQITESNLRLEYLRLKDSQNEFQIQVSLKEQALKEMEERSRKLI-<br>SEYEQMNEEMEAKMKTILEKEEEMESKEKELIHFENETKIERNELENLEK-<br>RLKEMQERLNEQEKLVIAKENSFSETKMHLTSFEEALNFRQESIELKLNEL-<br>ESKEKEMNEEKIKLLSLEENLKHKQVAVDEHKTALIKEINELQFQREEMEK-<br>EKLFLFDREKELNELNHTLRKQREGLNLERDALDSKESNLIDLERKLKQKE-<br>SMVEIKKESVEEKDNLTNVKMNSASKKMRELMEEKRQLQEDLSRLGEDT-<br>TVLQRERELVKLMRESMDAEYSVMNEEKERIEQSAEENERVRREAEKAL-<br>DEARREKEDVFRFKDMVEIEREELREEKRRFEHAYEELDAMKAILDEEKDEAKR-<br>MHEVRRRELEMQVMEINQMK |

| | | | |
|---|---|---|---|
| Nitella mirabilis | Charo-phyceae | NCBI\| JV767595 | MKSAKKASSMNGSASPKNPNPDNNMISAGSFEIRKMTTTTRTLRESASCEKKKK-<br>TVKEVEEKNAGHNFGGAFSREALALDLSGLPSDLKPIELGGAWRRFQDSGALSEQ-<br>GLEKKERNFMVDHIKQLEDELYKSQYHMGLLLMERKSLLADVAKLDHTAKEYEEI-<br>IKREREEWSMTRIEYEEIETTLKENLLAEQQCVKEVEKAIRDVQKEKDETTRLAELR-<br>FSEARALMSEVEKKEHGVEISRKNVEATRAEFTRKKVEIESRARELETKESLVRRE-<br>REQVRIESSELEMKRQELIERENVAREWEAKLASREQSVRKMEDRMKGREDTVL-<br>RKERDVEEGMARLDGKRKGLSEMEDDLRLREKDLAMKEESLVRREVNVVQQEQ-<br>AQQEIEERLSMKDEEMRSAKERLLESEHDVKLERERLKELEATLTRRQDIIEQQKQ-<br>QMSSKENALMERELSSASAVHNIKLEREDLERKDRDLRLLREEIEEQKALLNDSRR-<br>DAQQWEAKLEMRERSIEAKEESLRERQQDVELRGKGVLDKERELKMDRRRIEAIS-<br>KKIEEEREEVRLDMELAKKDREALANEREEVRKEIEDCRRLKVEADNVRADALAKR-<br>AEVKKEREDLARDREDFMRKREDMRKERDAVSKDREELKREKELLEQKRDQVIR-<br>ESENVDAEREEVEREREELDKEREEVKQLREENSKELEGLMKLKDDLNEKDRSLK-<br>IESRRVEIELRTVKEDRDAVRRESEELRKDKEKVQQLKIEVKNEKTELDIWKEEIETE-<br>RERMEREKHEVRRDRELLIRQEEVHVKLKIEIEESQRTFKMDKKKFELDHRALKEE-<br>RDDIKKEREELARERDLVDIERESLKKERDEVERDRLELELERERLEAKSELFEKEK-<br>EELETEREMVKKDMESLSRQESRYSSEVLQKETELSLEKRRWKEGLEENRVTRET-<br>LIREKEEFSREKEDAAKQRAENQREKEDLERDREELDRDRIELTVKRDELKRAREL-<br>HLRQEEELAKTMEDMLAKERMLKSERRKLESASAKLKEEREEVSGEANEVKRERE-<br>EVLRERESVRKEKDELLQDREDLERGKEDVRRERDEMRRERDVVRRERIQLLRE-<br>EETLSMDRDELMRERANTAKLELELTIQVEELKRQREELELVKERLCDQKAALSLQ-<br>NMELTKQGEEYRRKMDDLGRVEDTMRRRREAMIVEADAAVNHSGSLVATLLANVA-<br>HVEDGATAVLALPQALTASGEDALHGARSREERSLQERVSEGGREETDKISWRSE-<br>TRVSSKLREGRGSYFLRQHAAESENRRSSVRAQAIEDEERRKRKRGHSELSRTDL-<br>QKGESLVAVRSEEADEQANTDRRVDVSSAGRSGTPPSGADASPSRRRTRSMQDV-<br>INKGKEMWMTSTIGDEVDIDTIGSKGVGKLNSVQGKRRRKVRAMVDADEDTTTED-<br>DVGKEDSEVARKTEGKVKYNLRKTTVYHQATRVIESRDEQPSAGGAEMALQVAVT-<br>SAGLIPPSPAMGWKLTRSLGLKQQVPRRRPSEGEHTSSRDTSQETVALGARKSG-<br>GEEDASRGRSREEEEEEEEEEENNEDEGQDEEENEAEKDEGADAEEDEVVERGR-<br>ENSSWLEWLLPVPWNV |
| Nitella hyalina | Charo-phyceae | NCBI\| com-pound of:<br>HO490484,<br>HO531334, HO566387 | LFLMEKRSLLAETSKSDNTVEGYEEIIKRRREEWSIMDFEELETQLKDTYLKEQRD-<br>VEEVIKDXXXXXXXXXXXXXXXXXETKENLLRREREQVRILGADMETKRQELTERE-<br>NIAREWEAKLATREQGVKKTEERMKAREDTVLRKERDTEEGLAR-<br>LEGKRKGLSEMEDDLRLREKDLSTKEEGLXXXLNMKDEELRSAKERLLESEHDVK-<br>MERERLKELEAMLARRQEIIDQQKQQLSSRENAVTERELSSASAAHNMKLDREDIE-<br>RKERDLRILREEIEEQKALLNDSRRKHSSGKRSVELRERNXXXXXXXXXXXXXXXXX-<br>XXXXXXXXXXXXXXXXXXXXXXXFLRQHAAESENRRSTVRTTVTEEEARKRKRGHS-<br>ELSRTDLQRGESVAVAGSAELEEQGNADRRVDGVSSAGRSGTPPSGTDASPSRR-<br>RTRSMQDVINKGNEIWGASLVQGDEMEAQAVKGAGKxxSYIPKRRRKGQAMVDA-<br>XDADEDTTTDDDTGKDDTEVARKIDRSVKYNLRKSTVYHQAARFSEQNRDAQAYE-<br>GCGVLTRQVAGTSDRLVPPSPVMGRKLDSLVGLKQQASWRQPSERELT |
| Coleo-chaete or-bicularis | Coleo-chaeto-phyceae | NCBI\| GB-SL01031242 | MFQHQRKTPGRPSTGIFRNGPISQSPLPGIPLQGEAPSAREVATPVGR-<br>RRLEGEGHEDEAGVWQRFYEVGALDEESIEQKLKEDLMN-<br>QLQSKENELKRYQFEMGKVLLENTNLRDELVAAELAAKDRELRLEAEQNEHREM-<br>VAQMRTREQQLHKSVEAERRCTADLERAVREMQNESMEAKKKAEVQMEESM-<br>ALWKEAERKRQEAEAQVRSAQALHREAEQKMGEIRRREEDVAAREREVNREH-<br>AKRLAEFEAEKAEVDRRQREVASWDASLKECDGKLKEAQQRLRQLEKSLEERS-<br>ASILSNEKALEHRDSVLRAREENAKYKEEEVKAWEIERRAWIEALEEKLAVQQL-<br>NTEKWAAEVGARERAVVEAGDRLAVAEADVTQRSGELVQAQAEMAREHERLT-<br>ELSTRLLSDEAGLASQILKHDSDWEQRHSHLRTLESEIEKKLAEAERAKESAVA-<br>REESLTVALSDEQRRRAEITAARSACEAAQAQLQEKEENLRKREEAVERKSEN-<br>MDKKLEKVKEKERDLGERERALRAQKKAVELDRQSLEEAGRASMKDKADIEDL-<br>LEELEGKRAEFALEVDGLQTQKHALEEDKAALERENAEKLAGLQGELEKLKEEG-<br>EELAKIRQNLEAEREELKAERTRFEDKWVVLDQREKELAEMEERLAGEVKSER-<br>LQLQEERVGMEQAVRDAEARATEAERAAAARVEQVEAAAKMEMEKERVAERE-<br>LMDRQRAAELLKLEESGEKYRKDLEVEFATKLEEARLQMLQETVAEADKRMDK-<br>AMEEKAVEIDALRTQLANVEVMLKEKVEALERAEAALEEGRHEVREMRAEMAK-<br>KVAEAEEEKRVAANAEGLAVVERKELEKEREALEEQRRKVMAEAHAEAAVIVAT-<br>KVVEVQKNLESARMALLKEVEDLKRQAKEDLDKEREVLACARASAQEMKVREN-<br>AVETEHAKVKAALEKLRFERSKLVALKEELQAHQAQQLAGAVATGEIDGMHLPN-<br>VDAEAVYLHGGEPDAALLTPLAASQPMDAVGEKERAMDVAGKRAEGSVERFN-<br>QMRSPPLFSPGGTINWLQQCTRGFFTPKKNGDEQSPAEPEAQADAVDDNHNE-<br>EDQRMMVSCPVQGRSGSSGSLRRKTTIEVTVAEAALFLGSGGTASTHDGLPQ-<br>EQGHAEAETASGCKERQDVRPIPRTRTISETIEEGRRFLGLDAAAADDEDDDD-<br>MRVSMEMVMGPSVGAPLETVEEVDEEGEGAEQLGIELSAADLVAVRESGMPM-<br>EGERRGEDSDAITSAAKSALRVTKRTRSMRDVVDEGRKFVGISTDATDADDTTQ-<br>IPLSGRTRRSSKPTAAASMDNALELEASGPVSSAAAEGPIGNRLRRSNRPADHI-<br>KGGIDTDSETPSMQPVGVGVSVNRRSRRSNRPTEHTKAGPDTDSEAPSILQPG-<br>EESAQGSRRLRQRRHSMADYSKGNTDSESEGMRPRKSRRFSTIGPSETPAMS-<br>ADTATAHGSRYNLRKSKRFVWMDGCVIAGRHRSSGHTDVESESEATSAHAEATS-<br>PPKALD |
| Selaginella moellen-dorffii | Embryo-phyta | NCBI\| XP_002993584 | MFTPHRRGATPNRGAGFSVSTERREVRFASSSPPDGRQQHQSADGTLAGN-<br>GAGDGAGKSSSEIWQTFREAGALDQESLELKDRNALLAHISKLETELY-<br>DYQYQMGLLLLESNKLRGESERLKSVIDETRDGLKREQSAHMIALQEAERRED-<br>SLKRAVTTEKKCVADLEKALKEMHEEVAEAKAAAATQFQQGKATAMSAEEKLLE-<br>AESKLHSAEALLAKANRKHADAERKLQEVESREDALRRQRHSFLAECGAHKLE-<br>LEHEKQNLKGWERTLEESQARFVENEKLLNKREEYMQQRDDALTKLERDLDE-<br>ARKVLEKDRSALRQEQAEYSALLSALSLREEAAVERENAATKKEQEILLLQEKLA-<br>SRDRAFEQHEQMVRELEQANAKEKERLVDLEASLSTRENLLAVSKQSLVN-<br>IVFYVPHALICFCMVGGYLEDPG |

| | | | |
|---|---|---|---|
| Physcomi-<br>trella<br>patens 1 | Embryo-<br>phyta | jgi\|Phy-<br>pa1_1\|<br>169197\|es-<br>tExt_fge-<br>nesh1_pg.C<br>_2000036 | MILSSGFSSGMLQELASAGLTGYRLVEYLSRGLFLVFREADGTSNLLLKFPLVLQFY-<br>CRIGVLSFESIDVPQEFLPCSTMALHSPSEYVPLSAYRSATLSAGTSVHFNC-<br>PLDIRNWTTAHKIVGIFLYLKERSPRYLRWRKVEAEELVKGRRGSPQQRVTP-<br>TRSLAREKGKTPPITNSTIGALTTTTTTLAAMGEEAMALMDPLMGSPDMIG-<br>VADAVPETEVWKRFQNEGALDMPSLERKDRAALHARIAALEAELYDYQY-<br>NMGLLLLQRKTWTSQADDLKAAVADAQETLQREKAAHLLELSEVMRREE-<br>AAKKALETEKQCVADLEKALKEFQTDESEVRQAADKQLAQARELVASIEE-<br>RSVQADLKLAQVQVLRADANRKLQESELRLQEVEAREVALRRERHSLM-<br>ADVDARKEQVASEEASLKEWEKRLEEGRARLQEGERLLNERENSLKQR-<br>DEALKQTSRELAETRSYIENERALIKQTDADLNARVISLSERERTLSERELK-<br>ILTKEQDLLLAEERIAEQTREFENRELQVKETKEYVEQERARLDDYESALK-<br>FQETTLEEQKMELSEMEALLKIHTSDVDSKKAELLAAEEELRSVRKTLAAE-<br>KEEVETLKLAAEAREARSRHLETAITAREEELKLRVQEIVDREDVLNRRLE-<br>EVSNLEQGIRVEEKKYENEHERIAELKEEIRKSKEEMEENKLKLELQKQLI-<br>EEEREHLRRECELERQEIEEEREKVRKDWEEEREEWEQQRLIVQKDVEY-<br>KKEQLEFEKERLREELKAEREKQSAELERMRVNLHNELEALREKLKAEV-<br>EFERDALRKEIETDQERVAELREESRRAIQAEREQVDEERSRIRRELEVE-<br>RQQLAEESERAHAAIDLERRKIEDEQEKLKALEQERGELVRIQVQLKQEID-<br>EIRARKQFVDEEALELKLQKDRFEREWELLDEKREATRKERERFEEES-<br>KRMAEWMQDEEERLKETRRQVQEQSRRMTEELQKERESWESRLETER-<br>NQLYTQLDVERQALNRNLELQREDLDRRLELERDAFEKQFEEREAQLRA-<br>EVEQEKEDLRKNRGSVIGELEQLRAERAKLEKERQELLKQRVDAEKEWSE-<br>IKKDIEQLQLQGEKLREQRESLHLERQNTMREAERLQKLREQMKGSEG-<br>SMSMRVPEQPMRMEEEVVSPHPQGLLVRTDTQRAVGGRPAPGTHKPSS-<br>SHISSRRMIARTPSRLAWLQRCASRASLLFSSPTKLLTGQEPVEEEAEEE-<br>QVKQDPNAPSSSFNQSQLGQVEGNIDDGPRFRRTRSIQRVVEEANAILGI-<br>GVEETSESNRNRSNADAFTTTPAESAETRQKKRARGNVDDDDANPLEA-<br>DAHGGTQRKKRIKDIMVEIETNEDSLHTPHSRVSTPATKRYNFRPSTIVNM-<br>TAASENVSPRHHDRTSKKAASATSQPTAAAVDDLPEVISQPVQETEAMHE-<br>APAVGETLEAQEEGDAVAESPHSGETTTVRVDHVTETQVVTETTTIVTETV-<br>KELAVFDLNVEELDMAEIEAEEVVPTAEAVHLSRSVELPESGEETAGDSP-<br>MQAGEVDVGVTLDDSCDDEVEDVDAMEGNDEVDEDGEAAENGDNGVA-<br>VPENGELLSEEESEAEGEEASEAEVEEESEAESEAVVEEKSEAEVEEDV-<br>GEVEDDDENEDTREDDPEDEPDDEGEKPSIRAKIWDFLTT |
| Physcomi-<br>trella<br>patens 2 | Embryo-<br>phyta | jgi\|Phy-<br>pa1_1\|<br>233301\|es-<br>tExt_fge-<br>nesh2_pg.C<br>_760029 | MSGLSPMYTPQGMRGSPHQRETPIRSLAREKGKTSPATATTVGVVTTTMTTLT-<br>AGGEEGLVLMNPLTGAPDTNGEADGVPDTDVWKRFQSEGALDISSLERKDRAAL-<br>HARIAALEAEVKIATRTPSVLELMHDKCYESSSFTLGSSRTLGGQRLCKDDAAILY-<br>DYQYNMGLLLLQRKTWSSQVDELKAAVADAQGTLQREKAAHLLELTEVIRREE-<br>AAKSALETEKQCVADLEKALKEIQADESEVRQAADKQLAQARELVASIEERSIQA-<br>DLKLAQVQVVRADANRKLQESEHRLQEVEAREVALRLERHSLIADVEARKEQV-<br>ESEEASLREWEKRLEDGRMRLQEGERLLNERENSLKERDEALKQINREVAEAR-<br>SYIEKERVLIQKSDVDLNARAVAFSEKERALSERELEILKKDQDLILAEERIADKTR-<br>EFETREQQVRETEVYFGQERTRLSDFETALKFREESLEEQKHELAEMEKFLKS-<br>HTSDVDSKKAELLTAEEELRSVRKVLAAEKEEVETLKLVAESREARLRHLEAAIT-<br>AREEELELRVQEVVDREKVLERRLEEVSNLEQGIRFEEKKYENEHQRIAELKEEI-<br>RKAKQEMEENKRKLELQKQQIEEEREHLRRECELERQEIEEEREKVRKDWEEE-<br>REEWEQQRLLVQKDVEYKKEQLELEQERLRGELKAERERQGAELENLRVNLH-<br>NNLEVLREKLKAEVEYERDALRKEIEADQERVAGLREESRKAIEAEREQVDEGR-<br>SRIRRELELERQQLIEESERAHAAIYLERQKFEDEQEKLKALEQEREELVRIQVQ-<br>LKQEIDEIRARKQFVDEEAQELKQQKDRFEREWELLDEKREATRKERERFEEE-<br>FKRVTEWMRDEEERLKETRREFQEQSRRMTEELQKERISWESRLETERNQLY-<br>AQLDAERQELNRNLERQREDLDRRLELESEAFAKQFEEREAQLRAEVEQEKED-<br>LRKNRGSVIGELEQLRAERSKLEKERQELLKQRADAEKEWDEIKKDIHQLQVQG-<br>EKLREQRQSLHIERQNTLLETERLQKLRDQMKGSEGSMSMRVSEQPMRMDEE-<br>VVSPHSHGLVRTDTLRTSALPFVLGTHHASSSQTPSRRMIARTPSRLAWIQRCA-<br>SRASQLFLSPNKLLTGQEPILEEKTDDEGEPRLGANDPCSSFNQSQLGQVLDT-<br>TEDSHRFKRTWSTQRVVEEANTIPGLQEEKNFESRNRSNVVTFSTPDASGDTR-<br>KKKRARGNVDDEVPLLQSQPDAEDQGGTKRKKRIKDIMVESETNGDSLIDTPR-<br>SRVGTPATKRYNFRPTTIVNMMGASENESSRHHDHSNKKAASAANQPAAASVD-<br>RLPDASSQPVQDTEIDMHEAPTVEEGFEAREQNNSVAEDDQERDTTAAVADHF-<br>PITQVVTETTTTVTETIREQAVFDLNIGVENVEIARTVPEQEGIPTAGEVYLSRSV-<br>WLPGEAEEATAGDSLVQADEADARDAQNQSDRSDEEVAEVTDDSAEEVDEDG-<br>NGTESGENGDAASVGGESLSEEESGAEEVEEEDVGEVEDEDEYDTR-<br>EDEPDDEGPTPTIREKIWDFLTT |
| Pohlia nu-<br>tans | Embryo-<br>phyta | NCBI\| com-<br>pound of:<br>GACA01014689,<br>GACA01016482,<br>GACA01042116,<br>GACA01016308,<br>GACA01016301,<br>GACA01041377,<br>GACA01007203,<br>GACA01001674 | EGRQMSGLSPMYTPQGRRGSPQQRGTPLRALAREKGKASPNTTTPVGAVTTTVA-<br>VATAEEAMALMDPLLGSPDMNGAGDGTHTESEVWKRFQTEGALDMPSLERK-<br>DRAALHARIAALEAELYDYQYNMGLLLLQRKTWTSQVDELKA-<br>AVVDAQETLQREKAAHLLELSEVMGREEAAKNALETEKECVADLEKALKEVQAD-<br>ESEVRQAADKQLAQARELVASIEERSVQADLKLAQVQVVRADANRKLQESEHKL-<br>QEVEAREVALRRERHSLMADVGARKEQVEAEEASLREWEKRLEEGRVRLPDGE-<br>RLLNEREDALKMRDEALQQTSRELAETRSFLEKERALIQQSDVDLNARVVSLSE-<br>RDRALSERDLEVLKKEQDLILFEERIADKTREFETREQQVKETEEYVVQERARLN-<br>DFESALKLREETLEEQKIELAEMERLLKSHTSDVDSKKAELMAAEEELRSVRKTL-<br>AAEKEEVESLTLAAEAREARLRHLEAAITAREAEFELRVQEIVDREQVLERRLEEV-<br>SNLEQGIRVEEKKYENEHHRIAELKEEIRKAKEELEENKMERGELVRIQVQLKQEID-<br>EIRARKQFVDEEAQELKQQKDRFEREWELLDEKREATRKERERFEEESKRMAE-<br>WMHDEEERLKETRREAQEQSRRATDELQKERESWMSRLEIERNQLYSQLDSER-<br>QELVHNLESQRADLDRRLEQERDAFVKLFEEKEAQLRAEVDQEKEDLRMNRGSV-<br>IAELEQLRAERAKFEKERQELLKQRTDAEKEWSEIKKDIEQLQVQGEKLREQRQS-<br>LHLERQNTLRESERLQKLRDQLKGSEGSMSMRVSELPMRIEEEVVSPHHQGLLA-<br>RTETQRTTPGLPPPLTFRASSSHTQPSRRMIARTPSRLAWLQRCASRASQLLGS-<br>PVRLLTGQEPVLEENAEEEELEPKQDANAPSSSFNQSQLGQVVGTTEDGPKFKR-<br>TRSIKRVVEEANAFTGRHGEEIEISESNRNNDDFTTPPAANADTRRKKRGRGNVE-<br>DDDPKPEAEIHGSTQRKKRIKDILVENETNGDSPADTPHGRALTPAAKRYNFRPTT-<br>IVSMMAASENESPRHHHSTSKRAASATSQPVAIDELPEVSSQPNKEMETEEHGA-<br>PAVGEASDAQVGAVAENTEVVEAT |
| Amborella<br>trichopoda<br>2 | Embryo-<br>phyta | NCBI\|<br>XP_006855<br>781 | MLSPRKKENSGTLKSPGSRVSLQGSPSIVNGDEALWTRLREVGLDEETLKQRD-<br>KAALISYITKLESEMFDYQYHMGLLILEKKEWTSKYEQIKASADSAED-<br>KYKRDQAALLSALAEAEQREENLQRALGVEKECVASIEKALHEMRAECAETK-<br>VAAETKLAEVRCLVEDAQKKLLAVETKQHTVEALQTETSWQ-<br>HAVAERKLKEVEAREDELRRQQVSLKSEMEAKEKDLLNEKE-<br>SLRELEKVIQQGQEKLFEGQTLLNQREQCIKERSDRLSRLEK-<br>EVQAATVKLQEDLEILKEEKANLCLTSVALTTREEAIVQREVSI-<br>DKKEQELLLLQEKLTSREQDEIRRLTIEHQTAIELRESQFEE-<br>ELHEKHKSFEADLGLQRHALDLRDAELKHQEDLMHKDKHE-<br>LDLQLSELEEKRKELETGLKSLVEKEQSLDAREKKIEMERNC-<br>LEKENQELDVIKKELDVYRNSLENERKQILEEQRKLEVMNN-<br>DRKDLLALETKLKEEVDNLRAEKVKILAEADNLATEKEKFE-<br>KEWEQIDEKREQLQKEAEWVAEERMELSKFLKTEHEILNL-<br>EKDSLREQAKRDADSLCREREAFLSEMEHGHSEWFTRIQ-<br>RERADFVHDIEMQKREFQKGVDKRNEEIQRYLRERDDTFQL-<br>ERLREFQYIDAQKELVRKELEGISLEMKKLENERKNIALDRE-<br>QRDKEWSELKKDIEELQVQREKLKEQRELLHQDREDILKRI-<br>EDLKKLEDLKVPSETLMLPEMQSTGLNLNEVKTPANYLVGP-<br>CATKAAVEVHADECNENANIGAKSELLEQKESDSDVPTPKS-<br>WLKRCAEKLFNTSLEKIVVASNNNYETHFSRHKETGQGPL-<br>SFSLRQKSHRTDARRVKTFSLSRSTRPVPDEKNAVLEGPL-<br>VREEKDHLQEFDAETNVSAKNGSCNIKSSVFDSERAQTS-<br>ANNGGECEFLYGRKRSRGYTSIEDADAQFSRKQSKRQQQ-<br>APTAEHPRMGTSAELLVHSPVVHPEGANGLKPCSHIPDVR-<br>EVVDGGPSNGPAKVRGEEGEASGIVLEGLNNSSKKDALES-<br>QTSDIEGEKAYEVATQPHDNGVLAAKFDEQIGANSSSPEVTG-<br>WTFVMPALFIFAIEAIKVLLTNISLWHRFACADGNLIDVPSGAVALPF |

| | | | |
|---|---|---|---|
| Amborella trichopoda 1 | Embryo-phyta | NCBI\| XP_006849769 | MFTPQQQQPTRKAGGIWPFSPNSNKGGPVTRLASPDPPNITGILLHGGD-<br>KGRAKGKGVSSSFPLQEEEQATPPPPPRASLLSDENGRPRPYSSSASEVWRHF-<br>REAGSLDLDSLQKKEKDALLSHLAKLEDELFDYQYNMGLLLIEKKEWTSKYD-<br>DLKQALVEAQESLKREQASHLIALSEVEKREENLRKALGVEKQCVADLENALHEM-<br>RTEFAEIKFTADKKLAEARSLVASIEEKSLEAEAKLRSADAQLAEASRKSSNLERQ-<br>LQEIETRESVLRRERQSLKAEREAHETTFNRERENLRNWERKLKEGQERLVESQ-<br>GLLNQREELANEKEMFLTKKEKDLEVAWAKFEKGNLNLKDKEVEMNMRLRSLTA-<br>QEEEAAVRKRNLDEAQQELHLLQEKLNAREKEGIQKLLDEHNAVLELRKREFDIE-<br>MDQKRKSLEEEFEKKQVVVEQKLVEVDLKEEKINKKEQLLEKRTEKTKEKEKDLE-<br>LKTKSLKEKEKFLKIEQKDLDTDKKKMVIEKADLHSLKLELERIKAAVEEEKEKIVK-<br>EQENLKVTEDDKRELLRLQSELKQEIDEFRLQKLAVEKEREDLKLDKEKFEREWE-<br>VLDVKRDEVNKEVELHNVEKDEFLKRKCEEELKLKREEQKTSEKFQREYEALEL-<br>QKNSFTENMNHERSVILQNARRERDDMIREFELQKNALESSIQNRREDMEKQFL-<br>EKERDFQEVRERMWKEIEAQRELAQKEMEEMKLERTKLGRERQEVALSKKHVE-<br>GERLEIQKDVEQLHILTTKLKEQREELRRERDRILSRIEHLKRGQGDSIDVTDGLAL-<br>SELQSFKEFENNGGNLLPRLLDGYMKESMQGRSNVGPSNLMEETPPLGAVLNS-<br>TSPARFSWLQKCKSIFKLSPGKRLDEQVTNQEKSPSDVEADADQILENDSGGLV-<br>SGGANYDEPEISVGIQISQAVDFHRRAASPESIGRGDEEETVVTPSAADGTQSDM-<br>LEMQEGPSASAEISHPSAAAGGRARKKPRRGAPKLTRRTRSVKDVVKESKAILG-<br>ESSEELKTEEEEESAQANVDSKGQPIVKKGGRKRQHPTTSRTMSEQQQDADSQ-<br>SESVTRGRSKRRQIEPSHIQPPGGRRYNLRHSTLEKHVENPVGSQALASKVTTD-<br>ADENHSQHVTKSPGEVVEGQTSNHIHPDEPSIESLENAHGGGEAKTDVRMLQHT-<br>KFESIVEIHREFSTQKVIMIETGGALEETDVNDPGPNSSEQEPQANQGANDLLEY-<br>DEDGGGSGSRGGEDDDGNDDDDDGYVNDENQEASIGKKLWTFFTT |
| Musa acuminata 1 | Embryo-phyta | Uniprot\| tr\| M0RRY8 | MVLIKGDRFESNRTGASFPTTRRPDRRKIACIKGDGVDKRYWIVGSPIW-<br>DREALPFVLVFVLKFLDEELGFLMFTPQRKGWSPSPRYGDGVDNRMTTPAVNTRT-<br>GSGVAFLKGKGKSAVEALPPPPPLQALLGENGSIGVVDQGDAEVWRSF-<br>REAGLLDESSLQRKDRDALVQRISELEKELHEYQYNMGLLLIEKKDWASKYEEI-<br>RQALAEVDETLKKEKSACLASISEFAKREENLQKALGVEQQCVSDLEKALREMR-<br>SELAEVKFTSDKKLDDAHALEIGLEEKYLEVEQKLHAADAKLAEASRKSSDVDR-<br>KLEDVEAREHKLQKEYLLFDSGRKLHEKDITEQREHLRDWEQKLQDSQKRLVE-<br>TQRYLNEREDRTNEADRVLKKKEADAEEARKMIEATKKSLKTKEEEITKRLGSL-<br>AAKEKEVDVKVESLENKEKDLISREEKLNARERVEIQKLLDDHNLLISSKKEEFEL-<br>DLEKRRKSLSKEIECKIREVEKKRREIDSMEEQITKREQALQMNLQKLMDKEKD-<br>VDLKSNDLKKWEESVQNDEKKLEKERQQLASDSEEFLKSKSDLESLKAAIESRK-<br>EQIMKEEENLRLTKGEREEHLLLQSNLKQESEDCRILKESLLRDTEDLQQQREK-<br>FEEEWEVLDEKRLALEAERKKFNDEREKFEKWRHDEEERLNNEALVARANFER-<br>ELEELNQKTEAFGEIMEHERLEALEVLKRERADMARELELCKHELEMDMQKRQ-<br>EDTEKKLLDKENDFQRKRDLDFNQMISLSSSNDLKIQKLKMEEDRLEREKEDLS-<br>SYRKRLEIDRLEIQKDIDALRMLSRNLKEQREEFMKEKERFLAQAEQKTCKNCG-<br>LLVGDLDTFCIQDAGDVQLPNLGFEEHLNDTNAETTNAKVSPAASGGRMSWLQ-<br>KCSRLFNLSPGKKVLDSSQHPLDNSNLYSSLDREAFDGEASHKPAASYGVVDS-<br>SDSQRAQSVTGIGDNVESKRLCGVVEEPEPSFEVANNSIHIMRTQTQMDNGVR-<br>DVVDQLAMPSVSLNDREKYAPAGSDNLRVSFKQRQSQPGRRGRPKAVKRTHTI-<br>KAVVKDAKAILEQSSDEKNHGPHNGEAKDPRRVAQTSGVTNSDPDAEDSEAHS-<br>ESISLGGHRKRRQILASAVPVEKRYNFRRSTIAATTTAAQTMSDQTKGFKAGYD-<br>RQLTGNEILKEIGGEGSSRPAVEPVSDVVNSIIASNMLQKTAAVGIAEVREISSQK-<br>IVQAESNDDTVKSVEVSYQSGEDGHILDDAATGSRPATPSDDEDEDEEECEQQNA-<br>SVGRKLWTFFTT |
| Musa acuminata 2 | Embryo-phyta | Uniprot\| tr\| M0TQE3 | MFTPQKKGWPGWSPSPRVGDGVDNGMTTPVVNTRSGSVLAFLKGKGKGKGN-<br>NTAEALPLPLPLQASLGENGDTVVVGGGDAEVWRNFREAGLLDE-<br>SALQNKDREALVQRILALEKELHEYQYNMGLLLIEKKDWALKYEEIRQALMD-<br>VEETLKREKLAHLASISEFEKREENLQKALGVEQQCVSDLEKALREMHS-<br>ELAEVKFTSDKKLDDAHALEAGLEEKYLEVEQKLHSADAKLAEASRKSS-<br>VANRKLEDVEAREHKLQKEYLSLSSEWKLHEKGITEQREHLCYWEKKL-<br>QDSQKRLVESQRFLNQREYQANEADRFHKKKEAELEESRKMIEATKKSL-<br>KSKEEDITIKLRSIAAKEKEIDVKIESLGKKEKDLFSREETLNARERVEIQK-<br>LLDDHNALLISKREEFELNLEKRRKSFDADLEGKVHEVEEKKREIDCME-<br>DQVKKREQALEINLQKLMDKEKELDSKSKASKKWEESVKNDERKLEKD-<br>RQHLASECEELLKCNSELESLKAAIESSKKQIINEEENLRLTKVEREDHL-<br>LLQSNLKQEILDCRLMKELLLRDTEDLQLQRKKFEEEWEVLDEKRLALEA-<br>EIKKFNDEREKVEKWQCHEKERLNSEALIAKANFERELEELSQKEEALE-<br>KAMEHERLEAFELLKREHADMDRELELRKHELQMDMQKMQGMEKKL-<br>LDKENEFQRTRDLELSQMISLSSLNDSKSKRLKMEEDRLEREKEDILSH-<br>RKRLEVEQLEIEKDIDALCMLSRNLKEQREEFMKEKEHFLDQAEQKTC-<br>KNCGHPLGDMGTYCILDAGNVLLPNLVFEERSNNMNAKSSPNAMVSV-<br>PAASGGRMSWLQKCSRLFSPGKKTSCKPVSFHGVADFSYRQENKEPK-<br>RLGEAGEEPEPSLEVADNSIDIMRTWMDNGAREVVDDYVMPSFAQNER-<br>ENFAPAESDTLPESLKQRRSQPRRRGRPKAVKRTGTTKAVVTDVKAIL-<br>GKSSNEKNHGSQDLVLANSTTSAGQKRCVAQISGMTTSDLNLGDSEAH-<br>SESISLGGRHKKRQILAPAAQIPGEKRYNFRHSAIAAVTTAAQTIFERTK-<br>GPKAGGHEDSTGNEIPMQSGGEEGSARPVVEPVSDVDSKKASNMLQK-<br>TAVESTTEVHEIFPNKIVQAESNDDVKSVEHSDQSEDGFVVDDAATGTD-<br>PATPSNGGCSEDDEDEEEYDQLNASIGKKLWTFFTR |
| Musa acuminata 3 | Embryo-phyta | Uniprot\| tr\| M0T5I3 | MFTPQNKGWSLSPRIRGGADDGSGSTANPRGGLGGLASTKGKGKSVVEAAPP-<br>PQALLGDDGEDAFGGSTEVEAWRRFREAGLLDQSVLQRKDREALVQRITELEKEL-<br>HEYQYNMGLLLIEKKESIARYEEVRQALAEAEEILKREQTAHLI-<br>AISEYEKREETWLKDLGVEKQKVSALEKDLREVRFEISEVKFSSE-<br>RKLSEAHALETGLEEKYLEIEARMHAADAKLAEAGRRNSETNRK-<br>LEDIEAHERKLQRDCLSLTSERKAHEKDLLEQREHLFDWEKRLQ-<br>ESQRRLVEEQRLLNEREDSANEADHILKKKETELEETREAIEASKR-<br>SLKLEEDDITIRLSSLASKEKEAEIKMGSLERKERELFAREEKLNS-<br>RERVEIQKLLDDHNAMLDSKKHEFELEMENQRKSFEEEMKAKID-<br>EVEEIKKELDHKEEQILEREHALEINMQKLKEMEKNLESKSQALK-<br>RWEESVQIYEKKLEEDKQQLDRDRADIVKSISELESLKVTIEAAKE-<br>QIIKEEEKLRLTKEEREEHNLQKSKLKQEIEDYMIMKDSLCRDS-<br>EDLRQQREKFEEEWQLLDEKQLALELETKQINDERVRFGKWQY-<br>DEEERIRNEEKAKRISIATELEDLRMKKQAFEKTMEHERLNVHEM-<br>LTRERSAVAREFELRKDELEMDMRKRQEAMEKDLQDRESEFQR-<br>KMTIELDEIRSVSSDFELKSRNLEMEQDRLEREKEDLSAFRESLK-<br>TDQLEIQKDIDTLRVLSRELKDQREKFVEERDRFLGLANQFKIC-<br>KNCGSSVCNLDLLGLQNTDVVQLPSLTFEDRLEAKDSETSPRH-<br>MVSPSVSSGGRLSWLRKCSGFFSFSPKGSEDTAQNQVKNPISL-<br>DVRLAREALDGEASDEPAPSQGIFAKSFDTQRTQSDSGIRDNE-<br>VSKRLGRAREELESSFGLSVPPRNESQPEPSNEKPRQPKRSGR-<br>PRKISRTRTVKAVVEEAQAILGETSMGKNGQPNGLAKRSLNIQE-<br>STEGNLVHAGQKRGLTHISVAAASELDGEDSETRSESISLGGRR-<br>KRRQINIPETQTPGEKRYNFRHSTIAAAARSISDQTKGHKRGG-<br>HQQPSGDESLRGDGDGEGTSKLRLDVEPASSFAAESLKSVDM-<br>QKMAAENVLDVQEIFQKPVSHEIEECHADDAGKSVEFSKQTGI-<br>EGVMADGATAVEREPATPDDGCSEDDDSDEAEENSDDQNESS-<br>IAPMEKTEQEALYMVIQGFVGQRWNGSDLYPDPCGWTQIQGVS-<br>CDLFDGLWYVTALSIGPILENSLECTEKSEFTNLGQLRNLRSLVL-<br>VDNSLVGELPMELGNLIQLKRLMLSGNRFSGQIPASLCINLNQLLI-<br>LDLGGNSLTGSLPSSLCGLSSLLKLDINSNRLHGSLPPGLGNLSH-<br>LALLDLRNNSLSGVPSKSLAGMESLQVLLLSYNPWGGSLLEFEW-<br>KNLRNLTTLDLSHMGLEGTIPETIASLKRLRYLALDNNHLSGIVSSK-<br>FAALPSLTALYLNGNNLTGELEFPERFYRRMGKRFASWNNPNL-<br>CCNAAAMATGSAPHGVAQCKQDQEPSANGSNANERVDDRNPD-<br>QNSGLSTSFLFPASSISGFWWGIVVQEIRTWVLPLEL |

| | | | |
|---|---|---|---|
| Musa acuminata 4 | Embryo-phyta | Uniprot\| tr\| M0SIR4 | MLAPQKKGLFLSPRAVAARRNGPAQSSFGNWGGVVPGTRK-<br>GREVVAGNAIPQPEELPLCGDGEDREKEQSEAQVWRRFREAGFLDEAVLQR-<br>RDREALVRRISELEKELYQYQYHMGLLLIEKKEWAVKYDKLRQEM-<br>SEAAQLQKCMQAAHIVAVAEFEKSEGNLRRAMGFQRQSIIHLEKALNDMHAEIA-<br>EVKLDSQKKLSEAHTLEATIEEKCLEIKEKQHSLDARLAKVSRKSSEVDRRLEDV-<br>EAREHELFKQTSSFIAEKKAFEKDLSRQRENLRAWEQQLQDNQKKLGKWHST-<br>ENQREMETNERDNTFRKKEKELEEARKTLEISNELIKLKEEDMCMRIGALDAKE-<br>KEALLKQEFLEKKENELLAIEEQLNNKERVEIQKITDFHNSILESQKDEFELETEK-<br>KKRAVDEQLQGRIEEVAHKEIILENRERELFKKEQLLEREIGNLKNREKENDIMLS-<br>AVKVSIENEKEEMRQGRGKLEKEWELLGERRLSLEEGLKQLFDEKERFDQWR-<br>CTEEERLRKENPEVSIHAQMDLEDSISDEEAFKDKTTHQKMDVLEVFNSENAH-<br>VVYEIMQRIPEKVKETLLEKEDNSNRRSNIVLNNCKILSSLDESNILKLKEQEDQL-<br>KSEKQLLVLGKKSEAGQSTSGTLSRNNKDQVVEPAEEGDYLPASAEQLKACRY-<br>CGFEDGGDTALSGGSVEVSDQGTCPGSVKLEARIPCMQRCSRLLNFSPGKKA-<br>TEHSEKSVCLDGEPLEHEDNLEPGPLPGDVNAFQWAQSAGGVQYNAEPERS-<br>NNDDDATKRDSQIADRSADILIFELNDRVRDLEEPTLHSVDEQKYREGCSIRPEL-<br>NSLLWPLKQKQSGRSVRRKSILVKKSRSVNALVEDANLEEASQIKHSEQSTCRA-<br>QCLIKDKCLEEKYSLNDDEVTVCSKKRCLDLKYGMMSLEGECAEAHTEDVSSL-<br>GCCFQMENIPGTEIPGLKRYNLRHSTIVRAVAASQALACRTKQKRKGELELSLE-<br>NKVLKVARRDGEGAESHASASETLRSDIRNS |
| Musa acuminata 5 | Embryo-phyta | Uniprot\| tr\| M0TBW8 | MTSPRPGGTPASRAVNASKTPATGGTPLGDDAIWKRLKESGLDEESVKRRDKAAL-<br>ISYITKLESEIYEYQHHMGLLILERKELVSKYEQVKASSD-<br>SAEIAYKREEAKRSSALAEARKRELNLEKLLGIQKECVANIEKALHDNLVESAERKL-<br>GYESKIAEAHAMMTAAQEKLDEAEKKLLAAESLQAEANRTRNTAIRTLDDVEARED-<br>ELRRRLATFKSQCDAKENEISIQRQALYESQKTLHQQQERFLEGQTLLNQREEYIF-<br>ERTKELNRIEKELEESKANIEEESRTLKLERSNLDLEIAALRNREEVIVKRESMLDKR-<br>ERELLILQEKIACREHDEIQRIMEEHQSILEKKKSELEADIEQRHLLLKNELEAKKIACE-<br>IREADLCSREISLQEKEHAIELQSSVLAKKQEDVANKLRLLEDKEHNLSSTKREAEIE-<br>VQNMQKEREIFLKMKVDLEKTKAVLEDEKKEIILAEEKFEITLGERNELLLLENKLKEE-<br>IDSLRAQKLALVAEADILKAEKEKFEIEWEMIDEKREDLQKEAERIDEERKTLAQYLK-<br>NEHDSIKLEKENLHNQFKRDVERLSCEREEFICEMDRQHSDWFTKMQQERENFT-<br>KDIGIQRNELENSINERREEIETYLREKEESFEKDKVKELQLINSQKDMIAKQLEHVA-<br>SEMQKLNTERLEIAQDREQREREWADIKRFTEALDLQCEKLQKQRELLHAEREEIN-<br>QKIQQLKKLEELQIESENRALSVMQTDKCDASVGKSCQCINGADRHIATPNGVSTM-<br>KLLPQGTPNPSTPTSVTKSWIKKCTEAMFKHSPEKDSDTGHEENVESKMLAKSRD-<br>FRFSEMDLQGHGNFAEGKEVSVQEMDNFTPKRTKSNRQEKVNGQEIKCVRCNFD-<br>EQNMISDARPVAKSAQSPSEVGANSIKFNQALEDSGQKSRTLFSSINSWISRRKRS-<br>NDMLSHDHADMDSEPNPKQQKRPRQNGNSDVEGDSSNGLAEQQPNIDDECEPV-<br>LRNQTSGCEQLHAVAFKDQQHENMVVPNAEPIESSQHKLAVSNFDIVENGNFCKF-<br>EHSPLAGVGAATSSDANEISMKDKQVFDKEHIARKPSQETSVSASDLIVEDNDKLK-<br>EQDRYNEVLDELEDEDDGSGLSVKEKIWNFLIT |
| Oryza sati-va 1 | Embryo-phyta | NCBI\| BAC78596 | MFTPQGKGWTGWSTPAPANQRSGGGAPAASAPLGKGKGTTLRVAELEQEL-<br>HEYQYNMGLLLIEKKEWTAKLDEINQALTQKEEILKREQAAHLNAISEYERREESM-<br>RKALGVEKQCVTDLEKALREIRGEIAEVKFMSEKKITDAQSLEASLEEKRLEIEGKL-<br>HAADAKLAEANRKKSQADRDLEEVEARQRRLEKEKLYFENERKAGEDRIK-<br>RQEDSLRDWDKKLKESQNRILDLQRSLNDREERANENDKLFKIKQEELEE-<br>AKKALEHTKATLKIKEDDINKRLAELHLQEKEAESKNRKLEEREKKIAEREE-<br>KVSAREKVGLQKLLEDHNVKLESKRRDFDLQLENEKKSFDAMLVQKEADL-<br>VQREKDVRSSEEKLSKKEQVLNESKKKLEEWQNDLDTKSNALKKWEESL-<br>QNDEKQLSEQKLQIENERKQAEMYKLELESLKATVVAEKEKILQEQNNLKL-<br>TEEERQEHIMLTAQLKKEIDEYRMRSNSLSEETEDLRKQRQKFEEEWEQL-<br>DEKRTHLEEEAKKLNNEKKNLERWHDNEEKRLKDREDELDIKYKEQGENL-<br>ALKEKSLIDNIDHQRLENEELLKRERADLQRNLQLHRHELEMEMEKKQASK-<br>ERELEEKENELNRKMDFVENELKRAAELNESKIQKILLEKKQLQKEKEVLV-<br>EDRQKLETDKADIRRDIDSLNTLSKSLKERREAYNRDRNNLIDIFEKYKVCKN-<br>CGVIIFEGLDALALKDSTDIEYPSLAVEADDRSPNPDTLAQETGALVNSGGR-<br>LSLLQKCSRIFKFSPRKKAEQSSEQQAVKNTDFGARLEEASQSDDDYEPT-<br>PVYQVAYNSFDAEDLPSESGAFENEESERQDIADDVQMESSLGVADNCVD-<br>IHGTQSFDGNTDMVVDTTIVDVDQNGKDSAVLPVVDLEPETSKQGRRQQ-<br>NRKGRAKGGVKRTRSVLAVVEDAKEILGENLEVKKDDGQGDSVTVGGTR-<br>KRRFAGATISEQDEDSEAHSESVSLGGQRRKRRQTAAAVTQAPGEKRYNL-<br>RRTTVANAATAAQTNKKKAAKKGSKQTVEATADDTEGTSKAEEPATGSKG-<br>ASQSADDASQLPEYSQAEAGDTHGPVEVTSAEGVDIVDGIDAAPDAMPMT-<br>PSGSELGAEQDDEEDDDSERRNQSIGKKLWSFFTT |
| Oryza sati-va 2 | Embryo-phyta | NCBI\| BAC78597 | MASPRSAGGVGGGGGGGGGSGGAAAGDDAIWSKLREAGFDEESLKRRDKAALI-<br>AYISRLESEIYQYQHNLGLVLMERKELTSKHEQLRAASESAEIMHKR-<br>ERAAQQSALAEARKKEENLKKSLGIQKECVANLEKALHDMRGE-<br>TAETKVSYESKLAEALQLMEAAHKKFDEAEEKLLLAKSLEAESIRTHNAALRSLH-<br>DIDDREDQLRRDRISCELENEAKEKEISLQRKSLNDMKKILHEKEEVLLKEQALL-<br>NQRDENILERLAYVTHSEKRVEEEKNILEAERKVLLEEKYKLELKMEAIVSREEAL-<br>IQKESLLDKRESELLILQETIASKERAEIERLNQEQAIALERRKHDFESEMANKQ-<br>MSFDAAMEVTRNALHQRECALSEQESVVVQRSQNLDLQLAELASKEKALAGR-<br>SDELKEEEEKLLLHREAIHNELQKEREEIQRIKSDLEKEKAFFEEEKREAIQAQQD-<br>LAITQADRDELLTLQMKLKEEIDSLRAQKRELMADADRLQAEKERFEIEWELIDE-<br>KKEELQKEAIRIAEERRAITEYLKNESDIIKQEKDNLRVQFKSNSETLSREHKEF-<br>MSKMQQEHASWLSKIQQERQDLKRDIDIQRVELLNSAKARQMEIDSYLREREE-<br>EFEQKKAKELEHINSQKEMINTKLEHVAVELQKLKDERKEATLERERREQELSEIK-<br>GTIEALNNQREKLQEQRKLLHSDREAITVQIQQLNVLEELKIDSENKQLSLLQHD-<br>KSKLGSDINVKDNHHDNSHSSPKQRFGRKLDLSPVSTPISWVRKCAQVIFKRSP-<br>EKSASHDQFVQNGVPKKVGDSVDVEDVNLDFAKVGQKRLNHLVSCDQTEVLE-<br>PKRKHRRSTIQKVNGGEITSNCLSALEEKCSKNEHDEAPLGLSNTCKEHEYGD-<br>KGPENLTKPGEPASSVDVPYVNGIVDNSDSVQEEPSVEATVSATETSNVDGPE-<br>DNNDSDEEDEEEEEEKTSSAKKLWRFLIT |
| Daucus carota 1 | Embryo-phyta | NCBI\| BAA20407 | MGRVEDMGLNAKLMKLETELFDYQYNMGLLLIEKKEWTSKFEELQQVYTETK-<br>DALKQEQEAHLIAISDAEKREENLTKALGVEKQCVLDLEKALRDMRSDYAEIKFTSD-<br>SKLAEASALITKVEEKSLEVESKLHSADAKLAELSRKGSDIERKSHELEARESALR-<br>RERLALNAEREALTDNISRQREDLREWERKLQEDEERLAEVRRLLNQREERA-<br>NENDRLYQQKQSELDGEQKKIEIIMVSLKNKEDDISSRIAKLNIKEKEADAVKHS-<br>LEVKEKDLTEFEQKLNAREQSEIQKLLDEHKAILEVKKQSFEMEMDKRKNDFE-<br>NDLQNRAVEVEKKEVEVKHLEAKLAKREHALDQKHEKLKEKEQYLASKLQDL-<br>NEREKSMKLEENKIEDERNQLLSDKQEMLCLKAEIEKDRASTEEQRLKLSEEIE-<br>RLKITEEERLELARLQSELKQEIENCRHQRELLLKEEDELKQEKMRFEKEWED-<br>LDERRTALMKDLKDITVQKENFEKLKHSEEDRLNNKKLDTESYVQKELDALRL-<br>TKDSFAATMEHEKAVLAERTSSEKKQMLNDFELWKRELETKLFNEREDMENA-<br>LRLREKQFDEEREKELNNINYIKEVISKEREDIKLERSRIAKEKQEILMHQKHLD-<br>EQHVVMQKDIGQLVSLSEKLKDQREQFFKERECFIRFVESQKSCKNCGEMTS-<br>EFVVSDLQSLAELENLKALSVPQLAENYLRQDLQGTPDKNLSTVTPGAVGLGS-<br>PASGGTKSWLQKCTSKIFIFSASKKNNSPDQNTSRRLHVEASPNKLLNTEVIP-<br>ELPSGVAGETLEMQNMQVSNSNREMESNLNLSGTEQSNIDSKALDVEDSQQ-<br>SDVRAGNRKPGKRAKGRVRRKRSAKEVAEEAKTVLADPIELNENEHSNGLAS-<br>AYTNESRGDSSLVGKRTRNSRKRNPSQPSQSAAGDVGADSEGHSDSVTAG-<br>GRQKRRRKVVPAVQAPTGRYNLRRHKTAAPLVANGALSDPNKGKEKEIDDGG-<br>GIGEEIPDEVDGNTHLVQVTTLKKRINVVNEFSSAGFHGINATSESQDRDAANQ-<br>LVSDTMLSEEVNGTPEQSRGYQNQGDTSGAEGEDEDGDEVEHPGEVSMRKKVW-<br>KFLTT |

| | | | |
|---|---|---|---|
| Daucus carota 2 | Embryo-phyta | Uniprot\| tr\| N0DLR1 | MFTPQKSNTNRSNLIPTTTMSHTNPRSTNKAKSVVFVNDPAPPRALLGGDY-<br>VAVERGEEEDWRRFREAGLLDEAAMERRDRDAVVEKVAKLERELFDYQYN-<br>MGLLLMEKTEWTLKYEEMRRAQVELKEVLEQEQTTHLILLSESEKREENLRKALD-<br>MEKKCITDLEKALRDSGADNAQTKQSSEAKMVKANALLSGFKEKSMDVETK-<br>LHVADAKLEEVYKTSLELERKLQEVETRDSLLQRERMSFIAEREAHEATFSIQ-<br>KKDLQEWEKKLQEAEERLCEIRRTTSGREVKVNEMEMALNLKKQELNKAQK-<br>ENDLSTSVLKKEADDINHRLANLTAQEHKAETLRNELEMRDKELLALAEKLTA-<br>RESVEIQTLLDEQQAVLDAKMQEFEVDMDGKRKSLDEEMRSKLDAVQYKKDE-<br>ITHIEEKLNRLELSLENKSERIKEKEKDLESKLRTLKDKESLLKSDEKRLDLEK-<br>KHMLADKDTLQTLKDEIEKTRADISQQQSKIQEEIVKLKISEDERAEYIRLRSEL-<br>KEEIEKCRFEKELLLKAHKNLKEDRKSFEEKWEALDERSNALSREIKLIGEEK-<br>EKFEKFRLSMEEKIKNDRLATEDYIRRELETLETEKETFATITRQEQSLISEKA-<br>ELEYSQMLHEFELRRKDLEVDIQKKRDELESHMSEREREFEEEREKEHNNIS-<br>RLKEVAQKDMEELRSEKRRIEKDRQEIALKKKELKEHQLEMHKDIDELEVLNK-<br>KVKIQREQFIKERDRFLLFVDTLKSCNYCGGCTREYELSDLQLLEKEIDNSPI-<br>VELGPGVSYESQDRINLRSSNSGGHISWLQKCTSKIFKYSPGKAAQDSEFQ-<br>SDMLATVEEDERPSDGHLETRGLNIANDGPEPSFGIANESCEIHLLASNDNK-<br>RDADQRHEICTDELSNIDSKAPVAPEDSQQSELSSGRRRPGKKTRSGSVAV-<br>GTTKRKRQAQPSGVMKSAVTADHSEEHSESVSEVGRRKRQQSVTSSVQTP-<br>GEKRYNLRRNKIVGTSGSALASVDVLKVESEVDVNKTETVQDYALASSQVIA-<br>SEKDNPTGPLENMTCRSLEIYDLSTEGDVELKTSKSRDKSIDPAIMGNIEFNE-<br>EVNSTIPECSIENGRGSTLHEDRDNEVEVEVLNEDEDLDIDSEGDVSIHKKLWTFLTT |
| Daucus carota 3 | Embryo-phyta | NCBI\| BAI67718 | MASPRLTVIQSEKTTVTSSRVSRSSMSDDDIWKRLQEAGFDEDSIKRRDKASLIAY-<br>ITKLEAEIYDHQYQMGLLIMERKEWGSKFERVEAALNSAELMRKHDKNLYLKD-<br>LAEAKKREENLKKAIEIERECLANIEKTLHELRAEYAETKVMAD-<br>SKLVEARSMIEDALKKLSEADAKKHAAESL-<br>EAEASRYHSAAERKLHEVEAREDDLRRRAT-<br>SFKTECDTKEEEILHERRLLNERQKALQQS-<br>QQRLVDGQDLLNKRESHIFERTQELNRKEK-<br>ELEASKLKQEEELQALVEQQANLETKASSLS-<br>LREEVITKSELEVKKREEELCVLQEKLEKKE-<br>SERIQQLLANYEASLSMKKSEFEAELEVKR-<br>KSVHDDIENKRRDWELREVDLHHREELILE-<br>KEHELEMQSRAVVDKERDLAGRFSLLEEKEN-<br>RLHAVEKEIESKEALLQKEKEEIISSKLDIQ-<br>RSLDALEDEKKQLHHAEEKMEAMKSETNEL-<br>CVLESKLKEEIETIRAQKQELETEADEMKEL-<br>KLKFEIEWQSIDEKRKELQKEAECINEQRE-<br>SLELTLKDERNSLKLEKDAMRDEYMRNN-<br>ESLSRDREDFMKKMEHERSEWFSKIQKERS-<br>DYLLAIEVQSKDLEDRLAKRREEIESYLAER-<br>ERAFEEEKKKELMRMDTLRETLARETEQV-<br>NAELNRLDTERREINLDRERRDREWAELNT-<br>LIEELKVQRQKLEKQRELMRADKEEILVQIEH-<br>LKQLEDLKVVPDRIALTDIQQSDLQPSKR-<br>VSARRSLKRQSGLDSGCRAEDNGNASSGN-<br>GSVILSPPLSSPFSWLKRCASSLLEQKVSNKK-<br>MRHSEEIITPSTIPARLNAPDDEHAVISANQQTP-<br>VHAKETTVYIDKIITIREVTSFNDAIVD-<br>GNNQNLEEALSQRAEEKLEDDNNIESEKLEKNGEVDPKIMQASLTEQ |
| Vitis vinifera 1 | Embryo-phyta | Uniprot\| tr\| F6HSF7 | MELLSEIKKKNSVHVRLGFLFHLSLHSASPTLRVVEIVVMFTPQRKVWSGWSLT-<br>PRSDAQKNAAGSGSNLSPRNGGVGDGSVSKGKSAAFVEPVTPGENGGN-<br>MVERPGEVASDLEALVAKVSKLESEIFEYQYNMGLLLIEKKEWTSKYDELRQALVD-<br>VKDALKREQDAHLVAMSEVEKREENLRKALGIEKQCVLDLEKALHEMRSEY-<br>AEIKFTSDSKLAEANALVTSIEERSFEVEAKLHAADAKLAEVSRKSSEIERK-<br>SQEVDARENALRRERLSFNAEREAHETTLSKQREDLREWEKKLQEEEERL-<br>GEGRRILNQREERANENDKIFTQKEKDLEEAQKKNEMTHLTLKKKEDDISG-<br>RLSNLTLKEKETDAVRQSLEIKEKELLELEEKLCARERVEIQKLVDEHNIILDA-<br>KKREFELEIEQKRKSLEEELKSKVVEVEKKETEFNHMEAKVAKREQALEKK-<br>LEKFKEKEKEFESKSKALKEKEKSIRAEEKNLEAEKKHILADKEDLLSLKAVAE-<br>KIRVEIEEQKLKVHEEREQLEITEEERSEFLRLQSELKQEIEKYRLEKEVLLK-<br>EVEDLKLQRETFEREWEVLDEKRAEIEKDLIDVSEQREKLEKLKHSEEERLK-<br>TEKLATQDYIQREFESLKLAKESFAASMEHEQSVLSEKAQSEKSQMIHDFE-<br>LLKRELETDIQNRQEELEKQLQEREKVFEEERERELNNVNYLREVARQEM-<br>EEVKLERLRIEKEKQEVAANKKHLDEHQFEMRKDIDELVSLSRKLKDQREL-<br>FSKERERFIAFVEQQKSCKNCGEITCEFVLSDLQPLPEIENVEVPPLPRLAD-<br>RYFKGSVQGNMAASERQNNEMTPGIVGSGSPTSGGTISFLRKCTSKIFNLS-<br>PGKKIEVAAIQNLTEAPEPSRQAIVEPSKRLGSTEDEPEPSFRIANDSFDVQ-<br>RIQSDNSIKEVEAGQDLSIDESNIDSKALELQQHSQHSDLKGARRKPGKRS-<br>KQRIHRTRSVKAVVRDAKAILGESLELSENEHPNGNPEDSAHMNDESRGE-<br>SSFADKGTPRNGRKRQRAYTSQTMVSEQDGDDSEGRSDSVMARRQGKR-<br>RQKVPPAVQTLGQERYNLRRPKTTVTVAAAKSSTNLHKRKETETDGSGAG-<br>GTGEEIPDCNAAPATSVGLISENGGSTHVLQVETFKTIVDVHFPSDRVVRL-<br>EAAEDTQDDNADVTKELVENMALSEEVNETPDEGPMEYRNGDGDEDEDT-<br>NEDDEDEEYEHPGEVSIGKKLWTFLTT |
| Vitis vinifera 2 | Embryo-phyta | Uniprot\| tr\| A5BQE9 | MAAFKERNSESFGPFYFFRHKVSRKAWTGLSLTPRSEAQKSGGGAVSNPVNG-<br>GKGKSVAFVDGPPPPLGSLSGKAMLTGIDGGDMEDWRRLREAGLLDEAAMERK-<br>DREALVEKVSKLQNELFDYQYSMGLLLIEKKEWTSKYEELSQALAEAQEILKREK-<br>SAHFIAISEVEKREENLRKALGVERQCVAELEKALGEIHAEHSQIKLSSETKLSDA-<br>NALVAKIEKRSLEVEEKLLAADAKLAEASRKSSELERKLQEVEARESVLRRERLS-<br>LNAEREAHEATFHKQKEDLREWERKLQEGEERLCEGRRIINQREEKANEIDRTL-<br>KLKERNLEEAQKKIDLDSLNVKVKEDDINNRLAELTVKEKQAESMRGILEVKEKEL-<br>IVLQEKLSARERVEIQKLLDEHRAILDTKKQEFELEMEQKRNSVDEELRSKVHE-<br>VEQKEVEVLHREEKLGKREQALEKRLERVKEKEKELEAKLKTLKEKEKSLKAEE-<br>KRVEGEKKQMLADKESLHLLKDELEKIRADITEQELQIHEETERLKVTEEERSEH-<br>HRLQLELKQEIDKCRHQEEMLQKEREDLKQERIMFEKDWEALDEKRAVITKEMR-<br>EIGDEKEKLEKLHLSEEERLKKEKLAMEEHIQRELEAVRIEKESFAAIMKHEQVTL-<br>SEKAQNDHSQMLRDFELRKRDLEIEMQNRQDEIQKRLQERERAFEEERERELN-<br>NINHLKEVARREIEEMKTERRRIEKEKQEVLLNKRQLEGHQLEMRKDIDELGILS-<br>RKLKDQREQFIKERDRFLTFVDKHKTCKNCGEITREFVLNDLQLPEMEVEAFPL-<br>PNLADEFLNSPQGNMAASDGTNVKIXTGEIDLVSSGSGGRMSFLRKCATKIFNL-<br>SPSKKSEHVGVQVLREESPLLDLQVNLEKAEGPSIVGQSIAEDELEPSFGIANDS-<br>FDIQQLHSDSVMREVDGGHAQSVDGVSNMGSKEQEGPEDSQQSELKSGRRK-<br>PGRKRRTGVHRTRSVKNVLNGDERPNDSTYTNEEGERETSHAEKAASTITRK-<br>RQRAPSSRITESEQDAADSEGRSDSVTAGGRGKRRQTVAPVVQTPGEKRYNL-<br>RRHKTAGTVATAQASANLPKRDEKGGDGGDDNTLQTKANPKAASSPSLADSD-<br>NPKTTPLVHVTTLKSVEIREYSPDRVVRFKTVDIVGGNNDSARLAENMELRQEI-<br>PGNPGDTPGYEDENGSMSHEEDDNSDEDESEHPGDASIGKKLWNFFTT |

| | | | |
|---|---|---|---|
| Vitis vinifera 3 | Embryo-phyta | Uniprot\| tr\| D7TG95 | MASPQPARFSIAATPGSRVLQSPLSDDAIWKRLRDAGFDEESIKRRDKAALIAYIAK-<br>LEAEIFDHQHHMGLLILERKEWATKYEQIKTEAESAEIVYKRDQSAHSSALAEAR-<br>KREDSLKKALEIEKECIANLEKALHEMRQECAETKVAAEIKLAEAHSMVEDAQKR-<br>FVEAEAKLHAAEAFQAEAICFRRTAERKLQEVEAREDDLRRRLISFKSDCDEKE-<br>KEIILERQSLSERQKNVQQGQERLIDGQALLNQREEYIFSRSQELNRLEKELE-<br>ASKSNIEKELRALNEEKSNLELKLASLTTREEDVVKREALLNKKEHEILILQEKI-<br>ASKESDEVQKLMALHEIALKTRKAEFEAELETKRKLVEDEIEAKRRASELREV-<br>DLSNREDFALEREHELEVQSRALAEKEKDVTEKLNSLDEKEKYLNAAEKDVE-<br>LEKIHLEKEKEEINKMKLNIEKSLSSLEDKKKQVDHAKEKVEAMKSETSELLV-<br>LEMKLKEEIDVIRAQKLELMAEADELRAQKANFEAEWESIDEKREELRNEAER-<br>IAEERLAISKFLKDERDSLKLEKDAMRDQYKQEVESLSREREDFMSKMVHE-<br>RSEWFSKIQQERADFLLDIEMQKKELENCIDNRREELESYFKEREKTFEQEK-<br>MKELQHISSMKERVAKELEHVASEMKRLDAERMEINLDHERRDREWAELSNS-<br>IEELKMQRQKLKKQRELLHADRKEIHTQIEHLKKLEDLKIASDNIALAEMQQS-<br>NQEPSQRKVYVKRYYKAQNTIPNADFESHQKINVVKNGSGFNLPALPDSSSP-<br>STATPFSWFKRCAELIFKLSPEKPSIKHGEKSSISNSENANLTLAGNLDLSDG-<br>FDREVHDRNEKTHSISDRQPTRYALGEPKVILEVPSSGEDVKGLHTLESEIKK-<br>DTSENSSHSFSEKELLAGRKRRVVNSSSNDWVDTTLEQRQKNKKRRQQES-<br>AADPCGVSIQSDAREGQDVSISLNQTQGGAEETNLLITDEIIKISEVTCENVV-<br>FDNQAKPNALQNSVVELGQDIQHGGTNGLADSNAENCVLSSDFKAQEKIGK-<br>EVLFVDVGQVIEHSQPQDESISEKSQQELQEQGVPKSDDDKKLSEKVGRRMR-<br>SRQKS |
| Citrus clementina 1 | Embryo-phyta | Uniprot\| tr\| V4T3E0 | MFTPQKKAWSGWSLTPRGEKNGTGSVSNPTTVDGLTGKGKSIVAFTEPRT-<br>PQNGVGLADDVESLAEKVSKLENELFEYQYNMGLLLIEKKEWSSKYEELKQT-<br>FGEAKDALKREQAAHLIAITDVEKREENLRKALGVEKQCVLDLEKALREMRSE-<br>NAEIKFTADSKLAEANALVTSVEEKSLEVEAKLRSVDAKVAEI-<br>NRKSSEIERKSHELESRESALRMERASFIAEREAHEGTFS-<br>QQREDLREWERKLQDGEERLAKGQRIVNQREEKANEKEKI-<br>FKQKEKDLEEAQEKIDATNLSLMRKEDDINKRLANLITKEKA-<br>SEYDAARKSLEMKEEELRQLEEKLNAREKVEVEKLLDEHKA-<br>SLDAKQREFDLEIEQKRKAFDDDLKSKVVEVEKKEAEINHKE-<br>EKIAKREMALEKRLEKCKDKEKDVESKLKDLNGREKTMKSE-<br>EKNLETEKKQLLADKEDILTEKAELEKIRDANEQQLLKIYEEK-<br>NQLRISEEERAEYLRLQSELKEEIGKCRLQEEMLLKEAED-<br>LKQQKENFEKEWEQLDDKRAETEKLEKEKLSEEERIKRDK-<br>QLAEDHIKREWEALEVAKESFKATMDHEQSMITEKAESERR-<br>QLLHDFELQKRKLESDMQNRQEELEKDLKEKERLFEEEKER-<br>ELSNINYLRDIARKEMEEMKLERLKLEKEKQEVDSHRKHLEG-<br>EQVGIRKDIDMLVGLTKMLKEQREQIVKERDRFLNFVEKQKK-<br>CEHCAEITSEFVLSDLVQEIVKSEVPPLPRVANDYVNEKKN-<br>SEMSPDVLASGSPASAGTISWLRKCTSKIFKLSPSKKGENT-<br>VVRELTEETPSSGGQTKLQESSRRLGQTNEPDLSFAIVND-<br>SFDAQRYHSETSTREVEADQHKQVDGQNNLNGKAPEVQ-<br>ENSQPSDLNHGRQPRKRGRPRVSRTRSVKAVVQDAKAIL-<br>GEGFELTESENLNGNADDSVQEAAESRGEPSLDDKGTSR-<br>NARKRNHAQSSQITTSEHDVDDSEAQSGSVVVGQPRKRR-<br>QKVDPAEQTPVPTRYNLRRPKTGAPAAAVSEPNKEKEEVS-<br>EGVRGALEDEIVNSKAAPPNSVGVFSDNGRSSQLVRCGA-<br>VDNNDASKQFVENMAMTMSEEVNGTPEGAGDYGDADE-<br>FRSESPGEDASGFDGGDSDDECEHPGEASIGKKIWTFFTT |
| Citrus clementina 2 | Embryo-phyta | Uniprot\| tr\| V4TH87 | MFTPQRRPIPATKLTPRGTEAQRSGAISNARNIKGKAVAFAETPSVPPPPPVNSLL-<br>DYNSGSATVFPAESEDDWRRFREAGLLDEATMERKDREALMEKVSKLEKELY-<br>DYQYNMGLLLIEKKEWTSKIEELRQSFEETQEILKREQSAHLIAFSEAEKREDNLR-<br>RALSMEKQCVADLEKALRDMGEEHAQTKLFSEKTLTDANTLLGGIEGKSL-<br>EVEEKFHAAEAKLAEVNRKSSELEMKLQELESRESVIKRERLSLVTEREA-<br>HEAAFYKQREDLREWEKKLQIGDERLSELRRTLNQREVKANENERILKQK-<br>ERDLEELEKKIDLSSSKLKEREDEINSRLAELVVKEREADCLRSTVEMKEKR-<br>LLTIEEKLNARERVEIQKLLDDQRAILDAKQQEFELELEEKRKSIEEEMRSK-<br>ISALDQQEFEISHREEKLERREQALDKKSDRVKEKENDLAARLKSVKERE-<br>KFVKAEEKKLELEKQKLIADKESLQILKVEIDKIESENAQQELQIQEECQKLK-<br>INEEEKSELLRLQSQLKQQIETYRHQQELLLKEHEDLQQDREKFEKEWEV-<br>LDEKRDEINKEQEKIADEKKKLEKLQHSAEERLKKEECAMRDYVQREIEAI-<br>RLDKEAFEATMRHEQLVLSEKAKNDRRKMLEEFEMQRMNQEAELLNRR-<br>DKMEKELQERTRTFEEKRERVLNDIAHLKEVAEGEIQEIKSERDQLEKEK-<br>HEVKVNREKLQEQQLGMRKDIDELDILCRRLYGDREQFKREKERFLEFV-<br>EKHTSCKNCGEMMRAFVISNLQLPDDEARNDIPLPQVAERCLGNLQGDV-<br>AAPYDSNISNSHGGMNLGRADSGGRMSWLRKCTSKIFSISPIKKSEHIST-<br>SMLEEEEPQSAVPTIMQEKAEGPGVLVSKEAIGYSSPEDEPQSSFRLVND-<br>STNREVDDEYAPSVDGHSYMDSKVEDVAEDSQQSELRSGKRRPGRKR-<br>KSGVNRTRSLKAAVEDAKLFLGESPEGAGLNASFQAHEDSQGISSHTQE-<br>ASNMAKKRRRPQTSKTTQSEKDGAGSEGYSDSVTAGGGRRKRRQTVA-<br>TVSQTPGERRYNLRRHKTSSAVLALEASADLSKANKTVAEVTNPVEVVSN-<br>PKSASTFPPAVLNENRKSTHLAQVTSVKSMELSQDRAVRFKSTTNIVDEN-<br>ADAPKSIENTVLSEEVNGTSEYVDEDENGGRVLEDEEDDDDDSDHPGEASIGKKL-<br>WNFFTS |
| Citrus clementina 3 | Embryo-phyta | Uniprot\| tr\| V4SQK6 | MASPSSGRLSITPSSRVLQSPLSDESIWKRLKEAGLDEESIKRRDKAALIAYIAK-<br>LETEIFEHQHHMGLLILEKKELASKYEQIKASAEAAELLQKHDQASHLSAIAEAR-<br>KREESLKKTLGVEKECIASLEKAVHEIRAESAETKVAADSKFAEARCMVE-<br>NAQKKFAEAEAKLHASESLQAEANRYHRSAERKLQDVVAREDDLSRRIASFKAD-<br>CEEKEREIIRERQSLSDRKKILQQEHERLLDAQTLLNEREDHILSKLQELSRKEKE-<br>LEASRANVEEKFKALNEEKSNLDLTLVSLLKREEAVIEREASLQKKEQKLLVSQET-<br>LASKESNEIQKIIANHESALRVKQSEFEAELAIKYKLAEDEIEKKRRAWELRDLDLG-<br>QREESLLEREHDLEVQSRALVDKEKDLVERSHLLEEKENKLIAFEKEADLKKSLLQ-<br>KEKEEVNIIKSDLQKSLSSLDEKKKQVNCAKDKLEAMKSEAGELSVLEIKLKEELD-<br>VVRAQKLELMVETDKLQLEKAKFEAEWEMIDEKREELRKEAESVAVERVVVSKSL-<br>KDERDSLRQERDAMRDQHKRDVDSLNREREEFMNKMVHEHSEWFTKIQQERA-<br>DFLLGIEMQKRDLENCIEKRREELESSFREREKAFEEEKMRELQQISSLKEKAEKE-<br>LEQVTLEIKRLDLERMEINMDRQRRDREWAELNNSIEELKVQRQKLEEQRQLLH-<br>ADREEIQAESERLKKLEDLKIAVDYMAVSEMQRSRLEHSQKKISAKRHLNQQTSL-<br>AHADLGSDQKFDVTNNGDRFNTPSVQKTASASPPSLARFSWIKRFADLVFKHSG-<br>ENSIENDEEKSPTSDHEDASLTINSRKRQPVRYSFGEPKVILEVPSENEVVKRTVD-<br>LESENNQNAAQKCKQSVSEDGIHAARKRRVDVDCVDPSELLMQNNKRRKQQED-<br>FPRNSSEEAINHGAVAEQSNLPEDQHTLTSKNKSNVPEGLHTLTSNNHTQGGNE-<br>EASILIVDKIIKISEVTCEMPDADNFINQEKIDGSQNSVAESVQDIVKVGGTNDHST-<br>PAHTDDVVLPYVSEIDGMVQEKQMGNVKDLTECGQAQNEIGEHKLECELV-<br>QSDNSKKNKELIAYRTRSKQKK |

| | | | |
|---|---|---|---|
| Phaseolus vulgaris 1 | Embryo-<br>phyta | Uniprot\| tr\|<br>V7B0B4 | MFTPQKVWSGWSLTPNKSGVRGGTGSGSDLGPNSGDGVSAKEQGIVAVVENG-<br>GNNLDRGVLVERVSNLEKELYEYQFNMGLLLIEKKEWTSKYTEQSQDLVEVK-<br>DALEREKAAHLIALSEAEKREENLRKALGVEKECVLDLEKALREIRSENAKIKF-<br>TAESKLAEANALVASVEEKSLEVEAKLRSADAKFAEISRKSSEFDRKSQDLESQE-<br>SSLRRDRLSFIAEQEAHESTLSKQREDLWEWEKKLQEGEERLAKGQRIINERE-<br>QRANENDKLCRQKEKDLEEAQKKIDATNITLRSKEDDVNNRLADIALKEKEYDSL-<br>GINLDLKEKELSAWEEKLNAKEKVEMQKLLDEHNAVLDVKKQEFEVELNEKRKS-<br>FEDGLKDKLVELEKKEAEINHMEEKVGKREQALEKKAEKLKEKEKEYEQKVKAL-<br>KEKEKSIKSEERSLETTKKKIESEREELVTDKAEVEKIRSNNEQELLRINEEIERLK-<br>VTEEERSEYLRLQSQLKHEVDQYRHQKELLVKESEDLRQQKESFEREWDELDL-<br>KRADVEKELKSVIQQKEEILKLQQFEEEKLKNEKQAAQDHIKRELETLALAKESF-<br>AAEMELEKSSLAEKAQSQRNQMLLDFELQKKELEADMQNQLEQKEKDLIERKN-<br>LFEEKRESELNNINFLREVANREMDEMKLQRSKLEKEKQETDENKKHLESQRM-<br>EMQEDIDLLVDLNRKLKNQREQFIVERQRFIEFVEKLRSCQNCGEIISEFVLSDL-<br>QSSDDIENLEVPSLPKLAGDIILGDSIENLASSRKNIGASPATDQKSPVSAGTISW-<br>LRKCTSKIFKISPISKFESEDSGTLRDVMNLSVEKTNMDSRHENEAELSFAVVND-<br>SLDGRRARSGNDITEVEAVDQDPSVENQSNIDSKTPEESKAEQQKSRRGGGRT-<br>RIKRTHTVKAVLKEARGILGEAAELLPGESVDNHETEFPNGNAEDSANVNSESQ-<br>GLSNRRIPMNVRKRNRVQTSQMTVSEHDGEASEGHSDSVIPGQRKKRRQKAA-<br>APPAQTAGETRYNLRRPRTGATTSSARATSAGGKESQGEVHRVKDTEEEIVDSK-<br>ISHSLSVGITNEDGGSVHLEQSMKGVETRDGYGGDTTGTFANNITLSEEVNGTA-<br>DDAEENDAEYRSESHGEDAGGVEIDDDEDYQHPGEASIGKKLWNFFTT |
| Phaseolus vulgaris 2 | Embryo-<br>phyta | Uniprot\| tr\|<br>V7C8S5 | MFTPQQKAWPNAAVPFTPHRGGAATVSASAKGKAVADGPPPP-<br>PLGSLTETTVAVGFDTGNAEDWKRFTELGLLDESVMQRKDHEALMEKVLR-<br>LERELFDYQYNMGLLLIEKKEWNSKFDQLRQELAETEEILKRE-<br>QSAHLIALFEVEKREENLRKALSTERQCGADLERALRAMQEEHAQIQSKSHTKL-<br>AEASALVDGIEEKSSVVDKKLLDAEAKLAEVNRKNAELGMKLQEVEARESLLQK-<br>ERLSLVTDRELFDATFYKEREDLKEWERKLQQRENMLCNGRQNIGEKEENIVK-<br>TEKNLKQKERDLEVLEKKINSSNSILKEKEAEIIRRTADLNMEEKKVDSLKSMLEK-<br>KEKELFALESKLSSREREGIQKLLGEQKATLDLQLQQVEFEMEHKRKSLVEEFS-<br>SKEEALEQREVEVNHREKKVEKEEQALSKKAERLKEQSKEIEAKLKSLKEKEKT-<br>MKIKEKELEKDNQQLLADRESLENLNAELQKIKAEISLQELQICEETKNLKLTEDD-<br>RLENSRLQLELKQEIENTRLQKDSLVKEAESLREERQRFEKEWEVLDERREEIT-<br>RKQHDIDEEKESLRKLQNSEEERLRSKKQNMQEHIKKELEKLELEKESFRDSM-<br>NQEKHLLSEKVKNEQDKMLQDFESKTRNLENEIQKRQEEIEKDLQERERNFQE-<br>EMRKELDNINILKDVTEKEWEEAKAEGIRLENERKELELNKQQLKSGQQEMHE-<br>DSEMLMNLSQKVKKERQRLVAERKHFLELVENLKSCRVCGEVVGDFVISDIELP-<br>DFKESMAIPSPISPVLNYKSPKNSQDIVASSDINNSGSVRPVSWIRKCTSKIFKLS-<br>PNKRAEAVSALDTAGTSLPSDVNVSVEKADEPASLPNIEGARVILDERQPASGR-<br>AYHSLDTPLLQSENIDKELDDEYSQSVGDHSRVDSLVDGDRDDSHQSVPKLRR-<br>GRPGKKSKSGIARTRSVKAVVEEAREFLGKTPKKNENASLQSLTTDHIKEDSRE-<br>DSSHVEKAVGNTGRKRQRAQTSRVTESEQNAGDSEGQSESITAGGRRKKRQA-<br>LAPPAQVTSEKRYNLRQHKIAGKDSSTRDLPNATKSVVKEAAGGNKLKGEMSP-<br>EVVETSLAAADDNAQDKSMVQDSTTKTVEVSDERVVVRFEVPRDIVDDNGAAT-<br>DSLNPAEENGTPEHQNENGSTIHDFEDEDDDDDEEEDGDEEHPGEV-<br>SIGKKIFRFFTT |
| Phaseolus vulgaris 3 | Embryo-<br>phyta | Uniprot\| tr\|<br>V7CFW3 | MELSTPNSSKPLSITPGSRVLKSPLIDEQIWKRLRDAGFDEESIKHKDKAALIAYIAK-<br>LEAEIYDHQHHMGLLIMEKKDLASKYEQLEALAESSELMHKHDSAMNKSALAES-<br>RKREESLKKTVSVKDACIASLEKALHELRTESAETKVAAESK-<br>FAEAHQLIDEAQKKITEAEAKVRAAESLQTE-<br>ANRYHNAAERKLRDVEAREDNLRRKIMSFK-<br>ADCDEKDKEMIFERQSLSERQKGLQEEQER-<br>LLQSQSLLNQREEHFLSRSQELNRLQKELED-<br>TKAKVEKEHETLHDEKTTLKMKEATLMQREE-<br>ELAKWKTELSKKEQELLEFQAKLSIRESDET-<br>KKVIAGQEAALKTKKYNLEVELQMQRKWVEN-<br>DIETKRRAWELKEVDLKHCKDEILEKQHELEA-<br>LSRSLSEKEKDLKDLSSALEEKDQKLSAAEKE-<br>FELNKVLLQKEKDTIEQAKQDLQKSLASLENK-<br>RRQVDIDKERFEAVKNETGDLSILEVKLKEEID-<br>LVRSQKFELLAEADKLKAEKAKFEAEWELLDE-<br>KKEELQKEAEFIAKEREAVSTFIKNERDQLK-<br>EEKENLRYQYTQDLGFLASERESFMNKMA-<br>QEHAELFGKMQQERADFLREIEMQKQELNN-<br>LIEKRREEVESYLKEREKAFEEEKNTELHYI-<br>NARKEKVAKELDQVSLEMKRLQTERAEINL-<br>DRERRNREWAELTNCIEELEVQRDKLQKQRE-<br>LLHADRVEIFAQTEELKKLEDLKAVSDDNAIT-<br>EMLKSDMESNRKKISSRKNLKRQTLTQGGD-<br>KISNGFDTPFVERSSAGSPPSPVRFSWIKRC-<br>SELIFRNSPVASDADTGSNSQKHLENDKPLG-<br>IGKGQQMGFSFEESKVIVEVPSRDDARRRE-<br>IESEAKNVNGKSALLFPDGHLAGRRKRGR-<br>GNVTSKVGDPLVDLGQNKKSRAEGQTTENP-<br>IDQGTTRRVVSTQSDVLKVQQVLTSSNQTQ-<br>GNTEETRVVMVDKVIHVSEVTSEKVDALPID-<br>SQEPGDNPQNPALAEDHYGETIDQINSKTKREDILPRVSRVLGSTEEISK-<br>GNNGQDSENC |
| Prunus persica 1 | Embryo-<br>phyta | Uniprot\| tr\|<br>M5WQZ9 | MFTPQRWSGWSLTPKTGAEKTGTGSGSNMKSGTPNFNSGDGV-<br>VAKGKGLSLFEPRTPASGSVLENGGNMQVESGEGATDREELAQRVSE-<br>LENELFEYQYNMGLLLIEKKEWTSRHEELRQSLTEAKDAVRREQAAHLIAI-<br>SEIEKREENLRKALGVEKQCVHDLEKALHEIRSENAEIKFTADSKLAEANALVASIEE-<br>KSLELEAKSRAADAKLAEVSRKSSEFERKSKDLEDRESALRRDRLSFNSEQEAHE-<br>NSLSKRREDLLEWERKLQEGEERLAKGQRILNQREERANENDRIFKQKEKDLEDA-<br>QKKIDATNETLKRKEDDISSRLANLTLKEKASSEYDTMRINLEMKEKELLALEEKLNA-<br>RERVELQKIIDEHNAILDAKKCEFELEIDQKRKSLDDELRNRLVDVEKKESEINHMEE-<br>KVAKREQALEKKGEKVREKEKDFESKMKSLKEKEKSIKSEEKDLESEKKQLIADKE-<br>DLVRLLAEVEKIRANNEEQLQKISEEKDRLKVSEEEKSEYHRLQSELKQEIDKYMQ-<br>QKELLLKEAEDLKQQKELFEREWEELDDKRAEIEKELKNVNEQKEEVEKWKHVEE-<br>ERLKSEKVMAQDHIQREQDDLKLAKESFEAHMEHEKSVLDEKAQSERSQMLHELE-<br>TRKRELEIDMQNRLEEMEKPLREREKSFAEERERELDNVNYLREVARREMEEIKV-<br>ERLKIEKEREEADANKEHLERQHIEIRKDIDELLDLSQKLRDQREQFIKERESFISFIE-<br>KFKSCTNCGEMISEFVLSNLRPLAEIENAEVIPPPRLGDDYLKGGFNENLAQRQNNE-<br>ISLGIDSRSPVSGGTISWLRKCTSKIFNLSPGKKIEFGSPQNLANEAPFSGEQNVEA-<br>SKRGCGIENEAELSFGVASDSFDVQRVQSDNRIREVEAVQYPSPDEHSNMNSEAP-<br>DLPEDSQPSDLKGGCQKPSRRGGRRGRPAVKRTRSVKAVVKDAKAILGEAFETND-<br>SEYANGTAEDSVDMHTESHGGSSLADKRSARNGRKRGRAQTSQIAVSGGDDSEG-<br>RSDSVMGAQRKKRREKVIPAEQAPGESRYNLRRPKTGVTVAAASASRDLVKDNEE-<br>EVDNARATEHYSKAAPATSIGVGSENGGSTHFVRCGTLGDTQDGEADAIKNLEENT-<br>AVSEEVNGSTEGGQEYVDGDEYRSESQNGTPIEEDDDDEESEHPGEASIGK-<br>KLWTFFTT |

| | | | |
|---|---|---|---|
| Prunus persica 2 | Embryo-phyta | Uniprot\| tr\| M5Y1X5 | MMFTPQRKALNAQSLTPRSGAVVSNPRTAGKGKAVAFVDGPPPPLGSLSESGPK-<br>TIPDFDTGDMDDWRRFKEVGLLNEAAMERKDRQALADKVSKLQKELYDYQYN-<br>MGLLLIEKKEWALKHEELGEALAETQEILKREQSAHLISISEVEKREENLRKVL-<br>VAEKQCVAELEKALREMHEEHAQIKLKSEAKLADANSLVVGIEE-<br>KSLETDAKFLAAEANIAEVNRKSTELEMRLQEVEARESVLRRE-<br>HLSLSAEREAHKKTFYKQREDLQEWERKLQEGEERLCKLRRIL-<br>NEKEEKANENDLIMKQKEKELDEVQKKIELSNTILKEKKADVNK-<br>RLADLVSKEKEADSVGKIWELKEKELHELEEKLSSRENAEIEQ-<br>VLDKQRALCNTKMQEFELEMEERRKSLDKELSGKVEVVEQKE-<br>LKINHREEKLLKQEQALHEKSERLKEKNKELETKSKNLKENEK-<br>TIKVNEEMLEVERQQVLADLESFQNLKEEIQKIKDENVQLELQIR-<br>EEREKLVITQEERSEHLRLQSELQQEIKTYRLQNELLSKEAED-<br>LKQQREKFEEEWENLDERKAEISRGLEKIVEEKEKLEKLQGT-<br>EEERLKEEKHAMQDYIKRELDNLNLEKESFAAKMRNEQFAIA-<br>EKAQFQHSQMVQDFESQKRELEVDMQNRQQEMEKHLQEME-<br>RAFEEEKDREYTNINFLKEVAEKKSEELRSEKYRMEKEREEL-<br>ALNKKQVEVNQLEMRKDIDQLAMLSKKIKHQREQLIEERGRFL-<br>AFVEKIKSCKDCGEMTREFVLSDLQVPGMYHHIEAVSLPRLSD-<br>EFLKNSQADLSAPDLEYPESGWGTSLLRKCKSMVSKVSPIKKM-<br>EHITDAVSTELPPLSTMKVNEGARGHIGHEDEPEPSFRMPNDA-<br>ISQPLPSDNTTKEVDDGYAPSIDDHSFIDSKVKDVPDDSEQSE-<br>LKSYQCKPGRGRKSRLSRTRTVKATVEEAKIFLRDTLEEPSNA-<br>SMLPNDSSNIHEESRGDSSFVEKANTSIGRKRRRAQSSRITE-<br>SEQDDCDSEGRSGSVTTAGGRRKRRQSIASSVQAPGEQRYN-<br>LRHRKTAGSVTAAPAAADLKKRRKEEAGGGGAEPNPESVSS-<br>LGMAGETGQTAQLMQVTTSKSVEFSQERVVRFSTPEDIVDGN-<br>AADAAKTVENTELSGEDNGTPESGSGNNTVGESDDDYDDEERPGEASIR-<br>KKIWNFLTT |
| Prunus persica 3 | Embryo-phyta | Uniprot\| tr\| M5WL04 | MASPQSELFARTPGSGRALSITPGARILQSPFSDEAIWKRLKEAGFDEESIKRRD-<br>KAALIAYIAKLEAEIFDHQHHMGLLIMERKELASKYEEVKASNET-<br>TELLHKRDQAAYVSALAEARKREECLKKVVGVKEECISSIEKSMHEMRAESAETK-<br>VAAESKLAEARNMVEGAQKKFTEAEAKLHVAESLQAEASRFHRVAER-<br>KMQEVEAREDALRRNILSFKTDCDTKEKEISLERQSLCERQKTLQQEQ-<br>DRLLDAQALLNQREDFIFGRSQELNRLEKELEDVKANIEKERRALDDGK-<br>LNLELTEASLVNREEALTRREALLNKKEQEILVLQEKLVSKESDEIRKAL-<br>ASHEVELRKKKFEFDSELDVKRKLFEDEIEAKRRAWELREVDLNQRD-<br>DLLQEREHDLEVQLRTLVDREKDVAEMSNLVDEKEKTLRDAEKEFELN-<br>NVLLQREKEEIIKMKVELQCSLDSLEDKRKQLDCAREKFEVLKTETSEL-<br>SDLEMKLKEEIDLVRAQKQELMAEADKLAVEKAKFESEWELIDEKREE-<br>LQKEAEHVAEERLAFSKFIKDEHDNLRQEKEEMRDQHKRDVELLVSE-<br>REDFMNKMVHERSEWFGKMQKERADFLLEIEMRKRELENCIDKKHEE-<br>LECSLKEKEIAFEQEKKNEFQNINSLKEEAAKEREQVALERKRLETERI-<br>EINLDRERRDREWAELNNSIEELRVQREKLKEQRELLHADREEILGQIQ-<br>HLKELESLKAALDSASVSEMQQSDLVPRSRKTSRRYLKQLTSVREAD-<br>HNSHNEENVANISNSSIMLKSGFSPSSSARFSWLKRCRELLFKQSPE-<br>KHQTEYEENHVISREETSLTVTEQVDTSSKYDGHRYTGNGNSPRFFS-<br>KRQNAFGEPKVIVEVPFVGETVKGTHTESEIKEFDGESCSPLISEHVC-<br>QGGRKRRVDKSLSNDGFDPLLEPRQNLKKRRQQQDATVNSSEHANT-<br>HCIVSTQEKVLEDQNISMPLPSDQICEGAEEGSALIVDKIIKVSEVIFEE-<br>TGTGSLGNEGKLEAQNSIVEAHHGQNGVFQGAVGQVTEHCQ |
| Eutrema salsugi-neum 1 | Embryo-phyta | Uniprot\| tr\| V4KN99 | MSTPLKVWQRWSTPTKATNPDSNGKGPANMVTPVPGRVSEIYYDDPRILPEKVSE-<br>LEKELFEYQHNLGLLLIEQKEWSSKYEELQQEFEEVNECLKRERNAHLIAVAD-<br>VEKREEGLRKALGIEKQCAVDLEKALRELRSENAEIKFTADSKLTEANALVRSVEEK-<br>SLEVEAKLRAVDARLAEVSRKSSEVERKSKEVEARESSIQRERFSYIAERDADEATL-<br>SKQREDLREWERKLQEGEERVAKSQMIVKQREDRANEGDKIIKQKGKELEEAQKK-<br>IDAANLALKKKEDDISSRIKALAFREQETEVLKKSIETKERELLALQEKLDAREKVAV-<br>QQLIDEHQAKLEAAQREFELEMEQKRKSIDDSLRSKVVEVEKREAEWKHMEEKVA-<br>KREQALDRKLEKHKEKEKEFELRLKGVSSREKALKSEEKALETEKRKLLEDKDIILN-<br>LKAEVEKMKTENEVQLSEIHKEKEGLRVTEEERSEYLRLQTELKEQLEKCRSQEEL-<br>LLKEVEDLKAQRECFEKEWEELDERKAEIESELKNITDQKEKLERHSHLEDERLKK-<br>EKQAANDNMKRELETLEVAKASFAETMEYERSVISKKAESEKSQLLHDIEMLKRNL-<br>EADMQTKLEEREKELQAKEKLFEEEREKELSNINYLRDVARREMADMQNERQRIE-<br>KEKLEVDASKKHLEEQQTEIRKDVDDLVALTKKLKEQREQFISERNRFLSSMESNR-<br>NCNPCGELLQEIVLPDIDNLEMTNLSKLTNILENEAPRQEMRDISPTAAGLGLPVPG-<br>GTVSWLRKCTSKILKLSPIKMAETSATRNLAGQEPQSTEQANVNSGPSTMLQAQS-<br>VSDTREVEVNNADSDGDQSNINSKAQEVEANSLSTLNADGQSRIRGKARARVRRT-<br>HSVKAVVEDAKAIYGKSIEFNEPEDSTENVEDSSKANDGNTGEPDHSGKGASKNG-<br>RKRGRVGSLRTCTTEQDGTESDGKSDSVTGGERQRVKRRQKVTSEQQEVVGQR-<br>YNLRRPRRGAGKTALGKKNEETVTVQQEEGIYSAQTIATASVGVAVSDNGASANV-<br>VQSETMADSEDTDAGSPKRTCESAAMSEEDVNKTPQRAHSGNEYDGEEDE-<br>SESEHPGKQSIGKKLWTFLTT |
| Eutrema salsugi-neum 2 | Embryo-phyta | Uniprot\| tr\| V4MU99 | MFTPQRKPWISPAVTPRSETRKIGGVSNPRNDDRKGKAIAISEDPVISTLPPP-<br>PIGTLTGEGVYRGQAEEMDMGDWRRFREVGLLDEASMERKDREALLEK-<br>ISTLEEELYGYQHNMGLLLMENKEWVAKHEELNQAFQEAQEILKRE-<br>QSSHLYALTTVEQREENLRKALGLEKQCVEELEKALREIQEEN-<br>NKIRLTSEAKLAEANALVASVTGRSSDVENKIYSAESKLAEAT-<br>RKSSELEMRLKEVETRESVLQQERLSFAKERESYEGIFHKQ-<br>REYLHEWEKKLQEKEESIPEQKRSLNQREEKVTEKEKNLKL-<br>KAKQLEEWDRKVELSVSKSKETEEDMNKRLQELAAKEKESC-<br>TLQSMLVAKESELRALEEKLIVREGTEIQKLIDDQKEALAAKM-<br>LEFELECEERRKSLDRELQKKIEEVERQRVEINHSEEKLQKRN-<br>EALNKKFDRVNEKEIELEARVKTIKEKEKIMQAEEKKLSLDKQ-<br>QLLSDKENLKDLQQELENIRSEMMRKEEMIQEEHKSLEIKKEE-<br>REEYLRLQSELKSQIEKSRLHEEFLSKEVENLKQEKEKFEKE-<br>WEILDEKQAEYNKERMIISEEQAKFQRFQLLEGERLKNEENAL-<br>RAQIKQELDDIRLQRESLEANMDHERSALHEKAKLEHSKVLED-<br>IEMMRRNIEIELQKRKEQDEKDRQDRLAQFEDKRMKELSD-<br>LNHQKQALNREMEEMVSKRSALQKESEEIAKHKMKLKEQQV-<br>EMQNDISELGTLSNNLKKRREEFARERARFLAFVQKLKDCES-<br>CGQLANEFALSDLQLPYNEEEATLPPNGVLCDLPESSDASDS-<br>CNIKKSLDGDAPASGGSGRPTMSILQKCTSLLFSPSKRAEHG-<br>MDTGKPEHLSSSVAVSKEIKVEKPLPVDLRPRPSSSSIPEED-<br>EEYTVSRVQETSEGSQLSEFQSAKRGRGRGRPRKPKPALN-<br>PSSSVKHASPEESSKDEAGGHVSVTSEKTTGRGGRKRQHI-<br>EDTTTTGGRRKRQQTVAVLPQTPGQRRYNLRRNRTVDQAP-<br>ADDEDNAAGGEYDADIAALAPSKDNVEETSESVVESLRARR-<br>LESSEVRVERVVTVETVTDADVAANNNVGVSVANEELAPNI-<br>ARSPSVEDEQRQRTVDEDKNEEYEDGDEEVHDDQDD-<br>DDEGGDDDDDDDGDDDGLKAGEGSIRKKLWTFFTT |

| | | | |
|---|---|---|---|
| Eutrema salsugi-neum 3 | Embryo-phyta | Uniprot\| tr\| V4KM52 | MHVDTHLYDYKSIMHWTSTNEELQQAIDEASEILKRERMSKLIALNEAEKREENL-RKALISEKQFAAELERDLKYLQQEHSEVKSTSEAKLAEANALVMGIKENSLEV-DRKRAIAEEKLSVINRKSSELEKKLKEVETREKVLQREHLSLVTEREA-HEAKKLTLEEDRLSEAKRSVNHIEERIIESEKTIKKKEKILKEKEEFINSMLNDISM-KEKAFEAMKTNIDMKEKELHELEEKLIVREQMETGKLFDDQNAVLDSRKHEFEM-ELEQMRISLNEELERKKSEVEQLEVEISHKGEKLAKKESTLEKMEEIVKEKEKDL-EARQEVVKEKEKALKAEEKKLHMENKRLLEDKESLRQLKDEIEEIGAKTTKQESR-IREEHESLRITKEERLKFLRLQSELKQQIDRVEQEEELLLKEREELKTAKGRLEK-EWEALDEKRADKTREQKEVTEEKENLRSLQTSEKHRLKREDIILRDNSKREVD-DVEMQRESFEAGIETKKLFSHDNANIQTKREMEEVQYEKLALKREREEISVKRK-TLYKQSVVFYMGVDDSLRISLKEKREQICCAKERFALFLKENKLCNSCGEKFHK-FVQSNRAPDIETMKSEMKKLDLPAKNQTTPDVENGDKLSDNSKSASLIGTLAAIK-LPESWQHYDTLDTLDTLDTLDTVAGNDHEASGTEQSFAEIKSDKSRRGRGRSK-SVRGRPQATKAASRDSKTSDEEIVKVEAETEFKNDNRGKRPVQDPQFEAGSS-GEKKEDDGNISMIEEENKGEEEEEEETERPDEASIGKKIWAFLTI |
| Eutrema salsugi-neum 4 | Embryo-phyta | Uniprot\| tr\| V4KV78 | MATSRSERFPITPNTASNRLTITPGSRVLKSPLTEEVMWKRLKEAGFDEQSIKNRD-KAALIAYIAKLESEVYDYQHNMGLLILEKDELLSKYEEVKASVNEAD-LAHRRDQSAYVSALAEAKKREEDLKKDVGIAKECISSLEKTLHEMRAE-CAETKVSAGSKMSEAHLMIEDALKKYADAEAKMRAAEALQ-AEANRYHRIAERKLKEVESREDDLARRLASFKSDSETREN-EIDIERQTLSERRKSLQQEHERLLDAQASLNQREDHIFGRS-QELAELEKGLESAKTTFEEERRALEDKISNLEIALASLAKR-EEAVSERESSVLKKEQELLVAEEKIATKESELIQKVLANQEV-ILRKRKSDVEAELESKCKLVEDEIESKRRAWELREVDIRQ-REDLVGEKEHDLEVQSRATAEKEKDITERSYNLDEKEKNLN-AREKDINLKTTLLENEKERLKQLDLDLQQSLMSLEEKRKRV-DCATRKLEALKSETSDLSFLEMNLKKELDDLRAHKLELLA-EADRLKVEKAKFEAEWEHIDVKREELRKEAEYITRQREAF-SMYLKEERDNIREERDALRNQHKNDVEALNREREEFMN-KMVEEHSEWLSKIQRERADFLLGIEMQKRELEYCIETKRE-ELENSSRDREKVFEQEKKLEEERIQSLKESSEKELEHVQV-ELKRLDAERLEIKLDRERREREWAELKDSVEELKVQREKL-ETQRHMLRAEREEIRREVEELKKLENLKVTLDDMSMAKM-QLSNLERSWEKVSALKQKVVTRDDELYFQNGVSTVSNS-DDGYNSFMERQNGSTPSSGTPFSWIKRCTNLIFKASPEK-SPPMDPHQEGGLPLENLKLDSSRREERAYTEGLSIAVER-LEAGRKRRGNTGRDTSGPSSNKKRKHDDVTQKPKPSDE-TDPHSVISSPQNVPEDKHELPSSQTQTPSGMVVISETVKIT-KVTCETEVINKVTNIDCSENPSEAGTTMVEEQHDSGCNETVVNVSETVTRKEAE-SDNRKEQDSDDGGVVA |
| NUP-1: | | | |
| Paratrypa-nosoma confusum | Trypano-somatida | Non-public short-read genome assembly, kindly shared by Prof Julius Lukes, Ceske Budejovice, Czech Re-public | MITDSDLHRAGMFFTRARATGDGDGPQRASQGAHGHGAAGSSHLGGPRRGSMR-FVASVEAAPWLAQQDAAGASAAAAAHRDRRPVRDRSGSEHASQVAAAASET-PLAHSQPLRGPPFMYQALAGLSQTTTPGQRDPRTDAGESSHDHRLPS-GRSTAASAGLDTGSPGVGATRTGHAAGRASFHNFSEFRPSALEDMLTSDELRS-YASRLEDNVGIASDNLQQAFSLRDSYKDEAARLRAELDHQRLLRKRYDRDIERY-KEISSSQQRDIAFLKQKLDEANGTVRLLQGQLDAAHDLPSVKYKEEIRLRDASLE-HATNTISELQEANHRLRTNLARVETLELELRAAQVELRESVQRNHELEAALHSSQ-RDITAKDGDITRLKHEMDRLRAVDHENTQLRSQLQQVDKHSSAQKQYIEQLLDT-LQHRSTLTVEEEARLLQHVADITKELQRAHTTLKDKCHNVDLALAMNLISQTQISL-LEAKQQDVPNRLNPHEASADDNAAHRSHDTIHNLMEYVTSLERQIVDQAKLLE-NYENPLQTKDEKEPSSNRHKQNKNKPPTTTTCSQHSDEEAADGPTDAYPNRT-SNTTISNAQDTDTQTLPALSPSEQTLHKKPSDENHSELANCLKTIQQMATERVG-MEQRLRELDATVAQLERAAEEPETVVAALATELRHAQEQLQEACARAAAAAGA-RDDALRTVDELLAQRRDTRSATPRPSKGGSGEGDAVTPADLAAATARATAAEA-EAEAARKELADALAAVDRLATXXXXXXXXXXXXXXXXXXXXXXXXXXXXXXXXX-XXXXXXXXXXXXXXXXXXXXXXXXXXXXXXXXXXXXXXXXXXXXEAEAARKEKL-VLSTDGCEKSGLFRGFDTAEVGVCNFSCFGPGCRKECFCSGCLRVVEICREVG-LFCNSEVGSCSAVNDVAAVASRFVAMRTALVDVVDGFDSRMGGDGTLRDRIRR-ADESSCTEVSTYVVGCVASLCQATDALAARVEELSSALETEREVSRQLRRGIDE-MDIGLLAEATSDADALREIGDELAGLRESLLRMRRDGGSLHESDEDARRVALLQ-DALDERSVQVVSLTRALESLAVEYELMKAECAQKDSRASDLERKVERIRDDAAA-ELGAQASSVVWLMKAALRNTGTLTAEEEAIVRSVTRDSLGVSSDERLRRSGIVT-RFSDVHGTGLTAELRDVLQQIEQLAADRAYHEECVQSLREEVAAKGAALQRALE-ALGQASDEKVTVEQVAHERSEKILTTLSTLQSEFQRMKERLDPRPSPRSSAVP-MMDLVEAKRAVSRANEGRRLAERLLDDREGELHLLRQSLAEVTAEKVRIEAELR-AVNSQARLSTHLRETAELLRPPAPRGAAPAASSQAADDPAGPPADPTLASRFSS-PFNSTSDAVAASAAAVWQQQQRAMNVLEQSHVLQLSTLHAELHLTRQEAASLR-QRLKQVAAQVREKHSLLTEANTRLEEEVKTNDRVQEELVQMCEVYEAQLQAAR-LSDASSEEALKQLRLKYDRVCSDLSAARDSLARLEREKREAEIDARSQAEEFAK-TFRNVEAQLHALRREMDAQGIQSAPAPPSGGMRPPRASALSPDATRLNDTVRD-LQEALASRDEEVSQLQQLLALREKEIAQLEADLKRAVRALETVSDGEQEALRLA-ADRLMRLQAAEVKLADILAAASAGAAGGPRFTRHRILRSSLAVRAAGQPDASG-DGAALPPAPQKPERSRADQAEAPPRDDDGAGKAALTASGSPQGAARRSRPRA-SASSVTSPTRRGRHPSKGALGARRSGVAKKRPREV |
| Trypano-soma carassii | Trypano-somatida | Non-public transcrip-tome as-sembly, kindly shared by Dr Steve Kelly, Ox-ford, UK | MFTTGDARRYSSFFSRSWTPPPDGVNGGRGLHSATPPPISPRLGAPIHVRGLVGT-DPMRYSGPVTLSAGLQANRALVGMSRDEPNMTDSPYVSPYVSTLDA-GRGGRIPDDVLNYTARLEEDVGHVNNTLDRAYHLRDHYKEEAARLHKELM-RKQQMLDSLQCDHQHCGGMILSLRQEIDSLRQLLDEAEGEVQRLKIALASDGHQG-GLSVMYCKSELVRRETEIENLQVENEKLKKDLQELQEDIFVKKRVSDTSDAKSNVD-ETQQSEITIKALCDELRDLKSQIENERQEYEGRINSLTVERNALSAEVTSLNERLHS-TRTLCQTHADTVDELRESFIQRSSVTQQEYVVLQRRYKEIQEELESQRHSDTQNX-XXSNLESTLKLENQNLQLQIIILRQSEMEKEELLHRLQKDLDKQQSLSQNLQNELDL-LHKTHALQKTHLLDTEKKLIQSQKDNEEANRVLTDFRRRLESDCSTVNLAAGRSEQ-RGYKSTISHENIEPTIRPPPPFDISKKAKSPTNESCTPQDISTNTQREYKTQSNIIHID-DEVHTLRSRLAEADALRSRLAEADDTIEKMSAERVAQSAKVDELTAMVSRLQGSSE-TTTSDMIGALTAELRDTQDRLHEAEEACRTAADLHAADAVRITELTEQLSRDERNAR-ETQSPLELQLSSALSAVDCLAAEREALASKAADLEDRVGALEDELAAARSRADGIID-EADALRSRLAXXXNNLILLLEQYANIPEDVIGALAAELRDTQDRLRVAEGERATGLA-SNRRVTMGSVERNDRGSLVEKDLRYDVSSGCVGDDLSLRSQLATALAAIRRLGED-YDTMCEKYRSSEGQVSLLKMDLLAERRSLAEVNEISVHLREEIDMKSRALAAALVA-LDRVSAEYADAEVAQEERSANLLSTFEALYAQFEATKRSLEEQRTRNAEASAAELII-LRRNVQRAEEARRRAEDHLAECELELEREHEAHLRLQREHSTLERMSRSRGDRA-SEPMSESTAVDESPNLVAPVQAEYVDHTQFLQISSLQADLMLTRRRCRQLEGIREE-MQSELERRGTVGGNDTVSAARAELRRLRGQFEQLRVEHRVLQDQHNTMLRDHD-AALKDLLEQNTRLTHELGENQNALASLAKHNHSQQPHHPLNAPTRHESEALDRAF-RVLDAQMHVLREEVRHSTRTVSQPRRSASAPRRSAPPDNARVAALQQAVRRGEE-EIQRVQEELERREEEVDALEEELVSATERQNSSKRSPLHYEEIVEQLQNANEGPQE-SLRQVSVQRLTHVSQSASVSRRIRDTRISGREDSQGNIATSSSRPTEYRKRSRSSVR |

| | | | |
|---|---|---|---|
| Trypanosoma grayi | Trypanosomatida | Tritrypdb.org\|<br>Tgr_285_V1 | MFSADDARRYPGFFTRTWSAPPDDIRRSRSSVALTPPLLSPHLAAPIHVRGLAATD-<br>PISHRDRTVSPAATSTTAAAAAAAGMQASRVLVGMSRDEMALSPHMSLLDVS-<br>GAVRRTAVLHNRPGWEEMSQAERMDYTARLEGDVSQAKDALDRAYHLRDHY-<br>KEEAARLHRELMRKHGSLERIERDHQLCGDVIYRHQMEAQELRHQLDEANG-<br>EVQRLKALLHTTEEHRGGLSVIEYGVGLEQRQAEVNELREENRRLLDQLRSIQ-<br>DELSNERSTARERETTAIHSTTTRTAGEEEELKETRHVLQVLRAEMQDLRQQ-<br>MQQERLDHESSLEEVIKQRNSLQGLNTSLREEMEGLQTLCRAQNMTNEELQE-<br>TLLQRASVTHHEYHALNRRCEELQKHVELLQQEQSPSLDTTLELEKQTLQEQL-<br>LTLRREGEEKNEIIRKSNAEIDTMRQFTSNLEVELDLSRRAKTLIEQDLLVTKEQ-<br>LLSLQARCQESDNVIAAFHVRLEGQMSTIQALNDQLRHLQRATEKDTRSNIAL-<br>ASLCTLQNPTELTSDPLSISREGLMQPTTESSLSEAMISRDTKTQHLMQHNPE-<br>EQQKQKTSSGTEKDDNPLQLQQSLTESRQKKFGLEDRVAALEKELEDAAAAR-<br>XXXLEDRVAALEKELEDAAAARDASDADKAALSERLTSALAAVDELAAERNAQ-<br>TAKVAELTDAVARLESSGDAPARAVEALAAELRETQDRLREAEDELRAAAEXX-<br>XRETQDRLREAEDELRAAAELRKAAALGRFADEREILNDRAAGLVDRVAALEE-<br>ELDETSIRASVDSSTVVRLREELNVKSRLLADVLAALDRLTVEHTGPEVSYDER-<br>TENLLKTFEALQQQFEVTKKTFDEQWERKSEACTAELIAARRSAQRAIEARKR-<br>AEERAAQCARDLEREHSELLRVQRECRALERGVRIQSRVLTSHDEVFEDTDV-<br>IQSRRRVSNAEGLDLAQFLQISSLQAELMLARRHIRQLEARQEELQLVLEKTE-<br>WELASLPASAEESIVELRWVRAQHKDLQVEHRRLQEKFDALKNQRAEELEE-<br>MRQQNERLMQQLRERRDKLASVSRQMRESELAAKEQAEELSQAFNLLDTQ-<br>MRALRAEVGAPQGSRASPRRHSTKEPEDAQGKLLQGRITFLEHTLKQKEAEI-<br>HHLQQELERKEEQLDNLEEQVGSATRNVESTTRRSALLEKTLEQLRRTNDDL-<br>QDELRVVKGRVLAQAERLSSQPQPSRDTSRLPSRRLQSTSATATITRTKEVSH-<br>AASTRSHSRERQSIQEDEHQTPQRRTPRATSDRKRSRSGTHN |
| Trypanosoma cruzi | Trypanosomatida | NCBI\|<br>AAS44545 | MFNANDARRYPGFFTRTWTPPPDVFSLRNARSRRSGCATFAHATPPPLTPRLAT-<br>PIGVRGLAATDNLGRLGLSQALPEDVSGSRALVGMSREEPDISPHISTLDVGGS-<br>GRFTATHASRHGWEDMPRGERMNYAARLEDDVAQMQNALDRAYHLRDHY-<br>KEEAARVKNELMQKQRALDCLERDHRQCGEVICRHEREAVDLRHRLDCAEG-<br>EVQRLKYLQRSEDPNVRVPLLDAQRELRLKQTEVDSLLEEKRQLEEHVRRLT-<br>LEHSGEHYAARVHEGAVPRPPNVAHVEEGGEELTRAQHVIQVLCTELQDMR-<br>QQLQNERCRNEGTLDEAIQQRNGLQGVNTSLREELEGLKEMCCSQNRTIDEL-<br>RDTLLQRAPVTHHEHYALVRRCEKLKEEIERCQTRGGDSVNKAHELEKQAIQQ-<br>QLLVLRADGEETNTLLLKTRADVADMREYISTLETELDLTRKAKTLLEEELAAAK-<br>EKLQYATEQNKEAEQVIVNFNRRLQDQFSTIQVQKAQIDLLQQEATRASVAITP-<br>SKAAPVNSRPEGGNGDKETKNGMPPETPGVAETTMATQHKEAQAVRETVQA-<br>VSAENVKSQVEDRGKAHKTEGEVPQFPQQLSAAHAAIDQLSAERSAQAERVA-<br>ELTAAVARLESSADAPERVVEALSAELRETQDRLRQAEEEASQLAERIGSSEG-<br>LRESAVATRSAETSLAAQLSSALKALEQLAEERETLARGSAELEERVRELEQE-<br>LREAQTAHDVDSSDGLTLREQLAAAHAAIDQLSAERSAQAERVAELTAAVARL-<br>ESSADAPERVVEALSAELRETQDRLRQAEEEASQLAQRIGSSEGLRESAVATR-<br>SAETSLAVRLSSALKALEQLAEERETLARGSAELEKRVRELEQELRDAQTAHD-<br>VDSSDGLTLREQLAAAHAAIDQLSAERSAQAERVAELTAAVARLESSADAPERV-<br>VEALSAELRETQDRLRQAEEEASQLAERIGSSEGLRESAVATRSAETSLAARL-<br>SSALKALEQLAEERETLARGSAELEERVRELEQALRDAQTAHDVDSSDGLTLR-<br>EQLAAAHAAIDQLSAERSAQAERVAELTAAVARLESSADAPERVVEALSAELR-<br>ETQDRLRQAEEEASQLAERIGSSEGLRESAVATRSAETSLAAQLSSALKALEQ-<br>LAEERETLARGSAELEERVRELEQELREAQTAHDVDSSDGLTLREQLAAAHAA-<br>IDQLSAERSAQAERVAELTAAVARLESSADAPERVVEALSAELRETQDRLRQA-<br>EEEASQLAERIGSSEGLRESAVATRSAETSLAAQLSSALKALEQLAEERETLA-<br>RGSAELEERVRELEQELREAQTAHDVDSSDGLTLREQLAAAHAAIDQLSAER-<br>SAQAERVAELTAAVARLESSADAPERVVEALSAELRETQDRLRQAEEEASQLA-<br>ERIGSSEGLRESAVATRSAETSLAAQLSSALKALEQLAEERETLARGSAELEER-<br>VRELEQELREAQTAHDVDSSDGLTLREQLAAAHAAIDQLSAERSAQAERVAEL-<br>TAAVARLESSADAPERVVEALSAELRETQDRLRQAEEEASQLAERIGSSEGLR-<br>ESAVATRSAETSLAAQLSSALKALEQLAEERETLARGSAELEERVRELEQELR-<br>EAQTAHDVDSSDGLTLREQLAAAHAAIDQLSAERSAQAERVAELTAAVARLES-<br>SADAPERVVEALSAELRETQDRLRQAEEEASQLAERIGSSEGLRESAVATRSA-<br>ETSLAAQLSSALKALEQLAEERETLARGSAELEERVRELEQELREAQTAHDVD-<br>SSDGLTLREQLAAAHAAIDQLSAERSAQAERVAELTAAVARLESSADAPERVVE-<br>ALSAELRETQDRLRQAEEEASQLAERIGSSEGLRESAVATRSAETSLAAQLSS-<br>ALKALEQLAEERETLARGSAELEERVRELEQELREAQTAHDVDSSDGLTLREQ-<br>LAAAHAAIDQLSAERSAQAERVAELTAAVARLESSADAPERVVEALSAELRET-<br>QDRLRQAEEEASQLAERIGSSEGLRESAVATRSAETSLAAQLSSALKALEQLA-<br>EERETLARGSAELEERVRELEQELREAQTAHDVDSSDGLTLREQLAAAHAAID-<br>QLSAERSAQAERVAELTAAVARLESSADAPERVVEALSAELRETQDRLRQAEE-<br>EASQLAERIGSSEGLRESAVATRSAETSLAAQLSSALKALEQLAEERETLARGS-<br>AELEERVRELEQELREAQTAHDVDSSDGLTLREQLAAAHAAIDQLSAERSAQA-<br>ERVAELTAAVARLESSADAPERVVEALSAELRETQDRLRQAEEEASQLAERIG-<br>SSEGLRESAVATRSAETSLAAQLSSALKTLEQLAEERETLARGSAGLEERVRE-<br>LEQALRDAQTAHDVDSSDGLTLREQLAAAHAAIDQLSAERSAQAERVAELTAA-<br>VARLESSADAPERVVEALSAELRETQDRLRQAEEEASQLAERIGSSEGLRESA-<br>VATRSAETSLAARLSSALKALEQLAEERETLARGSAELEERVRELEQALRDAQ-<br>TAHDVDSSDGLTLREQLAAAHAAIDQLSAERSAQAERVAELTAAVARLESSAD-<br>APERVVEALSAELRETQDRLRQAEEEASQLAERIDSSEGLRESAVATRSAETS-<br>LAVRLSSALKALEQLAEERETLARGSAELEERVRELEQALRDAENSLCVREGE-<br>LLKLGEECSAKSALLSNALAAVDQVVQEHASAETSFEKQTSSLVKTFEVLHGQF-<br>LLTKKGFEEQWERRAEACTAELLAARRSAQRANEARKRAEENAAICMRKVEE-<br>VNAELMKAQRDYRALERLLRSSGRLSISRVENNDGEEHEREMSFTPTFLRNT-<br>EGAELAQFLQISSLQADLMLSRSTCHRLEARQLELQTALEQCEWRLASLPPG-<br>VEESHAELLRVRGQLENHQLEYHRLEGRYKALKRQRAEEVEQLQRQNERLV-<br>QQLREKRDKIGSLSRQMRDSEVAAKQQAEELAQAFNLLETQMQALREEVEG-<br>GQPRGRQARRQGVEETTEAEGKMLRGRITFLEHTLRQKETEMHRLQEELGR-<br>NEAQLDQFEDQVTVATQRLENTARRSTHLEQKVHELKRTNQELREELGAVRA-<br>CVVVQAERRSPQRAPSTDVLREALHGGRASSLASTPTQGEREGRAASALPRAA-<br>TKRPRAIDASS |
| Trypanosoma theileri | Trypanosomatida | Non-public transcriptome assembly, kindly shared by Dr Steve Kelly, Oxford, UK | MFSTNDARRYPGFFTRTWTPPPEGIGNRSNNIVFTHGTTPPLTPRLVTPISVR-<br>GLAATDPLGRLTHSMPTAADVQANRALVGISREEPNISPHISTLDVNASARIKDTQR-<br>CRSGWDDMTPEERMDYTARLESDMSNVKNTLDRAYHLRDHYKEEAARL-<br>RGEVMQKQALFDRLERDHRKCSEVIRSHQLEVQELRRQLDLSEGEVYRLKQVLQ-<br>SKGENEFFSPMDYERRFSQNETEMARLQEENRSLENQIQNLREELKAKSSLTVQK-<br>TTKSVQVDILDESLQKMGQYEEKEEQLAQSQHLIQVLRAEMQDLRQQLQKERLDN-<br>ESSLNELITQRNSTQSINTSLREEKEALDDLCRRQNKEIQELREDLLQRAPVSHQD-<br>YHALTKRYEELKEEIEHLKEKHDNSLDIALELEKQTLQQQLFNLRHEGEEKERLLQN-<br>SLKDASRLRELNENFQKELELSRMTKELLEKELSKTKKELQSTEQSNREAENIIEDL-<br>NKRVSTQLSTINQLTDRLNYIQHTTHQVKNYDEEQQSTQGIYGTTTSATTEIRTDTE-<br>IMSQKTTQTTIVKHPTKQTKVTESIQKSTDAKAVTEEQTQIPTSTAITEDITRLQMEL-<br>QNACNTRKDLEIENADLRERLSAXXXXXXXXXXXXXXXXXXXXXXXXXXXXXXXXXX-<br>XXXXXXXXXXXXXXXXXXXXXXXXXXXXXXXXXXXXXXXXXXXXXXXXXXXXXXXX-<br>XXXXXXXXXXXXXXXXXXXXDLRERLAAAMASIDQLAEERNVQAAKVAELTDAVSR-<br>LESTADAPALAVEALAAELRETQDRLREAEAESRAAAERHVAMTVELTERLEVAET-<br>QLRTRDTERRQSATADREAAEAAREHDAALSEQLSSALTALERLAEEREEAAEKAL-<br>QMEACIASLEESLHELRMLLSESRNEALSLREDLDVKGKLLSDALSALDRVGADHA-<br>NSEIEWGERSERFLRTFEELQSQFAVTKKGFEEQWQRRSEACTAELIAARRSAHR-<br>ATEARKRAEERAALCMQEWEQMHGELLRLQREYRVVERIVRAHGLYSTPHVDDID-<br>ESGETPPGVRPWNSEGVDLAQFLQISSLQADLMLSRKKCRLLESRQEELQAALER-<br>SELQLASLPASVEESHIELRRVRDQHETLLHEHQRLEEKYEAVKKQRIEEVEQLRQ-<br>QSERLMQQLREKRDKLTTLTRHMRESELSAKQQAEELSDAFNVLDGQMRLLRSEV-<br>ESNSRQSRSSRQHNEKVVADAETKMLQGRIKFLQRTLQQKEEEIRRLQEELERKE-<br>ELLDSFEDQVTSATHDLENTSRKSAQLEQTVHHLKRNNLELQEEIRILKSRATVHSE-<br>QQNTPTSSGLRVAVQRSSYVSRVTSTTTTPVRGTDVVRDLTPSSRGSSIEGQQE-<br>HQTESERRGRKRSRSTKRR |

| | | | |
|---|---|---|---|
| Trypano-<br>soma vivax | Trypano-<br>somatida | NCBI\| com-<br>pound of:<br>CCC46700<br>and<br>CCD21021 | MFTAGDARRYPGFFTRTWTPPPEGVSGVRGNSTSDSGIRYATPPLLTPRLAPPI-<br>HVRGLTSSSPVVPLNSPPSASSQGLLTSRALVGMSREEPCISPNVSTLNAGDTESF-<br>STIRQCRRAVTEMSREEQSNYIMRLEEDIRQMSNALHSVYQVRD-<br>HYKGEAARLHKELMDRQRSFDRLEQEHKNCRDVIHQQRNTEQELRERLDAS-<br>QTEVRYLKSLVGSSGAQQKATCANQAHEIEKKREEILELQRQNKALEVQLGGI-<br>QGQLSKEQTLAFQQQSEINASVAAWERGRRELKDTENLIKVMGAEMNDLREQ-<br>IQKERVQHEESLWAVTQERNNLQMLKMSLAEEVDRLRELCQAQSGTIDSLNET-<br>ILKRPALPVNSDHDEEAVQNTIANLNNEGQSLIRDRAVETTSDLEKQTLLHSMC-<br>ALRQDWEDSNSALQRAHIEMAQFKKHITTLEDEISTSKQEIANYEQKLIEAQIQ-<br>LSEAKETSRESESVIRDFKKMHEDQNATIYELREQIKIFQLESKGTNYSETGAH-<br>DDNKIKQNSSQGYTERQRTDMAKEHMKRQDGSHIENTEAQKKQTEVAQQKL-<br>CAELRETQARLREAEEQVARLTAEANAGATAASGSESASAAVDVRSTAEYVSV-<br>ASQLSAALAALDKLAEEREAMEHQAAEHHARVLELEEQARVWREARSREVD-<br>ADSVQQQLDRALATISEMAAERDAHAAKLAELSDTVSRLEGVETVPEHAVEAL-<br>CVELRETQARLREAEEQVARLTAEANAGATAASGSESASAAVDVRSTAEYVSV-<br>ASQLSAALAALDKLAEEREAMEHQAAEHHARVLELEEQARVWREARSREVD-<br>ADSVQQQLDRALATISEMAAERDAHAAKLAELSDTVSRLEGVETVPEHAVEAL-<br>CAELRETQARLREAEEQVARLTAEANAGATAASGSESASAAVDVRSTAEYVSV-<br>ASQLSAALAALDKLAEEREAMEHQAAEHHARVLELEEQARVWREARSREVD-<br>ANSVQQQLDRALATISEMAAERDAHAAKLAELSDTASRLEGVETVPEHAVEAL-<br>CVELRETQARLREAEEQVARLTAEANAGATAARGSKSASAAAERDAHAAKLAE-<br>LSDTVSRLEGVETVPEHAVEALCVELRETQARLREAEEQVARLTAEANAGATA-<br>ASGSESASAAVDVRSTAEYVSVASQLSAALAALDKLAEEREAMEHQAAEHHA-<br>RVLELEEQARVWREARSREVDADSVQQQLDRALATISEMAAERDAHAAKLAE-<br>LSDTVSRLEGVETVPEHAVEALCAELRETQARLREAEEQVARLTAEANAGATA-<br>ASGSESASAAVDVRSTAEYVSVASQLSAALAALDKLAEEREALDQRVVELSSD-<br>LERVQSDKTSYYLSMVDLNNEVSDLRLQLKNNDVAVTEILSCLGDVSAECRG-<br>GNSSGDSSALLKAIEDLYSRLSSAKRNFELYSTCNSETGVTELIAARRSADRM-<br>NTARRHAXXXXXXXXMNQECLFLRRELRLHGICFSYNSLDVEGSFASRLSAA-<br>NVNDECSDETQLLQISSLQAELLLCRKRCRRFEVNQEELLTTLEHHVSQAEVS-<br>ATNMAELRLDLAQLRLQFTQLQEEHSGLQDRFEDVNHSQLDELDRLRLQNDR-<br>LLCDLKEKSEKISAMEKEMRDAERSAKGRVNELLESLCIMEAQMGDLLKGAG-<br>SSVDSSESQQSDRVQGRALAARVSFLEQTSKRKEEEIQRLQDELLRKEDQLD-<br>QLEQRVTDSTQELDNAVKKVTHMESVVQRLQSAKDSLQEENRLLRAREVALT-<br>QHPSPSTAHVAELGALNQRPPRTGSAATVNMRTTRDNTAVVVPTALPSV-<br>SVGRRSTPTARTSGLTQARKRPRS |
| Trypano-<br>soma con-<br>golense | Trypano-<br>somatida | NCBI\| CC-<br>C89495 | MFSAGDARRYPGFFTRTWAPPPDSPGDIKNNRSSSIPQTGISHETPPLLTPRLAP-<br>PINVRGLAASDPMSRVRGSLSSPAGLLPDHALVGLAREEPSFSGYMCR-<br>RGLNDMSHEERLEYTSQLDGDMKHVSSALDRAHQL-<br>RDEYKNEAARLHRELMEKQRSLDRVVREYGECNKVLHRFRTENEE-<br>LQQKLDQSQGEVKHLRSVIQSANVQQELKSIGAGQLLGDKGNQERI-<br>QLEESNRRLERQLQELTEQLEKEQAYNRRWKNSNETATTTDQDSLA-<br>VRVEQGRLSLNVAMAEIDDLKNELQRKNDEYSRRLEQLTNERNSLHQ-<br>GHLTLSETLEKLEDTCKEQQQTIEDLTVRLLQEQSTNRKERNSHNEQ-<br>HIGYQHVHSEENTDQLRAKLCAIELEKHALQQQLFHLRQEGERKDEII-<br>KKANTDIRKLKDSETAMSRKIGEVWKQNEKLQERIEETTRQLHISQN-<br>QNEEDQYVIKNFHKRLEEQIKLTHNTQTENNTTENQSDSQKSRKQER-<br>QTTEIKKNQELGKKTTAEHENFPRETETQKHINKENECTTATITKERD-<br>AAVGAPSSDLATQLSSALAALNRLAEEREAVSERAAEMEDRLGVLED-<br>ELLKARTALDASSGNEEALKEQLEQALEATESLAAERNAHATKLKELE-<br>GTIARLEGCGDSSAKVIEALKSELAHTQKRLREAEQDCARLTRTDAV-<br>VKERDAAVGAPSSDLATQLSSALAALNRLAEEREAVSERAAEMEDRL-<br>GVLEDELLKARTALDASSGNEEALKEQLEQALEATESLAAERNAHATK-<br>LKELEGTIARLEGCGDSSAKVIEALKSELAHTQKRLREAEQDCARLT-<br>RTDAVVKERDAAVGAPSSDLATQLSSALAALNRLAEEREAVSERAA-<br>EMEDRLGVLEDELLKARTALDASSGNEEALKEQLEQALEATESLAAE-<br>RNAHATKLKELEGTIARLEGCGDSSAKVIEALKSELAHTQKRLREAE-<br>QDCARLTRTDAVVKERDAAVGAPSSDLATQLSSALAALNRLAEERE-<br>AVSERAAEMEDRLGVLEDELLKARTALDASSGNEEALKEQLEQALEA-<br>TESLAAERNAHATKLKELEGTIARLEGCGDSSAKVIEALKSELAHTQ-<br>KRLREAEQDCARLTRTDAVVKERDAAVGAPSSDLATQLSSALAALNR-<br>LEEEHEAVSERAAEMEDRLGVLEDELLKACTALDASSGNEEALKEQL-<br>EQALEATESLAAERNAHATKLKELEGTIARLEGCGDSSAKVIEALKSE-<br>LAHTQKRLREAEQDCARLTRTDAVVKERDAAVGAPSSDLATQLSSAL-<br>AALNRLAEEREAVSERAAEMEDRLGVLEDELLKARTALDASSGNEEA-<br>LKEQLEQALEATESLAAERNAHATKLKELEGTIARLEGCGDSSAKVIE-<br>ALKSELAHTQKRLREAEQDCARLTRTDAVVKERDAAVGAPSSDLATQ-<br>LSSALAALNRLAEEREAVSERAAEMEDRLGVLEDELLKARTALDASS-<br>GNEEALKEQLEQALEATESLAAERNAHATKLKELEGTIARLEGCGDSS-<br>AKVIEALKSELAHTQKRLREAEQDCARLTRTDAVVKERDAAVGAPSS-<br>DLATQLSSALAALNRLAEEREIALEEDAFEGPIVDVLKRDILFLKSSLG-<br>ECRRYISGLNDLNLLSCCDDGSSSMVNLFNRVGGGLSDDLPNKCDG-<br>TATLGATRVMQLGSDCVDEVSCVADLVAARRSLQRSNDARRRLEERN-<br>IELERELEKRSMDIAGLEKECRDLEKQVRARGSSYMRSIHDECDFR-<br>SSVSGGVCMETIDLAQFLQISSLQADLMLSRRTCRQLESNQEELLL-<br>SVEQGVAQSQNLLESVEELRMEVHNLRRARDELLCERRLLTERVND-<br>LELEREEDVEVLRKQNRWLTSQIEEAGDKLSAAEKSRREAELAWEKQ-<br>AEELIKAFNDLNAQMTHLRSEGAPRSRNSLSRSLSRSAVDSEDETLN-<br>QVRNARISFLETTILRKDGDIYRLQEELIKKEDNIDKLQREIIRMSNKIK-<br>NATRKSEKLESNLQQLHEKKEEMNKELYLLKTKAPQKKIVQPRFSTVT-<br>NQQTPETINTPSKRTSLVIIEDSDKQLHDISTGRRKAQTL-<br>SKEIKQEKIPKRESRKRGRN |

| | | | |
|---|---|---|---|
| Trypano-<br>soma bru-<br>cei | Trypano-<br>somatida | NCBI\|<br>XP_951625 | MFSAGDARRYPGFFTRTWTPPPENIGHVRSNRSASSIQGGLTHETPPLLTPRLAAP-<br>INVRGLAATDSIPRLNNPLPSPTGLLTNSALVGMSREEPSIMPLLSSKQTAPMGMHP-<br>TIFAGRRGLADMSEEERMEYTNRLEGDMTHVHNTLSRAYQLRD-<br>DYKNEAARLHRELQDKNHRFDCLLREHSACNDVIYRCKRENEELRQKLDESEG-<br>EVRQLRDKLVSVNSQGKYVPSGGERHVGRQEISALEEKNKKLEEELLELTKELE-<br>RERECIRHHAVAAEMGKSENTSHEEELAQSRYLLQVTRTEITDLQQLLRKERED-<br>YEESLREAIQARNNLHQQNTALQEQKEQLQEMCDEQHRTIEDLTSQLLQRKTE-<br>QAVQRGAPDTQMETTDENKTDTNTNNDDEVYRMLELQQHTLQQQFFLLRREG-<br>EAKDILLQKASEEIFNLQNLQQQLEAALQKSREHAAELTKSLSHTQNQLQTAQER-<br>ITEDSYVINNFHHQLREKIQISGSISGEKNIPQGGNKEESIELVTRETQMPSRSG-<br>NDSQYITANVQHEKLNQPQKADSGHNATGNNKELSSAQNDEYEQAIIKHKMTE-<br>EGLTEVIEALKTELQHTQKCLREAGEENVQLTNKLNAAGARGRSTSTTRSGSLT-<br>PNDTEGSLRTYNAGLKTQLSSALAALTQLAEQHDATLARATEMEERVSTLEEEL-<br>RTAHSTTKKMSAERELHVTKLTQLEETVSRLESYGTTPEQTVAAFTTELQHTQQ-<br>RLREAEEEIIQLTNKLNAAGVRVRTSQSDKDGNARAALVSDVAVRNADTDLGTQ-<br>LASALVALERLAEEREAALEKATEMEERVSTLEEELRTAHSTTKKMSAERELHVT-<br>KLTQLEETVSRLESYGTTPEQTVAAFTTELQHTQQRLREAEEEIIQLTNKLNAAG-<br>VRVRTSQSDKDGNARAALVSDVAVRNADTDLGTQLASALVALERLAEEREAALE-<br>KATEMEERVSTLEEELRTAHSTTKKMSAERELHVTKLTQLEETVSRLESYGTTP-<br>EQTVAAFTTELQHTQQRLREAEQEIIQLTNKLNAAGVRVRTSQSDKDGNARAAL-<br>VSDVAVRNADTDLGTQLASALVALERLAEEREAALEKATEMEERVSTLEEELRTA-<br>HSTTKKMSAERELHVTKLTQLEETVSRLESYGTTPEQTVAAFTTELQHTQQRLR-<br>EAEEEIIQLTNKLNAAGVRVRTSQSDKDGNARAALVSDVAVRNADTDLGTQLASA-<br>LVALERLAEEREAALEKATEMEERVSTLEEELRTAHSTTKKMSAERELHVTKLTQ-<br>LKETVSRLESYGTTPEQTVAAFTTELQHTQQRLREAEEEIIQLTNKLNAAGVRVR-<br>TSQSDKDGNARAALVSDVAVRNADTDLGTQLASALVALERLAEEREAALEKATE-<br>MEDRVSTLEEELRTAHSTTKKMSAERELHVTKLTQLEETVSRLESYGTTPEQTV-<br>AAFTTELQHTQQRLREAEEEIIQLTNKLNAAGVRVRTSQSDKDGNARAALVSDV-<br>AVRNADTDLGTQLASALVALERLAEEREAALEKATEMEERVSTLEEELRTAHST-<br>TKKMSAERELHVTKLTQLEETVSRLESYGTTPEQTVAAFTTELQHTQQRLREAEE-<br>EIIQLTNKLNAAGVRVRTSQSDKDGNARAALVSDVAVRNADTDLGTQLASALVAL-<br>ERLAEEREAALEKATEMEERVSTLEEELRTAHSTTKKMSAERELHVTKLTQLEE-<br>TVSRLESYGTTPEQTVAAFTTELQHTQQRLREAEEEIIQLTNKLNAAGVRVRTS-<br>QSDKDGNARAALVSDVAVRNADTDLGTQLASALVALERLAEEREAALEKATEME-<br>ERVSTLEEELRTAHSTTKKMSAERELHVTKLTQLEETVSRLESYGTTPEQTVAAF-<br>TTELQHTQQRLREAEEEIIQLTNKLNAAGVRVRTSQSDKDGNARAALVSDVAVR-<br>NADTDLGTQLASALVALERLAEEREAALEKATEMEERVSTLEEELRTAHSTTKK-<br>MSAERELHVTKLTQLEETVSRLESYGTTPEQTVAAFTTELQHTQQRLREAEEEII-<br>QLTNKLNAAGVRVRTSQSDKDGNARAALVSDVAVRNADTDLGTQLASALVALER-<br>LAEEREAALEKATEMEERVSTLEEELRTAHSTTKKMSAERELHVTKLTQLEETVS-<br>RLESYGTTPEQTVAAFTTELQHTQQRLREAEEEIIQLTNKLNAAGVRVRTSQSD-<br>KDGNARAALVSDVAVRNADTDLGTQLASALVALERLAEEREAALEKATEMEERV-<br>STLEEELRTAHSTTKKMSAERELHVTKLTQLEETVSRLESYGTTPEQTVAAFTTE-<br>LQHTQQRLREAEEEIIQLTNKLNAAGVRVRTSQSDKDGNARAALVSDVAVRNAD-<br>TDLGTQLASALVALERLAEEREAALEKATEMEERVSTLEEELRTAHSTTKKMSAE-<br>RELHVTKLTQLEETVSRLESYGTTPEQTVAAFTTELQHTQQRLREAEEEIIQLTN-<br>KLNAAGVRVRTSQSDKDGNARAALVSDVAVRNADTDLGTQLASALVALERLAE-<br>EREAALEKATEMEERVSTLEEELRTAHSTTKKMSAERELHVTKLTQLEETVSRL-<br>ESYGTTPEQTVAAFTTELQHTQQRLREAEEEIIQLTNKLNAAGVRVRTSQSDKD-<br>GNARAALVSDVAVRNADTDLGTQLASALVALERLAEEREAALEKATEMEERVST-<br>LEEELRTAHSTTKKMSAERELHVTKLTQLEETVSRLESYGTTPEQTVAAFTTEL-<br>QHTQQRLREAEEEIIQLTNKLNAAGVRVRTSQSDKDGNARAALVSDVAVRNADT-<br>DLGTQLASALVALERLAEEREAALEKATEMEERVSTLEEELRTAHSTTKKMSAE-<br>RELHVTKLTQLEETVSRLESYGTTPEQTVAAFTTELQHTQQRLREAEEEIIQLTN-<br>KLNAAGVRVRTSQSDKDGNARAALVSDVAVRNADTDLGTQLASALVALERLAE-<br>EREAALEKATEMEERVSTLEEELRTAHSTTKKMSAERELHVTKLTQLEETVSRL-<br>ESYGTTPEQTVAAFTTELQHTQQRLREAEEEIIQLTNKLNAAGVRVRTSQSDKD-<br>GNARAALVSDVAVRNADTDLGTQLASALVALERLAEEREAALEKATEMEERVST-<br>LEEELRTAKEKLERSVEEISFLKDEVLVSNRLLVDSVSSLNGKVGDSDGAVGADV-<br>ERLSRVVDELHAQVSATKRGFEEFYDRRSEGCVTELIVARRSVDRSNDARRRL-<br>EERNVRLEQDLERKCLEVVKLQKECQRLEQFVRAKDVRGAHSVLGVDGSVDV-<br>SSVGAEPVDLEAVDLAQFLQISSLHADLMLCRKTCRQLESNQEELLLSLEQNSS-<br>QSNAYLEDLDEIRQQLVEMRQQREELIAERRTLTERVDELGRERGEEVSRLKQ-<br>QNNSLSAQLQASRNKLSALEASKREGELAARQQAEELAKAFSLMEAQVQTLRE-<br>EVASTSGSPKRQSGSSRQKAVVEGDEARIRMSQARVTFLEKALQRKDEEVQR-<br>LQDELVQKDEQLDQYEQDAAKAAQDAENASRKTLQLESAVQKLQGDKKGLED-<br>ELRYAKTRVVTYGGRVSSEVAQHSSPPEQQIRGSPVLGAGRTTRERVSLSVES-<br>SHHSRITEQTQRQVRQVMDIRSTRKRSRSANAVS |

| | | | |
|---|---|---|---|
| Angomonas deanei | Trypano-<br>somatida | NCBI\| com-<br>pound of:<br>AUXM0100<br>0739 and<br>AUXM0100<br>0607 | MSSNWGNRGYNTSVNNYKSSFDILREQRLAAQNQSTPSAGRETPSGFYDHTP-<br>SPLERSRYEVLHNPISMAVQSGIEEPPYYTRAPPTPAVR-<br>PQLDLMTRDDLYNECAKLRWSFDNAQELRERSEQGRIYYRDL-<br>VAKYQAEKDLLNSRLQSAIEAKSRAEEETGNYRRSCESLRNQLNDLEEEFRRA-<br>KLDLDRAHADLRGQFLLEGEQGGAQVVVGRMKEDLREKDACIMDLSRQNSE-<br>LEAELERLRNALSPADQAAQPPASLSEYPRSTYDNDPLARQRLEDELQAARE-<br>ELDQLRNTLDDERTAAERELQHARSESSQMGHKELENALRNSDALQETIDQL-<br>AAELAKRAPVSLSDWEDYQQKYSDLLQKNKQSESTVRQLERQLENGPTPNEE-<br>ELAINENKDQLIQTYSEEVELRAKKEEALLQELEAVSAQLQQSTKEQERLARVQ-<br>KNRIVELELSEKDLKEANKKLEEEIAEKENALVDAAAATKRLGQALDASEQEKL-<br>DLEEQLRELQKQSSDDQKADEEIRRLQAALDEMADRVRDEDIDELQRQLDER-<br>TAELEAEVQRNRDLAANGRTLETEIVELNSQVAALESRLAAKETEVGVSSQQS-<br>EAAVAGLEQELANAVDAAERLSQALDASEQEKRDLEAQLHELQKQSNNNQR-<br>AADDSAEEIRRLQAALDEMADRVRDEDIDELQRQLNERTAELEAEVAHNRDLE-<br>AQLRELQKQAGNSVRAVDDSAEEIRRLQAALDKLADRVRDVDVEELQLQLDE-<br>RTVELESEVERNRGLEAQLRELQNQTTNNQKNADDSAEEIRRLQAALDELAD-<br>RVRDEDVDELQRLLDERTAELEAEVARNRDLATNGRTLEAEIIELCSQVAALES-<br>RLAAKEAEVGVSSQQSEAAQAGLEQELANAVDAAERLSQALDASEQEKRDLE-<br>AQLYKLQKQSSDNQNAAEEIRRLQAALDEMADRVRDEDVDELQRQLNERTSE-<br>LEAEVARNRDLEEQLRELRKKSTNSQKAADDSDEEIRRLQAALDELADRVRD-<br>EDVDGLQRQLGERTAELEAEVQHNRDLAANGRTLEAEIVELNSQVAALESRLA-<br>AKETEVGVSSQQSEAAVAGLEQELANAVDAAERLSQALDASEQEKRDLEEQL-<br>RELQKEAGSSQKAADDSAEEIRRLQAALDEMADRVRDEDIDELQRQLNERTA-<br>ELDAEVARNRDLAANGRSLEAEIVELCSQVAALESRLAAKEAEVGVSSQQSEA-<br>AVVSLEQELANAVDAAERLSQALDASEQEKRVLEAQLRELQKQSNNNQKSAD-<br>DSAEEIRRLKAALDELADRVRDEDIADLSASLAQEKDTNARLATELNALKSRLA-<br>GLEETVSLRDESIRSLVSDLKACDEDRRLLDTLKKQVSDQKEVAKLQKVHADS-<br>EQEIRRLQTALDEMADRVRDEDVDELQRQLNERTSELEAEVARNRDLEEQLH-<br>ELRKKSTNNQKAADDSDEEIRRLQAALDELADRVRDEDVDELLRQLGERTVE-<br>LDAEVARNRDLAANGRSLEAEIVELCSQVAALESRLAAKEAEVGVSSQQSEAV-<br>KAGLEQELANAVDAAERLSQALDASEQEKRDLEAQLRELQKQSNNNQKSAD-<br>DSAEEIRRLKAALDELADRVRDEDIADLSASLAQEKDTNARLAAELNALKSRLA-<br>GLEETVSLRDESIRSLVLDLKACDEDLRLLDTLKKQVSDQKEVAKLQKVHADSE-<br>QEIRRLQAALDEMADRVRDEDVDELQQQLDERKAELEAEVARNRDLAANGRT-<br>LEAEIVELNSQVAALESRLAAKETEVGVSSQQSEAAVAGLEQELANAVDAAER-<br>LSQALDASEQEKRDLEEQLRELQKEAGSSQKAADDSAEEIRRLQAALDEMAD-<br>RVRDEDIDELQRQLNERTAELDAEVARNRDLAANGRSLEAEIVELCSQVAALE-<br>SRLAAKEVEVGVSSQQNEAAQAGLEQELANAVDAAERLSQALDASELEKRVL-<br>EAQLRELEKQSNNNQKSADDSAEEIRRLKAALDELADRVRDEDIADLSASLAQ-<br>EKDTNARLAAELNALKSRLAGLEETVSLRDESIRSLVSDLKACDEDIKLLGQLQ-<br>KENSVLTEKGKALSDELSKLTSTLDKAEKRLAEGQQAYAAEVKRSACLQVSLD-<br>EMAITVESTKDALQQEQEKLFKMDHLRQENDSLMAYSQKLLERSEELVALLEE-<br>KTQLCDVSVSNYNALRRSLEEECLKSVTLEGEKKDLLAEVASLTSQLEETAEQ-<br>YAEKLLDVEYEADRKAAWRKVADRRLAQLKELLADYKMSVESIVEALRYTLQV-<br>DAKKENSSALVLSHVEAALTRADALRTNLNTIAVKDDDDENDSNPAATAGDDN-<br>LENFSQETAAALTEYKESLQTISEMQVRMAQQKAAIDVLAERGYDMDEAWKRI-<br>ENDIRVETEANRRHQEHRDLLLFELESANKARERAEEARREADERATLLESD-<br>LQVMRLQQGDAASGPSSEEVEQLRSRYAQLAADYETAMNRLQLLERSGVER-<br>AGDLYQQLLETEARCQVIQRELDLYKDNDVARRAALAKSNTLAKYEEMRYSDT-<br>LHTNLDKVQSLRATFGASDAKPSGTDPLSKLQDALQRNLQLEQEQIEWKSRY-<br>ERVEEKLHAIEEENNRLSRLSGVVERLSTSQRAPTLTRSMPPPSTPSFVHMGL-<br>SPVPTPGPLAAPSPLPQQPLFSNRDVSSSDEDEEEEEQTSRRSSRSFKSPQSR-<br>KRTRSASSKR |
| Angomonas desouzai | Trypano-<br>somatida | NCBI\| com-<br>pound of:<br>AUXL01001<br>657,<br>AUXL01006<br>132,<br>AUXL01002<br>005 | MSRTTPSFAPSTAASRSPRTTPAPSPPPAPRPALETMTREDLYNECSKLRWSFD-<br>DALERRERSEQAKQYYRDLVGKYQSELERMQDQKGE-<br>FLEAKRKAEEETERYKQSCDSLRRQLEDMDEALYEARAERDRLGRDLRSQSLMN-<br>NGHRGAETVVESLKGELRERDAAVFDLSRENERLEKELEALREELRRQAASPS-<br>PTAAPVDPFLAQDRLDSEVEELRDTVEALQAKLAETQEQHAAELRQEAEKVDQ-<br>RKRELDQNKDKDMENLLKRNAELQEVTDNLATQLSKRAPISSEDWKQFQNDYD-<br>NLAKKIKSLEEAEKKATTGLDAVNRQQQDELDSYKKEVELYQQKEEALLKEISN-<br>LQAQHQSTEANLAELRRGMDEMQKEQQSLLKENEAQRKTLEETSIQTATEDAA-<br>PQLEEMENRLQALQKAVDMLATSLEQSEQTRHELEQQLHHLQNQSTVSAETIE-<br>SAKGNAEENEALRAALDVMASKVSQEDVDALKMQIAELTNELEAERENSRAME-<br>TEFASLQAELAAKDAEVGVTSQQSEENQRHLEEDLQSANATADRLGEALAESE-<br>EQRLDLQRQLDELQQRLADAKKQADEAKGSTDEVEALRAALDVMASKVREED-<br>VARLEKLLADRTNELEAERDHSRSLNENGRALEAEIVELASQIXXXXXXXXXVAR-<br>LEKLLADRTNELEAERDHSRSLNENGRALEAEIVELASQIASLQAELAAKDAEVG-<br>ATSQQSEENQRHLEEDLQSANATADRLGEALAESEEQRLDLQRQLDELQQRLA-<br>DAKKHADEAKGSTDEVEALRAALDVMASKVREEDVARLEKLLADRTNELEAER-<br>DHSRSLNENGRALEAEIVELASQIASLQAELAAKDAEVGATSQQSEENQRHLEE-<br>DLQSANATADRLGEALAESEEQRLDLQRQLDELQQRLADAKKHADEAKGSTDE-<br>VEALRAALDVMASKXXXXXXXXXXXXXXLADRTNELEAERDHSRSLNENGRALEA-<br>EIVELASQIASLQAELAAKDAEVGATSQQSEENQRHLEEDLQSANATADRLGEA-<br>LAESEEQRLDLQRQLDELQQRLADAKKHADEAKGSTDEVEALRAALDVMASKV-<br>REEDLAALRANYDALKEKNVELNDAVKQLRLKIGDLESTVSLRDSSIRSLVSDLK-<br>ACDDDLRKLGELQKENEGLKLSLRGEKETLSSLTSKSNASDVALKNSEEATAALK-<br>AKLVASTEAGDRLAEQVVQLQSEKADLLQQIDVLSLKDKEGVEVKKYGEELLEK-<br>SEMLATLLEQRSRECNEHLANAVSLKEVLEEECLRNATLDQELKEKESALAALK-<br>EQLEDTVALYEEKLLEVEGATEKCETWRKTAEKRFQDSYRMAKEVETCVVGLV-<br>EAVGCATDPDSLSKTKTSSALSLKNVEGAVKNIRDTLAKMDTAAAEELISPSVPA-<br>ADAFSSKMNDAVSLCKKHISEIEEMQVRLTHLTAAVDVLAERGYDMGEAWKKIE-<br>QEIQTEAEANRRNQEHRDLLLFELDSANKAREKMDEARREADERATLLESEVQ-<br>VLRLQQSDARSVSPGELDRLRARNAQLVADYEIAVDRLRVLERAGGRHGEDLY-<br>QQLVEAEARCQVLQRELDLYKDSDAERRSALAKSNTLAKYDGLRYSHSLQSNL-<br>HKMQDLRAAFDPSAPHKSTSKDPTEIAIQRNEALEQERIELQTRYSRAEEKLSV-<br>LEEENRRLAQLAGVAERLSTSRRGTLLPKDMMPPPSTPGFVDVPLSPAPTPGPY-<br>NIPSPLPQQPLFRTLPYADDDEDEEEEVSRPSTVRSNTKSPSTQQGKAKKRPRSK-<br>SSRA |

<table>
<tr><td>Strigomonas culicis</td><td>Trypanosomatida</td><td>NCBI| compound of: AUXH01001038, AUXH01002460, ATMH01010215, AUXH01000948</td><td>MFRSTYKSTSSTSSSFPYQTSRVPTEGVEGGPHPTTTAPHPTVGDLEGPISVR-<br>DAPKWGGGSSSLIPRASAGGDTSSPLSAGRRTGGDGPRPPFYNPDDDSYYRYYR-<br>PEPATGVLPPPEPGHSTHGVVRAPRDALLWATMASGREPPAFAAGPSARQECLDE-<br>FLLTAAEGDELAHKTPAEVAAYVERLRSNYHYFGEQLRAACERAQHYKSEVARL-<br>REELETRRLQKQQFDVDVEAQDGVQHALEDQVRRLRGQLEEQEGLIYSLRQEL-<br>VARSAQTSTATSQEIMWYEAQRVRAAEEARETQQALRDLAAELQSSKAQVRAL-<br>EVQQQQQQQQQQPSELARRLQDLERENAQLRQSLAAEREEHEQQKQQLEEA-<br>LAAQRAAQVQLQADTDAMALALTRRAPLSLDEYDAQQQQLAALQREVEAQTA-<br>EAAQRKAAVAPLEATALELTELRQRVAAAAHDRTTLERNCHQRDEEIVRLQELL-<br>QCYKTENLAAQEVSRVRTEQYEHTLLEQVDAAEAARHAAEEAVDDLQRQLQG-<br>ERQGLVAEVAQLRQAAADAPAPQERQDLTEARAKIATLLQQVQNLGEALEVQTA-<br>ERDALQVQLSASGATTSQEQSVQTIQEGRAAFTTEVQRSAHSDHTTETHIQHP-<br>DATSDTASSSPYSHTDRDPHQVRESDGKAPAAPKPVAELEEALRSLAAEKTALQ-<br>EQLASAGDAQAAVAALQQENAYLGAEIQRAAAVVLRLEGELKQLAALKAQKTEA-<br>DAANAALEAKVRELEAARAQADADMQQLAAARKSDEKRARKEETAAKATPKQ-<br>DEKEXXXXXXXXXXXEDALRSLAAEKTALQEQLASAGDAQAAVAALQQENAYLG-<br>AEIQRAAAVVLRLEGELKQLAALKAQKTEADAANAALEAKVRELEAARAQADA-<br>DMQQLAAARKSDEKRARKEETAAKATPKQDEKEPRESDGKAPAAPKPVAELED-<br>ALRSLAAEKTALQEQLASAGDAQAAVAALQQENAYLGAEIQRAAAVVLRLEGEL-<br>KQLAALKAQKTEADAANAALEAKVRELEAARAQADADMQQLAAARKSDEKRA-<br>RKEETAAKATPKQDEKEPRESDGKAPAAPKXXXXXXXXXQEQLASAGDAQAAV-<br>AALQQENAYLGAEIQRAAAVVLRLEGELKQLAALKAQKTEADAANAALEAKVRE-<br>LEAARAQADADMQQLAAARKSDEKRARKEETAAKATPKQDEKEPRESDGKAP-<br>AAPKPVAELEDALRSLAAEKTALQEQLASAGDAQAAVAALQQENAYLGAEIQRA-<br>AAVVLRLEGELKQLAALKAQKTEADAANAALEAKVRELEAARAQSDIDLEDLLSS-<br>YAALLADEQTLISALKELKAQSVAGETASLQWEENVAAAVRAVEERSATEASSRR-<br>ATVDSIAAELSRRTEAHVVQMIKQDQTNSGKRMREEETVAKAAKVTPKQDEKE-<br>PRESDGKAPAAPKPXXXXXXXXXEADAANAALEAKVRELEAARAQSDIDLEDLLS-<br>SYAALLADEQTLISALKELKAQSVAGETASLQWEENVAAAVRAVEERSATEASSR-<br>RATVDSIAAELSRRTEAHVVQMIKQDQTNSGKRMREEETVAKAAKVTPKQDAK-<br>EPRESDGKAPAAPKPVAELEEALRSLAAEKTALQEQLASAGDAQAAVAALQQEN-<br>AYLGAEIQRAAAVVLRLEGELKQLAALKAQKTEADAANAALEAKVRELEAAAKA-<br>AEERAASWQEELAALGKELQRQNGKEVGDREAAVSVVAGEIEKLEEEVQLLAA-<br>ECTSLQTSQEALRVDREALVATVRKMREEAEDAERVRAKSDERHALELGRMES-<br>RTRAKVAEFQAALDTLAREKVHVVETLADREQTLSALRDELKTAERRVAELEST-<br>LKQAAQETERLRMVVEERDAALREQLESYNVLHADQALLINALQQMKETTASL-<br>EGARREAEEQRVAQVHAMEERAAALEKRLLEEGDSVAGMQAMLESTMQGAA-<br>GWREAFERAEKELAEREGELQELTASTRAAAEESLMKLQSMEQLHTESLAKEV-<br>ARKEEAFTALTRVKNEMQYAKMDAEKAQREVQQQLREVEEVNAQLRQALSRL-<br>ETQREAEKANADEALRQLAVKYTAGREEVSVLTAQRTALLATEKEAASLRTKAAE-<br>LESTLRATNARMEEQEQLIEELEAALQAAADQEEALLLQVQELAERGAELAAEK-<br>AQHLLQVEALKKTLEDTATSYNDRIVVEERLRDRMEEYRQLAAARQLELRTAAA-<br>VWLQATESLRDGLRVAEAAAGSLKGPATPPLKLQPLEKAAAWYADVALRETAAE-<br>ARRSSQDLSDDDSRRASTVQGGEGTHQEEVRVLFQRNAAGLCAEVGDQLRQ-<br>LWELGRAAARREETLRRKLELEQQLRAALDTLAVENVDLKEAGRNYQSLFNET-<br>QAALQQITNDFQQQLLEEKEAASVEVVTARRDMQRAGESRRRAEQRAQLLQE-<br>ELEQLQHVKGELEGQLLAMSEERDELRTRATETVEAVLAAEPAGAGSGDGSDL-<br>SVRASRYSTAMAQEQAQVLQVTSLQSDLLLVRRQLREMEAQDAALRRSAAASE-<br>REVAALRTERVRLQRELEREHVTTQRRYSQLLEQTDAGAAAEELRELRLKVRE-<br>LEGVVFRFEERERELARVRSQQEVQAGRRQEAVLTRLRSMSSRMTDVRRQLN-<br>DGGERASRGGLTLATDPQLVAEVQQLQHDLENYEKLAVEHHLRLESLQAQLAA-<br>EREARAMADKAVRQLSRHNERLRTTMQSERQSTLLALSPFTPPPPLTGVGASP-<br>LPRTPLPPTPTPEEEEEEEEVARTPTQSRKVEDDGAPSGGRGRGSAAKAKAPA-<br>PEGGTPTQPRKSSKAGRATGSRKRMRSHSTSSS</td></tr>
<tr><td>Strigomonas galati</td><td>Trypanosomatida</td><td>NCBI| compound of: AUXN01001911, AUXN01004474, AUXN01001425</td><td>MFRSTYMSTSSTSSSFPYQTNRNPTSTGEGVYISTPQPNNNNRQEMPAFSSLEG-<br>PISVRQAPKWTGGAAPPPLRTSAGGMADVSSPASAGR-<br>RTGGGGGDNSLNPTYPQQQQQHQQPFFNPDDDSYYRYYQQQPLPPP-<br>PQQQQASASLTPRGATRPAYTSVGGGDSVVQAPHDALLWATMASGREPPVFSH-<br>PYQQQNQQQQPQRQECLDEFLLTAAEGEGLEHKTPAEVKAYVLRLRSNYHYFG-<br>DQLREACERAQHYKLEVARLREALETQRLQKQRYNLDAEAQDSVQHALEEQVR-<br>RLRGQLEEQEGLILALRQQLLARSSEQTTSTATEIMRYEAERVKASEELRSAQQG-<br>LRDLAADLQRSKAQVRALEVQQQQQQPSELALRLKELEKENAQLQESLAAERAE-<br>HEVVKQRLEESLATLQADTDTMALALTKRAPISLEEYDAQQRRLAGQQRQLEELQ-<br>VEVEQCKAAVAPLEATALELNELRQHVAAASHEKAALQREGQRKDEELTRLQELL-<br>QCYKAETDAAEEANRVRTEKYEAALVEQVEAAEAARREAEAAVAELRQQLAQAQ-<br>AQTQTQMEEGARSSQANNHQQHHTDASSSSSSSPSPTSADGTTTTTAMSGML-<br>GGAGNEAHDLRVAMGKIAVLLKQVQELGAALQTQQTEHETVQGTLEGTLEELRE-<br>ENAYLGQEIQRGADVVLRLEARLKEQEKASKQSEKSAERRASTAAAHKEEEQEG-<br>LRVAAEKVAILSRQVEELGAALEQQLTEKGELQEQLLEAESALQEQGASASQQEE-<br>ALAALKEENKYLCEEIKRGAAVVLRLEARLKELEKELEKELSKQAQQPELAASNKA-<br>LEKSNKKLEERVRELEAAQAQAEAELQALTVAREQEEQYVRELEQGVKAAEERV-<br>STSQQEQSAAVKQVHQQEQEDLRVAAEKVSVLSQQVEELGAALEQLAAEKAAL-<br>QEQAVADEKTQDKTVSALKEDNAYLSKEIKRGAAVVLRLEALLKEQEKELERRAK-<br>QPEIAAENKKLEKSNKALEERVRELETAQAQAEAEVQALTVAREQEEQHVRELEK-<br>GVKAAEERVSTSQQEQAAAVRQAQAQEQEDLRVAAEKIAILSKQVEELGAALEQL-<br>AAEKAAMQEQASADQKSQDETVSSLKKDNAYLSEEIKRGAGVVLRLESHLKELE-<br>KKLAKNEYLPDIAADNKKLEKSNKALEESVRELEAAQAQAEAEVRALTVAREQEQ-<br>QHVRELEQNLRVALEQLAAEKASLQQRVATLEDELVQAQTDAEELELALEEKNDS-<br>LQALQEAHDELQKDEQALISALKQVKAESAAEHSTTEQLQQQEQADLRVAAEKIA-<br>VLSRQVEELGEALETQLAHKAELQAQLLEAXXXXXXXXXXNLRAALEQLAAEKASL-<br>QQRVATLEDELVQAQTDAEELELALEEKNDSLQALQEAHDELQKDEQALISALKQ-<br>VKAESAAEHSTTEQLQQQEQADLRVAAEKIAVLSRQVEELGEALETQLAHKAELQ-<br>AQLLEAESALQEQGASESQQEETLSTLKEENKYFCEEIKRGAAVVLRLEARLKEL-<br>EKELAQQAEVVAESKQLQERVRELEAAQVQAEAELQTVAAAREQEQQHVRELE-<br>QGVKAAEERASTSQQEQAVAAQQTQQQEQQAQAQALQEQLMSAVESVAVYEEA-<br>LNALKQENAQLGAEIKRGAAVVLRLEARLKEQEAAKSEKAEVAATKALESRVQEL-<br>ESALAKAEGASSADVANLRAALEQLAAEKASLQQRVATLEDELVQAQTDAEELEL-<br>ALEEKNDSLQALQEAHDELQKDEQALISALKQVKAESAAEHSTTEQLQQQEQAD-<br>LRVAAEKIAVLSRQVEELGEALETQLAHKAELQAQLLEAESALQEXXXXXXXXXVAV-<br>YEEALNALKQENAQLGAEIKRGAAVVLRLEARLKEQEAAKSEKAEVAATKALESR-<br>VQELESALAKAEGASSADVANLRAALEQLAAEKASLQQRVATLEDELVQAQTDAE-<br>ELELALEEKNDSLQALQEAHDELQKDEQALISALKQMKAESAAEHSTTEQTEKLV-<br>ENEKHEARLVYDQLKEQYTQLTAVLTEKEKALEEAAEERQAMQAAVKSSEKKITT-<br>LENTIAKAQTDAEELETALNDKDGALQALQKSYDELLKDEQALIVALKKMKEESAA-<br>LHSATEKSEAQQAAEVRKVEERSRMELSNLHAALDKLASEKAQLADTLANKEEAL-<br>NAVRTELKQAQASIKEGEKKVATLEDDLVKVQTDAEELEAALEEKDDSLKALQES-<br>YDELLKDEQALIVALKKMKETTASLEVTSRQTEEQREEKLHVAEARAAELEARLR-<br>DEGTSVAAVQALLETKAQEAASWQEQLAKAEQTLAEKDALVLQLTEAARTAAAEG-<br>LAKLEEMEQLHSASLAREVARKEEAFVTLTKLKNERQYAKMDAEKAMQQAQTEL-<br>RTLQETHAQLQETLARVEQQREKEKANADEALRRLAEKYAEGKEEIDVLTAQTQE-<br>LLVKEKDAERLRAKAAQLEATLRADNERLEEQEGLIEELEAALRTSGEEQQRLVQ-<br>QVQELADRGTELAAEKERLELEAEAFKKTLDDTARSYNDRIVVEDRLRDRVEQYR-<br>QLAAARQLELRTAAAVWLQATESVRDGLREVESGAAGRTAPHLPLQPLQEAVAW-<br>YVRVALREPPVEATRGSGDLSDEDSSRRSSTAASASAPDEVKSLFEHNATALCQ-<br>EVGDTLRQLCEAGRAAARREETLARKLELEQQLRAALDTLAVENLDLKEAGRNYL-<br>TLFNETQAALQQITNDFQQQLLEEKEAASLEVITARRDMERAEEARRRAEERLQL-<br>TREELAQLQEANRELEGQLIEIGEERDELRTRVTVANEAMRAEASLEGSPSRNG-<br>GGGDIHVRASRYSAAVAQEQAQMLQVTSLQSDLLLARRQLRELEAQESALRRSA-<br>TLAEREAVVLRTENTRLQKELVQEREHTTALKRYSQLLDQTDIGAAAEELRQLRLK-<br>VRELEGVVFRFEEKERALARSRSQQEVQAGRRQEAVLARLRSMSSRMTDVRKQ-<br>LEAGEGGGERASTGSARSRRGSLTLATDPQLVAEVKHLQHDLETYEKLAVEHQL-<br>QLESLQAQLSQEREARTTADKTVQQLSRHNEQLRQTLGHERQSAQLALSPFTPA-<br>PFLQGLGAPSPLPRTPLPPTPTPDDDNEEEEEEEETTRTPLRSIHTQEGEEEDEQ-<br>VVVRSKSATLVAVSNKRTSSNRRSTTTATKQTKIEEEEKTKKAKASITPVKKTESR-<br>KRNRSKSASS</td></tr>
</table>

| | | | |
|---|---|---|---|
| Strigomo-<br>nas on-<br>copelti | Trypano-<br>somatida | NCBI\| com-<br>pound of:<br>AUXK01001<br>933,<br>AUXK01001<br>270 | MFRSTYVSTSNTSSSFPHQTDRNTTPQGVAFSSSLEGPISVREAPRWGGHGNS-<br>GLPARPLDVTSSPASAGHRTGGGDLSDLQGQQPLFNPDDDSYYRYYQQQ-<br>PLPAPLPPPSAQSASFAAAAAPARPYRGGDSIVHAPRDTLLWATMASGREPPAF-<br>SPPSAERPAQRQECLDEFLLTAAEGEALDHKTPAEVAAYVQRLRANYHYFGE-<br>QLREACERAQHYKAEVARLREALETQRLQRQKFDIDCEAQDGVQHALEEQV-<br>RRLRGQLEEQEGLLLRLRQQLAARGAEQSTSTTSEIMRYEVERVKAAEEAR-<br>WAQQAMRDLAAELQRSKAQVKALEVQQQQEQQRPAELARQLAALEKENAQ-<br>LHQSLAAEREEHETQKQQLEEALAALQADTDTMALALTKRAPISLEEYEAQQQ-<br>RLAALQRQIEQLATETAAQKAAVAPLEATALEVTELRQHVAAAGHEKAALERA-<br>GQRKDEEITRLLELLQCYKTEADAAQEANRVRTERYEAALVEQVEVAEAARQA-<br>AETALALLRQQLEEAQASAQHQEHQGEEGNHHDAFFAASGMLGGAGNEAHD-<br>LRVAKGKIATLLTLVQELEASLAAQQAERQALQEQLATTAASRSHDAASLQEEL-<br>QQLRHEKAYLGEEVQRAATVVLRLEARLSEQATTQQQATAATAEEEKEKEELR-<br>AVMEKLGLLQQQLQALGKTLETQQAEKATLVEQLLAAEGVVQEQRGTLTQNTA-<br>ELEALRQENALFGDEIKRGAAVVLRLEAALRERERAEAQRDEVMQTNTELEK-<br>QNMTLKKSNAALEKRLLELEEAHAQAEADVQALATAHEKEQQRVRELEQGVQ-<br>AAEQRVLASQEEQVAEMHKEQERHASEDAHAAQLHATIAQYEGELEELKNER-<br>AYLAEEVKHGAAVVLRMEGLLKEHEKREAHRPDVITANTELEKANKALKKQLH-<br>HLEHVHSQSESHLQALQESSDELAKDKQALVAVLKQTREESAALRGAGEAAE-<br>ERARAELANLRAALDVLAREKAELTEALSAQEARAAQQEQQGARAAEERVSA-<br>SQQELAAEAQREGAHAAQLQAAVAQYEAEVEALRQENAVFGDEIKRGAAVVL-<br>RLEAALRERERAEAQLPEALAAKEALEQRVQELEAARAQAEADVQSLQESHD-<br>ALLADEQALVAALKQMKEESASLNAATLQAEERATAEAGTLRAALEQLAVEKT-<br>QLADAVTALTRDKEALEQSVRAGEAEAAALEDRAVQAQTNAEVLEVALKEKNS-<br>SLRALQESSDELAKDKQALVAVLKQXXXXXXXXXXXXXXXXXXXXXXXXXXXX-<br>XXXXXXXXXXXXXXXXXXXXXXXXXXXXXXXXXXXXXXXXXXXXXADVQSLQE-<br>SHDALLADEQALVAALKQMKEESASLNAATLQAEERATAEAGTLRAALEQLAV-<br>EKTQLADAVTALTRDKEALEQSVRAGEAKAAALEDRAVQAQTDAEVLEIALEE-<br>KNSSLRALQESSDELAKDKQALVAVLKQTREESAALRGAGEAAEERARAELA-<br>NLRAALDVLAREKAELTEALSAQEEALHVARSEAEEAHKTLKARETTAATLEDA-<br>IAKERSDVAALTEELETKAASLEALQSSCDELAKDKQALIVVLRQTREACANLE-<br>ENSHEAEAQQEEKLKVVEARAAALEARLRDEGVSVAAMHAELEAKAQEAAG-<br>WQERLAKAERELAEKDALVQRLSESAAAAAAESLAKLEELERLHTVSLANEVA-<br>RKEEAFVTLTKLKNERQYAKMDAEKALREAQERLRASEEAHAQLQAALAKVE-<br>KQRDADRVNADAALRRLAEKYTEGKAEIDVLTAQRAELLAKEKDAETLRQKAA-<br>QLEATLRADKERLEEQEGLIAELEEALRTSGEQQEQLVEQVQRLADRATALTA-<br>ERERLQMEADALRKTLEDTARSYNDRIVVEERVRDRAKRQTQLAAARQLELR-<br>TAAAVWLQATESIRDSLLAVEGAPDGRKDRPAPALPLEPLREAVAWYARVALRE-<br>PAIESPRDSYELSDDDSSRRSSTAGEGAATPSQPDPVRERFEHNAAGLCAEV-<br>GDRLRLLWEAGRAAARREESLQRQLALEQQLRAALDVLAAENLDLKEAGRNY-<br>LSLFNETQAAVQQITNDFQQQLLEEKEASSLEVITARRDMERAEEARRRAEQR-<br>LELTLEELEQLQSANRELEGQLVAVGEERDVLRTRVTVEREALLAEVSAETSP-<br>SRDVNVRPSRYSAAVAQEQAQMLQVTSLQSDLLLARRQLREMEAQETALRR-<br>SLAQAERETVVLRAEATRLQEELVQEREQTVALKRYSQLLDQSDVGAAVEELR-<br>QLRLKVRELEGVVFRFEEKERELARVQSQQEVQAGRRQEAVLARLRSMSSR-<br>MTDVRRQLEVGGERDAVGSSGGLSLATDPQLVAEVQHLQQDLENYEKLAVEH-<br>HLRVESLQAQLTHEREARTTADRAVQQLTRSNEQLRLTLGRERQSLQLGLSPF-<br>TPAPFAQGMGAPSPLPRTPLPPTPTPDDEEEEEEEETETTRTPVRSLHTQEGD-<br>EVIVVSRSRSRSRSSRTKKPTKKETKTEERRTKPNKTGVSVTPKLAASHKRT-<br>RSGSAASQK |
| Herpeto-<br>monas<br>muscarum | Trypano-<br>somatida | NCBI\| com-<br>pound of:<br>AUXJ01004<br>927 and<br>AUXJ01009<br>581 | MMPLNDPFRGPRASSSSAATYRQPTLAPELARPISVRELAYG-<br>GRSRQQQHQQQRQQSTFTAPPPPFLPDAPPLQTAPTAAPVYAATSSLGEY-<br>DMHTPGGRVSGARPSTSSLTAAAADRDGSISPLGDRQLRDFASSAQKSIDMM-<br>SPRELYDHTSRLQDEARQTRGYVAAAYAARDQYREEVGRLR-<br>QELHSRYLQSQALERDRGRYNDVIHKLQGDIAALRRDLDDAE-<br>ARENALQGRLARANTAMAGPSSSSSGSGGTRPPAAPEADPA-<br>QLAYYATQLAAKDGQLDELEAENTALRAALRDTEQQQQQQQQ-<br>RSAPALTGEDQRVAAQLQRALEDLQQENAVLADALATERESRA-<br>ALLAETAAEKTRMDADATRAAASNAALQKELDDLLEAFSSRAP-<br>ITNEHYEHLRRELAEQQRTIAALESEAANRAAPAVAAEAVERAD-<br>GAAELEASALRESVATLRAELADALKQLAKTQELVAEFEKETWL-<br>AETANTVRQGLIDDLRANLQAATAAREAAEAALAAERTAHEAL-<br>AGRTDADDVHSAVAELAAEVAALRATGQSLEGEKAALEVSLA-<br>STQAALDAMAERNSALEESEQRHGRAIEELQAQLREQQRAAA-<br>AVTGEPATQSAAVDALSGETALEAGVAAKAADERAQRAEAELA-<br>ATQKALEDMAGLLDEERQRAGALADTAAALDRSVAELSARLE-<br>GPTPPPPAAQLLDSGDQQQNQQQQMAALQSSHAEALRALSE-<br>AESATAAQHAAAMASLTEELQAAQAAAEKAVLLEAAVDTMAGQ-<br>LEAAEARARELVAGLAAEQSVSLALKEQLASKEAHLAAASAAVE-<br>GPAGDTQRTIIDLRTQLDALSTANTALSERLEAAEATLQAAAKD-<br>RDDQAEQLRRTSSELAEAREEADRHRRAAETTAQQLAAAEAQ-<br>VAALQLAIDSLAEQVDASQQRAVELEETLREVQSVGDGARAEA-<br>ARLTTEAEALQAAAAQGQGAHTSDAVELNSQLAAARAHNEEL-<br>TRRLQEMDAATSAAVAERGQQPHQQQQEEEASALASAQQLC-<br>EKAREAEAEERARREKADADVTALQAALEQLADELEKAKADA-<br>SERQGVYDTTTSMLQGQLEEVRTNNAALSARLQSSEAGVAST-<br>ATERDQQSTAVEDLRKRLESVEAELASRXXXXXXXXXXXXXDLR-<br>RRLEAAEADAVTKEAAVSAQTAAVAKAEADASALQAALDDVAV-<br>RLAETESVVVDQQQALDSTASTLQRQLEELRGQNAALLERLG-<br>SSAADCDGHPTAVEDLRSRLAAAEADAAARQTALDAVAQELD-<br>AAKAEVEDLRASLADARAHATTLQHEAARRESAVSHLTGAVVSR-<br>NEILHSTAGQSAISEALRRQDAAVEEKLRVTEAALAALADAHD-<br>ARSVELGQLQAKARSSKNAIAAAEAAAAQSAAAYRTAQEALN-<br>RMADEVARQQEARAAAETALAAQQGSNASLVATTAESASSAAA-<br>LQSALDTVSTALDAERAHAKELEGVVDELAARLEEGAARLSLA-<br>ATDAETSGALRAEVEALAAKAATLEKALADAEKSLADAEKVTR-<br>QLTAERDQLAAARAQAREAAEATRAEGAARASAVENGQLLQA-<br>AVNTLAGDLDAERARTAELAASESALRARAAALEADLAAAAKT-<br>SAALESTLAGEARSTEDRLRGFTEELTAQRDTAAQLQRDKAAL-<br>QERLDALQRSASTATAAVEELANRVSLSAEEAAALRSVEAEV-<br>AVERQRSDEQTRRCEALEKELATATAASQAAAAQVEELKRRIG-<br>DLETGAGAAAAAKATAATALATAQQRVVDLEEALTQAAVELSDA-<br>TAQLKELAERRAAEKKEHASALAAIDDLAKQLQEAQEAAGEL-<br>GTVRRRWEEEQRRCASLTERATQLDTTLADVMDRLEDTSALL-<br>ARSREEMLAATDESKALEAALAAMKEQLTTSERQRVDAEERL-<br>DAALLQNTQTAAMYNDRNDEISRTQARATDLKSRLMDRDSAL-<br>ELAAAHLCDCAKAVQVALLGVLRQPTTPAGRSSGSTARGAASQ-<br>IAKDLRPFAEALDWIATVVREDVADDDRVVALPDDCTEDETAA-<br>ALRRRSSARASSSSLSELAAADKEGNAQRMSSLTSALEELLNE-<br>RLQGAVRATGLEETLAATEERLRAMDEALVRLSEDKLSEVERA-<br>DSLYAALTNVQRAVSRTQAQYEQQMRERTESATADMIVAQRA-<br>AERAEEAKRRAEERLSLVEQELEEQRAELRGLQEANRDLGL-<br>ENNRLSRMLPNSHRGPHGHDDGADGRSEPGSATARRGFGD-<br>VDQAEEAQMLHVATMNVELMQTRRRVRELEANDATLRRAYTV-<br>LETQLEELRDEVGENERLRGELDRLREEHADLALRYDALVETT-<br>DATEGGVAQELKSLRQQRSLLEAELAEWKARYRDLNLARNLP-<br>KVEASQREAALSEQLRGLTSELDAAQAQYARPSTANGSGAG-<br>RGGDAAQARLAHLEASNREKEARLQQQAAERLRDQSEIEALE-<br>DEVARKTAALEEAERLAAQYARQVDSLTTNAKALRRSTQVGRA-<br>SSALRLSPPGTAQPGPSPQPRAPSEEGTRRGHSNSPSSTST-<br>RSPRASTVGSRTPRPSLLGAGRGGAKRRTTSPENPPATKRTKA |

| | | | |
|---|---|---|---|
| Phytomonas sp. Hart1 | Trypanosomatida | NCBI\| compound of: CAVR020001000 and CAVR020001443 | MYSRHSRFALHPASTKEVRMTSVFSPDLPRPISVRDLAYEDASSGVRLVRSRTQPISHSRAIPHITNPPCLGGGEGGRGMRTRSEIRVGAPTAGAVYTATGRLAEAPSSWRRNWARTTPQPDSTSLAACSLDEMNEGELYTFASRMQDEARHARGHIAQAYELRDQYIQEVARLKQELHSKYLQTAELERNRGKYEDTIQSLREDIQKLRRELDASEGREQVMRLRLAAGTSVPFPTLASGSDIPDGSGQYDFIVKYYQTELTTKNQQIQHLKEHIETLEKQLQNEQRIQTPESVFIPSKSPSRSAANANSEMYTIGTGDIFSRKVGLGESAVHEPGICWDPNELLRQISQLEATNSKLRQDLEESVQRQSTIQLEAERSRSELQASSQTRINNLEEEAEQLRTLNISLQQEVDMLTITLANQAPLTRGAYETFQDENETLKIRFSDLEAKYQSLERENLVLAAAALETDMLRESLTKVRLEHRDTTEQLKLKNEKLLELVSTFEEETKLSMTANNLRKTQVESFHKQIEELTSELNALQSLNEKKEIEIGAAEAAKVQKMNQLEAKLVELEEKCDNAEKKLNNTYIQIEDERKRRKKQEREMLNQTKQHKRLTQTVVXXXXXXXXXXXXXXXXXXXXXXXXXXXXXXXXXXXXXXXXXXXXXXXXXXXXXXXXXXXXXXXXXXXXXXXXXXXXXXXXXXXXLKTYRSNAENIIASLKSQISLLSAELSDHGFLLPVSQESIGPSDTILHDSIQFDDSSLVHLTTIKASSVQRNYSLQYALALLRDVISAFCCLTSDLNRFVKLNVEHLDNSSTLSTDTFVLPSGAFEACLEPVAMASRWAAQMQDILKKNSTDELGSCESFDRSLRIPKTNLVDISAEQEASWLISAIPEFSVLVEARKASDIVVCLRDLLSRVDSLVYSLQEEKEHSRSLSAAMDQIAADILDEKERSDSLFGTIADINQQLEKTKADFDEQIRKQEVASSVELIAARRAEGRALEAKRRAEEQLVLVETELEEERMALRKLRDNYGELKIEAERLTRLTGSISDANQASQQDWRATEAENTDTNHQIGLRDDQLEVLEQAQILQISTLQAELLQMRKMVREAESREAVLNQKNRVQEQTLAEQHEEVHKTAALLMDFDQLQSEYTDLLNRYKNLVESSSTTEISTIEELKQLRRQVRLLETELAEEKAHTRAIELAKNTPDLEAMLRQEALGETLTGLSTHLAAVQAQYARPSVTNSASPNDHSFSGEVPKSNDDVSGSGVAVGSNEDDTIFRTPNPSIDLLHSRITQLESSIKEKNKKVEKYAADILRQQTVIETLEDNLTNKLTTLDRTEQLNEQYLSTIDALTRSSKALRASAPVRLVVENTKTAAYASDKHSNRSRRSALRTTSGEDASRPGLSPLPRTPQQFVVETSSAQSDATESDDVSVPSKSIMKVVSPTISPKKKSTGARSRPRLELDKDNLNIGGVEKGHRRSNRRSESQSNSESPRVSTTTSRTSNLALHTSSARILTKRKRGRTIS |
| Phytomonas sp. EM1 | Trypanosomatida | NCBI\| compound of: CAVQ010000701 and CAVQ010000700 | MFSRHDQFVLHSGSAKKDVEMTTPFSPDLPRPISVRDLAYEDLSSGVRPVRRGDPVPLRSRTQPATGRPSRAVPPTSNPAERLWGGRNLPRISAPTAGEVYSATSRLAETPWTPRRSQGEPPLADSASLTARGLDDMDEGELYNLASRLQDEARHARGHIARAYELRDQYIEEVARLKQELHSKYLQTTELERNRRDYLDTIQSLREDIQKLRRELDVSEEREQAMRLRLAAGASVPFPTIAAGSDVSESSAQHAFVVKYYQTEITTKNKQIQQMKEHIEMLEKQLQKEQHIQTPESVFIPSKSPSRSAANAHSETYTIGTGNISSRELALGDRPVQELSTGPDLDQLLQQIAQLEGANAKLVQELEESVQRQSTIQLESERTQSELKMASQTRIVELETEVMQLRELNNSLQREVDTLAPALANQAPLTRGAYETFQEEHESLKMRFSELEIEYHSLECEARVLAATALETDMLRESLTKLRLEHRDTTEQLKLKNEKLVELIATFEEETNLSRTANDLRKAQVESLHKQIEDLTSELRTLRNHSTHEQVEMRVAEEGKVQKTDELETKLAMLEDQRINAEKMLQKAHMQIQDERKRCEDLEKEVLDRTTQLENLXXXXXXXXXXXXXXXXXXXXXXXXXXXXXXXXXXXXXXXXXXXXXXXXXXXXXXXXXXXXXXXXXXXXXXXXXXXXXXXXXXXFGEYRSNAESLIASLKSEVSLRLSEIEDLRLSILASQKSLEQVTSMYNDCIELDDSRQEALKAMRESFEDRHNSLRYAVAYLRDVISAFSRLTLDVNCFVKFSVARLEDPSSLSPDTSELSSVVFDACLQPVAEASRWAAQTHDTLHKVVADELGNFDFPDESLNTAKSNVSGASIDKEASWLALVIPEFSVKAEAQKALDVIACVQDVVSRADRLAHSLQEEKERSRSLTAAMDQIAADLLDEKERSDSLFSTISDINQQLEKTKTYFDEQMRQQAAAASVELIAARRAEGRAHEAKRRAEEQLVLVEAELEEERMALRKLRDTYGELKVEAERLTRMTSSISDVNQTSRQGWRAAEADDTDASRHIGLRDARLEVLEQAQMLQISTLQAELLLTRKMVREAESREAILSQRNRILEQTLTEQHEDVQTTTTLRVELAQLQSEYTDLLERYKNLVESSSTAEISAIEELKQLRRQVRLLETELTEEKAHTRALELAKNTPGLEAMLRQQALGETLTGLSTHLAAVQAQYARPSVSNSASPYRFSLPVGVLKANDGISGSGVTSGNSEEGANLRTPNPSIDLLQSRIAHLETVLKEKDKAMEKYASDNLRQQAEIEALEDNLASKLVALNRTEQLNEQYLGTIDALARSSKALRASAAVRLSVKTNKTAVCTASDQHLTRSPRASLTAASGGVSTGSGPSPPPRTPQPLLVETGSSYSEGSESDVTSTPFEPIEKVVSRSISPKKKSPRARLRARREVDEDDPISGDIVRVRRKSNIRSESQGSSVSPRVSTTTSRASNVARNSSLAQTLQRRKRGRKIS |
| Phytomonas serpens | Trypanosomatida | NCBI\| compound of: AIHY01001827, AIHY01014644, AIHY01019444, AIHY01020153, AIHY01009940, AIHY01020207, AIHY01020131, AIHY01002605 | LVRRGDPPPPPRSRTQPATGRPPPPPRLSAPTAGEVYSATSRLAETPWPPRRGRGGGEGPPQVDFSPLEPRGLDDLDENELYNVASRLQDEARHARAHIARAYEMRDQYMEEVARLKLELHNKYLQTAELERGRGDYEDTIQSLREDIQKLRRELDNAEGREQAMRLRLAAGVSVPFPTMDAGSDVAESSAQHAFVVKYYKTEITTKNQQILQMKEHIEMLEKQLQKEQHAQTPESVFIPSKSPSRSAAHTHSETYITGTGNISSHELALGERPVHESSSGQDSDQLLQQIAQLEMANAKLVQELEESVQRQTTIQLEAERNQMELKTTSEDRIVQLEKEIEKLRELNTSLQREVDTLAPVLANQAPLTRGAYETFQEEYESLKIRFSELEAEYHSLEREGRVLAAAALETDMLRESLTRLRLEHRDTTEQLKLKNEKMVELIATFEEETKLSRTANDLRKVQVETLHKQIEELNNELKALRNLNTQEQVDREDKVHRTDEFETKLAVLEGQRDNAEKMLEKAHIQIQDERKQREELEKQVLDQKTQLEHLAQVVTRVESRRVEIEESMQSTVAANFIPHQRFSELQSALETCEREKADVLQSYAKAQAAVDTLTAKLGETQRVLSATSDQMDQQNSLIDSLRDQLEDALHSDTISAMQAALDKLASDLEDERERCAELSEKLEDACRRAKAAELDKAQQAEQIEHLAQVVTRVESSRVEIEXXXXXXXXXXVESSRVEIEESMQSTVAANIEQQQRFSELQSTLETCEREKADVLQSYAKAQAAVDTLTAKLGETQRVLSATSDQMDQQNSLIDSLRDQLEDALHSDTTSAMQAALDKLASDLEDERERCAELSEKLEDACRRAKAAELDKAQQAEQIEHLAQVVTRVESSRVEIEESMQSTVAANFVPHQRFSELQSALETCEREKADVLQSYAKAQAAVDTLTAKLEETQRALSIASDQMDQQSSLIDSLREQSNEVSKSDTTSAMQAALDKLASDLEDERERCAELSEKLEDACRRAKAAELDKAQXXXXXXHLAQVVTRVESSRVEIEESMQSTVAANFVPHQRFSELQSALETCEREKADVLQSYAKAQAAVDTLTAKLGETQRVLSATSDQMDQQSSLIDSLRDQLEDSLQSDTTSAMQAALDKLASDLEDERERCAELSEKLEDACRRAKAAELDKAQQAEQIEHLAQVVTRVESSRVEIEESMQSTVAANFIPHQRFSELQSALETCEREKADVLQSYAKAQAAVDTLXXXXXXXTVAANFIPHQRFSELQSALETCEREKADVLQSYAKAQAAVDTLTAKLGETQRVLSATSDQMDQQNSLIDSLRDQLEDALHSDTTSAMQAALDKLASDLEDERERCAELSEKLXXXXXXXQSTVAANFVPHQRFSELQSALETCEREKADVLQSYAKAQAAVDTLTAKLEETQRALSIASDQMDQQSSLIEALREQSNEVSKSDTTSAMQAALDKLASHLEDERERCAELSEKLEDACRRAKAAELXXXXXXXXXANIEQQQRFSELQSTLETCEREKADVLQSYAKAQAAVDTLTAKLEETQRALSIASDQMDQQSSLIDSLREQSNEVSKSDTTSAMQAALDKLASHLXXXXXXXAQAAVDTLTAKLGETQRVLSATSDQMDQQNSLIDSLRDQLEDALHSDTTSAMQAALDKLASDLEDERERCAEXXXXXXXXXXXXELDKAQQAEQIEHLAQVVTRVESSRVEIEESMQSTVAANIEQQQRFSELQSTLETCEREKADVLQSYAKAQAAVDTLTAKLEETQRVLSATSDQMDQQSSLIDSLRDQLEDSLQSDTISAMQAALDKLASDLEDERERCAELSEKLEDACRRAKAAELDKAQQAKEFNEYRKNTESRITSLLSEVSVRSSEIEHLRLSLLTTQNSLEKVTVMLNDHMELDDSRQSHMKVMRESLMNKDNSLRYAVTHLCDVINAFSCFTLDVNRFVKFSVARLEDSSSVSSGTPVLSPVVFDACLEPVAEALRWATKTHDTLNQVAAVELESFVSLDQALETAKGNISEASTDQEASWLALVIPQFSVKEESEKALDVVACVRDVVSRADRLANSLQEERERSSSLMAAMDQIATDLLDEKDRSDSLFSTIADINQQLEKTKAYFDEQMRQQAAASSVELIAARRAEGRAQEAKRRAEEQLALVEAELEEERMALRKLRDSYSELKVEAERLTRLTSSISDANQASRQGWRATDADDTDASRHIGLRDARLEVLEQAQMLQISTLQAELLQTRKMVREAESREAVLSQRNRILEQTLTEQHEDVQTATTLRIDLAQLQSEYTDLLERYKNLVESSSTAEISTIEELKQLRRQVRLLETELTEEKAHNRALELAKNTPGLEAMLRQQALGETLTGLSTHLAAVQAQYARPSVSNSTSPYNLSLPVGVSKGNDGTLGSGVTSGSNEEGAILRTPNPSTDLLQSRIAHLETVLKEKEKAMEKYASDNLRQQAEIEALEDNLASKLVALNRTEQLNEQYLGTIDALARSSKALRTSAAVRLSVKANKTAVHTASDQHLAKSSRGSLAAASGGVATGSGPSPPPRTPQSFLMETGSAHSEGSESNVSTPSEPIEKMGSHSISPPKKSPSTRIRARRKVDEDDANTEEVTRVRRRSNTRSESQRSSVSPRVSTTPSRASNVTNHSSLAQTLPRRKRGRKNS |

| | | | |
|---|---|---|---|
| Crithidia fasciculata | Trypano-<br>somatida | NCBI\|AOD-<br>S01000118 | MWNSSFYAEPAHRAGGSAPSSTSLPQSSRKPPPVGVLSPHLARPISVRDLAQS-<br>RVPTVAPPRSSTHSFSFGSGAHPTPSPRTSGGFTGLAVPKAPVVFAATS-<br>GVSLGEHSGRPTDVSLTSLQNTVEASDLTSLAARNIDHLNFNDLYTYASHLQ-<br>DAAKNYSNNLSAAYTKRDMYRSQVAELKQELQERYLQVDLLRRDHERADDALL-<br>RLREDNTQLRQLLAESEGKLRNVSARLSLRNGADPSAAMRAYQAQLAQKDAQL-<br>RELVERLAQQTAEAAQRLGSEASAAGQRRSHTLSAAADEETRANEQQQHAEE-<br>EEARLQAKVRAMEERVREEAREATASAIEAATTVLQAEIDALHQQHATLQSQLA-<br>AETASHTQERTELLETIAALRREKGDMQESEDALIAALTLRAPVSKADFESLQAS-<br>YAELQASLANSEASVASLREQLSKAVVQEGYTEVLQQSLTTTQAERQELVTKTE-<br>QLQALVDNLTRELQLADEANQLRQQQMEETAVANSDRWQQSQHTIQELQERE-<br>ASQVAELQRVRAEMDSLRDELRDLQASRSAMTTTTTATTHNGDITHERTELMAE-<br>LETLRNTSGDLVRQRDALMSEVRHLEERVQAAKEELSHHSSTPPPALSPASQEG-<br>EAIADTAALQVAQQCIMALQHDLEVANSVAARAEADMQAQLAAQEKQVADLAEA-<br>LRVAQERQREAEEADLNHARAAAAAEHHLKDVVKALKEELELTQAQRTELLQR-<br>DKTHQEHHAAQLHVTEQLRERIVDLEKRASEVRPHEPTPPSTQPTHISARTSTA-<br>TEAETEKVRLTTMSAAIDRMAEQLAASEDRVRDLEAERQRQADELEHARRAHA-<br>ALEDRVVAHERGSRAVASDGGASAAPAPEHAMRVLAAERDAACAEMAELRSL-<br>LQSHEAHYAADLAAMEQRISSVGEELRVSKAALEQMATDLTTATHLVGSLTAEKT-<br>QLEAEKVDLSTALDAAQSSAVNAQKENDALVATDTERQNKLDAAQAAVSAAMQ-<br>ARNAAEQRATTAEEEVRAAKAALEQMAGDAADTAQQCAALSAANTELEAKVLS-<br>LASEFAAAQDMLMTLNSERDSNAEQESTASQKVQAAEARASAAAAELSAVTHR-<br>VSASEDEVRAAKAALEKMAGDLSDAEGAVQILTAAKSTLEAESASLKAALFAAEQ-<br>TAATLAAERSSHAGTESEARAMLEAAEAEASVVAAELSTAQQRLAASEEEVRAA-<br>KAALEQMAGDAADTAQQCAVLSAANTELEAKVLSLASELAAAQETVVALTSERD-<br>SRAESESTASQKLKAAEARAAELASKASELEQRVGTTTKELRVAKAALEKVVAD-<br>FTESTRLAELLSAGKADVEVENAALKGDIAAAQAALFSLRAERDKDNGSDSTVN-<br>AKVEAAEAMASAAAAELSAMTHRVAASEDEVRVAKAALEKMGADLFDATQLVEY-<br>LTASQSQLEAEKARLYSALSLAEESVATLRKEHNSCSMKDSEVRARLEAAEAMA-<br>LNAAAEVRALQQRIATTEEELRVSRAAVDSMAGTMTASAHNSDSALARQTSLQ-<br>DDVVAAEARIASLTVECSSARDEAAQLFVQAQAAEARSVAVTADRATMEERMN-<br>AAVEELRVSKAALEQMAADFTTATRLVGSLTAEKTLLEAEKIKLSTALDAAQSSAV-<br>DARKENDALVATDTERQDKLDAAQAAVSAAMQARNAAEQRATTAEEEVRAAKA-<br>ALEQMAGDAADTAQHCAVLSTANTELEAKVLSLVSELAAAQETVMTFTSERDS-<br>RAERESTAVQKVDAAEARASAAAAELSAVTHRVSASEDEVRAAKAALEKMAGD-<br>LSDAEGAVQILTAAKSTLEAESASLKAALSAAEQTAATLAAERSSHAGTESETRA-<br>MLEAAEAEASVVAAELSTAQQRLAASEEEVRAAKAALEQMAGDAADTAQQCAV-<br>LSAANTELEAKVLSLVSELAAAQETVVALTSERDNHAESESTASQKLQAAETRAA-<br>ELASKASELEQRVGSTAEELRVAKAALEKVVADFTESTQRANDLQLANTALEAL-<br>RSQDVADLASAQERVAATEAKLNVSESAVEKVAAELSNSAKRLEFLLAVKADVE-<br>EQYKQRSAELQLVEQRVAALESRLGDAERRSADAATAAEQLLSTYTTKCDELEK-<br>ANARLREQLSEARSAVNTLAEECTSATAQSSTLAQKGETEAELRKTAEAAVVTL-<br>QEELSSAKAAIAQLTDDLTAQRDYNDTVQTARAAQEEAMAVLVSKAAEDSAELA-<br>EVRRRGEELQTMLDTTLDRLAQKELLLEEVNDNAAELHKERAALADDVVTLRSA-<br>LETAVVERQDLQERLAAANEGLERTAELYNERILEDDRMQEELNAVSAAFAAQT-<br>QEMRAGLAAMTEMSAGFCNVLETVMAPAKETLLAITDAAASSTDGGAGGGAA-<br>SARSSQLISAITIALAPWSAALDWAGKMERRLDALERGRAAVDGEGDAKADTDA-<br>ASTPPSSSPLSHASDPATARSALLDFVQYNEAALGHLHHIRRTMADRDDELRQL-<br>RSALDSMADATVEERERVDHLLSAIQDAKDHVARAEREFDEQLRLRSEAITAEL-<br>VVARRAEERATDARVRAETELAATAEELRESHLALRKLQEEHRKLTRETERLSR-<br>YSARLTDTSAARGRGGLSGSVTPTISAMLAETATNSYAVTASAVEALEQSNLLQV-<br>SSLQQDLLLSRRHARDLEGREALLRQNHSTLEQEVVSLRVEAAEAADLREEVAT-<br>LRADNAELEERCEQLERMLAAAGGGTMQELKSLRTQLKLKETELADLRARMRD-<br>FLLNRSTSEFEVVRREGDLRETLSGITTQLAATQAQYSGGRTARERGTALLSG-<br>GAGGTAGDEASPQGVLSSRIDHLENVVRERDAVIEKMQASQLESRVTVDGLQE-<br>NLSEKEAALDRANTLLAQYAETIETLHSAGVAGTTTRQLPATPRRTLSESPHSAR-<br>SRNNTASVPPPTPLGMSSMHDDTAGSISRAGVTAPMASPAPRMPNLDEVAGEE-<br>EGENSSAGDEAEAPSESPASVRRRTGSNGQRSSRRAGGRRSSEERTSADAP-<br>VPSRSATRSASATTTAAAGRKRGRPPTRK |

| | | | |
|---|---|---|---|
| Crithidia acantho-cephali | Trypano-somatida | NCBI\| com-pound of: AUX-I01001182, AUX-I01003068, AUX-I01001621 | MWDSTFNPAPSRRPSDGSSLSASMSATAPRPPSSVLSPDLARPISVRDLAQSRV-<br>PAVPPPRASANPNYLSGTPNAHRYSSGDGERANEAPSPRPSS-<br>GFAGFGVPRAPVVYAATSGVLPTWPSSRVSSGLDTLPPELSTHGEASDLT-<br>SLAYRHIDHLSSDDLYNYASHLQDAAKHYSSHLSEAYAKRDAYRSQ-<br>VAELNHRLQEEYLKTDLLRRNREKADDALQKLREDNTELRHLLAEA-<br>EGQTRNLSAKLSLQSGAEPSVVIRALQAQLAQKDAQLREMSERLLH-<br>ATAEHESAVEALQQRRSSSSGGGAHPRASNASSAAGLEGEDGTR-<br>DPPQHEQQTFDMEVEERVQRKVTETTEQAVAAATAHLRAAMEDL-<br>QQRHDALQTQLTTQATAHAAALGRAAAQRAELVDAMTLLKQEKAE-<br>LQANEDDLVAALTQRAPVSKADFESFQAAYAELQASLRTAESAAAT-<br>LQEQQNASAAQEGYVSALQHSLTTTQAERQALADANRQLQALVDN-<br>LQKELELAGTSLRLQQEQVASAHANEEQLAAQLQRTLAAEVMSTA-<br>QQELERRCRQLEEEVRRLQVDNDGLHQQQQQQQQQQQPPQQQ-<br>VVEEQVEEQEPLTQPASNNNNNAADATHQNSSSSAPLEEAAANMA-<br>REKAELEAELETLRISRDSLVVSRDSLVEAVRDLTEQQQQQQEEMA-<br>RVQAGTATTAAAAAAAAAAQMERDAEVDVATLRAAQECIAALQRDL-<br>AAANADLARREAAAQSQLQTLTNALAEQETLLAAAMERQNGAEE-<br>ADRQRAAAAAAAQQQHADEVIEALKEELELTQARDHELRESQSAYK-<br>AQYEAKVREAAQLRERVDELERPATVALENTYAAAVHAQAVQQTGA-<br>ATANAALDRLAEQLADAEERTASLEREKHHLADELARAQRSVAVQ-<br>KERSSAAVQGAAEQASDHSAPAVQQASANNVTDLLRQRDTALED-<br>ASRLREMHAAAEARAATVAQERDAAQQRATAAADELRTATAALDR-<br>MVAELSEVTQRVEALSSSNNDLQREAAQLAAARDAAAENLSIAKAA-<br>LERVVEESAALTECVNNLAATNAHLTDECGTNERRALAAEADARTA-<br>QAALEKMAEESANVAQPVATLSAITATLEATNAAMQTELLTARDTVA-<br>ALTKEHDTLCDANVELQQTLNAAVAAHASAASTAAAEQRALQLRLD-<br>AAEDQLRVAEAAVAKVAEEQADAVQRAQALANTNTSLEAASAQQA-<br>MELQRVLHAVTNALRPVRGVAHSVKAADADVDSIVQELTSSIAFLM-<br>AEGTRMKAELSVARGAVASLADTNNTNSDADAELKKQLTTAEARA-<br>ATVAQERDAAQQRATAAADELRTATAALDRMVAELSTASQEAATLTA-<br>TVRALEAERDAAQQCASTAAEELRTAKAALDAMAAELSEVTQRVE-<br>ALSSSXXXXXXXXXKKQLTTAEARAATVAQERDAAQQRATAAADELR-<br>TATAALDRMVAELSTASQEAATLTATVRALEAERDAAQQCASTAAEE-<br>LRTAKAALDAMAAELSEVTQRVEALSSSNDDLQRDAAQLAAARDAA-<br>AENLSIAKAALEKIAGELSEVTQQRATLTAAVAHLTEERDEARQQES-<br>AAAAREVFHVQAALDSAKAEELSATAQRNSEVVAAMAQLEAERDA-<br>AQLNAVAATAECRTAQAALEKMADRLSEISQGADARMASVEELER-<br>QHTQLIADRNRMQDQLTAAEAEVRTAKTALERMADEHADAVDRLR-<br>TLSDASNSLEGQMAQATAEAARLQQLLSDAEARTATLERRNAELAT-<br>ASAAQVTAHTAQVEELKKENTRLKTDLSVARGAVASLADTNNTNSD-<br>ADAELKKQLTTAEARAATVAQERDAAQQRATAAADELRTATAALI-<br>AWWRSCRRRHRRQRPSPRLCVRWRRRGTQRSSVRAPLQRSC-<br>ARAKAARGWRVGRGXXXXXXAATVAQERDAAQQRATAAADELRTA-<br>TAALDRMVAELSTASQEAATLTATVRALEAERDAAQQCASTAAEEL-<br>RTAKAALDAMAAELSEVTQRVEALSSSNDDLQRDAAQLAAARDAA-<br>QKRAETAEEAVHVAQAARQEMTKALAAATQHNDTLSNSNQTFEKE-<br>IAQQTAELREVRAALAAAEARAADAERRGAEALAAVEKSVAEGTSK-<br>IAEVEAERTRLKEQLSVARTAVSTLADNCTQESSQIADLKRQLETEL-<br>HEHKSAETTVAAVKRDLANAQAAVAQLTDTLAVFSANEEAMKASVA-<br>AQQEGIALLQRKHDDDHAELAQVRQRGEELQGLLNTTLDRLTQTE-<br>ALVKELNDNAAELHDERAALAAQLATVQAAFETVVHERQDLQERL-<br>AAAQLGLEQTTDLYNERILEENRVQDELRAVAKAFAAQTEQVKVGI-<br>AHVLHMSEGFRGAVTAVMEHATSTTQATAELAALSASGAANADAAT-<br>RLTASSHNTAAAVTAVNAAFAPWAVTMEWVAQMQQRLQKLEASQ-<br>VDENGERCSGGDADDLAAAAAATLTTRSTDVSDTRNALLDFVEQQ-<br>TMSLRHLQTVQHALNHRDEELQLLRSALDTMASTNLDEKQRADHL-<br>LQAIQDARRRVTMAEQDFDAQLRLRSEAVAADLVVARRAEERATDA-<br>RLRAETQLETVEAELRTTQAALQKLEEEKRQLTRETERLSRYSTRV-<br>VDVSARLRGAANGSVTPPLTALYAESTSTHYTMNAAAVEALEQAHL-<br>LQVSNLQQELMLCRRQVRELEGQETTLRQAYMVLEQQVVDLRTD-<br>AAEVEDLREERDTLRAENAEWEEKNAQLQRVMDATQRSTHQELS-<br>QLRQQVRVLESELEEHKTRLQVLMIGKSTPEFEALRRQEAFKENLS-<br>GLTTELAATQAQYSGGSNAAASPQNVYITRIEHLQTAIKERDVVIEK-<br>LQAAQLQSRVAKDSLEEKLSEKAAALSRVSELNAQYLETIEALRSAG-<br>VSAATRAATPRRNTEASLLTRSQHDSVMPPTPLSLHTSTSEKTGK-<br>AGLTARVRWQCRPLHHACRISTTWRPRKATKQVRSKKWRRRRPL-<br>LPPRAGVPGLWEKAAPVAVAVSVVRVAGPQAPRLRQASHLVPRR-<br>MLARQRQPANVVVSKFTPAQTNVTTGTDPFEGDF |

| | | | |
|---|---|---|---|
| Crithidia mellificae | Trypano-somatida | NCBI\| com-pound of: AHI-J01002057 and AHI-J01000179 | MWNSDFYAARPHRVGSPSSSTAPTPPPKEVLSADLARPISVRELAQSRVPAVPP-<br>PRASTSSFSSGNSSGATYLNGAHLPPSPRTSGDFAGFGVPRAPVVYAATSGVLPN-<br>WAPTARTTVTVPPAELSTYGEASDLASLAERHIDRFSHDDLYNYASHLQD-<br>TAKRYSNNLSEAYTARNMYREQVAQLKQELQQKTLQVDLQRRDRERCDAAV-<br>QKLREDNTQLRLQLAEAEGQTRNIAARLSLQNGADPSVVIRAYQAQLAQRDAQ-<br>LRELTERLACESSVAEARQGSGDGDGEADTRPSRALGAAESNDDRLQRQQQ-<br>LLEEEIKAATAQTVAAATAELQAELNDLQQRKAALERQLTAQETAHAAALARCA-<br>AERTELQVTIERARQEQAEMQENEDALIAALAQRAPVSKANFEALQTSFAETQ-<br>AALTKAEAAVATLQEQQRTVAAQEGYVEVLQQALATGQAERQELTDQNRQLQ-<br>SLVDNLQREVELADTANQLRQQQLETALQGGEQLAEQVQHAEAMHAAHMSEE-<br>IGALQERCAASEAEMQRLQSENEELHRQLQEPPVSQTATHASHLVASDMANL-<br>EREKAELAAEVETLRGARDSLVVYRDALVNEVRELEERRQCCSAELEAAQLR-<br>NTAAQLEAEAPVSAAPPSPLDDEEDTEEHGVMLQAARQCIAALQQDLETERA-<br>DHAHAEADAHAQLLALERTVAEQRAALRTAEEQRTHAEAAAQNTVVERLTNL-<br>NTMHETLMTRAAAVTAERDALRQRAAAAEEEVRTVKAALEELAGDLSSTTQR-<br>ADTAAAANEALQAENAAMKTELSVARSAAASIASERDSRREDDGAAHERLEA-<br>AEAHARAAATECEGMQQRLRTAEENDRTAVAALEAMAEKLSDAEARVEALTS-<br>ENGALTQAKTQLTAERDDAQRRVAAAGENERTLKAAVEELAAKLAAADQHVED-<br>LTAQTGSLEAERAQLCSERDTLRQRVSDAAEELQSVKAALEDLAAECSDTKQ-<br>RADALAAQVAGLEGERLKLLAELEHAHVVLAEVEVRLADAEHRDSERTAAME-<br>HLSAAQLAQIASLEEQTSQLKKELSTARAAISTMAEHRADTETQISDLQSQLNH-<br>ASEQRKTEEKTASALRSELAVARAAVAQLADDLATHAADDTALRAAAQAQDEA-<br>VALLRTKHEEDNAELMELRRRAEELRGVLSTTLDRLAQKEELEKQLNDNAGE-<br>LAANNAALTEELATLRQXXXXXXXXXXXXXXXXXXXXXXXXXXRAAATECEGM-<br>QQRLRTAEENDRTAVAALEAMAEKLSDAEARVEALTSENGALTQAKTQLTAER-<br>DDAQRRGAAAGENERTLKAAVEELAAKLAAADQHVEDLTAQTGSLEAERAQL-<br>CSERDAAQRVAEVSEGNTSAAKAALERMVEDASALEQRASCLTAANAQLTAE-<br>RDAMQQRVAAAEEEVRTVKAALEELAGDLSSTTQRADTAAAANEALQAENAA-<br>MKTELSVARSAAASIASERDSRREDDGAAHERLEAAEARARAAATECEGMQQ-<br>RLRTAEENDRTAVAALEAMAEKLSDAEARVEALTSENGALTQAKTQLTAERDD-<br>AQRRGAAAGENERTLKAAVEELAAKLAAADQHVEDLTAQTGSLEAERAQLCS-<br>ERDTLRQRVSDAAEELQSVKAALEDLAAECSDTKQRADALAAQVAGLEGERL-<br>KLLAELEHAHVVLAEVEVRLADAEHRDSERTAAMEHLSAAQLAQIASLEEQTS-<br>QLKKELSTARAAISTMAEHRADTETQISDLQSQLNHASEQRKTEEKTASALRSE-<br>LAVARAAVAQLADDLATHAADDTALRAAAQAQDEAVALLRTKHEEDNAELMEL-<br>RRRAEELRGVLSTTLDRLAQKEELEKQLNDNAGELAANNAALTEELATLRQTL-<br>EMALAEERELLERLDAANRGVEQTASMYNERILDMDRLQGELDAVTQAFAAQ-<br>TEQLKSGLTRVVGMSDGFRRAVEAAMAPAATTVRATAKLAADASSYVFEDCE-<br>GREAGAACSVSAAVAAVKAALTPWSETLDWVAATRQRIEELEGSRANAGAGH-<br>EDFSTASADGAAAATTDARSQAGEDSPASSYLSEVSQARSALLDFVEQQTTS-<br>LAHLRRLRHALHERDQAAVDKQAALEAVAGGAVDAQERADGLFRATEDARHR-<br>ILQAEREWEEQLQLKSQATAAELIVARRAEERANDARTRAEAQLAAAEKELKE-<br>TQAALRTTQEEKQKLALQTERLSLLSSQQSDSSALQRRRGSVGDSVTPPTAA-<br>VSTGSRTTRYTVSSSTEEAVEQTHLLQVAGLQQELVRCRRQIRELESEEATLR-<br>QAYMALEQEVSDLRSDAAEVVNLRAEMATLRADYADSEERYERLRLMVDAVG-<br>DGTAQENRRLRQELKLKDAELEEYKTRQENSLVSRGAPDFETQRLEEAFRQS-<br>MSGIATQLTAKQAEFSGGHHGGNSLSASPQDVLNSRIDHLQTLVRERDAVISK-<br>MKAADLQSRVKCEGLEEKLAEKTAALNGAEELLAQYMERLDAMRAAGLAAAV-<br>TTRATTTPQRNTGKSSRERAKRDSALPPTPLSLNVTDGSTSTSHKGATPAGLA-<br>MPSPAPRMPNLDDVAAGSGEEDEEDEVRKINGNGSEDDASATPPATSRRRTG-<br>ATGENSSCGNRRTREVHSSSSGTTPPRASPKVGKTVSTRKRAR |
| Leptopmo-nas pyrrho-coris | Trypano-somatida | Non-public short-read genome assembly | MWNSDYYTAPPHRVGGTTASSSVSAHSSHPPPPPPAGVLSPDLARPISVRDLAQ-<br>TRALAGPPPRASAATSAPSFGSGRDGSRSDYPVPLPRTSGGGFAGFGVP-<br>KAPVVYAATSGLLPSWSSTQHADSSGGASELSTHGEASDLT-<br>SLAERNIDHLSNDDLYNYASHLQDTAKRYSHHLAEAHAKRDMYRNLVAVQKRE-<br>LQEKYVQIDRQRHEQETATAALRRLREDNTQLREQVATAEGKVRNIAAKLSLQ-<br>NGADPSVAIRAYQAQLASKEAQVRDLMERVVHASAERQSAEEASQRRSSEG-<br>GANPRQSDTLGAAGTRDHDQQQQRLHQWPEEEHPCTIDERVRRELAEANA-<br>AAAASEMAKLQAELNALHQRNAALQEQLAAQDKTHAEALASMTAEHTELKETI-<br>AQLKQGHAEMQENEDALVAALTQRAPVSKTDFEAFQATYAEMQAALTKTEATV-<br>ATLQAQQNASAVQDGYIEVLQQSLATSQAERQELTEKNRQLQALSENLQREV-<br>QLADTANRLRQQQLETAVAGGEQLAQEVQRTQEARHTEVHAEIVALRARCAA-<br>ADAELQRLRAENEELSRQMQDARTQSETNHHETQEVSADNTRERSALEAEL-<br>ETLRNARDNLVVSRDSLLGELRELEERRQTAERDAPQQSTNAQHASETHTTV-<br>AAAAAASSVQHDRDTAEDTAMLQAAQQCIEALERDLETTNSAHARMEVDMQ-<br>VQLRALETKMAEQADALRSAQEQQKRDAEAYHNRTIAAAAEQKHLEEVIQALK-<br>EELEFTQTQNRELRQSESASRGLYTSQLRQAEQLRERVVELERRASDVRGHE-<br>LTPPSTQPASVGTCGSTTADAPDAKMNPSTMSVAIDRLAQQLAEAQDRVHTL-<br>ETERHRQAEALQRVQRVAATLEDRSVSEEHSSKAAGSSATSTRPSAQKGTC-<br>RYANGVKEGIVADQVATLIAQQDAAAHQTAQLRDTLEAAEARTSAVATERDAA-<br>QQRAAAAEEEVRVARAALEKMAAELSDSAHRVDELSADIGELATVKAQLAAV-<br>QRRASVAEDELSAAKAVLDRFTKEVSGVTRCANNVDAVMAAVEAQWDAVQQ-<br>REDASEAELRTAKAALERVVEEASASAQHASSLAAATSQLEADKTRLTSERD-<br>AMQQRAAAAEEEVRVARAALEKMAAELSDSAHRVDELSADIGELATVKAQLA-<br>AVQRRASVAEDELSAAKAVLDRFTKEVSGVTRCANNVDAVMAAVEAQWDAV-<br>QQREDASEAELRTAKAALERVVEEASASAQHASSLAAATSQLEADKTRLTSE-<br>RDAMQQRAAAAEEEVRVARAALEKMAAELSDSAHRVDELSAAKACWESEKR-<br>QRDAELARLESTAAEWETRTADVERRSAEAAAASEKRIATLAAEAAALEKETV-<br>RLKARLSAARGAVSSLAEDCTDVNTQTGDLTRHVEREVEQRKSAEAMLSALK-<br>RELVTAQAAVVQLTEDLEMRADNDAAMRATLKAEEEAFALLKSERDAAQQRA-<br>AAAEEEVRVARAALEKMAAELSDSAHRVDELSAAKACWESEKRQRDAELARL-<br>ESTAAEWETRTADVERRSAEAAAASEKRIATLAAEAAALEKETVRLKARLSAA-<br>RGAVSSLAEDCTDVNTQTGDLTRHVEREVEQRKSAEAMLSALKRELVTAQAA-<br>VVQLTEDLEMRADNDAAMRATLKAEEEAFALLKSERDAAQQRAAAAEEEVRV-<br>ARAALEKMAAELSDSAHRVDELSAAKACWESEKRQRDAELARLESTAAEWE-<br>TRTADVERRSAEAAAASEKRIATLAAEAAALEKETVRLKARLSAARGAVSSLA-<br>EDCTDVNTQTGDLTRHVEREVEQRKSAEAMLSALKRELVTAQAAVVQLTEDL-<br>EMRADNDAAMRATLKAEEEAFALLKSQHDDDRAELSEVRRRGEELQRLLDTT-<br>LDRLAQEEELVKGLNDNAADLHAERAALTEEVVTVRLALETVVLERQDIQERLD-<br>AAKLGLEQTAELYNERILEDDRLQDELDAVTQAFSAQTMQVKQGMRHMLQM-<br>SAGFHDALEAVMALAAATVKAAADLAASCTAASTKTMTHGSTERKPAQATQD-<br>KFRAVTAINAALTPWSAALEWVATMQRRVHALEGSSADSTESPDRISAALSDG-<br>ANDPSHMPLSVSLAAASTVADAAKARGALTDFVELQEALLTHLRRLGHALDER-<br>DYELRLLRSAVDEMANSNVAEKERADHLLEAIQDAKNHVSLAEKEFDDQLQLR-<br>SEAVAAELVVARRAEERATDARLRAESQLAAVGAELTDALAALHKTEEERRKM-<br>SREVERLSRFSARLADPGARVHSGGTVGSVTPPSSGLFAEGVSNHYTMTASA-<br>VEALEQAHLLQVSSLQQELLLSRRQIRELERQEVILRQANVTLEQQVSDLRAD-<br>AEEAVDLREEVARLRAENADLEEKIEQLERILAASGGGVAHEVKQLRQQLKLR-<br>EAELEEEKARMQGLLVSKTAPEFAAARRQAALRESLSGITTQLAATQAQYSSG-<br>RSPRGSLSAALSSNSAATSASPLDVLSKRIDHLQSAMKERDALIEKMKAAQLQ-<br>SHVTVDGLEERLSEKAAALERANALVTHYAEALDGLRAARVSAAARTLPSRGN-<br>THSSSAAQSPPPAARAPPTPLALQTSVDSNVKGGVGDSPQHMTAPVPSPAP-<br>RMPHLAAVEAEAEVEGSLTPPATSRRRTGATGESSSRDGRRSRGTRSTSSSD-<br>ASSAHRSPPASSGTREGASHRKRTR |

| | | | |
|---|---|---|---|
| Endotrypa-<br>num mon-<br>terogeii | Trypano-<br>somatida | NCBI\|<br>AOF-<br>S01000490 | MWVNSSASPSGQQGSSLLCDERSPALCGAGNRLSSSYSAPPAHSLPAGVLSAD-<br>LARPISVRELAQSRVPVVPPVPAAVAAAPSMQRPCDSSTAGFS-<br>GFGVPTSSVVYAATSGVPAWQRFPTRTVAGDLMSLAQRDIDRLSSDELYIYASHLQ-<br>DASKQFTAHLQEAYAKRDQYRSEVAHLKQELQSRYVEVDLLRRERERASKA-<br>LLGVHEENTTLREQLAEVEGRLRNMQAKMSVLSSDDSSLALHFYQSQLTLK-<br>EAQMRELQQDYERVAAASLQRCSTSTAGAASARHSINIPVEDGAATECSLS-<br>RDPLNSGGGQDSEEVVAVELRATLEELRSLNASLSHRLEEERAAHDAALAAH-<br>TEAIAAAAADRTELLDTIFQLRQRCTEMQASEDELVAALTQRAPISKSDYAVF-<br>QSSYNDVTAALAKAEAQITELREKERHEYASARQAQEELHSSLALGMEEKN-<br>TLQARNEQMQSLVHHLQKELELAEEANQLRQKQLDAAVNGNQGLANNLRM-<br>AQQQLLSMSSEVEDLRNTRTNLEDQVNELRRHMDEFENDDRLQRRSDEQ-<br>RGDSQTDVDVTRTASPLRESDEAVERSALKNELATLRAQRDALLRDRDDAIL-<br>ELRQLNETRHPGKLLSPSQMERPTDSSDSRNTTDPRENERRGVFRQPEEV-<br>AALEEARQCIERMNDTISTVQLEHLRVENNYKLQISALEAELESQSRALAEDK-<br>AAGVAQLAHLEEIVMALRDELVATQTQEKELRASEDAQRVRIAEQQHAVEQL-<br>QAEVASLTTENADLKEKLSTSRAAVTTLASRQTDVEGAAVSLEERCASEVQR-<br>REAAEAEVVRLREALDAANVTAAKGIEHLASLEAAREEIAEMQANRDRVEAE-<br>LESQSRALAEDKAAGVAQLAHLEEIVMALRDELLATQTQEKELRASEDAQRV-<br>RIAEQQHAVEQLQAEVASLITENADLKEKLSTSRAAVTTLASRQTDVEGAAVS-<br>LEERCASEVQRREAAEAEVVRLREALDAANVTAAKGIEHLASLEAAREEIAE-<br>MQANRDRVEAELESQSRALAEDKAAGVAQLAHLEEIVMALRDELVATQTQE-<br>KELRASEDAQRVRIAEQQHAVEQLQAEVASLTTENADLKEKLSTSRAAVTTL-<br>ASRQTDVEGAAVSLEERCASEVQRREAAEAEVVRLREALDAANVTAAKGIEH-<br>LASLEAAREEIAEMQANRDRVEAELESQSRALAEDKAAGVAQLAHLEEIVMA-<br>LRDELVATQTQEKELRASEDAQRVRIAEQQHAVEQLQAEVASLITENADLKEK-<br>LSTSRAAVTTLASRQTDVEGAAVSLEERCASEVQRREAVEVEIAAVKEALKH-<br>REGELSEVLVRGEAVQRALDEALDRLSEEEVLVEELNGDAAQHSAEKMALA-<br>ESVAMLQKSLSTAIVDKEDLSAQLVATQKEVDRAIGQQDVLLDEAERLKDALE-<br>ATTSAFAAQTREVRLGMAHIVDMSNVFCGVLEVVAGAAHSARGADGYMEQE-<br>ETATSRGSAAQPIGGRCTTAKIGEALSPWDEALEWVATMEQRLEVLDSGAIT-<br>SGLNDGGGRPARRRSRRRSGAVTGEGHDAIEAEDGCQECGLSASFMCRA-<br>VPRSRAEATLARLFCEQASAKRTIADLRVSLAEKDVELGNVRFAMDALASEA-<br>ITERDRADNLSAAIDDATKKIVSVQAEFEEQLKQRMATTTAEVVEARRAEARA-<br>TASRLCAEEQLSAVEDDMKEQQALVRTLQDENRRLTHDVDRLLRSLRSLEG-<br>SSRVAAEVRESVTPASLSPAVVSARIASQPNTRSPSIAETIEQAQILQVSSLQA-<br>DLMRTRRQTRDLKGREATLQLSCATLEKELSELRPRAREAEQLRGDLDALR-<br>ADYAELELRHDQLERMTAAAGDGTMQELKQLRQQLKVREAELEEQKLKFRD-<br>LLLSQATPELEVARRQEALRESLTDITTHLVATQAQYRAGSEDSGAIRSRRGS-<br>LAMSCRGSASLTPPPSGPSATDVLTDRMNYLQALVRDREVELEKVQAAQLE-<br>SRAATDTLRDQLVAKAAALERAEGLVAQYTDTINTLTTTAMRGGGECSGRLN-<br>TTPSLSNETDALNTKVSSPSVPPLNQLTPRRLSATLTEAYTSVAMASPVPLAP-<br>CLDEAVKIHENGDMRKNVGPSADNEVLPATSRRTGGTNTQRSTRGSSVRRS-<br>SLSTNRVSSASSSPRGSTGKVSAGTSSRKRSRT |
| Leishmania<br>enriettii | Trypano-<br>somatida | NCBI\|<br>ATAF01000<br>382 | MWDNSVYKAGAHQQSSLRPDGLPISFISSGTALSPHSHPARLARPPPAGVLSAD-<br>LARPISVRELAQSRVPAVPPPAAPPAPRPSATTAGFAGFGIPTSPVIYAATSGVST-<br>SQLLSVRADASDLTSLAHRDIDHLSRNDLYNYASHLQDASKQYSGYL-<br>LEAYTKRDQYRSEVAHLKEELHNRYMQIDVLRREQERASDALLSVREENAQLREK-<br>LAEAEGHARNMQAKMSVLSGADPSVAVHFYQSQLALKDAQLRELQQGCERAAAA-<br>SSQRRGSSTTAADRGRLSTNISSEGAAATQASPPQDLPNAGEVQDGTDAASAPLH-<br>ITLEELQCLNTSLGRRLEEEQISHKAALVAHAEASATAAADRAQLLDTIAQLRQQCT-<br>EMQAAEDELVTALTQRAPVSKKDYEALQASYDDLTAALAKAEAHVGALQEKAQQH-<br>HQSAQNAQEELRHSLSIGLEERQALQARYEHMESLVNHLQRELELAERANQLRQE-<br>QLDAALNGNQQLAEHLRVAQEQLLSATSELQDLRKTHGDLESQLRELRQQKEGQ-<br>EHGAHRPRHHEGDEGTELSLHHTPASSADREAAAVTRPTLESEIATLRSERDALVV-<br>DRDDIMEDVQRLREERLALLQSPSPQAQAEVQNHLSAVKATVDTHADSEGSKPHD-<br>HLAALEAAYNCIEHMKQTIAALTSEHDRIETELKAEVTKLEGRLASQSSVLDQAEAS-<br>AAPQQNNLEEVMVALRDELLATQAQEKRLRVSEEALFERCGVHQRSAEQLQAHL-<br>MELEGAARHAASSLAPPQSAQPRTGDSESNVVNEALESLANQLGDAQRQIKQLA-<br>DEHARTVEELARATRAVAELEARAADAEGKREASHTPAEGASVELTARIAALMMEN-<br>AQLKEKLSASRAAVTTFASKQMDGESAAASLEELCAAEARRREAAEAEVSVLRAA-<br>LGDANAAAAKNAELLAGLDTARSAVASERAECAVLRQAQEGMAAELTEVLQRSEE-<br>MQATLECTLTRLAEQEALVERLNREALEFHAQRPALEREVAELRVALAVAMTDKVD-<br>VVAQLVATQKELDRAAEVHDEHYREEERLHEALSTTTSAFAVQTAQVRLGMAHVV-<br>DMSRAFCGVAEAVAQRAACSMSISEGVKREAAARSSKAVRLAAPTGDALISVKCLR-<br>EMLLPWTQALEWVAAMEKRLDALDAGAISGRTSSAGGHGERRRRRRGRAASAEE-<br>EDEVVFEAGNSALVLDESFLCRAVPTSHAEAAFARWMDVQSAAANIIAELRSALAE-<br>REAEVNSLRSAMDALATELMDEQDRADNLSAAIDDATEKIASAQAEFEEQLRQRTA-<br>ATTAEVVEARRAEARATAAQLRAEERLTVVEDDLKEQQALLRNLQDENRRLTRKAD-<br>CLLHSSRLTDPDSRDRSKVGDSATPQSLSPATPAAEGAAKRFATAASAVEAVEEALI-<br>LQVSSLQADLMLSRRHARELEGREATVRQACLALEQQVSELRVEAADTGQLREDF-<br>AALRADYTDLEQRYDELERMTTAAGGGTMQELKHLRQQLKVQEAELCESKARLRG-<br>LVLRKATPELAATRRQEALRESLSGITAQLAATQAQYGGCTGGSGSARSRGASLAA-<br>AVSNSDPLNRSAPSPSSQDVLTSRIEHLQLVVRNREMALDKVQAAQLESRAAADTL-<br>EDQLAMKTAALERAEGLVAEYADTIDTLTMTVLAGRGSRGARPTTTRPVRSVEGSP-<br>HEGAPSPSVPPLTPLSTRTRAARSEQPHAVAAEASPAPRMPSLDEVADDNNCRDS-<br>GKGVDSPRSSSSTPTPSATRRRTGTSSNREGAEGSATCLPSSSTSTKRSALSSPS-<br>NGSGRKSLAGATMRATTRKRDRSRSTA |

| | | | |
|---|---|---|---|
| Leishmania braziliensis | Trypano-somatida | NCBI\|<br>XP_001565<br>950 | MWGNDFYKAGAHQQPSLRHDGLPTSFASPSYTAQPARPLPAGVLSAD-<br>LARPISVRELAHSRVPAVPPPPAVPPAPRRNPAMTGFAGFGIPTSPIIYAATS-<br>GVSTLPPLSTRADASDLMSLAHRDIDNLSGDDLYNYASHLQGASKQYSKHL-<br>LEAYTRRDQYRGEVARLKEELHSKYLQIDVLRREQERASEALVKVREDNA-<br>QLHQKLAESEGHVRNMQAKVSVLSGADPSAAVHFYQSQLVLKDAQLRE-<br>LQQQCEREAAASSQRHSSSATAAEHGRRSINVSGEGAAVPQISPLRDLP-<br>STRKVHNGSEAASTALHTALEELQHLNTSLSRRLEEEHAAHKAALAVHAE-<br>ENATATADRAELLNTISQLRQQCTEMQSTEDELVAALTQRAPISKEGYAAL-<br>QTSYNDLTAALAKAEDQIAALHVKEQQYHQSAQQAQEELRHSLTTGLEE-<br>RQELQARNEQMQSLATDLQRELELAERANQLRQEQLDAAVSGNQQLAE-<br>HLRTTQEQLLGATAELQDLCNTRGSLESQLRELRQRIEEFEQEDQRRRQ-<br>QHRDVEGGATFSYTPAASAESETVKTREALEDELATLRSQRDALVGDRDE-<br>IISEVRRLQEDRLALLRSPLPQAEEVLQKHLSTVKATLGTHADDEELPQQE-<br>DIAALEAARHCIEQLKETIATLQSERVRVEAELQAKIKVLEAELASQSRALS-<br>DAEDAAAAQRAHLEGIVVALRDELVATQEQQMQFITSEEAQRERCAEQQ-<br>RAVEHLQAQVAQLQMAGQDSANSLATTWHAQSPVPAKSDLSTMNAALE-<br>SLAHQLEDAQYRAEELSAEHARTLEELARATRAAAELEARAAEAESRRE-<br>AAHAAAEGTEVELTARIAALATENAQLKEKLSASRAAVTTFASKQTDGEST-<br>AASLEERCATEARRREAAEAEALVLRETLDRTGAELTEVLQRSEGVQSTL-<br>TRTVDRLAEQEALVGVLGAENMRLQDELTAVRESILLLTSERDALVEETAQ-<br>LHGKLAAAESRMSDAAAAQQRLEVRLEEAEAAVRSKAVELGTVFAALDQ-<br>TASEVCGAEQQLRVYAEAKEELSAEHARTLEELARATRAAAELEARAAEA-<br>ESRREAAHAAAEGTEVELTARIAALATENAQLKEKLSASRAAVTTFASKQT-<br>DGESTAASLEERCATEARRREAAEAEALVLRETLDRTGAELTEVLQRSEG-<br>VQSTLTRTVDRLAEQEALVGVLGAENMRLQDELTAVRESILLLTSERDALV-<br>EETAQLHGKLAAAESRMSDAAAAQQRLEVRLEEAEAAVRSKAVELGTVF-<br>AALDQTASEVCGAEQQLRVYAEAKEELSAEHARTLEELARATRAAAELEA-<br>RAAEAESRREAAHAAAEGTEVELTARIAALATENAQLKEKLSASRAAVTT-<br>FASKQTDGESTAASLEERCATEARRREAAEAEALVLRETLDRTGAELTEV-<br>LQRSEGVQSTLTRTVDRLAEQEALLEQLNRNASELNDARAVLTGEVTRLQ-<br>AALAAAVTDKSDVMAQLVATQRELDRAVEQQDAQYCEEVRLQDALTTTM-<br>SAFGVQTRQVQLGMAHVVDMSKAFYRVVEVVAKGAASATVQGEIVDKE-<br>AAAQSFASVGVGSSVKGASPTATSVREILLPWSRALEWVASMEERLESL-<br>GTGSTSSDTTSSDSQCERRRRRRGRSTVADEEEEAIDEEETSGADAVST-<br>SFLCRAVPASHAEAAFARWIWEQAAAASTIADLRSALADKEVELNNLRSA-<br>MDTLANDAMAEQDRADNLSAAIGDATEKIALVQAEFEEQLRQRTVATTAE-<br>VVEARRAEERARAAQLRAEEHLTVVENELKEQQAVLRKLQDENHRLTRE-<br>ADRLRRSSRHLESDSRALNKISDSVTPQPLSYAGVSAVTEVESFTTAASA-<br>VEVVEQAQFLQVFSLQEDLMLSRRQARELEGREATLRQTCATLEQQVSQ-<br>LRVEATDAEKLREELATLRTDYAELEERYDQLERMTTAAGGGTMQELKQ-<br>LRQQLKARETELDELKARVRGLVLSKVAPELEAARRQEALRESLSGITTQ-<br>LAATQAQYGGGTGGSSNAGSRRASLASALSKPDHLSLSASSPSSQDLLTS-<br>RIEHLQILVHDHQEAFEKVQAAQLESRATMDTLEDQLAAKTAALERAEGLV-<br>AEYADTINVLTMTAVVGRGSGGAHLSTVQPLRNEVANLVAEVPSLSVLPLT-<br>PLTPRPDVAADGQPRAAVAVASPTPRMPNLDEVAANNDGSVTDVEGDSA-<br>GSSADTALPSATHRRTDAEGSRRSARGTPARRSSSSSSSNNTARKSFVGVT-<br>MIAKARKRARSSTTA |
| Leishmania mexicana | Trypano-somatida | NCBI\|<br>XP_003876<br>756 | MSGSRTSLSSPSYAAQPARPPPAGVLSADLARPISVRELAQSRVPAVPPPPPATQT-<br>PRPSATTAGFAGFGVPTSSAVYAATSGVPTLQLLSTHTDAGDLTSLAHRDVDHLS-<br>GNELYNYASHLQESSKQYSGYLSEAYAKRDQYRGEVARLKEELHNRYMQIDVLR-<br>REQARATDALVKVREDNAQLRQKLAEAEGHTRNMQAKMSVLSGADPS-<br>IAVHFYQSQLALKDAQLRELQQECERAAAASSQRRSSSTTAAEHGRRS-<br>ANISGEGAAAPHTSPSCDLPSTEELQERAEAASAALPTALEELQRLNISL-<br>SHRLEEEHAAHKAALAAHAEASATARADRAELLDTIAQLRQQCADMQSA-<br>EDELVAALTQRAPISKKDYAALQASYDDLTAALAKAEAQVAALQEKEQQH-<br>DHIARQAEEELRHLLTTGQEERQSMQARNEHMQSLVYHLERELELAER-<br>ANQLRQEQLDAAISGNQQLAESLHVAQEQLLSATAELQDLRDTCESLE-<br>SQLRELQQRIEELEQADQHQRHQHGDAEAERTLIHTSAASAESEALQQ-<br>RQELEAELASLCSQRDALVGDRDDIIGEMRRLQDERLALLTSPPAQAEE-<br>QLQRRSSAVKAPQDVRADDGEWQAQEDIAALEAAHQCIEQMKQTIAAL-<br>QSESARVEADLKAEVNTLKAELASQSRALTGAQAAAAAQQAHLEEVMLA-<br>LRDELVATQAQEKRLRASEEAHRERCVEQQRVMEQLQQHVEELERAA-<br>RDAASSLSTPPPDQPTTAAESELSAMNAALDSLANELEDAQLRAEQLA-<br>AEHARTVEELARGTRAVAELEARAADTEGKREAMHTAAEATEAELMAR-<br>VAALAAENAQLKEKLSTSRAAVTTFASRQTDGESTAAPLEERCAAAARR-<br>SEAAEAEASALREALSRSAAELVEVLQRSEDVQATLERTVARLAEQESL-<br>VGVLSAESVRVQDELASAQRCVLMLTSERDALGEEAAGLHGKLSAAED-<br>RASDAAAAQKRLQMRLEEAEAAVRSKDVELSTAFAALDRTASELCDAE-<br>QRLQAYSEAKEQLAAEHARTVEELARGTRAVAELEARAADTEGKREAM-<br>HTAAEATEAELMARVAALAAENAQLKEKLSTSRAAVTTFASRQTDGEST-<br>AAPLEERCAAAARRSEAAEAEASALREALSRSAAELVEVLQRSEDVQAT-<br>LERTVARLAEQESLVGVLSAESVRVQDELASAQRCVLMLTSERDALGE-<br>EAAGLHGKLSAAEDRASDAAAAQKRLQMRLEEAEAAVRSKDVELSTAF-<br>AALDRTASELCDAEQRLQAYSEAKEQLAAEHARTVEELARGTRAVAEL-<br>EARAADTEGKREAMHTAAEATEAELMARVAALAAENAQLKEKLSTSRAA-<br>VTTFASRQTDGESTAAPLEERCAAAARRSEAAEAEASALREALSRSAAE-<br>LVEVLQRSEDVQATLERTVARLAEEEALVEQLNYDASELNVARAALSDE-<br>VTRLQRALAVALTDKSDVVAQLLATQKELDRAAELQDAQYRDEERLQDA-<br>LATTTSAFDVQTKQVRLGMAHVVDMSRAFCRVAEVAVQWSAAQTDGL-<br>ASTGASVQEMLSPWSRALEWVAGMEQRLEALDIGAMLNSFSRTDGR-<br>RERRRRRRRGRTTESEEDDAAEEEPSSGSNALSLSSLCRAVPANRAEV-<br>AFARLMDAQAAAMNTIADLRRTLADKEADLTNLRSAMDALASDLMGER-<br>DRADNLSAAIDDASEKIGSAQAEFEEQLRQRTAATTAEVVEARRAEARA-<br>TAAQLRAEKQLAAVENDRKEQEAVLRKLQDENHRLAREADRLLRTSRL-<br>EPSSRVRSNGSGSVTPKSLSLSATSAEDAVQRFTTAPSTVEAAEQAQIL-<br>QVSSLQADLMLSRRQARELEGREATLRQACAALEQQVNELRVEAAEAE-<br>HLREDLATLRADYAELEQRYSELERMATAAGGGTMQELKQLRQQLKVR-<br>EAELEELKARMQDLVLSKAAPELEAARRQEALRESLSGITTQLAATQA-<br>QYGGGTGGSNKARSRRASLTDALSNSDLLSLSSSRPSPQDVLTSRIEH-<br>LQTLVHDREVALEKVQAAQLESRAAMDMLEDQLAAKTAALERSEGLVAE-<br>YADTINTFTMTAVVRRGNHGARPTTAPPLSSGMGSPDAEALSTSVPPLT-<br>PLTPRTRAAVNGQSHTAVAMASPAPRMPNLDEVADDSDGSVEGKHAD-<br>SAGPSTDSAPPPVTHRRTGASGSWRSAKSCPAHHPSSSTERSAPSSP-<br>STVPGRKSSAGASMPAATRKRTRSSTNA |

| | | | |
|---|---|---|---|
| Leishmania amazonensis | Trypanosomatida | NCBI\| APN-T01003049 | MWGNSFYKAGTHQQSSLCHDGQPVTMSGSRTSLSSPSYAAQPARPPPAGVLSAD-<br>LARPISVRELAQSRVPAVPPPPPATQTPRPSATTAGFAGFGVPTSSAVYAATS-<br>GVSTLQLLSTHADAGDLTSLAHRDVDHLSGNELYNYASHLQESSKQYS-<br>GYLSEAYAKRDQYRGEVARLKEELHNRYMQIDVLRREQARATDALVKVREDNAQL-<br>RQKLAEAEGHTRNMQAKMSVLSGADPSIAVHFYQSQLALKDAQLRELQQECERA-<br>AAASSQRRSSSTTAAEHGRRSANTSGEGAAAPHTSPSCDLPSTEELQERAEAASA-<br>VLPTALEELQRLNTSLSHRLEEEHAAHKAALAAHAEASATARADRAELLDTIAQLRQ-<br>QCADMQSAEEELVAALTQRAPISKKDYAALQVSYDDLTAALAKAEAQVAALQEKEQ-<br>QHDHIARQAEEELRHLLTTGQEERQSMQTRNEHMQSLVYHLERELELAERANQLR-<br>QEQLDAAISGNQQLAESLHVVQEQLLSATAELQDLRDTRESLESQLRELQQRIEEL-<br>EQADQHQRHQHGDAEAERTLIHTSAASAESEALQQRQELEGELASLRSQRDVLV-<br>GDRDDIISGMRRLQDERLALLTSPPAQAEEQLQRRSSAVKAPQDVCADDGEWQA-<br>QEDIVALEAAHQCIEQMKQSIAALQSESARVEADLKAEVNTLKAELASQSRALAGA-<br>QAAAAAQQAHLEEVILALRDELVATQAQEKRLRASDEAHRERCVEQQRAMEQLQK-<br>HVEELERAARDTVSSFSTPPPDQPSTAAESELSAMNAALDSLAHELEDAQLRAEQ-<br>LAAEHARTVXXXXXXXXXXXXXXXXXXXQATLERTVARLAEQESLVGVLSAESVRV-<br>QDELAAAQQCVLMLTSERDALGEEAAGLHGRLSAAEDRASDAAGAQKRLQMRLE-<br>EAEAAVRSKDVELSTAFAALDRTASELCDAEQRLQAYSEAKEQLAAEHARTVEELA-<br>RGTRAVAELEARAADTEGKREAMHTAAEATEAELMARVAALAAENAQLKEKLSTS-<br>RAAVTTFASQQTDGESTAASLEERCAAAARRSEAAEAEASALREALSRSAAELVEV-<br>LQRSEDVQATLERTVARLTEEEALVEQLNYDASELNVARAALSDEVTRLQKALAVAL-<br>TDKSDVVAQLLATQKELDRAAELQDAQYRDEERLQDALATTTSAFDVQTKQVRLG-<br>MAHVIDMSRAFCRVAEVTVQWSAAQTDGLASTGASVQEMLSPWSRALEWVAGM-<br>EQRLEALDIGAMLNSFSRTDGRRERRRRRRGRTTESEEEDAAEEEPSSGSNALS-<br>LSSLCRAVPANRAEVAFARLMDAQAAAMNTIADLRRTLAEKEADLTNLRSATDALA-<br>SDLMGERDRADNLSAAIDDASEKIASAQAEFEEQLRQRTAATTAEVVEARRAEARA-<br>TAAQLRAEKQLAAIENDRKEQEAVLRKLQDENHRLAREADRLLRTSRLEASSRVRS-<br>NGSGSVTPKLLSLSATSAEDAVQRFTTAPSTVEAVEQAQILQVSSLQADLMLSRRQ-<br>ARELEGREATLRQACAALEQQVNELRVEAAEAEHLREDLATLRADYAELEQRYSEL-<br>ERMATAAGGGTMQELKQLRQQLKVREAELEELKVRMRDLVLSKAAPELEAARRQ-<br>EALRESLSGITTQLAATQAQYGGGTGGSNKARSRRASLTDALSNSDLLSLSSSRPS-<br>PQDVLTSRIEHLQTLVHDREVALEKVQAAQLESHAAMDMLGDQLAAKTAALERSEG-<br>LVAEYADTINTFTMTAVVRRGNHGARPTTAPPLSSGMGSPDAEALSASVPPLTPLTP-<br>RTRAAVNGQSHTAVAMTSPAPRMPNLDEVADDSDGSVEGKHADSAGPSTDSAPP-<br>PVTHRRTGASGSWRSAKSCPAHRPSSSTERSAPSSPSTVPGRKSLAGASMPAAT-<br>RKRTRSSTNA |
| Leishmania infantum | Trypanosomatida | NCBI\| XP_001466421 | MWGNNFCKAGAHQQSALCCDGQPATMSGSRTSLPSPSYAAQPARPPPAGVLSAD-<br>LARPISVRELAQSRVPAVPPPPPATQAPRPSATTAGFAGFGVPTSSAVYAATSGVST-<br>SRPLSTHADAGDLTSLAHRGIDHLSGSELYNYASHLQESSRQYSGYL-<br>SAAYAKRDQYRGEVAHLKEELHNRYVEIDALRREQERASDALLKVREDNAQLRQK-<br>LAEAEGHARNMQAKMSVLSGADPSIAVHFYQSQLALKDAQLRELQQECERAAAAS-<br>SQRRSSSTTAAEHGRRSANISGEGAAAPHTSPSRDLPSAEELQDGAEAASAALHT-<br>ALEELQCLNTSLSHRLEEEHTAHKAALAAHAEASATATADRAELLDTIAQLRQQCA-<br>DMQSAEDELVAALTQRAPISKKDYASLQASYDDLTAALAKAEAQVAALQEKEQQHH-<br>HSAQQAQEELRHSLTTGLEERQSMQARNEQMQSLVNNLERELELAERANQLRQE-<br>QLDVAISGNQQLAESLHVAQEQLLSATSELQDLRDTRESLESQLRELRQRIEELEQ-<br>GEQHQRHRHGGAEAERTLIHTPAASAESEASQQRQESEAELASLRSQRDAVVGD-<br>RDDIIGEVRRLPDERRALLISPPAHAEEQLQSRSSAVRAPLDVRADDREWQKQEDI-<br>AALEAAHQCIEQMKQTIAALQSERVRVEADLKAEVNTLEAELASQSRALAEAEAAA-<br>AAQQAHLEEVMAALRDELVATQAQEKRLRASEEAHRERCAEHQRAVEQLQAHVE-<br>ELERAARDAASSLPTPPPAQPSTAAESELRAMNAALDSLAYQLEDAQHRVKELSA-<br>EHARTVEELARATRAVAELEARAADAEGKHEVMHTAAEVTEAELMARVAALTAENA-<br>QLREKLSTSRAAVTTFASRQTDGESAAASLEERCAAEARRSEAAEAEASALRETL-<br>NGTAAELAEVLQRSEDVQATLERTVARLAEQESLVGVLSAESTRLQDELATAQRSV-<br>LLLTSERDALGEEAAGLRGKISAAEDRASDAAAAQQRLQACLEEAEAAVRSKDVEL-<br>STALAALDRTASEVCDAEQRLQVYSEAKEQLAAEHARTVEELARATRAVAELEARA-<br>ADAEGKHEVMHTAAEVTEAELMARVAALTAENAQLREKLSTSRAAVTTFASRQTD-<br>GESAAASLEERCAAEARRSEAAEAEASALRETLNGTAAELAEVLQRSEDVQATLER-<br>TVARLAEQESLVGVLSAESTRLQDELATAQRSVLLLTSERDALGEEAAGLRGKISAA-<br>EDRASDAAAAQQRLQACLEEAEAAVRSKDVELSTALAALDRTASEVCDAEQRLQV-<br>YSEAKEQLAAEHARTVEELARATRAVAELEARAADAEGKHEVMHTAAEVTEAELM-<br>ARVAALTAENAQLREKLSTSRAAVTTFASRQTDGESAAASLEERCAAEARRSEAAE-<br>AEASALRETLNGTAAELAEVLQRSEDVQATLERTVARLAEQEALVEQLNYDASELN-<br>DARAVLTDEVTRLQKALAAAVSDKADVVAQLLATQKELDRAAEAQDAQYRDEERL-<br>QDALATTTSAFDVQTTQVRLGMAHVVDMSRAFCRVAEVAVQRSASQTDGASGTG-<br>TSVQEILSPWTRALEWVAAMEQRLEALDRGAMPNSFTRTNGRRERRRRRRGRTT-<br>ESENDDAVEMESNSGSYVLGTSPLCRAVPANRAEAAFARLIDEQAAATNTIADLRR-<br>TLADKEVDLTNLRSAMDALASDLMGERDRADNLSAAIDDASEKIASAQAEFEEQLR-<br>QRTAVTTAEVVEARRAEARATAAQLRAEEQLAAVEKDLKEQEAVLRKLQDENHRLT-<br>READRLLRTSRLEPSSRARSNVSGSVTPKSLSLSATSAENAAQRFTTAPSTLEAIE-<br>QAQILQVSSLQADLMLSRRQARELEGREATLRQACAALEQQVSELRAKAAEAEHL-<br>REDLATLRADYVELEQRYSELELMATAAGGGTMQELKQLRQQLKVREAELEEVKA-<br>RMRDLVLSKAAPELQAARRQEALRESLSGITTQLAATQAQYGGGTGGSSKARSRR-<br>DSLTAALSNSDLNLSSSRPSPQDVLNSRIEHLQTLVHDREVALEKVQAAQLESRAA-<br>MDTLEDQLAAKTAALERAEGLVAEYADTINAFTMAAVARRGNRGAVSTTVPPLSSG-<br>MGSPDAEALSASVPTLTPLTPRTRAAVNGQPHAAVAMVSPAPRMPNLDEVADDSD-<br>GSGEGKQADSAGPSTDSAPPPVTHRRTSASGSWRGAKSCPAHRPSSSTERSAP-<br>SSPSNVPGRKSFAGASVPATTRKRARSSTNS |
| Leishmania donovani | Trypanosomatida | NCBI\| AVPQ01001269 | MWGNNFYKAGTHQQSALCCDGQPATMSGSRTSLPSPSYAAQPARPPPAGVLSAD-<br>LARPISVRELAQSRVPAVPPPPPATQAPRPSATTAGFAGFGVPTSSAVYAATSGVST-<br>SRPLSTHADAGDLTSLAHRDIDHLSGSELYNYASHLQESSRQYSGYL-<br>SAAYAKRDQYRGEVAHLKEELHNRYVEIDALRREQERASDALLKVREDNAQLRQK-<br>LAEAEGHARNMQAKMSVLSGADPSIAVHFYQSQLALKDAQLRELQQECERAAAAS-<br>SQRRSSSTTAAEHGRRSANISGEGAAAPHTSPSRDLPSAEELQDGAEAASAALHT-<br>ALEELQCLNTSLSHRLEEEHTAHKAALAAHAEASATATADRAELLDTIAQLRQQCA-<br>DMQSAEDELVAALTQRAPISKKDYASLQASYDDLTAALAKAEAQVAALQEKEQQHH-<br>HSAQQAQEELRHSLTTGLEERQSMQARNEQMQSLVNHLERELELAERANQLRQE-<br>QLDVAISGNQQLAESLHVAQEQLLSATSELQDLRDTRESLESQLRELRQRIEELEQ-<br>GEQHQRHRHGGAEAERTLIHTPAASAESEASQQRQESEAELASLRSQRDAVVSD-<br>RDDIIGEVRRLPDERRALLISPPAHAEEQLQSRSSAVRAPLDVRADDREWQKQEDI-<br>AALEAAHQCIEQMKQTIAALQSERVRVEADLKAEVNTLEAELASQSRALAEAEAAA-<br>AAQQAHLEEVMAALRDELVATQAQEKRLRASEEAHRERCAEHQRAVEQLQAHVE-<br>ELERAARDAASSLPTPPPAHPSTAAESELRAMNAALDSLAYQLEDAQHRVKELSAE-<br>HARTVEELARATRAVAELEARAADAEGKREVMHTAAEVTEAELMARVAALTAENAQ-<br>LREKLSTSRAAVTTFASRQTDGESAAASLEERCAAEARRSEAAEAEASALRETLNG-<br>TAAELAEVLQRSEDVQATLERTVARLAEQESLVGVLSAESTRLQDELATAQRSVLLL-<br>TSERDALCEEAAGLRGKISAAEDRASDAAAAQQRLQACLEEAEAAVRSKDVELSTA-<br>LAALDRTASEVCDAEQRLQVYSEAKEQLAAEHARTVEELARATRAVAELEARAADA-<br>EGKREVMHTAAEVTEAELMARVAALTAENAQLREKLSTSRAAVTTFASRQTDGESA-<br>AASLEERCAAEARRSEAAEAEASALRETLNGTAAELAEVLQRSEDVQATLERTVAR-<br>LAEQEALVEQLNYDASELNDARAVLTDEVTRLQKALAAAVSDKADVVAQLLATQKE-<br>LDRAAEAQDAQYRDEERLQDALATTTSAFDVQTTQVRLGMAHVVDMSRAFCRVA-<br>EVAVQQSASQTDGASGTGTSVQEILSPWTRALEWVAAMEQRLEALDRGAMPNSF-<br>TRTNGGRERRRRRRGRTTESENDDAVETESNSGSYVLGTSPLCRAVSANRAEAA-<br>FVRLIDEQAAATNTIADLRRTLADKEVDLTNLRSAMDALASDLMSERDRADNLSAAI-<br>DDASEKIASAQAEFEEQLRQRTAVTTAEVVEARRAEARATAAQLRAEEQLAAVEKD-<br>LKEQEAVLRKLQDENHRLTREADRLLRTSRLEPSSRACSNVSGSVTPKSLSLSATS-<br>AENAAQRFTTAPSTLEAIEQAQILQVSSLQADLMLSRRQARELEGREATLRQACAA-<br>LEQQVSELRAKAAESEHLREDLATLRADYVELEQRYSELELMATAAGGGTMQELK-<br>QLRQQLKVREAELEEVKARMRDLVLSKAAPELQAARRQEALRESLSGITTQLAATQ-<br>AQYGGGTGGSSKARSRRDSLTAALSNSDLNLSSSRPSPQDVLNSRIEHLQTLVHD-<br>REVALEKVQAAQLESRAAMDTLEDQLAAKTAALERAEGLVAEYADTINAFTMAAVA-<br>RRGNRGAVSTTVPPLSSGMGSPDAEALSASVPTLTPLTPRTRAAVNGQPHAAVAM-<br>VSPAPRMPNLDEVADDSDGSGEGKQADSAGPSTDSAPPPVTHRRTSASGSWRG-<br>AKSCPAHRPSSSTERSAPSSPSNVPGRKSFAGASVPATTRKRARSSTNS |

| | | | |
|---|---|---|---|
| Leishmania tropica | Trypano-somatida | NCBI\| compound of: ATAT01001197 and ATAT01001196 | MWGNDFHQAGTHQQSSLCCDGQPVTMPGSRTSLPSPAYAAQPARPPPAGVLSAD-<br>LARPISVRELAQSRVPAVPPPPPATQTPRPRATTAGFAGFGVPTSSAVYAATSGVST-<br>SQPLSAHADAGDLTSLAHRDIDRLSGRELYNYASHLRESSQQYG-<br>GYLSEAYAKRDQYRGEVARLKEELHNRYMEIDVLRRQQERASDALLRVREENAQL-<br>RQKLAEAEGHARNMQAKMSVLSGADPSLAVHFYQSQLALKDAQLRELQQECERA-<br>AAASSQRRSSSTTAAEHGRRSANISGQCAAAPHTSPSCDLPSAEELQDGTEAASA-<br>ALHTALEELQCLNKSLSHRLEDEHTAHKAALAAHAEASATATADRAELLDTIAQLRQ-<br>QCADMQNAEDELVAALTLRAPISKKDYAALQASYDDLTAALAKAEAQVAALQEKEQ-<br>QYHHSAQQAQEELRHSLTTGLEERQSMQVRNEQMQSLVNHLERELELAERANQL-<br>RQEQLDAAISGNQQLAESLHVAQEQLLSATSELQDLRNTRESLESQLRELRQRIEE-<br>LEQGDQHQRHQHGDAEAEIEASQQRQQLEAELARLRSQRDAPVGNRDDIIGDVR-<br>RLPEERSALLTSTPAHAEEQLQSPSSAVRAPLDVRADNGEWQKQEDIAALEAAHRC-<br>IEQMKQTIAALESARMRVEADLKAEVNTLEAELASQSRALAEAEAAAAAQQAHLEE-<br>AIMALRDELVATQAQEKLLRASEEAHRERCAEQQRAVDQLQAHVEELERAGRNAIS-<br>SLSTPPPAQPATAAESELIAMNAALNSLAYQLEDAQHRVEELSAEHARTVEELARAT-<br>RAVAELEARAAGVEGKREVVHTAAEAAEAELMARVAALTTENAQLKEKLSTSRAAV-<br>TTFAXXXXXXXXXXXXXXXXXXXXXXXXXXXXXXXXXVLRRSEDVQATLERTVARLA-<br>EEEALVEQLNFDASELNDARAALTEEVTRLQKALAAAVTDKADVVAQLLATQKELD-<br>RAAEVQDAQYRDEERLQDALATTTSAFDVQTKQVRLGMAHVVDMSRAFCRVAEV-<br>AVQRSAAQMDGAASTGASVQEMLSPWTRALEWVAAMEQRLEALDMGAMSNCFS-<br>RTDGRRERRRRRRGRATELEDDNAVEMEANSGSHALSSSSLCRAVSANCAGAAF-<br>ARLMDEQAAATNTIADLRRTLADKEVDLTNLRSAMDALASDLMSEQDRADNLSAAI-<br>DDASEKIASAQAEFEEQLRQRTEATTAEVVEARRAEARATAAQLRAEEQLAAVEKD-<br>LKEQEGVLRKLQDENHRLTREADRLLRTSRLEPSSRVRSNVSGSVTPKSLSLSAAS-<br>AEDAAQRFTTAPSTLEAVEQAQILQVSSLQADLLLSRRQARELEGREATLRQACAA-<br>LEQQISELRVEAAEAEHLREDLATLRADYAELEQRYSELERMATAAGGGTMQELKQ-<br>LRQQLKVREVELEELKARMRDLVLSKAAPELEAARRQEALRESLSGITTQLAATQA-<br>QYGGGTGGSSKVRSRRASLTAALSNSDLLSLSSSRPSPQDVLTSRIEHLQTLVNDR-<br>EVALEKVQAAQLESRAAIDTLEDQLAAKTAALERAEGLVAEYADRINNAFTMAAVVR-<br>RSSHGAGPTTVPPLSSGMGSPDAEALSASVPPLTPLTPRTRAAVNGQPHAAVAMV-<br>SPAPRMPNLDEVADDSDGSGEGNQAGSAGPSTDSAPPPVTHRRTRASGSWRDA-<br>KSCPAHRPSSSTERRAPLSPSNVPGRKSFAGASVPATTRKRARSSTNA |
| Leishmania aethiopica | Trypano-somatida | NCBI\| compound of: AUM-B01001107 and AUM-B01001108 | MWGNNFHQAGTHQTSSLCCDGHPVTMPGSRTSLPSPPYAAQPARPPPAGVLSAD-<br>LARPISVRELAQSRVPAVPPPPPATQTPRPRATTAGFAGFGVPTSSAVYAATSGVST-<br>SQPLSAHADAGDLASLAHRDIDRLSGRELYNYASHLRESSQQYG-<br>GYLSEAYAKRDQYRGEVARLKEELHNRYMEIDVLRRQQERASDALLRVREENAQL-<br>RQKLAEAEGHARNMQAKMSVLSGADPSLAVHFYQSQLALKDAQLRELQQECERA-<br>AAASSQRRSSSTTAAEHGRRSANISGECAAAPHTSPSCDLPSAEELQDGTEAASA-<br>ALHTALEELQCLNTSLSHRLEDEHTAHKAALAAHAEASATATADRAELLDTIAQLRQ-<br>QCADMQNAEDELVAALTLRAPISKKDYAALQASYDDLTAALAKAEAQVAALQEKEQ-<br>QHHHSAQQAQEELRHSLTTGLEERQSMQVRNEQMQSLVNHLERELELAERANQL-<br>RQEQLDAAISGNQQLAESLHVTQEQLLSATSELQDLRDTRESLESQLRELRQRIEE-<br>LEQGDQHQRHQHGDAEAESEASQQRQQLEAELARLRSQRDALVSNRDDIIGDVR-<br>RLPDERRALLTSPPAHAEEQLQSRSSAVRAPLDVRADNGDWQKQEDIAALEAAHR-<br>CIEQMKQTIAALQSERVRVEADLKAEVNTLEAELALQSRALAEAEAAAAAQQAHLE-<br>EAMMALRDELVATQAQEKLLRASEEAHRERCAEQQRAVNQLQAHVEELERAGRN-<br>AVSSLLTPPPAPPATAAESELIAMNAALNSLAYQLEDAQHRVEELSAEHARTVEELA-<br>RATRAVAELEARAAGVEGKREXXXXXXXXXXXXXXXXXXXXXXXXXXXXGTLNRTA-<br>TELAEVLRRSEDVQATLERTVARLAEEEALVEQLNFDASELNDARAALTEEVTRLQ-<br>KALAAAVTDKADVVAQLLATQKELDRAAEAQDAQYRDEERLQDALATTTSAFDVQT-<br>KQVRLGMAHVVDMSRAFCRVAEVAVQRSAAQMEGAASTGASVQEMLSPWTRAL-<br>EWVAAMEQRLEALDMGAMSNSFSRTDGRRERRRRRRGRTTELEDDNAVEMEAN-<br>SGSHALSSSSLCRAVSANRAEAAFARLMDEQAAAANTIADLRRTLADKEVDLTNLR-<br>SAMDALASDLMSEQDRADNLSAAIDDASEKIASAQAEFEEQLRQRTAATTAEVVEA-<br>RRAEARATAAQLRAEEQLAAVEKDLKEQEGVLRKLQDENHRLTREADRLLRTSRLE-<br>PSSRVRSDVSGSVTPKSLSLSAASAADAAQRFTTAPSTLEAVEQAQILQVSSLQAD-<br>LLLSRRQARELEGREATLRQACAALEQQISELRVEAAEAEHLREDLATLRADYAEL-<br>EQRYSELERMATAAGGGTMQELKQLRQQLKVREVELEELKARMRDLVLSKAAPEL-<br>EAARRQEALRESLSGITTQLAATQAQYGGGTGGSSKVRSRRASLTAALSNSDLLSL-<br>SSSRPSPQDVLTSRIEHLQTLVNDREVALEKFQAAQLESRAAMDTLEDQLAAKTAA-<br>LERAEGLVAEYADSINNAFTMAAVVRRGNHGAGPTTVPSLSSGMGSPDAEALSAS-<br>VPLLTPLTPRTRAAVNGQPCAAVAMASPAPRMPNLDEVADDSDGSGEGKQAGSA-<br>GPSTDSAPPPVTHGRTRASGSWRDAKGCPAHRPSSSTERSAPLSPSNVPGRKFF-<br>AGASVPATTRKRARSSTNA |
| Leishmania major | Trypano-somatida | NCBI\| XP_003721989 | MWGNNFHNAGAHQQSSLCCDGQPAIMPSSHTSLPFPSYGEQPARPPPAGVLSAD-<br>LARPISVRELAQSRVPAVPPPPPATQTPRSRATTAGFAGFGVPTSSAVYAATSGVST-<br>SQPLPAHADAGDLTSLAHRDIDHLSGRELYNYASHLRESSQQYG-<br>GYLSEAYAKRDQYRGEVARLKEELHNRYMEIDVLRREQERARDALLKVREDNAQL-<br>RQKLAEAEGHARNMQAKISVLSGADPSLAVHFYQSQLALKDAQLRELQQECERAA-<br>AASSQRRSSSTTAAEHGRRSANISGEGAAAPHTSPSCDLPSAEELKGGTEAASAA-<br>LHTALEELQCLNTSLSHRLEDEHTAHKAALAAHAEASATATADRAELLDTIAQLRQQ-<br>CADMQNVEDELVAALTLRAPISKKDYAALQASYDDLTAALAKAEAQVAALQENEQQ-<br>HHHSAQQAQEELRRSLTTGLEERQSMQVRNEQMQSLVNHLEKELKLAERANQLR-<br>QEQLDAAISGNQQLAESLHVAQEQLLSATSELQDLRDTRESLESQHRELRQRIEEL-<br>EQGDEHQRHQHGEAEAESEASQQRQELEAELARLRSQRDVLVGDRDDIVGDVRR-<br>LPDERRALLTSPPAHAEEQLQSRSSAVRAPLDVHADDGERQNQEDIAALEAAHRCI-<br>EQMKQTIAALQSERVRVEADLKAKVNTLEAELASQSRALAEAEAAAAAQQAHLEE-<br>VVMALRDELVATQAQEKMLRASEKAHQERCVEQQRAVEQMQAHVEELKTAARNA-<br>ASSLSTPPPARPATAAESEQIAMNAALSSLAYQLEDAQHRVEELSAEHARTVEELAR-<br>ATRAVAELEARAAGAEGKREVMHTAAEVAEAELMARVAALTTENAQLKEKLSISRA-<br>AVTTFASRQTDGEGAAASLEERCAAEARRSEAAEAEASALRGTLDRTAAELAEVL-<br>QRSEDVQATLERTVARLAEQESLVGALSAESTRLQDELATAQRSVLLLTGERDALG-<br>EEAAGLRGELSAAEDRASDVAAAQQRLQVRLEEAEAAVRSKDVELSTALAALDRTA-<br>SEMCDAEQRLQVYSEAKEQLAAEHARTVEELARATRAVAELEARAAGAEGKREV-<br>MHTAAEVAEAELMARVAALTTENAQLKEKLSISRAAVTTFASRQTDGEGAAASLEE-<br>RCAAEARRSEAAEAEASALRGTLDRTAAELAEVLQRSEDVQATLERTVARLAEQES-<br>LVGALSAESTRLQDELATAQRSVLLLTGERDALGEEAAGLRGELSAAEDRASDVAA-<br>AQQRLQVRLEEAEAAVRSKDVELSTALAALDRTASEMCDAEQRLQVYSEAKEQLA-<br>AEHARTVEELARATRAVAELEARAAGAEGKREVMHTAAEVAEAELMARVAALTTEN-<br>AQLKEKLSISRAAVTTFASRQTDGEGAAASLEERCAAEARRSEAAEAEASALRGTL-<br>DRTAAELAEVLQRSEDVQATLERTVARLAEEEELVEQLNYDASELSDARAALTEEV-<br>TRLQKALAAAVTDKADVLAQLLATQKELDRAAEVQDAQYRDEELLQNALATTTSAF-<br>DVQTKQVRLGMAHVVDMSRAFCRVAEVAVQRSAAGVDGAASTGANVQEILSPWA-<br>RALEWVAAMEQRLETLDMGAMSNSLSRTDGRRERRRRRRGRTTELEGDNAVEM-<br>EANSGSHALSSSSLCRVVSINCAEAAFARLMDEQAAATNTIADLRRTLVDKEVDLTN-<br>LRSAMDALASDLISERDRADNLSAAIDDASEKIASAQAEFEEQLRQRTAATTAEVVE-<br>ARRAEARATAAQLRAEEQLAVVEKDLKEQEGVLRKLQDENHRLTREADRLLRTSRL-<br>EPSSRVRSNVSGSVTPKSLSLSAASAEDAVQRFTTAPSTLEAVEQAQILQVSSLQA-<br>DLLLSRRQARELEGREATLRQACAALEQQVSELRVEAAEAEHLREDMATLRADYA-<br>ELEQRYSELERMAIAAGGGTMQELKQLRQQLKVREVELEELKARMRDLVLSKAAP-<br>ELEAARRQEALRESLSGITTQLAATQAQYGGGTGGSSTVRSRRASLTAAVSNSDLL-<br>SLSSSRPSPQDVLTSRIEHLQTLVNDRDVALEKVQAAQLESRAAMDTLEDQLAAKT-<br>AALERAEGLVAEYADSVNNAFTMAAVVGRGNRGAGPTTVPPLSSEMGSPDAAALS-<br>ASVPPLTPLTPRTRAAVNGQPHAAVAMASPAPRMPNLDEVADDSDGSGEGKQAG-<br>SAGPSTDSAPPPVTHRRTRASGSWRDGKSCPAHRPSSSTERSAPSSPSNVPGRK-<br>SFASASVPATTRKRARSRTNT |

| | | | |
|---|---|---|---|
| Leishmania turanica | Trypano-somatida | NCBI\| compound of: ATBU01001269 and ATBU01001271 | MRRSEPGDAHTPHGQNRGEEEDEKGGELQRSISIGIESD-<br>SIHRAHGLRMWGNSFHNAGTHQQSSLCCDGQPAIMPGSRTSLPSPSHAAQ-<br>PARPPPAGVLSADLARPISVRELAQSRVPAVPPPPLATQT-<br>PQPRATTAGFAGFGVPTSSAVYAATSGVSTSRPLSAHADAGDLTSLAYRDIDHLS-<br>GRELYNYASHLRESSQQYGGYLSEAYAKRDQYRGEVARLKEELHNRYMEIDVLR-<br>REQERARDALLKLQEDNAQLRQKLAEAEGHARNMQAKISVLSGADPSLAVHFYQ-<br>SQLALKDAQLRELQQERERAAAASSQRRSSSTTAAEHGRRSANISGEGAAAPHT-<br>SPSCNLPSAEELQDGTEAASAALHTALEELQCLNTSLSHRLEDEHTAHKAALAAH-<br>AEASATATADRAELLDTIAQLRQQCADMQNVEDELVAALTLRAPISKKDYAALQAS-<br>YDDLTAALAKAEAQVAALQEKEQQHLHSAQQAQEELRRSLTTGLEERQSMQVR-<br>NEQMQSFVNHLERELKLAERANQLRQEQLYAAISGNQQLAESLHVAQEQLLSATS-<br>ELQDLRNTRESLESQLRELHQRIEELEQGDEHQRHQHGDADAESASSQQRQEL-<br>EVELARLRSQRDPLVGDGDDIVSDVRRLPDERRALLTSPPAHAEEQLQSRSSAV-<br>RAPLDVRADDGEWQKQEDIAALEAAHRCIEQMKQTIAALQSERVSVEADLKAKV-<br>NTLEAELASQSRALAEAEAAAAVQQAHLEEVVMALRDELVATQAQEKLLRASEEA-<br>HRERCAEQQRAVEQMQARVEELETAARNAASSLSTPPPAQPATAAESELIAMNA-<br>ALNSLAYQLEDAQHRVEELSAEHARTVEELARATRAVAELEARAAGAEGKREVM-<br>HTAAEVTEAELMARVAALTTENAQLKEKLSTSRAAVTTFXXXXXXXXXXXXXXX-<br>XXAEVTEAELMARVAALTTENAQLKEKLSTSRAAVTTFASRQTDGEGAAASLEER-<br>CAAEARRSEAAEAEASALRGTLNRTAAELAEVLQRSEDVQATLERTVARLAEEEA-<br>LVEQLNYDASELNDARTALTEEVTRLQKALAAAVTDKADVLAQLLATQKELDRAA-<br>EVQDAQYRDEERLQDALATTTSAFDVQTKQVRLGMAHVVDMSRAFCRVAEVAV-<br>QQSAAQVDGAASTGASVQEMLSPWTRALEWVAAMEQRLETLDMGAMSNSFSR-<br>TDGRRERRRCRRGRTTELEDDNAVEMEANSGSQALSSSSLCRAVSANRAEAAF-<br>ARLMDEQAAATNTIADLRRTLADKEVDLTNLRSAMDALANDLISERDRADNLSAAI-<br>DDASEKIASAQAEFEEHLRQRTAATTAEVVEARRAEARATAAQLRAEEQLAAVEK-<br>DLKEQEGVLRKLQDENHRLTREADRLLRASRLESSSRARSNVSGSVTPKSLSLS-<br>AASAEDAAQRFTTAPSTLEAVEQAQILQVSSLQADLLLSRRQARELEGREATLRQ-<br>ACAALEQQVSELRVEAAEAEHLREDLATLRADYAELEQRCSELERMAVAAGGGT-<br>MQELKQRRQQLKAREVELEELKARMRDLVLSKAAPELEAARRQEALRESLSGIT-<br>TQLAATQAQYGGGTGGSSKVRSRRASLTAAVSNSDLLSLSSSRPSPQDVLTSRIE-<br>HLQTLVNDREVALEKVQAAQLESRAAMDTLEDQLAAKTAALERAEGLVAEYADSV-<br>NTAFTMAAVVRRGNRGAGPTTVPPLSSGMGSPDAEALSASVPPLTPLTPRTRAA-<br>VNGQPDAAVAMASPAPRMPNLDEVADDSDGSGEGKQAGSAGPSTDSAPPPVT-<br>HRRTRASGSWRDAKSCPAHRPSSSTERSAPSSPSNVPGRKSFASASVP-<br>ATTRKRARSRTNT |
| Leishmania gerbilli | Trypano-somatida | NCBI\|ATBK01000608 | MRRSEPGDAHTPHGQNRGEEEDEKGGELQRSISIGIESDSIHRAHGLCMWGN-<br>NFHNAGTHQQSSLCCDGQPAIMPDSRTSLPSPSHAAQPARPPPAGVLSAD-<br>LARPISVRELAQSRVPAVPPPPPATQTPRPHATTAGFAGFGVPTSSAVYAATSGVST-<br>SQPLSAHADAGDLTSLAHRDIDHLSGRELYNYASHLRESSQQYGGYLSE-<br>AYAKRDQYRGEVARLKEELHNRYMEIDVLRREQERARDALLKVRDDNAQ-<br>LRQKLAEAEGHARNMQAKISVLSGADPSLAVHFYQSQLALKDAQLRELQ-<br>QERERAAAASSQRRSSSTTAAEHGRRSANISGEGAAAPPTSPSCDLPSA-<br>EELQDGTEAASAALHTALEELQCLNTSLSHRLEDEHTAHKAALAALAEASA-<br>TATADRAELLDTIAQLRQQCVDMQSVEDELVAALTLRAPISKKDYVALQAS-<br>YDDLTAALAKAETHVAALQENEQKHLHSAQQAQEELRHSFTTALEERQS-<br>MQARHEQMQSLVNHLERELKLAERANQLRQEQLYAAISGNQQLAESLHV-<br>AQEQLLSATSELQDLRNTRESLESQLRELRQRIEELEQGDGHQRHQHGD-<br>ADAESESSQQRQELEVELARLRSQRDALVGDRDDIVGDVRRLPDERRTL-<br>LTSPPAHAEEQLQSRSSAVRAPLDVRADDGEWQKQEDIAALEAAHRCIEQ-<br>MKQTIAALQSERVRVEADLKAKVNTLEAELASQSRALAEAEAAAAVRQAH-<br>LEEVVMALRDELVATQAQEKLLRASEEAHRERCAEQQRAVEQMQAHVEE-<br>LETAARNAASSLSTPPPAQPAAAADSELIAMNAALNSLAYQLEDAQHRVE-<br>ELSAEHARTVEELARATRAVAELEARAAGAEGKREVMHTAVGVAEAELMA-<br>RVAALTTENAQLKEKLSTSRAAVTTFASRQTDGEGAAASLEERCAAEARR-<br>SEAAEAEASALRGTLNRTAAELAEVLQRSEDVQATLERTVARLAEQESLV-<br>GVLSAESTRLQDELATAQRSVLLLTSERDALGEEAALLRGKLSAAEDRAS-<br>DAAAAQQRLQVRLEEAEAAVRSKDVELSTAFAALDRTASEVCDAEQRLQ-<br>VYSEAKEQLAAEHARTVEELARATRAVAELEARAAGAEGKREVMHTAVG-<br>VAEAELMARVATLTTENAQLKEKLSTSRAAVTTFASRQTDGEGAAASLEE-<br>RCAAEARRSEAAEAEASALRGTLNRTAAELAEVLQRSEDVQATLERTVAR-<br>LAEEEALVEQLNCDASELNDARAALTEEVTRLQKALEAAVTDKADVLAQL-<br>LATQKELDRAAEVQDAQYRDEERLQNALATTTSAFDVQTKQVRLGMAH-<br>VVDMSRAFCRVAEVAVQQPAVEVDGAASTGASVQEMLLPWTRALEWVA-<br>VMEQRLETLDMGAMSNSFSRTDGRRERRRRRRGRTTELEDDNAVEME-<br>ANSGSHALSSSLCRAVPANRAEAAFARLMDEQAAATNTIADLRRTLADKE-<br>VDLTNLRSAMDALANDLISERDRADNLSAAIDDASEKIASVQAEFEEQLRQ-<br>RTAATTAEVVEARRAEARATAAQLRAEEQLAAVEKDLKEQEGVLRKLQDE-<br>NHRLTREADRLLRTSRLEPSSRARSNVSGSVTPKSLSLSAASAEDAAQRF-<br>TTTPSNLEAVEQAQILQVCSLQADLLLSRRQARELEGREATLRQACAALE-<br>QQVSELRVEAAEAEHLREDLATLRADYAELEQRYSELERMAIAAGGGTM-<br>QELKQLRQQLKVREVELEELNARMRDLVLSKAAPELEAARRQEALRESLS-<br>GITTQLAATQAQYGGGTGGSSKVRSRRASLTAAVSNSDLLSLSSSRPSPQ-<br>DVLTSRIEHLQTLVNDREVALEKVQAAQLESRAAMDTLEDQLAAKTAALER-<br>AEGLVAEYADSVNTAFTMAAVVRRGNRGAGPTTVPPLSSGMGSPDAEAL-<br>SASVPPLTPLTPRTRAAVNGQPHAAVAMASPAPRMPNLDEVADDSDDSG-<br>EGKQAGSAGPSTDSAPPPVTHRQTRASGSWRDAKSCPAHRPSSSTER-<br>SAPSSPSNVPGRKSFASASVPATTRKRARSHTNT |
| Leishmania arabica | Trypano-somatida | NCBI\| compound of: ATBH01000268 and ATBH01000267 | MWGNNFHNAGTHQQSSLCCDGQPAIMSGSRISLPSPSHAAQPARPPPAGVL-<br>SADLVRPISVRELAQLQVSAVPPPPPATQTPQPRATTAGFAGFGVPTSSAVYAATS-<br>GVSTSQPLSAHADAGDLTSLAYRDIDRLSGRELYNYASHLRESSQQYG-<br>GYLSEAYAKRDQYRGEVARLKEELHNRYMEIDVLRREQERARDSLLKVREDNAQL-<br>RQKLAEAEGHARNMQAKISVLSGADPSLAVHFYQSQLALKDAQLRELQQERERAA-<br>AASSQRRSFSTTAAEHGRRSANISGEGAAAPHTSPSCDLPSAEELQDGTEAASAA-<br>LHTALEELQCLNTSLSHRLEDEHTAHKAALAAHAEASATATADRAELLDTIAQLRQQ-<br>CADMQNVEDELVAALTLRAPISKKDYAALQASYDDLTAAHAKAEAQVAALQEKEQQ-<br>HLHSAQQAQEELRRSFTTGLEERQSMQVRNEQMQSFVNHLERELKLAERANQLR-<br>QEQLYAAISGNQQLAESLHVAQEQLLSATSELQDLRNTRESLESQLRELQQRIKEL-<br>EQGDEHQRHQHGDADAESELSRQRQELEVELARLRSQRDALVGDSDDIVGDLRR-<br>LPDERRTLLTSLPAHAEEQLQSRSSAVRAPLDVHADDGEWQKQEDIAALEAAHRCI-<br>EQMKQTITALQSERVRIEADLKAKVNTLEAELASQSRALAEAEAAAAVRQANLEDV-<br>VMALRDELVETHAQEKLLRASEEAHRERCAEQQRAVEQMQAHVEELETAARNTAS-<br>SLSTPPPAQPATAAESELIAMNAALDSLAYQLEDAQHRVEELSAEHARTVEELARAT-<br>RAVAELEARAAGAEGKREVMHTAAEVAEAELMARVAALTTENAQLKXXXXXXXXX-<br>XXXXXXXXXXXXXXXXXGALNRTAAELAEVLQRSEDVQATLERTVARLAEEEALV-<br>EQLNYDASELNDARAALTEEVTRLQEALASAVTDKADVLAQLLATQKELDRAAEVQ-<br>DAQYRDEERLQDALATTTSAFDVQTKQVRLGMAHVVDMSKAFCSVAEVAVQQSA-<br>AQVDGAASTGASVQEILLPWTRALEWVAAMEQRLEMLDIGAMSNSFSRTDGRRE-<br>RRRRRRGRTTELEDDSAVEMEANSGSHALSSSSLCRAVSANRAEAAFARLMDEQ-<br>AAATNTIAELRRTLADKEVDLTNLRSAMDALANDLISERDRADNLSAAIDDASEKIAS-<br>AQAEFEEQLRQRTAATTAEVVEARRAEARATAAQLRAEEQLAAVEKDLKEQEGVL-<br>RKLQDENHRLTREADRLLCTSRLEPCSRARSNVSGSVTPKSLSLSAASAEDAAQH-<br>FTTAPSTLEAVEQAQILQVSSLQADLLLSRRQARELEGREATLRQACAALEQQVSE-<br>LRVEAAEGEHLREDLATLRADYAELEQRYSELERMAIAAGGGTMQELKQLRQQLK-<br>VREVELEELKARMRDLVLSKAAPELEAARRQEALRESLSGITTQLAATQAQYGGGT-<br>GGSSKVRSRRASLTAAVSNSDLLSLSSSRPSPQDLLTSRIEHLQTLVNDREVALEKA-<br>QASQLEYRAAMDTLEDQLAAKTAALERAEGLVAEYADSVNTAFTMAAVVRRGSCG-<br>AGPTTVPPLSSGTGSPDAEALSASVPPLTPLTPRTRAAVNGQPHAAVAMGSPAPR-<br>MPNLDEVADDSDGSGEGKQAGSAGPSTDSAPPPVTHRRTRASGSWRDAKSCLA-<br>HRPSSSTKRSAPSSPSNVPGRKSFANASVPATTRKRARSRTNT |
| MSC domain-containing proteins: | | | |

| | | | |
|---|---|---|---|
| Perkinsus marinus | Alveolata | Uniprot\| tr\| C5KFV5 | MSSSPGNYAAMTIAELRSILTRNGVKVPQSSIKKAVLHVFEGVGSGRFKKRIFYGE-<br>SATTNAVELWRKESRRGSSKGDRGSRVSKSSRVGRESTSRPT-<br>TNSRGSAVTRRSSRARTSISSAVDSEGTDLVDVARMFSSDEEEMSDSMGYLPG-<br>PRPIYCDDITKEDCEPCPPNAECRGRSMVCKPLFKRDGDVCVRDERL |
| Tetrahy-mena ther-mophila | Alveolata | NCBI\| EAR90926 | MNSDQQNRYRGGGSNISVSSMRKELIKNNIDIDKDADLASLYQQFLQEKGVYQPN-<br>QFSTPSEQNRENGQRQSHNLPPPKERQQSIQGILNMIDHSKEKKN-<br>NQNYVFVDQHNSDVTQGQSSNQMPNRNDIASNQNMQIPEEAEINQSSARQSR-<br>FQAFNFGQQNQSGRNSLQNNRASSASMNSNRLSGNQNGLGLQNQQQRI-<br>SNEGGNRSQQEIQSYSPVNYGNFNFRMSNQIYSPGINNGIRSSSNGMMNN-<br>QQGYFINENSSKRVSNQSYNNFNQIESANRRSQQLNSPVPQTGKKNSLWKN-<br>GEEINDFAGSAVFNRHQYTPNYPKSTSITDTKWKGLLDTARELSQKKQRQS-<br>NDSMMNISQYNIFSNKKSQVESRKSENIAEVDTSFQYLSQQQKLNIKDKVLIFF-<br>SKIEISHIVMFIMVGLFCIFFLQNIYDFLTHTPELEYCDSTLRQEQIEGSSCIPCP-<br>QNAICLNNNIKCKHSFVLIDKKCVTNPQIEPNTIRYLSTVVQNLQYLQAQYHC-<br>YDSNSSGYGNNLSRPYKEHKWESIVNILTIHHSKDLLFYDTLTKVKDYLKSKS-<br>IREQFDINYDSLRDTYYVTTFNPSYYCRIRLWSIQNVNIIIAVLCIFFLIFLLYQVA-<br>AVRRFIYFFKLYRIQQKIVNENKIQQQNNSLQQDLNKSIKDNQQLYQQGGQV-<br>SQRFASPQNSNEQSFSQRYSQQQNPSQQQQIKLLSQQQFLQVPQNQQNYL-<br>QIPSNQQQQQQQLKLDNSLLNLSQRRGKLS |
| Tetrahy-mena ther-mophila | Alveolata | NCBI\| XP_001007953 | MSKPVNFLDLIKNPDKIQNKQPIKGSNSNTTTPKEAVIPPNLAGAQKNEWLE-<br>QQQKKNSQNANKKDDDVQILDYNPFKNNQEAKDSLKKQIQKPNIQQAAPP-<br>KNISEGNKINNTYQIISKPNQLQQANSPQQQINKNNVVNIGGDQLVFRQTQNNKQD-<br>KNAIQPTEISKKNSIQFNSDSSNSSKNNNIINQNNKANQQISSNTNTHNTNNKIV-<br>SNNQRVQSNKSVGSIFSSNKSNTSNKSAGMSKQEMLKAKYSLVNQSKEDYKKIF-<br>SKIVKEYSDTENLTYNPSKINYSLYFIYFCVFVFFVMVSLDYVASNTPKFCKNEVK-<br>YDTPKCIVCPENAQCKNYVMKCNDGFIQEGYKCKLNPNLISSSESFIIEKITQIQY-<br>DNGHRMCFAQESDIDQKQKEEEEVIKISRAQLLEELQQRLADKKAVAQQKKILEK-<br>FLVSTENQEKLGFKYDQESQTLTSSRLRFTIFCKCKQLIKNNQKSIAISSLTLVSLIIV-<br>GIILKKHFKKIRIANEIYEELAHQIQETSDKQINVSYYKEGIVNRFKSDFNEDVWIL-<br>VDRKRVNEQKINVYRDCDGLYWSQI |
| Toxo-plas-ma gondii | Alveolata | NCBI\| XP_002365595 | MLLVIFRRSCSRVQRDTTLSALPEQPASLASLSPRVRRLASSPPSSSASSSSSS-<br>APSSSSAPSSSASSSSSAAPSSSRCSSSPMQRRVLTSDGGGGNTAGALASAR-<br>RRAGRTEASKTGGDEQLDDEASRVHTAGEQPQEQRRRARCRVGSKGGR-<br>RRGNSLRRGARRHSEERHCMPPSPSFASSSLSPAEERRQAATPSSLPLASSPLSS-<br>CPLFLRPLRVSLLISLLFALGLGVLLFLATPLTLRNGARDSQQLFLPYCNKTRNLPLA-<br>LVDNSPAPPPPALAARSRDCVPCPAHAVCLNGEMRCVPPYIQASTRSAALESLLAR-<br>FGVRATFLRSFFVLSGLSRGEDASSCVEDEAAKETAKRVTDAVVLFLRSKAGKAQC-<br>ASFAPLGSQQSQQSPRPASAPPCEEGVESGRGARHTVGCPDKSVFQGRDRDATF-<br>ARRTEKMFAETRGTEDIEEHSGRSSLSGRTSKSSGALSGERSARESRPSESATRW-<br>EIIQLAQVKDLLEDDNVYAYVFNHFLDEETAERNFSVRVEQTYSFQSLDLVVPTEKS-<br>AAARHEQTEALLPLFLREQRRSRGITYTYTYTGLHALRPWSCTLYIAVMYRLAPAVL-<br>ALLLLLLLSLPLAFWLRRVYLRHKLREVLLSRERAEASELFTLQRNALATAIFPGTSS-<br>KELSHLLLRSLVTLRTGRFLRWWLQRLARNTAEVDTLCHDLLVDKYIRLHTARIGEQ-<br>NFWWAETESPPESAQPKHPSNPVSPPGASGANVAGSWDAHSQFSSAGPPHEGV-<br>VTGCGPQELDFQQSARPTLLEMCGSNGPTNWCSYSLRGDGMCGETDMFASGG-<br>NRTVADGWLGERVRRSSSYLSACRLADSLHAETSLGQSGKSEMRRPTLGGSADA-<br>SWREQRGSYSQTAGDTLRQRATLLGGGIDQRLGVGLLRTEDTSIEPRTTSGLKALA-<br>TLMGGDQRVSLDPGSRNVRRLQGSLVSSEGQESGTVSSRQSSVKERAARDYSH-<br>AAFFSRASRRREGSRGATEQTDGYTAERGRDDLSFMSGEVADNHTINGDYKGTG-<br>PYGQTHLSSRARRRSERRRSSGMRRVDDDVEDHETGHSVLC |
| Ectocarpus siliculosus | Stramenopiles | Uniprot\| tr\| D7G5B7 | MARPSKRKPAGAAGSTSPPSSKKPRTPAKPAKTNKAEDRKSGRKNAAK-<br>WAEVTLKKKRTSGEGTSSEDEDDNVSDAMETSDLNDDTPGPSVRKPPAKRPAP-<br>SRNGRTRKPSLPPPMSETISETRAPPSTSWPESGSGSGSGRRPAQSMSNGSP-<br>PAPPPGTPTATPTKRATGRRLEGKTYTPSRKSSFLRHANGTEGTDSATAASASD-<br>GPSLNSDNLGGPKTRGQARRGPSFLDRERAGRDTVATAGAEGRTPDMKGTR-<br>GQKDKSKSKVDSVPQPAQATDAGADADGDDARPRRGKSLEWTFKCAVVVVLA-<br>PVVVLLLVSGLLVAWPHVQVAAGGAWGAFRGLTAKTPSVEKCFLQPGEQEDIV-<br>CKSGTEAMDCPDHATCGAGRILHCNLPYTLRKDRAACVLVAEAEKEAKAVVEAL-<br>RTLTAEELCKGKGLIMPRASARGSFITLTDAATAQSRGPLSLLAGMPPGNVVGLI-<br>ELEGEPAQVFGGGQDGAEGCAGSGMERVARLSESAHDAVMRTLGWRCKARL-<br>AAGSWFMTYWQYCLAMYCLVVGAVCLDLRRRNSYYRHVQVCTIRDLCYQTLL-<br>DHASKDSSPMQVSHIQSSVTDWTRQRVAKNRATIQRHIAGLWPLVEREVEHDE-<br>RIRRCYKSLAGKRSVACWKWVGPTGTRFETIGALNPGRAPRLLLLWCQERA-<br>RPMPWCQLCWLLQA |
| Phaeo-dactylum tricornutum | Stramenopiles | Uniprot\| tr\| B7G9F2 | MPSTAASRKRKARAPESPETAPARRSRAERESQKQAAREWAAAQVSHTRPET-<br>SAADKNEEVLQSPPKKRRLTPVVSATTSTNRKAPPSEVSKKAPAASQASETLST-<br>SRAQAPTTPPPVKLSAVEQRARAARYMQQQSSQSPKPPPNVSSETSRAPAATP-<br>PRPRTTTKAASTHVTKPVFGSPVPTSRQRHGDAARNRRRSE-<br>ETHVDSLPLDEVVKTTLHTRSQANEDEISLPSDDNIDEESFL-<br>DSDEEDEDYEDDDDEAEQEELAQARARERRLQVANDASRR-<br>GWCGSLLGVLLVIVSSMALASVFESGLSLETLAEWWSPIEPT-<br>QPCFLDTFPPGPEDDVPDLPPHCTANRQPCPDGGFCRDG-<br>HLQRCQSKYEQVSDHGDACILSADTNQTIANMVDLLRSWTI-<br>DYYCGLGDERAQDLHLREDPTTGRPLFHYSRVTGELEQGYD-<br>WNLVKTANAKDPQIFITTAVDADLFIGLHPDQPASRPLICVAK-<br>QWFVFGLGLMGTFVYSLGEFCLMFYYGRLRAAPAATVIVSVT-<br>GWLLVIFISRWQRRREAERQILLDVVTIRQAAFDRLSSAPGT-<br>ALSALQLRDQLMWEHFQTSRQGRERLVKRIWPQIVHDVRQ-<br>DNRVRKSLGVRDGKQDLVWTWIATTTKSPATTEDRRVAPTAVRFG |
| Phytoph-tho-ra par-asitica | Stramenopiles | NCBI\| ET-P40127 | MEHSTTHIKRKPPSSTASSSNKRRRHRPPASPTPAPSAPLSRPKPKTRRLVT-<br>SNKVAESKPKEKRRKVEQLTLFSRYADPLVQSPKPQINHCSTGATILETP-<br>PVRHTEPGSVNGGTVDLATVPDMTKLEIVEGDEILETMLRASTPRVKRKF-<br>PSEEALTDSEQKDREILRRRRVKLRSQYSAMKDQQVRDVGFTVPTQ-<br>SLNIERNLFQSEDKTEQIKAEEVERAQLEEEEAETDEETTESKINRLS-<br>KAIHLNWRQFCLWLLSGAFLLCVVVVTAPFVKKLLEPSLPYCDSEWI-<br>EANEGSFVLADPADYFDRSKALQPFIESTDISTQTSRPLCQPCPVYG-<br>NCLNGSVISCAPPYLLHSGVCKENPEIQENLDQLALTIQKFVVDKAA-<br>KNVCDNVSLWSYLNPGSKAESTRDLASPIEVLLFDVQAFVTRTISFG-<br>KAVAMLPREYVFNRALDMALRDLKDIVVTEDQRQLIVGGSVVPWSCR-<br>AKLQLYSHFKLIALAVALGTALVFSYRQFLLYRTERQLVDRFVKEVRFF-<br>LLDRTRKRDRFYPSDHLRDDLFEKQSLQNRAWLCKSVWPKVVAVVN-<br>DESRIRTRVIKVRGQDLVVWEWVSSSSPTHRRPGGNR-<br>GGGFHAASRSQQVTGDRSRSTHVQQRKKVT |
| Naegleria gruberi | Heterolobosea | Uniprot\| tr\| D2V886 | MSTSNNRASLSNNDEDESPTRLTIKELKSILTEHDVELPAQDKKRDFYVDLYNKHIK-<br>SKKRKNDEVNDELSDDENEPEQKKVKSSTEEEPAESSTTTTTTTEEISQPTVEEPK-<br>VATPMAEVKAKVQRKTKSFAPSSLEAETPQVKVSDVRRRTVGNRDLQYRQR-<br>PARNEINQGLLQTPVSSNRAPPPTPLSPTDSTLVQDIRPSEIDQEQNTLGKVLTS-<br>ILMASIWALFFFCVYLFLFGQRDPHLPYGGRYCQSEDGLLQLKPRDNCLPCPE-<br>NGYCSDGRLVLCQKPYVIKNAVCVEDSRITLNALEFITELQNELSSKSGKFICGR-<br>AESNSTSLEDIKAKLTVKLAGSGVAVEDVLEQVTKLVKSSPQNFQMNIAPVEGS-<br>DKEMIFSLNPTLPFDCRIQKATQENKGVIITILTLAVAILIANFYKNKVEREQNDVED-<br>LFRISITRIREEEEIIVEQLRDELKEKYGHEIDVEKYWSRVEDLLASDSRLSQ-<br>IPSNSGTAYQWAVATDEQE |

| | | | |
|---|---|---|---|
| Giardia in-<br>testinalis | Metamo-<br>nada | Uniprot\| tr\|<br>A8BJR2 | MDPNDYLNTDPSRLRVIDLKRAITEFGGVPEERATKATLIKQFAQLRSNYLRTVSRG-<br>PRSRPQSESFDIGGFVDAIDEQLVAVNQNLNYSKVGRGDLTQSAFDFTGAFNDYAL-<br>HEQLVRENEVDAAKPVSPLKRMKNPYGVERTPGERKDSTASAPSTGSSKLDGK-<br>SQTEKSKTTLTVSPVDQQEIARSVLRKSSYIDKGRDKHSSSQMQVHAEMK-<br>HSSKNTTKKHKSSERHRSKSKEKRVESQKELCKSEGAGQFPVKTPTIRIEA-<br>PTNPYPHIGSSSIPRDASHTAYGRVQLERSVHSHRGSTVHVPDKNLTHKST-<br>KRRDTLLQETQPLYKGKQYEDEHNAYNFKTTIHSHPTLVRLSFIFVLLVVSYQ-<br>LFSLASDPVKPQIPVISHVISVLFACIFSGPVYTETLTHTSAQYGITEPCPTFG-<br>TCSGGLLTSCMPGYQRAPSNNSFYPQVSFVSARNFFSAIGNNAMARWMP-<br>GAYKHMSDAASRSSDINGDDSYSLSQIQYSYGFMTRAPVLRRRMRCQRA-<br>SGVTTKLSSLLQALDHELQAYKGSLFCRSRGRKITVDTSLPLLQTRTISRFLR-<br>RNSLIFSVVNQTSTNGILSADSLKDLVTTYTDILQNDVLEALTQAILHEGTDN-<br>EVYLPPNIKFRLPEEARNIRSVVQCDSDDSFQDCMHKQYIYILSNTNVFTHS-<br>LPRFSLECKFKFWFHRDPAAALGKILQCTVSIAGSLYIATKIGVYVIAGYIARR-<br>IKTYLHDYKVDMLQVAKPGDAKLPGFAAVTLVQLRDLFLASYPRVIRLILWKK-<br>VLKNIHSDSRITITSSIDLTGRIAEFAVWNGLLLSNSFGNDAPIAGRHFKNHVTPVS-<br>LPPPSEDNE |
| Trichomo-<br>nas vagi-<br>nalis | Parabasa-<br>lids | NCBI\|<br>XP_001299<br>627 | MNNDYLSAVPRGQLFDALKKLGVTPRYDAPQEELVNLIKQKIQGTDIQRDKELL-<br>GETFDHHLDSIVNKLFIALFVALSAIVLIIAFNYLKPSKKLFCDSENPTGLCIKCP-<br>SNAICSEGKAKCHEGFKLMHRRCIYDDDDSIYISSLYEYEIKLLEKQAGRFDCRLDK-<br>TKYITRNDLLIHLIRHSKFKNVDIEYLSNKAVDHLVYENTIGNVTEDNQELYYSTNMQ-<br>KPISCIVRQEIESHIFLTISISIILLSTAIYIYVQSVKNNRRKQSFLFVQSMINSYRNHHIP-<br>VNEDTIRSNLVSIDPNPDSLMPYVLEELRRNPKVKTSYICGSLTFRFDQK |
| Trichomo-<br>nas vagi-<br>nalis | Parabasa-<br>lids | NCBI\|<br>XP_001313<br>092 | MRTAGPLDDAFVPTQRRILGNYTREQLVDILDKYDEAFDINASKEKLIDLIIAIMKN-<br>TKPVSQPETKRSDIYLYLSWISKYIVLLFIAYFAFLAVSLLIFYLFVPKPQKFC-<br>NTNKQSIYCITCPKFAKCKSNNVTCETGFTFDGRYCVKTDDDTHVISKMLDFATTEL-<br>RKRAGKHRCGKCLSNYLTIDQLDALIYSQNFINPQKYDFVFQKMIQILQQQYNISTQ-<br>YDDRTTLIASTIADRPFSCILRRFLISSTFLFILICILCYLIKYSIKRTKNQKFIENKAQYM-<br>AIKVINEMKNHDSEIDENTLKHKFSKNPEIDLSLWLIIDSYLKKSPYVIARKS-<br>KGVTYYRFIL |
| Acantha-<br>moeba<br>castellanii | Amoebozoa | NCBI\|<br>XP_004337<br>553 | MSYQVPDPNSLTIPKLKSVLTQHGFGDQLPAHQEKKGFYVDLYRKHIVPLLEAR-<br>NAASQSLTPGPTSTTTAASAKERATKTDLVDRRRTIHSLAPEPTEAAPTKLTQTKL-<br>VAKSAVPTGPTTPAAKADAAPAAVMPRRLFAPEQQPTTVPSSSSSSSSSSSSSAPIL-<br>SPKASASRRTTIATGAVSYPKLGAPAPAATTAKATTSAGPDLRQIIALQHARKKSA-<br>GGSSAAMWLFGVLVLAVAVGAGLFMYGQLPSLLQAEKPYFCDTTGVSDNRQNP-<br>REPVVPCVDCPNFGVCKDGRLIKCMRGYEVSGGVHCARDKEIALAFEFCKEAAH-<br>KTLALRAGEHQCGYPVPALPMEEQELRDILKQKMKDAALFPWDEAKFNEAFLDL-<br>KKDLQRSTSHRNVIIRASNGNYTFDTTDVVVPYACVLQRVVLEHIWYIVGTLVALAA-<br>VLRFVRLQQTKAQREADIETLVESTKTLIKEEKKAGKPPSSVLHIREDQREKQTEL-<br>KKRYPTVKDWLAIWAVAENRVRADTRFKEYSQMVAGQQQQVWEFVGTVDAAMA-<br>EKILSPPSRRSYPH |
| Galdieria<br>sulphuraria | Plantae | NCBI\|<br>XP_005703<br>659 | MASTPDKEAQGSTSVEKRKSTSASKVVASGDRGATGKARLEFRKGFYNKDTKD-<br>SLDRSSKSTGSRARGKENIHSAHKEDEQVVSPPIISPKLDEVESKSPFPVSRNL-<br>GTEFLSVEGTPQPKSKAPEHFSGIKLLSITILAVSIFFAFTYYSPPIQLPFCD-<br>SNNSKVLEDCRPCPSHGTCSAGELTCDHGFIESAGKCVEDKTFSVVTKDTKNRIEA-<br>ILRKRAGMSLCGTGEVQKEMDSAQLRKAVLLSFGTKLPLEEIRFNRAFTEAFYTIVN-<br>DSVSSDEFECREEGERIWCQALHPSLSLSCRLRFFVLQEWWKLLITMSILIGISRLY-<br>FWFRLRRWIDKQVKTVTEEVYRQLTASSQLTSPLSERKKVVVTRLRDDLLHDQSLL-<br>KWKDVIWRKVVERVDSDSRVQKRREIVNDLPQLVWEWHDKIV |
| Cyanidio-<br>schyzon<br>merolae | Plantae | merolae.bi-<br>ol.s.u-toky-<br>o.ac.jp \|<br>Locus_CM-<br>P246C Lo-<br>ca-<br>tion_c16f00<br>01<br>614070>61<br>7420<br>CDS_#642 | MVHSPLPGSYKTFSTADEKRRVPNQRPIRKHRGWRIRALSRNAFRNLPASRL-<br>STPMTDWKQWKVRELKQYLLEHDPSAMPAQQRPTKAELVEAVEQVHQRLQR-<br>RQKTKQNPPRALPKEEAREPRRPGPRPRQSALGDERKLPTTRAPRTP-<br>KKAERQQQDSNRGVGKSEAPERPSSSESVTATSRKTRQKRSARKTEAVQGSSGV-<br>DGSSSGRKRVRRSPLRGEEARRRLVGDDIPEDRSPDGGAWAESQHPLLWSVRPR-<br>SEAQLSPASLIQDLRRRPFSANATSTRTSSPDRHRSASPRASSTWQDTTREFRPT-<br>KQGRSPSPTVSASNGAGPSTSSGLGLREALRDVLSMVKSHDQLEPMEYVEYPEQ-<br>VLPAEAESHARWHALTHQVSAVTPEAGDGASRSSLHSSPVAGRDVPWLRQPRNA-<br>AAQTKEASRITLLRDTCDPLAQSQHMPPREDVVALSTGSPGELTSGGTLAGTRSTP-<br>TRAYTVQQDEAASGFMTGSMEGTRLASLHEQVSAVLERIRAEQANLVSGPEAATS-<br>TALSRNADSLATDALHQRLGDSGAAAPAVASLENRSPGGSAAALTRGTLTVAPEPP-<br>FAQSPLQAFRTLRQNRERMPVSSAATLTGSLRGRPVHPLEQRSGDSAERSTSATT-<br>TDPTRSPGAFHASWSLPSTDAHSGDQPSISESQRYLARPETHAAVQETSAGTPGN-<br>ASRVSRRDPALLGNRALEHRHWLRSHWRVLVALCLLLWFAVRIYLVLPSIPFCDSSE-<br>SGSGPVLGCRPCPEHGICVGGVLTCRDGYVVLGSTCAPDRDLNRYAHIIQGQVHR-<br>LLGESAGRYRCGERAIRWKWTERELREALEQHPYMERLVRSSKWKAAFRRALDT-<br>LDDPVVKQPRSAHDTVHRSPRSVDDANDTVPAEAFQRGGAAERPLLADDSGTNS-<br>NSNSNISPNMASNESMQPDSDATQTGQLADDELLLYWSTDAQRPMWCRLWQLTE-<br>AHLGSLIWLGSITAVTLMFATTLRRKRLRQRQIRSLQQAVHGRLQEQKRSYLVALQR-<br>GRADVVTPFLIDVHLRDELLGDIANIYDRQRLWEGVSARARTDSRLQLRSETFTDG-<br>RRALVWEWTGPLGSAENAAPHLVLSGGTTSVE |
| Physco-<br>mitrella<br>patens | Plantae | Uniprot\| tr\|<br>A9RFH2 | MPLPQSSPVCVAKPSHYASPVKFETKYEPASTPTSSGKPDKDRRHKKKQHHSS-<br>GKKRSGTPLSLKEQIVQLIAILLTAAVVYNVTLTAIGWFEEARRPFCDDQQDPLRD-<br>NCRPCPENGICRGGELRCIPGFRRQGALCVPDKQIDRNAQNLIPEHQIW-<br>DASEEEKMGLDAENVLLVHEKALQLVRDKLNIRIDDFGLREFRCPIALALTYQ-<br>PIGCRIRKLIKANIYGIFIASFIVIFVSVYILRYLQSRRLVTRAEELYMQVCEVL-<br>EERSTGSSGETKWVVASRLRDHLLLPSERKVATLWKEVERLVQEDSRIDQ-<br>YPKLVNGDSRVVWEWQAATVSRIRICVAYGLRGS |
| Amborella<br>trichopoda | Plantae | Uniprot\| tr\|<br>W1P105 | MKRSRASSSSSSSISDLLPSTKEQFLQLLGVAAIAVSVAMSCNYFLNYSSLTITLPFCD-<br>NGADSSDFCEPCPSNGLCSEGKLSCIDGYRKHGRLCIEDGELNETAKRL-<br>SKMVELQACGSYAHFLCHGTGAIWFQEEEILRSVWESNLKVNYGIDSHILEH-<br>TIQKVREIVESSFDIKTSPDGMRELKCPDWLAEQYKPLPCILRIWFLNHIHYILPIS-<br>LLLVAFVRLLWRVHMHRKLSTRAEQIYEQVCEALEENAVMVKNSRGDCGAWV-<br>VASRLRDHLLLPNERKNASLWKKVGELVQEDSRIDRYPLLIKGETKIVWEWQ-<br>VGQDQLVRFGLRKLADPDIELDILWFDVGCKVPPVQDPQLDYCDDWFLFRG-<br>GLSTFKDENNTKSEQDETF |
| Arabidopsis<br>thaliana | Plantae | Uniprot\| tr\|<br>F4KHI7 | MDSIPRKRPKSETRTGRTPKSSSSSSSPIRSMLEPPQSLFPSKGEFFTLLKVLLVA-<br>CAVAFTCNFLSKSLSSNPSKSFCDSNFNPIDSDLDICEPCPINGECYQGKLQCNL-<br>GYKNQRNLCVEDGEINESTKKLVGYFERKVCESYAHNECYGTGTIWVPEND-<br>VWTELRSNSFLSNLDESAYNFLKGKAVEGVTELLEKRTNSNGIDELKCPESVAK-<br>SYKPLTCRLHQWILRHILIISSSCAMLVGSAMLRRRIQRKQCFSRRVEELYDQVC-<br>DFLEENAVASNSAETSNCEPWVIASWLRDYLLLPRERRDPLLWTKVEELIKEDSR-<br>IDRYEKLLKGEKKVVWEWQVEGSLSLSKLKKQRETQKKVRKSIDSSTSLQEY-<br>YNRRIAETSS |

| | | | |
|---|---|---|---|
| Homo sapi-<br>ens<br>(Man1) | Holozoa | Uniprot\| sp\|<br>Q9Y2U8 | MAAAAASAPQQLSDEELFSQLRRYGLSPGPVTESTRPVYLKKLKKLREEE-<br>QQQHRSGGRGNKTRNSNNNNTAAATVAAAGPAAAAAAGMGVRPVSGDLSYL-<br>RTPGGLCRISASGPESLLGGPGGASAAPAAGSKVLLGFSSDESDVEA-<br>SPRDQAGGGGRKDRASLQYRGLKAPPAPLAASEVTNSNSAERRKPHSWWGARR-<br>PAGPELQTPPGKDGAVEDEEGEGEDGEERDPETEEPLWASRTVNGSRLVPYSCR-<br>ENYSDSEEEDDDDVASSRQVLKDDSLSRHRPRRTHSKPLPPLTAKSAGGRLETSV-<br>QGGGGLAMNDRAAAAGSLDRSRNLEEAAAAEQGGGCDQVDSSPVPRYRVNAKK-<br>LTPLLPPPLTDMDSTLDSSTGSLLKTNNHIGGGAFSVDSPRIYSNSLPPSAAVAASS-<br>SLRINHANHTGSNHTYLKNTYNKPKLSEPEEELLQQFKREEVSPTGSFSAHYLSMF-<br>LLTAACLFFLILGLTYLGMRGTGVSEDGELSIENPFGETFGKIQESEKTLMMNTLYKL-<br>HDRLAQLAGDHECGSSSQRTLSVQEAAAYLKDLGPEYEGIFNTSLQWILENGKDVG-<br>IRCVGFGPEEELTNITDVQFLQSTRPLMSFWCRFRRAFVTVTHRLLLLCLGVVMVC-<br>VVLRYMKYRWTKEEEETRQMYDMVVKIIDVLRSHNEACQENKDLQPYMPIPHVRD-<br>SLIQPHDRKKMKKVWDRAVDFLAANESRVRTETRRIGGADFLVWRWIQPSASCDKI-<br>LVIPSKVWQGQAFHLDRRNSPPNSLTPCLKIRNMFDPVMEIGDQWHLAIQEAILEK-<br>CSDNDGIVHIAVDKNSREGCVYVKCLSPEYAGKAFKALHGSWFDGKLVTVKYLRL-<br>DRYHHRFPQALTSNTPLKPSNKHMNSMSHLRLRTGLTNSQGSS |
| Homo sapi-<br>ens<br>(LEMD2) | Holozoa | Uniprot\| sp\|<br>Q8NC56 | MAGLSDLELRRELQALGFQPGPITDTTRDVYRNKLRRLRGEARLRDEERL-<br>REEARPRGEERLREEARLREDAPLRARPAAASPRAEPWLSQPASGSAYATP-<br>GAYGDIRPSAASWVGSRGLAYPARPAQLRRRASVRGSSEEDEDARTP-<br>DRATQGPGLAARRWWAASPAPARLPSSLLGPDPRPGLRATRAGPAGAARARP-<br>EVGRRLERWLSRLLLWASLGLLLVFLGILWVKMGKPSAPQEAEDNMKLLPVDC-<br>ERKTDEFCQAKQKAALLELLHELYNFLAIQAGNFECGNPENLKSKCIPVMEAQEY-<br>IANVTSSSSAKFEAALTWILSSNKDVGIWLKGEDQSELVTTVDKVVCLESAHPR-<br>MGVGCRLSRALLTAVTNVLIFFWCLAFLWGLLILLKYRWRKLEEEEQAMYEMVKK-<br>IIDVVQDHYVDWEQDMERYPYVGILHVRDSLIPPQSRRRMKRVWDRAVEFLAS-<br>NESRIQTESHRVAGEDMLVWRWTKPSSFSDSER |
| Hydra vul-<br>garis | Holozoa | Uniprot\| tr\|<br>T2M302 | LFNVGPPSKDHDYRINMAEINVTELSNAELRKKLTEFGKSVGPITKSTRVTLEN-<br>KLKKLINESEQSISNSSNHKKKVVRKSNVSSRRSSLSRSESSNLKFAPFSSDEEDE-<br>VLFKLKKSKNSEKLLDLEKPKLVNKNELIEAKVQIPSTYTSASPISQDIISKSSPLS-<br>GKKYNSSNDYQSGCDLNRKNIAEYSDDEHFLEKLNEEKRQNIIRLSKKEIKSPSME-<br>NNVFRTPSSPVVISRTSMNAIDKNIRGEVDACKAEVRNSFTSKKPSPPTISRRVQLS-<br>SYNNYNAKNKVEGFKDREANKNEHSEKHFFLIKCLKSLFSEKQIKCIACILLFGLSAV-<br>LVGLYWYNEEDPLKLTYLKDSQGKEKDINIFIECLYKDLITRKGMVECGYSSGPDYL-<br>RREDFPQLAEGCLKNQKDIQFNVIANDTWEEAKKSYTEFFRFNETMVTSIGSYKPF-<br>HCRVGQALYAVLFRIFIILITIIFGMAIYFYMKRKWSAEDKETRQVLYFVHRIVEVVRK-<br>HDTQCLNNKELSPYLPIPHVRDMLIPLEKRKEMAKTWQKAVEFLSSSDSRIRVETQR-<br>ISGEDFEVWRWIGVRTPGIKEKKINSNFHLNSDQDKCWQGPAFDQIEKVIRLPIITPA-<br>PCLKIRCMHEGPDEREEDWVKKVENAVLEKCENDGARILHIHVDINSREGCVYVK-<br>CDTLESARKAFRSMYGNWFDGRLVIVKFVTLARYHQRFPDALKCVQALSPSGSNA-<br>VVNSNKDI |
| Capsaspo-<br>ra ow-<br>czarzaki | Holozoa | NCBI\|<br>XP_004364<br>881 | MPVSLRSSGSRSSASASAPAATSSVPAPASPTRRRWTKRLPSRSPGEGPVGW-<br>PSVTAPTTAASSHGGREDRYDDDDDDDDDDDNEQVQVVQRAPRSRT-<br>VAVPRRSADGVAKRARGSGAGVPGAHAALQQQGIVSDDDVAANSDDAANELQI-<br>GRIGGQIGRRAGRTATLPQIGWDFVKSLVQGKRQASSVHQNEDAASSRRAVRG-<br>SVHASKSAASSSSLSRWFSSTAVLKAFTALCLLLSVMVVVSHHQQMPYCPTRST-<br>AVASTKQQCKPCPEHAVCLGGPTFTCNDPYQQGWRGCVLNTALEKLKDNIAHE-<br>IVVEAQRYRGQRECESSPSVTLSLAVPREAMRELVKARMETDAQFTTAWNFVC-<br>AHPEKYFGDAVEVRMDGFLAKAGIKPFECQLREELLYLLDCVAQAVFDFFSYVW-<br>QLLWLPLLLVLFFTLAVSYFKWQKSVKQNEQDALNTMVLAIMTLLREQAISQPFQ-<br>PAVSVSHLRDEIIDVSDRKRLAARWDEACKIVRRDSRVAQSVQSIHGIPQDMLS-<br>WVSPVVVASPMAAQRQASPLVDNPDNADNGGGDENDRQP-<br>AIRSPQAQHRPYASSIYPNIADL |
| Saccharo-<br>myces<br>cerevisiae<br>(Src1) | Fungi | Uniprot\| sp\|<br>Q03707 | MNSDLEYLEDGFDPNSMKVATLRRILVENNVDFPSNARK-<br>NALVGLFDEKVKPQIPQLRKMYLNVRPSDEGIVKMDRPSSSPSIASPRRSRRAR-<br>REKSASPMAKQFKKNRILDDVSNDDDDDDDDDDDNDKKDDPLIVPSGTDTDEVD-<br>DEEDDVITSSSNKSDTNDFQQNSDTRKKRKDPDSDDWSESNSKENKIDNKHLNL-<br>LSSDSEIEQDYQKAKKRKTSDLNQEHGNGSAILGKLSVKTPIKNTNRKPVSMDNF-<br>NDSLTSSGTENDPFVPNIRHNPKELGTANGTGHSTPLSKLKVSASFADKLPQKEV-<br>PSTILVPEVEQQEPSQSERTPSLFSSEGSGSESEAPLLPEITTPGPHQPMGNTSN-<br>NVVEMIDTDSSNLVSDEDEVLVPTRIETPQLPTEKDVEKCEARVQELQEEVNEQL-<br>EHENGSEFDVKQGSGKVGNRHKFKRALKFLSKSLLALFLFCIFIVIPLLFGLWYRE-<br>QRLLIGYCGHEVPSHRVSGNSFEFIQKLDNLLQDYRPKCIPCPPNGICYPYLKLKC-<br>KPDYKLAPSRLDFLEIIPAQGKCVKDDKKQQLVSEVVEKSLEFLRAKNAQISCGD-<br>GKDDIESGMTEDALYQIFNEARAPWIRDDEFEDLWIQVIKDLTEEPEILWRQLSPT-<br>DNNIGGNSNNIIKTNDVPRQKRHLPEKFISKTRNFRSTSKKYIGMKCRFEREIYQT-<br>YKKFQRPIWLMFLLIVISKVIEIKLKNYYRKKARIEELVTQTMEKLKFQKIKSMSDPK-<br>ENAYLSIVQLRDIFLSDIVDLKYKNQLWSEVVKYLEHNNSNIKSNLTEIRGEIMKCW-<br>EWIGPMELNEPKDSAENKI |
| Saccharo-<br>myces<br>cerevisiae<br>(Heh2) | Fungi | Uniprot\| sp\|<br>Q03281 | MDHRNLDPKTLKVSQLRRVLVENDVAFPANARKPVLVKLFEEKVRQRLQSS-<br>PEASKVRTSIQKVVKSGAKNADRKKTLKSKKLESSSSESKTVKDENVETNKRKRE-<br>QISTDNEAKMQIQEEKSPKKKRKKRSSKANKPPESPPQSKSDGKATSADLTSE-<br>LETVEELHKKDSSDDKPRVKELPKPELPNLKVSNEFLAQLNKELASAATENY-<br>DHSIKSTDLSSIRIETEEPVGPSTGAETRNESEVMENINLEVQPEVKEAKEEL-<br>TKISETFDNQDEEDTSRLSSKKNIRSPKGRTRHFIANKTKRGIDIMKPFIAHL-<br>FIWLWNGAIFLSIICPILFGLWYREQRIQVGYCGHEKPLKSLAISAFPQTERV-<br>DSVLQAYRPNCLECPEHGICSSFMNVECEPGYEPKSSILETYGIIPFPKYCA-<br>KDESKEKEVDELVWKVNEYLKKKNAQHECGEGENLFESGETETKLYDIFS-<br>HSRPSWESQREFNDHWKNVLEILKKKDDIIWLPLDFETNGKREKSKSNNTN-<br>YIYRSTSKKWVTLQCHLEGDIQEYITKYGGSLFITLGVLFLIKKIQSTLDNYVQ-<br>GEQIIEKLVKEAIDKLKDVKKNKGEEPFLTTVQLRATLLSDIPNIKEQNNLWAQ-<br>TKEKIMKEQSENIELYLLEENGEIMTCWEWKE |
| Schizo-<br>saccharo-<br>myces<br>pombe<br>(Heh1/<br>Lem2) | Fungi | Uniprot\| sp\|<br>Q10109 | MDNWEDPNFELRNLRVIDLKKILHESGVSFPVNARKIEYIRMVDRIRKNKLSSGPQH-<br>LLSHLQKEENSNTSKASSSEDEIAPKYLYPSSPSKSTKKPHNETEPLLSPQFIDKP-<br>SNIETPVKIESPHVSQNNTFQSYSELSPNVETSLTMKTPPAHASTPKFRSHK-<br>SHRVAVPMSFMDSSALHTSPAFSERLKLLSSSNNFSPQLRSP-<br>KISHRLQTSATSSPLQHKRPFTNVPERVSRDIEFAPLDSARP-<br>SESSSPYSEVDSAEEDDELFQNYVLQQTRKESKLWSFIKKVF-<br>HDIKYANYRLLHNLRAFPGISAISSSYLVHIFMILLGVVAAIFLA-<br>LLREKMFTAGFCDSGASGSSASILGISFPSLCRTCPPNAICPS-<br>PNYVECKPGYVLYEPWYSSLGFWPSKYCVSDTSREESVNIF-<br>REECLSVLRSWNAILHCSNNSSDLLERNMSYNAHPYVADNLN-<br>ISSDHISFPSKPFALGLLHDTLLERKSPTLGLEMFEDLFKASLA-<br>VLSETNEVVMDSKLICYDSWAGIPLRCRLKQQLIKFVWRNKVFL-<br>FGILALSGVIFKLINFFRTRSIVAKYLPSASRFCVESLKRQKANY-<br>QMSRSQEPVIPLIEMHDILFHGNGPLEQIHMTKATARTLWEAI-<br>VERVEQVGSVRTRESEVDGEWTRVWEWVGTNTLDFQTDRSFINTTSPLRE |

| | | | |
|---|---|---|---|
| Schizo-<br>saccharo-<br>myces<br>pombe<br>(Man1) | Fungi | Uniprot\| sp\|<br>O13712 | MEVPSYFDPDYDPSSLRVVDLRNILTEYQIYYPSTAKKAQLITLFSKLR-<br>RAKNGLISMTELQQKNVPPSSRSPRRRVAGVTNNVTARISSKRKINMVDE-<br>ANDTEISKTSQFEDNVMGMLQDENVQVLNTNTITISEESEFHASKIAKIDSRNEEI-<br>THIPFETQTELNAAVVNLDNSMESSFSIVQNLTNKDSSVDTATYDFSAEVGNIV-<br>TPASKFLDYDQSYLVNASVSGDPTPVKVLNTTSPKSENPLNQSSFLSFLGENL-<br>KPKFTSRSSSVYASPIKSSLNSLECNPSNLLSVRKNFQQSSDSYLKSNKSFDQ-<br>LNNLVGLSTGNSENFTPENNSFSWTHPKKNSSSPLPQSQSSSIFVEHLNQLYE-<br>ANASIHRPVNPAFSTNFGLEASNTSTPEKKKFDSQKPDDDSVNEISSDLGLSTT-<br>GIDRVEENISLTKDRQPKRPYFSLGSFISLIFSFTKVVNSLWLVLLVVPLLGFVG-<br>FWHQEVQRVGFCGVPAEPYPSSLYYLQPGVLRSSIESAYSFAHSLGIEASCQ-<br>PCPENAECGFNRQLFCKEGLKASFPLLADFGLKPYPRCIPNTVKVNKVEEMV-<br>QAFMSIIGKWYYKAPKEFATFESAKNLNGKSFVDNFKDRYYMYKQDIDNVVGL-<br>KDFKVYLKTTLNRLYNSKLTRKVLYYLFSPLFTLELWKLRVRGALSKFPTNCLR-<br>SVYSHTVSLMKYLTSAVISCWRIYLLIGILAAITGTVVWRIRVYAKKHVVKHGVS-<br>VCVSHCIAKLQKTKLKSLTDFSVNPRVEVVQLRSDCFVSGVADDKGLFELVHLP-<br>LSIQLEIWEKVVSVLEGMVSVKVWDSERLAKNRAWEWIGVFSDDIAL |
| Encepha-<br>litozoon<br>romaleae | Fungi | NCBI\|<br>XP_009265<br>646 | MPKDMVPEDYLNKDFDYTKLTKTQLRKIMYENGVEAIPPLTAKKSELI-<br>DAYKENIYKRIDVLMSRRKNISMENSFQNVSPKKKYVFEEVPDIPVTNSKGED-<br>PYKKPYSEASMSDIGTPRKDVKEIKESTSRNFSPKSSSFVEKAPSNQEKRNS-<br>FVEDEAPGGSSLVAGMSAANNSTIGFSGSSFSSSFIHSSSSGRSSISRRNEKQGSS-<br>SDVRKRAFTSDPTLDKPELQSEHSSPRSSSNFTEINATRFRSRSKKNKWFLVLSIIG-<br>FAVAGYFRFMCPYCLDGRRFCIPLPEHSKINNGRLACDKGFIIKHGILRDYCIEDNR-<br>HEKEMSKKIKELKKILERRSGDYMYGMIPTKSVPVKLLSSDPEIIERLKKEVGIVISN-<br>EMIYSVNARVSMKTFFRYYFRRMIMISTPLAIIIIMIKIIGMFRKKKKERLQAAQKIVKDI-<br>ADVLVRQIYVSTRNANFPSHVYIEQLKDCFGVDRKVWREAEKMIRRNSNIRESCIE-<br>GKSAWEWVGPILYKPEFNGSLF |
| Reticulo-<br>myxa filosa | Rhizaria | NCBI\|<br>ETO27229 | ESSPMPANLDTTPLSASKKETTATKIKEDDEVKEAAYKAPLITKIEDDEVK-<br>GATYKDPFSTSKVPSTQHWNEGDLSAMAFPSNPQNSVLPNKITRRASVSD-<br>KEGLGKKKVEQRRFSYSQAQESQKHQLLQDNMVFPKIDVFPYETNKTFDTLEN-<br>NEQIFTPKASQNAHSKVAVASMTPMLMDEGTDDHASARLMQSFS-<br>DELPVHKNQKRTDAIAATATVTDATASISVQVKKDETNLKSSSPRKT-<br>IGKICTVLWTIVRLFLFALVVVGLVVIAQFQWDRYFTELSDSNNSP-<br>RFVGDQLPECVESPLLYEGQPPESNGVRLCTDEELVVLLETKIHA-<br>AAQLMLSQKYGQYECGDETVRYKMEMTEIEDNVRDVLALSEAE-<br>KSRWKEAFEKFDAKVRENKISGVIQYDDNDKTCKSTKVVKPLGCI-<br>LSQWSKGHSVEIFATSTLLVIVSGFIFWYQKRKVNKKTPQGVNYICP-<br>LFCLLFIYTYTRMYIIVIVIVIVIVCLKWTDSGVVH |
| Reticulo-<br>myxa filosa | Rhizaria | ETN97409 | MNSSALLSDGFSKGNKSQSQKNKTNNGWCGRCMVPSISVMLVTVVLAIVVYNVN-<br>VPNYAIENWNKWLEGFLADEYTDSTLFCDTNRNTVFGQRDAEGNPLRC-<br>VACPVHALQCVNGIALCEKGYILEEYECVVDQVIVR-<br>MAQEIYAQVKDGFVLPFNIFFFFFFFPQRGNYNNQIAKKFLG ERKGKHVCGYD-<br>VESDMSEAELKRLVQMYVLI |
| LEM domain-containing proteins: | | | |
| Capsaspo-<br>ra ow-<br>czarzaki | Holozoa | Uniprot\| tr\|<br>E9C9D3 | MSAVSYNHLTDKEIHEELLRYGEDMVVGASTRKLAVSKLQKLQAAGASSPARSKSP-<br>SRSKSPSRTAAPKSPARAKSPSRSRSPSRAAAAAEESPKRSTAATRV-<br>GRALAAEADEIAAVPTPTRKSRTSASPSRSPARVSKTAAGRAKQSEEFEEE-<br>VIVEKIKPISPAKSRGTKTAAVDNASNNAAQIGYTLLAVFAFVLLLFVVFHFDGLQ-<br>CLRQWVESFKASGAMAIENASTKQ |
| Amphime-<br>don<br>queens-<br>landica | Holozoa | Uniprot\| tr\|<br>I1G4M5 | MEETYKSLYTTMLQEHEAVFAQQDLGQLRDFLAAIQNCNGSVFVHGCGRE-<br>GIAARAFAMRLTHLGKRTYWLWDDTTPGMHAGDLFILVNGSGAIGNIMYMYE-<br>QAKQTGAAVAMITGDPLRCPKDVQHTLFLPAMVYKGTCPDVVPSAQPMGNLFE-<br>QHLFLLFDVMAMMLETQMGLSHAEMESRHRNIE |
| Nemato-<br>stella<br>vectensis | Holozoa | Uniprot\| tr\|<br>A7RQP3 | MYRVVLAIKRKVEKPPTRPQPKLVIRKPVKGLPYDVHSLSDGDLARQLKSYGQVVG-<br>PITETTRPLYQKKLVKLLQEESKKPAGSQTPDKKPRPWEYSDNEEEEAKEEVM-<br>DAKPGNVTMENGDDHDEGDADDEIDAVPEPQLTKPYPRRTKKAVEKPKEEEEVKP-<br>SAPQCVKRETIIQETYMTTRKRTAAEKASSKAETVTAKTEVTPNVPKQAKSKSSFW-<br>WKVIIFLILIVSIALLVVYFMEGNPVKPSIAAGRSH |
| Hydra vul-<br>garis | Holozoa | Uniprot\| tr\|<br>T2M302 | LFNVGPPSKDHDYRINMAEINVTELSNAELRKKLTEFGKSVGPITKSTRVTLEN-<br>KLKKLINESEQSISNSSNHKKKVVRKSNVSSRRSSLSRSESSNLKFAPFSSDEEDE-<br>VLFKLKKSKNSEKLLDLEKPKLVNKNELIEAKVQIPSTYTSASPISQDIISKSSPLS-<br>GKKYNSSNDYQSGCDLNRKNIAEYSDDEHFLEKLNEEKRQNIIRLSKKEIKSPSME-<br>NNVFRTPSSPVVISRTSMNAIDKNIRGEVDACKAEVRNSFTSKKPSPPTISRRVQLS-<br>SYNNYNAKNKVEGFKDREANKNEHSEKHFFLIKCLKSLFSEKQIKCIACILLFGLSAV-<br>LVGLYWYNEEDPLKLTYLKDSQGKEKDINIFIECLYKDLITRKGMVECGYSSGPDYL-<br>RREDFPQLAEGCLKNQKDIQFNVIANDTWEEAKKSYTEFFRFNETMVTSIGSYKPF-<br>HCRVGQALYAVLFRIFIILITIIFGMAIYFYMKRKWSAEDKETRQVLYFVHRIVEVVRK-<br>HDTQCLNNKELSPYLPIPHVRDMLIPLEKRKEMAKTWQKAVEFLSSSDSRIRVETQR-<br>ISGEDFEVWRWIGVRTPGIKEKKINSNFHLNSDQDKCWQGPAFDQIEKVIRLPIITPA-<br>PCLKIRCMHEGPDEREEDWVKKVENAVLEKCENDGARILHIHVDINSREGCVYVK-<br>CDTLESARKAFRSMYGNWFDGRLVIVKFVTLARYHQRFPDALKCVQALSPSGSNA-<br>VVNSNKDI |
| Trichoplax<br>adhaerens | Holozoa | Uniprot\| tr\|<br>B3RIA0 | MKSDLAKDPSVLTKDRLKALLSTNGVPLPEGDQRKDVYVKLYMQHIQNKNIDTRN-<br>TEPNVRSEFSSDDDTQSVSKSAKKRKPAKKRKSTDTNNVFNIDSLSDEELLAN-<br>LKKYDSAAGPIIDSTRNVYKRKLKAFIQRSEATVKDGEFSDPDGVAEDST-<br>SKKVVKDTPTPRRGRTKSRRSVSRKEFVKSEVKDSTDSSDRKDDIAILNTSH-<br>SVKPSQSNVESPQSQDLQETNLWYFIIILVLALAIYTGVHILEIGRKH |
| Homo sapi-<br>ens<br>(Lap2β) | Holozoa | Uniprot\| sp\|<br>P42167 | MPEFLEDPSVLTKDKLKSELVANNVTLPAGEQRKDVYVQLYLQHLTARNRP-<br>PLPAGTNSKGPPDFSSDEEREPTPVLGSGAAAAGRSRAAVGRKATKKTD-<br>KPRQEDKDDLDVTELTNEDLLDQLVKYGVNPGPIVGTTRKLYEKKLLKLRE-<br>QGTESRSSTPLPTISSSAENTRQNGSNDSDRYSDNEEDSKIELKLEKR-<br>EPLKGRAKTPVTLKQRRVEHNQSYSQAGITETEWTSGSSKGGPLQAL-<br>TRESTRGSRRTPRKRVETSEHFRIDGPVISESTPIAETIMASSNESLVV-<br>NRVTGNFKHASPILPITEFSDIPRRAPKKPLTRAEVGEKTEERRVERDI-<br>LKEMFPYEASTPTGISASCRRPIKGAAGRPLELSDFRMEESFSSKYVP-<br>KYVPLADVKSEKTKKGRSIPVWIKILLFVVVAVFLFLVYQAMETNQVNPFSNFLHVD-<br>PRKSN |
| Homo sapi-<br>ens<br>(LEMD1) | Holozoa | Uniprot\| sp\|<br>Q68G75 | MVDVKCLSDCKLQNQLEKLGFSPGPILPSTRKLYEKKLVQLLVSPPCAPPVMNG-<br>PRELDGAQDSDDSEELNIILQGNIILSTEKSKKLKKWPEASTTKRKAVDTYCLDYKP-<br>SKGRRWAARAPSTRITYGTITKERDYCAEDQTIESWREEGFPVGLKLAVLGIFI-<br>IVVFVYLTVENKSLFG |

| | | | |
|---|---|---|---|
| Homo sapi-<br>ens<br>( LEMD2) | Holozoa | Uniprot\| sp\|<br>Q8NC56 | MAGLSDLELRRELQALGFQPGPITDTTRDVYRNKLRRLRGEARLRDEERL-<br>REEARPRGEERLREEARLREDAPLRARPAAASPRAEPWLSQPASGSAYATP-<br>GAYGDIRPSAASWVGSRGLAYPARPAQLRRRASVRGSSEEDEDARTP-<br>DRATQGPGLAARRWWAASPAPARLPSSLLGPDPRPGLRATRAGPAGAARARP-<br>EVGRRLERWLSRLLLWASLGLLLVFLGILWVKMGKPSAPQEAEDNMKLLPVDC-<br>ERKTDEFCQAKQKAALLELLHELYNFLAIQAGNFECGNPENLKSKCIPVMEAQEY-<br>IANVTSSSSAKFEAALTWILSSNKDVGIWLKGEDQSELVTTVDKVVCLESAHPR-<br>MGVGCRLSRALLTAVTNVLIFFWCLAFLWGLLILLKYRWRKLEEEEQAMYEMVKK-<br>IIDVVQDHYVDWEQDMERYPYVGILHVRDSLIPPQSRRRMKRVWDRAVEFLAS-<br>NESRIQTESHRVAGEDMLVWRWTKPSSFSDSER |
| Homo sapi-<br>ens<br>(Man1) | Holozoa | Uniprot\| sp\|<br>Q9Y2U8 | MAAAAASAPQQLSDEELFSQLRRYGLSPGPVTESTRPVYLKKLKKLREEE-<br>QQQHRSGGRGNKTRNSNNNNTAAATVAAAGPAAAAAAGMGVRPVSGDLSYL-<br>RTPGGLCRISASGPESLLGGPGGASAAPAAGSKVLLGFSSDESDVEA-<br>SPRDQAGGGGRKDRASLQYRGLKAPPAPLAASEVTNSNSAERRKPHSWWGARR-<br>PAGPELQTPPGKDGAVEDEEGEGEDGEERDPETEEPLWASRTVNGSRLVPYSCR-<br>ENYSDSEEEDDDDVASSRQVLKDDSLSRHRPRRTHSKPLPPLTAKSAGGRLETSV-<br>QGGGGLAMNDRAAAAGSLDRSRNLEEAAAAEQGGGCDQVDSSPVPRYRVNAKK-<br>LTPLLPPPLTDMDSTLDSSTGSLLKTNNHIGGGAFSVDSPRIYSNSLPPSAAVAASS-<br>SLRINHANHTGSNHTYLKNTYNKPKLSEPEEELLQQFKREEVSPTGSFSAHYLSMF-<br>LLTAACLFFLILGLTYLGMRGTGVSEDGELSIENPFGETFGKIQESEKTLMMNTLYKL-<br>HDRLAQLAGDHECGSSSQRTLSVQEAAAYLKDLGPEYEGIFNTSLQWILENGKDVG-<br>IRCVGFGPEEELTNITDVQFLQSTRPLMSFWCRFRRAFVTVTHRLLLLCLGVVMVC-<br>VVLRYMKYRWTKEEEETRQMYDMVVKIIDVLRSHNEACQENKDLQPYMPIPHVRD-<br>SLIQPHDRKKMKKVWDRAVDFLAANESRVRTETRRIGGADFLVWRWIQPSASCDKI-<br>LVIPSKVWQGQAFHLDRRNSPPNSLTPCLKIRNMFDPVMEIGDQWHLAIQEAILEK-<br>CSDNDGIVHIAVDKNSREGCVYVKCLSPEYAGKAFKALHGSWFDGKLVTVKYLRL-<br>DRYHHRFPQALTSNTPLKPSNKHMNSMSHLRLRTGLTNSQGSS |
| Homo sapi-<br>ens<br>(Emerin) | Holozoa | Uniprot\| sp\|<br>P50402 | MDNYADLSDTELTTLLRRYNIPHGPVVGSTRRLYEKKIFEYETQRRRLSPPSS-<br>SAASSYSFSDLNSTRGDADMYDLPKKEDALLYQSKGYNDDYYEESYFTTR-<br>TYGEPESAGPSRAVRQSVTSFPDADAFHHQVHDDDLLSSSEEECKDRERPMY-<br>GRDSAYQSITHYRPVSASRSSLDLSYYPTSSSTSFMSSSSSSSSWLTRRAIRPENR-<br>APGAGLGQDRQVPLWGQLLLFLVFVIVLFFIYHFMQAEEGNPF |
| BAF: | | | |
| Homo sapi-<br>ens | Holozoa | NCBI\|<br>NP_848572 | MDNMSPRLRAFLSEPIGEKDVCWVDGISHELAINLVTKGINKAYILL-<br>GQFLLMHKNEAEFQRWLICCFGATECEAQQTSHCLKEWCACFL |
| Salpin-goe-<br>ca rosetta | Holozoa | NCBI\|<br>XP_004995<br>959 | MAERTTSKKHDEFVRERMEDKAATELAGIGEKGGEQLAALGFATAKQVL-<br>GQFLVMGEDPDVFGDWLQQNVSKLNSKHRGDLIFCLQEWIKRNL |
| Capsaspo-<br>ra ow-<br>czarzaki | Holozoa | NCBI\|<br>XP_004343<br>428 | MAETTSQKHQNFIKEQMRDKPVNQVAGIGEKISGELNDQGFKYAYQLL-<br>GQFLILNKNEGDFLDWFKEVAPSANSKHRQDAYECLSKWCELML |
| Phyto-ph-<br>thora para-<br>sitica | Strameno-<br>piles | NCBI\| ET-<br>M47535 | MADGYDPQKSRVAEDTLADFLRAPLTGDLTEVPGIGKAAVAKLSEAEDGEDAVTNT-<br>FQLIGKFLMLKANSDDNDDGVIDCAAHCDAFWFWLKSKGITAYRSGIVMA-<br>IAEKVNTMLPGIYDAAEFQ |
| Thalassio-<br>sira pseu-<br>do-nana | Strameno-<br>piles | NCBI\|<br>XP_002294<br>632 | MMDFIRGSVTGDITEVPGIGPAAAKKLANVEGEEGITNTYQLIGKFLMLKGPDT-<br>DTNKVESFEHCEKFWYWLQAMGISAHRSAIVKAIAQKVNGLLPGVYDPDLYEDED-<br>DEEE |
| Ectocarpus<br>siliculosus | Strameno-<br>piles | NCBI\| CB-<br>J48376 | MAEVGYDPKRSKVSDDKLAEFIRSAVTGDLTEVPGIAARGAEILADGEGDDKVT-<br>NTYQLIGKFLALRGPDKTDHEVDSVEHCDKFWYWLQAKGINSNRSGIVNAIAEKVN-<br>TWIPGIYDADSYTDTA |
| Aplano-<br>chytrium<br>kergue-<br>lense | Strameno-<br>piles | jgi\| Aplke1\|<br>103957\|es-<br>tExt_fge-<br>nesh1_pg.C<br>_610016 | MATGFDVNRSKVSDDALAEFLSKPVEEDITTVPGIGPKAAEILAKNCENDPGIET-<br>TFQLIGKFLSLKGKGMGSKEHCDAMWFYLQAKGVNSFRAGIVHSIAERT-<br>NIMMPGIYESNED |
| Emiliania<br>huxleyi | Haptophyta | NCBI\|<br>XP_005762<br>802 | MLISMVPGLGDAGIRKLNVAGYATTYQLIGKFLPLNRNEEEFLAFLLKQGNTRPHVH-<br>NTAPLAL |
| Guillardia<br>theta | Cryptophyta | Uniprot\| tr\|<br>L1JLR7 | MPSCSRSQGLALCQVGRLVSCDGSSDTTAADKLKEANIDTAEKLMGNFMVM-<br>GRSEEKMAKWLEDVCEVRSVEAKKISAALVEKGDKICSM |
| Lamin-B receptor: | | | |

| | | | |
|---|---|---|---|
| Strongylo-<br>centrotus<br>purpuratus | Holozoa | NCBI\|<br>XP_786536 | MPSTTSFGDGDAVMSRWPGSTLWFKSKILRVSEDGYKVQFEDGTEEEIPLTDVK-<br>SESYFTRSRSRSRSRSPGRRRSRSPARKSSPARTQQSRKPRSPGRK-<br>PAAVKKEVVETPVSRTQEKRTESKLISRTQTVTKTEHHSYTTRAQTRS-<br>GKQQLELPVKMKAGKVAKTTHYEFGGPIGTFLMIFGLPLVVYFLYFTCLPSGCKLV-<br>YNPPFSLDWRDYYDQEAYLFYVGWFLFQAILALLPFGKVVQGQPLRSGQRLSYR-<br>TNGLFALIVTCATFGGMIYYKCPVTLIVDKILPLMTASALFSLLLSTLLYIKARCGPNS-<br>ALATGGNSGNFFYDFFMGHELNPRLGSLDLKFFCELRPGLFLWALINMACLTKVW-<br>TEFPDNPPWNLILVCVFQFLYVFDALLYESAILTTMDIIQDGFGFMLVFGDLTWVPF-<br>TYTLQARFLADHPPAFPDYCLIPVALLFSLGYFIFRMSNSEKNAYRQNPYGKNVAG-<br>LQTIPTDTGKRLLVSGWWGFVRKPNYLGDLLMALSWSLCTGFVSIVPYFYPIYFFV-<br>LLVHRERRDDASCRQKYGGAWTKYCATVKYRIIPYIY |
| Petromyzon<br>marinus | Holozoa | Uniprot\| tr\|<br>S4R4L3 | MVHSKFTIGEEVMARWPGSCMWYKVQVLSYSEESSIYN-<br>VKYEDGTELELETSSIKKIGSFRTPRKSRSRSRSRSRSKSPGRRSNSPGRRPRAR-<br>SPIRSPKVNDKEAKSSRATKPEEEPVQTTTNHTAVMQVPQAITKVKEISKEKN-<br>GAETGKQAAVVVKEKAMKEEKEMKKKKMGAVASESSPLVIYRLRSRQGIN-<br>QPKETPPVELPKLSSKPEEICAEPGLGVKLDPAAVCGSAGTALLPLALPLAL-<br>LYVLHVCCSDGCSLLSPPAPPPGLASLWDMCTLVLCLGWCLFQALLCHLPL-<br>GYVTERVPPNTEQRLKYRLNGIHALVLSISAFSLAKYKEAYVAYVHDKFLQLA-<br>VSAVLLAYLLSAYLFVRARCASHIAVATTRRPGNIFYDFFFGRELNPRIGNMD-<br>LKSFFELRPGFIGWAIISLVMVLEDLQKNEAFSPALLLYVALQFIYVADVLWNE-<br>ERSLSTGDIPHKYFGFLLAFTNLAWIPFASSLPAYYLVHHHQELTAYWAAAIC-<br>LLYLVGYVISRRAHTQKNAQSDSAGDRGKPADSRSSPVLGSWGRLRHPEL-<br>LGELLMLLAWSLPCGFSHPLPYIPVVYSLVMLLHREAR |
| Callorhyn-<br>chus milii | Holozoa | Uniprot\| tr\|<br>V9KH74 | MPAKHYEDGDMVMGRWPGSSLYYEVQVTGFDKISQLYGVKYKDGTELELKES-<br>DMKPLNYFKSKKSRSRSSSRQRGRSRSRSRSPARAPSSKRSTSR-<br>GRETKKEVEKQIVPEVKPAPQKLQENSNNSKTTLVAELNEEQENHTKT-<br>STYKITEHKIRSEIVTDELSLRYNLRTRKEKEEAKPIKLETKDKLLYIKKDVPQ-<br>TQELEFGGRIGAFFMVFLLPATVFILLLMCGQEDSSILSFPPQVPPLETLWAC-<br>TALGIFVLWFLFQALLYLLPIGKIVEGIPLPNGKTLKYRINAFYAFLLSAAAVGA-<br>ALYNGVNLSYIHANLLQVAISAMVFSLLLSVYLYLRSWRVPTDELAPGGNSG-<br>SFIYDFFIGRELNPRIRNFDLKYFCELRPGLMGWVIINLGMVFAEMNVQNL-<br>DMPSLAMILVNSFQLLYVLDALWNEEAILTTMDIVHDGFGYMLAFGDLVWVP-<br>FIYSLQAFYLVKHPSELTWPVAAAIIALNTVGYIIFRGANSQKNAFRRNPLDP-<br>KLAHLKTIPTATGKNLLVSGWWGFVRHPNYLGDLIMALAWSLPCGFNHVLPY-<br>FYVIYFTCLLIHREARDEHQCRKKYGVAWDKYCQQVRYRIFPYIY |
| Homo sapi-<br>ens | Holozoa | Uniprot\| sp\|<br>Q14739 | MPSRKFADGEVVRGRWPGSSLYYEVEILSHDSTSQLYTVKYKDGTELELKENDIK-<br>PLTSFRQRKGGSTSSSPSRRRGSRSRSRSRSP-<br>GRPPKSARRSASASHQADIKEARREVEVKLTPLILKPFGNSISRYNGEPEHIERN-<br>DAPHKNTQEKFSLSQESSYIATQYSLRPRREEVKLKEIDSKEEKYVAKELAVRT-<br>FEVTPIRAKDLEFGGVPGVFLIMFGLPVFLFLLLLMCKQKDPSLLNFPPPLPALY-<br>ELWETRVFGVYLLWFLIQVLFYLLPIGKVVEGTPLIDGRRLKYRLNGFYAFILTSA-<br>VIGTSLFQGVEFHYVYSHFLQFALAATVFCVVLSVYLYMRSLKAPRNDLSPAS-<br>SGNAVYDFFIGRELNPRIGTFDLKYFCELRPGLIGWVVINLVMLLAEMKIQDRAV-<br>PSLAMILVNSFQLLYVVDALWNEEALLTTMDIIHDGFGFMLAFGDLVWVPFIYSF-<br>QAFYLVSHPNEVSWPMASLIIVLKLCGYVIFRGANSQKNAFRKNPSDPKLAHLK-<br>TIHTSTGKNLLVSGWWGFVRHPNYLGDLIMALAWSLPCGFNHILPYFYIIYFTM-<br>LLVHREARDEYHCKKKYGVAWEKYCQRVPYRIFPYIY |
| SUN-domain proteins: | | | |
| Ostreo-<br>coccus tauri | Plantae | NCBI\|<br>XP_003079<br>402 | MKTTAVLNAKVPFAVAASLVIATAGCATAALGWHARETALALASARVSATSVDLGPA-<br>TRRIRVLEEQVEALQRNREDGRKSAERVVREALLDAQVKALGTQKGSKVAGLFKR-<br>DADTPALKKDLKAIETTLASLTKSQGAFATSAQLKELVDAVDALRKSSSGYASS-<br>DVVETLKVSVTELTNKTKSGSMKEVAALRKALEELASTQKSVDA-<br>RATSEVKSLRDDITSLKTNIQETYASKSALQTLDVSSELKSLEAA-<br>IAGLSKDRDSYATATQLKLLDAQVKALGTQKGSKVAGLFKRDADT-<br>ATLKKDLKAIETTLASLTKSQGAFATSAQLKELVDAVDALRKSSS-<br>GYASSDVVETLKVSVTELTNKTKSGSMKEVAALRKALEELAST-<br>QKSVDARATSEVKSLRDDITSLKTNIQETYASKSALQTLDVSSEL-<br>KSLEAAIAGLSKDRDSYATATQLKLLDAQVKALGTQKGSKVAGL-<br>FKRDADTATLKKDLKAIETTLVSLTKSQGAFATSAQLKELVDAV-<br>DALRKSSSGYASSDVVETLKVSVTELTNKTKSGSMKEVAALRK-<br>ALEELASTQKSVDARATSEVKSLRDDITSLKTNIQETYASKSALQ-<br>TLDVSSELKSLEAAIAGLSKDRDSYATATQLKLLDAQVKALGTQ-<br>KGSKVAGLFKRDADTATLKKDLKAIETTLVSLTKSQGAFATSAQL-<br>KELVDAVDALRKSSSGYASSDVVETLKVSVTELTDKTKSGSG-<br>SASKNLRDLEQQVMERIEGYINAIPKDTSMKLKKHIEKAATLWF-<br>ADRTGRQDFALSTGGGRVVGHSQLTPFVARGDGPLMSAVSF-<br>LRSGVHPKSDEWMLTPSMEQPGDCLALHGSYGYVDVRLRQP-<br>VKVDAVTLEHTNSLNAYDMHSAPRDIQIFGWYAHGNNCKHSKP-<br>PKSLIAMGNYTYHTAGDSVQSFEVSAPHTVDHVRLIVKNNHGHARWTCIYRFRV-<br>HGIPSS |
| Arabidopsis<br>thaliana | Plantae | Uniprot\| tr\|<br>Q9FF75 | MSASTVSITANTAAATRRTPILAGEKKSNFDYPQSESLANGGVGEAGGTSRDL-<br>SRGEATLDRSQGQDLGPVTRRSVSAATGTNTTATQRRTRKVATPKSEKARWK-<br>TVVRIFAKQLGALLIIVGLIQLTRKMILKASSPSSPISSYETEMAFSGLESRI-<br>AEVDGLVKATTNSMQVQVELLDKKMEREAKVLRQEIERKASAFQSELK-<br>KIESRTESLEKSVDEVNAKPWVTKDELERIYEELKKGNVDDSAFSEISID-<br>ELRAYARDIMEKEIEKHAADGLGRVDYALASGGAFVMEHSDPYLVGK-<br>GSSWFATTMRRAHTNAVKMLSPSFGEPGQCFPLKGSEGYVQIRLRGPI-<br>IPEAFTLEHVAKSVAYDRSSAPKDCRVSGSLQGPESSAETENMQLLTE-<br>FTYDLDRSNAQTFNILESSSSGLIDTVRLDFTSNHGSDSHTCIYRFRVHGRA-<br>PDPVPVVGTNLDQDSSPESE |
| Galdieria<br>sulphuraria | Plantae | Uniprot\| tr\|<br>M2YAH2 | MNLTSPLTRAKARELALAQSPSRSVLTEPLYTSPRRSLFDNTDDSAGLSRRK-<br>TISRSGRKSKNTKYSFSSDD-<br>KENISIHRSPKSREESSLSSQKGQGKAKESYQIDQNLGNAFQKDGTTSYSTLSSP-<br>PYFSKTSNSERVVEDSSPLTSNSLDPKNSSLHSNSNERKNRSFMQSSIDSKK-<br>RNHSKANVFKLLLILFLAFILIGTFIVFSLRTNTTLESSRRQFTSYRNMLGWFRAI-<br>FYRPKNERDSFETALSFMITQITDLAISLEQKKNHPEAILHSIQDIMEKTENILKE-<br>GIPYRELREWRETLYRQLFNFINLHEVNDISSNDIIARLLESKLRLYYKLTKQDYA-<br>LSSEGAKVICSTPSKLRQQSRYWLARMASFLDPSINTTVLRYPKGPETILSDN-<br>VSVGNCWAFAGKTATVTIQLSKTIQPIAFSLEHIINLDSKNVLNAPKLFRVYLIQD-<br>NKSENGQRLDWLVGTYQYCIEKRSRQLFPVRFQNLPYIDKIRLEIVDNY-<br>GGAYTCLYRFRVHGHEQQESSLQQ |
| Phaeo-<br>dactylum<br>tricornutum | Strameno-<br>piles | Uniprot\| tr\|<br>B7FQ97 | MRSKLSQLLLGVVALGTGCVLLVSSNADDTGLAGERHAAYQALASAASFQSIVET-<br>WHTRATQLIDRTVDLHSKYDAVEESVFEAEQAIRLFNAQQLKVVIEGFDAVHEAEL-<br>ERRAIQQPQDLEDVPMPLTKDEFRNAIPLDSIVDPSDVRMEHWVVDYIDRVL-<br>NERTPPTQVQTTPSPTHSCVTPQKAVQEVHAALVRHATDGIGMEDHARGARI-<br>VHEMTTTTYTPPPQSHQRLGNVWWRRFIPQDWESFLPSGWEEWDARVPLF-<br>FSHTFGSKAPVSAKPESILIPLTTPGACWPMDGSNGHVTLALAYPVAVSAITID-<br>HVSKYLLNEPSEQLTSAPKDFRIVAYPPCIEHCHGLSFDVNDPFDLAQGTFER-<br>DGTTVQTFATQNLDPPLLGREHTMEEGSCSAAAATTCGVPDANQGLVAAVQV-<br>QILSNWGNEDYTCLYRIRVHGESADL |

| | | | |
|---|---|---|---|
| Phaeo-<br>dactylum<br>tricornutum | Strameno-<br>piles | Uniprot\| tr\|<br>B5Y466 | MSKRKGEDLPVSPDDFSPPGVKTRSIRKSGKRQRRIQVAEES-<br>DAEPSSPSVAAFQNPPSSSSSIPTDRQSNNNNNNNNKKDDDDRDDSS-<br>DPEDPMEDSNRRRRTNPSKTHDTARRKAPPAASGSPKEAGENVPLSRRLDYRET-<br>DTRKESPTTLPTESVLRERDVLENGHDKTTTDDVDAEEHRRNNDHDDVRPHVS-<br>VHIRVYQFVTELVAGPTSAPQVETEMDVPVNTDTASAGSRTWMQLSWVWVLLI-<br>LAWHIICFPLAINPGLVTVSSTGNYLTTVYRAQAKLAQAVQGLQTVQHQQLARLR-<br>TARIALEQAENAFRSQQLAAEENLARLEQSWETEASSVMERLEKEEATARTLNH-<br>WIEQVLVEVPDDEEEEEYVEAVSVPPEIRNVLGPTQDALLDSSFITLWDVPEPVI-<br>CETPDVSGLAGGLYKEDVEQAISDLITDIVQVDEEMEEMVRKWVENYLDTKAG-<br>DAMTTTTTADANIPPLDGVVDADALKKLRAFIDGRMEVERADQTGLIDYASLLNGA-<br>RIIRVGDRSTSMSLVDQLPVFNRLAALLSLRFYGHGPEAALLPTYPPNALGQCW-<br>SFEPPASRRSGPFGVLTVQLSRPIHVQSVSIEHPPPELTDKSQTAIRSFRIEGFE-<br>DTQTHGKAHSLGSFEYDGQKGLRQDFDVDRNVPRLQSISLVVDTNWGEPYACLY-<br>RFRVHGQE |
| Phyto-ph-<br>thora infes-<br>tans | Strameno-<br>piles | Uniprot\| tr\|<br>D0NXG7 | MADGNTYTRRLRSRRRSDSTSSEEEEDPQRVTRSGSRRYGIYTPEPVQRTLELRS-<br>DEFEDDDEEEDSDFEELDDYGETVYRSTTYRPPQVYEQDVVELEDVHEDDE-<br>VDIEVQETPEHEAQRSELKRRAAGAAAYFKKSNKVDRMWQKVTDSKAMKTISKYL-<br>RRFWRFMLRNSFMAVNVLWLLAPLCCFVVAITVPHHLTTAFQYVDDLSSSWIGGR-<br>GNADAGFEKGAMRSVVQEIVDMKLVGLNEEIGMLRQTVQTQEHEIEALKLLHVTLR-<br>LDLDEQRQKFSLSEPDSAINVHIEKVVTKHTEELWEKIIDRTSQLQQDLQNATKQQS-<br>VISSVLKEQEEKMDSVQTIVEKTASTPAPDAASENARAMKKEFTQWRQSFEIELQS-<br>EMQRKVQAIESRMSRVLQDEKDALSSSADALRGLDATDPGILRVIEVAVQAVEIKKT-<br>GRVDHAALANGASVIHSERDLLYQDSSSPVQLLAQLVGLSDSDGDSRFTSPSYRR-<br>APAPFLGQLLSSGENPWWLSRHNGRPETALSETMEIGSCWGISGSSGRLSVKFA-<br>QQIVADAITIDHIPAQIASDFSSAPNQFRVLGISGHPLRETVELISFGNFSYASNGPAS-<br>QTFKLTSSLSQRSAIDGITLEVLSNHGNPEYTCLYRFRVHGQPA |
| Guillardia<br>theta | Cryptophyta | Uniprot\| tr\|<br>L1J5Q2 | MSSSHFPAWTSSSTVTPAEDSKNKNKDYIALGALAAVLLVIAAFAMSSGSKPAISM-<br>DADTHKLVSDMNKRFEEMKTMYNENVGLLKTAKQNGQVIDKNFDEALSGIKST-<br>MAKTEESMKKISESQRNFESVKTELSSDIQKLRKYMLDQIEEQKKSSEAKTADLEK-<br>SLSKLREKAEKMLSDAESLNSRLLQSLETAKTEIDSKMQKEVNSLLATERKAIEKKIS-<br>EASQSMLVAAKSEILAMAISKMNEELNEKIKQAVAQLVKQRQSDDLEKQIKHVLRAL-<br>FQERIGRIDWLLEVNGASITNHSETFPACKNTSVFSYLKCSTSQQLNPKTFIHSDVA-<br>GLSMIDSPSMMLGNCWAMSGSQGFVEIRLSKLLRVTEAVIQHIPKRISPDIRSAPRG-<br>FRILGSLSGAETEEGSFKVLVEGEYEVREDASEEELLPHVQTFAVKNSPLVDTIRYEI-<br>LSNQGKQEFTCLYHMRLHGEEATPMVSS |
| Trichoplax<br>adhaerens | Holozoa | Uniprot\| tr\|<br>B3S288 | MALNNQTDFPQVSKIPDNEGLFAQGSLYCSMKRDSLGSVA-<br>GRKSVRSNNWYRKYGGMTYDHGPLRALIGLVRVFVIIIYTISALIIYTDAVLIIKIKGIIK-<br>TAFMLPLYPSLNIKTSHKTPTQTTLNASLSLAIIVVGLTCTIAVFIYPVTFQK-<br>IQSVSSTLLTKSSGDPVIKDTNNTNC<br>LNGIGDRIRIRELEAVIMQLKSEIQEIKSYPRQKVILSQDITFNIPQTLNRVYLMINYLE<br>DNLKELKKSMISKEIVEQIVNSALKLYDEDKIGLADYALYPAGGRVISIGN-<br>TKPYLNSEGKPFHSPNIMIQPDLQPGNCWAFEGRMGEVTIQGLDDIYAHEKLLLGS-<br>FTFEDSNVMNLQRFTVQHFPNRPFNLIKIIFTSNHGSSYTCVYRFRVHGFLEKSTL |
| Monosiga<br>brevicollis | Holozoa | Uniprot\| tr\|<br>A9V320 | MSVVRASTPTRQRRRTGPRTPPGFATNTLFSDDEDADRSVASEYENQAET-<br>FASVLHSRINIATPNTSSSIRHVQTTTELRRVDNDLGILSEDELELGPNETLLYDDDG-<br>PIVYERRVHRVLRNTSEDDSASAQTESVGRSPHAFAPRIIDSALLAARYQQERT-<br>SLDDVDKALQSLIQARTQAEASAHSQTERPKGHFTICKQIKACVSS-<br>NLIMLRDKLARLEAQQTNFTHQQLHVRDALNSQAQIMRQTMMT-<br>MAEAQFVNKQKASNASRSAAACPKCPSCPTCPTCPTCPNCPTNA-<br>PTKACPETAPAPPSPLVNLASYARGARIIETHCARARSSSWRSR-<br>WESLFSMQPDQSARAMLSDSMEPKQCWAFRGAAATALIQLAAP-<br>THVESVALSHVAQAALPASHNQSSAPRRFRVWAAGNSAAPAMPA-<br>AHKMLLEADFDPARSPQSFAIPAVHQHEARFIKLEIQSNHGNEYTCVYSFQV-<br>NGRATLVA |
| Capsaspo-<br>ra ow-<br>czarzaki | Holozoa | Uniprot\| tr\|<br>E9BZX3 | MAPRVGPESTPSARRASILVAADAATDEAPRSVRRSTRLQQLQQQSHSHSQA-<br>GAGQHESGDETVSVSNTPARARAPHSAVRPSRTTAAAAREDTQLQLQVPASV-<br>GRASRSGLSLRSAAHDNSSADLTTPTKKSRSARSASTTATAAAAASAAAAD-<br>NGAYAVNEDEAPSTAASTSSRWTASSKGRSESLRSRALDLSFTTNASQPDLDS-<br>SVLMHSAMEDADEDEGDDAENASMTAARARLSPTTRSPRSQATAKSPPAAAAA-<br>AATDNRRKGLSPTSFSSPSSLRSGSPKAAAGSTPINSHSQSPATPSTLTRIVNVL-<br>TSPFRRSAEGDSSKSAASSPGKPSPLRGAPLAEVHRPQRSADDDVLFGLDSS-<br>DTEDDGGSKSDKSRARAPNVAAATTAAAGSTTTTSFVRQLPRSLGDLLRSIGVL-<br>FYLFTTSIMLLNVFLLAKGGQALRRIRGGGKPSARSAAAATATPSQSPSRSLSYS-<br>PHYRVRGSNLDDLDASSDEELLHDQHRGQSASIRRQLGEVVPALARRLWKGAL-<br>VALLILLVLALALRRLPAGVQHASSPLLDHQQVDDRLKLVQASAHDGNLASGKSV-<br>EELVQLVQLLSAELTSLEKKFLAAQKDDDRRLEQLSADAAAAAQKLTQQAAQKP-<br>AQPVVDDSAKGANLALQAQAKELDTLKGEMERLKAALDKESNNYQTADSKLQE-<br>SIRSCVTLDVLSEQIAPQVNSIVERRLSEATLPKSTDSDTVAVQQAAIDPETLRQL-<br>IHEEVAARAPTTLSEEEREKELSNLVTRVIAIMEPRNREALDLEVSRLNTRVSTLH-<br>SEVVQFQQQQQKHQQQQQQQQQQASVDSEDVRALIREAIALYDADKIGVVDF-<br>ALASAGGSVVESCTSASFSVPRLALFGMPVMMQSTSPLLALQRDNSVGNCWA-<br>MSGQSGQLTVRLARSISITGFSVEHVAKSISLNTMKSAPRAFRVVALNAACDTEG-<br>TLLGQYSYDIDGTPLQQFAVQSKPSRSFEYVRLEIASNYGEEDYTCLYRFRVHGVA |
| Encephali-<br>tozoon in-<br>testinalis | Fungi | Uniprot\| tr\|<br>E0SA25 | MNRRDRLQVKRTPDSTLNLGMDETIILQAAAPKARANKSVEPMEG-<br>NGYGQERKGIKDYPIYMAIAIPYVLFFYMAIKRPMDSMILTNLMEEINILREENSRIS-<br>SQIEMMKHIKEVNYAKIEEGARIRIESMSQLFSYGFLGFRKHKEPSTIFDENVGIGE-<br>CLAFKGAGCKFSIDLEKEAAISKIGLYHPVTKDTSSAIREFEVFSNSPEGNL-<br>LLGRFEYDTSTCGFQTFEWEETPISSVEIVVRSNGGNKKYTCIYKVYLFGNK |
| Batracho-<br>chytrium<br>dendroba-<br>tidis | Fungi | Uniprot\| tr\|<br>F4P736 | MGTPAADSGEVSGTDLSDTLDSTSGVRRSKRAHRNINYSPIRTASPSKRIGHPAAN-<br>HESSISVNTIRRTSISSTASDCSRSSRTSRATHVTSISSNNTLSPITTPTARSYSHRN-<br>VLFSTPQDPILSPSDSVLIKPDTTEQPTSAWNHPFRSTNNGIDSGHEINSTKSF-<br>SSTESTKRTPTKPKIARHKMAKSNPAASDSELDNDLIPKTPNSSQLNPSKTPESFV-<br>EITPSKFSLTSFFSPFRSPMNLKHILENVGMTPRTHKRKVAKSWLGRMATSSDQIE-<br>VEYESDLCDKSDDELGENRILFPDIFAEEAVIEEPGPLLRFIDWLSDFYIVQLVIPIFS-<br>WIASIIALLIWGVCTLMWNIVYYLCGKPLGFIGWTFLRVAGFIFFTTFRVLGYHRNM-<br>CVAGLLCGLGVYLLTTKPGHSIDLLASLDPVNQSPQLQSMFTPNISMIFEHLRSTF-<br>ASTETEHIEINSQDTQSNTDSRPESNWKSQKQLVMLEKRVATVEKKLLLVTQGLV-<br>EVAGDMKSQHSRLEKSIEHTIAGQDSLKDTLSAQIADVTKQIFDLRNTLDALHDAT-<br>EKTTIEQSAYIKHLHDSLEIHKSSIVELEKQIHEDVQNLSVSNEVSKGLAEKLSTLSE-<br>TVTRLGQQLEAYGSVDDVVKRVMDTIRTSTDFPGFIAATKSSETSEIELPPELWKA-<br>IESKLNMFGDKDQTIDVSNTAEARLKTFILAQIDDIRVTYATNADVDVRIRMALKDAI-<br>KDYTQSGQNDASLAARVDLKMDEVKALLQTKVEELNKLEQETREKLQGAFSGTK-<br>KEYTQLDLHLKTLTEQIASHTTSLKEVMSEQDRLGVFYDNQKREFELIKTTVDTS-<br>THWHNFLEQNRQALSTIIHEQVDTHPNSHIILTQEQTLSAIEVKLESIVSKSSQDMAI-<br>IMNRLESLDSVSSSYAHLKGDAPLSRDATESLIHRIVASALEEYRADVLAIPDYALE-<br>SAGARVVYNLTSSTFTTHFKPPVLGLFARVMGIRKASGRSPSTALTQDISPGNCW-<br>AMSDSSGTLAISLAEPIIPTDMTIEHSHIQTSISDRHTSAPRQIELWAVFDAVEFAKL-<br>DLNNNQVRLQTLGTLSSSNKKTQPAGILLGDFEFNPMTAALKTYPLHRILNIKVNM-<br>VVVRIKNNWGNPKWTCIYRVRIHGRE |

| | | | |
|---|---|---|---|
| Rhizopus delemar | Fungi | Uniprot\| tr\| I1CPD1 | MELKSISKKPYAFNSHPEDDTSIDEGYEDFDAYDELELMVNHPELEEAMR-<br>RMIQQREQLQQMEEQLLAQHKQQRKNQSNNETQQEAQEQPEGSLIRYYASKWLE-<br>FIIFIFFMIYWIIKEPIIRTVTFFTMIVSSLLVTPAIFVWSKLFDQWTPNNELRKRITSLST-<br>GLLFAYLAYLAYPRVQPYIPTYHWTHATVNPPTADLTNIIRHISRWEERVNQL-<br>SDKQVRHEALYNDLSSKVNSELQSIRNQIKQSTSNLIQRISSQQNNIEGINSQ-<br>LEQSTSNLLQRMSNQQGDIEAIANQLEQSTTNFLQKFTHQQGDIENLSNQHS-<br>NIANQYNDVANQYDSLTNQHSNLANEHSSIINQQEALVQKLNEIEKMLSSVE-<br>WGHSQGSWPDLTQQEIQKYITDKVNQFIPHETADFALESRGARVIHTMTSKT-<br>FQPMKPWLQHIRRITGVSSRLRTIPEMALQPQTYPGECWSMEGTSGSLAIL-<br>LSQPVHLESITIEYPTPEIMSFNMSTAPKNIQILGIKDFKHHPESTVSLGLVQYD-<br>IYKNQAIQNFLLDSNNDVFEAVIIKILSNWGNLLHTDLYRVRLHGAPPV |
| Giardia intestinalis | Metamonada | Uniprot\| tr\| V6U6N9 | MPPRTRSSGALVRMNPTREDEAYTKEVLRKTSDGFRAELTSGKSERITQNLRN-<br>QDEGTLRLQEYLMAAEVDARPPKKIDSLMDQSDALKLQKSLTALQVA-<br>PIQRSRVQEPPRYEITSEDKDSSSSKPHQAPLSETSSTSTPPESSSTPSPPPSPP-<br>PRKSPKDVPKPATPTPVKLPAAPIKTKSPTIQPSQEDEYTDEYVMVKQRVK-<br>RKPRAPSKLSASTKVVTAGEPGMLYAYRKAWGCAEYVYASVCGLLALMVLF-<br>IFSYLLMRLWTGCAGLGIVGTSAPLVPVYTGDGTAGVSCTGPSLREIQSLLE-<br>SNNKILLKEWGNTRTFALDSKEVQSIATQVANKLSDQHQRLELKIKEIVDKH-<br>TARTADQGITTSQLKDLAKTIREEVVSATKERISTLSAAIAKFEKEMLDAQSV-<br>TTELLKDLFKNSSFSVSSASAKKASEKIKAAVGQMSDAADAAVAAGSANFTA-<br>LFTMIESLSRKLETSYRSENITKDLQSLHQSILLDIQTQLKEVAATTTQMIGDS-<br>LTSIRAQTDNLSATLIDFVAKSAAGAEASISGTAPVTFASESLSSMQRAIDRIV-<br>IALGELSSQVASGVNNKDVHGDSHSSLATLTDIEEMQRKTAQHITQQAENVTL-<br>TILERIDASDNRMSQEANEHVDHLKTGLAGVIKKLLLKQTDYSSGSMGTTP-<br>QGVVDELRLQYTDFTKQSFGTRIAGKSDDITNIAESLKTLISGNERVRLMF-<br>NDNMSPGSCWPTKKTGYVVLRFKHPVTLYYGSISHPAAPKLSTGRTTVPR-<br>DLTFTGRTTTGKEVQLGSFVFDVDGPEQQAFRLQENHDIIQVRVGFTNNGGEYT-<br>CIYNLGLFGEKDSKNP |
| Naegleria gruberi | Heterolobosea | Uniprot\| tr\| D2VL48 | MAPIPELEDDGSLHDSERDVSMKETPAPTRKKNQSHKRKSTDDEAVEDAKKRK-<br>SNPTEEEQTILEEETFRTDTPFQSPDVRRKSTGLRTKPQQLNQQVFVNRKHKSVP-<br>SNVLDANTSYQTPKKTNIEAEVPPSISTFIPEKQFAISRNKASKTGTYTILASLVAI-<br>ISIVYLIYVQYVTLDIPKTNVTEESNLSNNTQNSAPIIKYFYVTNN-<br>VTSNVNHSEIFEKLIEKHSKQFKNNMLDLIDTRLYELERKLNNN-<br>IRSSISSSSVSVKKEQQKELEKLKESLLSKISTEVDIILSQKLKSI-<br>NDINEQSISEIKGRLDTIIPGVKNLIDESLDKYDADKIGLTDYALS-<br>SLGSKIVEHSPTYSPSKFWPQLFVPVKTPDMIIKPDTTIGNCW-<br>PMKGSSGFVVIEIAHSIIPTSFSIDHVPKALSPNISSAPKQISVF-<br>GYENETTLTKLSAFEYDVHGSPTQTFPVNESTNKKYNKFRFQ-<br>ISGNYGNSFYTCIYRFRIHGDSQAMIDKTFADGLIRVLEPLTTEY-<br>DSKVKDIQISQTKLAEEIDRLAKKLDSCKEEAQFVNVAPYLQKL-<br>ANSRKRVINISTTLGHISDRLSRLNKLAKQKYPELEQLRQQR-<br>ERLKQQGSSGKLVVQQQSSSSSEVVPATSTTTPSSTTEQQS-<br>SSTTTPTEQTPPEQSSTTLSNEDETTKSTPSTSEEAQDTNTNITTSSATTEN-<br>PVEAVDQQ |
| Acanthamoeba castellanii | Amoebozoa | Uniprot\| tr\| L8GXN1 | MPRVYYYRLRQQPASALVDAADSGLHYRGQQGKNGECEEGAGVYGHATEEIPN-<br>QNQVFSLENEESDDFLEEELPHKKYVEEKERTQLGEELSQTTSSVGEWLQQAWTL-<br>LLTIAYLLIATGTFVRKLWTTKTYRYGLLVALLVALFLYFYQPTPTVVEVP-<br>VAKPGVVVMPGGTTTVVYDDRLPLNVEVFVKRLVEQQLAKKQDDTLSRVMQKLA-<br>ADKEELKRRSKEELLHYLDDKLGKMDAMIRAHLDTPATINDSVSKELKELEAKVGS-<br>ELQHLNEKLSKMDVTVRSRLNAPATTATAAAPIAAVVDGSVSQELKELEARVESEL-<br>LRLTREMKSGNDGSAEAKRSLQAQLDALQRRVSELTESVDSKIEAARLSQEQASV-<br>ALSDEQLREVVRLVNANQRAVGAQSLGEDQVRELIQKELEVFAADRLNMSDWAL-<br>GAAGARILHAYTSDRYGSSLLGHLLGQVGEPPSALLTPSLVMGHCWAFPGEQGNA-<br>TIQLPRRIKPTAFSLDHVSRGVARDFRSAPQHFKVWGYTDKAAVADESKSVLLGE-<br>YRYDIYGTQVQTFAVQPDAQLDAEQSFSVIKLAVSSNYGHEDFTCVYRFRVHGDAV |
| Dictyostelium discoideum | Amoebozoa | Uniprot\| sp\| Q558Z2 | MSGDYKPNYQSSPSRKRLPLQSKDQASIYKYQTPSTLNLYN-<br>NTVNNNSSNNSNNHLLHNSNPNSSYLYDSSKQYSNQINIRNNSNSNSNTNNIT-<br>SKKASSSYSINNKVDHNSHNNNDDDDIEDDVDINYSTNNASSNILHNRF-<br>SNSNKDDSYIDYSTDENPKILKQPQPLYNHLNNQIQQQQQQQ-<br>QQQQQQQQQQQQQQQQQQQQQQQQQQQQRNNNNNNSNSSN-<br>NNNTSTTIKRNNQQIDNNSNKNIISKFIGDPWKNFYYGSNKSLWP-<br>FERNNNSNNSSNNNNKVNFKQAIWIFIFSVLFIGCLLGLFSTNFYGI-<br>HIYFPSFSTTKTNSPFNSTNNNIQFSNLITKEQLYPIIDEYFKKNEILK-<br>SYNKLFEKIENDIKYLSEREQYKDIINEIKEELKLVKLSNMDEDRVNQ-<br>LISKMINHYNNNENNKQELKELLSKSIEELTKLKSDSKEQLIQISTE-<br>SMNQLGQLKSESINQLGQVKSESIDKFQSTLKSLSKEEQSKIERE-<br>FNHQFNQLNKDADQLLSQHSLKIEKLREEINENQQSSLLKLTQEY-<br>KQLEERLKEFSSKLQQSISSSSMDQFESWKLVFIKDIEERINKESSK-<br>LTNQYIQLTQQFTKIQSFIKDNPSIDSLTNTIESLEGIKLLIEDILEVYS-<br>ADKIAKVDYALGLAGASIEYNALHYRVSETYPPIKGSGSGSGSGG-<br>ANGNSLGLYYYNLATNWIFPQPKPNPPETILDPMVNTGSCWGFYTG-<br>NGTIVIRLAKKIAITEVTMEHISSNISHHIDSAPKEFQVFGLINSSDIG-<br>QSLGVFTYDTTINRHLQTFKVNKIQSTTTTTTNQDQNDDDNIQEFS-<br>HVALRILSNHGYRYTCIYRFRVHGYQIPHPEQEQIQIIQE-<br>EQSFKQEEINQQQIEQIEQIEQIEKQQQSDEL |
| Toxoplasma gondii | Alveolata | Uniprot\| tr\| V4ZQQ5 | MDSFEREAEPGIGREAAHAVAFSRAYEHRRLTRPVLLERESPLYHRQSASRL-<br>SQSALSGIKARTQDTDSAACRRVAGQFAGSVDHAADVKRPTGHGAAATPRASPTF-<br>SAFARPEDAREEDAETDSEEDYDDEDEEEDEYEEEEASPLRPNRQNGRSPRP-<br>SASLRLERQPLPREGDPANPAPPAPRRVSLWGSILMHARRSLPFLGCDGPDGSL-<br>PPSPKPPAYDSEDLHQLGVSLLSSLSAPRYLARKLGKNLEHRLHALCPDDAGSG-<br>GTGATRLCRGAAEERGGESEKSFSCSAGSFSSLSRFPASDSTAHRRLEKNSFTPL-<br>SIRAHRVGAFDAPDVATLLWASLRRRLSQCFVFFFFLSVLFGVYRHLAWTASFSD-<br>PDRDLRGELPGTPAGRGSAAAWLVEKEIQKLRRELEEERAALSEYRERMQMQL-<br>KKHVASLTQKVEDNLESQRKDLEDLFVKLREEKNAQGETEEGEQAQQLLADVDN-<br>LQEEIRGLEKRNEALETTVDDMRRNLAALAQKQDEGPQKSLLTQAFARKVEDLYT-<br>HLVVEEEQTIDWALESLGGRIVVSETAPPLLKPPSWASSVSAAMWSLVHGEEAAE-<br>AAGAVGFWAHKKPAVMLQPDRHAGSCYAFRGERGTVAIQLPTAVFVTSVAIDDVA-<br>SDLFTASAPRRFRLWGFEDPRASSAVSRNSFTKNTGASLPSESNCGVLGKGRL-<br>CGWFSGFLTALGKQEEQGGGNLSGVRDSPLRVFLGEFEVKQRKKLVLFELKEKP-<br>VRPLRKIVFEFLNNFGHPYTCIYRLRVHGEKAVLKSTTLN |
| Plasmodium falciparum | Alveolata | Uniprot\| tr\| W7FJ75 | MSMNISNSSKNTFISDENNVTYEDDYNETDNNKDEGDDRNTLIHVLHSYED-<br>FQKKNMNYGKVNPYKKKETQLSKIKKNLVRLLSSNDI-<br>TIHKFNSEEIKGNKKKKMENIKFLTSRAAMWYQVEKNDDPDYDLKLESSKKKNFVY-<br>ITMNYINIFMNDLLNDKRGMTYIATFMIVLSIIITFISGFITLYNNNNNNNINHYNNNHNNNH-<br>KYHNINSSNNNNNNNNTINSNAFFSTNEQYLKNLDEKNNWNLNMSRNNYDDINQYM-<br>NYSALQIKRGNDQENYKKYNEEIFEILEQLKKEINENKNIKSSEKIQNENKKDDKLNI-<br>YEIRLNIEKKINELENKIINNNKNSDSFKSNLIKEFENFKNIFHDNYEKFQTQFKDYTN-<br>IVNNIKSVIHNKDTFINNIQKTFTQNQVDIKNNLTSHIENEKKELLQKINELQSQVKVM-<br>EWNILKQENLYKGMKQNILKILGQNKLNTIDNNNNNNNNNNNNLYDADNDSSYDDE-<br>WFGENKNILMGPPIDMDKKKKKQENENNKNNKNYSNHRFSHNYMPHHISKDIHSN-<br>EIETLYTQNEFKELLNVINDIKEQINMLQEKNINSKNYLDDTFLQMEEKILKNAEYKIK-<br>YYLEIYKKDILNEITESKVIYNEEKFKSLTLKHERLQADLLKNINNQIKIQSKLIKDDISK-<br>SIHFMMEQKKGKHNYNNINNNNNNNNIISSSNGSSNNNKMIYSDHLEIIQKKVDELYN-<br>EFILDYNEIDWALESLGAKIVYKMTSSPLNRNDFIEKFLNQIASFLPSEEIYGMIKPM-<br>GKDPAIVLKPTNFPGDCFSFKGNHGKITIHLPATIDITSISIQHVHENISNNSNATPKYF-<br>SVYGMVDLNWPEQFEENDINYDDFKNSSLYSCLHSTYGNIIQPNEILQRWLKDNKQ-<br>PNLIHIGDFYFDRKKRIATYPTKSCFPMKRIIFEFTENYGASYTCVYRLKVHGKRCI-<br>RKFK |

| | | | |
|---|---|---|---|
| Reticulo-myxa filosa | Rhizaria | Uniprot\| tr\| X6M7T6 | MRNKDDLLLKQVYNKKQINLRMAPKNLITCNIWLTTVKKKTFCRCM-<br>FAAKKKKKKKGKGDSNKELIKEWTGSVFRSHQHFLLGISIVVAVTYMFTYGMVTI-<br>AANMDNNKYSARAFSLENDTFDMYTDGQSTSSTILDIEIKDLGSIHDLLFIQHSKG-<br>PITDNSAKRSDSKSTMEPNTVNDSIPINWSQIHIGSKVTLYLDAGDTVTAIIRN-<br>VDVEGQRIFVKYINCWWKCSHWIDLKSGNLVTYAKDNLGISNGKLKHQTKVL-<br>DDKISVEDIITNGSFFFFRGCPCENEILKLSRGIQKLESKIEHVLEQSKPSSTQ-<br>NNGAKGREPMDFLVLKQVVEEQQQALDLLRGSVDALRLKSSAEHSSLPLKT-<br>SSTPTTSTSAKVGGEIENRPTLQEFEKRLETNLFEKILLLIFLFTIITKLNKYHQ-<br>EVKSEQQLAIEGVITSVKELRSSVHSLEQQLKAMEKRKEKDGAESKDNETLL-<br>SLSSPSNDWSYQVIKHSMVKSETWIDRLLDYVTLNGWRSYRQQAIMGEVTS-<br>KTPGECIPLDFQHVLSNNNDDQLQKLTDDILLRNPNLQYAYLHVELYRPVIIKQ-<br>FSLFHLHSDLIPDDITRRSAPKLFHVLASNDKERWFNLGLFVYDYDDVKRTS-<br>PDRFDGVHKYRVAQHFHSDGNGYNGALKFKFVVFRILTNGGSDYTCIYRVMVHGTE |
| Bigelowiella natans | Rhizaria | jgi\|Bigna1\|89433\|est-Ext_fge-nesh1_pg.C_490068 | MPTTRRSSRRSASEQRELPGDAQSKRKRKTPGRRSSSTSSSTKRRRSTRKSS-<br>RKSSKKAPLMESVPEDREQQEDNSQVVGIQGVNLVESFDDVSNEGNEEGLEGIDE-<br>VEEEEQEEEDLSISSAAHAGLRRRVRQGRDTTTTAATTAPTPRPHTTSPYTITVTPP-<br>KEDRPDRRKHIFSISIILLLLLGIYYILQLPPSGHNREAIDFSKMKAELNGRIKQK-<br>MKTLGSDYDRTVKKLKSEVDEMMKNLHEKYENFGNDNTDVVKKATDFAKEQI-<br>RKNPKFHKAIKEEVQKRMNEYTEKIHKKIDQLQAKQNQFRAEMEKQESALSTL-<br>KANVDTHAKDYTEKKMKEIQSELEKMKREFMLEVKNFKKTLNQPIAGEQMSQ-<br>LKIWTEQLAGDTLDLMINKYLEKFAADRTGIVDYAMAATRGRVVDHSPTYKQK-<br>KRGVTNIMNFLKSSQPISRNPPAQVLKPGIEPGNCWSMEGADGFVLIRLSKRI-<br>VPDKVSLEHASPKILLSTGSAPKDFEVYGTNAGKYTDDMEKTLLGRGTYDLNG-<br>KSSLQQFNLKAGGKSFNHTLFKVKSNYGEAYTCIYRIRIHGSEL |
| Bigelowiella natans | Rhizaria | jgi\|Bigna1\|84919\|est-Ext_fge-nesh1_pg.C_10366 | MSQRTRTGLWSLIRARFLPSQGNNMEVENEGNTLRSSQPERKRRRRGSP-<br>SRAHTRDGTLRPLNPSHRYQTRHKENMLHMAASLDVVASAVRFRRRRSRSP-<br>SRESKRKRQRIAAPTGRIIPGTDIIVAGMRGNGIGNLPEDQTEAVEQRLAQELHPF-<br>INGDEDEINVEATPRRLSSRLKKLKSPAPTAELSSSLLGEEAQEDIMVQGHQN-<br>TKHAHNLKAPEATVARRGSPDTIFELLRFFIIILLLLTTALVARRIYYKSTIKTGTEY-<br>FNDVDASLDENLARSKQRQISKWNSEERTLRHEVTSGGYRSQREIKENFLQEV-<br>KKSIKEWVDKDSEDQILKILLEEGNVIARDHISLSNPLEPLGSQLEKMEGIFDM-<br>KNQFMVEEDSLLERVEKAIDEVINSKRSAIASKFATMKENVKSEFADSERILAAI-<br>EKYAADRVGEIDYASKLWNSRIIGKSESAKSNTAKSFLPRVWSQFKDFVNPSR-<br>RKGPDVILENNMNPGDCWALNGPAGYVTVKLGRKVKVSKVSVQHISKGIAPEK-<br>STALKKFKVWGKLSGKDKESIQIGHASYQFGSTFSVPTIQYAEMYCEEWFDTIT-<br>IEFTENYGGSYTCIYRVRVHGGESARNLDGKTMNERSERLNNVTPSNHKSF |

**Edited alignments used for phylogenetic analyses:**

Lamin:

```
>Dicdis
SPNRAAQRLREKDELSLIHNRLKSALKKLESAETELEKKNQEYEELDQKHTATIKQLKQR
SDQVE----KQLIEEQNQNSDLTSNRNILEN---ELKSKESVWKKEKDEILLKFQESINK
LNQENSLQSQLKSEIVSKEYEIDGLKSEINRLKDDLQYRIREGEEKDEEIKKYKFELKEK
EKSSNAM-----NKKENELNNLIQAHERQIEDMRDSINREWELKAAQMMEEHHARVDSFN
EEKERIKSQMETLNGQIEDINIKNNEYEDRIKEMNVLLSQKDNRKQADLKSKDGQIALLQ
IEINTKDNKCNTQIDPEIPLDPEINSLKELVKGFEKTV-------NPDTVSFSLVDSNQE
FIKLSVHGDNGLSISKWRLIVVKPKSGFSFPDGIQPFKGKSVTVWTGRPRQGTPTENEFY
WARTELWTSEGTIVKLVSPSEET-TTVTLPA
>dicpur
SPNRNALRLKEKDELNNIKSKLQLCLKKLESTENELEKKNKELDDMEHEHSLAIGNLKNR
NEHVE----KQLIEEIKTKSELMSSRDALES---ELKTKEASWKKDQDEYVTKLTETINK
LNQEHSTVSQLKTDIAAREYESDTLKTEVNRLKEDLQYRMRESEDKDEEIKKLKVEVKEK
DKLHSAS-----SKKENELKHLIEAHERQIEDIRDSINREWEVRAVQMVEEHNARVESFE
EEKQRYKGQISLYGAQIDELNIKNNEFEDKVKELNDLIAQKDSRKQADLKNKDSQISLLQ
VEMNGRESKCATQTEPDIPLTREIEEIKTLVGVFEKNV-------NPNTVTFAVVDTNQE
FIRLSVHGDNGLSLSKWRLIVAKPKAGFSFPEGIQPFRGKSVTVWTGRPRDSTTTENEFY
WSRQGLWSQEGTIVKLVSPSEET-TTVTIPE
>dicfas
GRSSTAARLKEREDLIQLNSRLRDLLASSEQKDNEIRRLRDEYITTSRQSTDQISNLEKK
LEDIE----ARLGNESKLTVELSSQLQLAHQ---DARSKEQGHQAEIQNFQNKMDETIKK
ITEENSQVVALRNDNSKKVMEIESLKSDVRQLQAELLTRSKEAEEKDAEIKKLKTELKEK
EKLQVEY-----SRKESQLQQTIEGYERQAEDMRDSVNREWEIKCVQLVEEQNAKANSFE
DEKEIYKSQLQVYQGQIDDLNIKNHEFEDRIISLQKDLDTKDDRKVADSKKKDIAIAMLQ
DEINGKDVKCNSSPEQDIPLTREIEQLKSLVNNFERRV-------QVNTISLLSIDAISE
CIRLTVSGEDGVSISGYKIVVTKPRIGFSFPENIVPFKGNIINLFTGKTRPGTTPPNEFY
```

WSRPNIWESEGTIVKLLTPTDEVLANVTLPS
>diccit
SPNRAAQRLREKDELSLIHNRLKTALKKLESAEIELEKKNQEYEELDQKHTATIKQLKQR
SDQFE----KQLMEEQKQNSDLASNRNILEN---ELKSKESVWKKEKDEILLKFQESINK
LNQENSLQSQLKSDIVSKEYEIDSLKSEINRLKDDLQYRIREGEEKDXXXXXXXXXXXXX
XXSSNAM-----NKKENELNNLIQAHERQIEDMRDSINREWELKAAQMMEEHHSRVDSFN
EEKERMKSQMETLNGQIEDLNIKNNEFEDKIKEMNVLLSQKDNRKQADLKSKDGQIALLQ
IEINTKDNKCNTQIDPEIPLDPEINSLKELVKGFEKTV-------NSDTVTFSLIDSNQE
FIRLSVHGDNGLSISKWRLIVVKPKAGFSFPDGIQPFKGSSVTVWTGRPRQGTPTENEFY
WARTELWTSEGTIVKLVSPSEET-TTVTLPA
>dicfir
SPNRAAQRLREKDELSLIHQRLKSSLKKLESTEIELEKKNQELEEIDQKHTLTINKLKQR
SDQFE----RQLLEEQNQNSDLTSNFNILDN---ELKTKESSWKKEKDEMLSKFQESINK
LNQENSLQSQLKADIVTKEYEIEGLKTEINRLKDDLQYRVREGEDKDXXXXXXXXXXXXX
XXSSNAM-----NKKENELNNLIQAHERQIEDIRDSINREWELKAAQMMEEHHSRVDSFN
EEKERIKSQMDTLNGQIEDLNIKNNEYEDKVKEMNVLLSQKDNRKQADLKSKDGQISLLQ
IEINTKDNKCNTQIDPEIPLDPEINSLKELVKGFEKTV-------NPGTVSFSLIDSTQE
FIRLSVHGDEGLSISKWRLIVVKPKAGFSFPDGIQPFKGKSVTVWTGRQRQGTPTENEFY
WSRQELWTSEGTIVKLVSPSEET-TTVTLPV
>dicint
SPNRAAQRLREKDELSLIHQRLKASIKKLESVEIELEKKTQEYEELDQKHTATISKLKQR
SDQVE----KQLIEEQTQNSDLSSNFNILEN---ELKSKESSWKKEKDELLLKFQESINK
LNQENSLQSQLKADILNKEYEIESLKTEINRLKDDLQYRIREGEDKDXXXXXXXXXXXXX
XXXXXXXXXXXXXXXXXXXXXXXXXXXXXXXXXXXXXXXXXXXXXXXXXXXXXXXXXXXX
XXXXXXXXXXXXXXXXXXXXXXXXXXXXXXXXXXXXXXXXXXXXXXXXXXXXXXXXXXXX
XXXXXXXXXXXXXXXXXXXXXXXXXXXXXXXXXXXXXXXXXXXXXXXXXXXXXXXXXXXX
XXXXXXXXXXXXXXXXXXXXXXXXXXXXXXXXXXXXXXXXXXXXXXXXXXXXXXXXXXXX
XXXXXXXXXXXXXXXXXXXXXXXXXXXXXXX
>polys
QSPLSINRMKEKEELTEVTKKLSKILTILEERNNEIVRLKAELNDSNKSSSVEVSALRTR
LDETE----NRLTQEIKQRAEAASTLELTLA---DLKNKEDLYMHDRNTFDARLEDTISS
ITKEHSALTVLRTDLNKRLHEIEQLKGDIKLLNADLLTKSKEQDEKDDDIKKVKLTLKEK
EKIIIAN-----QRKEAELNQTVEAFERQVEDIRDSINREWEIKVAEAIEEQTSKAKSFD
FERETFRNQIAVIQQQTEDLNVKNHELSDRILDLSNELRNKEQRRSAELKSKDGKLSLLQ
DEISSKEIKFQQSMDPEIPIGDEIRRIKELVNTLTHQS-------KLNTMCILSIDTTKE
TIRLNASGVNGMTITDWKIIVNKPKYGFQFPANITPLKGNVITLHTGRSRNTTAAENEFY
WSRTGIWDSEGTTIQLVSPKEVLQESVSL-T
>polio
SPSRSVMRLKEKDELNAIHKKLQQCITSLKEKDEEIEKKEREIESLQKEKTKSTNAYKTK
LEEAE----RLLSQEIKEKTEFLSRADFLES---ELKTKEATWNKDKQDMISKMDEAINK
LTHEHTLLSSVKSDLVKSEYDNETKKNEIARLQGELHTRVKEYDEKDEDIKKLKMELKEK
EKLSLVA-----QRKENELNQTIQSYERQLEDIRDSINREWELKTAAMVEEXXXXXXXXX
XERERFKTQITVIQEQVNELNIKNQEYQDLIEQLNNTLMNKENRKLADLKNKDSQISLLQ
DEINSKETKLITQNDPEIPLTREIASLKELVNGFEKSVXXXXXXXXXXXXXXXXXXXXQE
YIKLSVHGKDGFCISGWKLIVLKPKCGFSFPENIQPVKGHTITVWTGRSRHIQTPENEFF
WSREEIWTKEEITLKLVASNETT-AKVSLPE
>trichop
MSPLKIKRVQEKEELKNLTDRLASYIEKSRFLESRNARLNEEIRSSRRSGDENIKSLKAL
YEQELQEARKTIDEMANEKSKITITVMKYTKQVEELTSDLEKVKASKSSLQVALNNAEKR
ATELEGEVVNERKNVAELREVVIGHKEKIQRSSEELESANKLAEQHQQEIRSLKKNLKTV
DDQRSTLQDDFQSEYDTKMTEALQQLRRDNEENSKRLKQEVEELYRSQVKELESQITELK
NECRKFRDKSESISSEVHVLRAENSSLQTRKRELEESLIEERQKNTNKFEEYEMEMRALQ
LKLEEQSTEYKELLDVKIELDNEIAAFRKLLEGEETRLSSSPSSPATGDVQISDVDKHGL
YVRLLNTSKTDCHIGNFVIKHQVNEISYKFNTKAILKGNCVTTVMAADSGQSHKPPANYL
WKQQNNFGLEEMVTRLVNAGGEEIAIFNLTK
>brachi

```
LSPTRLTRMQEKQELQWLNDRLAQYIDRVRYLEAENSRLMVQVTSSEEITQREVTNIKSM
FEQELTDARKLLDDTAKEKARVQIEAGKYRAEADELRAKLAKSEGALATAEKKRHQAESA
LNEKEGRLQNAIADKQRAEGELAALRLEFANLEKQLATARKQLEEYEQELTESRTR---K
EVSITEVDAG-EAAFESRLQEALREMREQHELEARSMREELETMYTQKITSMRTDLSVAR
EEMRESRARIDSLMSQVSGLQGQNASLEARCKELEGMMARMSEESRSSAEQYEREIRQLR
EEISQMMVDYQELMDIKIALDLEISAYRTLLEGEEQRLKLTPTPSSSGAVAVGGVDLEGK
FVKLQNTSEKDMSMGGWLLKRTVGEISYKFPSRYVLKAGQSVTVWATEGGGTHSPPSDLL
FRGQASWGSDDTKTLLVNDSGEEMATRSVTR
>priapul
LSPARITRLQEKEDMINLNDRLANYIDKVRNLETENSQLSIQVGESEETITREITNVKAL
YERELADARKLLDETSREKAKLLIDCNKYQSENDELRTKLARLTRDLTGAQRSLTTAETQ
VPDLTIKYDTANKDRKKAEDELRQLKKDMADFQTQLSAARSELENYENEMKEVRAK---K
ELAVTEVDSRLQEEYEHKLADALRELRDQYEEQSAIHRDEIEQMYESKLADLKHVARLAR
AQIKEMRSEYDSLKAQAESFESERAALEARMRGLERALDREREEHRDQMSQRDDEIKNLY
AQIAQNLQEYQDLMDIKIALDMEIAAYRKMLEGEEARLNIDTPPSSKGEVRVMECDPDGK
FVKLSNTSDKDIHIGTWQLKRITGEITYKFHRNIILKAGQHINVWSSDVKVTHNPPSDLV
MKNQ-KWYSESMKTILIDSEGEEVGERENFR
>drosop
LSPTRHSRVAEKVELQNLNDRLATYIDRVRNLETENSRLTIEVQTTRDTVTRETTNIKNI
FEAELLETRRLLDDTARDRARAEIDIKRLWEENEELKNKLDKKTKECTTAEGNVRMYESR
ANELNNKYNQANADRKKLNEDLNEALKELERLRKQFEETRKNLEQHSQEINESRRI---K
QTEYSEIDGRLSSEYDAKLKQSLQELRAQYEEQMQINRDEIQSLYEDKIQRLQEATHKSI
EELRSTRVRIDALNANINELEQANADLNARIRDLERQLDNDRERHGQEIDLLEKELIRLR
EEMTQQLKEYQDLMDIKVSLDLEIAAYDKLLVGEEARLNITPATNAKGNVEIKEIDPEGK
FVRLFNKGSEEVAIGGWQLQRLINSTTYKFHRSVRIEPNGVITVWSADTKASHEPPSSLV
MKSQ-KWVSDNTRTILLNSEGEAVANLDRIK
>nematos
PGSAKFTRAQEKAELQHLNDRLATYIDRVKNLEQENSKLRSEVTVSRKTVEREVDSMKSL
YETELADARRLLDETAKEKAKQQIESSKNSNDAQEFKNKFDKEAAARKKAEKELNDVRKL
LHDKENQLTRRNQEALNLESVLRELQGECEELKDALKAAKYALEQYDKELSDIRSQLKTV
ETKRVVVETDYKDKYEGLMAEKLQELREDYDSDARSFKEETELLYSSKFEELRIQLAKLR
EENRNLSKSVDELSSQVHQLEAKNNALVSRVSDLQGLRAQDKEKHDNEILLRENEIAELR
TSIDDALRDYEDLMGVKVALDMEITAYRKLLESEETRLEITPPASAEGAIQFTEADPDGK
YIKIYNSGEKDEALGGWTIQRQVTPSVYKFTPKYVLKSQSHVTVWSAQGGGTHKPPSDLV
FKQLPSWGSNEARTALVNAGGEEMATLLEEK
>amphimed
PTPERRTRMKEKEDLQLLNDRFVTYINRVRRLRDEKEKLNVTLEHMQTTTQEESGAIKRM
FEKELQDARLLIDETAKEKARYQIQSSKTEDRIKDLETELKEFREDYDRMQQELAEAKKS
AETFESLYKSSLVDNQSAKQKIQDLEEEIDDLRARLREVQDSLEQYDEELLTIKRT--GL
TTVHRDGVEETDDDFAERLQAAIDEAREEIEKETEQFKLDLEKSYKTRLESLESQRVRLD
GELKNCHTTLAKYNRDIAKLQEKNEQLESSLREKSEDLARAKQLHSAELANLMDEMRALR
VNYDQKMKEYEELFDLRIQLEQEIATLSALLQEEEQRLNLQTTPRAVGFIQIIDVDPDGR
FVQIKNMSDKVTEIPKNKLQLVGVLIASKYEEMYAPEVADFVYITDSTYSTEIKAMERNI
LKTL-DYSFNPLCLHFLSRAGDATPQMHTMA
>daphnia
LSPTRLTRLQEKVELQNLNDRLANYIDRVRQLESENNRLTLQITTTQDTISREVTSVRGS
YEKELSDTRQLLDETAKEKARLQLEAGRARAELGELTPKYNKAKNDLLQAEKKVQQLETR
NNELNASLHQLKSDMARAIEEKKDAVSERDKLAKQVPELQQQLETYEREMTEVRSN---K
QVEISEIDGRLQEQYQARLQSTLQELREQYESQIQQSRDEVEVLYQNKVEDLEDQAGAHY
EELIQARNRLKDANLKLSELELANSHMNDRLAEMERNMETERQTHATAMSMCQGEITRLR
EQMAIQLQEYQDLMDIRTALDMEISAYRKLLEGEETRLKLTPTAGAKGEVQVEEVDSEGK
FIRLINKSEKEVSLSGWQLVHKAQETIYKFHRSLKVAPGGTVSVWSAGSGTAHEPPSTLV
MKEQ-RWFVNEMVTQLLDNSGEEMAQRESKR
>hymenol
PSPLNISRVDEKEELAHLNDRLASYIDYVRKLERDKESLTRRISTITEERLAKVDNARST
YENEIQSLRRLVDDLAKEKAARDVELKKHMDDASDAKSKLNKRDVELRNLQRKYDTLERD
```

```
IGTY-----------KSDHERYQKLLPEYEHLEKSLEAAKEGLKAFEEERSKLVMR---S
LTVEEEVAETKAKEYESRLADELQAYRESANEELEQYRLQMESTFQNKLEQLQKAGGRLN
EELIAMRRMNDDLSHELARKSREVELLHSRIADLEKLLNKAHDDYQAQLAAEREEVKRLR
AELEQRFAEFTDLMNTKIVLDQEILMYRKMLEGEESRLNLTSPKRAVGSIEFSDQDGSGN
WIKLSNVGKEDINIGNWVLKHKSDEVTYKFGRNVILKPGTPCTVWSVDAGATHDPPEDIV
MKNQ-RFHAANTTVTLTNENEEEQASCLIVR
>aplysia
PSPARLSRIQEQEELQGLNDRLAAYIDKVRSLEAENSRLALQVRSTEEVVKREVTSVKTL
YESELAELRRLLDETSKEKARLQIEANKYKTDYEDLLAKYNKRDRDANALERRVQVLERQ
LAEFSAR---------DFEKENQNLKNELNRLEQQLAAAKKQLEEYEQELEESRVR---T
TTQLEEVDGRIEQEYEARLQDALREIRAQHDYDLQSVKVELETLYESKVEDLRNQSSAAW
EELMLARRSVDDLSAEIGRLKSENAGLDLRVKDIDKQLERERDEFRLRMKQRDEEISRLR
AQVEELEREYAALLEIKIKLDREIEAYRKLLESEETRLNISASMTSSCGVDISDVDTDGK
FVKLTNTTDKDVHLFHWQIRHVSGETRHKFQKN-VLKAGQTITVWSADSDQTHNPPSDLV
MKGK-RWFVGDMTTTLLDQDETEVASCSMSR
>laminB1
LSPTRLSRLQEKEELRELNDRLAVYIDKVRSLETENSALQLQVTEREEVRGRELTGLKAL
YETELADARRALDDTARERAKLQIELGKCKAEHDQLLLNYAKKESDLNGAQIKLREYEAA
LNSKDAALATALGDKKSLEGDLEDLKDQIAQLEASLAAAKKQLADYEEEINETRRK---H
ETRLVEVDSGRQIEYEYKLAQALHEMREQHDAQVRLYKEELEQTYHAKLENARLSVNSAR
EELMESRMRIESLSSQLSNLQKESRACLERIQELEDLLAKEKDNSRRMLTDKEREMAEIR
DQMQQQLNDYEQLLDVKLALDMEISAYRKLLEGEEERLKLSPSPSATGNVCIEEIDVDGK
FIRLKNTSEQDQPMGGWEMIRKIGSVSYKYTSRYVLKAGQTVTIWAANAGVTASPPTDLI
WKNQNSWGTEDVKVILKNSQGEEVAQRSTVF
>salpin
GSPQFRRRVKEREQLDELNNELAAYLVRVRELQKENGELTSQLEAIRFSRKRFESTATIK
LEGQVNDLLEEIRRKTTEIGTLNHQVQDLRKQLNKTEGDALEATREKDDLAKQLNEALSK
VQELEGQVSGLQRDVSQRDTTVTNLQRRVASLEADLGRQVDDTKRYQQQVTALEEQIK--
------IGGTSGGLSEEDLEAALTEAKEHYKQAVNNFRTQMRSYFAQQ--PIIEPRARLQ
TEVIEWRSKYEDVKAQGATAQTEIEGLKSKLAGLEDVFAQIKKSHTDEIRHKEDFIFKLT
KTMQDKDDLYRDLLDERIALDAEIDRYRRQLESALRMFEARSPGTTQGALSVEDVDREGQ
YILLKNTTASPLDLADWAVTVTNRLGTFEFP-AVTLAPGRSTRVIRGAGDGAEKLEGDVV
WTEFPDLAAPELAVYAFDPDDNESSTWAIMR
>salpin2
AIAAVRNTMKEREEMEKLNDNLEVYIRRTSALYSSHSTLVSLQASRRTSVVASTPTASPS
KSNE--------ERLRKEVKRQQAEIDRLQSLASQLEKQFENAVEVKDTALNQLEECAAT
QQDLRTEL---EKQKEAYDARLASTEATISSMTAELEESRARAHAHETTVTTLEEE---L
QACTKAMA--SKQAPDTTIDAAVAEVKKEFQAAANAYRHKLRQYYATHARGPQAMRMALK
QQVFTLTKEKADLSTTNASLHATVDACKQEVAAIRQSMKDAVHHHTTEIKAKEERIAALT
AHIRANEKKYADLLDERLWLDAEIDEYRRLLNSIQRRLSLPAAPRGPGRLELRDVDTKVE
CLLVANPSTSSVDIDGWALTASADLISTLEFSAHVLPPGGVLRVFQQRNEENRDCLWDEL
TRPR-EWGDQKXXXXXXXXXXXXXXXXXXXX
>monos
PSPKRRTRELERQRLEDLNHALSRYISRVRALETQVVTLETHIESFASVKAPKNDEVLET
LRAQLEQAKAELDDASQRYADLQLEHSNLQEDFKRAEGDIQGLTSLKERLQGRIESLQAM
VNRLEGQLASAQSSLQEEKEDGDLARQQLKDLKADLVKHRGRADSHQSETDALQQQLQVA
LSQVVVSVKTTGNQYEEEMSRLLRVAKSSYAEAADAFREKLKKYYQMNPTPSAAPLKRLQ
AENEALRNRVAARDQDVADLKNEARHAEDKIDRLTHDLASTKAENKAHLANKDMEIKNLQ
QRISGLEDQYRVLLDRNLSLDAEIDQYRGLLGEEYKRLNMHDQSLSNGQIQIQSVDDRGF
DLHLQNIGQEPINFDDCVLTVDNGSETLVMPHGHVVAVGQVVRVVSGLGEQSHR-PGDIA
WTSFRDFEFNTINVQLE--RGSQVICRAEYT
>capsa
SSPDRAARLEEKEELQFLNSKLELYIHRRQELQTLVKTLESELERERNDAQQRLEIAHRQ
YEATLTETRHALDKAASEASHASSSLVQVNAELAATKERARTESSAADALRSRYEALQLA
KHELDGRLAVSEIAIKELKDNLKHADSDRKHAEKESDALRKSSEKGADLNAYHSEEMRRL
RSDLSSLERKLRAEHDQELAEALSEHRRQTEAESAQYRAEVQALYESRLEAAREQVAGLQ
```

QALSAAQAAQDRARAEVTALSQRSETLSTRVRTLEHELIIERDTVVEVQRLREEATRLMQ
EAIAAKDSEVRTLTETNVALDKEIAMYRTLLEVEEKRLGEDFTPTEAGLVVFEPVNLVHN
KLVLRSTNEEEVSLHGWKVRTTSESATYEIPAGIALSAEPTVTIWTGVSAGKRKSRSELL
WKQDINYASNGACIQLLDNEDNEIARLEVRE
>creol
LSPMVAKRLEDQIRLTQLNSKLKEVIEKMENAQPTNIEIESTCARLKEDEDNSLNRELER
THAKMADAQDRKKLLTKEKANIEKKDAEAFKNKTKRETEAKALHKSAEKLRKQTKDLQSK
IDDCTKQVGNIRDELAQANQALEQLEASVAAFVDTKRTLGEKSAENKEALQHLRAR----
---IAGVAAKKERELQTKAAEAEADVVGVFNRALQKHVNDIKVLHEKKLETAIQTDVEDK
AEVKRLDGKIKDLKKLRKELEDSAQKQKAIIAELEKQLENMKKKHAVEM---KAQTQQVM
AEKEIVQLAYLQAEKAQKLLGDQINEYEKLLDFEER---------ITGIVELVAVDPQGL
YVTLVNETEDEIHLDDYHLIGVSHTRRFDFSSDCRLDARGKVTVYTNAEEYERSIGNAQL
WETDPVWAAEGPSQMKLYNSGGLVSAATISV
>sphaero
SSPLVKQRKTAKRKLEALNAQLANVIHRMEHAEPTEXXXXXXXXXXXXXXXXXXXXXXXX
XXXXXXXXXXXXXXXXXXXXXXXXXXXXXXXXXXXXXXXXXXXXXXXXXXXXXXXXXXXX
XXXXXXXXXXXXXXXXXXXXXXXXXXXXXXXXXXXXXXXXXXXXXXXXXXXXXXXXXXXX
XXXXXXXXXXXXXXXXXXXXXXXXXXXXXXXXXXXXXXXXXXXXXXXXXXXXXXXXXXXX
XXXXXXXXXXXXXXXXXXXXXXXXXXXXXXXXXXXXXXXXXXXXXXXXXXXXXXXXXXXX
XXXXXXXXXXXXXXXXXXXXXXTIEEYARLLDYSDAELDASFQPTITGVVSLKEVDPKGT
KVLLENQTEEVVDLSGWHLVGVSHTARYDFDKDATLNARCSVLVFSYG--YGYDIPXXXX
WKTDPVWSAEGPSTMKLYHEGGLVSAATISV
>creol2
MEEVEKLRTDNKVQIDVLHEKLNTIVSQCDALETENAALVAQTEAIEVEKVKLMDDIESL
HEVH-ADLQDLLKRKRQECKELESETGKAIKKKKRLEETERKTA----------------
-------------------QQCDSLTSNNAEKDKVLADLRNEADVMKREISDHENVITNL
TIELQKTEDAAKAEYAQKFKKIASEKRDEMKALLEEKKKQRTAELEAELQSLKKSVNKAQ
AEVDELSTIIKEKEASLPDVE----HLKQAAEHIKADIKMWKERIDTLKKKTEEEIAELT
NEKLGHDEQMTQLDRYGITLEEQLNNYRDLLHVAAEGDGVDP---HIPVYSLDSINPNTS
SVGVRNNSITDVDLAGWTLTIEYTSIKYTFGDNTMIKPEAIYTVTTDPDEVTHN----AV
WIKVDVWGYNNVKIVLKDTSGELQCSAVVSA
>sphaero2
XXXXXXXXXXXXXXXXXXXXXXXXXXXXXXXXXXXXXXXXXXXXXXXXXXXXXXXXXXXX
XXXXXXXXXXXXXXXXXXXXXLEKETGKLFKKKKKFEDTERKTA----------------
-------------------QKCDDLTVEIKDLDGLLVQRQQEADQMKEAFKAHEATVNAL
KEELXXXXXXXXXXXXXXXXXXXXXXXXXXXXXXXXXXXXXXXXXXXXXXXXXXXXXXXX
XXXXXXXXXXXXXXXXXXXXXXXXXXXXXXXXXXXXXXXXXXXXXXXXXXXXXXXXXXXX
XXXXXXXXXXXXXXXXXVDLDEQLNDYQNLLHVKPSADGLAP---TTPSLYIDHIRPAQS
SLGVRNNTERDVDLHSWMIRVRYTHITYTFSGPAIVSPDQLYTLTTDEHESTHD----EV
WESLDVWAYRTVYLELLDADKLLVSTAVVXX
>pirum1
LSPASQERKAEFERLCNLNGRLKYYIRMVREKYPEENQHQREMASQKDRSDNEKQEMKEH
FNGKIQNLEE--------------EVARHSASNRESLKKIDQLHREKANFEKAIENLQTD
LVKKENHINQLKEEKKTSSVLVQELEEELSELKKELADAQDEKAGLTEELEETKSN----
---LENNEQELNSAYEERLEIALAEAKKVHQQNADKMLDDVERSYEKIFAEKEADTQGLQ
KENDELKEQKETILQEGEEIK-------------------------NENNELQQSVVVLK
AKIDELEKQYNNVSKSKANLTREIDSINKLLTEEEARLERDIAKESSGDVIIKEVDAEGF
FIRLQNVTSAPIQMDQWLLRSYYEAIDFNFSKDLVLEPEQYLTIITRLDEEEIQPDSEVQ
WDSEPLWNNEGGFVELFNQEGASVSAAQIEL
>pirum2
XXXXXXXXXXXXXXXXXXXXXXXXXXXXXXXXXXXXXXXXXXXXXXXXXXXXELQNGREV
LESKIEDLKV------------------------TLSSKTQKFTEEKQNLQDSLDNAERE
LNEIKTI--------------------------------------YEEKLEKTRQD----
----------LAEEYNARLEDALLNARDQHERENEEFELRLNNEHQEELSELNVKLKQSI
AKLKQVNDTNDELRRELSQKEAEKKELENQLVDVKKSFEQLQADYEEQVNENDSHRRLLD
-----------EVAEENSDLRTEIDYTFKMIEQEEARMGMKFDPKHSTTFTLDEVDPEGN

FFYLKNNSKKPVDLSTWVLRASSAYVDFHFPDNITLKGSEKVAVLTSQNTEESDMKLDLF
WTVTSIWANDKGSVNLFK--GELCSAATITE
>abeofor1
LSP---SLAKEQKRLADLNDRLSDLFSGLRGKR---------------------DDIISG
YERKIQDLNKQLLEAGEVLHNLEADIAERDAAIQKLEIVKQNLEASREDLQKVYESKERQ
CEDLHREIQLQVEKLSEEKAKNKEMTKELKEVNKQLNSASQKLEKLNAQIEKLEHD---L
KEKDEENAEEAECDFDVRMQLALQEARQLFETDLESQLEAVESKREAEMKNLKTQDESHA
KEKKKLEDQLEQLKANFEKLEDEKEKMEDQLLQMKKELEKERKQSEKNLKMDEEYQREKE
TQKAANDLAYDELKAEYENLVQTITDVTNLLELEEERLGIPITPSYGHQINFDEIKTNGQ
YIKIKNESEENVSLTGWSLKCTFEEITFTF-DDYELASEDSVAIVTSSDYMSNIKGNKIL
FETEPFWAVDNGVVELYDDRGDVICSAVVEE
>abeofor2
ATPVRKKDSGQNARLQNLNGRLKYYINLVRQKHPEE-----------------MDKVKNE
YEVKMKDLQGQINALQDEISELNSLNVLQEKELKEKNASLRQKD----DLMNKQEQFTEG
LEQS---IADLQVANKVLTNKERDLKAKVSALKEDYDELCEANEAHAIEMKLSHDQ----
--------AEKERLASEKHSNEMREMSEEFFEKERNLKNELRQVEKIKIESEKATFEQKE
NELKEVTHKLNKLADSKKTLEDENAKLLQKIKDLELNSEDLYK-----------KLTKVE
TKLENKTRKYH---DSSQTILEQLDIYNNLLLEEEKRIEGLPKFSSTGSIVLEEIDATGA
FIRIFNNSKHPVCLSGWNILVKNPIGQFCFPDDFTLAASGKVSVTSPSFNNDDDTEVHLQ
WNEPSIWSSDGCTIQLFDMNGIIASTGKVEE
>hypoch
XXXXXXXXXXXXXXXXXLNNRLEAYVLRVKEMDDARSHLDHELKTISTRYQKEMDALRER
LSRDVDTLRSELEYEIDQKTVHQVKAEDLTLQVEKMTAQVRITSKLNLEEE---EERERE
MDREKESCAFKREXXXXXXXXXXXXXXXXXXXXXXXXXXXXXXXXXXXXXXXXXXXXXXX
XXXXXXXXXXXXXXXXXXXXXXXXXXXXXXXXXXXXXXXXXXXXXXXXXXXXXXXXXXXX
XXXXXXXXXXXXXXXXXXXXXXXXXXXXXXXXXXXXXXREEAKRYKDEIRRLEKEVSALR
DSQAESEKQFNDLMDVKIGLDNEIRHFKAILDMEEDRVGVFSPERAVPDVVVQMIDTENE
CVVIKNNSSEAKDLSHWTLSNKAGTQKFEFPEGFSIPADSSVRIWNGKKNSKHSPPENIY
WSSKFVWNADGEAALLLSPSGDLAASSAGGE
>phytcap
LSPIKSKRMEEKASLQKLNSRLEMYVLGVNELESAKHAAERELETIKQRMQQDLESVRIR
LTKELEETRKKLDDEVDQNNRLQTLEQEQNRELVKLRAQLKEFGEL----KVLVETLKVE
RDREKTNAESAKQALSEQTTQLNSARRSVKELERDLRSHKAALTDATQELQDLRKKCANY
DMAKDSELTKLRREWAAKHLEAQAIWKKDAEERLATMEMEVRSHFNSMSTSLESQLDDVR
TELESTKKELDRTANDYEDSLKARQSLTEKVAQLERDYREARSKSTKDRKAYEETLERFR
SSKVAKEREFNELMDVKIALDAEIMKYRRILDREESRVAITTPNTPSSAVQIASLDLEKD
RIVIQNTSNEPVSLGGWAVRGQMD-QTFRFPKTYVMRPRSTLTVLSSKRNHESKKGEAAF
LATQFSLNPNGDIVFLVTDDDIPVSMMSEGL
>phytpar
LSPIKSKRMEEKASLQKLNSRLEMYVLGVNELEGAKHAAERELETIRQRMQLELESVRSR
LTKELEETRKKLNDEIDQNAKLQTLEQEQHKELVKLRAQQTELGEA----KVLVETLRVQ
LDREKANAVSAKETLSEQTTQLNLARRHVKELERELRGHSAALGDATKELEELRKKSVDF
DLTRDAELTKLRREWTAKHLEAQAQWKKDAEDRLQSMEMEVRSHFESVSGSLQTQLDDVR
TELESTKTELDRTANDYEESLKARQSLTEKVAQLEREYREVRSKATKDRKAYEETLERFR
SSKVAKEREFNELMDVKIALDAEIMKYRRILDREESRVAVATPNTSASAVQIASMNLEKD
RIVIKNSSSDPVPLGGWVVRAQMD-QIFRFPATYVMRPQSTLTVHSSKRNNERKKGEDSF
LANKFTLNPKGDFVVLVNSDDIPVSMMSEGL
>phytcin
LSPIKSKRMEEKASLQKLNSRLEMYVLGVNELEGAKHAAERELETIKQRMQQDLDSVRAR
LTKELEETRKKLDDEMDQNARLQTLEQEQHKELVKLRAQQVELGEV----KVLVETLRVQ
LDREKANADSAKETLSEQTTQLNVARRRVKDLEREVRGHVAALGDATQELQELRKKSADF
DLTRDAELSKLRREWTAKHLDAQAQWKKEAEDRLLSMEMEVRNHFETVSGSLQGQLDDVR
AELDSTKKELDRTANDYEESLKARQSLTEKVAQLEREYREVRSKSTKDRKAYEETLERFR
SSKVAKEREFNELMDVKIALDAEIMKYRRILDREESRVAVSTPNTSSSAVQIAALDLAKD
RIVLKNSSSDKVPLGGWEVRGQMD-QTFRFPPTYLMRPQSTLTVHSNKRNHERKKGEDSF
LANKFSLNPKGDCVILITSDGIPVSMKSEGL

```
>phytinf
LSPIKSKRMEEKASLQKLNSRLEMYVLGVNELEGAKHAAERELETIKQRMQQDLESVRSR
LTKELEETRKKLNDEIDQNAKLQTLEQEQHKELVKLRAQQTELGEA----KVLVETLRVQ
LDREKANATSAKETLSEQTTQLNLARRRVKELERDLRSHAALLSDATKELEELRRKSVDF
DLTRDAEMTKLRREWTAKHLDAQAQWKKDAEDRLQSMEMEVRSHFDSVSGSLQTQLDDVR
AELDSSKMELDRTANDYEESLKARQSLTEKVAQLEREYREVRSKSTKDRKAYEETLERFR
SSKVAKEREFNELMDVKIALDAEIMKYRRILDREESRVAVATPNTSASAVQIAAINLDKD
RLVIKNSSSDPVPLGGWVVRGQMD-QTFRFPATYVMRPQSTLTVHSSKRNNERVKGEDSF
LANKFSLNPKGDFVVLWTADDIPVSMKSQGL
>phytso
LSPIKSKRMEEKASLQKLNSRLEMYVLGVNELEGAKHAAEKELETIKQRMQQDLDSVRTR
LTKELEETRKKLDDEMDQNARLQTLEQEQHKELVKLRAQQVELGEV----KVLVETLRVQ
LDREKANADSAKETLSEQTTQLNSARRRVKELEREMRGHVAALSDATQELEELRKKSADF
DLTRDAELSKLRREWTAKHLEAQAQWKKEAEDRQRTMEMEIRSHYDTVSGSLQSQLDDVN
AELESTKKELDRTANDYEESLNARQSLTEKVAQLEREYREVRSKSTKDRKAYEETLERFR
SSKVAKEREFNELMDVKIALDAEIMKYRRILDREESRVTVSTPNTSSSAVQIAALDLVKD
RIVLKNSSSDAIPLGGWIVRGQME-QTFRFPPTYVMRPQSTLTVHSSKRNNERKKGEDSF
LANKFSLNPKGDFVVLLSSEDIPVSMKSAGL
>phytra
LSPIKSKRMEEKTSLQKLNSRLEMYVLGVNELEGAKHAAERELETIKQRMQQDMESVRSR
LTKELEETRKKLDDEMDQNARLQTLEQEQHKELVKLRAQQTELGEA----KVLVETLRIQ
LDREKANAESAKETLSEQTTQLNSARRRVKELERDMRGHVAALSDATQELEELRKKSADF
DLTRDAEMTKTRREWATKHLEAQALWKKDAEDRLHTMEMEVRSHFDNVVTSLQNQLDDVR
TELDSTKKELDRTANDYEDSLKARQSLTEKVAQLERDYRVERAKSTKDRKAYEETLERFR
SSKVSKEREFNELMDVKIALDAEIMKYRRILDREESRVALATPKTSSSSVQIASLDLEKD
RIVLKNSSSEPVPLGGWVVRGQMD-QTFRFPATYVMRPQSTLTVHSSKRNNERKKGEDSF
LANKFSLNPDGDFVILVTSDDVPVSMESEGL
>phytlat
LSPIKSKRMEEKASLQKLNSRLEMYVLGVNELEGAKHAAERELETIKQRMQQDVESVRSR
LTKELEETRKKLDDEMDQNARLQTLEQEQHKELVKLRAQQTELGEV----KVLVETLRVQ
LDREKANADSAKETLSEQTTQLNSARRRVKELERDMRGHVAALSDATQELEELRKKSADF
DLTRDAEMTKTRREWAAKHLEAQALWKKDAEDRLHTMEMEVRSHFDNVVTSLQTQLDDVR
TELDSTKKELDRTANDYEDSLKARQSLTEKVAQLERDYRVERAKSTKDRKAYEETLERFR
SSKVSKEREFNELMDVKIALDAEIMKYRRILDREESRVALATPKTSSSSVQIASLDLEKD
RIVLKNTSSEPVPLGGWVVRGQMD-QTFRFPATYVMRPQSTLTVHSSKRNNERKKGEDSF
LANKFSLNPNGDFVILVTSDDVPVSMESEGL
>hyalop
LSPIKSKRMEEKASLQKLNSRLEMYVLGVNELEGAKHAAERELAIIKQRMQQDLERVRSR
LTKELEDTRRKLNNEMDQNARLQTLEQEQHKELVKLRAQQTEWSEV----KVMLETLKAQ
LIGEKANAKAAKETLSEQTTLLNAAQRRVKELERETRSQSVALSDATQELEELKKKSADF
DLTRDVELTKLRREWTAKQLESQAQMKKDAEDRLLTMETEVRSHFESVVSSLQNQLDDVK
SELESTKQELDRTANDYEESLKARQALTEKVAQLERDYREVRSKATKDRKAYEETLERFR
SSKVSKEREFNELMDVKIALDAEIMKYRRILDREESRVAGATPNSSASAVEIASLDLEKD
RIVLRNTSSKPVPLGGWVVRGLMD-QTFRFPATYVMRPHSTLTVHSSKRNDERKKGEDSF
LANKFSLNQKGDFIVLVTSDDVPVSMKSEGL
>pseucub
LSPIKSKRMEEKASLQKLNSRLEMYVLGVNELEGAKHAAERELETIKQRMQQDLENMRSR
LTKELEETRKKLDDEMDQNARLQTLEQEQHKELVKLRAQQVELGEA----KVLIESLRVQ
LDREKANAESAKETLSEQTVQLNSARRRVKVLERDMRGNAAVLNDATQELEELRKKCADF
DLARDAELSKLRREWTAKHLETQAQWKKDAEDRLQTMEMEVRSHFESVSTSLQSQFDDVK
AELESTKKELDRTASDYEESLKARQSLTEKVAQLERDYREVRTKATKDRKAYEETLERFR
SSKVSKEREFNELMDVKIALDAEIMKYRRILDREESRVAVATPNTPVSAVRIDSLNLEKD
RIVLKNTSSEPVPLGGWIVHGQMD-QIFRFPATYVMRPQSTLTVHSSKRNNERKKGEDSF
LANKFSLNPKGDFVVLLTSDDIPVSMKSEGL
>pythult
LSPIKSKRLDEKSTLQFLNGRLELYVLRVKEMEDAKNVAERELETIRQRMQADIDALRLR
```

MSKELDETRKKLDVELDQKARLQVLEQEQHVELVKLRAQVKELSEI----KALAESLQVE
LTKERSNSKASKETLSVQTTQLQSARRRIKDLERENRSYEASLSDTTKELDELRKKSSDF
DLTRDLEITSIRKEMNQKHQEALAQWKKDTEERVLGVEKEVRLYFEGVVQGFKSQVEDLS
VELDSTKKELDRTASDYEESLQVRQQLTEKVAHLEREYREERTKFKEDRKKYESTLEHFR
SSKFAKEQEFNDLMDVKIALDAEITAYRRILDREETRFGLPTPTNAPSSVQIVHLDLEKD
RIVLENSSDKPVSLAGWEVRGRAENQVFRFPATYSMRPKSKMTVYSSKRNDERKPNEDSF
LATKFSLNASGDFAVLLTADGVPVSTRAEGL
>pytharr
LSPIKSKRLDEKTSLQTLNSRLELYVMRVKEMEDAKNVGERELETIRERMQKDIDNVRSR
LSHELEETRKKLDVELDQKARLQVLEQEQHVELVKLRAQVKELSEI----KALAESLKAE
LSKEKANAQLSKEELSTLTTQLQAARRRIKDLERENRAHEASLNDATQELEHLRKKSADF
DLTRDAEISNIRKEMSLKHTEALAQWKKDTEDRLKGVEKEVRNYFESVVSGLKNQVEDLS
SELDSTKKELDRTANDYEESLKVRQSLTDKVAQLEREYREDRAKFKEDRKVYETNLEKLR
SWKVTKEGEFNDLMDVKIALDAEIETYRRILDREETRVGLPTPHTAPTAIHIAHLDLEKD
RIVVENTSDQAVSLGGWEVRGRAENQVFRFPSTYSMRPKSKMTVYSSKRNDERKPNEDSF
LATKFSLNQSGDFAVLLTNEGVPVSMKGEGL
>pythirr
LSPIKSKRLDEKSTLQLLNGRLELYVMRVKEMEDAKNVAERELETIRQRMQADIDAIRLR
LSRELEETRKKLDIELDQKARLQVLEQEQHVELVKLRSQVKELSEI----KALAESLRVE
LTKEQANSKSSKETLSVQTTQLQSARRRIKDLERDNRSHEASLNDVTKELEDLRKKSADF
DLTRDSEITTIRREMNQKHQEALAQWKKDTEERLQAVEKEVRHYFEGVVQGFKNQVEDLS
LELDSTKSELDRTANDYEESLKVRQSLTEKVAHLEREYREERTKFKEDRKKYESTLEHFR
SSKFAKEQEFNDLMDVKIALDAEITTYRRILDREETRVGLPTPTSAPSSVQIVHLDLEKD
RIVLENTSDKAVSLAGWEVRGRSENQVFRFPSTYSMRPKSKMTVYSSKRNEERKPNEDSF
LATKFSLNAGGDFAVLLTAEGVPVSTRAEGL
>pythiwa
LSPIKSKRLDEKSTLQLLNGRLELYVMRVKEMEDAKNVAERELETIRQRMQADIDAIRLR
LSKELDETRKKLDIELDQKARLQVLEQEQHVELVKLRSQVKELSEI----KALAESLQIE
LTKERSNSKSSKETLSVQTTQLQSARRRIKDLERDNRSHEASLNDVTKELEDLRKKSADF
DLTRDSEITTIRKEMNQKHQEALAQWKKDTEERLQAVEKEVRHYFEGVVAGYKNQVEDLG
QELDSTKKELDRTANDYEESLQVRQKLTEKVALLEVEYREERNKFKEDRKRYESTMESFR
SSKFAKEQEFNDLMDVKIALDAEITTYRRILDREEVRVGLPTPTSAPNTVHIVHLDLEKD
RIELENTSDKPVSLAGWEVRGRSENQVFRFPSTYSMGPKSKMTVYSSKRNEERKPNEDSF
LATKFSLNASGDFAVLLTADGVPVSTRAEGL
>pythvex
LSPIKSKRMDEKTSLQTLNSRLELYVLRVKEMEDAKNVAERELETIRQRMQLDIDNIRTR
LTKELEETRKNLSDELEQNARLQVLEQEQHAELVKLRAQMKELSEV----KALAETLRLE
LTREQSNAKASKETLSEQTTQLQLARRRLKELERENRSQSAHLNDTSMELEELRKKSASF
DLTRDGEIANIRKEMNAKHQEALAQWKKETEERLLGVESETRSYFDNVVTGYKSQVEDLS
SELESTKAELDRTATDYEESLKVRQQLTEKVAQLEREYREDRTKFKEDRKQFEITIERAR
SSKFAKEQEFNELMDVKIALDAEITAYRRILDREESRVGLPTPNTPSSSVQIVRLDLEKD
SVYIENTGDKPVALGGWEVRGQLEGQLFRIPSTYTIRPKTTCVVISSMRNKERKKGEDSF
LATKFSLNPKGDLAVLTTPDGVPVSLCSEGM
>albugo
FSPIKSKRLDEKASLQSLNSRLEIYVMRVKEMEDAKTVAERELETIRDRMQQDMDVVRLR
LTKELDDTRKKLDHELDQKTRLQILEQEQHVELVKLRAQVKELGDL----KTLTEQLKSE
LSKERSNATAAKEELSRQTTELQAVRRRIKDLERERRGMDAALSDATTELEHLRKKSATF
DLVRDTELSNIRKEMNAKHQEALANWKKDTEDRIHAVEKEVRNYFEGVVTTSKSQAEELS
LELDSTKKELDRTANDYEESLQVRQSLADKVAQLERDYREERKKFKDDRKMYETMVEKLR
YAKLSKEEEFNDLMDVKIALDAEITAYRRILDREETRVGLPTPKTNDDPIRIAQLNLEKD
FVVFENQGDKPVSLGNWEVRGKLETQVFRFPSNYVVKPHARVTVFSAKRNADVKPDEDAF
MTKKFSWNHSGDWAVLYDDEGVPVSSLAQGL
>albcan
FSPIKSKRLDEKASLQSLNSRLELYVLRVKEMEDAKTVAERELETIRDRMQQDMDVVRMR
LTKEVEDTRKKLDHELDQKTRLQILEQEQHVELVKLRAQVKELGDL----KTLTEQLKSE
LSKERSNATAAKEELSRQTTDLQAVRRRIKDLERERRGLDSALSDATTELEHLRKKSATF

DLVRDTEISSIRKEMNAKHQESLANWKKDTEERIHAVEKEVRNYFEGVVQSFKSQVEELS
LELDSTKKELDRTANDYEESLQVRQSLADKVAQLEREYREERKKYKDDRKVYETTVEKLR
YAKQSKEEEFNDLMDVKIALDAEITAYRRILDREETRVGLPTPKTNDGPIRIAQLNLERD
FVVFENQSEKPVALGNWEVRGKLETQVFRFPSTYVIKPHARVTVFSAKRNADVKPDEDAF
MTKKFSWNHSGDWAVLYDSDGSPVSSLAQGL
>sapdic
FSPMKSKRLDEKTTLQALNNRLEMYVLRVKEVEDSKEIAEKELESIRDRMQLDLTMTKTR
LSKELEEVRKKYEEEREAKIRVQNLEKEQYNELVQLREQVKELNNT----KTYLETVQSD
LAKEKESCKAAKEALASNATALQSERRKLSDMEKEYRKTLASLHDATSELEQLKQKTSEF
SMTRDSEMTALRKEMNAKHSEALAAWRRESEERQQVVENEVRSHFEGVVEGLRSQLEEAT
KETTALRTDYERTANDYDESLKLRQSLTEKLAVVESQYRADRKKFQEDRRVYESNIEAAR
KARKEKDLEFNDLMDIKIALDAEISAYRSILDREETRFGVEPKASASGDFRIRQLNLDEG
RIILENTGATPLPLNGWQITSKSSNHVFSFPDDYVIQPGGKVSVLSGK-NEGEQDLMDFY
AVKKAIWNPKGDVALVKNAEGEVVCSHAEGL
>sappar
FSPMKSKRLDEKTTLQALNNRLEMYVLRVKEVEDSKEIAEKELESIRDRMQLDLTMTKTR
LSKELEEVRKKYEEEREAKIRVQNLEKEQYNELVQLREQVKELNNT----KTYLETVQNE
LTKEKESCKAAKEALASNATALQSERRKLNDMEKEYRKTLASLHDATSELEQLKQKTSEF
SMTRDSEMTALRKEMNAKHSEALASWRRESEERQQVVENEVRSHFEGVVEGLRSQLDEAT
KEITDLRTDNERTANDYDESLKLRQSLTEKLAVVESQYRADRKKFQEDRRAYEANIEAAR
KARKEKDLEFNDLMDIKIALDAEISAYRSILDREETRFGVEPKATASGDFRIRQLNLDEG
RIILENTGATPLPLNGWQITSKSSNHVFSFPDDYVIQPGGKVSVLSGK-NEGEQDQMDFY
AVKKAIWNPKGDVALVKNAEGEVVCSHAEGI
>eurych
XXXXXXXXXXXXXXXXXXXXXXXXXXXXXXXXXXXXXXXXXXXXXXXXXXXXXXXXXXXX
XXXXXXXXXXXXXXXXXXXXXXXXXXXXXXXXXXXXXXXXXXXXXXXXXXXXXXXXXXXX
XXXXXXXXXXXXXXXXXXXXXXXXXXXXXXXXXXXXXXXXXXXXXXXXXXXXXXXXXXXX
XXXXXXXXXXXXXXXXXXXXXXXXXXXXXXXXXXXXXXXXXXXXXXXXXXXXXXXXXXXX
XXXXXXXXXXXXXXXXXXXXXXXXXXXXEKYTALEKNFADHKKRWKEEKAALEHTLDVNR
NARVEKEKQYNTLLDQKLTLDAEINXXXSILEREESRVGLPTPKASNEPVIIHDLDFEKD
FIQLRNTSDSAISFSGWTVLSDGPTHKFSFPENYSLKANTVVTVLGGKKND---PDKDKF
WTNKYMWK-DSNIAEVRDAKGELVVYKVYGD
>bremia
LSPIKSKRMEEKASLQQLNSRLEMYVLGVNELEGAKNAAERELETIRQRMQQDLDSVRTR
LTKELEETRKXXXXXXXXXXXXXXXXXXXXXXXXXXXXXXXXXXXXXXXXXXXXXXXXXX
XXXXXXXXXXXXXXXXXXXXXXXXXXXXXXXXXXXXXXXXXXXXXXXXXXXXXXXXXXXX
XXXXXXXXXXXXXXXXXXXXXXXXXXXXXXXXXXXXXXXXXXXXXXXXXXXXXXXXXXXX
XXXXXXXXXXXXXXXDYEESLKARQALTEKVAQLEREYREIRSKATRDRKAYEETLERFR
SSKVAKEHEFNELMDVKIALDAEIMKYRRILDREEHRVAVATPNTLVSPVQIAAIDLKKD
RITLKNTSNDAIPLGNWVVRGQMD-QTFRFPETYVMRPHSTLTVHSSKRNNERQKGEDSF
LANKFSLNVKGDFVVLMTSDDVPVSIKSEGL
>aphinv
FSPMKSKRLDEKTTLQELNNRLEMYVLRVKEVQDSRDVAEKELETIRERMQMDLSMTKTR
LSKELEDTRKLLEFEIDQKTRLQVLEQEQHTELVKLRTQVKEFGDI----RVELEQVQAE
LAKEKESSKAAKEALALQTTSLQSARRKLKDLDKENRKLTSSLSDTTNELDQLKQKTSEF
SLTRDTEITLVRKEMNAKHLEALAAWRRESEERLHSVEAEVRAHFEGQIEQLRSQVDEVN
LELDSLKVEYERTANDYDESLKIRQSLTDKLSTIETQYRNERKKFQEDRKMYEANIDNAR
QARLAKETEFNDLMDIKIALDAEISAYRSILDREESRVGIDQANHSTGVLRITYLNLEQG
RITLENTGSDALSLSGWQVSSKATNVVFAFPEDYVIQPNGRVSVISGR-NEEEKESMDFY
VIKKAMWNTQADVAQLTNPSGDVVSSYAEGM
>aphast
FSPMKSKRLDEKTTLQELNNRLEMYVLRVKEVQDSRDVAEKELDTIRDRMQMDLSMTKTR
LSKELEDTRKLLEFEIDQKTRLQVLEQEQHTELVKLRTQVKEFGDI----RVELDAVQGE
LAKEKESSKAAKEALALQTTSLQSARRKLKDLDKENRKLASSLSDTTNELEQLKQKTSEF
SLTRDTEITLVRKEMNAKHLEALAAWRRESEDRLHNIEAEVRSHFEGQIEGLRSQVEEAN
LELDSLKIEYERTANDYDESLKIRQSLTDKLSTIETQYRNERKKFQEDRKTYEINIDNAR

QARLAKETEFNDLMDIKIALDAEISAYRSILDREETRVGIDHANHSTGAVRITYLNLEQG
RITLENTGTVALPLAGWQVTSKATNVVFAFPEDYVIQPSGHVSVISGR-NEEEKESMDFY
VIKKAMWNSHADVAQLTNPAGDVVSSYAEGM
>apheu
XXXXXXXXXXXXXXXXXXXXXXXXXXXXXXXXXXXXXXXXXXXXXXXXXXXXXXXXXXXX
XXXXXXXXXXXXXXXXXXXXXXXXXXXXXXXXXXXXXXXXXXXXXXXXXXXXXXXXXXXX
XXXXXXXXXXXXXXXXXXXXXXXXXXXXXXXXXXXXXXXXXXXXXXXNELEQLKQKTSEF
SLTRDTEITAVRKEMNAKHLEALAAWRRETEERMHNIEAEVRSHFEGQIEGLRTQVEETN
AELDQLKVEYERTANDYDESLKIRQSLTDKLSTLETQYRNERKKFQEDRKTYETNIDAAR
QARLAKENEFNDLMDIKIALDAEIAAYRSILDREESRVGLDSAKHSTGDLRITYLNLEQG
RITLENTGSTPLPLTGWKVTSKTSKHVFTFPDDYVIQPGGQVSVVSGR-NEEEKEAMDFY
VIKKAMWNPQADIALLTNPQDEVVXXXXXXX
>nannoch
GTPAIGKDKQNKSRGDDMYKQVEEYVMRVKDLEAAKKADEKELEAERRRLQQEVDELRND
LKRNLQDENENLERELEHQESLGVVVEEEKPGVPELEAEIRTLKEQ---VEGLTEDLR--
---TRMSAAEETESLKDEVEELRASLKAATSADRERR---EARDSIVTQLEVCREQ----
-----ARKEKDRCQELQKRAEALVEAQKAAEEKARRLESPSRTIVKARATALESTVKSLE
GELSLLRRERNKNLGDVDALQEDKEALEDEVRQIEQQRRQAEAVLARETESLERKIDRLR
GTSLGGTEEGSELVEENARLRREVDVYRELAGSQHDQISL---------VYIQEVNAQAD
YLVVENRTNLDVSLAGWALGTSLSESQFEFPEELMLSSRASLKVWWGARNSRPTRG-NLF
WEESDIFQAQNDEVLLMDAEGCEMSMAVEGS
>ochrom
XXXXXXXXMEEQNTIDDLRRDLESYVNSVAVIEXXXXXXXXXXXXXXXXXXXXXXXXXXX
XXXXXXXXXXXXXXXXXXXXXXXXXXXXXXXXXXXXXXXXXXXXXXXXXXXXXXXXXXXX
XXXXXXXXXXXXXXXXXXXXXXXXXXXXXXXXXXXXXXXXXXXXXXXXXXXXXXXXXXXX
XXXXXXXXXXXXXXXXXXXXXXXXXXXXXXXXXXXXXXXXXXXXXXXXXXXXXXXXXXXX
XXXXXXXXXXXXXXXXXXXXXSENKQFKEIIAQLEKERELEAERWNKGKESWEQAIQHGR
HQKKQTMIECEKVMDSSVLLSCLLRANSSTERAEHDR-GVFESMAIKPTVTVQDFDIEDN
HVSISNRTDMEVSLTGCSLKFKGSGAKYKFPDDLIIHPTSTISLWYGKENQAQNRGSLYW
HSNSAITTDFAESVGLVCKKGTTFCRHATGQ
>cafe
GFAGSRRRVEEMRVLGSLNNRLEQHVRQMRTLAKERDALLDHSTTMRDRAAAHQEEV-SR
LNRQIRSLTGKAD-------KAETAVRRLEQVVKTLQGQVDDIDVVRQQRDSLAANLRN-
---TRSDLANMEELLRDAMEGLEAAKAGSKDLELQLAAETAALDAAQTALRRLRDQADAA
SEASDDEEDTIRAELRARKVE------------LEDAEAELRAELEGLRGGSAGQIAALK
AELERVRAEL-------AEALSQQEQLTERANDAEKAAAAAEARAEASERAVEAAAEAAA
AARAAAEAEVSDLEEERSVLAAELKQYERILDTDMAFLGINTPQRKGXXXGASHLDLHQE
VVIVVNTGSDTVAMDGWHVLS-EQGQRFDLPEGQSIKAGSCIAVWSGPDGNAATHKASLL
WTERPVWNNKGDVARLVDSSGDIAAERAVSD
>corallo
ASPARQTRASEKDEMQELNRRLEFYINKQREREARRSTEERELMTAKQVVSREIDRARAE
YQSQLAEVRKQRDQHVASKTHLQTTIARLQQNVYDLEGRLKQEQAEHKELESRRDQLSNQ
LKDVNKELQNAKELLRSTEGDLEDQQKQNDALEGEIDRLRKEYEDAVKNASEAEARMKQL
SNIDKDVENQLRQEFTNQLNSILAERQNQYEAEKNALANELRTLYDEKVSAYRAQVEKSS
QEKAQLELETQAAQAEVNKAIADRNSLIAQIQALQQELSQARDGPEREIEHKKRIIRRMK
EAYQRKEAEFDALMDVKIALSMEIKRYRQMLDEEEDRLGIXXXXXXXXXXXXXXXXXXXX
XXXXXXXXXXXXXXXXXXXXXXXXXXXXXXXXXXXXXXXXXXXXXXXXXXXXXXXXXXXX
XXXXXXXXXXXXXXXXXXXXXXXXXXXXXXX
>retic1
XXXXXQHRLEEKRELRDLNQRLTMLVRHQREGRSEVNHLKKLLAETEVDFRQKLKDSEKH
YQLLQDKFMAENEQLNLHNKTLREQLDTTEKPKEPVKMLRADM--------TELELMQNQ
LEG---LMNELVAVKASVKASSKNDEQLAGQLQEQCDQWKSKFDMTCEELAKIKSRLREY
VKKGQELQNQIRQEFDTKMTEFVEKREIQYQSEKEEWMKIFKEEYQKKMTQFKESNKKLE
SEINDLKLRLTRVRREKAEIDAEKHHLEEELEKARNDTDELRQRKDDELREKTLLVSSLQ
ESLKQKETAFEELSGAKIQLDNEIAVENSFFKKTEKEAGYVNPLEXXXXXXXXXXXXXXX
XXXXXXXXXXXXXXXXXXXXXXXXXXXXXXXXXXXXXXXXXXXXXXXXXXXXXXXXXXXX

XXXXXXXXXXXXXXXXXXXXXXXXXXXXXXX
>retic2
XXXXXQQHVTEKKELSNLNHRLEMFVRHQRDSRNEISRLRKALVDTEVDLRQRLKDAEKR
HQIIQDKIMAENEQLSIQNKTLQDQLETTEKAYQSSESRRASLDEKVTCKMTEIELLQNQ
LEA---LTNELITVKSSVKLMVKNDEHTAEHLQAQCDQWKNKFDVTCEELAKFSFLLNES
TTKFQDMEDQLRKEFDVKMLEFTQKRESQYQNEKEEWIKMFKEEFQKKLAHFKESNKKLE
CEIVDTKARLARVRREKLELEAEKRSLEEEVEKSHSDVNELRQKKDEEIHEKTESLRTLN
DTIRQKDIQYQQLSSAKVQLDNEIAXXXXXXXXXXXXXXXXXXXXXXXXXXXXXXXXXXX
XXXXXXXXXXXXXXXXXXXXXXXXXXXXXXXXXXXXXXXXXXXXXXXXXXXXXXXXXXXX
XXXXXXXXXXXXXXXXXXXXXXXXXXXXXXX
>retic3
XXXXXXXXXXXXXXXXXXXXXXXXXXXXXXXXXXXXXXXXXXXXXXXXXXXXXXXXXXXX
XXXXXXXXXXXXXXXXXXXXXXXXXXXXXXXXXXXXXXXXXXXXXXXXXXXXXXXXXXXX
XXXXXXXXXXXXXXXXXXXXXXXXXXXXXXXXXXXXXXXXXXXXXXXXXXXXXXXXXXXX
XXXXXXXXXXXXXXXXXXXXXXXXXXXXXXXXXXXXXXXXXXXXXXXXXXXXXXXXXXXX
XXXXXXXXXXXXXXXXXXXXXXXXXXXXXXXXXXXXXXXXXXXXXXXXXXXXXXXXXXXX
XSIKQKDCEYQKLSAAKVQLDNEIALYRNILSNAEKETDHFNPLGQLCPLQFSAMDLSSS
TLEVQNTSEEPLNLNGFYLTNTDTSKQFHLPSNRIAHIGFVFAIIVGDTPGLKVKTGDLR
WHDNVWSGLKDELVRLYNPQHEEIARIEIAP
>retic4
XXXXXXXXXXXXXXXXXXXXXXXXXXXXXXXXXXXXXXXXXXXXXXXXXXXXXXXXXXXX
XXXXXXXXXXXXXXXXXXXXXXXXXXXXXXXXXXXXXXXXXXXXXXXXXXXXXXXXXXXX
XXXXXXXXXXXXXXXXXXXXXXXXXXXXXXXXXXXXXXXXXXXXXXXXXXXXXXXXXXXX
XXXXXXXXXXXXXXXXXXXXXXXXXXXXXXXXXXXXXXXXXXXXXXXXXXXXXXXXXXXX
XXXXXXXXXXXXXXXXXXXXXXXXXXXEEELEKSHNDLDELRQRKDEEIREKTLLIMSLQ
EQIRQKDIQFDALSGAKIQLDNEIALYRSIWSEAEKEAGYSCPLGXXXXXXXXXXXXXXX
XXXXXXXXXXXXXXXXXXXXXXXXXXXXXXXXXXXXXXXXXXXXXXXXXXXXXXXXXXXX
XXXXXXXXXXXXXXXXXXXXXXXXXXXXXXX
>retic5
XXXXXXXXXXXXXXXXXXXXXXXXXXXXXXXXXXXXXXXXXXXXXXXXXXXXXXXXXXXX
XXXXXXXXXXXXXXXXXXXXXXXXXXXXXXXXXXXXXXXXXXXXXXXXXXXXXXXXXXXX
XXXXXXXXXXXXXXXXXXXXXXXXXXXXXXXXXXXXXXXXXXXXXXXXXXXXXXXXXXXX
XXXXXXXXXXXXXXXXXXXXXXXXXXXXXXXXXXXXXXXXXXXXXXXXXXXXXXXXXXXX
XXXXXXXXXXXXXXXXXXXXXXXXXXXXXXXXXXXXXXXXXXXXXXXXXXXXXXXXXXXX
XXXXXXXXXXXXXXXXXXXXXXXXXLYRDILNEAEKDAECFFQTNDPFPLQFSGMDLCSS
TLELQNVSEEPVDLKGFYLTNSNKSKIFILPHHHVLMPKPKFNIIVGNDGDLNVGKDDLW
WQENVWNGLEDEIARLFNSKNEEVATVNITT
>retic6
PFNSIQQQMDDKHKIRDLNTRLKMFVRHQQEGRSEVARLKQLLEETEFNFRQRIKDSEKR
FQLLQDKMQAENEQLTVQNNSLREQFENAEKARQNAESRCANLDQTVARKSTELEVLQKH
LDS---LTNDLIAVKASAKASLKNDEQLTGQLQEQCDQWKAKFESNCEDLAKIKARLKEC
WKKSQDMENQMRKEVDIKMSEXXXXXXXXXXXXXXXXXXXXXXXXXXXXXXXXXXXXXXX
XXXXXXXXXXXXXXXXXXXXXXXXXXXXXXXXXXXXXXXXXXXXXXXXXXXXXXXXXXXX
XXXXXXXXXXXXXXXXXXXXXXXXXXXXXXXXXXXXXXXXXXXXXXXXXXXXXXXXXXXX
XXXXXXXXXXXXXXXXXXXXXXXXXXXXXXXXXXXXXXXXXXXXXXXXXXXXXXXXXXXX
XXXXXXXXXXXXXXXXXXXXXXXXXXXXXXX
>retic7
XXXXXXXXXXXXXXXXXXXXXXXXXXXXXXXXXXXXXXXXXXXXXXXXXXXXXXXXXXXX
XXXXXXXXXXXXXXXXXXXXXXXXXXXXXXXXXXXXXXXXXXXXXXXXXXXXXXXXXXXX
XXXXXXXXXXXXXXXXXXXXXXXXXXXXXXXXXXXXXXXXXXXXXXXXXXXXXXXXXXXX
XXXXXXXXXXXXXXXXXXXXXXXXXXXXXXXXXXXXXXXXXXXXXXXXXXXXXXXXXXXX
XXXXXXXXXXXXXXXXXXXXXXXXXXXXXXXXXXXXXXXXXXXXXXXXXXXXXXXXXXXX
XXXXXXXXXXXXXXXXXXXXXXXXXXXXXXXXXXXXXXXXXXXXXEPNPLQFSTMDLCSS
TLEVQNVSDHAIILKGFYLTNLDKSKKFLLPPGRVLKPSFVIFIIVGNDSNLRVAEGDLW
WNESVWSGTKDEIARLYDSSNQEISRVEISP
>ammonia1

```
PGSIAKQALAQKRNVRDLNTRLEMFVRAQAEKTRAINQLKESLARQEMDYKSKLTQQRVQ
FQDNVEKLRKENQNLAYENKCTQQEFTNALTSKAEFEERLLKSEEKNAALSSENSNTQQE
FDD-------MRKAFATMKYKYDSYEAERCSFESRMAKQKSKTEQLLKELTQKEATVKKL
ELENSTTQSRLRQEFDNKLAEFVQKREEQYKQEKDEWMRIFKEEFNRKLRSFKHSNIKQS
EEIQDLRARISKLKQQKTELEVTNRNNEEEIEKLRNDLDDLRRTKDAEIKLKNSLIIQER
DRFKAKELQFDELAGMKLQLDAEIELYRNILNEAERDCGYNSPMVEGGALQFSGLDLNKG
MIEIQNMGEEPVSLDGFALTNQSGSMIYDLPTDMELQAKNTLRIYVGEKDSESEKDQQVF
WGNDVWTGTDTDCARLYNPSQEEIARIEISP
>ammonia2
PGSPAKQALTQKRTVRDLNTRLEMYVRAQAEKTRNINQLKEALARNEMDYRNKLKQQKIH
FQEKVEKQRKQIENLEYDKKCTQQELENAMSAKTEYEERLISSEGRNAALASECEQLSKE
LEG-------LRKTYASLKYKYDSYEIERNSFGEKMQKLKSKNEQLLRELTANEQHLKEL
ELTHNSTQTRLRQEFDQKLAEFVTNREQQYETEKAEWMRIFKEEFNRKLRSFKHANVKQS
EEITDLRTRMSKLKQQKTELEVLNRNIEEDAEKLRTDLDNLRRQKDEEIKQKNAVILQQR
DAYKAKELQFDELASIKMQLDSEIELYRNILNDAEQECGYTSPLAEGARLQFSGLDLLKG
MIEIQNMCEQPISLRGYTLSNDTGTSQFPLPNDVVLDHNQKIRIYCGKQEDDEDAAPQVS
WGQDVWTGQSNDCARLYNPNHHEIARIEISP
>elphi
XXXXXXXXXXXXXXVRDLNTRLEMFVRAQCEKSREVNTLKEALARSEMEYRNKMKQQKLQ
YQDQIEKSRKAIENLEYDNKCTQQELDNGIASKKELEDRLMSSESRNASMSSENDTLKTE
LDT-------VRTAYAALKYKYESYELERNSFDDKVNALKSKNDKLLRELTQTTSTNKQL
KLANTTTQTRLRKEFDSKLAEFVQKREEQYKQEKDEWMRIFKEEFNRKLRSFKLANCKQL
EENTELRERISKLKQLKMELEVQNRNNEEETEKLRTDLDNLRRQKDEELKQKNAIIIQQR
DAYKAKELQFDELAGIKMQLDSEIELYRSILNEAEQACGYVSPFNEGSALQFSGLDLVKG
MIEIQNVGEHAMPLKAHALSNKEGTQQFPLPSHITLDSHQKLRIYVGEYKAEDDAQQEVF
WTKDVWTGDSKDCARLYNAQQQQIQHIEISP
>D1_289
XXXXXXXXXXXXXXXXXXXXXXXXXXXXXXXXXXXXXXXXXXXXXXXXXXXXXXXXXXXX
XXXXXXXXXXXXXXXXXXXXXXXXXXXXXXXXXXXXXXXXXXXXXXXXXXXXXXXXXXQE
IIR-------ARADAGSANLKLEIANKNIARLQDQLVEYQAADTDTKALLATERQKVLSP
STQKSDAAGPLQQVFDKRLQSVIADQCSAAERNRQIAMTEQKLYFQEKVKGYQAQLQVAG
CEIEQQKNRLEAAAEDYNEAMAFANALEVRIFGLEEAIKKEKSKPSKIIAEKNEIIRKVQ
EVLKEREEEYDELMETKINLQVELQNYQSLLQQEESRCGWENATNADNKICVLSAGSDHR
GLFLVLSSSEFLDISEWSFQVSGQRLVYTVPRDFSIEGAGSFRVFISSEPVSVEEKTEAW
WXXXXXXXXXXXXXXXXXXXXXXXXXXXXXX
>D1_625
PKPTRKARLEEKSELQDLNKRLEFYILKQREKDANAGSIQRTISENKEYYENEIKKLKSL
HETQVNNFRKQRDELDSEIQSLTEENKRQASMLKSYTSQLSADKTRGTDLEDRVSQLTEE
LLEAQAATAVAEADAKKSAHGLKKAESEIKHLQKELKDSQARDKDHAVELAAAQSLESAL
SSTRANAEDKLRALFGAQLKEILAERQRQFDEKKAEGLQELRDSYEGKLSEANEKLEEAS
KEVAALKEQANSASSTNSSEESERTKLNAKINTQKKELSKAEGQARKQAAFLEEQCESHK
QENQTLNEEYISLMDVKVGLDMEISEFRRLLSDEESRLQPQEEEPPAKPTIISGID-SKK
WIEITNSVKEAVCLDGWSLKVVSSKECFFFPDVAPLKPGKTLKINLGS--KAKKGKNAVV
WAKDDIFKEE-DKVYLMSPEGVGHSSVAWND
>spongos
ESPIRIHPEVERKNLEELNGRLQQYIMKQRAKDASREAFEKDVVAIQQQARVAIHANTKK
YEEQLRQMRQQRDEHASAREELQVRVARQEATVTRLKDQIAEEKKFQSDLNGRLSSLSSQ
LEAANAQVQRLQEQLRKTEHNFKSAESAGRAAEEQLIEARDAADQYSHEASSLRAKIEQL
SAQVTITEDSCRQEFASQLSSIVEDYRKQCNDDKTRITRELKSHFVPKLAELRAAYEESC
QHESELREQVNQLKKDNQTLAEMKSLHEKRIAELSEELDTERNVTHDAIAAKDKEIEKLR
HTCQRLETDFDDLMDVKIQLALTIEKYRELLSEEEHRLGLETPGRQTPALKLWVNDEVEN
CLFVKNTSDKIVNLNGMILQS--GSFSFDFPDDAYVGGQCEVCIWIGETSNSLEASSEIS
WEGVNPEDLTAAPMQLIDQNGTVIDQFEVET
>plasmod
ESPIRMQRDVERKNLEELNGRLEQYIMTQRRKDASREAWEAELKAVQTSAAQALADTTAK
YETWLRQMRLQRDEHASAREELKVRVSRMEETISRLKEQIANEKKYENELSTRVSNMSAQ
```

LEARDALVVKLQENVRKLEQSLKVAENAGRAAEEQLVEARELADQYSHEASAMRAKVAKL
TKSSAESEDTIRTEFMKQLESVVEQYRHQCNEDKARITRELKSHYMPKLTEARQALEETC
NREAELREEITLLKGQLETMQELKTVHEKRINELVAELDKERNVIHKAIAEKEKEIEKLR
QRYARLETDFDNLMDIKIQMALTIDKYREILTEEERRVGLETPGRPTLPVQLA-IDEENG
CLVVENVSDEDFSLDGWSLQS--SGFVFEFPEDTLLPAKSRVSVWIGEAPEEQEGDDELV
WEGVDCTELAEEPMCLCNEDGAVQDQIEVEA
>beta
ESPVRLQRDVEQKNLEELNARLEKYILKQRAKDASREAFEKDLAKIQQQARQAIHANTKK
YEEQLRLMRQQRDEHASAREELQIRVSRQEAVITRLKDQLTDEKKYQNDLNQQVSGLKSQ
LEEARGQNSKLQDQLRKTVHALKVAESAGRSAEEQLAEAREGADKSSHEASALRAKXXXX
XXXXXXXXXXXXXXXXXXXXXXXXXXXXXXXXXXXXXXXXXXXXXXXXXXXXXXXXXXXX
XXXXXXXXXXXXXXXXXXXXXXXXXXXXXXXXXXXXXXXXXXXXXXXXXXXXXXXXXXXX
XXXXXXXXXXXXXXXXXXXXXXXXXXXXXXXXXXXXXXXXXXXXXXXXXXXXXXXXXXXX
XXXXXXXXXXXXXXXXXXXXXXXXXXXXXXXXXXXXXXXXXXXXXXXXXXXXXXXXXXXX
XXXXXXXXXXXXXXXXXXXXXXXXXXXXXXX
>festuc
ESPVRLHRDVEQKNLEELNARLEKYIFKQRAKDASRESFEKDLANIQQSARLAIHSNTKK
YEEQLRLMRQQRDEHASAREELQIRVSRQEAVITRLKDQLTEEKKYQNDLNQQVSSLKTK
VEEAHGQNSKLQDQLRKTLHALKVAESAGRSAEEQLAEARDTADKFSHEASSLRAKIDKL
QAQIAITEDNCRSEFSSQLAKIVEDYRKQCNDDKTRVTRELKAHYMPKLTELRVSLDEAM
RRENDLRDQVSQLKREMQSLNDIRTVQEKRIQELTEELDRERNFTHDVIGAKDKEIERLR
HECQRLECDFDDLMDIKIQLALTIEKYRELLSEEEHRLGLETPGRPEGPSQLGVHEQLDN
CLLIKNASGETMNVNGFVLKS--GSLDFHFPDNIQLPAYGEICVWIGEXXXXXXXXXXXX
XXXXXXXXXXXXXXXXXXXXXXXXXXXXXXX
>lingul
XXXXXXXXXXXXXXXXXXXXXXXXXXXXXXXXXXXXXXXXXXXXXXXXXXXXXXXXXXXX
XXXXXXXXXXXXXXXXXXXXXXXXXXXXXXXXXXXXXXXXXXXXXXXXXXXXXXXXXXXX
XXXXXXXXXXXXXXXXXXXXXXXXXXXXXXXXXXXXXXXXXXXXXXXXXXXXXXXXXXXX
XXXXXXXXXXXXXXXXXXXXXXXXXXXXXXXXXXXXXXXXXXXXXXXXXXXXXXXXXXXX
XXXXXXXXXXXXXXXXXXXXXXXXXXXXXXXXXXXXXXXXXXXXXXXXXXXXXXXXXXXX
XXXXXXXXXXXXXXXXXXXXXXXXXXXXXXXXXXXXXXXXXVVISSLDLEAD
SVVITNNGSEAVSLKGWKLVSKTGDQEYAFKAKDSIDAGASLTVLSGKKAARKGGADHVF
WTARYIWNNEGDEAGLLNADGEEVSSLAGTG
>gleno
XXXXXXXXXXXXXXXXXXXXXXXXXXXXXXXXXXXXXXXXXXXXXXXXXXXXXXXXXXXX
XXXXXXXXXXXXXXXXXXXXXXXXXXXXXXXXXXXXXXXXXXXXXXXXXXXXXXXXXXXX
XXXXXXXXXXXXXXXXXXXXXXXXXXXXXXXXXXXXXXXXXXXXXXXXXXXXXXXXXXXX
XXXXXXXXXXXXXXXXXXXXXXXXXXXXXXXXXXXXXXXXXXXXXXXXXXXXXXXXXXXX
XXXXXXXXXXXXXXXXXXXXXXXXXXXXXXXXXXXXXXXXXXXXXXXXXXXXXXXXXXXX
XXXXXXXXXXXXXXXXXXXXXXXXXXXXXXXXXXXXXXXXXXXXXAPSGIKIDNVDLVSD
CVSIVNTTGASQSLKGWHLTSEVGDQIFHFPDDLVLPPGERVTVWSGKNARHHNPPYHLF
WTKRFIWNNNGDTAVLVDANNNTVSKITSGP
>alex
XXXXXXXXXXXXXXXXXXXXXXXXXXXXXXXXXXXXXXXXXXXXXXXXXXXXXXXXXXXX
XXXXXXXXXXXXXXXXXXXXXXXXXXXXXXXXXXXXXXXXXXXXXXXXXXXXXXXXXXXX
XXXXXXXXXXXXXXXXXXXXXXXXXXXXXXXXXXXXXXXXXXXXXXXXXXXXXXXXXXXX
XXXXXXXXXXXXXXXXXXXXXXXXKWKEESDSRAAGIEAELREAFTSSSEQLKERAQILN
TQNVRLQEEMDSLMEELQKANEEAKERLQELQASRASHSEESQAWAMERSRLEKEVTSLH
EAHAEKIDEFNDLMDIKVSLQAEIEQYRAILEGEEARLGVDAPSSAASGVKLDNVDLVSD
CVSIKNFTSEDQPMKGWVLKSAVGSQVFRFPDDLVLPPGKRVTVWSGKTARHHSPPYNLF
WTRRFIWNNHGDTAVLINADNMEVSRITSAP
>symbio
ESEIRQSRVQEKHQLHNLNDRLQNFVLHSKAMEEENADLRRQLRDMEERMKESLQEVHQE
YSKQSKKLRTSVTTMTAKNNGLESENARLLAEVHALNKKIQQTDSLREENHKLSDQVRD-
---LSDELRKYKAKVSQNEEEIERLREIIANHEEVIATREDQLQEATKELETVQKTIKKG
ARHSDGELAE---RVRASLTEAHKKWQRETQTALQKLETDLRQHFKHTTVELNERSETLV

```
RQNERLRKENERLANELDNNAGGQQDLLNKLAEQERQHKHVVTQLQRDLMKREEELTTAA
SSLVNKNNEFNDLMDVKISLQTEIEKYRSILDVEEARINTPTQDRKTGPLHISDVDLISD
CVSVANETSAPVPMKGWTLISETGNQVFNFPDALVLKPGDRVTVWSGAHASHHAPPDNLF
WTRRYVWNNHGDTAILVNPAGEHVSIVSGVP
>pavlov
ESATRLTRVEERETLQNLNSRLQYFLLRMKELEARNGELQTKVDSGSAGAKAHNDSMRAV
FEKQIAELRSAQETDVELIAELEAKASENGKVASAYKKKAERCDEAEVKLRAVIEERDKA
LVL----FKNTEERAEKAEQAAATFEAKASLLEKKL----APLREQLSTAEELRRKIDEL
SGANRGELSRMQAAYDAQMQDALDVAKLRAEEGSRAELEQLAAHFTSVESELTREVAEEK
AAKESALKRANDLAAN-KESIQARVSIAERVTELERTLADNSRESDGKCAKLEQALEVAR
SERKTKEGEFNELMDVKISLDEELKTYNMLLGREEARLGITPTQDTQGPMYVSQLNLNDD
CVAVKNAGDEPALLGGYTIKSHSEDLVFVFPSEFELLPGASVTIWSGKKNRRRDPQTELW
WSARYMWSDDGDMATLFDTTGAEVSKLEAAA
>pavlosp
DSATRLSRVEERDTLHNLNSRLQLFLSRMKELEARNTELQSKLDMGSSASQKVTDSMHAL
MEKQIQDLRAASEKDTELIAELQASSGENIRSNQAMKAKAERAAELEGKLRVITDERDRL
AAA----LKDAERRADVAEQAAVTSESKATVLEKKL----APLRDQLQTSEELRRKISEL
STANKTEVARIQADYDKQLQAALAAAKKRADEERAAEVAQLAEHFNVVEAQLTREAKDER
ASKEAALARAAELGKT-NDQLQSRADASERAAAAEKNLTERARQHEATVAELDSLLHQAR
AARKEKEGEFNDLMDVKISLDEELKTYRLLLEQEEQRLGYTPEPVKSGPVYLAQVDLEND
CFTVQNAGDEACVLGGYSVKAADSEGAYEFPADFELLPGSSVTVWSGKKNKKRDPQAELW
WTARYMWNNEGDTGILLGPGGEEVNRVTAPA
>pavl
DSATRLSRVEERDTLHNLNARLEYFLSRMKELEVANDDLQVRVDTAASVHKTQSDQLRDM
YEKQLTDMRKTSEKDVELITELQKEANEATKDKVALTRKAQLADELDVKLRAVTDERDRL
ASR----AKEAEARAAELEKSAAVFEAKANMLEKKL----APLRDQLATAEELRRKIAEL
SGGSRSEIDKLKASYDKQLLEAMEKQKKRADEERLTELAQVREHFTAVEAELMRAVDEEK
AARASADERMGDLNAQ-VRQLRARASQAEQAAALEKTLAERIAHFDAAIARLDQQLQEAR
AARQQKEVEFNELMDVKISLAEELKTYTKLLEGEEERLGITPTKEASGPLYLATLDLIND
SFALKNAGDAPAPLGGFKVVSHATDVSFEFPDDFELQPGATVTVYSGKKNKKRDPRTELW
WSPRYMWRNEGDEAIMYDSSGSEVGRLVAEA
```

NMCP:

```
>daucus1
EDMGLNAKLMKLETELFDYQYNMGLLLIEKKEWTSKFEELQQVYTETKDALKQEQEAHLI
AISDAEKREENLTKALGVEKQCVLDLEKALRDMRSDYAEIKFTSDSKLAEASALITKVEE
KSLEVESKLHSADAKLAELSRKGSDIERKSHELEARESALRRERLALNAEREALTDNISR
QREDLREWERKLQEDEERLAEVRRLLNQREERANENDRLYQQKQSELDGEQKKIEIIMVS
LKNKEDDISSRIAKLNIKEKEALEVKEKDLTEFEQKLNAREQSEIQKLLDEHKAILEVKK
QSFEMEMDKRKNDFENDLQNRAVEVEKKEVEVKHLEAKLAKREHALD
>daucus2
DRDAVVEKVAKLERELFDYQYNMGLLLMEKTEWTLKYEEMRRAQVELKEVLEQEQTTHLI
LLSESEKREENLRKALDMEKKCITDLEKALRDSGADNAQTKQSSEAKMVKANALLSGFKE
KSMDVETKLHVADAKLEEVYKTSLELERKLQEVETRDSLLQRERMSFIAEREAHEATFSI
QKKDLQEWEKKLQEAEERLCEIRRTTSGREVKVNEMEMALNLKKQELNKAQKENDLSTSV
LKKEADDINHRLANLTAQEHKALEMRDKELLALAEKLTARESVEIQTLLDEQQAVLDAKM
QEFEVDMDGKRKSLDEEMRSKLDAVQYKKDEITHIEEKLNRLELSLE
>daucus3
DKASLIAYITKLEAEIYDHQYQMGLLIMERKEWGSKFERVEAALNSAELMRKHDKNLYLK
DLAEAKKREENLKKAIEIERECLANIEKTLHELRAEYAETKVMADSKLVEARSMIEDALK
KLSEADAKKHAAESLEAEASRYHSAAERKLHEVEAREDDLRRRATSFKTECDTKEEEILH
ERRLLNERQKALQQSQQRLVDGQDLLNKRESHIFERTQELNRKEKELEASKLKQEEELQA
LVEQQANLETKASSLSLREEVIVKKREEELCVLQEKLEKKESERIQQLLANYEASLSMKK
SEFEAELEVKRKSVHDDIENKRRDWELREVDLHHREELILEKEHELE
```

```
>citrus1
DVESLAEKVSKLENELFEYQYNMGLLLIEKKEWSSKYEELKQTFGEAKDALKREQAAHLI
AITDVEKREENLRKALGVEKQCVLDLEKALREMRSENAEIKFTADSKLAEANALVTSVEE
KSLEVEAKLRSVDAKVAEINRKSSEIERKSHELESRESALRMERASFIAEREAHEGTFSQ
QREDLREWERKLQDGEERLAKGQRIVNQREEKANEKEKIFKQKEKDLEEAQEKIDATNLS
LMRKEDDINKRLANLITKEKEYLEMKEEELRQLEEKLNAREKVEVEKLLDEHKASLDAKQ
REFDLEIEQKRKAFDDDLKSKVVEVEKKEAEINHKEEKIAKREMALE
>citrus2
DREALMEKVSKLEKELYDYQYNMGLLLIEKKEWTSKIEELRQSFEETQEILKREQSAHLI
AFSEAEKREDNLRRALSMEKQCVADLEKALRDMGEEHAQTKLFSEKTLTDANTLLGGIEG
KSLEVEEKFHAAEAKLAEVNRKSSELEMKLQELESRESVIKRERLSLVTEREAHEAAFYK
QREDLREWEKKLQIGDERLSELRRTLNQREVKANENERILKQKERDLEELEKKIDLSSSK
LKEREDEINSRLAELVVKEREAVEMKEKRLLTIEEKLNARERVEIQKLLDDQRAILDAKQ
QEFELELEEKRKSIEEEMRSKISALDQQEFEISHREEKLERREQALD
>citrus3
DKAALIAYIAKLETEIFEHQHHMGLLILEKKELASKYEQIKASAEAAELLQKHDQASHLS
AIAEARKREESLKKTLGVEKECIASLEKAVHEIRAESAETKVAADSKFAEARCMVENAQK
KFAEAEAKLHASESLQAEANRYHRSAERKLQDVVAREDDLSRRIASFKADCEEKEREIIR
ERQSLSDRKKILQQEHERLLDAQTLLNEREDHILSKLQELSRKEKELEASRANVEEKFKA
LNEEKSNLDLTLVSLLKREEAVLQKKEQKLLVSQETLASKESNEIQKIIANHESALRVKQ
SEFEAELAIKYKLAEDEIEKKRRAWELRDLDLGQREESLLEREHDLE
>prunus1
DREELAQRVSELENELFEYQYNMGLLLIEKKEWTSRHEELRQSLTEAKDAVRREQAAHLI
AISEIEKREENLRKALGVEKQCVHDLEKALHEIRSENAEIKFTADSKLAEANALVASIEE
KSLELEAKSRAADAKLAEVSRKSSEFERKSKDLEDRESALRRDRLSFNSEQEAHENSLSK
RREDLLEWERKLQEGEERLAKGQRILNQREERANENDRIFKQKEKDLEDAQKKIDATNET
LKRKEDDISSRLANLTLKEKEYLEMKEKELLALEEKLNARERVELQKIIDEHNAILDAKK
CEFELEIDQKRKSLDDELRNRLVDVEKKESEINHMEEKVAKREQALE
>prunus2
DRQALADKVSKLQKELYDYQYNMGLLLIEKKEWALKHEELGEALAETQEILKREQSAHLI
SISEVEKREENLRKVLVAEKQCVAELEKALREMHEEHAQIKLKSEAKLADANSLVVGIEE
KSLETDAKFLAAEANIAEVNRKSTELEMRLQEVEARESVLRREHLSLSAEREAHKKTFYK
QREDLQEWERKLQEGEERLCKLRRILNEKEEKANENDLIMKQKEKELDEVQKKIELSNTI
LKEKKADVNKRLADLVSKEKEAWELKEKELHELEEKLSSRENAEIEQVLDKQRALCNTKM
QEFELEMEERRKSLDKELSGKVEVVEQKELKINHREEKLLKQEQALH
>prunus3
DKAALIAYIAKLEAEIFDHQHHMGLLIMERKELASKYEEVKASNETTELLHKRDQAAYVS
ALAEARKREECLKKVVGVKEECISSIEKSMHEMRAESAETKVAAESKLAEARNMVEGAQK
KFTEAEAKLHVAESLQAEASRFHRVAERKMQEVEAREDALRRNILSFKTDCDTKEKEISL
ERQSLCERQKTLQQEQDRLLDAQALLNQREDFIFGRSQELNRLEKELEDVKANIEKERRA
LDDGKLNLELTEASLVNREEALLNKKEQEILVLQEKLVSKESDEIRKALASHEVELRKKK
FEFDSELDVKRKLFEDEIEAKRRAWELREVDLNQRDDLLQEREHDLE
>vitis1
DLEALVAKVSKLESEIFEYQYNMGLLLIEKKEWTSKYDELRQALVDVKDALKREQDAHLV
AMSEVEKREENLRKALGIEKQCVLDLEKALHEMRSEYAEIKFTSDSKLAEANALVTSIEE
RSFEVEAKLHAADAKLAEVSRKSSEIERKSQEVDARENALRRERLSFNAEREAHETTLSK
QREDLREWEKKLQEEEERLGEGRRILNQREERANENDKIFTQKEKDLEEAQKKNEMTHLT
LKKKEDDISGRLSNLTLKEKETLEIKEKELLELEEKLCARERVEIQKLVDEHNIILDAKK
REFELEIEQKRKSLEEELKSKVVEVEKKETEFNHMEAKVAKREQALE
>vitis2
DREALVEKVSKLQNELFDYQYSMGLLLIEKKEWTSKYEELSQALAEAQEILKREKSAHFI
AISEVEKREENLRKALGVERQCVAELEKALGEIHAEHSQIKLSSETKLSDANALVAKIEK
RSLEVEEKLLAADAKLAEASRKSSELERKLQEVEARESVLRRERLSLNAEREAHEATFHK
QKEDLREWERKLQEGEERLCEGRRIINQREEKANEIDRTLKLKERNLEEAQKKIDLDSLN
VKVKEDDINNRLAELTVKEKQALEVKEKELIVLQEKLSARERVEIQKLLDEHRAILDTKK
QEFELEMEQKRNSVDEELRSKVHEVEQKEVEVLHREEKLGKREQALE
```

```
>vitis3
DKAALIAYIAKLEAEIFDHQHHMGLLILERKEWATKYEQIKTEAESAEIVYKRDQSAHSS
ALAEARKREDSLKKALEIEKECIANLEKALHEMRQECAETKVAAEIKLAEAHSMVEDAQK
RFVEAEAKLHAAEAFQAEAICFRRTAERKLQEVEAREDDLRRRLISFKSDCDEKEKEIIL
ERQSLSERQKNVQQGQERLIDGQALLNQREEYIFSRSQELNRLEKELEASKSNIEKELRA

LNEEKSNLELKLASLTTREEDVLNKKEHEILILQEKIASKESDEVQKLMALHEIALKTRK
AEFEAELETKRKLVEDEIEAKRRASELREVDLSNREDFALEREHELE
>phaseo1
DRGVLVERVSNLEKELYEYQFNMGLLLIEKKEWTSKYTEQSQDLVEVKDALEREKAAHLI
ALSEAEKREENLRKALGVEKECVLDLEKALREIRSENAKIKFTAESKLAEANALVASVEE
KSLEVEAKLRSADAKFAEISRKSSEFDRKSQDLESQESSLRRDRLSFIAEQEAHESTLSK
QREDLWEWEKKLQEGEERLAKGQRIINEREQRANENDKLCRQKEKDLEEAQKKIDATNIT
LRSKEDDVNNRLADIALKEKEYLDLKEKELSAWEEKLNAKEKVEMQKLLDEHNAVLDVKK
QEFEVELNEKRKSFEDGLKDKLVELEKKEAEINHMEEKVGKREQALE
>phaseo2
DHEALMEKVLRLERELFDYQYNMGLLLIEKKEWNSKFDQLRQELAETEEILKREQSAHLI
ALFEVEKREENLRKALSTERQCGADLERALRAMQEEHAQIQSKSHTKLAEASALVDGIEE
KSSVVDKKLLDAEAKLAEVNRKNAELGMKLQEVEARESLLQKERLSLVTDRELFDATFYK
EREDLKEWERKLQQRENMLCNGRQNIGEKEENIVKTEKNLKQKERDLEVLEKKINSSNSI
LKEKEAEIIRRTADLNMEEKKVLEKKEKELFALESKLSSREREGIQKLLGEQKATLDLQL
QQVEFEMEHKRKSLVEEFSSKEEALEQREVEVNHREKKVEKEEQALS
>phaseo3
DKAALIAYIAKLEAEIYDHQHHMGLLIMEKKDLASKYEQLEALAESSELMHKHDSAMNKS
ALAESRKREESLKKTVSVKDACIASLEKALHELRTESAETKVAAESKFAEAHQLIDEAQK
KITEAEAKVRAAESLQTEANRYHNAAERKLRDVEAREDNLRRKIMSFKADCDEKDKEMIF
ERQSLSERQKGLQEEQERLLQSQSLLNQREEHFLSRSQELNRLQKELEDTKAKVEKEHET
LHDEKTTLKMKEATLMQREEELLSKKEQELLEFQAKLSIRESDETKKVIAGQEAALKTKK
YNLEVELQMQRKWVENDIETKRRAWELKEVDLKHCKDEILEKQHELE
>arab1
DPRILPEKISELEKELFEYQHSMGLLLIEKKEWSSQYEALQQAFEEVNECLKQERNAHLI
AIADVEKREEGLRKALGIEKQCALDLEKALKELRAENAEIKFTADSKLTEANALVRSVEE
KSLEVEAKLRAVDAKLAEVSRKSSDVERKAKEVEARESSLQRERFSYIAEREADEATLSK
QREDLREWERKLQEGEERVAKSQMIVKQREDRANESDKIIKQKGKELEEAQKKIDAANLA
VKKLEDDVSSRIKDLALREQETIETKARELQALQEKLEAREKMAVQQLVDEHQAKLDSTQ
REFELEMEQKRKSIDDSLKSKVAEVEKREAEWKHMEEKVAKREQALD
>arab2
DQEALLEKISTLEKELYGYQHNMGLLLMENKELVSKHEQLNQAFQEAQEILKREQSSHLY
ALTTVEQREENLRKALGLEKQCVQELEKALREIQEENSKIRLSSEAKLVEANALVASVNG
RSSDVENKIYSAESKLAEATRKSSELKLRLKEVETRESVLQQERLSFTKERESYEGTFQK
QREYLNEWEKKLQGKEESITEQKRNLNQREEKVNEIEKKLKLKEKELEEWNRKVDLSMSK
SKETEEDITKRLEELTTKEKEALLAKENELRAFEEKLIAREGTEIQKLIDDQKEVLGSKM
LEFELECEEIRKSLDKELQRKIEELERQKVEIDHSEEKLEKRNQAMN
>arab3
DRDALIEKILKLEKELFDYQHNMGLLLIEKKQWTSTNNELQQAYDEAMEMLKREKTSNAI
TLNEADKREENLRKALIDEKQFVAELENDLKYWQREHSVVKSTSEAKLEEANALVIGMKE
KALEVDRERAIAEEKFSVMNRKSSELERKLKEVETREKVHQREHLSLVTEREAHEAVFYK
QREDLQEWEKKLTLEEDRLSEVKRSINHREERVMENERTIEKKEKILENLQQKISVAKSE
LTEKEESIKIKLNDISLKEKDFVDIKEKELHEFEENLIEREQMEIGKLLDDQKAVLDSRR
REFEMELEQMRRSLDEELEGKKAEIEQLQVEISHKEEKLAKREAALE
>arab4
DKAALIAYIAKLESEVYDYQHNMGLLLLEKNELSSQYEEIKASVDESDLTHMREKSAYVS
ALAEAKKREESLKKDVGIAKECISSLEKTLHEMRAECAETKVSAGSTMSEAHVMIEDALK
KLADAEAKMRAAEALQAEANRYHRIAERKLKEVESREDDLTRRLASFKSECETKENEMVI
ERQTLNERRKSLQQEHERLLDAQVSLNQREDHIFARSQELAELEKGLDTAKTTFEEERKA
FEDKKSNLEIALALCAKREEAVLLKKEQELLVAEEKIASKESELIQNVLANQEVILRKRK
```

SDVEAELECKSKSVEVEIESKRRAWELREVDIKQREDLVGEKEHDLE
>oryza1
KGKGTTLRVAELEQELHEYQYNMGLLLIEKKEWTAKLDEINQALTQKEEILKREQAAHLN
AISEYERREESMRKALGVEKQCVTDLEKALREIRGEIAEVKFMSEKKITDAQSLEASLEE
KRLEIEGKLHAADAKLAEANRKKSQADRDLEEVEARQRRLEKEKLYFENERKAGEDRIKR
QEDSLRDWDKKLKESQNRILDLQRSLNDREERANENDKLFKIKQEELEEAKKALEHTKAT
LKIKEDDINKRLAELHLQEKEALEEREKKIAEREEKVSAREKVGLQKLLEDHNVKLESKR
RDFDLQLENEKKSFDAMLVQKEADLVQREKDVRSSEEKLSKKEQVLN
>oryza2
DKAALIAYISRLESEIYQYQHNLGLVLMERKELTSKHEQLRAASESAEIMHKRERAAQQS
ALAEARKKEENLKKSLGIQKECVANLEKALHDMRGETAETKVSYESKLAEALQLMEAAHK
KFDEAEEKLLLAKSLEAESIRTHNAALRSLHDIDDREDQLRRDRISCELENEAKEKEISL
QRKSLNDMKKILHEKEEVLLKEQALLNQRDENILERLAYVTHSEKRVEEEKNILEAERKV
LLEEKYKLELKMEAIVSREEALLDKRESELLILQETIASKERAEIERLNQEQAIALERRK
HDFESEMANKQMSFDAAMEVTRNALHQRECALSEQESVVVQRSQNLD
>musa1
DRDALVQRISELEKELHEYQYNMGLLLIEKKDWASKYEEIRQALAEVDETLKKEKSACLA
SISEFAKREENLQKALGVEQQCVSDLEKALREMRSELAEVKFTSDKKLDDAHALEIGLEE
KYLEVEQKLHAADAKLAEASRKSSDVDRKLEDVEAREHKLQKEYLLFDSGRKLHEKDITE
QREHLRDWEQKLQDSQKRLVETQRYLNEREDRTNEADRVLKKKEADAEEARKMIEATKKS
LKTKEEEITKRLGSLAAKEKEVLENKEKDLISREEKLNARERVEIQKLLDDHNLLISSKK
EEFELDLEKRRKSLSKEIECKIREVEKKRREIDSMEEQITKREQALQ
>musa2
DREALVQRILALEKELHEYQYNMGLLLIEKKDWALKYEEIRQALMDVEETLKREKLAHLA
SISEFEKREENLQKALGVEQQCVSDLEKALREMHSELAEVKFTSDKKLDDAHALEAGLEE
KYLEVEQKLHSADAKLAEASRKSSVANRKLEDVEAREHKLQKEYLSLSSEWKLHEKGITE
QREHLCYWEKKLQDSQKRLVESQRFLNQREYQANEADRFHKKKEAELEESRKMIEATKKS
LKSKEEDITIKLRSIAAKEKEILGKKEKDLFSREETLNARERVEIQKLLDDHNALLISKR
EEFELNLEKRRKSFDADLEGKVHEVEEKKREIDCMEDQVKKREQALE
>musa3
DREALVQRITELEKELHEYQYNMGLLLIEKKESIARYEEVRQALAEAEEILKREQTAHLI
AISEYEKREETWLKDLGVEKQKVSALEKDLREVRFEISEVKFSSERKLSEAHALETGLEE
KYLEIEARMHAADAKLAEAGRRNSETNRKLEDIEAHERKLQRDCLSLTSERKAHEKDLLE
QREHLFDWEKRLQESQRRLVEEQRLLNEREDSANEADHILKKKETELEETREAIEASKRS
LKLEEDDITIRLSSLASKEKEALERKERELFAREEKLNSRERVEIQKLLDDHNAMLDSKK
HEFELEMENQRKSFEEEMKAKIDEVEEIKKELDHKEEQILEREHALE
>musa4
DREALVRRISELEKELYQYQYHMGLLLIEKKEWAVKYDKLRQEMSEAAQLQKCMQAAHIV
AVAEFEKSEGNLRRAMGFQRQSIIHLEKALNDMHAEIAEVKLDSQKKLSEAHTLEATIEE
KCLEIKEKQHSLDARLAKVSRKSSEVDRRLEDVEAREHELFKQTSSFIAEKKAFEKDLSR
QRENLRAWEQQLQDNQKKLGKWHSTENQREMETNERDNTFRKKEKELEEARKTLEISNEL
IKLKEEDMCMRIGALDAKEKEALEKKENELLAIEEQLNNKERVEIQKITDFHNSILESQK
DEFELETEKKKRAVDEQLQGRIEEVAHKEIILENRERELFKKEQLLE
>musa5
DKAALISYITKLESEIYEYQHHMGLLILERKELVSKYEQVKASSDSAEIAYKREEAKRSS
ALAEARKRELNLEKLLGIQKECVANIEKALHDNLVESAERKLGYESKIAEAHAMMTAAQE
KLDEAEKKLLAAESLQAEANRTRNTAIRTLDDVEAREDELRRRLATFKSQCDAKENEISI
QRQALYESQKTLHQQQERFLEGQTLLNQREEYIFERTKELNRIEKELEESKANIEEESRT
LKLERSNLDLEIAALRNREEVILDKRERELLILQEKIACREHDEIQRIMEEHQSILEKKK
SELEADIEQRHLLLKNELEAKKIACEIREADLCSREISLQEKEHAIE
>eutrem1
DPRILPEKVSELEKELFEYQHNLGLLLIEQKEWSSKYEELQQEFEEVNECLKRERNAHLI
AVADVEKREEGLRKALGIEKQCAVDLEKALRELRSENAEIKFTADSKLTEANALVRSVEE
KSLEVEAKLRAVDARLAEVSRKSSEVERKSKEVEARESSIQRERFSYIAERDADEATLSK
QREDLREWERKLQEGEERVAKSQMIVKQREDRANEGDKIIKQKGKELEEAQKKIDAANLA
LKKKEDDISSRIKALAFREQETIETKERELLALQEKLDAREKVAVQQLIDEHQAKLEAAQ

```
REFELEMEQKRKSIDDSLRSKVVEVEKREAEWKHMEEKVAKREQALD
>eutrem2
DREALLEKISTLEEELYGYQHNMGLLLMENKEWVAKHEELNQAFQEAQEILKREQSSHLY
ALTTVEQREENLRKALGLEKQCVEELEKALREIQEENNKIRLTSEAKLAEANALVASVTG
RSSDVENKIYSAESKLAEATRKSSELEMRLKEVETRESVLQQERLSFAKERESYEGIFHK
QREYLHEWEKKLQEKEESIPEQKRSLNQREEKVTEKEKNLKLKAKQLEEWDRKVELSVSK
SKETEEDMNKRLQELAAKEKESLVAKESELRALEEKLIVREGTEIQKLIDDQKEALAAKM
LEFELECEERRKSLDRELQKKIEEVERQRVEINHSEEKLQKRNEALN
>eutrem3
XXXXXXXXXXXXXXXXXXXXXXXXXXXXXXXXWTSTNEELQQAIDEASEILKRERMSKLI
ALNEAEKREENLRKALISEKQFAAELERDLKYLQQEHSEVKSTSEAKLAEANALVMGIKE
NSLEVDRKRAIAEEKLSVINRKSSELEKKLKEVETREKVLQREHLSLVTEREAHEA----
---------KKLTLEEDRLSEAKRSVNHIEERIIESEKTIKKKEKILK------------
--EKEEFINSMLNDISMKEKAFIDMKEKELHELEEKLIVREQMETGKLFDDQNAVLDSRK
HEFEMELEQMRISLNEELERKKSEVEQLEVEISHKGEKLAKKESTLE
>eutrem4
DKAALIAYIAKLESEVYDYQHNMGLLILEKDELLSKYEEVKASVNEADLAHRRDQSAYVS
ALAEAKKREEDLKKDVGIAKECISSLEKTLHEMRAECAETKVSAGSKMSEAHLMIEDALK
KYADAEAKMRAAEALQAEANRYHRIAERKLKEVESREDDLARRLASFKSDSETRENEIDI
ERQTLSERRKSLQQEHERLLDAQASLNQREDHIFGRSQELAELEKGLESAKTTFEEERRA
LEDKISNLEIALASLAKREEAVVLKKEQELLVAEEKIATKESELIQKVLANQEVILRKRK
SDVEAELESKCKLVEDEIESKRRAWELREVDIRQREDLVGEKEHDLE
>ambor1
EKDALLSHLAKLEDELFDYQYNMGLLLIEKKEWTSKYDDLKQALVEAQESLKREQASHLI
ALSEVEKREENLRKALGVEKQCVADLENALHEMRTEFAEIKFTADKKLAEARSLVASIEE
KSLEAEAKLRSADAQLAEASRKSSNLERQLQEIETRESVLRRERQSLKAEREAHETTFNR
ERENLRNWERKLKEGQERLVESQGLLNQREELANEKEMFLTKKEKDLEVAWAKFEKGNLN
LKDKEVEMNMRLRSLTAQEEEALDEAQQELHLLQEKLNAREKEGIQKLLDEHNAVLELRK
REFDIEMDQKRKSLEEEFEKKQVVVEQKLVEVDLKEEKINKKEQLLE
>ambor2
DKAALISYITKLESEMFDYQYHMGLLILEKKEWTSKYEQIKASADSAEDKYKRDQAALLS
ALAEAEQREENLQRALGVEKECVASIEKALHEMRAECAETKVAAETKLAEVRCLVEDAQK
KLLAVETKQHTVEALQTETSWQHAVAERKLKEVEAREDELRRQQVSLKSEMEAKEKDLLN
EKESLRELEKVIQQGQEKLFEGQTLLNQREQCIKERSDRLSRLEKEVQAATVKLQEDLEI
LKEEKANLCLTSVALTTREEAIIDKKEQELLLLQEKLTSREQDEIRRLTIEHQTAIELRE
SQFEEELHEKHKSFEADLGLQRHALDLRDAELKHQEDLMHKDKHELD
>Physc1
DRAALHARIAALEAELYDYQYNMGLLLLQRKTWTSQADDLKAAVADAQETLQREKAAHLL
ELSEVMRREEAAKKALETEKQCVADLEKALKEFQTDESEVRQAADKQLAQARELVASIEE
RSVQADLKLAQVQVLRADANRKLQESELRLQEVEAREVALRRERHSLMADVDARKEQVAS
EEASLKEWEKRLEEGRARLQEGERLLNERENSLKQRDEALKQTSRELAETRSYIENERAL
IKQTDADLNARVISLSERERTLILTKEQDLLLAEERIAEQTREEMEALLKIHTSDVDSKK
AELEEELRSVRKTLKEEVETLKLAAEAREARSRHLETAITAREEELK
>Physc2

DRAALHARIAALEAELYDYQYNMGLLLLQRKTWSSQVDELKAAVADAQGTLQREKAAHLL
ELTEVIRREEAAKSALETEKQCVADLEKALKEIQADESEVRQAADKQLAQARELVASIEE
RSIQADLKLAQVQVVRADANRKLQESEHRLQEVEAREVALRLERHSLIADVEARKEQVES
EEASLREWEKRLEDGRMRLQEGERLLNERENSLKERDEALKQINREVAEARSYIEKERVL
IQKSDVDLNARAVAFSEKERALILKKDQDLILAEERIADKTREEMEKFLKSHTSDVDSKK
AELEEELRSVRKVLKEEVETLKLVAESREARLRHLEAAITAREEELE
>selag
DRNALLAHISKLETELYDYQYQMGLLLLESNKLRGESERLKSVIDETRDGLKREQSAHMI
ALQEAERREESLKRAVTTEKKCVADLEKALKEMHEEVAEAKAAAATQFQQGKATAMSAEE
KLLEAESKLHSAEALLAKANRKHADAERKLQEVESREDALRRQRHSFLAECEAHKLELEH
EKQNLKGWERTLEESQARFVENEKLLNKREEYMQQRDDALTKLERDLDEARKVLEKDRSA
```

LRQEQAEYSALLSALSLREEAAATKKEQEILLLQEKLASRDRXXXXXXXXXXXXXXXXXX
XXXXXXXXXXXXXXXXXXXXXXXXXXXXXXXXXXXXXXXXXXXXXXX
>pohlia
DRAALHARIAALEAELYDYQYNMGLLLLQRKTWTSQVDELKAAVVDAQETLQREKAAHLL
ELSEVMGREEAAKNALETEKECVADLEKALKEVQADESEVRQAADKQLAQARELVASIEE
RSVQADLKLAQVQVVRADANRKLQESEHKLQEVEAREVALRRERHSLMADVGARKEQVEA
EEASLREWEKRLEEGRVRLPDGERLLNEREDALKMRDEALQQTSRELAETRSFLEKERAL
IQQSDVDLNARVVSLSERDRALVLKKEQDLILFEERIADKTREEMERLLKSHTSDVDSKK
AELEEELRSVRKTLKEEVESLTLAAEAREARLRHLEAAITAREAEFE
>nitell
ERNFMVDHIKQLEDELYKSQYHMGLLLMERKSLLADVAKLDHTAKEYEEIIKREREEWSM
TRIEYEEIETTLKENLLAEQQCVKEVEKAIRDVQKEKDETTRLAELRFSEARALMSEVEK
KEHGVEISRKNVEATRAEFTRKKVEIESRARELETKESLVRREREQVRIE----SSELEM
KRQELIERENVAREWEAKLASREQSVRKMEDRMKGREDTVLRKERDVEEGMARLDGKRKG
LSEMEDDLRLREKDLAMKEESLLSMKDEEMRSAKERLLESEHDRRQDIIEQQKQQMSSKE
NALELDLERKDRDLREEIEEQKALLNDSRRDAQQWEAKLEMRERSIE
>nithil
XXXXXXXXXXXXXXXXXXXXXXXXLFLMEKRSLLAETSKSDNTVEGYEEIIKRRREEW--
SIMDFEELETQLKDTYLKEQR---DVEEVIKDXXXXXXXXXXXXXXXXXXXXXXXXXXXX
XXXXXXXXXXXXXXXXXXXXXXXXXXXXXXXXXETKENLLRREREQVRIL----GADMET
KRQELTERENIAREWEAKLATREQGVKKTEERMKAREDTVLRKERDTEEGLARLEGKRKG
LSEMEDDLRLREKDLSTKEEGLLNMKDEELRSAKERLLESEHDRRQEIIDQQKQQLSSRE
NAVELDIERKERDLREEIEEQKALLNDSRRKHSSGKRSVELRERNXX
>spirog
ERDALSIRLQELELEYGDYKLQTEAAFDERDRCLSQFSTIQSQLKEMVEKFKLEKNAHQS
TIASNTEKERKLRDALTHTQRTADELELALTEKNLEFDNFRQSLTSLENETIRKKCIAEE
KILECEISLKESFSLKGEYEKKKVEYESKLQETQITESNLRLEYLRLKDSQNEFQIQVSL
KEQALKEMEERSRXXXXXXXXXXXXXXXXXXXXXXXXXXXXKLISEYEQMNEEMEAKMKT
ILEKEEEMESKEKELIHFENETLENLEKRLKEMQERLNEQEKLETKMHLTSFEEALNFRQ
ESIELELESKEKEMKIKLLSLEENLKHKQVAVDEHKTALIKEINELQ
>coleo
LKEDLMNQLQSKENELKRYQFEMGKVLLENTNLRDELVAAELAAKDRELRLEAEQNEHRE
MVAQMRTREQQLHKSVEAERRCTADLERAVREMQNESMEAKKKAEVQMEESMALWKEAER
KRQEAEAQVRSAQALHREAEQKMGEIRRREEDVAAREREVNREHAKRLAEFEAEKAEVDR
RQREVASWDASLKECDGKLKEAQQRLRQLEKSLEERSASILSNEKALEHRDSVLRAREEN
AKYKEEEIEALEEKLAVQQLNTVGARERAVVEAGDRLAVAEADELSTRLLSDEAGLASQI
LKHDSDWEQRHSHLESEIEKKRSACEAAQAQLQEKEENLRKREEAVE
>klebsi
EKEELQRQLDDVERELADYRYNLGVLILEHKKCKPQIDELEKALQRTREELQKEGRSLQL
ALDDVTRREDGLRASLKAERTVIADLKQSLEEMHAKLQAEEKSSRERLARAQELEAGAVK
DRERSEEDLKGAQQLEEGLKVRESELDGKLRDAASREKALNEGEAALNAG----KRELTE
KGIKLAERKRKVEGELEE--ERKKLGREREEEELRVEALRRSVEGEREQMQKEMQAEKEA
MSTRKAEISVKEDSLAAQEKALLERKQEDANKRDQDLDQREKL----LGDEKREMLRT--
---EADLREQQKSMRARLAEQESEWQRIEAKIDARVKEIDAREAAVQ
>klebsub
XXXXXXXXXXXXXXXXXXXXXXXXXXXXXXXXXXXXXXXXXXXXXXXXXXXXXXXXXXXX
XXXXXXXXXXXXXXXXXXXXXXXXXXXXXXXXXXXXXXXXXXXXXXXXXXXXXXXXXXXX
XXXXXXXXXXXXXXXXXXXXXXXXXXXXXXXXXXXXXXXXXXGEAALKAG----KREVIE
NGIELAGRKRKVEGELEE--ERKKLEWKRQEEELRIEALRRSVEGEREQMRKEMQEEREA
LSARKADISTKEESLAAQEKALLERKHKNAIKRGQDLDQREKL----LGDEKREMLRK--
---DADLREQEKSMRARLTEQESEWRRIEAKIDARVKELDGREAAVQ

NUP-1:

>Lmaj

PSYGEQPARPPPAGVADLARPISVRELALTSLAHRDIDHLSGRELYNYASHLRESSQQYG
GYLSEAYAKRDQYRGEVARLKEELHNRYMEIDVLRREQERARDALLKVREDNAQLRQKLA
EAEGHARNMQAKISVLSGADPSLAVHFYQSQLALKDAQLRELQQECERAAAASSQRRSSS
TTAAEHGRRSANISGEKGGTEAASAALHTALEELQCLNTSLSHRLEDEHTAHKAALAAHA
EASATATADRAELLDTIAQLRQQCADMQNVEDELVAALTLRAPISKKDYAALQASYDDLT
AALAKAEAQVAQENEQQHHHSAQQAQ-EELRRSLTTGLEER----QSMQVRNE---QMQS
LVNHLEKELKLAERANQLRQEQLDAAISGNQQLAESLHVAQEQLLSATSELQDLRDTRES
LERSEDVQATLERTVARLAEEEELVEQLNYDASELSDARAALTEEVTRLQKALAAAVTDK
ADVLAQLLATQKELDRAAEVQDAQYRDEELLQNALATTTSAFDVQTKQVRLGMAHVVDMS
RAFCRVAEVAVQRSATGANVQEILSPWARALEWVAAMEQRLETLDAAFARLMDEQAAATN
TIADLRRTLVDKEVDLTNLRSAMDALASDLISERDRADNLSAAIDDASEKIASAQAEFEE
QLRQRTAATTAEVVEARRAEARATAAQLRAEEQLAVVEKDLKEQEGVLRKLQDENHRLTR
EADRLLR-TSR-LEPSSEDAVQRFTTAPSTLEAVEQAQILQVSSLQADLLLSRRQARELE
GREATLRQACAALEQQVSELRVEAAEAEHLREDMATLRADYAELEQRYSELERMAIAAGG
GTMQELKQLRQQLKVREVELEELKARMRDLVLSKAAPELEAARRQEALRESLSGITTQLA
ATQAQYLLSLSSSRPSPQDVLTSRIEHLQTLVNDRDVALEKVQAAQLESRAAMDTLEDQL
AAKTAALERAEGLVAEYADSVNAFTMAAVVGRGNRGAGPTASASVPATTRKRAR
>Linf
PSYAAQPARPPPAGVADLARPISVRELALTSLAHRGIDHLSGSELYNYASHLQESSRQYS
GYLSAAYAKRDQYRGEVAHLKEELHNRYVEIDALRREQERASDALLKVREDNAQLRQKLA
EAEGHARNMQAKMSVLSGADPSIAVHFYQSQLALKDAQLRELQQECERAAAASSQRRSSS
TTAAEHGRRSANISGEQDGAEAASAALHTALEELQCLNTSLSHRLEEEHTAHKAALAAHA
EASATATADRAELLDTIAQLRQQCADMQSAEDELVAALTQRAPISKKDYASLQASYDDLT
AALAKAEAQVAQEKEQQHHHSAQQAQ-EELRHSLTTGLEER----QSMQARNE---QMQS
LVNNLERELELAERANQLRQEQLDVAISGNQQLAESLHVAQEQLLSATSELQDLRDTRES
LERSEDVQATLERTVARLAEQEALVEQLNYDASELNDARAVLTDEVTRLQKALAAAVSDK
ADVVAQLLATQKELDRAAEAQDAQYRDEERLQDALATTTSAFDVQTTQVRLGMAHVVDMS
RAFCRVAEVAVQRSATGTSVQEILSPWTRALEWVAAMEQRLEALDAAFARLIDEQAAATN
TIADLRRTLADKEVDLTNLRSAMDALASDLMGERDRADNLSAAIDDASEKIASAQAEFEE
QLRQRTAVTTAEVVEARRAEARATAAQLRAEEQLAAVEKDLKEQEAVLRKLQDENHRLTR
EADRLLR-TSR-LEPSSENAAQRFTTAPSTLEAIEQAQILQVSSLQADLMLSRRQARELE
GREATLRQACAALEQQVSELRAKAAEAEHLREDLATLRADYVELEQRYSELELMATAAGG
GTMQELKQLRQQLKVREAELEEVKARMRDLVLSKAAPELQAARRQEALRESLSGITTQLA
ATQAQY-LNLSSSRPSPQDVLNSRIEHLQTLVHDREVALEKVQAAQLESRAAMDTLEDQL
AAKTAALERAEGLVAEYADTINAFTMAAVARRGNRGAVSTAGASVPATTRKRAR
>Ldon
PSYAAQPARPPPAGVADLARPISVRELALTSLAHRDIDHLSGSELYNYASHLQESSRQYS
GYLSAAYAKRDQYRGEVAHLKEELHNRYVEIDALRREQERASDALLKVREDNAQLRQKLA
EAEGHARNMQAKMSVLSGADPSIAVHFYQSQLALKDAQLRELQQECERAAAASSQRRSSS
TTAAEHGRRSANISGEQDGAEAASAALHTALEELQCLNTSLSHRLEEEHTAHKAALAAHA
EASATATADRAELLDTIAQLRQQCADMQSAEDELVAALTQRAPISKKDYASLQASYDDLT
AALAKAEAQVAQEKEQQHHHSAQQAQ-EELRHSLTTGLEER----QSMQARNE---QMQS
LVNHLERELELAERANQLRQEQLDVAISGNQQLAESLHVAQEQLLSATSELQDLRDTRES
LERSEDVQATLERTVARLAEQEALVEQLNYDASELNDARAVLTDEVTRLQKALAAAVSDK
ADVVAQLLATQKELDRAAEAQDAQYRDEERLQDALATTTSAFDVQTTQVRLGMAHVVDMS
RAFCRVAEVAVQQSATGTSVQEILSPWTRALEWVAAMEQRLEALDAAFVRLIDEQAAATN
TIADLRRTLADKEVDLTNLRSAMDALASDLMSERDRADNLSAAIDDASEKIASAQAEFEE

QLRQRTAVTTAEVVEARRAEARATAAQLRAEEQLAAVEKDLKEQEAVLRKLQDENHRLTR
EADRLLR-TSR-LEPSSENAAQRFTTAPSTLEAIEQAQILQVSSLQADLMLSRRQARELE
GREATLRQACAALEQQVSELRAKAAESEHLREDLATLRADYVELEQRYSELELMATAAGG
GTMQELKQLRQQLKVREAELEEVKARMRDLVLSKAAPELQAARRQEALRESLSGITTQLA
ATQAQY-LNLSSSRPSPQDVLNSRIEHLQTLVHDREVALEKVQAAQLESRAAMDTLEDQL
AAKTAALERAEGLVAEYADTINAFTMAAVARRGNRGAVSTAGASVPATTRKRAR
>Lmex
PSYAAQPARPPPAGVADLARPISVRELALTSLAHRDVDHLSGNELYNYASHLQESSKQYS

GYLSEAYAKRDQYRGEVARLKEELHNRYMQIDVLRREQARATDALVKVREDNAQLRQKLA
EAEGHTRNMQAKMSVLSGADPSIAVHFYQSQLALKDAQLRELQQECERAAAASSQRRSSS
TTAAEHGRRSANISGEQERAEAASAALPTALEELQRLNISLSHRLEEEHAAHKAALAAHA
EASATARADRAELLDTIAQLRQQCADMQSAEDELVAALTQRAPISKKDYAALQASYDDLT
AALAKAEAQVAQEKEQQHDHIARQAE-EELRHLLTTGQEER----QSMQARNE---HMQS
LVYHLERELELAERANQLRQEQLDAAISGNQQLAESLHVAQEQLLSATAELQDLRDTCES
LERSEDVQATLERTVARLAEEEALVEQLNYDASELNVARAALSDEVTRLQRALAVALTDK
SDVVAQLLATQKELDRAAELQDAQYRDEERLQDALATTTSAFDVQTKQVRLGMAHVVDMS
RAFCRVAEVAVQWSATGASVQEMLSPWSRALEWVAGMEQRLEALDVAFARLMDAQAAAMN
TIADLRRTLADKEADLTNLRSAMDALASDLMGERDRADNLSAAIDDASEKIGSAQAEFEE
QLRQRTAATTAEVVEARRAEARATAAQLRAEKQLAAVENDRKEQEAVLRKLQDENHRLAR
EADRLLR-TSR-LEPSSEDAVQRFTTAPSTVEAAEQAQILQVSSLQADLMLSRRQARELE
GREATLRQACAALEQQVNELRVEAAEAEHLREDLATLRADYAELEQRYSELERMATAAGG
GTMQELKQLRQQLKVREAELEELKARMQDLVLSKAAPELEAARRQEALRESLSGITTQLA
ATQAQYLLSLSSSRPSPQDVLTSRIEHLQTLVHDREVALEKVQAAQLESRAAMDMLEDQL
AAKTAALERSEGLVAEYADTINTFTMTAVVRRGNHGARPTAGASMPAATRKRTR
>Lbra
PSYTAQPARPLPAGVADLARPISVRELALMSLAHRDIDNLSGDDLYNYASHLQGASKQYS
KHLLEAYTRRDQYRGEVARLKEELHSKYLQIDVLRREQERASEALVKVREDNAQLHQKLA
ESEGHVRNMQAKVSVLSGADPSAAVHFYQSQLVLKDAQLRELQQQCEREAAASSQRHSSS
ATAAEHGRRSINVSGEHNGSEAASTALHTALEELQHLNTSLSRRLEEEHAAHKAALAVHA
EENATATADRAELLNTISQLRQQCTEMQSTEDELVAALTQRAPISKEGYAALQTSYNDLT
AALAKAEDQIAHVKEQQYHQSAQQAQ-EELRHSLTTGLEER----QELQARNE---QMQS
LATDLQRELELAERANQLRQEQLDAAVSGNQQLAEHLRTTQEQLLGATAELQDLCNTRGS
LERSEGVQSTLTRTVDRLAEQEALLEQLNRNASELNDARAVLTGEVTRLQAALAAAVTDK
SDVMAQLVATQRELDRAVEQQDAQYCEEVRLQDALTTTMSAFGVQTRQVQLGMAHVVDMS
KAFYRVVEVVAKGAATATSVREILLPWSRALEWVASMEERLESLGAAFARWIWEQAAAAS
TIADLRSALADKEVELNNLRSAMDTLANDAMAEQDRADNLSAAIGDATEKIALVQAEFEE
QLRQRTVATTAEVVEARRAEERARAAQLRAEEHLTVVENELKEQQAVLRKLQDENHRLTR
EADRLRR-SSRHLESDSVTEVESFTTAASAVEVVEQAQFLQVFSLQEDLMLSRRQARELE
GREATLRQTCATLEQQVSQLRVEATDAEKLREELATLRTDYAELEERYDQLERMTTAAGG
GTMQELKQLRQQLKARETELDELKARVRGLVLSKVAPELEAARRQEALRESLSGITTQLA
ATQAQYHLSLSASSPSSQDLLTSRIEHLQILVHDHQEAFEKVQAAQLESRATMDTLEDQL
AAKTAALERAEGLVAEYADTINVLTMTAVVGRGSGGAHLSVGVTMIAKARKRAR
>Lger
PSHAAQPARPPPAGVADLARPISVRELALTSLAHRDIDHLSGRELYNYASHLRESSQQYG
GYLSEAYAKRDQYRGEVARLKEELHNRYMEIDVLRREQERARDALLKVRDDNAQLRQKLA
EAEGHARNMQAKISVLSGADPSLAVHFYQSQLALKDAQLRELQQERERAAAASSQRRSSS
TTAAEHGRRSANISGEQDGTEAASAALHTALEELQCLNTSLSHRLEDEHTAHKAALAALA
EASATATADRAELLDTIAQLRQQCVDMQSVEDELVAALTLRAPISKKDYVALQASYDDLT
AALAKAETHVAQENEQKHLHSAQQAQ-EELRHSFTTALEER----QSMQARHE---QMQS
LVNHLERELKLAERANQLRQEQLYAAISGNQQLAESLHVAQEQLLSATSELQDLRNTRES
LERSEDVQATLERTVARLAEEEALVEQLNCDASELNDARAALTEEVTRLQKALEAAVTDK
ADVLAQLLATQKELDRAAEVQDAQYRDEERLQNALATTTSAFDVQTKQVRLGMAHVVDMS
RAFCRVAEVAVQQPATGASVQEMLLPWTRALEWVAVMEQRLETLDAAFARLMDEQAAATN
TIADLRRTLADKEVDLTNLRSAMDALANDLISERDRADNLSAAIDDASEKIASVQAEFEE
QLRQRTAATTAEVVEARRAEARATAAQLRAEEQLAAVEKDLKEQEGVLRKLQDENHRLTR
EADRLLR-TSR-LEPSSEDAAQRFTTTPSNLEAVEQAQILQVCSLQADLLLSRRQARELE
GREATLRQACAALEQQVSELRVEAAEAEHLREDLATLRADYAELEQRYSELERMAIAAGG
GTMQELKQLRQQLKVREVELEELNARMRDLVLSKAAPELEAARRQEALRESLSGITTQLA
ATQAQYLLSLSSSRPSPQDVLTSRIEHLQTLVNDREVALEKVQAAQLESRAAMDTLEDQL
AAKTAALERAEGLVAEYADSVNAFTMAAVVRRGNRGAGPTASASVPATTRKRAR
>Lama
PSYAAQPARPPPAGVADLARPISVRELALTSLAHRDVDHLSGNELYNYASHLQESSKQYS
GYLSEAYAKRDQYRGEVARLKEELHNRYMQIDVLRREQARATDALVKVREDNAQLRQKLA
EAEGHTRNMQAKMSVLSGADPSIAVHFYQSQLALKDAQLRELQQECERAAAASSQRRSSS

TTAAEHGRRSANTSGEQERAEAASAVLPTALEELQRLNTSLSHRLEEEHAAHKAALAAHA
EASATARADRAELLDTIAQLRQQCADMQSAEEELVAALTQRAPISKKDYAALQVSYDDLT
AALAKAEAQVAQEKEQQHDHIARQAE-EELRHLLTTGQEER----QSMQTRNE---HMQS
LVYHLERELELAERANQLRQEQLDAAISGNQQLAESLHVVQEQLLSATAELQDLRDTRES
LERSEDVQATLERTVARLTEEEALVEQLNYDASELNVARAALSDEVTRLQKALAVALTDK
SDVVAQLLATQKELDRAAELQDAQYRDEERLQDALATTTSAFDVQTKQVRLGMAHVIDMS
RAFCRVAEVTVQWSATGASVQEMLSPWSRALEWVAGMEQRLEALDVAFARLMDAQAAAMN
TIADLRRTLAEKEADLTNLRSATDALASDLMGERDRADNLSAAIDDASEKIASAQAEFEE
QLRQRTAATTAEVVEARRAEARATAAQLRAEKQLAAIENDRKEQEAVLRKLQDENHRLAR
EADRLLR-TSR-LEASSEDAVQRFTTAPSTVEAVEQAQILQVSSLQADLMLSRRQARELE
GREATLRQACAALEQQVNELRVEAAEAEHLREDLATLRADYAELEQRYSELERMATAAGG
GTMQELKQLRQQLKVREAELEELKVRMRDLVLSKAAPELEAARRQEALRESLSGITTQLA
ATQAQYLLSLSSSRPSPQDVLTSRIEHLQTLVHDREVALEKVQAAQLESHAAMDMLGDQL
AAKTAALERSEGLVAEYADTINTFTMTAVVRRGNHGARPTAGASMPAATRKRTR
>Ltur
PSHAAQPARPPPAGVADLARPISVRELALTSLAYRDIDHLSGRELYNYASHLRESSQQYG
GYLSEAYAKRDQYRGEVARLKEELHNRYMEIDVLRREQERARDALLKLQEDNAQLRQKLA
EAEGHARNMQAKISVLSGADPSLAVHFYQSQLALKDAQLRELQQERERAAAASSQRRSSS
TTAAEHGRRSANISGEQDGTEAASAALHTALEELQCLNTSLSHRLEDEHTAHKAALAAHA
EASATATADRAELLDTIAQLRQQCADMQNVEDELVAALTLRAPISKKDYAALQASYDDLT
AALAKAEAQVAQEKEQQHLHSAQQAQ-EELRRSLTTGLEER----QSMQVRNE---QMQS
FVNHLERELKLAERANQLRQEQLYAAISGNQQLAESLHVAQEQLLSATSELQDLRNTRES
LERSEDVQATLERTVARLAEEEALVEQLNYDASELNDARTALTEEVTRLQKALAAAVTDK
ADVLAQLLATQKELDRAAEVQDAQYRDEERLQDALATTTSAFDVQTKQVRLGMAHVVDMS
RAFCRVAEVAVQQSATGASVQEMLSPWTRALEWVAAMEQRLETLDAAFARLMDEQAAATN
TIADLRRTLADKEVDLTNLRSAMDALANDLISERDRADNLSAAIDDASEKIASAQAEFEE
HLRQRTAATTAEVVEARRAEARATAAQLRAEEQLAAVEKDLKEQEGVLRKLQDENHRLTR
EADRLLR-ASR-LESSSEDAAQRFTTAPSTLEAVEQAQILQVSSLQADLLLSRRQARELE
GREATLRQACAALEQQVSELRVEAAEAEHLREDLATLRADYAELEQRCSELERMAVAAGG
GTMQELKQRRQQLKAREVELEELKARMRDLVLSKAAPELEAARRQEALRESLSGITTQLA
ATQAQYLLSLSSSRPSPQDVLTSRIEHLQTLVNDREVALEKVQAAQLESRAAMDTLEDQL
AAKTAALERAEGLVAEYADSVNAFTMAAVVRRGNRGAGPTASASVPATTRKRAR
>Larab
PSHAAQPARPPPAGVADLVRPISVRELALTSLAYRDIDRLSGRELYNYASHLRESSQQYG
GYLSEAYAKRDQYRGEVARLKEELHNRYMEIDVLRREQERARDSLLKVREDNAQLRQKLA
EAEGHARNMQAKISVLSGADPSLAVHFYQSQLALKDAQLRELQQERERAAAASSQRRSFS
TTAAEHGRRSANISGEQDGTEAASAALHTALEELQCLNTSLSHRLEDEHTAHKAALAAHA
EASATATADRAELLDTIAQLRQQCADMQNVEDELVAALTLRAPISKKDYAALQASYDDLT
AAHAKAEAQVAQEKEQQHLHSAQQAQ-EELRRSFTTGLEER----QSMQVRNE---QMQS
FVNHLERELKLAERANQLRQEQLYAAISGNQQLAESLHVAQEQLLSATSELQDLRNTRES
LERSEDVQATLERTVARLAEEEALVEQLNYDASELNDARAALTEEVTRLQEALASAVTDK
ADVLAQLLATQKELDRAAEVQDAQYRDEERLQDALATTTSAFDVQTKQVRLGMAHVVDMS
KAFCSVAEVAVQQSATGASVQEILLPWTRALEWVAAMEQRLEMLDAAFARLMDEQAAATN
TIAELRRTLADKEVDLTNLRSAMDALANDLISERDRADNLSAAIDDASEKIASAQAEFEE
QLRQRTAATTAEVVEARRAEARATAAQLRAEEQLAAVEKDLKEQEGVLRKLQDENHRLTR
EADRLLC-TSR-LEPCSEDAAQHFTTAPSTLEAVEQAQILQVSSLQADLLLSRRQARELE
GREATLRQACAALEQQVSELRVEAAEGEHLREDLATLRADYAELEQRYSELERMAIAAGG
GTMQELKQLRQQLKVREVELEELKARMRDLVLSKAAPELEAARRQEALRESLSGITTQLA
ATQAQYLLSLSSSRPSPQDLLTSRIEHLQTLVNDREVALEKAQASQLEYRAAMDTLEDQL
AAKTAALERAEGLVAEYADSVNAFTMAAVVRRGSCGAGPTANASVPATTRKRAR
>Ltrop
PAYAAQPARPPPAGVADLARPISVRELALTSLAHRDIDRLSGRELYNYASHLRESSQQYG
GYLSEAYAKRDQYRGEVARLKEELHNRYMEIDVLRRQQERASDALLRVREENAQLRQKLA
EAEGHARNMQAKMSVLSGADPSLAVHFYQSQLALKDAQLRELQQECERAAAASSQRRSSS
TTAAEHGRRSANISGQQDGTEAASAALHTALEELQCLNKSLSHRLEDEHTAHKAALAAHA
EASATATADRAELLDTIAQLRQQCADMQNAEDELVAALTLRAPISKKDYAALQASYDDLT

AALAKAEAQVAQEKEQQYHHSAQQAQ-EELRHSLTTGLEER----QSMQVRNE---QMQS
LVNHLERELELAERANQLRQEQLDAAISGNQQLAESLHVAQEQLLSATSELQDLRNTRES
LERSEDVQATLERTVARLAEEEALVEQLNFDASELNDARAALTEEVTRLQKALAAAVTDK
ADVVAQLLATQKELDRAAEVQDAQYRDEERLQDALATTTSAFDVQTKQVRLGMAHVVDMS
RAFCRVAEVAVQRSATGASVQEMLSPWTRALEWVAAMEQRLEALDAAFARLMDEQAAATN
TIADLRRTLADKEVDLTNLRSAMDALASDLMSEQDRADNLSAAIDDASEKIASAQAEFEE
QLRQRTEATTAEVVEARRAEARATAAQLRAEEQLAAVEKDLKEQEGVLRKLQDENHRLTR
EADRLLR-TSR-LEPSSEDAAQRFTTAPSTLEAVEQAQILQVSSLQADLLLSRRQARELE
GREATLRQACAALEQQISELRVEAAEAEHLREDLATLRADYAELEQRYSELERMATAAGG
GTMQELKQLRQQLKVREVELEELKARMRDLVLSKAAPELEAARRQEALRESLSGITTQLA
ATQAQYLLSLSSSRPSPQDVLTSRIEHLQTLVNDREVALEKVQAAQLESRAAIDTLEDQL
AAKTAALERAEGLVAEYADRINAFTMAAVVRRSSHGAGPTAGASVPATTRKRAR
>Laeth
PPYAAQPARPPPAGVADLARPISVRELALASLAHRDIDRLSGRELYNYASHLRESSQQYG
GYLSEAYAKRDQYRGEVARLKEELHNRYMEIDVLRRQQERASDALLRVREENAQLRQKLA
EAEGHARNMQAKMSVLSGADPSLAVHFYQSQLALKDAQLRELQQECERAAAASSQRRSSS
TTAAEHGRRSANISGEQDGTEAASAALHTALEELQCLNTSLSHRLEDEHTAHKAALAAHA
EASATATADRAELLDTIAQLRQQCADMQNAEDELVAALTLRAPISKKDYAALQASYDDLT
AALAKAEAQVAQEKEQQHHHSAQQAQ-EELRHSLTTGLEER----QSMQVRNE---QMQS
LVNHLERELELAERANQLRQEQLDAAISGNQQLAESLHVTQEQLLSATSELQDLRDTRES
LERSEDVQATLERTVARLAEEEALVEQLNFDASELNDARAALTEEVTRLQKALAAAVTDK
ADVVAQLLATQKELDRAAEAQDAQYRDEERLQDALATTTSAFDVQTKQVRLGMAHVVDMS
RAFCRVAEVAVQRSATGASVQEMLSPWTRALEWVAAMEQRLEALDAAFARLMDEQAAAAN
TIADLRRTLADKEVDLTNLRSAMDALASDLMSEQDRADNLSAAIDDASEKIASAQAEFEE
QLRQRTAATTAEVVEARRAEARATAAQLRAEEQLAAVEKDLKEQEGVLRKLQDENHRLTR
EADRLLR-TSR-LEPSSADAAQRFTTAPSTLEAVEQAQILQVSSLQADLLLSRRQARELE
GREATLRQACAALEQQISELRVEAAEAEHLREDLATLRADYAELEQRYSELERMATAAGG
GTMQELKQLRQQLKVREVELEELKARMRDLVLSKAAPELEAARRQEALRESLSGITTQLA
ATQAQYLLSLSSSRPSPQDVLTSRIEHLQTLVNDREVALEKFQAAQLESRAAMDTLEDQL
AAKTAALERAEGLVAEYADSINAFTMAAVVRRGNHGAGPTAGASVPATTRKRAR
>Lenri
HSHPARLARPPPAGVADLARPISVRELALTSLAHRDIDHLSRNDLYNYASHLQDASKQYS
GYLLEAYTKRDQYRSEVAHLKEELHNRYMQIDVLRREQERASDALLSVREENAQLREKLA
EAEGHARNMQAKMSVLSGADPSVAVHFYQSQLALKDAQLRELQQGCERAAAASSQRRGSS
TTAADRGRLSTNISSEQDGTDAASAPLHITLEELQCLNTSLGRRLEEEQISHKAALVAHA
EASATAAADRAQLLDTIAQLRQQCTEMQAAEDELVTALTQRAPVSKKDYEALQASYDDLT
AALAKAEAHVGQEKAQQHHQSAQNAQ-EELRHSLSIGLEER----QALQARYE---HMES
LVNHLQRELELAERANQLRQEQLDAALNGNQQLAEHLRVAQEQLLSATSELQDLRKTHGD
LERSEEMQATLECTLTRLAEQEALVERLNREALEFHAQRPALEREVAELRVALAVAMTDK
VDVVAQLVATQKELDRAAEVHDEHYREEERLHEALSTTTSAFAVQTAQVRLGMAHVVDMS
RAFCGVAEAVAQRAASVKCLREMLLPWTQALEWVAAMEKRLDALDAAFARWMDVQSAAAN
IIAELRSALAEREAEVNSLRSAMDALATELMDEQDRADNLSAAIDDATEKIASAQAEFEE
QLRQRTAATTAEVVEARRAEARATAAQLRAEERLTVVEDDLKEQQALLRNLQDENRRLTR
KADCLLH-SSRLTDPDSEGAAKRFATAASAVEAVEEALILQVSSLQADLMLSRRHARELE
GREATVRQACLALEQQVSELRVEAADTGQLREDFAALRADYTDLEQRYDELERMTTAAGG
GTMQELKHLRQQLKVQEAELCESKARLRGLVLRKATPELAATRRQEALRESLSGITAQLA
ATQAQYPLNRSAPSPSSQDVLTSRIEHLQLVVRNREMALDKVQAAQLESRAAADTLEDQL
AMKTAALERAEGLVAEYADTIDTLTMTVLAGRGSRGARPTAGATMRATTRKRDR
>endotr
SSYSAPPAHSLPAGVADLARPISVRELALMSLAQRDIDRLSSDELYIYASHLQDASKQFT
AHLQEAYAKRDQYRSEVAHLKQELQSRYVEVDLLRRERERASKALLGVHEENTTLREQLA
EVEGRLRNMQAKMSVLSSDDSSLALHFYQSQLTLKEAQMRELQQDYERVAAASLQRCSTS
TAGAASARHSINIPVEQDSEEVVAVELRATLEELRSLNASLSHRLEEERAAHDAALAAHT
EAIAAAAADRTELLDTIFQLRQRCTEMQASEDELVAALTQRAPISKSDYAVFQSSYNDVT
AALAKAEAQITREKERHEYASARQAQ-EELHSSLALGMEEK----NTLQARNE---QMQS
LVHHLQKELELAEEANQLRQKQLDAAVNGNQGLANNLRMAQQQLLSMSSEVEDLRNTRTN

LERGEAVQRALDEALDRLSEEEVLVEELNGDAAQHSAEKMALAESVAMLQKSLSTAIVDK
EDLSAQLVATQKEVDRAIGQQDVLLDEAERLKDALEATTSAFAAQTREVRLGMAHIVDMS
NVFCGVLEVVAGAAHTTAKIGEALSPWDEALEWVATMEQRLEVLDATLARLFCEQASAKR
TIADLRVSLAEKDVELGNVRFAMDALASEAITERDRADNLSAAIDDATKKIVSVQAEFEE
QLKQRMATTTAEVVEARRAEARATASRLCAEEQLSAVEDDMKEQQALVRTLQDENRRLTH
DVDRLLR-SLRSLEGSSRIASQPNTRSPSIAETIEQAQILQVSSLQADLMRTRRQTRDLK
GREATLQLSCATLEKELSELRPRAREAEQLRGDLDALRADYAELELRHDQLERMTAAAGD
GTMQELKQLRQQLKVREAELEEQKLKFRDLLLSQATPELEVARRQEALRESLTDITTHLV
ATQAQYSLTPPPSGPSATDVLTDRMNYLQALVRDREVELEKVQAAQLESRAATDTLRDQL
VAKAAALERAEGLVAQYTDTINTLTTTAMRGGGECSGRLNGKVSAGTSSRKRSR
>leppyr
SAHSSHPPPPPPAGVPDLARPISVRDLALTSLAERNIDHLSNDDLYNYASHLQDTAKRYS
HHLAEAHAKRDMYRNLVAVQKRELQEKYVQIDRQRHEQETATAALRRLREDNTQLREQVA
TAEGKVRNIAAKLSLQNGADPSVAIRAYQAQLASKEAQVRDLMERVVHASAEASQRRSSE
--GGANPRQSDTLGAAAAAAAASEMAKLQAELNALHQRNAALQEQLAAQDKTHAEAL----
---ASMTAEHTELKETIAQLKQGHAEMQENEDALVAALTQRAPVSKTDFEAFQATYAEMQ
AALTKTEATVATLQAQQNASAVQDGYIEVLQQSLATSQAER----QELTEKNR---QLQA
LSENLQREVQLADTANRLRQQQLETAVAGGEQLAQEVQTQEARHTEVHAEIVALRARCAA
ADRGEELQRLLDTTLDRLAQEEELVKGLNDNAADLHAERAALTEEVVTVRLALETVVLER
QDIQERLDAAKLGLEQTAELYNERILEDDRLQDELDAVTQAFSAQTMQVKQGMRHMLQMS
AGFHDALEAVMALAAAVTAINAALTPWSAALEWVATMQRRVHALEGALTDFVELQEALLT
HLRRLGHALDERDYELRLLRSAVDEMANSNVAEKERADHLLEAIQDAKNHVSLAEKEFDD
QLQLRSEAVAAELVVARRAEERATDARLRAESQLAAVGAELTDALAALHKTEEERRKMSR
EVERLSRFSARLADPGAEGVSNHYTMTASAVEALEQAHLLQVSSLQQELLLSRRQIRELE
RQEVILRQANVTLEQQVSDLRADAEEAVDLREEVARLRAENADLEEKIEQLERILAASGG
GVAHEVKQLRQQLKLREAELEEEKARMQGLLVSKTAPEFAAARRQAALRESLSGITTQLA
ATQAQY--NSAATSASPLDVLSKRIDHLQSAMKERDALIEKMKAAQLQSHVTVDGLEERL
SEKAAALERANALVTHYAEALDGLRAARVSAA------ARSGTREGASHRKRTR
>Crifas
TSLPQSSRKPPPVGVPHLARPISVRDLALTSLAARNIDHLNFNDLYTYASHLQDAAKNYS
NNLSAAYTKRDMYRSQVAELKQELQERYLQVDLLRRDHERADDALLRLREDNTQLRQLLA
ESEGKLRNVSARLSLRNGADPSAAMRAYQAQLAQKDAQLRELVERLAQQTAEAAQRLGSE
A-SAAGQRRSHTLSAAASAIEAATTVLQAEIDALHQQHATLQSQLAAETASH--------
------TQERTELLETIAALRREKGDMQESEDALIAALTLRAPVSKADFESLQASYAELQ
ASLANSEASVASLREQLSKAVVQEGYTEVLQQSLTTTQAER----QELVTKTE---QLQA
LVDNLTRELQLADEANQLRQQQMEETAVANSDRWQQSQ-----------TIQELQEREAS
QVRGEELQTMLDTTLDRLAQKELLLEEVNDNAAELHKERAALADDVVTLRSALETAVVER
QDLQERLAAANEGLERTAELYNERILEDDRMQEELNAVSAAFAAQTQEMRAGLAAMTEMS
AGFCNVLETVMAPAKLISAITIALAPWSAALDWAGKMERRLDALESALLDFVQYNEAALG
HLHHIRRTMADRDDELRQLRSALDSMADATVEERERVDHLLSAIQDAKDHVARAEREFDE
QLRLRSEAITAELVVARRAEERATDARVRAETELAATAEELRESHLALRKLQEEHRKLTR
ETERLSRYSARLTDTSAETATNSYAVTASAVEALEQSNLLQVSSLQQDLLLSRRHARDLE
GREALLRQNHSTLEQEVVSLRVEAAEAADLREEVATLRADNAELEERCEQLERMLAAAGG
GTMQELKSLRTQLKLKETELADLRARMRDFLLNRSTSEFEVVRREGDLRETLSGITTQLA
ATQAQYAGGTAGDEASPQGVLSSRIDHLENVVRERDAVIEKMQASQLESRVTVDGLQENL
SEKEAALDRANTLLAQYAETIETLHSAGVAGTTTRQLPATSATTTAAAGRKRGR
>CritAcant
SASMSATAPRPPSSVPDLARPISVRDLALTSLAYRHIDHLSSDDLYNYASHLQDAAKHYS
SHLSEAYAKRDAYRSQVAELNHRLQEEYLKTDLLRRNREKADDALQKLREDNTELRHLLA
EAEGQTRNLSAKLSLQSGAEPSVVIRALQAQLAQKDAQLREMSERLLHATAELQQRRSSS
SGGGAHPRASNASSAAEQAVAAATAHLRAAMEDLQQRHDALQTQLTTQATAHAAAL----
---GRAAAQRAELVDAMTLLKQEKAELQANEDDLVAALTQRAPVSKADFESFQAAYAELQ
ASLRTAESAAATLQEQQNASAAQEGYVSALQHSLTTTQAER----QALADANR---QLQA
LVDNLQKELELAGTSLRLQQEQVASAHANEEQLAAQLQTLAAEV--MSTAQQELERRCRQ
LERGEELQGLLNTTLDRLTQTEALVKELNDNAAELHDERAALAAQLATVQAAFETVVHER
QDLQERLAAAQLGLEQTTDLYNERILEENRVQDELRAVAKAFAAQTEQVKVGIAHVLHMS

```
EGFRGAVTAVMEHATAVTAVNAAFAPWAVTMEWVAQMQQRLQKLENALLDFVEQQTMSLR
HLQTVQHALNHRDEELQLLRSALDTMASTNLDEKQRADHLLQAIQDARRRVTMAEQDFDA
QLRLRSEAVAADLVVARRAEERATDARLRAETQLETVEAELRTTQAALQKLEEEKRQLTR
ETERLSRYSTRVVDVSAESTSTHYTMNAAAVEALEQAHLLQVSNLQQELMLCRRQVRELE
GQETTLRQAYMVLEQQVVDLRTDAAEVEDLREERDTLRAENAEWEEKNAQLQRVMDATQR
STHQELSQLRQQVRVLESELEEHKTRLQVLMIGKSTPEFEALRRQEAFKENLSGLTTELA
ATQAQY------AAASPQNVYITRIEHLQTAIKERDVVIEKLQAAQLQSRVAKDSLEEKL
SEKAAALSRVSELNAQYLETIEALRSAGVSAA------TRHLVPRRMLARQRQT
>CriMelif
SPSSSTAPTPPPKEVADLARPISVRELALASLAERHIDRFSHDDLYNYASHLQDTAKRYS
NNLSEAYTARNMYREQVAQLKQELQQKTLQVDLQRRDRERCDAAVQKLREDNTQLRLQLA
EAEGQTRNIAARLSLQNGADPSVVIRAYQAQLAQRDAQLRELTERLACESSVAEARQGSG
DGGEADTRPSRALGAAAQTVAAATAELQAELNDLQQRKAALERQLTAQETAHAAAL----
---ARCAAERTELQVTIERARQEQAEMQENEDALIAALAQRAPVSKANFEALQTSFAETQ
AALTKAEAAVATLQEQQRTVAAQEGYVEVLQQALATGQAER----QELTDQNR---QLQS
LVDNLQREVELADTANQLRQQQLETALQGGEQLAEQVQAEAMHAAHMSEEIGALQERCAA
SERAEELRGVLSTTLDRLAQKEELEKQLNDNAGELAANNAALTEELATLRQTLEMALAEE
RELLERLDAANRGVEQTASMYNERILDMDRLQGELDAVTQAFAAQTEQLKSGLTRVVGMS
DGFRRAVEAAMAPAAAVAAVKAALTPWSETLDWVAATRQRIEELESALLDFVEQQTTSLA
HLRRLRHALHERDQAAVDKQAALEAVAGGAVDAQERADGLFRATEDARHRILQAEREWEE
QLQLKSQATAAELIVARRAEERANDARTRAEAQLAAAEKELKETQAALRTTQEEKQKLAL
QTERLSLLSSQQSDSSAGSRTTRYTVSSSTEEAVEQTHLLQVAGLQQELVRCRRQIRELE
SEEATLRQAYMALEQEVSDLRSDAAEVVNLRAEMATLRADYADSEERYERLRLMVDAVGD
GTAQENRRLRQELKLKDAELEEYKTRQENSLVSRGAPDFETQRLEEAFRQSMSGIATQLT
AKQAEF-----SLSASPQDVLNSRIDHLQTLVRERDAVISKMKAADLQSRVKCEGLEEKL
AEKTAALNGAEELLAQYMERLDAMRAAGLAAAVTTRA---PKVGKTVSTRKRAR
>Herpet
PRASSSSAATYRQPTPELARPISVRELAFASSAQKSIDMMSPRELYDHTSRLQDEARQTR
GYVAAAYAARDQYREEVGRLRQELHSRYLQSQALERDRGRYNDVIHKLQGDIAALRRDLD
DAEARENALQGRLARANGTRPPAALAYYATQLAAKDGQLDELEAENTALRAAQQQQ----
-------RSAPALTGE---DQRVAAQLQRALEDLQQENAVLADALATERESRAALL----
---AETAAEKTRMDADATRAAASNAALQKELDDLLEAFSSRAPITNEHYEHLRRELAEQQ
RTIAALESEAAAAEAVERADGAAELEASALRESVATLRA-------ELADALKQLAKTQE
LVAEFEKETWLAETANTVRQGLIDDLRANLQAATAAREAAEAALAAERTAHEALAGRTDA
DDRATQLDTTLADVMDRLEDTSALLARSREEMLAATDESKALEAALAAMKEQLTTSERQR
VDAEERLDAALLQNTQTAAMYNDRNDEISRTQARATDLKSRLMDRDSALELAAAHLCDCA
KAVQVALLGVLRQPTAASQIAKDLRPFAEALDWIATVEDVADDDRSALEELLNERLQGAV
RATGLEETLAATEERLRAMDEALVRLSEDKLSEVERADSLYAALTNVQRAVSRTQAQYEQ
QMRERTESATADMIVAQRAAERAEEAKRRAEERLSLVEQELEEQRAELRGLQEANRDLGL
ENNRLSRMLPNSHRGPHEPGSATARRGFGDVDQAEEAQMLHVATMNVELMQTRRRVRELE
ANDATLRRAYTVLETQLEELRDEVGENERLRGELDRLREEHADLALRYDALVETTDATEG
GVAQELKSLRQQRSLLEAELAEWKARYRDLNLARNLPKVEASQREAALSEQLRGLTSELD
AAQAQY----------GGDAAQARLAHLEASNREKEARLQQQAAERLRDQSEIEALEDEV
ARKTAALEEAERLAAQYARQVDSLTTNAKALRRSTQVGRATTSPENPPATKRTK
>StriCul
PYQTSRVPTEGVEGGGDLEGPISVRDAPLTAAEGDELAHKTPAEVAAYVERLRSNYHYFG
EQLRAACERAQHYKSEVARLREELETRRLQKQQFDVDVEAQDGVQHALEDQVRRLRGQLE
EQEGLIYSLRQELVAR-SAQTSTAIMWYEAQRVRAAEEEARETQQALRDLAAE--------
--SSKAQVRALEVQQQQQQQQQQPSELARRLQDLERENAQLRQSLAAEREEH--------
------EQQKQQLEEALAAQRAAQVQLQADTDAMALALTRRAPLSLDEYDAQQQQLAALQ
REVEAQTAEAAQRKAAVAPLEATALELTELRQRVAAAAHDRTTLERNCHQRDEEIVRLQE
LLQCYKTENLAAQEVSRVRTEQYEHTLLEQVDAAEAARAAEEAVDDLQRQLQGERQGLVA
EVKAAELESTLRATNARMEEQEQLIEELEAALQAAADQEEALLLQVQELAERGAELAAEK
AQHLLQVEALKKTLEDTATSYNDRIVVEERLRDRMEEYRQLAAARQLELRTAAAVWLQAT
ESLRDGLRVAEAAAGGPATPPLKLQPLEKAAAWYADVALRETAAEAEVGDQLRQLWELGR
AAARREETLRRKLELEQQLRAALDTLAVENVDLKEAGRNYQSLFNETQAALQQITNDFQQ
```

```
QLLEEKEAASVEVVTARRDMQRAGESRRRAEQRAQLLQEELEQLQHVKGELEGQLLAMSE
ERDELRTRATETVEAVLGSDLSVRASRYSTAMAQEQAQVLQVTSLQSDLLLVRRQLREME
AQDAALRRSAAASEREVAALRT---ERVRLQREL--ER-EHVTTQRRYSQLLEQTDA--G
AAAEELRELRLKVRELEGVVFRFEERERELARVRSQQEVQAGRRQEAVLTRLRSMSSRMT
DVRRQL-RGGLTLATDPQ--LVAEVQQLQ-------HDLENYEKLAVEHHLRLESLQAQL
AAEREARAMADKAVRQLSRHNERLRTTMQSERQSTLLALSSKAGRATGSRKRMR
>StriGal
PYQTNRNPTSTGEGVSSLEGPISVRQAPLTAAEGEGLEHKTPAEVKAYVLRLRSNYHYFG
DQLREACERAQHYKLEVARLREALETQRLQKQRYNLDAEAQDSVQHALEEQVRRLRGQLE
EQEGLILALRQQLLAR-SSEQTTSIMRYEAERVKASEELRSAQQGLRDLAAD--------
--RSKAQVRALEVQQQ----QQQPSELALRLKELEKENAQLQESLAAERAEH--------
------EVVKQRLEESLAT-------LQADTDTMALALTKRAPISLEEYDAQQRRLAGQQ
RQLEELQVEVEQCKAAVAPLEATALELNELRQHVAAASHEKAALQREGQRKDEELTRLQE
LLQCYKAETDAAEEANRVRTEKYEAALVEQVEAAEAAREAEAAVAELRQQLAQAQAQTQT
QMKAAQLEATLRADNERLEEQEGLIEELEAALRTSGEEQQRLVQQVQELADRGTELAAEK
ERLELEAEAFKKTLDDTARSYNDRIVVEDRLRDRVEQYRQLAAARQLELRTAAAVWLQAT
ESVRDGLREVESGAAGRTAPHLPLQPLQEAVAWYVRVALREPPVEQEVGDTLRQLCEAGR
AAARREETLARKLELEQQLRAALDTLAVENLDLKEAGRNYLTLFNETQAALQQITNDFQQ
QLLEEKEAASLEVITARRDMERAEEARRRAEERLQLTREELAQLQEANRELEGQLIEIGE
ERDELRTRVTVANEAMRGGDIHVRASRYSAAVAQEQAQMLQVTSLQSDLLLARRQLRELE
AQESALRRSATLAEREAVVLRT---ENTRLQKELVQER-EHTTALKRYSQLLDQTDI--G
AAAEELRQLRLKVRELEGVVFRFEEKERALARSRSQQEVQAGRRQEAVLARLRSMSSRMT
DVRKQLRRGSLTLATDPQ--LVAEVKHLQ-------HDLETYEKLAVEHQLQLESLQAQL
SQEREARTTADKTVQQLSRHNEQLRQTLGHERQSAQLALSITPVKKTESRKRNR
>StriOnc
PHQTDRN--TTPQGVSSLEGPISVREAPLTAAEGEALDHKTPAEVAAYVQRLRANYHYFG
EQLREACERAQHYKAEVARLREALETQRLQRQKFDIDCEAQDGVQHALEEQVRRLRGQLE
EQEGLLLRLRQQLAAR-GAEQSTSIMRYEVERVKAAEEARWAQQAMRDLAAE--------
--RSKAQVKALEV--QQQQEQQRPAELARQLAALEKENAQLHQSLAAEREEH--------
------ETQKQQLEEALAA-------LQADTDTMALALTKRAPISLEEYEAQQQRLAALQ
RQIEQLATETAAQKAAVAPLEATALEVTELRQHVAAAGHEKAALERAGQRKDEEITRLLE
LLQCYKTEADAAQEANRVRTERYEAALVEQVEVAEAARAAETALALLRQQLEEAQASAQH
QEKAAQLEATLRADKERLEEQEGLIAELEEALRTSGEQQEQLVEQVQRLADRATALTAER
ERLQMEADALRKTLEDTARSYNDRIVVEERVRDRAKRQTQLAAARQLELRTAAAVWLQAT
ESIRDSLLAVEGAPDDRPAPALPLEPLREAVAWYARVALREPAIEAEVGDRLRLLWEAGR
AAARREESLQRQLALEQQLRAALDVLAAENLDLKEAGRNYLSLFNETQAAVQQITNDFQQ
QLLEEKEASSLEVITARRDMERAEEARRRAEQRLELTLEELEQLQSANRELEGQLVAVGE
ERDVLRTRVTVEREALL--DVNVRPSRYSAAVAQEQAQMLQVTSLQSDLLLARRQLREME
AQETALRRSLAQAERETVVLRA---EATRLQEELVQER-EQTVALKRYSQLLDQSDV--G
AAVEELRQLRLKVRELEGVVFRFEEKERELARVQSQQEVQAGRRQEAVLARLRSMSSRMT
DVRRQL--GGLSLATDPQ--LVAEVQHLQ-------QDLENYEKLAVEHHLRVESLQAQL
THEREARTTADRAVQQLTRSNEQLRLTLGRERQSLQLGLSVTP-KLAASHKRTR
>Pserp
-----------------------------FSPLEPRGLDDLDENELYNVASRLQDEARHAR
AHIARAYEMRDQYMEEVARLKLELHNKYLQTAELERGRGDYEDTIQSLREDIQKLRRELD
NAEGREQAMRLRLAAGVGSDVAESVKYYKTEITTKNQQILQMKEHIEMLEKQKEQHAQTP
ESSKSPSRSAAHTHSEESSSGQDSDQLLQQIAQLEMANAKLVQELEEQTTIQLEAERNQM
ELKTTSEDRIVQLEKEIEKLRELNTSLQREVDTLAPVLANQAPLTRGAYETFQEEYESLK
IRFSELEAEYHSLEREGRVLAAAALETDMLRESLTRLRLEHRDTTEQLKLKNE---KMVE
LIATFEEETKLSRTANDLRKVQVETLHKQIEELNN--------------ELKALRNLNTQ
EQRCAELSEKLEDACRRA---KAAELDKAQQAKEFNEYRKNTESRITSLLSEVSVRSSEI
EHLRLSLLTTQNSLEKVTVMLNDHMELDDSRQSHMKVMRESLMNKDNSLRYAVTHLCDVI
NAFSCFTLDVNRFVKSPVVFDACLEPVAEALRWATKTHDTLNQVAEKALDVVACVRDVVS
RADRLANSLQEERERSSSLMAAMDQIATDLLDEKDRSDSLFSTIADINQQLEKTKAYFDE
QMRQQAAASSVELIAARRAEGRAQEAKRRAEEQLALVEAELEEERMALRKLRDSYSELKV
EAERLTRLTSSISDANQTDASRHIGLRDARLEVLEQAQMLQISTLQAELLQTRKMVREAE
```

```
SREAVLSQRNRILEQTLTEQHEDVQTATTLRIDLAQLQSEYTDLLERYKNLVESSSTAEI
STIEELKQLRRQVRLLETELTEEKAHNRALELAKNTPGLEAMLRQQALGETLTGLSTHLA
AVQAQYEGAILRTPNPSTDLLQSRIAHLETVLKEKEKAMEKYASDNLRQQAEIEALEDNL
ASKLVALNRTEQLNEQYLGTIDALARSSKALRTSAAVRLSSSLAQTLPRRKRGR
>Phart1
HPASTK---------PDLPRPISVRDLASTSLAACSLDEMNEGELYTFASRMQDEARHAR
GHIAQAYELRDQYIQEVARLKQELHSKYLQTAELERNRGKYEDTIQSLREDIQKLRRELD
ASEGREQVMRLRLAAGTGSDIPDGVKYYQTELTTKNQQIQHLKEHIETLEKQNEQRIQTP
ESSKSPSRSAANANSEEPGICWDPNELLRQISQLEATNSKLRQDLEEQSTIQLEAERSRS
ELQASSQTRINNLEEEAEQLRTLNISLQQEVDMLTITLANQAPLTRGAYETFQDENETLK
IRFSDLEAKYQSLERENLVLAAAALETDMLRESLTKVRLEHRDTTEQLKLKNE---KLLE
LVSTFEEETKLSMTANNLRKTQVESFHKQIEELTS--------------ELNALQSLNEK
KE----------------------------------TYRSNAENIIASLKSQISLLSAEL
SDHGFLLPVSQESIGPSDTILHDSIQFDDSSLVHLTTIKASSVQRNYSLQYALALLRDVI
SAFCCLTSDLNRFVKPSGAFEACLEPVAMASRWAAQMQDILKKNSRKASDIVVCLRDLLS
RVDSLVYSLQEEKEHSRSLSAAMDQIAADILDEKERSDSLFGTIADINQQLEKTKADFDE
QIRKQEVASSVELIAARRAEGRALEAKRRAEEQLVLVETELEEERMALRKLRDNYGELKI
EAERLTRLTGSISDANQTDTNHQIGLRDDQLEVLEQAQILQISTLQAELLQMRKMVREAE
SREAVLNQKNRVQEQTLAEQHEEVHKTAALLMDFDQLQSEYTDLLNRYKNLVESSSTTEI
STIEELKQLRRQVRLLETELAEEKAHTRAIELAKNTPDLEAMLRQEALGETLTGLSTHLA
AVQAQYDDTIFRTPNPSIDLLHSRITQLESSIKEKNKKVEKYAADILRQQTVIETLEDNL
TNKLTTLDRTEQLNEQYLSTIDALTRSSKALRASAPVRLVTSSARILTKRKRGR
>Pem1
HSGSAKK--------PDLPRPISVRDLASASLTARGLDDMDEGELYNLASRLQDEARHAR
GHIARAYELRDQYIEEVARLKQELHSKYLQTTELERNRRDYLDTIQSLREDIQKLRRELD
VSEEREQAMRLRLAAGAGSDVSESVKYYQTEITTKNKQIQQMKEHIEMLEKQKEQHIQTP
ESSKSPSRSAANAHSEELSTGPDLDQLLQQIAQLEGANAKLVQELEEQSTIQLESERTQS
ELKMASQTRIVELETEVMQLRELNNSLQREVDTLAPALANQAPLTRGAYETFQEEHESLK
MRFSELEIEYHSLECEARVLAATALETDMLRESLTKLRLEHRDTTEQLKLKNE---KLVE
LIATFEEETNLSRTANDLRKAQVESLHKQIEDLTS--------------ELRTLRNHSTH
EQXXXXXXXXXXXXXXXX---XXXXXXXXXXXXXFGEYRSNAESLIASLKSEVSLRLSEI
EDLRLSILASQKSLEQVTSMYNDCIELDDSRQEALKAMRESFEDRHNSLRYAVAYLRDVI
SAFSRLTLDVNCFVKSSVVFDACLQPVAEASRWAAQTHDTLHKVVQKALDVIACVQDVVS
RADRLAHSLQEEKERSRSLTAAMDQIAADLLDEKERSDSLFSTISDINQQLEKTKTYFDE
QMRQQAAAASVELIAARRAEGRAHEAKRRAEEQLVLVEAELEEERMALRKLRDTYGELKV
EAERLTRMTSSISDVNQTDASRHIGLRDARLEVLEQAQMLQISTLQAELLLTRKMVREAE
SREAILSQRNRILEQTLTEQHEDVQTTTTLRVELAQLQSEYTDLLERYKNLVESSSTAEI
SAIEELKQLRRQVRLLETELTEEKAHTRALELAKNTPGLEAMLRQQALGETLTGLSTHLA
AVQAQYEGANLRTPNPSIDLLQSRIAHLETVLKEKDKAMEKYASDNLRQQAEIEALEDNL
ASKLVALNRTEQLNEQYLGTIDALARSSKALRASAAVRLSSSLAQTLQRRKRGR
>Tbru
PGFFTRTWTPPPENIPRLAAPINVRGLATIFAGRRGLADMSEEERMEYTNRLEGDMTHVH
NTLSRAYQLRDDYKNEAARLHRELQDKNHRFDCLLREHSACNDVIYRCKRENEELRQKLD

ESEGEVRQLRDKLVSV-NSQGKYVPSGGERHV--GRQEISALEEKNKKLEEERERECIRH
HA---------MGKSENTSHEEELAQSRYLLQVTRTEITDLQQLLRKEREDYEESLREAI
QARNNLHQQNTALQEQKEQLQEMCDEQHRTIEDLTSQLLQRKTEQAVQRGAPDTQMETTD
ENKTDT-----NTNNDDEVYRMLELQQHTLQQQFFLLRREGEAKDILLQKASEEIFNLQN
LQQQLEAALQKSREHAAELTKSLSHTQNQLQTAQERITEDSYVINNFHHQLREKIQISGS
ISTTKKMSAERELHVTKLTQLEETVSRLESYGTTPEQTVAAFTTELQHTQQRLREAEEEI
IQLTNKLNAAGVRVRTS-----------QSDKDGNARAALVSDVAVRNADTDLG--TQLA
SAL--------------VALERLAEEREAALEKATEMEERVSTLEEELRTAKEKLERSVE
EISFLKDEVLVSNRLLVDSVSSLN---GKDGAVGADVERLSRVVDELHAQVSATKRGFEE
FYDRRSEGCVTELIVARRSVDRSNDARRRLEERNVRLEQDLERKCLEVVKLQKECQRLEQ
FVRAKDVRGAHSVLGVDSVDVSSVGAEPVDLEAVDLAQFLQISSLHADLMLCRKTCRQLE
SNQEELLLS---LEQNSSQSNAYLEDLDEIRQQLVEMRQQREELIAERRTLTERVDELGR
```

ERGEEVSRLKQQNNSLSAQLQASRNKLSALEASKREGELAARQQAEELAKAFSLMEAQVQ
TLREEVQKAVVEGDEARIRMSQARVTFLEKALQRKDEEVQRLQDELVQKDEQLDQYEQDA
AKAAQDAENASRKTLQLESAVQKLQGDKKGLEDELRYAKTRQVMDIRSTRKRSR
>Tcongo
PGFFTRTWAPPPDSPPRLAPPINVRGLASGYMCRRGLNDMSHEERLEYTSQLDGDMKHVS
SALDRAHQLRDEYKNEAARLHRELMEKQRSLDRVVREYGECNKVLHRFRTENEELQQKLD
QSQGEVKHLRSVIQSA-NVQQELKIGAGQLLGDKGNQERIQLEESNRRLERQKEQAYNRR
WKT--------ATTTDQDSLAVRVEQGRLSLNVAMAEIDDLKNELQRKNDEYSRRLEQLT
NERNSLHQGHLTLSETLEKLEDTCKEQQQTIEDLTVRLLQEQSTNRKERNSHNEQHIGYQ
HVHSEE-----NTDQLRAKLCAIELEKHALQQQLFHLRQEGERKDEIIKKANTDIRKLKD
SETAMSRKIGEVWKQNEKLQERIEETTRQLHISQNQNEEDQYVIKNFHKRLEEQIKLTHN
TQATESLAAERNAHATKLKELEGTIARLEGCGDSSAKVIEALKSELAHTQKRLREAEQDC
ARLT-----------------------------RTDAVVKERDAAVGAPSSDLA--TQLS
SAL--------------AALNRLAEEREIALEEDAFEGPIVDVLKRDILFLKSSLGECRR
YISGLNDLLSCCDDGSSSMVNLFNRVGGGLPNKCDGTATL------------GATRVMQL
GSDCVDEVSVADLVAARRSLQRSNDARRRLEERNIELERELEKRSMDIAGLEKECRDLEK
QVRARG--SSYMRSIHDECDFRSSVSGGVCMETIDLAQFLQISSLQADLMLSRRTCRQLE
SNQEELLLS---VEQGVAQSQNLLESVEELRMEVHNLRRARDELLCERRLLTERVNDLEL
EREEDVEVLRKQNRWLTSQIEEAGDKLSAAEKSRREAELAWEKQAEELIKAFNDLNAQMT
HLRSE-RSAVDSEDETLNQVRNARISFLETTILRKDGDIYRLQEELIKKEDNIDKLQREI
IRMSNKIKNATRKSEKLESNLQQLHEKKEEMNKELYLLKTQEKIPKRESRKRGR
>Tvivax
PGFFTRTWTPPPEGVPRLAPPIHVRGLTTIRQCRRAVTEMSREEQSNYIMRLEEDIRQMS
NALHSVYQVRDHYKGEAARLHKELMDRQRSFDRLEQEHKNCRDVIHQQRNTEQELRERLD
ASQTEVRYLKSLVGSS-GAQQKATCANQAHEIEKKREEILELQRQNKALEVQKEQTLAFQ
QQ---------ASVAAWERGRRELKDTENLIKVMGAEMNDLREQIQKERVQHEESLWAVT
QERNNLQMLKMSLAEEVDRLRELCQAQSGTIDSLNETILKRPALSDHDEEAVQNTIANLN
NEGQ-------SLIRDRAVETTSDLEKQTLLHSMCALRQDWEDSNSALQRAHIEMAQFKK
HITTLEDEISTSKQEIANYEQKLIEAQIQLSEAKETSRESESVIRDFKKMHEDQNATIYE
LRTISEMAAERDAHAAKLAELSDTVSRLEGVETVPEHAVEALCAELRETQARLREAEEQV
ARLTAEANAGATAASGS------------------ESASAAVDVRSTAEYVSVA--SQLS
AAL--------------AALDKLAEEREALDQRVVELSSDLERVQSDKTSYYLSMVDLNN
EVSDLRLQLKNNDVAVTEILSCLGDVSAEGGNSSGDSSALLKAIEDLYSRLSSAKRNFEL
YSTCNSETGVTELIAARRSADRMNTARRHA------------XXXXXXXXMNQECLFLRR
ELRLHGICFSYNSLDVEGSFASRLSAANVNDECSDETQLLQISSLQAELLLCRKRCRRFE
VNQEELLTT---LEHHVSQAEVSATNMAELRLDLAQLRLQFTQLQEEHSGLQDRFEDVNH
SQLDELDRLRLQNDRLLCDLKEKSEKISAMEKEMRDAERSAKGRVNELLESLCIMEAQMG
DLLKGASSESQQSDRVQGRALAARVSFLEQTSKRKEEEIQRLQDELLRKEDQLDQLEQRV
TDSTQELDNAVKKVTHMESVVQRLQSAKDSLQEENRLLRAARTSGLTQARKRPR
>Tcruzi
PGFFTRTWTPPPD-VPRLATPIGVRGLAATHASRHGWEDMPRGERMNYAARLEDDVAQMQ
NALDRAYHLRDHYKEEAARVKNELMQKQRALDCLERDHRQCGEVICRHEREAVDLRHRLD
CAEGEVQRLKYLQRSE-DPNVRVPLLDAQRELRLKQTEVDSLLEEKRQLEEHGEHYAARV
HERP-------PNVAHVEEGGEELTRAQHVIQVLCTELQDMRQQLQNERCRNEGTLDEAI
QQRNGLQGVNTSLREELEGLKEMCCSQNRTIDELRDTLLQRAPVTHHEHYALVRRCEKLK
EEIERC-----QTRGGDSVNKAHELEKQAIQQQLLVLRADGEETNTLLLKTRADVADMRE
YISTLETELDLTRKAKTLLEEELAAAKEKLQYATEQNKEAEQVIVNFNRRLQDQFSTIQV
QKAIDQLSAERSAQAERVAELTAAVARLESSADAPERVVEALSAELRETQDRLRQAEEEA
SQLAERIDSSEGLRESA--------------------------VATRSAETSLA--VRLS
SAL--------------KALEQLAEERETLARGSAELEERVRELEQALRDAENSLCVREG
ELLKLGEECSAKSALLSNALAAVDQVVQEETSFEKQTSSLVKTFEVLHGQFLLTKKGFEE
QWERRAEACTAELLAARRSAQRANEARKRAEENAAICMRKVEEVNAELMKAQRDYRALER
LLRSSGRLSISRVENNDEMSFTPTFLR--NTEGAELAQFLQISSLQADLMLSRSTCHRLE
ARQLELQTA---LEQCEWRLASLPPGVEESHAELLRVRGQLENHQLEYHRLEGRYKALKR
QRAEEVEQLQRQNERLVQQLREKRDKIGSLSRQMRDSEVAAKQQAEELAQAFNLLETQMQ
ALREEVRQGVEETTEAEGKMLRGRITFLEHTLRQKETEMHRLQEELGRNEAQLDQFEDQV

TVATQRLENTARRSTHLEQKVHELKRTNQELREELGAVRA-----PRAATKRPR
>Tcara
SSFFSRSWTPPPDGVPRLGAPIHVRGLV--------------DDVLNYTARLEEDVGHVN
NTLDRAYHLRDHYKEEAARLHKELMRKQQMLDSLQCDHQHCGGMILSLRQEIDSLRQLLD
EAEGEVQRLKIALASD-GHQGGLSVMYCKSELVRRETEIENLQVENEKLKKDVKKRV---
-----------SDTSDAKSNVDETQQSEITIKALCDELRDLKSQIENERQEYEGRINSLT
VERNALSAEVTSLNERLHSTRTLCQTHADTVDELRESFIQRSSVTQQEYVVLQRRYKEIQ
EELESQRHSDTQNXXXSNLESTLKLENQNLQLQIIILRQSEMEKEELLHRLQKDLDKQQS
LSQNLQNELDLLHKTHALQKTHLLDTEKKLIQSQKDNEEANRVLTDFRRRLESDCSTVNL
AARADGIIDEADALRSRLAXXNNLILLLEQYANIPEDVIGALAAELRDTQDRLRVAEGER
ATLASNRRVTMGSVERN-------------DRGSLVEKDLRYDVSSGCVGDDLSLRSQLA
TAL--------------AAIRRLGEDYDTMCEKYRSSEGQVSLLKMDLLAERRSLAEVNE
ISVHLREEIDMKSRALAAALVALDRVSAEEVAQEERSANLLSTFEALYAQFEATKRSLEE
QRTRNAEASAAELIILRRNVQRAEEARRRAEDHLAECELELEREHEAHLRLQREHSTLER

MSRSRGDRASEPMSESTAVDESPNLVAPVQAEYVDHTQFLQISSLQADLMLTRRRCRQLE
GIREEMQSE---LERRGTVGGN--DTVSAARAELRRLRGQFEQLRVEHRVLQDQHNTMLR
DHDAALKDLLEQNTRLTHELGENQNALASLAKHNHSQQAPTRHESEALDRAFRVLDAQMH
VLREEVRSASAPRRSAPPD--NARVAALQQAVRRGEEEIQRVQEELERREEEVDALEEEL
VSATERQNSSKRSPLHYEEIVEQLQNANEGPQESLRQVSVTSSSRPTEYRKRSR
>Tthei
PGFFTRTWTPPPEGIPRLVTPISVRGLADTQRCRSGWDDMTPEERMDYTARLESDMSNVK
NTLDRAYHLRDHYKEEAARLRGEVMQKQALFDRLERDHRKCSEVIRSHQLEVQELRRQLD
LSEGEVYRLKQVLQSK-GENEFFSPMDYERRFSQNETEMARLQEENRSLENQAKSSLTVQ
KTQVDILDESLQKMGQYEEKEEQLAQSQHLIQVLRAEMQDLRQQLQKERLDNESSLNELI
TQRNSTQSINTSLREEKEALDDLCRRQNKEIQELREDLLQRAPVSHQDYHALTKRYEELK
EEIEHL-----KEKHDNSLDIALELEKQTLQQQLFNLRHEGEEKERLLQNSLKDASRLRE
LNENFQKELELSRMTKELLEKELSKTKKELQSTEQSNREAENIIEDLNKRVSTQLSTINQ
LT------------------------------------------EVAETQLRTRDTERRQ
SATADREAA----------------------------------EAAREHDAALS--EQLS
SAL--------------TALERLAEEREEAAEKALQMEACIASLEESLHELRMLLSESRN
EALSLREDLDVKGKLLSDALSALDRVGADEIEWGERSERFLRTFEELQSQFAVTKKGFEE
QWQRRSEACTAELIAARRSAHRATEARKRAEERAALCMQEWEQMHGELLRLQREYRVVER
IVRAHGLYSTPHVDDIDESGETPPGVRPWNSEGVDLAQFLQISSLQADLMLSRKKCRLLE
SRQEELQAA---LERSELQLASLPASVEESHIELRRVRDQHETLLHEHQRLEEKYEAVKK
QRIEEVEQLRQQSERLMQQLREKRDKLTTLTRHMRESELSAKQQAEELSDAFNVLDGQMR
LLRSEVQHNEKVVADAETKMLQGRIKFLQRTLQQKEEEIRRLQEELERKEELLDSFEDQV
TSATHDLENTSRKSAQLEQTVHHLKRNNLELQEEIRILKSHQTESERRGRKRSR
>Tgray
PGFFTRTWSAPPDDIPHLAAPIHVRGLAAVLHNRPGWEEMSQAERMDYTARLEGDVSQAK
DALDRAYHLRDHYKEEAARLHRELMRKHGSLERIERDHQLCGDVIYRHQMEAQELRHQLD
EANGEVQRLKALLHTTEEHRGGLSVIEYGVGLEQRQAEVNELREENRRLLDQNERSTARE
REH--------STTTRTAGEEEELKETRHVLQVLRAEMQDLRQQMQQERLDHESSLEEVI
KQRNSLQGLNTSLREEMEGLQTLCRAQNMTNEELQETLLQRASVTHHEYHALNRRCEELQ
KHVELL-----QQEQSPSLDTTLELEKQTLQEQLLTLRREGEEKNEIIRKSNAEIDTMRQ
FTSNLEVELDLSRRAKTLIEQDLLVTKEQLLSLQARCQESDNVIAAFHVRLEGQMSTIQA
LN---------------------------------------AEXXXRETQDRLREAEDEL
RAAAELRKA---------------------------------------------------
-----------------AALGRFADEREILNDRAAGLVDRVAALEEELDETSIRASVDSS
TVVRLREELNVKSRLLADVLAALDRLTVEEVSYDERTENLLKTFEALQQQFEVTKKTFDE
QWERKSEACTAELIAARRSAQRAIEARKRAEERAAQCARDLEREHSELLRVQRECRALER
GVRIQSRVLTSHDEVFEDTDVIQSRRRVSNAEGLDLAQFLQISSLQAELMLARRHIRQLE
ARQEELQLV---LEKTEWELASLPASAEESIVELRWVRAQHKDLQVEHRRLQEKFDALKN
QRAEELEEMRQQNERLMQQLRERRDKLASVSRQMRESELAAKEQAEELSQAFNLLDTQMR
ALRAEVRHSTKEPEDAQGKLLQGRITFLEHTLKQKEAEIHHLQQELERKEEQLDNLEEQV
GSATRNVESTTRRSALLEKTLEQLRRTNDDLQDELRVVKGRRTPRATSDRKRSR

>Paratryp
GMFFTRA-----RATPWLARPVRDRSGSFSEFRPSALDMLTSDELRSYASRLEDNVGIAS
DNLQQAFSLRDSYKDEAARLRAELDHQRLLRKRYDRDIERYKEISSSQQRDIAFLKQKLD
EANGTVRLLQGQLDAAH-------SVKYKEEIRLRDASLEHATNTISELQEARVETL---
----------------ELELRAAQVELRESVQRNHELEAALHSSQRDAKDGDITRLKHEM
DRLRAVDHENTQLRSQLQQVDKHSSAQKQYIEQLLDTLQHRSTLTVEEEARLLQHVADIT
KELQRAHTTLKDKCHNVDLALAMNLISQTQISLLEAKQQEASADDNAAHRSHDTIHNLME
YVTSLERQI--------VDQAKLLENYENPLQTKDEKEPSSNRHKQNKNKPPTTTTCSQH
SDKVERIRDDAAAELGAQASSVWLMKAALRNTGTLTAEEEAIVRSV--TRDSLGVSSDER
LR-----------------------------------------RSGIVTRFSDVHGTGLT
AELRDVL--------------------------------------QQIEQLAADRAYHEE
CVQSLREEVAAKGAALQRALEALGQASDEEQVAHERSEKILTTLSTLQSEFQRMKERLDP
RPSPRSSAVMMDLVEAKRAVSRANEGRRLAERLLDDREGELHLLRQSLAEVTAEKVRIEA
ELRAVNSQSSQAADDPAASAAAVWQQQQRAMNVLEQSHVLQLSTLHAELHLTRQEAASLR
QRAAQVREKHSLLTEANTRLEEEVKTNDRVQEELVQ-------MCEVYEAQLQAARLSDA
SSEEALKQLRLKYDRVCSDLSAARDSLARLEREKREAEIDARSQAEEFAKTFRNVEAQLH
ALRREMRPPRASALSPDATRLNDTVRDLQEALASRDEEVSQLQQLLALREKEIAQLEADL
KRAVRALETVSDGEQEALRLAARLMRLQAAEVKLADILAALGARRSGVAKKRPR
>Angom
QRLAAQNQSTPSAGR-------------PTPAVRPQLDLMTRDDLYNECAKLRWSFDNAQ
ELRERSEQGRIYYRDLVAKYQAEKDLLNSRLQSAIEAKSRAEEETGNYRRSCESLRNQLN
DLEEEFRRAKLDLDRAHGAQVV--VGRMKEDLREKDACIMDLSRQNSELEAEPADQAAQP
PASLSEYPRSTYDNDP-----LARQRLEDELQAAREELDQLRNTLDDERTAAERELQHA-
------RSESSQMGHELENALRNSDALQETIDQLAAELAKRAPVSLSDWEDYQQKYSDLL
QKNKQSESTVR---------------------QLERQLENGPTPNEEELAINE---NKDQ
LIQTYSEEVELRAKKEEALLQELEAVSAQLQQSTKEQEASEQEKLDLEEQLRELQKQSSD
DQENDSLMAYSQKLLERSEELVALLEEKTQLCDVSVSNYNALRRSLEEECLKSVTLEGEK
KDLLAEVASLTSQLEETAEQYAEKLLDVEYEADRKAAWRKVADRRLAQLKELLADYKMSV
ESIVEALRYTLQV--KENSSALVLSHVEAALTRADALRTNLNTIADNLENFSQETAAALT
EYKESLQTISEMQVRMAQQKAAIDVLAERGYDMDEAWKRIENDI--------RVETEANR
RHQEHRDLLLFELESANKARERAEEARREADERATLLESDLQVMR---------------
------------------------------------------------------------
------------LQQGDAASGPSSEEVEQLRSRYAQLAADYETAMNRLQLLERSGVERAG
DLYQQLLETEARCQVIQRELDLYKARRAALAKSNTLAKYEEMRYSDTLHTNLDKVQSLRA
TFGASD------AKPSGTDPLSKLQDALQRNLQ------------LEQEQIEWKSRYERV
EEKLHAIEEENNRLSRLSGVVERLSTSQRAPTLTRSMP--SRSFKSPQSRKRTR
>AngDes
----------------------------PPPAPRPALETMTREDLYNECSKLRWSFDDAL
ERRERSEQAKQYYRDLVGKYQSELERMQDQKGEFLEAKRKAEEETERYKQSCDSLRRQLE
DMDEALYEARAERDRLGGAETV--VESLKGELRERDAAVFDLSRENERLEKERQ------
--AASPSPTAAPVDPF-----LAQDRLDSEVEELRDTVEALQAKLAETQEQHAAELRQEA
EKVDQRKRELDQNKDDMENLLKRNAELQEVTDNLATQLSKRAPISSEDWKQFQNDYDNLA
KKIKSLEEAEK---------------------KATTGLD----------AVNR---QQQD
ELDSYKKEVELYQQKEEALLKEISNLQAQHQST---------------------------
--EGVEVKKYGEELLEKSEMLATLLEQRSRECNEHLANAVSLKEVLEEECLRNATLDQEL
KEKESALAALKEQLEDTVALYEEKLLEVEGATEKCETWRKTAEKRFQDSYRMAKEVETCV
VGLVEAVGCATDP--TKTSSALSLKNVEGAVKNIRDTLAKMDTAAPAADAFSSKMNDAVS
LCKKHISEIEEMQVRLTHLTAAVDVLAERGYDMGEAWKKIEQEI--------QTEAEANR
RNQEHRDLLLFELDSANKAREKMDEARREADERATLLESEVQVLR---------------
------------------------------------------------------------
------------LQQSDAR-SVSPGELDRLRARNAQLVADYEIAVDRLRVLERAGGRHGE
DLYQQLVEAEARCQVLQRELDLYKERRSALAKSNTLAKYDGLRYSHSLQSNLHKMQDLRA
AFDPSA--PHKSTSKDPTEIAIQRNEALE------------------QERIELQTRYSRA
EEKLSVLEEENRRLAQLAGVAERLSTSRRGTLLPKDMMP-SPSTQQGKAKKRPR
